# RNA interference: a promising biopesticide strategy against the African Sweetpotato Weevil *Cylas brunneus*

**DOI:** 10.1038/srep38836

**Published:** 2016-12-12

**Authors:** Olivier Christiaens, Katterinne Prentice, Ine Pertry, Marc Ghislain, Ana Bailey, Chuck Niblett, Godelieve Gheysen, Guy Smagghe

**Affiliations:** 1 Department of Crop Protection, Faculty of Bioscience Engineering, Ghent University, B-9000 Ghent, Belgium; 2 Department of Molecular Biotechnology, Faculty of Bioscience Engineering, Ghent University, B-9000 Ghent, Belgium; 3 International Potato Center (CIP), Genomics and Biotechnology Program, Nairobi 00603, Kenya; 4 Institute of Plant Biotechnology Outreach, VIB, Technologiepark 3, B-9052 Ghent, Belgium; 5 Venganza Inc., St. Augustine, FL 32080, USA

## Abstract

The African sweetpotato weevil *Cylas brunneus* is one of the most devastating pests affecting the production of sweetpotatoes, an important staple food in Sub-Saharan Africa. Current available control methods against this coleopteran pest are limited. In this study, we analyzed the potential of RNA interference as a novel crop protection strategy against this insect pest. First, the *C. brunneus* transcriptome was sequenced and RNAi functionality was confirmed by successfully silencing the *laccase2* gene. Next, 24 potential target genes were chosen, based on their critical role in vital biological processes. A first screening via injection of gene-specific dsRNAs showed that the dsRNAs were highly toxic for *C. brunneus*. Injected doses of 200ng/mg body weight led to mortality rates of 90% or higher for 14 of the 24 tested genes after 14 days. The three best performing dsRNAs, targeting *prosα2, rps13* and the homolog of *Diabrotica virgifera snf7*, were then used in further feeding trials to investigate RNAi by oral delivery. Different concentrations of dsRNAs mixed with artificial diet were tested and concentrations as low as 1 μg dsRNA/ mL diet led to significant mortality rates higher than 50%.These results proved that dsRNAs targeting essential genes show great potential to control *C. brunneus*.

Sweetpotato *Ipomoea batatas* (L.) Lam. is one of the most important staple foods in Africa. The production of this root crop covers over 3.6 million hectares with an estimated production of nearly 20 million tons^1^ on the African continent. It is important for smallholder farmers, due to its drought tolerance and positive role in food security. Furthermore, the sweetpotato outranks many other high carbohydrate staple foods in terms of vitamin, mineral, dietary fiber and protein content. The production of sweetpotato is often heavily affected by sweetpotato weevils from the *Cylas* genus, which are considered to be the most devastating pests on sweetpotato^2^,^3^. In Africa, *Cylas brunneus* and *Cylas puncticollis* are the major constraint on sweetpotato production, while in Asia and America, it is *Cylas formicarius* which causes most damage. Both *C. brunneus* and *C. puncticollis* are endemic in West- and Central-Africa and are often present together in the same sweetpotato fields. They are very similar morphologically but can be distinguished from each other based on a few characteristics, such as the distance between the eyes*. C. brunneus* have widely separated eyes, while those of *C. puncticollis* are more narrowly separated^4^. Furthermore, they also differ in the number of aedaegal sclerites that can be found^4^. The primary damage to the crop is caused by the larvae living and feeding inside the roots, and to a lesser extent by adults feeding on the upper parts of the crop. However, secondary effects such as the production of unpalatable terpenoids by the plant as a reaction against the weevil feeding, and secondary fungal infections greatly increase the economic damage. In addition, they can give rise to health issues as a recent investigation revealed the accumulation to toxic levels of phytoalexins produced in the healthy parts of damaged tubers which are consumed^5^. These weevil infestations can cause up to 100% yield losses in sweetpotato crops and are therefore of major concern for the food security and economic viability of smallholder farmers in Sub-Saharan Africa^6^,^7^. An effective weevil control strategy would therefore be of major importance. Conventional pest control, using an integrated pest management (IPM) or chemical approach have failed to provide a satisfactory solution so far, and although breeding programs for weevil-resistant sweetpotato varieties are ongoing, this is a slow process due to the plant’s complex genetics (hexaploidy and a high degree of heterozygosity) and due to the fact that only moderate levels of weevil resistance have been found in the plant’s germplasm.

The potential of RNA interference (RNAi), the post-transcriptional silencing mechanism present in eukaryotic cells, in crop protection has been suggested soon after its discovery in 1998 by Fire and Mello^8^,^9^,^10^,^11^. The main principle entails delivery of sequence-specific double stranded RNAs (dsRNA), specifically silencing essential genes in the pest organism, causing a rapid and widespread mortality within the pest population. Over the past decade, research has indeed shown that RNAi-based pest control is a viable option for several species^12^,^13^,^14^,^15^,^16^. Furthermore, RNAi-based pest control is considered an environmentally safe control method, given its species specificity and also the biodegradability of the compounds that elicit the insecticidal effect.

The major drawback of RNAi is that its efficiency seems to be very variable among insects. Species belonging to the order of Coleoptera (beetles, weevils) generally seem to be very sensitive to oral RNAi, while insects belonging to hemipteran and lepidopteran orders exhibit a much lower or a very variable RNAi efficiency^16^,^17^,^18^,^19^,^20^. Recently, a first proof-of-concept experiment has shown that RNAi works in the close relative of *C. brunneus*, namely *C. puncticollis*, at least by microinjection^20^. In this research, we investigate the efficacy and efficiency of oral RNAi in *C. brunneus* and hence investigate whether an RNAi-based approach is a viable option for control of the sweetpotato weevil *C. brunneus*.

Since no genomic or transcriptomic database was available, the transcriptome of *C. brunneus* was first sequenced. Subsequently, the RNAi machinery genes and a list of 24 potential target genes were identified in this transcriptome, based on their critical role in diverse and vital biological processes. Third, RNAi functionality was confirmed by a microinjection experiment targeting the *laccase2* gene. Subsequently, a screening on toxicity of the dsRNAs targeting 24 potential target genes was performed using a microinjection approach in *C. brunneus* larvae. And finally, the most promising target genes were then investigated in oral bioassays for their potential to cause mortality in weevil populations.

## Results

### Transcriptome analysis and identification of the RNAi related genes and potential target genes

After sequencing, the raw transcriptome data (209,736,054 100 bp reads) were assembled into 58,579 components, with an average length of 3580 bp and a 41.1% GC-content, using the Trinity software. The transcriptome data have been uploaded onto Genbank (Accession ID XXX). A homology search using tBlastn was then performed using the available protein databases of *Tribolium castaneum* (14,366 proteins), *Drosophila melanogaster* (27,813 proteins) and *Dendroctonus ponderosae* (2,502 proteins), resulting in protein matches for 83.4%, 83.4% and 98.4% of the *T. castaneum*, *D. melanogaster* and *D. ponderosae* sets of proteins, respectively. The transcriptome was then searched for the RNAi related genes. Homologs were found for all *Tribolium* query sequences, except for the *Sid1*-*b* gene, which has thus far shown to be a gene only present in *Tribolium* and not in other insects. All other expected RNAi-related genes were identified in the *C. brunneus* transcriptome, including the full siRNA-, miRNA and piRNA-pathway core genes, auxiliary factors, antiviral immunity genes and a number of known dsRNases. An overview table of the identified RNAi-related genes in *C. brunneus* and the Blastp scores are given in Supplementary Table S1. The full contigs nucleotide sequences, predicted protein sequences and the alignments between the *C. brunneus* sequences and their *T. castaneum* homologs are presented in Supplementary Data 2.

Next, we identified possible RNAi target genes for which silencing was expected to cause high lethality in treated weevils. The selection was made based on information available in the Database of Essential Genes (http://tubic.tju.edu.cn/deg/)^21^. These include genes involved in key cellular functions. A full list of the selected target genes is presented in Table 1. Additionally, the full nucleotide sequences are given in Supplementary Data 3.

### DsLaccase2 injection disrupts normal cuticle maturation

**Figure 1:**
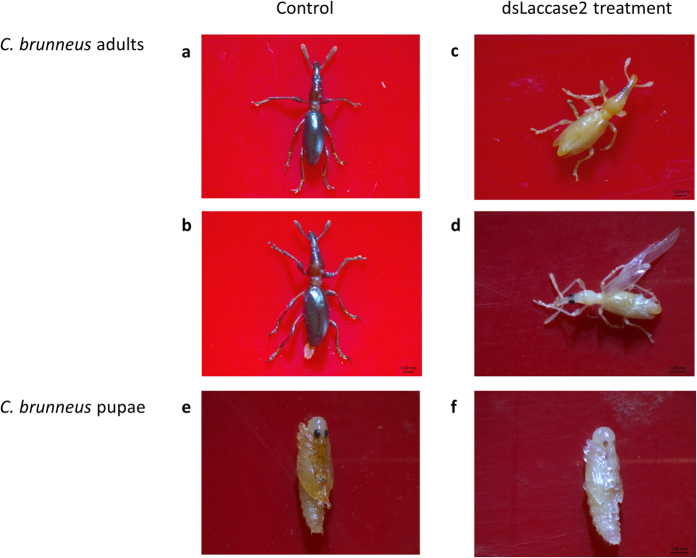
Resulting phenotype after injection of dsRNA targeting the *laccase2* gene in *C. brunneus*. Second instar weevils were injected with 0.2 μg dsRNA/mg body weight and subsequently placed on fresh sweetpotato root slices where they were evaluated for phenotypical changes. (**a,b**) show two adult individuals in the control group, showing a typically brown-black and sclerotized cuticle, including one pair of hindwings and one pair of hardened forewings or elytra; (**c,d**) show two adult individuals after *laccase2* silencing exhibiting a soft, white cuticle, and two pairs of unsclerotized wings, lacking the sclerotized elytra which are typical for coleopteran species. (**e**) shows a control pupa and (**f**) represents a dslaccase2-treated pupa, exhibiting a similar lack of sclerotization of the cuticle.

**Figure 2:**
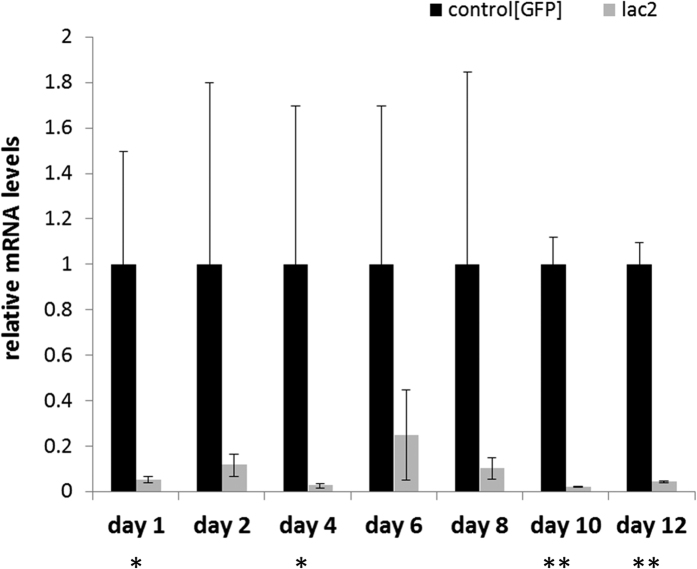
RT-qPCR results showing *laccase2* silencing after microinjection of dslaccase2 (0.2 μg dsRNA/mg body weight). The data was normalized to the expression levels in the control, which were fed dsRNA targeting the green fluorescent protein gene *gfp*. Statistical analysis was performed using the Student’s t-test in qBase+ and showed significant downregulation at 4 of the chosen timepoints (*p < 0.05; **p < 0.005). Error bars represent the SEM.

To further confirm RNAi functionality in *C. brunneus*, dsRNA specific for the *laccase2* gene (dslaccase2) was injected in second-instar larvae, at a dose of 0.2 μg dsRNA/mg body weight (BW) (~0.5 μg dsRNA per larva). These injections caused clear abnormalities in the development of the cuticle, at the moment of adult emergence. The cuticles of the treated adult weevils were softer and also paler, often even completely white (Fig. 1c,d), compared to those in the control group (Fig. 1a,b). In some cases, deformities of the cuticle, which acts as an exoskeleton in insects, or the wings were seen, such as those shown in Fig. 1d. Additionally, we observed that these treated weevils had difficulties walking in a normal way and also had difficulties feeding on the fresh sweetpotato roots. Eventually, after 14 days, 86% of these treated weevils had died compared to 21% mortality in the control. Real time quantitative PCR (RT-qPCR) analysis confirmed gene silencing of *laccase2* on a transcript level (Fig. 2). At 1 day post-injection (dpi), 94.8% silencing (p = 0.0063) was already observed. At subsequent time points, this was 88.3% (p = 0.081), 97.4% (p = 0.0106), 75.1% (p = 0.184), 89.7% (p = 0.137), 98.0% (p = 0.00001) and 95.7% (p = 0.0007) for 2, 4, 6, 8, 10 and 12 dpi, respectively.

### A first microinjection-based screening reveals potential target genes

**Figure 3:**
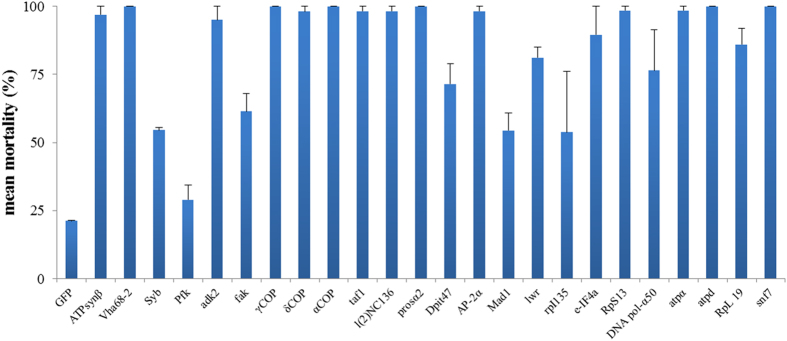
Mortality recorded for the microinjections assays in *C. brunneus*, testing the efficacy of 24 different target genes on day 14 post injection. Second instar weevils were injected with 0.2 μg dsRNA/mg body weight and subsequently placed on fresh sweetpotato root slices where they were evaluated for mortality. The injection experiment was replicated 3-fold, with 10-20 individuals per replicate. The figures give the means of these replicates. Error bars represent the SEM. Control weevils were injected with dsGFP.

Figure 3 gives an overview of the mortality observed in weevils which were injected with 0.2 μg/BW dsRNA specific for the 24 target genes. For most target genes, a strong mortality was observed compared to the control, for which 24 ± 2.9% mortality was recorded. All tested dsRNAs, except those targeting *Syb*, *Pfk*, *Mad1* and *rpl135* caused over 60% mortality and for 14 of them, mortality higher than 90% of the injected individuals was observed. These results show the potential of these dsRNAs for the control of *C. brunneus* and suggest that RNAi in this weevil is highly efficient. Eventually, five of the best performing dsRNAs, targeting *vha68*-*2*, *adk2*, *prosα2, rps13* and *snf7*, causing 100%, 95%, 100%, 99% and 100% mortality respectively, were chosen for oral bioassays.

### Feeding target gene dsRNAs to *C. brunneus* causes significant mortality

**Figure 4:**
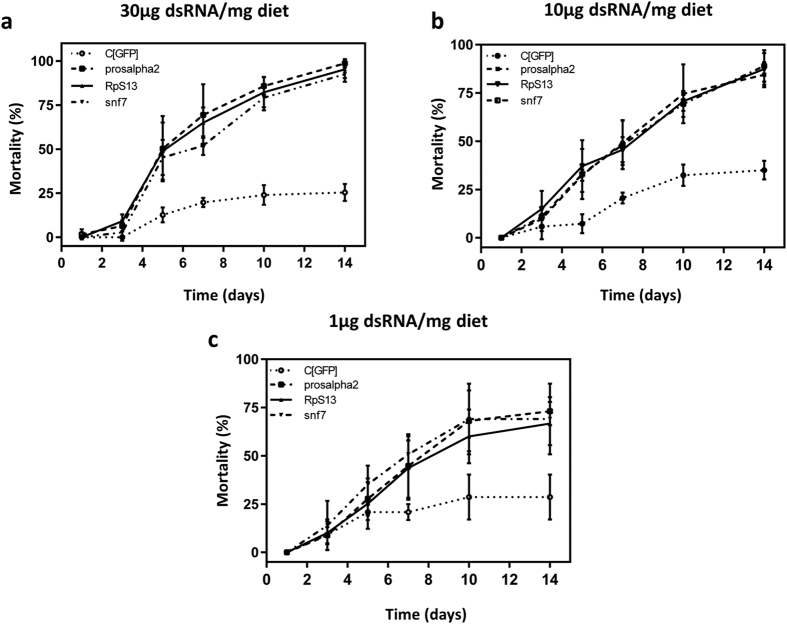
Mortality recorded in *C. brunneus* weevils in the oral bioassays administering dsRNA specific for *prosα2, rps13* and *snf7* at (**a**) 30 μg dsRNA/ml diet, (**b**) 10 μg dsRNA/mL diet and (**c**) 1 μg dsRNA/mL diet. dsGFP was used as a control. Three biological repititions of the experiment were performed, each starting with a sample size of 20–25 individuals per treatment group. The figures give the means of these replicates. Error bars represent standard deviations.

Based on the first oral bioassay results, testing a concentration of 30 μg dsRNA/mL diet, *vha68*-*2* and *adk2* were discarded from the target gene list and were not further tested (data not shown). Figure 4 shows the results for the oral bioassays for the three remaining genes (*prosα2, rps13* and *snf7*), at different time points and for different concentrations of dsRNA fed to the insects. At day 7, a concentration of 30 μg dsRNA/mL diet caused 69.4 ± 17.3%, 65 ± 8.6% and 51.9 ± 5.3% of mortality for *prosα2, rps13* and *snf7*, respectively. Control mortality was 19.7 ± 2.7% (Fig. 4a). For the 10 μg dsRNA/mL diet concentration, this was 49.2 ± 11.6%, 45.6 ± 6.5% and 48.3 ± 12.7%, respectively (Fig. 4b) with 20.6 ± 2.8% mortality for the control. Finally, for 1 μg dsRNA/mL diet, 44.7 ± 16.5%, 43.7 ± 16.4% and 50.8 ± 7.2% of mortality was recorded, respectively (Fig. 4c), with a control mortality of 20.8 ± 4.2% %. At day 7, a significant mortality was recorded for all three dsRNAs at the two highest tested concentrations, compared to the controls (p < 0.05).

At day 14, 30 μg dsRNA/mL diet caused 98.6 ± 2.4%, 95.3 ± 5% and 92.5 ± 4.3% of mortality for *prosα2, rps13* and *snf7*, respectively (Fig. 4a). For the 10 μg dsRNA/mL diet concentration, this was 84.5 ± 6.5%, 87.5 ± 8.3% and 89 ± 8.1%, respectively (Fig. 4b). For the lowest tested concentration, 1 μg/μL dsRNA/mL diet, 73.1 ± 7.3%, 66.7 ± 11.3% and 69.1 ± 18.2% of mortality was recorded for *prosα2, rps13* and *snf7*, respectively (Fig. 4c). Mortalities in the dsGFP control groups at day 14 were 25.4 ± 4.8%, 35.1 ± 4.8% and 28.6 ± 11.7% for the 30 μg/mL diet, 10 μg/mL diet and 1 μg/mL diet, respectively. For each of the tested concentrations, a significantly different mortality between the target genes and the controls was recorded (*p* < *0.05*). The p-values for all statistical comparisons are shown in Supplementary Data 4.

**Figure 5:**
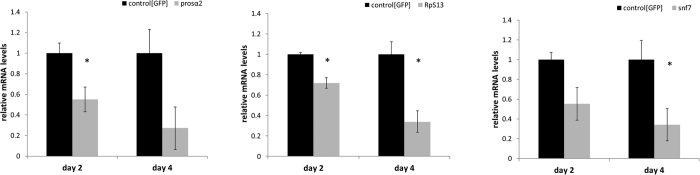
RT-qPCR results for the three genes evaluated in the oral feeding bioassay (1 μg dsRNA/mL diet): *prosα2*, *rps13* and *snf7*. *Prosα2* transcript levels were downregulated by 45% at day 2 after being placed on the dsRNA-containing diet, and by 73% at day 4 after being placed on the dsRNA-containing diet. For *rps13*, gene expression was downregulated by 28% at day 2 and by 66% at day 4after being placed on the dsRNA-containing diet. For snf7, this was 45% and 65%. The data was normalized to the expression levels in the control, which were fed dsRNA targeting the green fluorescent protein gene *gfp*. Statistical analysis was performed using the Student’s t-test in qBase+ and showed significant downregulation for all three genes at the different timepoints (*p < 0.05). Error bars represent the SEM.

To confirm that mortality is accompanied by silencing, RT-qPCR was performed on samples taken from the bioassays (1 μg dsRNA/mL diet) to investigate the transcript levels of the target genes (Fig. 5). For *prosα2*, transcript levels were downregulated by 45% (p = 0.048) at day 2 after being placed on the dsRNA-containing diet, and by 73% (p = 0.098) at day 4 after being placed on the dsRNA-containing diet. For *rps13*, gene expression was downregulated by 28% (p = 0.023) at day 2 and by 66% (p = 0.028) at day 4 after being placed on the dsRNA-containing diet. For *snf7*, this was 45% (p = 0.073) and 65% (p = 0.041). For *rps13*, the downregulation was found to be significant for both time points (p < 0.05), while for *prosα2* and *snf7*, downregulation was significant only for day 2 and only day 4, respectively.

## Discussion

Over the last decades, agricultural practices used to manage pests, diseases and environmental conditions have evolved enormously. The discovery of several synthetic pesticides in the 1940s has allowed farmers to greatly increase productivity of their crops. However, public concern about the environmental consequences of many of these compounds has urged scientists to develop new methods that are more environmentally friendly. In the 1970s and 1980s, the use of biopesticides took flight after the discovery of several control agents of microbial, fungal and plant origin such as *Bacillus thuringiensis* toxins, pheromones and entomopathogenic viruses and fungi. When the RNAi mechanism was discovered in 1998 by Fire and Mello^8^, it quickly caught the attention of crop protectors who recognized a promising biopesticide for the control of several pests and diseases^9^,^10^. The main advantages of this technique are its expected environmental safety and the potential species-specificity. Over the last decade, a sizable body of research has been published on RNAi, confirming that RNAi really has a huge potential for the control of some insect pests^12^,^13^,^14^,^15^,^16^.

In this research, we wanted to investigate whether RNAi could be a solution for the problem that is posed by *Cylas brunneus* infestations in sweetpotato. Weevils of the genus *Cylas* are a major pest in sweetpotato, an important staple food in Africa, and conventional methods have not been very successful in its control. Since little to no sequence data were available for *C. brunneus*, the transcriptome was first sequenced. Our search of these data revealed a full set of RNAi core genes in *C. brunneus*, and we identified all RNAi-related genes we searched for in this query, indicating that RNAi is probably functional in this weevil species. The functionality of RNAi in *C. brunneus* was confirmed by an experiment where the *laccase2* gene expression was knocked down. This gene, encoding a copper-containing enzyme which oxidizes phenolic compounds to their corresponding quinones, was chosen based on its involvement in cuticle tanning and sclerotization and on the expectation that silencing this gene would result in a clear phenotypical effect, as reported earlier in *C. puncticollis*^20^, *T. castaneum*^22^, *D. virgifera*^23^, *Nephotettix cincticeps*^24^, *Monochamus alternates*^25^ and *Aedes albopictus*^26^. Treated *C. brunneus* weevils clearly showed defects in their cuticle development during and after pupation. The emerging adults were paler compared to the control group, which was injected with dsRNA specific for a jellyfish gene *gfp*, encoding the green fluorescent protein. Furthermore, silencing of *laccase2* also led to a high rate of mortality. We assume this is because the weak cuticle, including the biting/chewing mouth parts, prevented the weevils from eating. Furthermore, RT-qPCR confirmed the gene silencing at the transcript level. Confirming *laccase2* silencing at the transcript level is not easy, given the very variable natural expression profile of this gene. This is reflected in the high variation of *laccase2* gene expression in the controls, as previously observed and discussed^20^. Nevertheless, gene expression at 1, 4, 10 and 12 dpi were significantly different from the controls. These results proved that RNAi was functional and efficacious in *C. brunneus* and that the silencing effect was long-lasting, throughout different stages of development.

With the RNAi functionality confirmed, a list of 24 potential target genes was composed, using the Database of Essential Genes^21^. These genes were chosen based on the expectation that their knockdown would result in a lethal phenotype. Additionally, since silencing the *snf7* gene in the western corn rootworm *Diabrotica virgifera* was shown to be very efficient in causing mortality in this coleopteran species^12^,^16^, we annotated the homolog of the *snf7* gene in *C. brunneus* and added this gene to the target gene selection. Microinjection experiments were chosen for the first selection round, as only small amounts of dsRNA are needed for these experiments. While three genes, namely *Pfk*, *Mad1* and *rpl135,* did not perform well, failing to cause over 50% mortality, for most tested dsRNAs, injection indeed led to high rates of mortality. Fourteen dsRNAs recorded mortality higher than 90%, compared to 21% mortality in the control. One of the best performing dsRNAs was the one targeting *snf7*, causing 100% mortality in the injection experiments. In the western corn rootworm *D. virgifera*, feeding experiments with dssnf7 proved highly efficacious in causing mortality, and it is expected that transgenic corn containing a dssnf7-expressing construct, under development at Monsanto, might be one of the first commercial RNAi products on the market. Six of our selected dsRNAs performed as good as dssnf7, reaching 100% mortality 14 days after injection, namely *vha68*-*2*, *αCOP*, *l*(*2*)*NC136*, *prosα2*, *AP*-*2α*, *RpS13* and *atpd*. Similar to observations in other insect species^12^,^27^,^28^,^29^, dsRNA targeting genes coding for the subunits of the vacuolar ATPase enzyme in *C. brunneus* (vha68, vATPase A and vATPase D) proved highly efficacious in causing mortality. Similarly, dsRNAs targeting genes coding for ribosomal proteins (RpS13 and RpL19) and coatomer proteins (αCOP and βCOP) also resulted in very strong insecticidal effects, also confirming results published in other species^12^,^28^,^29^,^30^. One of the most lethal dsRNAs in our microinjection assays was the one targeting *prosα2*, encoding the proteasome alpha type-2 subunit. Ulrich *et al*.^31^ concluded in a recent publication that the proteasome could be a prime target for RNAi-based pest control in insects.

In many cases, insects have proven to be refractory to RNAi when dsRNA was administered orally, despite exhibiting sensitivity for RNAi by microinjection. With oral delivery, factors such as digestive dsRNA degradation, cellular uptake in the gut and systemic transport to the target tissues play a role as well. For example, in the desert locust *Schistocerca gregaria*, researchers have found that RNAi through microinjection works extremely efficiently, requiring only pictograms of dsRNA injected into the haemocoel for a strong systemic RNAi response, while feeding dsRNA does not elicit a silencing effect^32^. Similarly, Allen & Walker^33^ demonstrated that the hemipteran *Lygus lineolaris* is sensitive to RNAi by injection, but refractory when the dsRNA is administered orally. A metastudy collecting all available data on RNAi experiments in Lepidoptera^17^, also indicated a large discrepancy between RNAi efficiency through microinjection and oral delivery of dsRNA.

Therefore, in order to examine the potential of RNAi in a crop protection context against *C. brunneus*, the most promising genes had to be tested for efficacy through oral delivery as well. An artificial diet for *C. brunneus* had been developed before by Ekobu *et al*.^34^. However, upon validation of the bioassay using this diet, substantial mortality was observed. The diet was very moist and very susceptible to bacterial and fungal contaminations. Therefore, an alternative delivery way was first attempted wherein the dsRNA was fed to the weevils in a liquid form, by placing the larvae in 96-well plate wells containing 10 μL of a 1 μg/μL dsRNA solution. Preliminary tests with food colorant dyes had confirmed that the larvae were drinking from the solution in this setup. After 30 minutes, larvae were then placed on fresh sweetpotato root slices for evaluation. While these preliminary soaking experiments did elicit a silencing effect and subsequent mortality (data not shown), they also required large amounts of dsRNA to conduct and showed a very high variability in terms of silencing efficiency. Additionally, high mortality was sometimes seen in the control groups. Therefore, we decided to turn back to the artificial diet developed by Ekobu *et al*.^34^ and attempted to modify and optimize the recipe. Increasing the agar concentration resulted in a less moist diet which was less susceptible to bacterial and fungal contaminations. This led to lower mortality rates (20–30% maximum) in our water controls which were deemed acceptable for the RNAi experiments to be conducted. We believe that most of the remaining mortality is caused by the manipulations and transfer of the larvae from the diet to the fresh root slices around the time of pupation, which are unavoidable.

Eventually, five target genes (*vha68*-*2*, *adk2*, *prosα2*, *rpl13* and *snf7*) were initially selected for oral RNAi evaluation, based on their efficiency in the microinjection experiments and based on their involvement in distinct biological and cellular processes. The results were promising in terms of potential applicability of RNAi in the control of the sweetpotato weevil *C. brunneus*. Concentrations of 1 μg dsRNA/mL diet caused between 69% and 73% mortality after 14 days, depending on the target gene. In our experiments, the *prosα2* gene came out as the most effective in causing mortality at low concentrations in our assays, with *snf7* as a close second. No significant differences could be observed though between the three target genes which were tested. Our results also confirm the observations that Coleoptera in general often show a high RNAi sensitivity for both microinjection and oral delivery of dsRNA. Species such as *D. virgifera* and the Colorado potato beetle (CPB) *Leptinotarsa decemlineata* exhibit a very efficient silencing response upon feeding specific dsRNAs^35^,^36^. One of our hypotheses for this difference between Coleoptera and other insects is a possible lack of dsRNA degradation in the digestive system. Data generated in our laboratory seems to suggest that gut secretions of CPB are not able to degrade the dsRNA^36^, unlike many other insect species^19^,^33^,^37^. Interestingly though, the sweetpotato weevils *C. brunneus* and *C. puncticollis* do exhibit degradation of dsRNA by their digestive system. The degradation in *C. puncticollis* gut secretions happens faster than in *C. brunneus*. Comparing the oral RNAi results presented here and by Prentice *et al*.^38^, we observed that *C. brunneus* is more sensitive to oral RNAi than *C. puncticollis*. This could be related to the stronger dsRNA degradation capacity that was observed in *C. puncticollis* compared to *C. brunneus*^38^.

Since larvae are feeding inside the roots of the sweetpotato, a topical (spraying) application of the dsRNA will only expose the adults living above and potentially also in the soil while the larvae will likely not be exposed to a sufficient amount of the dsRNA. Therefore, we believe that the most logical delivery mode when applying this technology on the field will be the use of transgenic sweetpotato, expressing the insect-specific dsRNA. *In planta* RNAi, where the host plant is modified with hairpin-RNA (hpRNA) expressing construct specific for a pest target gene, has been shown to be successful in targeting several other insect species, including beetles, caterpillars, aphids and planthoppers^12^,^13^,^14^,^15^. Additionally, Kumar *et al*.^39^ have demonstrated the potential of a plant-virus based dsRNA producing system (VDPS). They infected *Nicotiana benthamiana* tobacco plants with a tobacco rattle virus (TRV) which was engineered for insect-specific dsRNA production in plants and managed to achieve knockdown of the target genes and weight reduction in the herbivorous *Manduca sexta* feeding on these plants.

One possible challenge with *in planta* dsRNA delivery is that the expressed long hpRNA is processed to siRNA by the plant’s own RNAi machinery^13^,^39^. Since short siRNA has been shown to lead to less efficient RNAi than longer dsRNA in several insects, this could lead to a diminished efficacy in pest control^10^,^16^,^40^. In fact, in two plant-mediated RNAi studies, researchers found that silencing the plant’s own *dicer* genes, and therefore preventing long hpRNA to be diced into shorter siRNA, led to increased insecticidal effects^13^,^39^, confirming that long dsRNA is more efficient than siRNA in at least some insects. Possible adaptations to the *in planta* RNAi strategy, such as expression of dsRNA in plastids, could increase the efficacy for insect control. By visual observation of the amount of diet that was eaten by the weevil larvae in these experiments, the estimated average dose of dsRNA taken up by the larvae accumulated over the 5 days on the diet was around 100–200 ng. This means that an average daily dose of 20–40 ng is enough to kill the majority of the population of weevils. Unfortunately, we did not manage to find any absolute data on attainable dsRNA levels in plant tissue, let alone in sweetpotato roots, so speculating on the possibility of producing high enough levels of dsRNA in these roots to have an insecticidal effect on *C. brunneus* is difficult. However, the results of earlier *in planta* RNAi experiments have shown effectiveness for a number of other species. Therefore, further validation of the insecticidal properties against *C. brunneus* using these transgenic sweetpotato plants will be necessary. Additionally, it would be interesting to investigate whether constructs could be made that are active against both *C. brunneus* and *C. puncticollis*, since both species are endemic in West- and Central-Africa and sweetpotato fields are often infested by populations of both weevil species. When examining the sequence identity of four of our target genes for both species (*laccase2*, *prosα2*, *rpl13* and *snf7*), we found sequence identities of 69%, 81%, 84% and 76% on nucleotide level, respectively. In theory, the sequences share sufficient identity to enable us to design dsRNAs that would affect both species. In fact, preliminary experiments at our lab have shown toxic effects in *C. brunneus* when injected with dsSnf7 which is specific to *C. puncticollis*. All injected species died from the treatment, albeit later than when *C. brunneus*-specific dsSnf7 was injected.

There are a few potential alternative delivery methods, such as viral-induced gene silencing (VIGS), symbiont-mediated dsRNA delivery, dsRNA-uptake into the roots of the plants and also the use of an attract and kill strategy, where pheromones are added to the dsRNA. While direct viral delivery, using engineered insect viruses, is to the best of our knowledge still a theoretical idea, researchers have very recently demonstrated that symbionts expressing the dsRNA can cause lethality in the host insect^41^. They managed to specifically silence genes in the kissing bug *Rhodnius prolixus* and the western flower thrips *Frankliniella occidentalis* by feeding them dsRNA-producing bacteria which were able to colonize the insects. Additionally, the proof-of-concept that dsRNA could be delivered to the host plant, either by drenching young roots in a dsRNA solution, or by injecting the dsRNA in the stem of the plants, has been provided recently in citrus trees^42^. *Diaphorina citri*-specific dsRNA could be detected in the trees for at least 56 days after delivery and small siRNA was even detected until the third month. However, these new delivery approaches are still under development and whether they could be a valid alternative to the *in planta* RNAi by transgenic plants in many cases is still under investigation. Additionally, the production price will have to be taken into consideration as well. In the case of root-drenching and stem-injection for example, large amounts of dsRNA will be necessary. This challenge could then be overcome by large-scale bacterial production systems.

Some important aspects to take into consideration in the future will be the biosafety of this technology, and the potential for resistance. While the technology itself is sequence-specific, the design of the dsRNAs used in the field will determine the actual species-specificity. Predicting the specificity of the dsRNAs *in silico* will be difficult, especially in the near future, since that would require genome sequence data of all organisms living in the ecosystem of the intended crop or plant. However, some measures to minimize the chance of harmful effects on non-target organisms, such as avoiding very conserved regions of the target gene, could already be taken for these insects of which the genome has not been sequenced. Furthermore, efforts in filling this sequence data gap are ongoing and increasing at a rapid rate, for instance with the i5k project^43^.

In conclusion, this research has shown that RNAi could be a powerful tool in the control of the sweetpotato weevil *C. brunneus*. We have identified several target genes in the transcriptome for which the delivery of low doses of dsRNA leads to effective silencing and a rapid and high mortality. Further testing, using transgenic plants which express the weevil-specific dsRNA will be necessary to demonstrate applicability in the field.

## Methods

### Sweetpotato weevil colony

*C. brunneus* sweetpotato weevils were collected from a sweetpotato field near Namulonge, Uganda. Weevils were reared on sweetpotato (*I. batatas*) in plastic cages (100 × 100 × 160 mm) at the Lab of Agrozoology, Ghent University under a 16:8 light:dark regime at 27 °C and 65% RH. Fresh sweetpotato roots were supplied every three days.

### *C. brunneus* transcriptome sequencing

The RNeasy Mini Kit (Qiagen) was used to extract total RNA from *C. brunneus* second instar larvae, following the manufacturer’s instructions. RNA quality and concentration were examined on an Agilent 2100 Bioanalyzer using a RNA Pico Chip. Following the instructions of the TruSeq Stranded mRNA Sample Preparation Guide, 1 μg of the RNA was purified to isolate poly-A containing mRNA and subsequently fragmented into small pieces using divalent cations. The purified mRNA fraction was then used for synthesis of first and second strand cDNA. After the end repair on the double-stranded cDNA, 3′ ends were adenylated and adapters with indexes were ligated for multiplexing. The cDNA library was amplified by PCR and then AmpureXP beads were used for purification. The quality of the final library was then evaluated using Agilent’s Bioanalyzer High Sensitivity DNA Chip prior to clustering on the Illumina cBot. The cDNA libraries were sequenced on the Illumina sequencing platform (HiSeq2000) where each sample was collocated in one lane of a 100 bp single-end run. After sequencing, the Trinity software (http://trinityrnaseq.sourceforge.net/) was used for *de novo* assembly of the transcriptome. Finally, protein sets of *Tribolium castaneum* (14,366 proteins), *Drosophila melanogaster* (27,813 proteins) and *Dendroctonus ponderosae* (2,502 proteins) were used in a transcriptome-wide homology search, using a cut-off E-value of 1.0^−3^.

### Annotation of RNAi-related genes

The presence of the RNAi-related genes in *C. brunneus* was confirmed by performing queries of homologous *Tribolium castaneum* sequences against the transcriptome, using the tBlastn tool (http://brcclusterrac.statgen.ncsu.edu/Niblet/). The same list of RNAi-related genes was used as in Prentice *et al*. and Swevers *et al*.^20^,^44^ and is comprised of 51 genes, covering the RNAi core machinery genes, auxiliary factors, nucleases, genes related to the antiviral RNAi response and to dsRNA uptake (Supplementary Data 1). The ORF Finder algorithm from the National Center for Biotechnology Information (NCBI) was used to detect the open reading frames in the transcriptome data. Hits in the *C. brunneus* transcriptome were run against the NCBI non-redundant protein database using Blastp. In case frame shifts were discovered, sequences were further analyzed using Blastx against the non-redundant protein database at NCBI.

### Target gene selection and annotation

The Database of Essential Genes (http://tubic.tju.edu.cn/deg/)^21^ was used to select 20 potential target genes which were suspected to lead to a lethal phenotype when silenced. Additionally, four extra target genes were selected based on literature. Table 1 presents an overview of these selected target genes. These genes were then identified in the *C. brunneus* transcriptome and the sequences retrieved as described for the RNAi-related genes.

### dsRNA synthesis

Total RNA from second instar *C. brunneus* larvae was extracted using the RNeasy Mini Kit (Qiagen) and first strand cDNA was subsequently synthesized with the Superscript II First strand cDNA synthesis kit (Invitrogen), using oligodT primers. Gene-specific primers containing a T7-promotor sequence (GCGTAATACGACTCACTATAGGGAGA) at the 5′ end were used in a PCR to amplify the template for the dsRNA synthesis reaction (Table 1). The PCR products were subsequently purified using the CyclePure E.Z.N.A kit. (Omega Bio-Tek). Finally, the dsRNA was synthesized using the MEGAscript kit (Ambion), following the manufacturer’s instructions. Nuclease-free water was used for the final elution of the dsRNA. The full sequences of all dsRNA fragments used in this research are listed in Supplementary Data 3. The dsRNA quality was evaluated by gel electrophoresis and quantified with a NanoDrop ND-1000 (Thermo Scientific).

### Larval injection of dsLaccase2

Second instar *C. brunneus* larvae were injected with a dose of dsRNA targeting the *laccase2* gene. The injections were performed with the FemtoJet (Eppendorf), using self-pulled glass capillary needles. The injected dose was 0.2 μg dsRNA/mg body weight (BW) (~0.5 μg). This dose was based on our experiences from earlier experiments in a related species, *C. puncticollis*. The control group was injected with dsGFP. After injection, the larvae were placed on fresh root slices for phenotypical evaluation.

### Target gene screening by microinjection

A dose of dsRNAs for the 24 target genes was injected into second instar larvae. The injections were performed as described above for the dslaccase2 experiment and each larvae was injected with a dose of 0.2 μg/mg BW. The control group was injected with dsGFP. Larvae were placed on fresh sweetpotato root slices for further evaluation. Every 2 days, the larvae, pupae or adults were moved onto fresh root slices. Mortality was evaluated for 15 days past injection. Statistical analysis was performed using SPSS.

### Oral dsRNA feeding bioassays

Three target genes were selected, based on the injection experiments, for oral evaluation, namely *prosα2, rps13* and *snf7*. Second instar larvae of *C. brunneus* were placed on an artificial diet, adapted from Ekobu *et al*.^34^ mixed with the dsRNA, at different concentrations (30 μg dsRNA/mL diet, 10 μg dsRNA/mL diet and 1 μg dsRNA/mL diet). Seven days after the insects were placed on the dsRNA-containing diet, around the moment of pupation, the insects were transferred to fresh root slices for further evaluation. Root slices were replaced by fresh ones every 2 days. The insects were observed for mortality for another 15 days. Samples were collected at day 2 and 4 post injection, immediately homogenized in RLT buffer (Qiagen Rneasy Kit) and then stored at −80 °C for possible future RNA extraction. The control groups were fed with dsGFP. GraphPad Prism software was used to generate the figures and perform the statistical analyses.

### Real-time quantitative PCR

For RT-qPCR analyses, total RNA was first extracted from whole insect bodies using the RNeasy Mini Kit (Qiagen) and following the manufacturer’s instructions. Three biological samples of 5 pooled insects were prepared for each time point. Possible DNA contamination was avoided by subjecting the samples to a DNaseI treatment (Ambion). Subsequently, the RNA was quantified using a NanoDrop ND-1000 (Thermo Scientific) and the quality was assessed by 1.5% agarose gel electrophoresis. cDNA was synthesized from 0.9 μg of this total RNA using SuperScript II First Strand Reverse Transcriptase (Invitrogen), following the manufacturer’s instructions. RT-qPCR was performed with a CFX 96^TM^ real-time PCR Detection System and the data was analyzed using the CFX manager software (Biorad). Primers used for RT-qPCR are listed in Supplementary Data 3. These were first validated with a standard curve based on a serial dilution of the cDNA to determine the primer annealing efficiency and a melting curve analysis was performed over a temperature range from 60 to 95 °C. The RT-qPCR reaction mixture consisted of 10 μl SsoFast^TM^ EvaGreen Supermix (Biorad), 0.4 μl of both primers (10 μM), 8.2 μl nuclease-free water and 1 μl cDNA, in a total volume of 20 μl. The RT-qPCR was run in 96-well Microseal PCR plates (Biorad). The amplification conditions were 3 min at 95 °C followed by 39 cycles of 10 s at 95 °C and 30 s at 58 °C. Two reference genes, ribosomal protein L32e (rpl32) and β-actin, were used for normalization. Appropriate controls, no-template control and no reverse transcriptase control, were also included in the assay. The relative transcript levels of the target were normalized to the expression level of the reference genes. Statistical analysis was performed using the Student t-test on log-transformed data which is integrated in the qBase+ software (Biogazelle, Zwijnaarde, Belgium).

## Additional Information

**How to cite this article**: Christiaens, O. *et al*. RNA interference: a promising biopesticide strategy against the African Sweetpotato Weevil *Cylas brunneus*. *Sci. Rep.* **6**, 38836; doi: 10.1038/srep38836 (2016).

**Publisher's note:** Springer Nature remains neutral with regard to jurisdictional claims in published maps and institutional affiliations.

## Supplementary Material

Supplementary Data S1–S4

## Figures and Tables

**Table 1:** Overview of the target genes selected for RNAi evaluation.

Gene name	Abbreviation	dsRNA length	dsRNA synthesis primers*
ATP synthase, β subunit	ATPsynβ	370	TGTCGCAGCCTTTCCAAGT
			TAGGTAATTTGTTTCCTTTATTTTTAC
Vacuolar H^+^ ATPase 68 kDa subunit 2	Vha68-2	436	GATATGGCAACGATTCAGGTA
			ACATTGTATATTTTGTACGCTCTCCG
Synaptobrevin, isoform A	Syb	438	GCACTATTGCGCCACCCTG
			GGTGGTTAACTGTGATTTGAGGAG
phosphofructokinase	Pfk	415	GTCACCGCCGCTAGTGAACAC
			ATATATATTTTTTATGTTGTTAAAC
adenylate kinase-2	adk2	370	CGTAACTGGCGAGCCCTT
			ATTATTGCCGATATTTGTGCGAG
Focal adhesion kinase isoform D	fak	400	CAAAGTCGGCCAGTTTGACGC
			TGCAAGGGCGACGCCGACCT
gamma-coatomer protein, isoform C	γCOP	400	AGGCGTGGCCGAGCTTATAAC
			AACTTATGCACCATTTATTGACAA
delta-coatomer protein, isoform A	δCOP	417	GCCCTTGGGGCATTGAATTCC
			GCGAGGCGTTCAATTGCAAACC
alpha-coatomer protein, isoform D	αCOP	400	AATCAAATTGGCCTTTACTGACCG
			TGAAGAAGCCTGCAAGCTGATTC
TBP-associated factor 1, isoform D	taf1	380	GAAAAGTCCTCGGCTGAAGG
			ATTTTCTATGTGTCAACAATTATAAC
lethal (2) NC136, isoform B	l(2)NC136	400	TCAATTAAGGTCTCTGTCCTCC
			GTTCTACCACCTGCCGCA
Proteasome α2 subunit	prosα2	400	TGGTCCAAATTGAGTACGCG
			GAAGGGTCGCATTGGAACAG
DNA polymerase interacting tpr	Dpit47	400	ATTTTGTCGCAGAATTCGACGG
containing protein of 47 kD			TTTTCATGAAGGAACCCCCC
α-Adaptin, isoform A	AP-2α	440	ATTATTGGTGTGGTTGGCGACA
			AAGTGGGAGGTTATATTTTAGGTG
Mad1	Mad1	420	CTCGTCGAAGCCCGAAACAT
			GGACGCCAGTTTCTTCTGAG
lesswright	lwr	410	GCTTAGCGGAAGAAAGGAAAG
			ATACTCTAAACGATTTTGGCAAT
RNA polymerase I 135 kD subunit	rpI135	400	ACCGGTCCCACCGATATTACA
			TTTGCAGACACTTTATTCTTACAT
Eukaryotic initiation factor 4a	e-IF4a	400	AAAACCATCTGCAATTCAACAGAG
			CATTTCATCTGCTTCATCTAAC
ribosomal protein S13e	RpS13	393	GCTTGCGAATAGCAACAGCTTTC
			CAGGAAAGACCAATTTCCTTT
DNA polymerase α 50 kD	DNA pol-α50	430	TCAGCAATTGCGGATCTAAC
			GAAGAGAAATCTCATTTACTCTC
vATPase A	atpα	284	TCAGCGTCCATTGAAAGATA
			CGTCAAATTCTGTTTCCAAAAC
vATPase D	atpd	206	TGGAGGCCATTCATGTTGCT
			TTCAGCGGTAGTACCACCAA
Ribosomal protein L19	RpL 19	203	GGCATCTGTACCACTCACTGTA
			CACCTCTTGTTTCTTGGTAGCA
snf7 (shrub ortholog)	snf7	251	AGGGAAACGGAAGAAATGCT
			GCGGCATTTTTCATGGTAGT
Laccase2	Lac2	364	CGCTTTAGATTTGGGTAGCA
			GACACCGTCAGCCAAGATAC

^*^A T7 promotor sequence (GCGTAATACGACTCACTATAGGGAGA) was added to the 5′ end of each dsRNA synthesis primer.
