# Supplementary material for: RNA interference: a promising biopesticide strategy against the African Sweetpotato Weevil Cylas brunneus
Source: Sci Rep. 2016 Dec 12;6:38836. doi: 10.1038/srep38836 (PMC5150260; doi:10.1038/srep38836)
Supplement: Supplementary Data S1–S4 [file srep38836-s1.pdf]

Supplementary information for:

RNA interference: a promising biopesticide strategy against the African Sweetpotato Weevil *Cylas brunneus*.

Authors

Olivier Christiaens[1$,] Katterinne Prentice[123$], Ine Pertry[24], Marc Ghislain[3], Ana Bailey[5], Chuck Niblett[5], Godelieve Gheysen[2], Guy Smagghe[1]*

Supplementary Table S1: Overview of RNAi-related genes in *Cylas brunneus*

		Contig	*start*	*stop*	*AA*	*Comparison to Tribolium (blastp)*	*First hit BLASTp*
**miRNAi**							
**Dcr-1**	**full length**	**Cb.comp39694_c0_seq3**	**254**	**5704**	**1816**	**E=0.0; bits=2388**	**hypothetical protein YQE_09128, partial [Dendroctonus ponderosae]**
**Ago-1**	**partial (N-39)**	**Cb.comp42266_c0_seq6**	**383**	**3052**	**889**	**E=0.0; bits=1764**	**argonaute 1 [Tribolium castaneum]**
**Loquacious**	**partial? 52AA gap**	**Cb.comp43240_c1_seq4**	**144**	**1118**	**324**	**E=6e-164; bits=474**	**PREDICTED: similar to tar RNA binding protein; hypothetical protein TcasGA2_TC011666 [Tribolium castaneum]**
**Drosha**	**partial (N-135, C-72)**	**Cb.comp34198_c0_seq1**	**406**	**3951**	**1181**	**E=0.0; bits=1675**	**PREDICTED: similar to ribonuclease iii [Tribolium castaneum]**
**Pasha**	**partial (N-79)**	**Cb.comp41893_c0_seq1**	**3419 (3251 aag)**	**4732**	**437 (493)**	**E=0.0; bits=673**	**hypothetical protein YQE_10523, partial; hypothetical protein D910_05983, partial [Dendroctonus ponderosae]**
**Exportin-5**	**partial (C-15)**	**Cb.comp42659_c0_seq1**	**-3924**	**-289**	**1211**	**E=0.0; bits=1882**	**hypothetical protein D910_01904 [Dendroctonus ponderosae]**
**siRNAi**							
**Dcr-2**	**full length**	**Cb.comp38178_c0_seq1**	**1837**	**6702**	**1621**	**E=0.0; bits=1590**	**hypothetical protein D910_09530, partial [Dendroctonus ponderosae]**
**Ago-2**	**partial (N-39)**	**Cb.comp42256_c0_seq1**	**149**	**3376**	**1075**	**E=0.0; bits=997**	**hypothetical protein D910_08685 [Dendroctonus ponderosae]**
**R2D2**	**full length**	**Cb.comp39376_c0_seq2**	**243**	**1181**	**315**	**E=1e-74; bits=243**	**hypothetical protein YQE_06343, partial [Dendroctonus ponderosae]**
**piRNAi**							
**PIWI**	**partial(N-45)**	**Cb.comp37817_c0_seq1**	**148**	**2868**	**906**	**E=0.0; bits=996**	**piwi [Tribolium castaneum]**
**AGO-3**	**full length**	**Cb.comp38974_c0_seq1**	**213**	**2807**	**864**	**E=0.0; bits=995**	**hypothetical protein YQE_10018, partial [Dendroctonus ponderosae]**
**Aubergine**	**partial (N-106)**	**Cb.comp37817_c0_seq1**	**148**	**2868**	**906**	**E=0.0; bits=977**	**piwi [Tribolium castaneum]**
**Zucchini**	**full length**<br><br>**full length**	**Cb.comp31873_c0_seq2**<br><br>**Cb.comp42309_c1_seq15**	**-653**<br><br>**-844**	**-53**<br><br>**-119**	**212**<br><br>**241**	**E=4e-54; bits=184**<br><br>**E=9e-44; bits=158**	**hypothetical protein YQE_07414, partial [Dendroctonus ponderosae]**<br>**hypothetical protein TcasGA2_TC010319 [Tribolium castaneum]**
**Protein methyltransferase 5 gene**	**full length**	**Cb.comp15581_c0_seq1**	**455**	**2311**	**618**	**E=0.0; bits=815**	**hypothetical protein YQE_02756, partial; hypothetical protein D910_07881 [Dendroctonus ponderosae]**

**Tudor-domain containing proteins**	**full length**	**Cb.comp38296_c0_seq1**	**289**	**3507**	**1072**	**E=0.0; bits=808**	**hypothetical protein D910_07958 [Dendroctonus ponderosae]**

		Contig	*start*	*stop*	*AA*	*Comparison to Tribolium (blastp)*	*First hit BLASTp*
**Nucleases**							
Snipper = Eri1	fulle length	Cb.comp34051_c0_seq1	100	831	243	E=8e-90; bits=276	Snipper [Tribolium castaneum]
Nibbler	partial (N-404)	Cb.comp42400_c0_seq1	249	2876	875	E=0.0; bits=927	hypothetical protein TcasGA2_TC002596 [Tribolium castaneum]
Sdn1-like	full length	Cb.comp38443_c0_seq2	63	2063	666	E=0.0; bits=714	hypothetical protein D910_11144 [Dendroctonus ponderosae]
	partial (147AA mismatch)	Cb.comp40516_c0_seq1	101	3442	1113	E=0.0; bits= 790	hypothetical protein D910_06808 [Dendroctonus ponderosae]
dsRNAse	partial (N-12)	Cb.comp34069_c0_seq2	29	1243	404	E=8e-118; bits=360	hypothetical protein YQE_04599, partial [Dendroctonus ponderosae]
	partial (N-80)	Cb.comp37521_c0_seq1	-1321	-392	310	E=8e-83; bits=278	hypothetical protein TcasGA2_TC001268 [Tribolium castaneum]
Exosome	partial (N-23, C-210)	Cb.comp37515_c0_seq1	-2830	-104	908	E=0.0; bits=753	PREDICTED: similar to Rrp6 CG7292-PB [Tribolium castaneum]
Poly(A) polymerase	full length	Cb.comp42000_c0_seq4	-2481	-688	597	E=0.0; bits=864	hypothetical protein TcasGA2_TC003818 [Tribolium castaneum]
**Antiviral**							
Ars2	partial (N-387)	Cb.comp43566_c0_seq35	-6983	-5049	644	E=0.0; bits=725	hypothetical protein YQE_07634, partial [Dendroctonus ponderosae]
CG4572	partial (n-17)	Cb.comp37407_c0_seq1	-1503	-91	470	E=0.0; bits=687	PREDICTED: similar to salivary/fat body serine carboxypeptidase [Tribolium castaneum]
	partial (N-7)	Cb.comp33398_c0_seq1	281	1669	462	E=0.0; bits=529	PREDICTED: similar to salivary/fat body serine carboxypeptidase [Tribolium castaneum]
	partial (N-7)	Cb.comp44717_c0_seq1	126	1487	453	E=7e-168; bits=493	hypothetical protein YQE_12754, partial [Dendroctonus ponderosae]
Egghead	full length	Cb.comp36926_c0_seq1	505	1883	462	E=0.0; bits=797	PREDICTED: similar to conserved hypothetical protein [Tribolium castaneum]
ninaC	partial (103AA gap)	Cb.comp34434_c0_seq1	163	3668	1168	Trib not in first 100 E=0.0; bits=704	Neither inactivation nor afterpotential protein C [Acromyrmex echinatior]
**dsRNA uptake**							
CG4966= orthologous to the Hermansky-Pudlak Syndrome4	partial (127AA gap)	Cb.comp43091_c0_seq27	911	3106	731	E=0.0; bits=591	hypothetical protein TcasGA2_TC002372 [Tribolium castaneum]

FBX011	partial (N-55)	Cb.comp41779_c0_seq1	292	3036	914	E=0.0; bits=1639	hypothetical protein D910_09724 [Dendroctonus ponderosae]
Scavenger receptor SR-C-like protein	partial (N-58, C-138)	Cb.comp41729_c0_seq2	-1209	-1	402	E=6e-151; bits=450	PREDICTED: similar to scavenger receptor SR-C-like protein [Tribolium castaneum]
Eater	partial (N-38)	Cb.comp30666_c0_seq1	-2957	-1893	354	E=1e-135; bits=402	hypothetical protein D910_03817 [Dendroctonus ponderosae]
Sid-1 related C precursor	partial (N-336)<br>partial (C-445)	Cb.comp42797_c0_seq1<br>Cb.comp37306_c0_seq1	2471<br>422	3853<br>1385	460<br>321	E=0.0; bits=584<br>E=4e-94; bits=306	hypothetical protein YQE_05958, partial [Dendroctonus ponderosae]<br>hypothetical protein TcasGA2_TC015033 [Tribolium castaneum]
SID1	NOT<br>partial (N-35, C-434)	Cb.comp37306_c0_seq1	422	1385	321	E=2e-47; bits=179	hypothetical protein TcasGA2_TC015033 [Tribolium castaneum]
SID1-B precursor	NOT<br>NOT						

		Contig	*start*	*stop*	*AA*	*Comparison to Tribolium (blastp)*	*First hit BLASTp*
**RISC**							
Translin	full length	Cb.comp39483_c0_seq2	298	1012	238	E=2e-112; bits= 333	hypothetical protein YQE_05829, partial [Dendroctonus ponderosae]
Similar to translin associated factor X	partial (C-283)	Cb.comp39981_c0_seq1	292	1110	272	E=4e-105; bits=327	hypothetical protein D910_08298 [Dendroctonus ponderosae]
HEN1	partial (N-27, C-43)	Cb.comp43241_c0_seq15	2984	5749	921	E=5e-138; bits =445	hypothetical protein D910_04572, partial [Dendroctonus ponderosae]
Similar to Gawky	full length	Cb.comp42534_c0_seq11	282	4454	1390	E=0.0; bits=1214	hypothetical protein TcasGA2_TC006679 [Tribolium castaneum]
Similar to fragile X mental retardation syndrome related protein 1	partial (C-205)	Cb.comp32338_c0_seq1	-1154	-1	518	E=0.0; bits=722	hypothetical protein D910_07822, partial [Dendroctonus ponderosae]
Maelstrom	partial (C-103)	Cb.comp40771_c0_seq3	236	1678	480	E=9e-70; bits=239	hypothetical protein YQE_09694, partial [Dendroctonus ponderosae]
Tudor-SN	full length	Cb.comp39931_c0_seq1	-2897	-168	909	E=0.0; bits=1389	hypothetical protein YQE_11841, partial [Dendroctonus ponderosae]
Elp-1	partial (N-50, frame shift)	Cb.comp42860_c1_seq1	-4842	-1793	1015	E=6e-133; bits=434	hypothetical protein D910_02697 [Dendroctonus ponderosae]
Vasa intronic gene (VIG)	partial	Cb.comp35716_c0_seq2	168	1400	410	E=1e-100; bits=315	hypothetical protein D910_11911

	(N-22)						[Dendroctonus ponderosae]
Homeless (spindle-E)	partial (N-19)	Cb.comp40708_c0_seq1	145	4458	1437	E=0.0; bits=1291	hypothetical protein YQE_03529, partial [Dendroctonus ponderosae]
Staufen	partial (C-17)	Cb.comp23511_c0_seq2	-3319	-1085	744	E=0.0; bits=939	hypothetical protein YQE_06727, partial [Dendroctonus ponderosae]
Clp1 homolog (kinase)	full length	Cb.comp39887_c0_seq2	256	1530	424	E=0.0; bits=707	PREDICTED: similar to AGAP007701-PA [Tribolium castaneum]
RNA helicase Belle	partial (C-312)<br>partial (N-380)	Cb.comp15415_c0_seq1<br>Cb.comp38184_c0_seq1	-1170<br>3	-1<br>947	389<br>314	E=0.0; bits=586<br>E=5e-157; bits=467	ATP-dependent RNA helicase belle [Tribolium castaneum]<br>hypothetical protein YQE_06337, partial [Dendroctonus ponderosae]
p68 RNA helicase	full length	Cb.comp35296_c0_seq1	150	1760	536	E=0.0; bits=819	hypothetical protein YQE_12421, partial [Dendroctonus ponderosae]
Gemin3 homolog	partial	Cb.comp41450_c0_seq3	-2395	-449	648	E=2e-90; bits=306	hypothetical protein TcasGA2_TC003675, PREDICTED: probable ATP-dependent RNA helicase DDX20 [Tribolium castaneum]
Armitage	MOV10 helicase	Cb.comp41200_c0_seq1	55	3441	1128	MOV10 helicase	hypothetical protein D910_08795 [Dendroctonus ponderosae]
GLD-1 homolog	full length	Cb.comp41351_c0_seq6	198	1247	349	E=0.0; bits=645	held out wings [Tribolium castaneum]
ACO-1 homolog	full length	Cb.comp24263_c0_seq1	223	2904	893	E=0.0; bits=1520	PREDICTED: cytoplasmic aconitate hydratase-like [Tribolium castaneum]
PRP16, mut6 homolog	full length	Cb.comp43081_c0_seq5	-5049	-1501	1182	E=0.0; bits=2066	hypothetical protein D910_03265 [Dendroctonus ponderosae]

Orf finder and blastp for Brunneus (e- and bits scores 11, 17,25/04/14)

Supplementary Data S2: Sequences RNAi-related genes in *Cylas brunneus*

siRNAi pathway *Cylas brunneus*

Dcr-2

>Cb.comp38178_c0_seq1 len=6773

cDNA

```
GGGGGGTTTACGCATTTATTAGCCTAAATTAATAATGTTCTCTCAAATTCCAAACGCAGTCTCAATTTTTTATTACAATTACGTTA
AAACGATGGACGGGGACTTTTTTCAAGGCAACCACTCTGCGTCGGGGTTGATCCTGTCTTTCGCCTCTTGAATTTTGTTCCTTACG
GTCTGAGATTCTTCGTCTGCCTTATCTGATATTTTGTGGTACACGTCCTTGGTTTTTCCCGCGATGTACTCAACACCGTCACCTAT
TTTTTCGCCCGCCTTCCTGATTACACCGGCCGCCGCTTTGGCCGATATCGTTTCGTCATTAATGTCGATGTTGTACCCCGCGGTCA
AGTTCCTGGGGCAGTCCTTTTGACTGATTATAGACAGTTCGAACGGATCGATTCCCGCCGAGGCGAGCTTATTACGGATTTTGTCC
TGGTACATTTTGTCCAACGTCCTGGATCGAGAGAGAACGGTGGCGGATTGCCGATGGGCGAACGTCAGCTTCTGACACGTGAATAT
ACCGAGGTAGTTGCTGTAATCGGTAGCAAATACTGTGAACGAGGAGGAACCCGCGACGCTCAGCGGAAATTTGACGGTCATTTTGG
CGGGTATCGCGCTGTCGGGGACTTTAAGGTTACCGGTGTAGTGGTAACCGTGTTTCAAAGGCGTCAAGGCGAGCAAAAAGTGCTGG
CTGATCTCTTCGACCTTGTATTCGCCGGGTTCGTCCGTTGCGGTTATGTTGTACACGATGCAAGAGCTAGCGGTGCTAGTTTTCTC
GATTACGAACCAAATGCCAAGAATCCCTCTCATATTAAAATCCGACTGTGGTTCGATATTCGGACAAGCCCCGAGATGATACGAGT
GCATGTCCCCGCTTTTGGCCGCCAAAAGCAGCGCGAACGCGGCAAATAAACCCTTCATTTTGTAAAAACGCGAGCCGACGAAAGGT
CAATATTTCACTCACTGTGATCGCAGCGGCTACGTCGAAGTCGGCTAACTACGTTAATTTAATAATGATAATTCCTTTTTATGCGG
GCAAGGCGTCAATGAAAACGAGCTGACCCATGCCGTTTATTGTAATCGGCCTGTTGGTTCTTGGAAACGTCTATTATAAGGTTTAT
TGTTGTCCGCTGTTGATAGCGGCAGCCAGTGACTGGGCCGAAGAAAACCAGCGGCGGGTCGTGATTTTGTTAAGCCACACGTCTCC
CGCGTTGGTATTTATAATAATTTGAAGTGCAACTCCGCCCCTGGTTACTCTAAATAAAACGCACTTCGAAACCGTTATAAAATGAC
TCAACGGCTAAGAAGTGTGATTTTCGAATTAATTTCGCGTCACTCTGACATAATTCAATAAAAATTCCCAGTTATTTTTGTTTGGG
TATGGTTCAAAGTGCACGAACGGAATATGATAAGCAAGCCGTGAGTCACAATGGGCGCCGATGACCGAATAGAAGGAAAACTTTTG
ACTTTACCGTTTTTGAGGTTAGGTTACGTGTTCCCGATATGCCCAGGTTCGGATTCTGAACGGATTGCACAAAGCGGAAGTTTGCT
TCTGCCTGTTTCCAATTTTTAATTAGACCAGTCAATATAATAGTATTTTGAAGAATTTAATTAAAGGAAATTCCAGACCCTTTCTT
GAAGAAGGCACGAATGACGTGAGCTCATTGAAAGAAGAAGAAGAAAAGAAGAAGAATTTATGAAAATTTTATATTGGTAGTAG
AGAGAGGGTTGATTCTGTTGGTAGCTCTGTTTATTGTTGGCGTGGTATTCGCAGAATCAACCAAATCAACATCGCGTTAAAAATTT
TGTGTACGCGACGTTGCGCAATAAATCGTCATGGAGGTGGATATGGAAACCGGGGACCATTTTACCCCGAGAAATTATCAACTGGA
ACTGATGGAGATCGCGATGAAGAAAAACACAATTATATATTTGCCCACTGGCTCTGGGAAGACTTTTATTGCGGTGTTGGTGCTCA
AGCAAATGGGTCATTGCCTCTCCAAATCGTACAGTCAAGGAGGCAAGCTGTCCTTTATTCTAGTGAACACAGTGGCCCTGATTGAC
CAGCACGCTGACGTTATTAAAAACAGGACCTGCTTTAAAGTTGGTCGCTACTCGGGCGAAATGAACTTGGACTCTTGGTCGAAATC
GAAATGGTTGGAAGAATTTGATAAGCACCAGGTTATTGTTATGACCGTTCAGATACTTGCCAATTTGGCAAATTCTGGGTTTATAG
ACTTGAATAAAGTCAATTTATTGGTATTTGACGAATGCCATCGTGGGGTTAACGATCAGCCCATGAGGCAGTTATGTAAATTATTT
GAACACTTACATGACAAACCCAGGGTCCTCGGACTGACGGCGACGTTATTAAACGGCAACTGCAAACCCAACAAGGTGATTGAGAA
TGTGAGAGAATTGGAAGTTACATATCACGGGCAAGTCGCCACCGTTGAAGGTTTGAATCAAGTTGTTGGATATTCCACCAATCCGG
AAGAAATCATATTGCAAATTACTCCGCACATATTAACCACCTCTGAAAACAGGGTCAAGTCCCTCTTGAAACTCGCCACAGAACAA
ATTAGAACATTCAAGATATTGGATCCTGTTATCGAGCCGCCAAGCAGTGACCTGAGACCCCTAAACAAGAATAAGGGACTCAAGCA
GTTGGAGAATCTGATTTTTGATGTCATTTTGCAAATAGAATGGATGGGCGCGTATGGGGGCGACAAGTCATTGCTGGCCCACAGCA
TACAAATCGAGAGGATGTTGAAACACTGCAGCGATCTCAGTTTGCATAAAATCCTTAGCTATGTGCAGCTGATTTTGAGTTATGGC
CGGCAAATTTTCCACCAAACCATGAATGGACACACTGAATATGAGAAAATTATTTTCTTCTCATCAGACAAGATGCGAAAATTGAT
TAAAATTTTCGAAGATTACCCGAAAACGTCCCAGGAACCGAGCGCGCTCGTCTTTACGAAACGACGGTTTACCGCGAAGGTCATCT
ACTACGTTCTGGACAGCCTGAGCAGGGCGTCGACCAAATTCAAATACATCAAGGCAAATTTCATGGTCGGGAACAACGGGAACCCT
TTCATGGACACCAGGGAGGCGATGTACCTATCCAAAAAGAACAGGGATATCCTGCAGAGGTTCAACAAAAAGGAGATCAATGTGTT
GGTCGCATCGAACGTGTTGGAGGAGGGCGTCGACATAGCCACATGCTCGCTCGTCATTAAATTTGAGGCTCCCGAGGAGTATCGCT
CGTACATACAGTCCAAGGGACGGGCGAGGAACAAGTCCAGCCGGTACATCATGCTGGTTAACGGCGACGAGATGGGCAACTTTCAG
GGGCGCTACCGGGAGTACCAAGAGATCGAATCCATCTTGAACGAGTTTCTGATCGGCAAAAATCTGGGCCGCTGCGAACCCTCAGA
CGCCGACGTGAACGACTTTTACAACGAAGACTGCCTCCGTCCCTACTTTGTGGACAGTCCAAATTCCGCCCGGGTAACGTCGACGT
CCGCCATATCTCTGTTGTGCTCCTATTGTCTTTCTCTCCCGTCCGATAAATACACGGTGCACGCTCCCGAGTTGTTTTACAAGACG
AAAGAGGGGGCGAACTTGAAAAAACTACACAGCGTCGTCATTCGGATGCCCGTTATTTGTCCCATCGACATTGTCACGGGCCCTTT
CATGCCCAGCTTGAAACTGGCAAAACGCGCGGCCGCGCTCAAAGCGTGCGAGATGCTCCACAAGTGCCGCGAGTTGGACGACACGC
TAACGCCGCGAAAGAGGACAGTGCTCGAAGAGGACGTCGGGTTCCTCTTCGAGCACTACCCGGCCGTAAAGGAGCCCGACGCCGGC
ACAAACAAGCGCAAGCGGCTCCATAGGCACGCGATTCCTCCGTGCGTTAAGGGGGCGTTACCGCAGGCCGACCCGGTTTACCTTCA
CGTTATACATCTGACACCTGCGTTCGCGAGGGGCGAGAACGTCAACACGGCCACCATGTACGACATGTACGATTCGGCGCTGTGTT
ACGGTGTAATCACGCCGAATCCTGCGCCCGTGATCTGCGATTTTCCGGTTTACGTGTCGGCGGGCACCATAAACGTGTCGCTCGAC
GTGAACGTCGCGCTGATCAGCCTCGACGAGCGCGATCTTGACGACATCAGGGCGTTCAACGTGCTCGTATATTGCGACGTGTTGCG
CTGTTTAAAGGAGTTTTTGATCGTCGACAATTCGGAAGGGGGCGTGTCGATGTGGATCGTGCCCGTCGATCGGGATCGCGGCCGCA
TCGACCTGGACACACTAAGGGAGTACAAAGCGGTCGGCGAGATCGCGGAACCCACCCGCGAGGAAAAGGCGAATTTGGAGGTCAGC
CTCGACAATTACCTCAGAAAGATCGTCGCGCCATGGTACAGGGACTCCGGGTTTTACATCGTGACGGAGGTGACGTTCACGAAGAC
CGCCAGGAGCGAGTTCCCAAACGAGTCGTTCGGCACGTATGAGGAGTACTTCAGGGACAAACACAATCTGCACCTGGTCGACCCGG
```

ACAAGCCGCTATTGTACGTCAAGTCGCTGTCGAAACGTCTAAATTGCCAGAAGCCGCGCGGCGACTCGAAAAAGAAACGCGACGAG
AAGTTCGACGACCTGGAAATACATCTGGTGCCCGAGCTCGTCGTCAAGCAAGAGTTCCCGGCGCCGTTGTGGGTCCAGGCCGGCCT
GCTGCCGACCGTGCTGAACAGGCTGTCGTTTTTATTTCGTTTGGAGCACCTGCGGTCGACGATCGCGAGGGAGGCGGGACTAGGCC
GCGAGATCGTTCCTATCAAGGCGCCCCTCGAACTCGACCGCGACCTCTTAAATTATGAACCCCGTTCGAAAAATGAGACCGAGGCG
AATGTGGTGGGGGTGTTGCCCGGCGACGGTTTTCAGCTTCACGCGCTGCCCGCGCTCAACGTCAACAAAGATTACGCGACCAAAGT
GCTCGAACGGGACTACTCGTGGAAGGATATCGAGGAACCGAAAGACGTGGAGAGGGACATCGAAGACGTCACCATAATGGATATCG
AGTACTACGAGAAGTTTATCGGCTTGCCGCTCCAGGAGGGCGACGTGCATCTGAGGAACCACAGGCCCGTTATAGGCAACCAACTG
GCACTGACTTATCACAAGCATTTCGTGCCGAAGCCGATCCAGCTGTTGGAACGGAAGAGCGTAGCCGGACCGGAACTGGCCTCGAT
ATATCAAGCGGCGACCACGGCCAAAGCGAACGACATCGTCAACATGGAACGACTAGAGACGCTGGGCGATTCGTTTTTGAAGATGT
TCGCGTCGATATACATCTACTTGAAATTTCCGGCGTACAACGAGGGTGTGTCGACGGCGCTCAAGGGCCGCCTGATCAGCAACAAG
AACTTGTACTATTTGGGCGAGCGGCGCCGGATCGGCGCTACGCTCAAAAACAACGACTTGCAGATCTCCAATTGGTTGCCGCCGGG
GTTTAAGATCCCGGACTTGGTGACAAGGCGCATTGAGAGCAAGGAGGCCGCTCTGGCGTCCCTGTACCACGTGTGGATACCCGTGG
AAGAACAAATGTCCGGAAAACTGTCCGCCGCTACGATCGACGCGATAACGAACGACCGCACGGAGCCGGACCCGAGCGAAGAAGGG
CTGATCAACGAGATCGCGCCCCTCTTCAGGTCCAACCACGCCGGCGACAAGCAGGTGGCCGATTGCGTCGAGTCCCTACTGGGGGC
GTATTTCGAGTATTGCGGGATCCCGGGCGGCCTTAAGTTTCTCGAATGGGTGGGCGTCATTCCAAAGTCGGAACGTTTGGCCGACC
TGCTCGCCGCCGAGGGTAAGAACCCGATCCTGAACCCGGACAGGACGTCCGCGGCCGACATCAATCACCACGTGCCGTTGTGCGCG
GAAATCGAGGCGACGCTCGGGTACCGGTTCCAGAACCGGGGGTACCTTTTGCAAGCGCTGACGCACGCGTCTTACGCTTCCAATCG
GATCACGCACTCGTATGAAAAGCTCGAGTTCATTGGAGATGCGGTGCTCGACTTCCTGATCACGTGCCACATATACGAGTCTTGCG
GGTACCTGACGCCAGGCGACCTGACGGACCTGCGCTCCGCCCTCGTCAACAATAATACTTTCGCGAGCCTCGCGGTCAAGTATAAC
CTGCACAAACACTTGCTGGTGACCAACAGCAAGCTGCAGGACCTGATCGACAAGTTCGCCGAGTACATAGAGTCCAAGGGCTTCGA
GGTCGACGACGAGGTCCTCTCGTGGCTGACAGAGGGCGTCGACGACGACGACTGCTTGAATATTGCCGAGTACATCGATGTTCCCA
AGGTGTTGGGCGATCTGTTCGAATCGATAGCCGGCGCGATATATCTGGATAGCGGCAAGGAGCTGCGCATGGTATGGTCCGTGTTT
CATAGGTTGATGTGCAAAGAAATAGAGGCGTTTAGCGCGAAAGTTCCCAAAAACCTGATCCGCCGTTTGTATGAGTGGCTGCCGAA
TCCTCATCCCAAATTCTGCCGAGCTGTCGATGTGCAGAAAAACAAAGTGATGGTCCCGCTGGAATTTATGCTAGACGGGCATGTGC
AGAGGGTCCACGGATTCGGTTCCAACAAGTCCCTGGCGAAAAAGGCGGCGGCCAAATTGGCGCTGCGTTTCCTGAGCTGAATAAGC
AACTATTAATGTTATGTAGATTTTTTTATGATATAAAACAATTCAATTTTACAGTTAAAAAAAAA

Protein RF 1: 1837-> 6702 (1621AA)

MEVDMETGDHFTPRNYQLELMEIAMKKNTIIYLPTGSGKTFIAVLVLKQMGHCLSKSYSQGGKLSFILVNTVALIDQHADVIKNRTCFKVGRYS
GEMNLDSWSKSKWLEEFDKHQVIVMTVQILANLANSGFIDLNKVNLLVFDECHRGVNDQPMRQLCKLFEHLHDKPRVLGLTATLLNGNCKPNKV
IENVRELEVTYHGQVATVEGLNQVVGYSTNPEEIILQITPHILTTSENRVKSLLKLATEQIRTFKILDPVIEPPSSDLRPLNKNKGLKQLENLI
FDVILQIEWMGAYGGDKSLLAHSIQIERMLKHCSDLSLHKILSYVQLILSYGRQIFHQTMNGHTEYEKIIFFSSDKMRKLIKIFEDYPKTSQEP
SALVFTKRRFTAKVIYYVLDSLSRASTKFKYIKANFMVGNNGNPFMDTREAMYLSKKNRDILQRFNKKEINVLVASNVLEEGVDIATCSLVIKF
EAPEEYRSYIQSKGRARNKSSRYIMLVNGDEMGNFQGRYREYQEIESILNEFLIGKNLGRCEPSDADVNDFYNEDCLRPYFVDSPNSARVTSTS
AISLLCSYCLSLPSDKYTVHAPELFYKTKEGANLKKLHSVVIRMPVICPIDIVTGPFMPSLKLAKRAAALKACEMLHKCRELDDTLTPRKRTVL
EEDVGFLFEHYPAVKEPDAGTNKRKRLHRHAIPPCVKGALPQADPVYLHVIHLTPAFARGENVNTATMYDMYDSALCYGVITPNPAPVICDFPV
YVSAGTINVSLDVNVALISLDERDLDDIRAFNVLVYCDVLRCLKEFLIVDNSEGGVSMWIVPVDRDRGRIDLDTLREYKAVGEIAEPTREEKAN
LEVSLDNYLRKIVAPWYRDSGFYIVTEVTFTKTARSEFPNESFGTYEEYFRDKHNLHLVDPDKPLLYVKSLSKRLNCQKPRGDSKKKRDEKFDD
LEIHLVPELVVKQEFPAPLWVQAGLLPTVLNRLSFLFRLEHLRSTIAREAGLGREIVPIKAPLELDRDLLNYEPRSKNETEANVVGVLPGDGFQ
LHALPALNVNKDYATKVLERDYSWKDIEEPKDVERDIEDVTIMDIEYYEKFIGLPLQEGDVHLRNHRPVIGNQLALTYHKHFVPKPIQLLERKS
VAGPELASIYQAATTAKANDIVNMERLETLGDSFLKMFASIYIYLKFPAYNEGVSTALKGRLISNKNLYYLGERRRIGATLKNNDLQISNWLPP
GFKIPDLVTRRIESKEAALASLYHVWIPVEEQMSGKLSAATIDAITNDRTEPDPSEEGLINEIAPLFRSNHAGDKQVADCVESLLGAYFEYCGI
PGGLKFLEWVGVIPKSERLADLLAAEGKNPILNPDRTSAADINHHVPLCAEIEATLGYRFQNRGYLLQALTHASYASNRITHSYEKLEFIGDAV
LDFLITCHIYESCGYLTPGDLTDLRSALVNNNTFASLAVKYNLHKHLLVTNSKLQDLIDKFAEYIESKGFEVDDEVLSWLTEGVDDDDCLNIAE
YIDVPKVLGDLFESIAGAIYLDSGKELRMVWSVFHRLMCKEIEAFSAKVPKNLIRRLYEWLPNPHPKFCRAVDVQKNKVMVPLEFMLDGHVQRV
HGFGSNKSLAKKAAAKLALRFLS

Comparison with *Tribolium* Dicer-2(1623AA)

```
Query  5      METGDHFTPRNYQLELMEIAMKKNTIIYLPTGSGKTFIAVLVLKQMGHCLSKSYSQGGKL  64
              M+  D   PRNYQ+ LMEIA+++NTIIYLPTGSGKTFIA++VLKQ+   + + YS GGK+
Sbjct  1      MDEEDELKPRNYQVNLMEIAIRENTIIYLPTGSGKTFIAIMVLKQLCAPILRPYSDGGKI  60

Query  65     SFILVNTVALIDQHADVIKNRTCFKVGRYSGEMNLDSWSKSKWLEEFDKHQVIVMTVQIL  124
              S ILVN+VAL+DQH   +++   F VG Y+GEMN+D WS+++W ++F+K+QV++MT QI+
Sbjct  61     SVILVNSVALVDQHGKYVRDHATFSVGTYTGEMNVDFWSEAEWEQQFNKYQVVIMTSQIM  120

Query  125    ANLANSGFIDLNKVNLLVFDECHRGVNDQPMRQLCKLFEHLHDKPRVLGLTATLLNGNCK  184
               NL N+ FIDL KVNL++FDECH GV DQPMRQ+ K F    DKPRVLGLTATLLNGNCK
Sbjct  121    VNLINNRFIDLGKVNLMIFDECHHGVEDQPMRQIMKHFHSCTDKPRVLGLTATLLNGNCK  180

Query  185    PNKVIENVRELEVTYHGQVATVEGLNQVVGYSTNPEEIILQITPHILTTSENRVKSLLKL  244
               +KV++ +R LEVT+H +VATVEGL+ VVGYSTNP+E+     P  L+    +V + L+
Sbjct  181    LSKVMDEIRSLEVTFHSKVATVEGLDVVVGYSTNPQELFKVCQPGALSLDAKQVLNNLRQ  240

Query  245    ATEQIRTFKILD---PVIEPPSSDLRPLNKNKGLKQLENLIFDVILQIEWMGAYGGDKSL  301
                  +    I D    V    S  L+PL   +  LK L NLI D+++ IE +GA+GG  +
Sbjct  241    LINDLEHINIKDEQNSVNLLQSETLKPLEPSDVLKSLRNLISDLMIHIEMLGAFGGHIAC  300

Query  302    LAHSIQIERMLKHCSDLSLHKILSYVQLILSYGRQIFHQTMNGHTEYEKIIFFSSDKMRK  361
              +AH IQIER+ KHC +  L  +L+YV  I+   + +  +TM G+   EKI  FSSDK+ K
Sbjct  301    VAHMIQIERIKKHCQNHQLFIVLNYVMTIMGTTKLLLEETMAGYEPLEKIRKFSSDKVLK  360
```

```
Query  362   LIKIFEDY-PKTSQEPSALVFTKRRFTAKVIYYVLDSLSRASTKFKYIKANFMVGNNGNP  420
             + +I ++Y  K+ +E   LVFTKRRFTAKV+++++D  S+   KF +IK+NF+VGN  NP
Sbjct  361   VFEILDEYKTKSDEELCCLVFTKRRFTAKVLHHIIDKASQVDPKFYHIKSNFVVGNKNNP  420

Query  421   FMDTREAMYLSKKNRDILQRFNKKEINVLVASNVLEEGVDIATCSLVIKFEAPEEYRSYI  480
             + DTRE +Y++KKNR++L  F  KEINVLV+SNVLEEGVDI  C+LVIKF+  E+YRSYI
Sbjct  421   YNDTRENLYITKKNREVLNSFVSKEINVLVSSNVLEEGVDIPKCTLVIKFDKSEDYRSYI  480

Query  481   QSKGRARNKSSRYIMLVNGDEMGNFQGRYREYQEIESILNEFLIGKNLGRCEPSDADVND  540
             QSKGRAR+   S Y  +V    ++  +  +Y  ++EIE+++N+ LIGKN  R  P+ +++ +
Sbjct  481   QSKGRARHIKSLYYTIVETTDVAKYDKKYSAFKEIENLVNDLLIGKNSERDHPNLSEIRN  540

Query  541   FYNEDCLRPYFVDSPNSARVTSTSAISLLCSYCLSLPSDKYTVHAPELFYKTKEGANLKK  600
              YNED L PY+V+ PNSA+V  TSA++LLC YC +L SDKYT +APE +Y  +E ++  K
Sbjct  541   MYNEDKLEPYYVNGPNSAQVNMTSAVALLCRYCSNLASDKYTTYAPEWYY--EEDSSSAK  598

Query  601   LHSVVIRMPVICP-IDIVTGPFMPSLKLAKRAAALKACEMLHKCRELDDTLTPRKRTVLE  659
             L  VVI +PV+CP ID + GP+M + K AKRAAAL AC  LH+C ELD+ L P K+ + E
Sbjct  599   LR-VVIFLPVVCPLIDPIVGPYMHNKKDAKRAAALVACIKLHQCGELDNNLLPWKKQLDE  657

Query  660   EDVGFLFEHYPAVKEPDAGTNKRKRLHRHAIPPCVKGALPQADPVYLHVIHLTPAFARGE  719
              DV +LF H+P  KE DAG  K+KRLH   I P VK A+     +YLH I++ P + R +
Sbjct  658   ADVSYLFTHWPQEKESDAGNKKKKRLHDKEIAPSVKSAIQPDRVLYLHTININPQYKRSD  717

Query  720   NV-NTATMYDMYDSALCYGVITPNPAPVICDFPVYVSAGTINVSLDVNVALISLDERDLD  778
             ++ N  T+YD+Y + L +G+++P P P +C FP++ S GT+ + +  NV  +     ++
Sbjct  718   DLKNAVTIYDLYKTPLKFGLLSPKPLPDLCKFPLFDSNGTLEIEIRNNVREVEFAANEMK  777

Query  779   DIRAFNVLVYCDVLRCLKEFLIVDNSEGGVSMWIVPVDRDRGR---IDLDTLREYKAVGE  835
             ++R F+ LV+ D+L  LKEFLI DN+     M +V   +DR     +D   +R+ K +
Sbjct  778   EMREFHFLVFNDLLEILKEFLIFDNTGMNSEMLLVVPVQDRCGDVCVDFRVIRDNKNLKN  837

Query  836   IAEPTREEKANLEVSLDNYLRKIVAPWYRD-SGFYIVTEVTFTKTARSEFPNESFGTYEE  894
               EP   E+ NL V+ + YL KIV+PWYR     Y+VT+V   K+A S FPN  +  +
Sbjct  838   KLEPAATERINLNVTEETYLHKIVSPWYRSPPKMYVVTKVCPDKSALSRFPNHEYPNFVS  897

Query  895   YFRDKHNLHLVDPDKPLLYVKSLSKRLNCQKPRGDSKKKRDEK-FDDLEIHLVPELVVKQ  953
             Y+ +KH+L ++DP +PLL VK LS+RLN  KPRG   K++ EK +++LE +L+PELV+KQ
Sbjct  898   YYSEKHSLSILDPSQPLLLVKGLSERLNAFKPRGAGGKRKKEKMYEELEEYLIPELVIKQ  957

Query  954   EFPAPLWVQAGLLPTVLNRLSFLFRLEHLRSTIAREAGLGREIVPIKAPLELDRDLLNYE  1013
             EFP+ LW+QA  LP++L+RL++L +L+ L+ IAR G   E +    PLEL+  LL+YE
Sbjct  958   EFPSCLWIQARFLPSILSRLAYLLKLQQLQVDIARGIGAKAEYLKDCPPLELNLHLLHYE  1017

Query  1014  PRSKNET-EANVVGVLPGDGFQLHA---LPALNVNKDYATKVLERDYSWKDIEEPKDVER  1069
             P     T E++    L  +  L      L  + NKD+A K+LE +Y WK IEEPKD+ER
Sbjct  1018  PNDPQLTQESDKSTPLIDNCLALECPKNLRTIQYNKDFAAKMLEAEYYWKTIEEPKDIER  1077

Query  1070  DIEDVTIMDIEYYEKFIGLPLQEGDVHLRNHRPVIGNQL-ALTYHKHFVPKPIQLLERK-  1127
             +I +VT+MDIEYYE FI     +   L+N  PV     + A+TY   F  K +Q+L+ +
Sbjct  1078  NI-NVTVMDIEYYETFISHQPSKTGRLLKNDSPVKQQNVPAITYDCQFEAKQLQILDVQF  1136

Query  1128  SVAGPELASIYQAATTAKANDIVNMERLETLGDSFLKMFASIYIYLKFPAYNEGVSTALK  1187
                 P L  IYQA T A+ANDIVN+ERLETLGDSFLK  AS+YI  KFP YNEG ST LK
Sbjct  1137  DNQSPNLCQIYQALTAAEANDIVNLERLETLGDSFLKFVASLYIIFKFPTYNEGKSTTLK  1196

Query  1188  GRLISNKNLYYLGERRRIGATLKNNDLQISNWLPPGFKIPDLVTRRIESKEAALASLYHV  1247
             G+L+SNKNLYYLG R+ +G  LKN+DL  S+W+PP F IP  +++ I +KE ++ SL++
Sbjct  1197  GKLVSNKNLYYLGVRKNLGGILKNSDLSPSDWVPPCFCIPQTISKAIGNKEYSVVSLFNC  1256

Query  1248  WIPVEEQMSGKLSAATIDAITNDRTEPDPSEEGLINEIAPLFRSNHAGDKQVADCVESLL  1307
              I  EEQ+SG L+  T+  +T +   PD  EE     +        + GDK +AD VE+LL
Sbjct  1257  CISPEEQVSGNLNRKTLSDMTTEEIAPD--EENSYGNMCNFLNKQYVGDKSIADSVEALL  1314

Query  1308  GAYFEYCGIPGGLKFLEWVGVIPKSERLADLLAAEGKNPILNPDRTSAADINHHVPLCAE  1367
             GAYF   GI GG+KF+EW+G++P SE++  L+     +P+LN  +++  D++ H+P   E
Sbjct  1315  GAYFLSGGIQGGIKFMEWIGILPLSEQIQRLIETTQVDPVLN-KKSTKTDVDFHMPQWRE  1373

Query  1368  IEATLGYRFQNRGYLLQALTHASYASNRITHSYEKLEFIGDAVLDFLITCHIYESCGYLT  1427
             IE  LGY F NR +LLQALTH+SY+ NRIT SYE+LEF+GDAVLDFLITC+I+E CG+L
Sbjct  1374  IEQRLGYTFTNRAFLLQALTHSSYSPNRITLSYERLEFLGDAVLDFLITCYIFEHCGHLE  1433

Query  1428  PGDLTDLRSALVNNNTFASLAVKYNLHKHLLVTNSKLQDLIDKFAEYIESKGFEVDDEVL  1487
             PG +TDLRS+LVNNNTFASL V+   HK LL+ NS LQ  IDKFA+Y+  SK + +DDEVL
Sbjct  1434  PGQVTDLRSSLVNNNTFASLVVRCGFHKFLLMMNSNLQGHIDKFADYLASKNYVIDDEVL  1493

Query  1488  SWLTEGVDDDDCLNIAEYIDVPKVLGDLFESIAGAIYLDSGKELRMVWSVFHRLMCKEIE  1547
```

```
                L E     D +NIAEY+DVPKVLGD+FE++AGAIYLDS K+L+ VW VF++++ +EI+
Sbjct  1494  ILLEE-----DEMNIAEYVDVPKVLGDIFEALAGAIYLDSNKDLKTVWRVFYKIIWREID  1548

Query  1548  AFSAKVPKNLIRRLYEWLPNPHPKFCRAVDVQKNKVMVPLEFMLDGHVQRVHGFGSNKSL  1607
              FS  VPKN+IRRLYE      P+F +A++V   K MV L+FM +G  +RVHGFG+NK L
Sbjct  1549  LFSKNVPKNVIRRLYECHTVYPPQFSKALEVGNQKTMVSLDFMCEGRKKRVHGFGTNKIL  1608

Query  1608  AKKAAAKLALRFL  1620
              AK+AAAK+ALR L
Sbjct  1609  AKRAAAKIALRAL  1621
```

Graphical representation

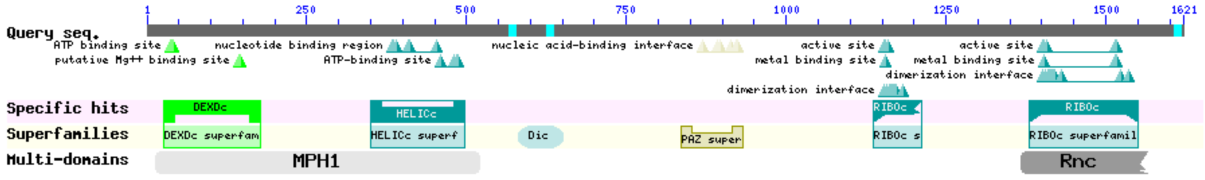

Ago-2

>Cb.comp42256_c0_seq1 len=4381

cDNA

CTCTAATGTTAGCAAGGTCTGTTTAACACGTAAACTTGGCATCGTCGCTGCAATTGTTTTCCGACATTTTATTTTAATATGTGATACGGAAGGA
TATTTTTGTGTTTTAGTGAATTAACTTTATTTGAGAAATACTACATTGTGAAAAATGGGAAAAAAAGGAAAAAAGAAGCCTGAACCTCCTGTGC
CGGGAGCGGCCCAAGGTTCTTCCACAGAAAGGAGGGAACCACAACAGTCTACAACTAGACCTGTTCATTCAGAAGGGCCACCTCAGCAAAAGCA
AGGTCCCCCAGTTAAACAATTTGCTCCACAAGAAGGCCTAGGGGCCAGTCAGGGGACGCAACCGGTCCATCTCGGTCCTCAACAGGGACAAGGG
GGCGGAGGTCGAGGTCGTGGTTGGGGGCGAGGCGGCGGTCCCGGTAGAGACCTGCAGCAACAGCAGCAGCAGCCTTTGGGCCATACACCAGGCG
GGCATCAACATCATACACAGCCTCAAGAATTTGGACAAGTGCAGGGGCATGGAGGCCAGGATCGTGGTCGTGGTCGTGGTTGGGACCGAGGCGG
GGGTCGCGGTAGAGACGGAAGCCAAGGTCGAGGCCTGCAGCAGCAGCAGCAGCAGCCGGTGGGCCAAGTTGTGTACGGCCAGACACCAGGCGGA
CCCCAACAACAAAAAGAGCCTCAAGAATTTGGACAAGTCCAGGCTGGCCGAGGCCATGATTGGCATCAGGGTCGAGGTGGAGATAGAGGTAGAG
GCCAGCAAAGCGAACACCAGGCAGTCCCGTCTGCTCAAGTTTCGGCTAGCCAGCAACCGCAGACGGGAGGTCCGAGGCCCCAGCAGGCTAAGAA
GACGGAACCGGTAGAAAAAATTACACAGGGCTTAGCTAAAGTTCAGCTAGGGGCATCTGACGATTCCGCTTTGGTATCGCAGCAGAAAATCCAA
CCCGGTACACAGGGGCGAATTATAAAAGTGGAGTCGAACCACTTGCAACTGTCGCTGGGTAAATTGAAGGAGGTATACCATTATGACGTCGATA
TCCAACCAGAAACCCCGAGAAAGTTTATGCGGCCCGTGGTTGAGCAGTTCCGTCGGAAACGGTTCCCGACTCGGTATCCGGCGTATGATGGTCG
AAAAAATATGATCACGTCGTTCATGCTCAGCGCCAACATCTATGACATCATCGAGGAGGAGATCGTCATCACCGAGGAGGACGGACGCTCTAAG
ACCTTCAAAATCAAGGTCAAATATGCGAATACCGTTGACTTGACGCCGCTTAGGGACCTCGACAAATCGCCCGTCACGCCTCAGCTCGCACTAC
AGTGCGTCGACATCATACTGAGAAGCGCTCCGCTCACCACATGTATACCGGTGGGCAGATCATTTTTCGTCAAGCCCGATCGGATTATCGATTT
GGGTCAGGGTATGGAAATGTACAATGGCTTCTATCAGTCAGCCATCAGGGGTTGGAAGCCGCTATTAAATGTAGACGTTGCCCATAAGGCATTT
CCGAAAGCGATTAGGGTGATTGACGCCCTGTGCGAACTGTTGAGCGGAAGGCAGCCTGTCAGCGCGGAGGATCTGAGGGGGGGTCTCGATCGAT
ACCAGGCAGAGAACTTTGAAAAATTCATGAAAACGTTGCGCATCAACTATGAAATACCTGGCCACCCGGGTACCAAGCGTGGTTACCGTGTGAA
CGGCATTGGTCAGCCGCCAAACGTGGCCCAGTTCCAGCACCAAGAACGAACGATGACCATCTCAGAGTACTTTGGGACCGTGAAAAACTATAGG
CTAAGGTATCCGGACCTGCCCACACTGTGGGTGGGCAGTAGTCAGCGGGCTGACAAGATCCTGCTGCCCCTGGAACTGTGCACGATTGTCGAGG
GACAGGCATTAAATAGGAAAATGACAGAAATGCAAACAAGGGAAATGATTAGATTTGCAGCCACCAGCACTAAGGTCCGCAAGGAGAAGATCAT
GACGGGCTTGCAGATGGCCAACTACAACCAGAACGCTTGCGTTCGGGAGTTTGGCATCTCGGTCGGTAACGAGTTTCAGAAATTGGACGCACGC
GTACTCAATCCTCCCGAATTGAAATACGCCGGGAGGCAAGTGAAGCCTTGGAAGGGTGTCTGGCGCAACGAGAAGTTTGTGAAACCCGTGGTCA
TTAGCAAATGGACCATTGCTAGCGTGGTGCCCCGTTATGGGCCGCGACCTGACGATTTGAAGCGGATGGCTGATATGCTCTTCAGGGCCGCGAA
CGAGGTGGGCGTAAGATTCGATAGCCCCGCCCAAGAACCCTTCATTGCAGTCCAGCCTCGGCAGGACCTCCAATCGATCATAACCTATTTCAAG
GGGCAAAAGGTTAAGGGTTTCGACCTAATATTTGTTGTGGTACCTAACAGTGGTCCGCAATACTCCTACGTTAAGCAGGCGGCTGAAATAAGTG
TTGGATGTCTCACACAGTGCATCAAGTCGGACACCGTTGGACGCCGAATGAACCCCCAAACGGCCCTCAACATTCTGTTGAAGGTAAATAGCAA
ATTGAACGGCGTGAACCATACGCTGGCCATAGCGCCACCATTGATGAGACGCCCTGTGATGATAATGGGCGCGGACGTAACTCACCCCGGTCCC
GATGCCCAGACGATCCCCAGTGTCGCGGCGGTCACCGCCTCCCACGACCCGAAGGCGTTCCAGTACAATATTTGCTGGCGGCTGCAACCGCCTC
GCGTCGAAATCATTGTGGATTTGGAAGCGATAGTTGTGGAGCAGCTGTTGTTCTTTAACCGGAAAACTAATTGCAAGCCGGAGGCGATCGTGTT
TTTCAGGGACGGAGTGTCCGAGGGGCAATTCGAAGAGGTGAAAAAAGCCGAAATACGGGCGATCCGTAGCGCTTGCAAAAAACTGCAGGCGCAG
GACTACGAGCCAAAAATTACATTCGTGGTGGTGCAAAAGCGGCACCATACGAGGCTGTTTCCGCTGAATGCAAGTGATTCGGAAGATAAGAACA
TGAACGTACCAGCCGGTACATGCGTCGACAAAGATATTACACACCCCTTCATGCAGGACTTTTATTTGGTGTCGCACGCCAGCATTCAAGGCGT
GGCCAAGCCGACCAAGTATTGCACCCTGTGGGATGACAATAATTTGGATAATGATCAAGTGGAGCAACTGGCGTATTATCTTTGCCACATGTTC
ACCAGGTGTAACCGCTCCGTGAGCTACCCCGCCCCCACGTATTACGCTCATTTGGCAGCCGCGAGGGCAAAGGTGTACATAGAAAATGACACAC

```
TGAACATGAGCGCTCTAATGAGGGAATTTGAACGCTACCAGATAAAAGACGAGATCCGCAAAGGACTGCCGATGTTTTTCGTTTGAACTCAGCT
CATCTCAAAATTGATGATTTAATAAAAAACGCGTTTTAACTTTTAGCTATTAGCCCTTTGTTACACTTATTTCTTTCTCTTTTATTAATTGTTA
TTTTTATTTAGAGGATGATTTTAAAAAATATGTTTATGTAATTTAATAATATTCTTGAATAAACTGGTGATTAGCAAGGTACTAGAAAGTTAGC
AGAAAGAACTTATATAGTTGCTTTCATTCTGAGCGTTGAGCAGTCATGAGATACAGCTTACCTAGTGTTAATTGTTCTGAGAGAATCTCTTATC
TCTCAATTTCACCCTAAGAGGTTCCAGAAACTAGATCAAAAATGGATCTCAGAAGCCTAATTCATTCATTCAGATCAGAAGTTCGCATGGCGCA
TGAATAGAAACAGCACAAAAATAAACGCTATAAATAGAAATAGAGTGATTATTATTACAGTAAGGAAAAAAATCTGTGTATTCCTAAAGTACCT
AAAATTCCTGAGAACCTGAGATTAGTTTCTTCTTTGCCCCATCGAAAAAAAAAGTTTTATTTCCAATGACAGGCATAAAGTCTGTTTATAACAG
CGCTCTTTATGGCTGTAGATTTCGATTTAAAAAGCTACAACAAAATTCAACAAATTTTCAGCAGAAGGACCCACCGACGGGGGCGCTGCTATTT
GTAAACAAATATGGTGGAAACCAGGAAGCATATGAAAATGAATACTCTTGTCTCTTCCTTTTCTGTAAGGGCAACCATAAACAGAACAGTTGGT
GTTAAACCTTCGGTTTATTAGTAAACGCTTTGGAAGTCGACCGTTCTTCAGATGCCATTATCAGATAAAACACATCTGTTTTTGTCCAAGTATA
CAACCTACCAATAAATTGTTTTTGTATAAAATTGCTTATACATAAGTCCCAACACACAATATCACAAAAAAATGTATGTATGATCCGAATAAAC
AATGAAAATGACCGGCGTGCGTTTTGAGTATTGCTTTCAAAACCAGCTTACAATTTT
```

Protein RF 2 :149 -> 3376 (1075AA)

```
MGKKGKKKPEPPVPGAAQGSSTERREPQQSTTRPVHSEGPPQQKQGPPVKQFAPQEGLGASQGTQPVHLGPQQGQGGGGRGRGWGRGGGPGRDL
QQQQQQPLGHTPGGHQHHTQPQEFGQVQGHGGQDRGRGRGWDRGGGRGRDGSQGRGLQQQQQQPVGQVVYGQTPGGPQQQKEPQEFGQVQAGRG
HDWHQGRGGDRGRGQQSEHQAVPSAQVSASQQPQTGGPRPQQAKKTEPVEKITQGLAKVQLGASDDSALVSQQKIQPGTQGRIIKVESNHLQLS
LGKLKEVYHYDVDIQPETPRKFMRPVVEQFRRKRFPTRYPAYDGRKNMITSFMLSANIYDIIEEEIVITEEDGRSKTFKIKVKYANTVDLTPLR
DLDKSPVTPQLALQCVDIILRSAPLTTCIPVGRSFFVKPDRIIDLGQGMEMYNGFYQSAIRGWKPLLNVDVAHKAFPKAIRVIDALCELLSGRQ
PVSAEDLRGGLDRYQAENFEKFMKTLRINYEIPGHPGTKRGYRVNGIGQPPNVAQFQHQERTMTISEYFGTVKNYRLRYPDLPTLWVGSSQRAD
KILLPLELCTIVEGQALNRKMTEMQTREMIRFAATSTKVRKEKIMTGLQMANYNQNACVREFGISVGNEFQKLDARVLNPPELKYAGRQVKPWK
GVWRNEKFVKPVVISKWTIASVVPRYGPRPDDLKRMADMLFRAANEVGVRFDSPAQEPFIAVQPRQDLQSIITYFKGQKVKGFDLIFVVVPNSG
PQYSYVKQAAEISVGCLTQCIKSDTVGRRMNPQTALNILLKVNSKLNGVNHTLAIAPPLMRRPVMIMGADVTHPGPDAQTIPSVAAVTASHDPK
AFQYNICWRLQPPRVEIIVDLEAIVVEQLLFFNRKTNCKPEAIVFFRDGVSEGQFEEVKKAEIRAIRSACKKLQAQDYEPKITFVVVQKRHHTR
LFPLNASDSEDKNMNVPAGTCVDKDITHPFMQDFYLVSHASIQGVAKPTKYCTLWDDNNLDNDQVEQLAYYLCHMFTRCNRSVSYPAPTYYAHL
AAARAKVYIENDTLNMSALMREFERYQIKDEIRKGLPMFFV
```

Comparison with *Tribolium* Argonaute 2b (879AA)

```
Query  226   PRPQQAKKTEPVEKITQGLAKVQLGASDDSALVSQQKIQPGTQGRIIKVESNHLQLSLGK  285
             P P    + EP   ++ G      G     ALV    + PGT+GR I++ESNHL L+LGK
Sbjct  40    PEPSSPPRQEPAPPLSGG------GDCLSGALV----VTPGTKGRRIQIESNHLSLNLGK  89

Query  286   LKEVYHYDVDIQPETPRKFMRPVVEQFRRKRFPTRYPAYDGRKNMITSFMLSANIYDIIE  345
             L E YHYDV I P+TP+  +R V+  F RK +P  +PA+DGRKN+ +   L     +  +
Sbjct  90    LTEAYHYDVAITPDTPKCLLRDVMNLFGRKHYPQNHPAFDGRKNLYSPKKLP--FPNDTK  147

Query  346   EEIVITEEDGRSKTFKIKVKYANTVDLTPLRDLDKSPVTPQLALQCVDIILRSAPLTTCI  405
              + +  E + R K FK++VK A TVDLTPL D+ ++  +PQ ALQC+DI+LR+AP   CI
Sbjct  148   SDTIEVEGENRKKEFKVEVKLARTVDLTPLHDIMRTTQSPQDALQCLDIVLRNAPSNACI  207

Query  406   PVGRSFFVKP--DRIIDLGQGMEMYNGFYQSAIRGWKPLLNVDVAHKAFPKAIRVIDALC  463
               GR FF  P   +II LG GME+Y GFYQSAIRGWK LLNVDVAHKAFPKA  V+D +C
Sbjct  208   IAGRCFFTPPRDGQIIPLGDGMELYYGFYQSAIRGWKALLNVDVAHKAFPKASNVLDIVC  267

Query  464   ELLSG-RQPVSAEDLRGGLDRYQAENFEKFMKTLRINYEIPGHPGTKRGYRVNGIGQPPN  522
             E+ S  R  ++  +L   L  +   +FEKF+K L++ YEIP    +KR +RVNG+G+PP+
Sbjct  268   EIGSDFRTTMTRANLSQPLREFVQRDFEKFIKQLKVKYEIPNQSSSKRIHRVNGLGEPPS  327

Query  523   VAQFQHQE-RTMTISEYFGTVKNYRLRYPDLPTLWVGSSQRADKILLPLELCTIVEGQAL  581
              A+F+  + R  T+  Y+  VK  +L+YP LPTLWVGS +R  KILLPLE CT+V GQA+
Sbjct  328   QAKFKLDDGRMTTVERYYQEVKRCKLQYPHLPTLWVGSRERESKILLPLEFCTVVGGQAI  387

Query  582   NRKMTEMQTREMIRFAATSTKVRKEKIMTGLQMANYNQNACVREFGISVGNEFQKLDARV  641
             NRKM E QT  MIR AATST VRK+KIM  L+ ANYN + C+REFG SV N F+KLDARV
Sbjct  388   NRKMNENQTSAMIRKAATSTDVRKDKIMQTLRTANYNNDPCIREFGFSVSNNFEKLDARV  447

Query  642   LNPPELKYAGR-QVKPWKGVWRNEK--FVKPVVISKWTIASVVPRYGPRPDDLKRMADML  698
             LNPP L YA   Q+KP KGVWR ++  F+    I+KWTIAS   RY  R  D  ++ADM+
Sbjct  448   LNPPSLLYADNAQIKPSKGVWRADRNRFLVGATINKWTIASGT-RYPSR--DADKLADMI  504

Query  699   FRAANEVGVRFDSPAQEPFIAVQPRQDLQSIITYFKGQKVKGFDLIFVVVPNSGPQYSYV  758
             FR A+  G++  S A  P   +  RQ L+  I YFKG++   +DLI VVVPNSGPQYS+V
Sbjct  505   FRMASSNGMQITSKAT-PSTHIGGRQGLRDFIDYFKGKQ--DYDLIIVVVPNSGPQYSFV  561

Query  759   KQAAEISVGCLTQCIKSDTVGRRMNPQTALNILLKVNSKLNGVNHTLA--IAPPLMRRPV  816
             KQAAE++VGCLTQCIK  T+GR +NPQT  NILLK+NSK+NG NH L+    P +M+RP
Sbjct  562   KQAAELNVGCLTQCIKERTIGR-LNPQTVGNILLKINSKMNGTNHRLSPNSRPLIMKRPC  620

Query  817   MIMGADVTHPGPDAQTIPSVAAVTASHDPKAFQYNICWRLQPPRVEIIVDLEAIVVEQLL  876
             MIMGADVTHP PDA+ IPSVAAVTASHDP AFQYNICWRLQPP+VEII DL  I VEQL
Sbjct  621   MIMGADVTHPSPDARDIPSVAAVTASHDPNAFQYNICWRLQPPKVEIIEDLCNITVEQLK  680
```

```
Query  877   FFNRKTNCKPEAIVFFRDGVSEGQFEEVKKAEIRAIRSACKKLQAQDYEPKITFVVVQKR  936
             FF +KT  KPE+IVFFRDGVSEGQF++V++AEI AI+ ACK LQ  DYEPKITF+VVQKR
Sbjct  681   FFYQKTGFKPESIVFFRDGVSEGQFKQVQRAEIAAIQKACKMLQKDDYEPKITFLVVQKR  740

Query  937   HHTRLFPLNASDSEDKNMNVPAGTCVDKDITHPFMQDFYLVSHASIQGVAKPTKYCTLWD  996
             HHTRLFP N  DSEDKN NVPAGTCVD  IT+P MQDFYLVSHASIQGVAKPTKYCTLWD
Sbjct  741   HHTRLFPTNPRDSEDKNNNVPAGTCVDTHITNPRMQDFYLVSHASIQGVAKPTKYCTLWD  800

Query  997   DNNLDNDQVEQLAYYLCHMFTRCNRSVSYPAPTYYAHLAAARAKVYIENDTLNMSALMRE  1056
             DNN++ND +E+L Y+LCHMFTRCNRSVSYPAPTYYAHLAAARAKVYIEND L+MS L R
Sbjct  801   DNNMNNDDIEELTYHLCHMFTRCNRSVSYPAPTYYAHLAAARAKVYIENDKLDMSQLKRH  860

Query  1057  FERYQIKDEIRKGLPMFFV  1075
              E+ QI+++I KG PMFFV
Sbjct  861   QEKCQIQEKIVKGKPMFFV  879
```

Graphical representation

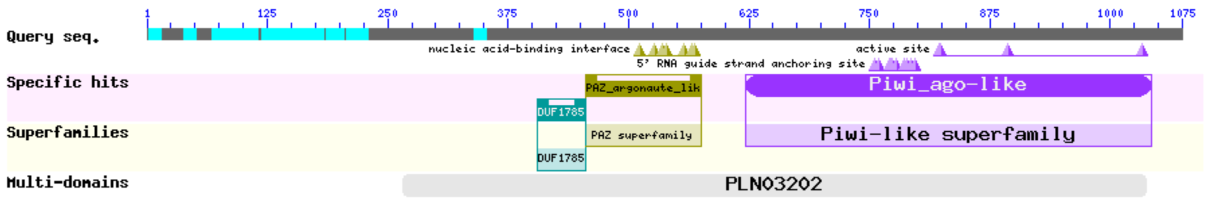

R2D2

>Cb.comp39376_c0_seq2 len=1397

cDNA

```
ATTTTATGTGGCCCGACCTAGTGTATAACGTAAACGAAGAAGTGTCCAACTTAAGAACGTATATAGAAGAAAAATAAATGTTTTAAATTCGTTC
CAATCGGCATAGGTCTATTTTCTTCGGGCCTCTGTATGTGATGTGATGTTGGACAGAAGCGTTTAAATATTATTGCAATGAAAACCCAACCTTA
TTTTATAATAATTTAATAAAAACCCTGGTAACAACGCTTCAAAAAATGGCGAATACCAAAACTCCCGTTATGGTATTGCAAGAGCTAGCAACGA
AGAAAGGCTTTGCACCGCCTGATTATAAAATTGTTAGAAGTGTATCTGGAACACACTTGAATCGTTTCGATTTTGTAGTATCTGTAGGCGGAAT
AGTGGCCGAAGGAAGCGGATCTTCCAAACAAATTGGTAAACACGAAGCGGCACACAACGCCTTGATCCAATTACAAGAAATGGGTGTATACAAT
CCAGCTGTTAACCCGGTAACAACATTCAAGGCTGCTCTGAGGGACAGTGATAGTTCTTATAAGACTACTATTAATTGTATTGTAAATTTGCAAA
ATCTTTGCCTCGAACATAAAACGCCTCCTCCATTATTCACTGAAATTTCGTCGGTTGGTCCTCCCCATGCGAGAGAGTTCACTTATGAATGCAA
AGTTGGGTCGTTAGTAGCACAAGCCAAAGGCAATAGTAAAAAAATGGCCAAACAATTAGTGGCCAAAGAAATGCTCGATAGGGTGACAGGCGTT
TTACCAGAGCTGTCTGCGCAACTGGAGGATAGCCGCAACGCGCTTACGGAACTAGACGAGAAAGCCACAGTTAGATACAATGAGTTTCGTGGCG
TCATTCCTGACAAAACCGTAAAAGTGAGCTGCATGTCTCACACCTTCAAAAAATTAATGATGCAAAGAGACCTTACCTACGAGGATCGTTTCGA
AAAATATTTCGAACACGCTTCGGAAGATAACCTCGAGAAGATTCTCGACAAACTCGAATTAAAACACGAAATCGAAGTGATGCAAGAAACGCCG
CCGATAGTCATCCTTAGCATTAATACCGACACGTCCTTTACGCTTATCAGTGGAGGCAAAACGCATGCGGAAGCAAGGGTTTTAGTCCTAAAAC
AGGCTTTCGAAATAATCCAGAGTTTTATGCAAATTGATTTGGATTCCATATAACAAATTTAATCAATATTGATTCGCATTTGACATGATTTTGA
TTGATTTTTCGTTAAATAAACTTGATTCGTATTCTATTTGATTGTGATTGATTTCTTTTGTTAATTAATATTGACTCGCATTTGATTTGATTGA
TTTCTCACGTTGATTAATACTGATTCGCATTTAATTTGATTGTGATTGATTTATTTCGTTAATTAAATAGATTAGCATTTG
```

Protein: RF3: 243 -> 1181 (315AA)

```
MANTKTPVMVLQELATKKGFAPPDYKIVRSVSGTHLNRFDFVVSVGGIVAEGSGSSKQIGKHEAAHNALIQLQEMGVYNPAVNPVTTFKAALRD
SDSSYKTTINCIVNLQNLCLEHKTPPPLFTEISSVGPPHAREFTYECKVGSLVAQAKGNSKKMAKQLVAKEMLDRVTGVLPELSAQLEDSRNAL
TELDEKATVRYNEFRGVIPDKTVKVSCMSHTFKKLMMQRDLTYEDRFEKYFEHASEDNLEKILDKLELKHEIEVMQETPPIVILSINTDTSFTL
ISGGKTHAEARVLVLKQAFEIIQSFMQIDLDSI
```

Comparison with *Tribolium* R2D2 (320AA)

```
Query  3     NTKTPVMVLQELATKKGFAPPDYKIVRSVSGTHLNRFDFVVSVGGIVAEGSGSSKQIGKH  62
             NTKTP MVLQE   K+GF+PP+Y +V S +GTH N F + V+V  +   G G SKQ+ KH
Sbjct  5     NTKTPAMVLQEFTMKRGFSPPEYILVMSKTGTHENEFHYKVNVANVCGLGFGRSKQVAKH  64

Query  63    EAAHNALIQLQEMGVYNPAVNPVTTFKAA--LRDSDSSYKTTINCIVNLQNLCLEHKTPP  120
              AA  AL  L E G+Y+P+ NPV  F A     +SDS  K  +N I NL+++C E K P
Sbjct  65    NAASKALEILAEQGLYDPSSNPVQEFNAQSHRNESDSPQKPPVNFIGNLKDMCCEFKLPY  124
```

```
Query  121  PLFTEISSVGPPHAREFTYECKVGSLVAQAKGNSKKMAKQLVAKEMLDRVTGVLPELSAQ  180
            P F EIS VGPPH REFTYEC + S+  QA  N+KK AKQL A+EML+++    P+L+ Q
Sbjct  125  PEFKEISDVGPPHCREFTYECCIASITTQATANTKKQAKQLAAREMLEKIRETCPQLAEQ  184

Query  181  LEDSRNALTELDEKATVRYNEFR---GVIPDKTVKVSCMSHTFKKLMMQRDLTYEDRFEK  237
                 N++     +   +Y+E      V+P++ V +   S   K+ M  +++ +ED F+K
Sbjct  185  FAAESNSILADSHEVIKKYSELSTTLDVMPNRAVLIEDYSTAIKRRMEDKNVCFED-FQK  243

Query  238  YFEHASEDNLEKILDKLELKHEIEVMQETPPIVILSINTDTSFTLISGGKTHAEARVLVL  297
             ++   ++ L+ I +KL+++++I++ QE+PP+       DT FT+++ G +   A   ++
Sbjct  244  QYKLKDKEGLDYIFEKLDIRYQIDLFQESPPVYCALFGLDTPFTVMAVGSSQENAMANLV  303

Query  298  KQAFEIIQSFM  308
             + + +++ +M
Sbjct  304  FEIYRLLEIYM  314
```

Graphical representation

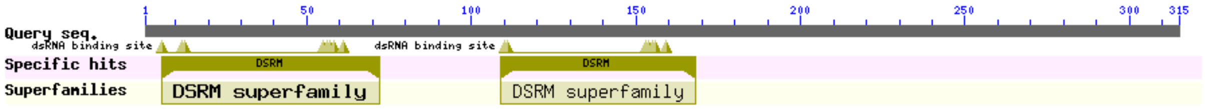

miRNAi pathway *Cylas brunneus*

Dcr-1

>Cb.comp39694_c0_seq3 len=6925

cDNA

```
GGCGAAGCAAGGAAGGTTCTATGGTCATTGTGACTGAAATTTGGGCTTTGTTATTAACAAAACCACAACACAGAAGAAACAGGTTA
TTAGTGAAACCCTGTTCGAAAAAATCAATATAATTTAGAAATACGAAAAGAATTCCGCAAAATGGGTAAATTACTAGGTTAAATTT
GTGACATAACCTACAAACTAAAACGAAAACATAACCTATGTTTTTTTGCTTTGTAACTTTAAATTTAGTAAGCTATTAAAAATGGC
TTGCTATATTAATGAAAACGTTTATACGCATACATTTACCCCTAGAGAATATCAGGTTGATTTGTTAGACTCTGCGAAAAAACGAA
ATACCATAGTGTGTTCCAGTACAAGTTCTTCGAAAGCATTTATTGTAGTAAAGTTATTACAAGAACTCTCTTCTCAGATGCGCGGA
AATCAAAGAAAGAAGGCTCTGTTAATCTTAGATCCACAAAATGTGCATGTCATGGTTTCCCATGTGAAATTATTGACTGATTTAAC
TGTAGTTAATATCGATACTCAGATTCTTGAAGACATTAACAGATATTTCCAAGATAATCAAGTCATTGTTACAACAGCAGAAATTT
GTATAAGCAGTAATATTTTATTAGATTTAAAATCTTACAACTTGCTTGTTATTGATGATTGCTTATATGGAAAAAAGCAAATTATG
ACTAAACAAATAATGAGCAAATATCATGATTTAGATCCTAAAGAGCAACCTAGAATTTTAGGACTGACAACGGGACTTCTTGGATC
CGAATTACGACCTGAAAGATTAGAAGCAGAATTTCAAAGGTTAGAAAAACTTCTGAATGCCATTGTAGACACATCTAGTGAAATAG
TGACTCTAATTCGTTTATCTTGCCATCCTCGCGAATCGATAATTCAGTGCTCCTCATTGAATTATTTTCAAGTTCAGGAAGAATTA
ATAAAAATTGTTAAAGATTGTATTGAATTTATTAACGAACATAGGTATGATCCCAGTGAAATTTATGAAGATGAGTTCTTAGAAGA
ATATAAGGACATTCCGGATCCAAAAATAACTCCTTTGGAGTTATTGCATGATTACCTGTCTATTTTGGAAGATTTGGGCCCGTGGG
GCGCTGATAGGGCAGCAATGAATTTGCTTTCTAAAATAGAAAAACTAAAGGTGAAGACCCCTTATGAAAGGCACTATCTTTTACTT
TGCACTGTATCAACCACTTTAGTCCGTACTAGAGCTGTTTGTGACTATGAATTTGAAAAATGTGATAGTGAAATAGCAAAACTTAA
ACAGTTCTCAACATGGAAAGTCACAAAACTGATTAACATTTTAAGACAGTTTAAGCCTGCTGGAGAGAAGCCCAAGATTAATGAGG
AAGACAAATCATCTGATTTACATAACTCGAATTTTAAGAAATCAAAAGGCAAACCTAGGAGATTTCAACACCGACCACAATTTGAA
GATATGCTGTGTGCTCTTATTTTCGTCGAAAATCGATACAAAGCCAAAGCATTATTTGGGCTACTATGTACGCTTTCACAATTTGA
CGATGACTTCTGGTGGATATCCGCATTATTCTCTGTTGAAAAAATGGCAGATATTATGAAAGAGCCCCACGAAGCTGAACGTGAAC
ATAAAATACAGGAAGAAGTATTGAGAAAGTTTCGATGTCATGAATGCAACATTTTGATTTCGACGTCAGTACTAGAACAAGGCTGT
GATTTACCAAAGTGCAATCTTGTAATAAGATTTGATCTACCTAAAACATTCCACAGTTACATTCAGTGCAAAGCAAGAGCTAGAGC
ACCCGAAGCGCATTACATATTATTTGCCAAAGATGATGAAATAGAAAATTTTGTCACAAGTTTAGCGCAATATAATGAAGTGGAAA
ATACACTGCTAAAACGTTGTTCCAGTCTTGAACCAGATAAAAGAGAAGAACAACTAGCGGATAGCTTTTCAAAGTGTTGTAAGCCT
TACCAACCCTTAAAGGAGGAAGGATCGCCTAGCGTGACTTTGTTTAATTCTATATCACTTGTTAATAAATATTGCGCAAAACTTCC
TAGCGACACATTTACCCGTTTAACGCCAATTTGGTATGAGGAGAAATTAGGTGATAGTTTTGTGTGTCATATAAGACTTCCCATCA
ATTCTCCTGTAAAACAAGTTGTAACCAGTCCTCCCATGCCAAACTCTTTATTGTCGCGAAGAGCTGCAGCATTTATGGTGTGCCAG
TATTTACACAAATATGGTGAATTGGACAATCAATTGCAACCAATAAATAAAGAAAATTTTGTTCCCGCCGAGGAGGATTGGAATAA
CATCCCCCTTGATGAACAGTACGATGAAACTTCAGAAGTTAGACCAGGTACAACAAAAAGAAGACAATATTATTATAAAAAAGTTG
CTGATGCATTAATACATTGCCATCCGATAAATGGGCAGCCAACATATTTTTACCAAATCGTTATGACATTGACGTGCCCTTTACCT
GAAGAGCAGAATACAAGAGGCAGAAAAATTTACCCACCCGAGGAATCCCTTCAAGGTTTTGGGATTTTAACCAACAGGGAAATACC
AAAGATAAGTGCTTTTCCAATATTTACAAGATCTGGTGAGGTGGAAGTTGAGCTTCAGCTCTGTTCAAAACAAGTCATTGTTGACG
AAAAGCAAACTCACAAAATTCAAGAATTTGTCAACTACACATTTACTACCGTACTGAGATTGCAAAAATATCTAATGCTGTTCAAA
CCTGAACTGTCTGACAAAAACTACCTAATAGTTCCTACTATTAAAACTGATAAAGGAATTAATGTTGATTGGGACTTTATAGATCT
CATCTATGATAATTTACACTTTGTTCCAAAAATGATACCGGATGAGGAGCGAAAAGATTACATATTTAATATTGACGATTATCAAG
ACGCAGTAGTTATGCCCTGGTATCGCAATCAAGACCAACCACAATACTTTTATGTAGCTGAAATTTGCTCATTTTTAAATCCCACG
TCAGCTTTTCCCGGTTCCGAATACAAATCGTTTGAACAATATTATTTATTAAAATACGGTATTCAGATTCAGAATCTGGAACAGTA
TTTGCTCGATGTGGATCACACTTCAGCGCGACTTAATTTTCTTACGCCAAGATATGTAAATCGAAAAGGCGTCGCTTTACCCACAA
GCAGCGAAGAGACCAAAAGGGCCAAGCGCGAAAATTTAGAACAAAAGCAAATTCTGGTACCAGAACTTTGTTCTATTCATCCATTT
TCGGCTTCTCTGTGGAGGAAAGCTGTAGCCTTACCGTGCATTCTCTACAGGATTAACGCCCTCTTGCTAGCTGATCAAATTCGAAC
AATTGTTGCTTCGCACTTAAATTTGGGCAAGACAAAATTAGATGATGACTTTAAGTGGACTCCATTAAATTTCGGATGGAGTTTAT
CTGATGTATTGAAGAAATCAAGGGAAGACGAAATGCGGAAACAAGAAGAGAAATTAAAATCGTTGAGGGAAATGGCCGAAGAGAAA
CTGGACGAGTTAAAAATAATCAATTTGAATCTAGATTATGATACCTTAAAATCGGATACCAATAGTTTGGATGAAGACGACGAAGA
GGATAGTTCCACAAATAGTAAATGGGTGGTCGGAACTTGGTCTAACGAAATGGCTCAATCCAGTCAAGCAGATTCTTCAGCTCTCG
TAAGGTATGTGTCCCCAACTAGCTGGTTACAGGGAAACACTTACGACGATGACTTTTCTGATGATATGTCGGATAATTCCGATATT
GATTCGGAAGATTCCATTCACGAATGGGGTAAACTGCGTATCGAATTTACCGGAGATCATCAGGCTGAAGCGTTAGACGATGGGGA
GTCTAAAGAGGACGAGCTGTTTTTTGTTGAAGATAAGAATGCTTGGAAAGTCGACGACAATAGTCTCGAAACTGATAGATTGAGAA
ACGAATTTACCAAAGCGTGTCTCAGAAATAAGGAACACATTTGGTCAAGTGGAATATTGATTAAAAACGGCGAAGTTTTCGAAAAA
AAATCGCAGAATAATCTGGCAAATGATATTTTTCCCGTTTGTCCCGAGAATTTTGATTTTGACTCACTAATGATTGGGTCTATTAA
TATCGAACAGACGCCCGCTAGCCCGCAAGAACCGTTCGTTAATACTTATGACATATCAGAGGAGCGGCAAAATTTTAGTTTTGACG
AACAGCCAGATTTAGATACTCATCCAGGCCCAAGTCCAAACGTTCTCTTACAAGCGTTGACGATGTCGAACGCAAATGACGGAATT
AACTTGGAGAGGCAAGAGACTGTGGGCGATTCATTTCTCAAATATGCAATAACAACTTATCTATACAAAACTTACGAAAATATTCA
CGAGGGTAAATTGAGTCACTTACGTTCCAAACATGTGAGCAATTTAAATCTGTACAAGTTGGGCAAGTTGAAAAATTTGGCCGAGT
ATATGGTTGCGACAAAATTTGATCCGCACGATAATTGGCTTCCTCCTTGTTTTTATGTTCCAAAACAGCTCGAAGATGCACTTATC
GACGCACAATTCCCTGCTAATTGTTGGACTGTGGCTGACATGGCAGCAACTAGAAATATGACCATTGATGAAATATGTACTTTGGT
AAGGGAACGGGGAATTTACTCTCTTCCAAATATTATCCCTTACAATCTAGTAACCCAACATAGTATTCCTGATAAAAGTATAGCAG
ACTGTGTGGAAGCTCTGATTGGCGCCTATTTGATCGAATGTGGGCCCAGAGGTGCCTTGCTATTCATGGCTTGGCTTGGGATCAAA
GTTTTACCTAAAGATCAAGATGGCAACTACGGACATTTAGATTTTCCAAAATCTCCTCTGCTAAGAAATATACCAAATCCGGAAGA
AGAATTAGAGAAATTGTTAGACGGTTATGACGCTTTTGAAAAACATATTGGATATAAGTTCAGAGATCGCGCGTACTTGTTACAGG
CCTTCACGCATGCTTCTTATTCCCCCAATCGATTGACAGACTGTTATCAAAGATTGGAATTCCTGGGCGATGCTCTTTTAGATTTT
ATTATAACAAAAGCCCTTTTTGAAGATATCAGAATGCACTCACCTGGAGCTCTAACCGATTTAAGATCTGCTCTGGTTAACAACAC
AATTTTTGCTTCTTTAGCCGTGAAACACGGATTTCATAAATACTTTAAGCATCTTTCTCCAGGATTAAATGAAGTTATCGAAAGGT
TCGTCCGACTACAAGAAGAAAGTGGCCATACTATTGTCGACGAACATTATCTTATTGATACGGAATGCGAAGAAGTTGAGGACATT
GAAGTGCCAAAGGCCCTGGGTGATGTTTTTGAATCAGTGGCCGGGGCCATTTATCTAGATTCGGGAATGTCACTGGATGCCGTATG
GAAAGTTTATTACAGAATGATGAAAGCAGAGATAGAACAATTTTCCAATAAAGTGCCTAAATCGCCTATTAGAGAACTTCTTGAAC
TTGAGCCTGAAACTGCCAAGTTCGGACGCCCGGAAAAATTAGCAGATGGAAGAAGAGTAAGGGTAACAGTTCAAGTGTTCGGAAAA
GGTACTTTTAAAGGTATCGGACGGAATTACAGAATAGCCAAATGTACCGCTGCCAAATGTGCATTGAAACATCTGAAACGAAGAGG
ATTGCTCAGAAGCAAAAACGATATTTAAAGATATATAATATGTGCCATAGTGTAAATTTTCTTCAAATAAGCGCACATCTTATTCT
```

GGCATTAGTTTGTACAAAAGGAACTTAAGAAAAAGCAAACACAAAAATGTTTTCTCATTTACGTACTAACTGATCTCAAACCATTA
CCACGTCCTGATAGGCCAAATTTTTAGAAAGACCTCAAAATATCTTGTTTGTTTCTAAGGTGGCCGTCAGAGATGATTATTTTTCT
AGTCAATGATGTCCTAGAACTTTTAATTTATCTCCCAATTATTCGTAAACAATTTGGTAGGCTAGGTAAACAATCTTAGTGGCAAG
AATTGTTTGTAAATTAAATATCGAGGTAGTTTTTATACCAATAGGGACTTTTAAAGCACCGAGGGAGTCAAATAAAAACTCAGTAT
TAGCCGGACTATAGGACACAATTTACACTTAAAATGTATATCACAAGTATTTTGAGTTCTACTTTTAAAAATAAGTTTTAAATATT
TGTGCAATTCACATACCAAGAAATATTTTAATGAAGGTTCTGTATTATACTCAAAATATACGATATTTCCGCGAGCAAAACTGCTT
AGTAAAAAAACAGACTTCATATTTTATAAAACTTAATGAATTAGAAAACCTTCCTTAAATAAAACATTTATAGCAAGGCTGTGAAC
TGGGAAAGGTAATGCCTTTGCTTCCAGAGGGACAGAAATAAGAAAGTTTTTTTCCATGACTTTAGAACGCAGGATGTACCTCACCA
AAGATGCGAGAATTTTTGCTTGCATAAAAAGCCACAGCGTCGCAGTGGTAGAGATGGCCCGTTTTAATAACGTTTTTTTTTTATAT
AAATCTTGGGTATGTAGTTAGGTAAACAAATTTACAAACTGTTACTCTATACAATATGCAACTGTCTTGTATCGGGTAAGTATTTG
TTACATTCCTTTTGCATATTGCCAAATTGCTACTCCGCCAATTAGTTTATTCGTCTATTATTTTTTAATTTCTCAAAAGTAGTACA
AAGTTTATGTTTATGTCTTTAGTCAAAACGATTTTTTAGATATATATGTTACAATGTGATAAGTGGCAGCTTGTGATATCACTGCC
CACTTTTTTAATAAGTTAATTTTAAGTACACATTCGGTTTAGTTTTGTCTAGAATTGTTTTTTTCGTTTTTTGTAGCTTACTTATT
TTAATAAAGTTTTGTATTAAAGTTAACGATAATATTTTAATCAAA

Protein

RF 2: 254 -> 5704 (1816AA)

MACYINENVYTHTFTPREYQVDLLDSAKKRNTIVCSSTSSSKAFIVVKLLQELSSQMRGNQRKKALLILDPQNVHVMVSHVKLLTDLTVVNIDT
QILEDINRYFQDNQVIVTTAEICISSNILLDLKSYNLLVIDDCLYGKKQIMTKQIMSKYHDLDPKEQPRILGLTTGLLGSELRPERLEAEFQRL
EKLLNAIVDTSSEIVTLIRLSCHPRESIIQCSSLNYFQVQEELIKIVKDCIEFINEHRYDPSEIYEDEFLEEYKDIPDPKITPLELLHDYLSIL
EDLGPWGADRAAMNLLSKIEKLKVKTPYERHYLLLCTVSTTLVRTRAVCDYEFEKCDSEIAKLKQFSTWKVTKLINILRQFKPAGEKPKINEED
KSSDLHNSNFKKSKGKPRRFQHRPQFEDMLCALIFVENRYKAKALFGLLCTLSQFDDDFWWISALFSVEKMADIMKEPHEAEREHKIQEEVLRK
FRCHECNILISTSVLEQGCDLPKCNLVIRFDLPKTFHSYIQCKARARAPEAHYILFAKDDEIENFVTSLAQYNEVENTLLKRCSSLEPDKREEQ
LADSFSKCCKPYQPLKEEGSPSVTLFNSISLVNKYCAKLPSDTFTRLTPIWYEEKLGDSFVCHIRLPINSPVKQVVTSPPMPNSLLSRRAAAFM
VCQYLHKYGELDNQLQPINKENFVPAEEDWNNIPLDEQYDETSEVRPGTTKRRQYYYKKVADALIHCHPINGQPTYFYQIVMTLTCPLPEEQNT
RGRKIYPPEESLQGFGILTNREIPKISAFPIFTRSGEVEVELQLCSKQVIVDEKQTHKIQEFVNYTFTTVLRLQKYLMLFKPELSDKNYLIVPT
IKTDKGINVDWDFIDLIYDNLHFVPKMIPDEERKDYIFNIDDYQDAVVMPWYRNQDQPQYFYVAEICSFLNPTSAFPGSEYKSFEQYYLLKYGI
QIQNLEQYLLDVDHTSARLNFLTPRYVNRKGVALPTSSEETKRAKRENLEQKQILVPELCSIHPFSASLWRKAVALPCILYRINALLLADQIRT
IVASHLNLGKTKLDDDFKWTPLNFGWSLSDVLKKSREDEMRKQEEKLKSLREMAEEKLDELKIINLNLDYDTLKSDTNSLDEDDEEDSSTNSKW
VVGTWSNEMAQSSQADSSALVRYVSPTSWLQGNTYDDDFSDDMSDNSDIDSEDSIHEWGKLRIEFTGDHQAEALDDGESKEDELFFVEDKNAWK
VDDNSLETDRLRNEFTKACLRNKEHIWSSGILIKNGEVFEKKSQNNLANDIFPVCPENFDFDSLMIGSINIEQTPASPQEPFVNTYDISEERQN
FSFDEQPDLDTHPGPSPNVLLQALTMSNANDGINLERQETVGDSFLKYAITTYLYKTYENIHEGKLSHLRSKHVSNLNLYKLGKLKNLAEYMVA
TKFDPHDNWLPPCFYVPKQLEDALIDAQFPANCWTVADMAATRNMTIDEICTLVRERGIYSLPNIIPYNLVTQHSIPDKSIADCVEALIGAYLI
ECGPRGALLFMAWLGIKVLPKDQDGNYGHLDFPKSPLLRNIPNPEEELEKLLDGYDAFEKHIGYKFRDRAYLLQAFTHASYSPNRLTDCYQRLE
FLGDALLDFIITKALFEDIRMHSPGALTDLRSALVNNTIFASLAVKHGFHKYFKHLSPGLNEVIERFVRLQEESGHTIVDEHYLIDTECEEVED
IEVPKALGDVFESVAGAIYLDSGMSLDAVWKVYYRMMKAEIEQFSNKVPKSPIRELLELEPETAKFGRPEKLADGRRVRVTVQVFGKGTFKGIG
RNYRIAKCTAAKCALKHLKRRGLLRSKNDI

Comparison with *Tribolium* Dicer-1 (1835AA)

```
Query  1      MACYINENVYTHTFTPREYQVDLLDSAKKRNTIVCSSTSSSKAFIVVKLLQELSSQMRGN  60
              MACY+NENVYTHTFTPREYQV+LLDSAKKRNTIVCSS SS+KAFI +KLLQE S +MR
Sbjct  1      MACYLNENVYTHTFTPREYQVELLDSAKKRNTIVCSSASSAKAFITIKLLQEFSHKMRVP  60

Query  61     QRKKALLILDPQNVHVMVSHVKLLTDLTVVNIDTQILEDINRYFQDNQVIVTTAEICI--  118
                K+AL +LD  NV +M SHVKLLTDLTV +ID    E+     + + VIVTTAE+C+
Sbjct  61     HGKQALFVLDGPNVPIMTSHVKLLTDLTVTSIDK---EENPPSLKASNVIVTTAEVCVLL  117

Query  119    SSNILLDLKSYNLLVIDDCLYGKKQIMTKQIMSKYHDLDPKEQPRILGLTTGLLGSELRP  178
                   + L SY L+VID CLYG +Q + ++IM++Y  +    +PRILGLT GLLGSE++P
Sbjct  118    CKKNFVHLDSYALIVID-CLYGGQQSLVREIMARYQAIQ-APRPRILGLTAGLLGSEMQP  175

Query  179    ERLEAEFQRLEKLLNAIVDTSSEIVTLIRLSCHPRESIIQCSSLNYFQVQEELIKIVKDC  238
              +RLEAE QRLEKLL++ VDTSSEI+TLIRLSC PRE I++C       +Q+++   +  C
Sbjct  176    DRLEAELQRLEKLLSSSVDTSSEILTLIRLSCRPRERIVECFKPIPSPLQDKIKATITSC  235

Query  239    IEFINEHRYDPSEIYEDEFLEEYKDIPDPKITPLELLHDYLSILEDLGPWGADRAAMNLL  298
               +F+ +HRYDPSEIY+D+ LEE+K +PDPK  PL    D+L IL+DLGPW ADRAA  +L
Sbjct  236    QDFLKDHRYDPSEIYDDDLLEEFKQVPDPKEQPLSFFDDFLEILDDLGPWSADRAAYGML  295

Query  299    SKIEKLKVKTPYERHYLLLCTVSTTLVRTRAVCDYEFEK-CDSEIAKLKQFSTWKVTKLI  357
               KIEKLKVK PYERHYLLLC  S+ LV  RA+C+ EF+   D E  K+ +FST KV + +
Sbjct  296    IKIEKLKVKVPYERHYLLLCVASSVLVSIRALCELEFQDYTDKE--KVFRFSTPKVLRFL  353

Query  358    NILRQFKPAGEKPKINEEDKSSDLHN--SNFKKSKGKPRR-FQHRPQFEDMLCALIFVEN  414
               +L+QFKP G+KP+    DK  DL +      K+   PRR +  R Q ++MLCAL+FV+N
Sbjct  354    QVLKQFKPTGDKPETC--DKLPDLKDPKKGKGKNYKGPRRPYISRAQSDEMLCALVFVKN  411

Query  415    RYKAKALFGLLCTLSQFDDDFWWISALFSVEKMADIMKEPHEAEREHKIQEEVLRKFRCH  474
```

```
                RYKA+ALF LLC +S+ D+++WW+S  FSV K+AD ++EP EAE EHK QEEVLRK+R H
Sbjct  412     RYKAEALFALLCVMSKSDEEYWWVSVSFSVNKIADPVREPREAESEHKRQEEVLRKYRSH  471

Query  475     ECNILISTSVLEQGCDLPKCNLVIRFDLPKTFHSYIQCKARARAPEAHYILFAKDDEIEN  534
               ECNI+I+TS LEQGCDLPKCNLVIRFDLP++FHSYI  KARARA EAH++L A ++E+ +
Sbjct  472     ECNIMIATSALEQGCDLPKCNLVIRFDLPQSFHSYIHSKARARANEAHFLLLANENEVSD  531

Query  535     FVTSLAQYNEVENTLLKRCSSLEPDKREEQLADSFSKCCKPYQPLKEEGSPSVTLFNSIS  594
               FV +LA+YNEVENTLLKRC SLEPDK EE +AD+ S  C+PYQP  E G+ SV+L N+I+
Sbjct  532     FVENLAEYNEVENTLLKRCYSLEPDKNEELVADASSLQCRPYQPSAEPGANSVSLSNAIA  591

Query  595     LVNKYCAKLPSDTFTRLTPIWYEEKLGDSFVCHIRLPINSPVKQVVTSPPMPNSLLSRRA  654
               LVN+YCAKLPSDTFTRLTPIW+EEK+ + ++C IRLPINSPVK+ VTSPPM N+LL+RRA
Sbjct  592     LVNRYCAKLPSDTFTRLTPIWHEEKVENGYICSIRLPINSPVKKTVTSPPMINTLLARRA  651

Query  655     AAFMVCQYLHKYGELDNQLQPINKENFVPAEEDWNNIPLDEQYDETSEVRPGTTKRRQYY  714
               AAFM+CQ LHK GELD+ LQPI KENF   EEDWN+  L+E  +E  + RPGTTKRRQYY
Sbjct  652     AAFMICQLLHKAGELDDNLQPIGKENFKVNEEDWNSSALEESDEENLDPRPGTTKRRQYY  711

Query  715     YKKVADALIHCHPINGQPTYFYQIVMTLTCPLPEEQNTRGRKIYPPEESLQGFGILTNRE  774
               YKKVADAL+ CHPI GQPTYFY+IVM LTCPLPEEQNTRGRKIYPPE+S QGFGILT++E
Sbjct  712     YKKVADALLDCHPIIGQPTYFYKIVMKLTCPLPEEQNTRGRKIYPPEDSPQGFGILTSKE  771

Query  775     IPKISAFPIFTRSGEVEVELQLCSKQVIVDEKQTHKIQEFVNYTFTTVLRLQKYLMLFKP  834
               IPKISAFPIFTRSGEV V+LQLCS Q+IV E Q  KI+EF+NYTFT+VLRLQKYL LF P
Sbjct  772     IPKISAFPIFTRSGEVSVDLQLCS-QLIVTENQICKIREFLNYTFTSVLRLQKYLTLFNP  830

Query  835     ELSDKNYLIVPTIKTDKGINVDWDFIDLIYDNLHFVPKMIPDEERKDYIFNIDDYQDAVV  894
               + S  +YLIVPTI       VDWDFIDLIY NL  +P++IP+E RK Y F+ + Y+DAVV
Sbjct  831     DASANSYLIVPTID-GATTTVDWDFIDLIYANLTVLPEIIPEEVRKSYEFDPEKYRDAVV  889

Query  895     MPWYRNQDQPQYFYVAEICSFLNPTSAFPGSEYKSFEQYYLLKYGIQIQNLEQYLLDVDH  954
               MPWYRNQDQPQYFYVAEICS LNP S FPGS+Y +FE+YYL KY IQIQN  Q+LLDVDH
Sbjct  890     MPWYRNQDQPQYFYVAEICSNLNPASDFPGSDYATFEEYYLRKYSIQIQNKSQHLLDVDH  949

Query  955     TSARLNFLTPRYVNRKGVALPTSSEETKRAKRENLEQKQILVPELCSIHPFSASLWRKAV  1014
               TSARLNFLTPRYVNRKGVALPTSSE TKRAKRE LEQKQILVPELC+IHPFSASLWRKAV
Sbjct  950     TSARLNFLTPRYVNRKGVALPTSSEATKRAKREKLEQKQILVPELCAIHPFSASLWRKAV  1009

Query  1015    ALPCILYRINALLLADQIRTIVASHLNLGKTKLDDDFKWTPLNFGWSLSDVLKKSREDEM  1074
                LPCILYRINALLLADQIR  VA  LNLGK +LD +FKW PLNFGWSL+DVLKKS+++E
Sbjct  1010    CLPCILYRINALLLADQIRRTVALELNLGKIELDSEFKWPPLNFGWSLADVLKKSKDEEK  1069

Query  1075    RKQEEKLKSLREMAEEKLDELKIINLNLDYDTLKSDTNSLDEDDEEDSSTNSKWVVGTWS  1134
               +KQE+    + E+   ++ +++  + +                            +GTWS
Sbjct  1070    KKQEKIEPVIEEIPCTEIAKIEDFDQD--------------------DDEEEMIEIGTWS  1109

Query  1135    NEMAQSSQADSSALVRYVSPTSWLQ-GNTYDDDFSDDMSDNSDIDSEDSIHEWGKLRIEF  1193
               N+MAQ +      +VRY SPTSW+   NTY     D    +SD   ++S  EWG LRIEF
Sbjct  1110    NDMAQLNSDQEFPVVRYASPTSWMDLQNTY----DDSSFSDSDYSGDESESEWGGLRIEF  1165

Query  1194    TGDHQAEALDDGESKEDELFFVEDKNAWKVDDNSLETDRLRNEFTKACLRNKEHIWSSGI  1253
               TGD+ AEA+DD   K+D+   V+  N WKV+D S  T  LR +F  AC RNK+HI SSGI
Sbjct  1166    TGDNVAEAVDDENKKDDDFELVDYSNVWKVEDESEITQTLRKQFHDACARNKDHILSSGI  1225

Query  1254    LIKNGEVFEKKS--QNNLANDIFPVCPENFDFDSLMI-----------------------  1288
               L+   E F+K S   N    D       +FDF  L I
Sbjct  1226    LVSKSEQFQKCSDCDNTTTKDSQVANSYDFDFGKLFIELDQHKALQIDLAPQDARNEYDI  1285

Query  1289    -------------------GSINIEQTPA-----SPQEPFVNTYDISEERQNFSFDEQPD  1324
                                   S N+ Q  A     +PQ+   N YDISE    F FDEQP+
Sbjct  1286    SETMTFKFDEQPNLVEHPGPSPNLHQHKALQIDLAPQDA-RNEYDISET-MTFKFDEQPN  1343

Query  1325    LDTHPGPSPNVLLQALTMSNANDGINLERQETVGDSFLKYAITTYLYKTYENIHEGKLSH  1384
               L  HPGPSPNVLLQALTMSNANDGINLER ET+GDSFLKYAIT YLY  YEN+HEGKLSH
Sbjct  1344    LVEHPGPSPNVLLQALTMSNANDGINLERLETIGDSFLKYAITNYLYSKYENVHEGKLSH  1403

Query  1385    LRSKHVSNLNLYKLGKLKNLAEYMVATKFDPHDNWLPPCFYVPKQLEDALIDAQFPANCW  1444
               LRSK VSNLNLY+LG+ K L EYM+ATKFDPHDNWLPPCFYVPK+LE+ALIDAQFPANCW
Sbjct  1404    LRSKQVSNLNLYRLGRRKGLGEYMIATKFDPHDNWLPPCFYVPKELEEALIDAQFPANCW  1463

Query  1445    TVADMAATRNMTIDEICTLVRERG-IYSLPNIIPYNLVTQHSIPDKSIADCVEALIGAYL  1503
               TVADMAATR+MT+D+IC++VR+RG   SL NIIPYNLVTQHSIPDKSIADCVEALIGAYL
Sbjct  1464    TVADMAATRDMTLDDICSMVRQRGESLSLSNIIPYNLVTQHSIPDKSIADCVEALIGAYL  1523

Query  1504    IECGPRGALLFMAWLGIKVLPKDQDGNYGHLDFPKSPLLRNIPNPEEELEKLLDGYDAFE  1563
               IECGPRGALLFMAWLGI+VLP+ +DG YG ++ PKSPL  ++  P EEL+ LLDGYD FE
```

```
Sbjct  1524  IECGPRGALLFMAWLGIRVLPQLEDGTYGEIELPKSPLSNHLTYPREELDMLLDGYDQFE  1583

Query  1564  KHIGYKFRDRAYLLQAFTHASYSPNRLTDCYQRLEFLGDALLDFIITKALFEDIRMHSPG  1623
             +HIGYKFRDR+YLLQA THAS+SPN LTDCYQRLEFLGDA+LD++IT+ L+ED RMHSPG
Sbjct  1584  RHIGYKFRDRSYLLQALTHASFSPNTLTDCYQRLEFLGDAVLDYLITRHLYEDTRMHSPG  1643

Query  1624  ALTDLRSALVNNTIFASLAVKHGFHKYFKHLSPGLNEVIERFVRLQEESGHTIVDEHYLI  1683
             ALTDLRSALVNNTIFASLAV++GFH+YF++LSP LNEV+E+FVRLQE+SGHT+VDE YL+
Sbjct  1644  ALTDLRSALVNNTIFASLAVRNGFHRYFRNLSPSLNEVVEKFVRLQEDSGHTLVDELYLV  1703

Query  1684  DTECEEVEDIEVPKALGDVFESVAGAIYLDSGMSLDAVWKVYYRMMKAEIEQFSNKVPKS  1743
                   EEVED+EVPKALGDVFESVAGAI+LDSGMSLDAVWKVYY MMK+EIEQFSNKVPKS
Sbjct  1704  VET-EEVEDVEVPKALGDVFESVAGAIFLDSGMSLDAVWKVYYNMMKSEIEQFSNKVPKS  1762

Query  1744  PIRELLELEPETAKFGRPEKLADGRRVRVTVQVFGKGTFKGIGRNYRIAKCTAAKCALKH  1803
             PIRELLELEPETAKFG+PEKLADGRRVRVTV+VFGKG FKGIGRNYRIAKCTAAKCALK+
Sbjct  1763  PIRELLELEPETAKFGKPEKLADGRRVRVTVEVFGKGVFKGIGRNYRIAKCTAAKCALKN  1822

Query  1804  LKRRGLLR  1811
             LK+RGL++
Sbjct  1823  LKKRGLIK  1830
```

Graphical representation

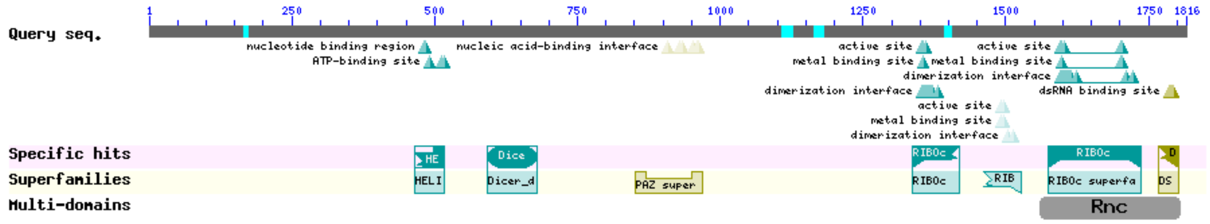

Ago-1

>Cb.comp42266_c0_seq6 len=3687

cDNA

GAAATTACCCGGAAATACGTTTAAAAACGCGCTTGGCAACATCGCTTTAACGTTTTTGTCCGCGGCTATGACGTCACTTTGGAATGTTTGCGTT
GCATCTGCAACGTGCACCAAACTGTTACGCATACAAGACCACTGCTTCATCCGCTAAGGACGCAGATTTTGTGTGCCACGCAAAGTGTCCTCGC
TTTAATACTCCGTAAAATTTCTGGGCCCTAAATCGGTAAAGAAAACGACCCGTAACTTTGGCAACGTCGGCGGAACAGCGGAGCACAACAATTG
CAAATGGCCGACGTGGGTTCATCGAGATCGTAGACGATTTATCGAATTGACTTAGTGGGTGGTGTATGTGTGCTTGTGTGACTAATTTATTTAT
TTAAACATGTACCCGGGACCTTCTGGACCAGGAACGTCGGGCACCAGCCCGATGGGTCCAACGACTACGACGGTGGCCTTGCCCGGCACCACGA
CCGCGACTAGTCTGACGACGGTGTCACCGCCAGCTGAACCTCCCGTGTTTCAGTGCCCGCGAAGGCCCAACTTGGGTCGGGAGGGTCGTCCCAT
CGGTCTCAAAGCGAACCATTTCCAGATATCGATGCCCCGTGGCTACGTCCACCACTACGACGTCAACATACAACCGGACAAGTGCCCGCGCAAA
GTGAACCGGGAGATAATCGAAACGATGGTTAAATCGTACGGGAAGATATTCGGTAACCTGAAGCCAGTGTTTGACGGACGGAACAACTTGTACA
CCCGTGACCCCTTGCCGATCGGTAACGCTAGGGAGGAGCTGGAGGTTACGCTGCCGGGTGAAGGCAAGGATCGGCTGTTCCGCGTCAGCATTAA
GTGGGTGGCGCAGGTGTCGCTGTACGGCTTGGAGGAGGCCCTCGAGGGTAGAACTCGGCAGATACCGTTCGAGGCGATCCTCGCGTTGGACGTG
GTGATGCGTCACCTGCCCTCGATGAGTTACACGCCGGTGGGGAGGAGCTTTTTCAGCAGCCCCGAGGGATACTACCACCCGTTGGGTGGCGGGC
GGGAGGTATGGTTCGGATTTCATCAGTCCGTGCGGCCCAGTCAATGGAAAATGATGCTCAACATCGACGTGTCCGCGACCGCATTTTACAAAGC
GCAGCCTGTGATAGAGTTTATGTGCGAGGTGCTCGACATCCGGGACATCAACGAGCAACGCAAACCGCTGACCGACAGTCAGCGCGTCAAGTTC
ACCAAGGAGATCAAGGGGCTGAAGATCGAGATCACGCATTGTGGGGCGATGCGCCGGAAATACCGGGTGTGCAACGTCACGCGACGACCCGCCC
AGATGCAATCGTTTCCGCTGCAGCTGGAAAATGGCCAGACGGTCGAGTGCACCGTCGCGAAGTATTTCCTCGACAAGTACAAGATGAAACTACG
TTATCCCCACCTGCCGTGCCTCCAGGTGGGACAGGAACACAAGCACACTTACCTACCGCTAGAGGTCTGCAATATCGTTGCCGGGCAACGATGT
ATCAAGAAACTGACCGACATGCAGACGTCGACGATGATTAAGGCGACGGCCAGGTCGGCTCCGGACAGGGAGCGCGAGATCAACAACCTGGTGC
GCAGGGCTGACTTCAACAACGACGAGTACGTGCAGGAATTCGGCCTGACGATTTCGAATAACATGATGGAGGTGCGGGGCAGGGTGCTGCCACC
GCCGAAGCTCCAATATGGCGGTCGGGTTGCGTCCCTCAGCGGACAGAACAAGCAGCAGGCGTGCCCGAACCAGGGCGTGTGGGACATGCGCGGC
AAGCAGTTCTTCACGGGCGTCGAGATTCGGGTATGGGCGATCGCGTGTTTCGCGCCGCAGAGGACGGTCCGCGAGGACGCACTGCGGAACTTCA
CGCAGCAGCTGCAAAAGATCTCGAACGACGCGGGCATGCCAATCATCGGACAGCCTTGCTTCTGCAAATACGCCACCGGGCCCGACCAAGTGGA
GCCTATGTTTCGATACCTGAAGACGACATTCCAGTCCTTGCAGTTAGTTGTTGTTGTGTTGCCCGGCAAAACGCCGGTTTACGCCGAAGTGAAG
CGCGTGGGCGACACGGTATTAGGAATGGCCACCCAATGTGTTCAAGCGAAGAATGTCAACAAGACGTCGCCGCAGACGTTGTCCAACCTGTGCC
TGAAGATAAACGTCAAGCTGGGAGGCATCAACAGTATTCTAGTGCCATCGATCAGACCAAAGATATTCAACGAGCCGGTGATATTCTTAGGCGC
GGACGTCACTCATCCGCCGGCCGGCGACAACAAAAAACCGTCGATAGCGGCGGTCGTGGGCTCGATGGACGCTCACCCGTCGCGTTACGCCGCC

ACCGTCCGCGTGCAGCAGCACCGTCAAGAGATTATACAAGAGTTGAGCTCGATGGTGCGCGAGTTGCTCATCATGTTCTACAAGTCGACCGGCG
GCTACAAGCCGCACCGCATCATTTTGTATCGCGACGGTGTGTCGGAGGGTCAGTTCTTGCAGCTGTTGCAGCACGAGTTGACCGCGATCCGCGA
GGCTTGCATCAAGCTCGAGGCGGACTACAAGCCGGGCATCACGTTTATAGTGGTGCAGAAGCGGCATCATACGCGACTGTTTTGCGCTGACAAG
AAGGAGCAGAGCGGGAAGAGCGGCAACATACCGGCGGGGACGACGGTCGACGTCGGTATCACCCATCCGACCGAGTTCGACTTTTACCTCTGTA
GCCATCAGGGTATTCAGGGTACATCCAGACCGTCCCACTACCACGTGCTGTGGGACGACTCGCACCTAGACTCGGACGAGCTGCAATGCTTAAC
CTATCAGCTATGCCACACCTACGTCCGGTGTACCCGGTCGGTGTCAATTCCCGCCCCCGCGTATTACGCGCACTTGGTAGCATTCAGGGCCAGG
TACCACCTCGTCGAAAAGGAACACGACAGCGGAGAGGGTTCCCACCAGTCTGGCTCGTCCGAAGACCGGACACCGGGAGCGATGGCGCGGGCGA
TCACCGTTCACGCAGACACCAAGAAGGTCATGTACTTCGCTTAGGGCGGGAACGTGCCGGTTTTTTTTTCCAATTTTCTCTTTCAATCCGGGCC
GACGAAGACGAACCGAGTCCGGTCACAGTTCTGATCGCGCGGCAGCGGAACACCGTCGAATGCTCTCGACGCTACGTGTTAGGTTAAGACGTTC
GGGGAATTCCCGTCCCCATCCTTCCACTTCACCACGCCATGCTTGATTAGTTGTTACGTCGTGATATTATCGGAGAAAAAAATACAAAAAAAAA
ATTGGCCCCGCGGTAATTGATAGGTCGGCTTAAAATCGGTTCCGTTTCGACTTTTTTAATCGGAAAACTGTTCTGCGTGTAGCATCGGCCTCGA
TTCGGAAATATGAGTTTGCGGAGAGTTTAAGAAAAACGCCATGTTATTTCTACAGGCACACCTACACCACCCATTAATTTACGGACGGACGATA
CTTCACAGTGTGTTGCCGGTAATTGTAGTAACGCGACTGCGCCTAGTTGCATCACTGTAAAAAATTTTAAATACCTTTGGAACCTGTGTTGAAA
CCGTCGCTTCTATCATTTAAGAGCGTAGCCTAACCTTTGATATGTTTACGTTAAAGAAGTGAAAAATGTTTTTTGGCCAATCAGTTGTGCTTGT
CGGATAACCTAAAAATCGATT

Protein RF2: 383 -> 3052 (889AA)

MYPGPSGPGTSGTSPMGPTTTTVALPGTTTATSLTTVSPPAEPPVFQCPRRPNLGREGRPIGLKANHFQISMPRGYVHHYDVNIQPDKCPRKVN
REIIETMVKSYGKIFGNLKPVFDGRNNLYTRDPLPIGNAREELEVTLPGEGKDRLFRVSIKWVAQVSLYGLEEALEGRTRQIPFEAILALDVVM
RHLPSMSYTPVGRSFFSSPEGYYHPLGGGREVWFGFHQSVRPSQWKMMLNIDVSATAFYKAQPVIEFMCEVLDIRDINEQRKPLTDSQRVKFTK
EIKGLKIEITHCGAMRRKYRVCNVTRRPAQMQSFPLQLENGQTVECTVAKYFLDKYKMKLRYPHLPCLQVGQEHKHTYLPLEVCNIVAGQRCIK
KLTDMQTSTMIKATARSAPDREREINNLVRRADFNNDEYVQEFGLTISNNMMEVRGRVLPPPKLQYGGRVASLSGQNKQQACPNQGVWDMRGKQ
FFTGVEIRVWAIACFAPQRTVREDALRNFTQQLQKISNDAGMPIIGQPCFCKYATGPDQVEPMFRYLKTTFQSLQLVVVVLPGKTPVYAEVKRV
GDTVLGMATQCVQAKNVNKTSPQTLSNLCLKINVKLGGINSILVPSIRPKIFNEPVIFLGADVTHPPAGDNKKPSIAAVVGSMDAHPSRYAATV
RVQQHRQEIIQELSSMVRELLIMFYKSTGGYKPHRIILYRDGVSEGQFLQLLQHELTAIREACIKLEADYKPGITFIVVQKRHHTRLFCADKKE
QSGKSGNIPAGTTVDVGITHPTEFDFYLCSHQGIQGTSRPSHYHVLWDDSHLDSDELQCLTYQLCHTYVRCTRSVSIPAPAYYAHLVAFRARYH
LVEKEHDSGEGSHQSGSSEDRTPGAMARAITVHADTKKVMYFA

Comparison with *Tribolium* Argonaute-1 (919AA)

```
Query  12   GTSPMGPTTTTVALPGTTTATSLTTVSPPAEPPVFQCPRRPNLGREGRPIGLKANHFQIS  71
             T+P G  +T VA+ G T+ T+L TV P  +PPVFQCPRRPNLGREGRPIGLKANHFQ++
Sbjct  40   ATAP-GTASTAVAVVGATS-TALATVPPTTDPPVFQCPRRPNLGREGRPIGLKANHFQVT  97

Query  72   MPRGYVHHYDVNIQPDKCPRKVNREIIETMVKSYGKIFGNLKPVFDGRNNLYTRDPLPIG  131
            MPRG+VHHYDV+IQPDKCPRKVNREIIETMV +YGKIFGNLKPVFDGRNNLYTRDPLPIG
Sbjct  98   MPRGFVHHYDVSIQPDKCPRKVNREIIETMVHAYGKIFGNLKPVFDGRNNLYTRDPLPIG  157

Query  132  NAREELEVTLPGEGKDRLFRVSIKWVAQVSLYGLEEALEGRTRQIPFEAILALDVVMRHL  191
            N+REELEVTLPGEGKDRLFRV+IKWVAQVSLYGLEEALEGRTRQIP+EAILALDVVMRHL
Sbjct  158  NSREELEVTLPGEGKDRLFRVTIKWVAQVSLYGLEEALEGRTRQIPYEAILALDVVMRHL  217

Query  192  PSMSYTPVGRSFFSSPEGYYHPLGGGREVWFGFHQSVRPSQWKMMLNIDVSATAFYKAQP  251
            PSMSYTPVGRSFFSSPEGYYHPLGGGREVWFGFHQSVRPSQWKMMLNIDVSATAFYKAQP
Sbjct  218  PSMSYTPVGRSFFSSPEGYYHPLGGGREVWFGFHQSVRPSQWKMMLNIDVSATAFYKAQP  277

Query  252  VIEFMCEVLDIRDINEQRKPLTDSQRVKFTKEIKGLKIEITHCGAMRRKYRVCNVTRRPA  311
            VIEFMCEVLDIRDINEQRKPLTDSQRVKFTKEIKGLKIEITHCG MRRKYRVCNVTRRPA
Sbjct  278  VIEFMCEVLDIRDINEQRKPLTDSQRVKFTKEIKGLKIEITHCGTMRRKYRVCNVTRRPA  337

Query  312  QMQSFPLQLENGQTVECTVAKYFLDKYKMKLRYPHLPCLQVGQEHKHTYLPLEVCNIVAG  371
            QMQSFPLQL+NGQTVECTVAKYFLDKYKMKLRYPHLPCLQVGQEHKHTYLPLEVCNIVAG
Sbjct  338  QMQSFPLQLDNGQTVECTVAKYFLDKYKMKLRYPHLPCLQVGQEHKHTYLPLEVCNIVAG  397

Query  372  QRCIKKLTDMQTSTMIKATARSAPDREREINNLVRRADFNNDEYVQEFGLTISNNMMEVR  431
            QRCIKKLTDMQTSTMIKATARSAPDREREINNLVRRADFNND YVQEFGLTISNNMMEVR
Sbjct  398  QRCIKKLTDMQTSTMIKATARSAPDREREINNLVRRADFNNDPYVQEFGLTISNNMMEVR  457

Query  432  GRVLPPPKLQYGGRVASLSGQ----NKQQACPNQGVWDMRGKQFFTGVEIRVWAIACFAP  487
            GRVLPPPKLQYGGRVASLSGQ    +KQQA PNQGVWDMRGKQFFTGVEIRVWAIACFAP
Sbjct  458  GRVLPPPKLQYGGRVASLSGQVGWHSKQQAMPNQGVWDMRGKQFFTGVEIRVWAIACFAP  517

Query  488  QRTVREDALRNFTQQLQKISNDAGMPIIGQPCFCKYATGPDQVEPMFRYLKTTFQSLQLV  547
            QRTVREDALRNFTQQLQKISNDAGMPIIGQPCFCKYATGPDQVEPMFRYLK+TFQSLQLV
Sbjct  518  QRTVREDALRNFTQQLQKISNDAGMPIIGQPCFCKYATGPDQVEPMFRYLKSTFQSLQLV  577
```

```
Query  548   VVVLPGKTPVYAEVKRVGDTVLGMATQCVQAKNVNKTSPQTLSNLCLKINVKLGGINSIL  607
             VVVLPGKTPVYAEVKRVGDTVLGMATQCVQAKNVNKTSPQTLSNLCLKINVKLGGINSIL
Sbjct  578   VVVLPGKTPVYAEVKRVGDTVLGMATQCVQAKNVNKTSPQTLSNLCLKINVKLGGINSIL  637

Query  608   VPSIRPKIFNEPVIFLGADVTHPPAGDNKKPSIAAVVGSMDAHPSRYAATVRVQQHRQEI  667
             VPSIRPKIFNEPVIFLGADVTHPPAGDNKKPSIAAVVGSMDAHPSRYAATVRVQQHRQEI
Sbjct  638   VPSIRPKIFNEPVIFLGADVTHPPAGDNKKPSIAAVVGSMDAHPSRYAATVRVQQHRQEI  697

Query  668   IQELSSMVRELLIMFYKSTGGYKPHRIILYRDGVSEGQFLQLLQHELTAIREACIKLEAD  727
             IQELSSMVRELLIMFYKSTGGYKPHRIILYRDGVSEGQFLQLLQHELTAIREACIKLE+D
Sbjct  698   IQELSSMVRELLIMFYKSTGGYKPHRIILYRDGVSEGQFLQLLQHELTAIREACIKLESD  757

Query  728   YKPGITFIVVQKRHHTRLFCADKKEQSGKSGNIPAGTTVDVGITHPTEFDFYLCSHQGIQ  787
             YKPGITFIVVQKRHHTRLFCADKKEQSGKSGNIPAGTTVDVGITHPTEFDFYLCSHQGIQ
Sbjct  758   YKPGITFIVVQKRHHTRLFCADKKEQSGKSGNIPAGTTVDVGITHPTEFDFYLCSHQGIQ  817

Query  788   GTSRPSHYHVLWDDSHLDSDELQCLTYQLCHTYVRCTRSVSIPAPAYYAHLVAFRARYHL  847
             GTSRPSHYHVLWDDSHLDSDELQCLTYQLCHTYVRCTRSVSIPAPAYYAHLVAFRARYHL
Sbjct  818   GTSRPSHYHVLWDDSHLDSDELQCLTYQLCHTYVRCTRSVSIPAPAYYAHLVAFRARYHL  877

Query  848   VEKEHDSGEGSHQSGSSEDRTPGAMARAITVHADTKKVMYFA  889
             VEKEHDSGEGSHQSGSSEDRTPGAMARAITVHADTKKVMYFA
Sbjct  878   VEKEHDSGEGSHQSGSSEDRTPGAMARAITVHADTKKVMYFA  919
```

Graphical representation

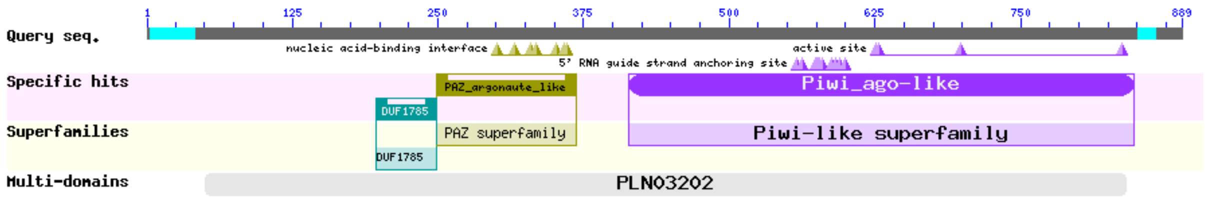

Loquacious

>Cb.comp43240_c1_seq4 len=3238

cDNA

GTTGCGAATGCGCCGTGTCGTCGGTTGAAGGACGCAGGCCTGGATTATACTACGAACGAACGATTTTTTGTTAGCGGTTTTTATTTTCCGTTTG
TAACAGAAGTTTGCGAAGTGCTCGGAGCAACCTATCCGCTCAACCGAATATGGATCCGAACATGGCCATAATGCATCCCACCGGTCCGGTAATG
CACCCAGTCGGTCTGATGCATCCGAGACGCGGGAAAAACCCGAAACACACGATGACCAGCACGATAACCCTGGCGGAAGAAGCGAAACTCGTTT
ATTCGCAAGAAATGTCCGGGATTAACAGCAAGACGCCCGTGTCGGTCCTGCAGGAGTTGTTGAGTCGCAGGGGCTCGACCCCCAAGTACGAGCT
GGTGCAGATCGAGGGGGCGATCCACGAGCCGGTGTTTAGGTACAGGGTGTTTTTGAGTAACGATCTGGTCGCAACGGGGACCGGTAGGTCGAAG
AAAGATGCGAAGCACGCGGCCGCCAAGAACCTTCTGGACTTAATAGTCGGCAAGCAGACCCCCGAACAGGCGAACCAAACCAATGGCACGCCAG
GTTCGACCGATATTACCGCCCAGGTGGTGTCTCCGTTCGACGACAAAGTGATGGGCAACCCGATCGGATGGCTGCAGGAAATGTGCATGTCGCG
CCGCTGGCCGCCCCCGCTCTACGAAATGGAACACGAGGAGGGCTTGCCTCACGAGCGGCAGTTCACGATCGCTTGTCAGGTTTTGAAGTACCGT
GAGGTGGGCACCGGCAAGTCGAAGAAACTGGCGAAGCGGGTGGCGGCCCACAGGATGTGGCAGGCGTTGCAGGACCTGCCGATGGAGGGCAACA
CCCCGCAGGCGTTTGAGAGCGACGAGGAGTGCCTGAACATGAAGGAGTTCAATTTCGTGCAGTTCCTGCAGGAGATCGCGTCCGAGCAAAACTT
CGAGGTGACGTTCGTCGACGTGGAGGAAAAATCGTTGACGGGACGCTGCCAGTGCTTGGTGCAGCTGTCGACGTTGCCGGTGGCCGTTTGCTTC
GGAACCGGCAAGACGCCGAAGGACGCGCGCTCTAACGCCGCCCTCAACGCCCTCGAGTACCTCAAGATCATGACGAAGAAGTAAGCGCTGCCGG
TTCGCAGGTAAAAAAAAAATAAAATAAATTTCCGCTCGTCCGCAATCGTGTTTCCTGTCCGAGAAAACCGCGGGCGGGGCGGCGGGCCACGGTC
GTCGCCGGGGGTCTTCCTCTCGGCCCCCCCTTCGATCGTTTCCAACGCTTTCTTCGTCTTCGGTGTGTCGACCGGGGGACGCTGGTGGACCGTT
TGTCGTGCTGGTTTTTCTTTTTATTTTTCATTATTTTTGAAGAAATATTTTTTGGTTGACTAATATATATTAGTATATACAGGGAGTTTGTCGA
ACATTTGTAAAAAATTCGTGGGGCGGGGACTTTTCATGAAAAAAAGTAAATATAAACGTTTGCGGGAAAATGCTTCCTAAGGGAGTTACGCCCC
TATAAGTGGGTCACCGTGAAGGCGGTTTATTCGCGATATTTCCGAGACCGTTCATACGGTAAAATTATCAGGGGCTTAAAGGGTTAAAATAACC
CTATTTTTTTAAGCGGATATTTTTCTTCACATTTTGGTTTTAAAATATGATACCTGAAAATTTTGTACATAAAAAAGGTTTTCTGAGCTCATAT
CGATCGAAGCCTTAGTTTTCGAGAAAATCGAATTTTTCTACATCAGTGCATCCGGACTCCAATTGAATTTTTCTAAACATTGGTAAAAAGTGTA
GCGATTGATAAAAACAAGTTCATTTAAACATATTGTTCGTAAAATGCTTCCTAAGGGAGCAACGTCCCTTCAAATCGAGTGCACCGTAGATGGG
AAAGTACATCTTCAGATTGCCCCATTTAAAAAAGGGCGTTGCTCCTTTAGGAAGCATTTTCCGATCTCATGAATCACCTCACGAATTTTTTACT
AATGTTTAGGACACACTCTGTATATATATGTAGTTTTTATTGTCCCGAACAGACTCGGTTTCTTTTACCTTCGAACTCGTCTGAATTTTTGCGT
GCAGATTTTTCAATTATTTATAACTGACGTGAGCGTCGAATCGGGACTATCTGTATTCATATTCAGTGCAATTTAGATGAAGATTTTTAAAGAA

```
ATGTTCTCTTGCTTCAATGTTACCAGGTCGGTCTCTGACGATTTGTTTACATCTTTGCGCTGTAATTCGACAATTCTGCCGGCGTAAAATTTCC
TGAATATTTTTAAATAAAATTTAGATATTATCCGGATTGGAAATTCGTGTAATTAAGGCGTGTCGCACCGCAAACGGTCCAAACGCGACATCCG
AGGAAAATATGTAAAAAGGAGAATGGCGAGTGTTTTTTACAAGCTATACTTTGCATAGAATTAGTGTTTTTTTTGTGGGTAATAATTAATAATA
ATAATCATATAATTTCTGATTTTAGAGTGTGGTTGTGTGACTTGTATTATACACACTAGAGTAAAGTTCTTCTTTCTTAGATAAAAAAAAAATG
TCCCTAGGCGTTTTAGCTACTTATATTATATGCGTACGGTATATATGCTGCCCCATGAAGTATACGCCGTATTCGTGTAAAGGGAGCATTTATT
TGATGCAAAGTATAGAGGTTGTATCCCGTTTTACAGTGTTTTTTTTTTACAATAACATTTATATAACGATAAATAATAATAATTAACATATATG
TCAATAGGTCGAGGATCGTCGCCTGACGATTCGAAATTCTAGAGTCTTAGTTATCTGTTCATTTATATTGATGTGTATGCTAATTACAGTATAT
TTGAATGGCGCCTTGCCGTTTTTTTTTTATTTTGCAGCACCCAAAATCTTAAGCCCAGACTATTTGACGTTAGCAAGTATCAAATTACACTTTG
ATAGTAAAAATGACAAAGCATAAAGAGCGAGGACATATTTTTTGGAGAATCAGTCATCTGACTGACTAATTATCCTTAGAAATTCTGAAAAATG
TAAAAGTAATGTTAAATATCGTTTATCTACTATTTACCTATTTACTGTTTTTTGACGTCACTGTTTTCGCGACCGTTTTAACGGGTCAATTTTA
TAGGCACGATAACCGCCACTAACCTTCGGAAATCAGGACCACAATTCTTGGAACTGGGGAGAACTGGGAACTGGGAGTGACGGTCTCCCGGGAA
TGTGTTCTGTCCTTATCGCTCTTACTTATTTTTTCATCAAGG
```

Protein RF3: 144 -> 1118 (324 AA)

```
MDPNMAIMHPTGPVMHPVGLMHPRRGKNPKHTMTSTITLAEEAKLVYSQEMSGINSKTPVSVLQELLSRRGSTPKYELVQIEGAIHEPVFRYRV
FLSNDLVATGTGRSKKDAKHAAAKNLLDLIVGKQTPEQANQTNGTPGSTDITAQVVSPFDDKVMGNPIGWLQEMCMSRRWPPPLYEMEHEEGLP
HERQFTIACQVLKYREVGTGKSKKLAKRVAAHRMWQALQDLPMEGNTPQAFESDEECLNMKEFNFVQFLQEIASEQNFEVTFVDVEEKSLTGRC
QCLVQLSTLPVAVCFGTGKTPKDARSNAALNALEYLKIMTKK
```

Comparison with *Tribolium* tar RNA binding protein (384AA)

```
Query  1    MDPNMAIMHPTGPVMHPVGLMHPRR--GKNPKHTMTST-ITLAEEAKLVYSQEMSGINSK  57
            MDPNM ++H +   + +     +HPRR    +N   H M +   ++L+EEAKL     EM+ + +K
Sbjct  13   MDPNMTLLHSSSQIHN----VHPRRKNNRNTLHGMQAERLSLSEEAKL----EMASLPTK  64

Query  58   TPVSVLQELLSRRGSTPKYELVQIEGAIHEPVFRYRVFLSNDLVATGTGRSKKDAKHAAA  117
            TPVSVLQELLSRRG+TPKYELVQIEGAIHEP+FRYRVF++NDLVATGTGRSKKDAKHAAA
Sbjct  65   TPVSVLQELLSRRGATPKYELVQIEGAIHEPIFRYRVFINNDLVATGTGRSKKDAKHAAA  124

Query  118  KNLLDLIVGKQTPEQANQTNGTPGSTDITAQVVSPFDDKVMGNPIGWLQEMCMSRRWPPP  177
            KNLLD++VGKQ+PEQAN +NGTPG+ DITAQVVSPFDDKVMGNPIGWLQEMCMSRRWPPP
Sbjct  125  KNLLDVLVGKQSPEQANASNGTPGANDITAQVVSPFDDKVMGNPIGWLQEMCMSRRWPPP  184

Query  178  LYEMEHEEGLPHERQFTIACQVLKYREVGTGKSKKLAKRVAAHRMWQALQDLPMEGNT-P  236
             YEMEHEEGLPHERQFTIACQVLK++EVGTGKSKKLAKR+AAH+MWQALQD+P+EGN   P
Sbjct  185  SYEMEHEEGLPHERQFTIACQVLKFKEVGTGKSKKLAKRMAAHKMWQALQDMPLEGNNLP  244

Query  237  QAFESDEE----------------------------------------------------  244
            Q ++ DEE
Sbjct  245  QGYDDDEELAAKMCNLQGRYSGLKDSKIPTLNIQHTQKVSQFHKALKQSNGPKLKELQNI  304

Query  245  CLNMKEFNFVQFLQEIASEQNFEVTFVDVEEKSLTGRCQCLVQLSTLPVAVCFGTGKTPK  304
             LN K+FNF+QFL EIASEQ FEVT+VD+EEK+L+G+ QCLVQLSTLPVAVC+G G TPK
Sbjct  305  VLNSKDFNFIQFLHEIASEQQFEVTYVDIEEKALSGKSQCLVQLSTLPVAVCYGAGATPK  364

Query  305  DARSNAALNALEYLKIMTKK  324
            +A+S AALNALEYL+IM+KK
Sbjct  365  EAQSAAALNALEYLRIMSKK  384
```

Graphical representation

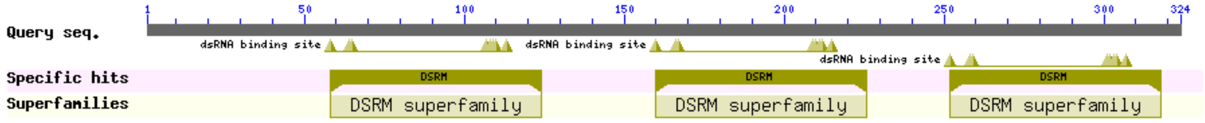

Drosha

>Cb.comp34198_c0_seq1 len=4497

cDNA

```
TTAGACTTCCAGTAGAAACACACCCACTTTCTATTGGGTTTTCTAGTCTATTGCCGTCAAGAATAGGGCCTCTGTCTAGTTAAAGAAGAGGTAA
AGGTTATAGCCACTTTTCTCTATCGTTGCAGGGGGTAATCTTGAGTAAAAACTAAAAGTGCTCAATTAATTAACCAATTTTATTGAATATGAGC
AAAAATTTATTAAGTTATATTATTAAATAATAAATGCATTCGTTATTAATTGATTGATTGTTAGCTGTAATTGAAGAAACCCTATTTGTTTTTA
TAGGAGAAGGAAGGAACAGCTTTATGTAATTTATTTCAAATGATTCCATAATTGAGCAGAACAGAACCGATAATTTAAAATCCTTAATCTTCCA
TTTCGCCATAAGTTGTGTCCTTTATTAAAATGGATGACCATTGGTATTATGGACAGCAATGCCCTGTTCCTAATTCCGGCCCTTCCCATATAAA
CTATGTGCATTATCCACCTCCACAGAGCCATGCAGAACATTTTATGCAGTGGCAACAAGCATCATCTCAGTTGCCAGTGCCTCCTTATCCTCCA
CCCGTAGTAGGTCCTTACACAATTCCTCCTCCAAATTTTCCTTCCTCCTCCAGCAGTTACGATTATTCATTTCAACAAAGTGTTCAGACATCTT
GTCAATATCAGTACTCATACCAAGGTTCACGCAATATGCAGAGAGGACATGATTATAAAAAGGAGCTGGATGACTACAGGGCTGTGAAAAAGGC
TAGCATAAGAGATAGTCCTTCAAGTTACAATCACAAACGGTCTGGTGAGTCAAGTAGCAGCAGGAGTGCTTCATATTCCAAAAGAAGTAGAAGC
CGGAGCAGAAGTCTGAGTAAAAGTCGTAGTAGGGCTCATAGTAGAGACCGATGTAAGTACATAAATAAAAACAAAGAAAGGGAATCTTCAAAAG
AAAAATACAAAGGTAGAGATACTGCTAGGCAAGTGCAAAATGAAAGAGATGAAATACTAAACAGATATAAGCGCAACTATTGCCATACCGAAAA
GCAAATATCTCAAAAACTCGAAGAAATTAGCCGTAAACATATCGACTTTTTGGATCAAGAAAAGAACTTTTGGATTAGATCAACACCATCAGAA
CTATTTTATCAGAAAGATGAAAACAACTTGAAAATCACCAAGGCCACTGCAAAACTGATCAAGTTGTGTCTAGAGTTTGATGAACTTCTGGTCT
CAAGGGCTCTTAAAGTGAACTCTTTGAAGAGTAAATATATTCCTCCTCCTAGAAAGAACAGATCAAGGGTGTGCAAGCATAGGACTGAAGTGTC
AACCACTTCTGATTCAGAGAGTTCAGAGGATAATTTGACAGATGAGGAAAATTTTTCAATGGAGGAATTACAAAGAAAACAACAGCATCCAGAC
AGGTTGCATCCAGAGATGTGGTATAACGACCCAGGAGAAATGAATGATGGTCCGTTGTGTCGGTGCTCCTTTAAGTCGAAGAAATCAGGTATTA
GGCACGGAATTTATCCCGGCGAAAAACAAATTTCAAAATGCAACCCATATAGTAACAATGCCGATAGACTTTACCATTATAGAATCATAATTTC
GCCCCCGACAAATTTCCTAATCAAGGCTCCTACTATAATACAGCATGATGAGCACGAGTTTATATTTGAAGGATTTTCGATATTCTCTCACTCA
CCACTTGTCCAATTGCCTCACTGCAAAGTAATCAGATTCAATATCGAGTACACCATCATCTACTTGGAAGAGAAAATCCCAGATAACTTTACCG
TTTGCGAATTGGATATGTTCACAGAGTATCTATTCCGTGAAGTATTAGAGCTGATCGATTTAGATTTAACGACTCCCAAAGATAGCAAGCGCTG
CTCTCAGTTCCACTACATGCCCAGATTCGTTAGAGAACTCAACGAAAACGGCAAGGAAATCCTCTGCATGGAAGTGGTTCTGCAATATTTATTA
AACTGTTCAGTGCCGCTGATACAAAAAAGTGATCTAAAAACTATGGTCAAGATGTCTCAGTATGAGTGGCAACACTTTGCTGACGAAATCAAAG
GTATGGTAGTCACTTATCCAGGCAAGAAGCCCAGTTCAGTACGTGTAGATCAACTGGATAGAAATATTGACCTTCAAAAGGAGGGAGATTATAA
GTTTCCAGAAATTGTTCATTTCGGCATTCGACCTCCTCAGTTAAGCTATGCTGGCAATCCAGAATATCAAAAAGCGTGGAGGGAGTATGTAAAG
TATCGCCATCTTATAGCAAATATGTCCAAGCCAACTTTTGAAGATAAACGGAAACTAGAAGCTAAAGAAGGCAGGCTTCAAGAGATTCGCACTC
AGGGAAAGATGAAACGCGATGTCACTATAGCGGTATCAGCAGAGGGCTTTTACCGCACTGGAATTATGTGTGATATTGTTCAGCATGCCATGCT
CATTCCAGTTCTAATTTGCCATTTGAGGTTTCATAATGCTCTCAATGTATTAGAAGAGTCCATAGAATACAAATTTAAAAATCGAGGACTGTTG
CAAATAGCTTTAACCCATCCCTCCTACAGACAAAATTTCGGTACCAATCCGGATCACGCAAGAAACAGCTTGACAAATTGTGGCATTAGGCAGC
CAGAGTATGGCGACAGGAGAATTCATTATATGAATACTAGAAAAAGAGGTATAAACACACTTATAAACATTATGTCAAGATTCGGCCGTCAGCA
AGAGACTGAATCTAACATAACTCATAACGAACGCCTTGAATTCTTGGGAGATGCAGTTGTCGAATTTTTGTCTTCCATTCACTTATTCTTTTCA
TTTCCTGATCTTGAAGAAGGTGGATTAGCTACATATAGGGCCGCTATTGTTCAAAATCAGCATTTAGCTCTATTGGCTAAAATCCTAAACTTGG
ACCAATTTATGCTTTACGCTCATGGTTCTGATTTGTGTCACGATCTCGAGTTAAAACACGCAATGGCCAATTGCTTTGAAGCCCTTATGGGTGC
CCTATTTTTGGACGGCGGCATCGACGTAGCAGATAAAGTGTTCTCCAACACTCTATACAAAGACGAGCCAGAACTACTAAAAGTGTGGAAAGGC
TTACCGCTACACCCGTTGCAAGAGCAGGAGCCTCACGGCGACCGACATTGGATTGAGAAATTCGAAATTCTCCAAAAATTGACCGGTTTTGAGA
AAGCTATTAACGTTGAGTTTAATCATATCCGTTTGTTAGCCAGAGCTTTTACCGACAGAAGTGTGGGTTACACGAACCTTACTATGGGCTCGAA
TCAAAGGTTAGAATTTTTAGGAGACACTGTGCTTCAATTGATCGCTTCTGAGTACTTGTATAAGTACTTTCCCGAACACCATGAAGGTCATTTG
TCACTGTTGAGGAGTTCACTGGTTAACAACCGAACGCAGGCCGTTGTCTGTGACGACTTAGGTATGGCCCAGTATGCCATCTATAACAACCCAA
AGCCAGAGTTAAAAACAAAAGACCGGGCTGACCTTCTGGAAGCTTTTATTGGTGCTCTTTATGTGGACAAAGGAATGGAGTACTGCGAGGTGTT
TTGCCAAGTGACTTTGTTCCCGAGATTGCAAGACTTTATAATGAACCAAGACTGGAACGATCCCAAATCTAAATTACAGCAGTGTTGTCTGACT
CTTAGGACAATGGATGGAGGCGAGCCGGATATTCCAGTTTATAAGGTTATAGAATGCAAAGGTCCCACCAACACTCGAGTCTACACGGTTGCTG
TATATTTCAGAGGTAGGAGATTGGCTAGCGCAATGGGTCACAGTATCCAGCAAGCAGAAATGAACGCGGCAAAGAAGGCTTTGGAAATATCTCA
CCATCTGTTCCCCCAACTTGACCACCAAAAACGGGTGATCGCAAAAAGTATGAAAAAAACAGAAAAAGAGCGGACGATCGAAATCCCGAAGCTT
TGAAAGCCTAAGAAGGAAATCTCCCGAGAGTTACTCAAGGAATCGGTTTAAAAGTCGTTCCAGATCTCGAAGTACGTCGAGATCTAAACGAAGT
ACAAGTCGCGGCGATTTAGATTCGAGTCGACAGGCGTCCAGGCAAGGGAAGAAAACATCAAGATCGAGAAGTAGTAGCAGCGGAAGCAGCAGCA
ACGACAGCGTTAAAGGAAGCAAAAAATGAGCAGTTTGCAGTAAGCTGCGAAGTTTATGTTCAGTAAAATAGGCATGTAGTGAAAAAATAATCTC
TTAATCTTTATTAAGTCTGAACTGCAGTAACTTATTACAAGTAACAAGGGAAAGTCAAGGTGCCAGTTAGTAAGGTAAAAAAATCCAAGCAAGC
ATATCTGCTGACCGTCCGTACCATAACATTTGATTGATACTAATCCTCACGTTGCCGATTTCTCACAATATTTTGCCAGTGCTAGTTTTTATAA
TAGTACAGTATACAGATTATGACACCAGTAAACATGACTAGTATACCGGCAGTCGCATATACAGGGTGTCCGGAAATGA
```

Protein RF 1: 406 -> 3951 (1181 AA)

```
MDDHWYYGQQCPVPNSGPSHINYVHYPPPQSHAEHFMQWQQASSQLPVPPYPPPVVGPYTIPPPNFPSSSSSYDYSFQQSVQTSCQYQYSYQGS
RNMQRGHDYKKELDDYRAVKKASIRDSPSSYNHKRSGESSSSRSASYSKRSRSRSRSLSKSRSRAHSRDRCKYINKNKERESSKEKYKGRDTAR
QVQNERDEILNRYKRNYCHTEKQISQKLEEISRKHIDFLDQEKNFWIRSTPSELFYQKDENNLKITKATAKLIKLCLEFDELLVSRALKVNSLK
SKYIPPPRKNRSRVCKHRTEVSTTSDSESSEDNLTDEENFSMEELQRKQQHPDRLHPEMWYNDPGEMNDGPLCRCSFKSKKSGIRHGIYPGEKQ
ISKCNPYSNNADRLYHYRIIISPPTNFLIKAPTIIQHDEHEFIFEGFSIFSHSPLVQLPHCKVIRFNIEYTIIYLEEKIPDNFTVCELDMFTEY
LFREVLELIDLDLTTPKDSKRCSQFHYMPRFVRELNENGKEILCMEVVLQYLLNCSVPLIQKSDLKTMVKMSQYEWQHFADEIKGMVVTYPGKK
PSSVRVDQLDRNIDLQKEGDYKFPEIVHFGIRPPQLSYAGNPEYQKAWREYVKYRHLIANMSKPTFEDKRKLEAKEGRLQEIRTQGKMKRDVTI
AVSAEGFYRTGIMCDIVQHAMLIPVLICHLRFHNALNVLEESIEYKFKNRGLLQIALTHPSYRQNFGTNPDHARNSLTNCGIRQPEYGDRRIHY
MNTRKRGINTLINIMSRFGRQQETESNITHNERLEFLGDAVVEFLSSIHLFFSFPDLEEGGLATYRAAIVQNQHLALLAKILNLDQFMLYAHGS
DLCHDLELKHAMANCFEALMGALFLDGGIDVADKVFSNTLYKDEPELLKVWKGLPLHPLQEQEPHGDRHWIEKFEILQKLTGFEKAINVEFNHI
RLLARAFTDRSVGYTNLTMGSNQRLEFLGDTVLQLIASEYLYKYFPEHHEGHLSLLRSSLVNNRTQAVVCDDLGMAQYAIYNNPKPELKTKDRA
```

DLLEAFIGALYVDKGMEYCEVFCQVTLFPRLQDFIMNQDWNDPKSKLQQCCLTLRTMDGGEPDIPVYKVIECKGPTNTRVYTVAVYFRGRRLAS
AMGHSIQQAEMNAAKKALEISHHLFPQLDHQKRVIAKSMKKTEKERTIEIPKL

Comparison with *Tribolium* ribonucleae III (1180AA)

```
Query  193   ERDEILNRYKRNYCHTEKQISQKLEEISR-KHIDFLDQEKNFWIRSTPSELFYQKDENNL  251
             ERD IL+++++NYC T +++S K+ E+++  H + L+QEKN W RSTPS+L+Y+KDE+N
Sbjct  136   ERDLILSKWRKNYCSTREEVSNKIHELAKVDHEEVLEQEKNIWTRSTPSDLYYRKDESNA  195

Query  252   KITKATAKLIKLCLEFDELLVSRALKVNSLKSKYIPPPRKNRSRVCKHRTEVSTTSDSES  311
             ++T+AT +L +LC +F++ LV RA KVN LK KY PPPRKNR+R+CKH++E S++S S
Sbjct  196   RVTRATKRLTQLCDKFNDCLVMRAAKVNKLKPKYEPPPRKNRARLCKHKSEESSSSGSSE  255

Query  312   SEDNLTDEENFSMEELQRKQQHPDRLHPEMWYNDPGEMNDGPLCRCSFKSKKSGIRHGIY  371
              E  LTDEE+ +MEELQRKQQHPDRLHPEMWYNDPGEMNDGPLCRCS KS+KSGIRHGIY
Sbjct  256   EE--LTDEEDCTMEELQRKQQHPDRLHPEMWYNDPGEMNDGPLCRCSIKSRKSGIRHGIY  313

Query  372   PGEKQISKCNPYSNNADRLYHYRIIISPPTNFLIKAPTIIQHDEHEFIFEGFSIFSHSPL  431
             PGEK + KC P SNNA+RLYHYRI ISPPTNFLIK PTII +DEHEFIFEGFS+FSH PL
Sbjct  314   PGEKHLEKCVPDSNNAERLYHYRITISPPTNFLIKTPTIIHYDEHEFIFEGFSMFSHFPL  373

Query  432   VQLPHCKVIRFNIEYTIIYLEEKIPDNFTVCELDMFTEYLFREVLELIDLDLTTPKDSKR  491
              +LP CKVIRFNIEYTI+Y+EEKIPDNFTV ELD+F +YLFRE+LEL+DLD     D
Sbjct  374   EKLPTCKVIRFNIEYTILYIEEKIPDNFTVRELDLFHDYLFREILELVDLDFKAAGDVDG  433

Query  492   CSQFHYMPRFVRELNENGKEILCMEVVLQYLLNCSVPLIQKSDLKTMVKMSQYEWQHFAD  551
             CSQFH+MPRFVREL +NGKEIL M  VLQYLL+ SV LI++ DL+ M+KM+QYEWQ +AD
Sbjct  434   CSQFHFMPRFVRELPDNGKEILAMNEVLQYLLDSSVSLIEEKDLEDMIKMTQYEWQSYAD  493

Query  552   EIKGMVVTYPGKKPSSVRVDQLDRNIDLQKEGDYKFPEIVHFGIRPPQLSYAGNPEYQKA  611
             EIKGMVVTYPGKKP SVRVDQLDRNIDLQK GDYKFPEIVHFGIRPPQLSYAGNP+YQKA
Sbjct  494   EIKGMVVTYPGKKPCSVRVDQLDRNIDLQKPGDYKFPEIVHFGIRPPQLSYAGNPDYQKA  553

Query  612   WREYVKYRHLIANMSKPTFEDKRKLEAKEGRLQEIRTQGKMKRDVTIAVSAEGFYRTGIM  671
             WR+YVK+RHL+ANMSKPTFEDKRKLE+KE +LQE+RTQGKMKRD+T+AVSAEGFYRTGIM
Sbjct  554   WRDYVKFRHLLANMSKPTFEDKRKLESKENKLQEMRTQGKMKRDITVAVSAEGFYRTGIM  613

Query  672   CDIVQHAMLIPVLICHLRFHNALNVLEESIEYKFKNRGLLQIALTHPSYRQNFGTNPDHA  731
             CDI+QHAMLIPVL+CHLRFH++LN+LEES+ YKFKNR LLQ+ALTHPSYR+NFGTNPDHA
Sbjct  614   CDIIQHAMLIPVLVCHLRFHHSLNILEESVNYKFKNRALLQLALTHPSYRENFGTNPDHA  673

Query  732   RNSLTNCGIRQPEYGDRRIHYMNTRKRGINTLINIMSRFGRQQETESNITHNERLEFLGD  791
             RNSLTNCGIRQPEYGDRRIHYMNTRKRGINTLINIMSRFG+QQETESNITHNERLEFLGD
Sbjct  674   RNSLTNCGIRQPEYGDRRIHYMNTRKRGINTLINIMSRFGKQQETESNITHNERLEFLGD  733

Query  792   AVVEFLSSIHLFFSFPDLEEGGLATYRAAIVQNQHLALLAKILNLDQFMLYAHGSDLCHD  851
             AVVEFLSSIHLF++FPDLEEGGLATYRAAIVQNQHLA+LAK L LDQFMLYAHGSDLCHD
Sbjct  734   AVVEFLSSIHLFYTFPDLEEGGLATYRAAIVQNQHLAVLAKTLKLDQFMLYAHGSDLCHD  793

Query  852   LELKHAMANCFEALMGALFLDGGIDVADKVFSNTLYKDEPELLKVWKGLPLHPLQEQEPH  911
             LEL+HAMANCFEALMGALFLDGGI+V D+VFS TL+K  P+LL+VW  LP HPLQEQEP
Sbjct  794   LELRHAMANCFEALMGALFLDGGINVVDRVFSETLFKVNPDLLEVWMNLPPHPLQEQEPT  853

Query  912   GDRHWIEKFEILQKLTGFEKAINVEFNHIRLLARAFTDRSVGYTNLTMGSNQRLEFLGDT  971
             GDR WI KFE+LQ LT FE+++ ++FNHIRLLARAFTDRSVGYTNLT+GSNQRLEFLGDT
Sbjct  854   GDREWIPKFELLQNLTKFEESVGLQFNHIRLLARAFTDRSVGYTNLTLGSNQRLEFLGDT  913

Query  972   VLQLIASEYLYKYFPEHHEGHLSLLRSSLVNNRTQAVVCDDLGMAQYAIYNNPKPELKTK  1031
             VLQLIASEYLYKYFPEHHEGHLSLLRSSLVNNRTQAVVCDDLGM+ YA+YNNPK ELKTK
Sbjct  914   VLQLIASEYLYKYFPEHHEGHLSLLRSSLVNNRTQAVVCDDLGMSNYAVYNNPKAELKTK  973

Query  1032  DRADLLEAFIGALYVDKGMEYCEVFCQVTLFPRLQDFIMNQDWNDPKSKLQQCCLTLRTM  1091
             DRADLLEAFIGALYVD+G+E+CEVFCQVTLFPRLQDFIMNQDWNDPKSKLQQCCLTLRTM
Sbjct  974   DRADLLEAFIGALYVDQGLEFCEVFCQVTLFPRLQDFIMNQDWNDPKSKLQQCCLTLRTM  1033

Query  1092  DGGEPDIPVYKVIEC--KGPTNTRVYTVAVYFRGRRLASAMGHSIQQAEMNAAKKALEIS  1149
             DGGEPDIPVYK + C      NTRVYTVAVYFRGRRLASAMGHSIQQAEMNAAKKALEIS
Sbjct  1034  DGGEPDIPVYKSVVCFTNSEVNTRVYTVAVYFRGRRLASAMGHSIQQAEMNAAKKALEIS  1093

Query  1150  HHLFPQLDHQKRVIA  1164
               LFPQLDHQKRVIA
Sbjct  1094  QDLFPQLDHQKRVIA  1108
```

Graphical representation

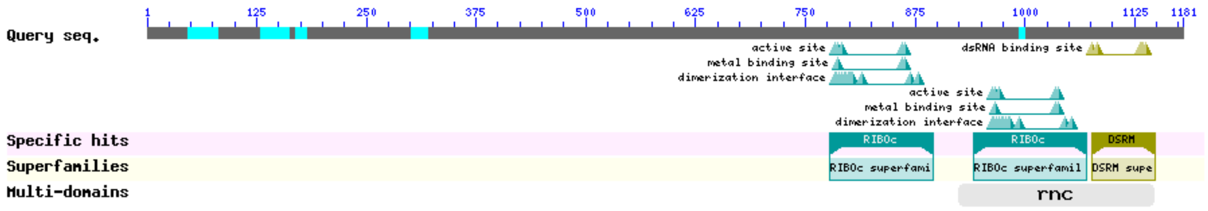

Pasha

>Cb.comp41893_c0_seq1 len=6734

cDNA

```
TTTATTTATTTATTTATTTATTGACAAAAAAACGCAGAGGTTAGGAATTAATTTTTTTAATAATTACACACCAGTTACCTTGATCATAATAACT
TTTTTCAATAACTGTGTTAGATAACTTTTGACACAAATTATCCAATTCTTGTTCATCAAATACATGGTAGTAGCGAAAATGAGTATTGTCGTCG
TCATTCTTTAATTTCCAAGGCACCAGGACATCTTTTGTCTGAAACTGTGTTCTGTTGGTATGAACTGGAAACGATATATTCAAAACTGAAACTT
CTTTGGATGGAACTGATTTATGATCTACACAATCATGTTTTCTGTTCTTTCTGTCTTGTTTGATGTAACTTGTTTTCTCCTGATCCTTTAATTG
GTCCTTGGCCCACACATAAATTAAAGCTTTTCCTCCAATTTTCAATATTCTTACAATTTCTGACACTGCAAGTAATCTTCTAGCCTCATTTGCC
AAATGATGGATAACTGCAATACAAATAATCCCATCTGCAATTTCACTCCTTAATGGAATATTTAAACAGTTGGCAACAAATATTTCAAATCCCC
TTTCACTACATATTTTAGCCAATTCTAAGCTGCTATCACAACCTATATCAAATATGTTTACATTTTTTGCCAAATATTTTCCATTTCCACAGCC
AACATCTATAAGTATTGATCCTAACTCAAATGATTCTACAAATTTTAATACATTGGGCCAAGGTTTATGTCTGGTATCGCTAAAATGATTGGCA
ATAGAGTTGTATACTCCAAGAACATGCAAATTTTCCAACTTGGAGGCTGCAACATTATTCATTTCTAGAAACTTTTTGTACGAGTCACACTTAT
CATTGTAAATGCAATGACATTCAGTATTTGATACTTTCCTAAAAGTAAATGATATTCGAATGCCTCGCTTAAATGATGTATATCCTGCTGCAGT
TGGAATTATGTCATATTTTCTTGGTATTATGCCATGAGTCCAGTTATAACGACTCTCGCCAGACATTACCAGAAGGGATCTTTGGGGTAAAAAA
ACACAAACATGTCTGTTATCACTTCTGAACTCCATTACAATACCAGATTTTAAAGATAGACATATAATGGGATCATCAAATGCACTGTGAGTAT
CTACATGATGGGGTATTCCCTGACCAGGATTATAGTGATTAATCGTCAACTGATCTGGCATGAAATTTTTGCATTCTAAACCTATACTCAATAA
TCTTTGCCACAAGAAACTACATTCCATTGGAATTTTAGCATCAAGTGGTTTTTCCTTGTCAACATTATTAATATCATATCTAAATTCATAACCA
AAATGCTTTACATGTCTATGCCTCATATTTCCCAACTGTGCCTCTTCAAAATTGCACAATTTAAGTAACTCCTCTTCTTCCTCCATTGTAATAA
AATCTTTTAAAATTGTTAAACCTGGAGGATACCACATTGTTTCCCATATTTTGCTTTCTTTGAAATTGGGAAATGATTCAGTAAAAAGAAGATA
AATGGGTTTATCATATTGCGCTATGTTTAATTTTCCATTGTAGGCATTGTAGGCATTTGAAGAAGCTTCACAGTTATCATAGGATAAAAAACTG
CAAGATTTTCCTGATAAAAGACAAATTTTGGTGAGGTTGCCAAATTTAATAAAATGTTGAAATACAACTTCTTCCGTTAATCCATTGACCAAAC
CTGCATTGCATATTACTAAATTCTTGCTCGGAATTGCGGTGCATATAATTCCAGTTTCTTTCTTAATTATGTGCTGAAATTTCTTTAATTTCCT
ATCAATTTTTTTAAATTTGGAATTGGATATATTTTCTAAAGTCTGCATTTTGATCAATCAATTCTTAATCCACAAACATAGAAAAAAATAAGTT
GAGATAATTATTTTTGTCCTTCAAATACAAATCATAATCAGTTGATCAAGTCACAAACACACACAGAAAATTGTAAGTACGGTGCGGGGTTAGG
TAATCCATGATGAAACGACATTAGGTAACATTGATTATTAAAGGATATTTTAGTCGGTGATAGTTAAATGAAAGTTATCGCGACAATATTTGGA
CAAAGGAAAACAAATTACTAACAAAAGATAATTTCTTCCAATATGGAGGGTTTAAACTTGAAACAGGTATATGGTTTATGGTGAGGAAGTCTAT
ATTTTTATTCTGTGTGGTACCATCGAAATCTTTTTCATACATTTTCTGAGTCTTTCCGAAACCGATACCCGACTTTGGGTGTTGTGTTAGCTGG
TTTGTTGAGTGATAATAAAATTTTGGAGGAAATAGTTTTAGGCAGGAGTGTCTTTGAATTTAATAGGGTGACAAGAAACAATCCAAAAACATTA
TTTACAACATCTGAATCATTATAAAACTTCTGTAGTTATTTGTCTCTATCATTTCAAGGTTATCTCTCTTAGACAAAGAGGAATGACCTGGAAC
ATGAGCTATGTAGCTTGTTTTCCTCGGTTCCCCTCTAAAGAATATTTCAATTTTTCATTTAATATTGTTGAAGTATTCTTTTGATTGTTACCGT
GTGAAAACTTAATCAACGACAATGAAGAGCAATGAGTAAGTAATGAGGAAGAATCTCATTATGGAAGGCATTAGTGGGTCAGTAGTGGAAACCA
TTGAAGTAGCTATCAAAACCTGTACTGGGAATCGAACTAATACTGTTTGCAATCATGTTGAAGAGAACAATTTTGAAACAGAGGCATGCAACCA
AGCAGAAAACTACAGCACACTCAATGGTCACAATCCTTTAATTAGCAAAGTAATTCAAGCCAGTAAAAACTCGCAACTGTTATCTAAAAAAGAA
CTTAACAGTATGGAAGGGGAAGATTATATAGTGAATATGAGTAAACAGTGCCCATTTAAAAATGGAAAATTCAATAAAGTTTTGGAATCTAATT
CCACACTAAGTGAGGATACTTCGAAAGCAAATGATTGTTCTGAAATAAGTAATAAGAAAGAAAGTGTGCACGAAATGTACATAGGGGAATCCAA
GGAAGTAAACACAACTCAAAATAATCTTATAAATGGGACTCCACATCAATCCAATTTTGAACCAATTGGTTGTCAGACTGGGAATGCACTGCAC
CTCCCTTTGAATTTATCTAGAGGATATGATGGCTTAAAAGAAAACAACATAAGCCAAAGCAATGAAAATCAATATTACAGCTATGAATCTGATG
AGTCAATTGAGTATGACTCTGATATACCTGATGAGGAAGTTGAGAAAATGCTAGAAGAAGCACTTATTACAAAAAAAAAGAAAAGCAGGGCAAG
CTGGATTGGATGCAGATAACACTAAAGTACCTTTTGAGGAAAAAACTAAAATAGTGTTGGTGGAAAAAGGAAGAAATCATTTTGATGTTCTTCC
AGAAGGATGGATACAAGTGACTCACAATAGTGGGATGCCAGTATATTTGCACAAAACTACAAGGGTGTGTACTTTGTCAAGACCTTATTTTTTG
GGTCCTGGCTCTGCCAGAAAGCATCTAATACCCGTGAATGCAATCCCTTGTTTGAATTATAAAAAAGCTTTAGAAGATGAAAAGAAGGAGCAAC
AAGAAGTCGAGGAGAAAAGGACAGACGAAACGAAATTGCCCGAGAATTTACCGAACGCTAGGATAGAAACTGTCCAGGAGAATATAGAAACTCA
AAATTTAACTGCGGAAGCTTTAAGGAATTATTGCGAGAATGTGTTTCAGTTCCAATCCATTAATGTTTTGCGGTTTAAATCTTGGTCCGAGAGG
CGAAAATTTACCAAGAAAAGGAAACACGAAAGTCAGCTTCAAAGGCCAACATTGCCTGATGGTACAAAGCTTATAACATTTCCGATAAAAGATA
TTGACAGTAGTGAAAAAACTAACCCAAACGCAAAAAAAGAATGGATTATGAATCCAAATGGGAAATCATATGTGTGCATATTGCACGAGTATGT
CCAACATGCCCTAAAGAAACAGCCCACTTACCAATTTACAGAACTGGAAAATGCCGCGACTCCTTATGCGGCAACTGTTATCATAATGGATATG
```

AGGTATGGTGTCGGATATGGTACTAGTAAAAAGCAAGCGAAATCAGAAGCCGCCCGCGCTACTTTAGAAATCTTGATCCCAGAAATGAAATCAA
AAATAACTACAGATAATCAGACAGGAAGAACAAGAAAAGAAAAAGAGCAAGATCTATCGTTCTTTGACGAAATCAGGGTCGAAGATCCGCGCAT
CGCGGAATTCTGCGCCAAAACCACCGAACCTGCGCCGTACGACATTCTCTTGACTTGTTTGCAACGGAACTTCGGGTTAGACGATCTGAAGATT
CACTATCAAGGCAATACGTCAAAACACCAAGGCAACGAGTTTACAATGACTGTCGGAAAATATACGACGACAGTGGCCTGCAAAAACAAACGGG
ATGGCAAACAAAGGGCCTCGCAGGCGATTTTACAGGCCTTACATCCGAGTATAACTTCATGGGGTTCGCTTTTGCGGTTATACGGAAATCACTC
TGTAAAGAGTTTTAAAGAGAAGAAAATGGAGGAACAAGAAATTACTTCTTTACAAAGCAAGGCGGCAGTGAATCAGCCCAATTTTGCCATCCTC
AACAAACTGAAAATGGAATTGGGTAAACTGGACGAGAAGAGGAAAACGGTAAGGCCAGTAGGGTTAGTAAACACTTCGGAGGGTGAAATTATTT
TAAAGTCGTCCAACGATAAGAAAAATATATAAAGAGTGTTATATTATTATTATTTGCCATAAAATTGAATTAGGTATTTTAAGCTATCCCGCAG
TAAAATTAAAATTGTCTTTGTGTGTACCAGACTGAGTAGCATTGTAGCAAATAAATTAGCAATTGACCAAATGAGCTATTATTCTAGGGAAGTA
GATTAATTATGCTTTTTTTAAATGTGTTAAATTATGTGCAAATATTTTTAGAACGGCTTCTTGCATCAAGATACCAAGAACTGTGTTTGTGACA
TATCGTTACAGCTAATGGCAGTCCTAGCAGCTTTTCTTTAAAAAATTCATTTTTGTAATTTTGCTACTCTTTTCGAAATAACATTTATTTTATC
TAATTGTTCTCATTGATGAAATTGCGATGTCATGAGTTGATTGACAGTTGACCTTATGACATCCATATTCAGTTCTCAATGCGGATATTGTATG
ATTGCGTCTCTTGCTGTTTGATTTTCAGGGCGTGAAAAACACAAATACAAAGAATATTGTTCTTTTTAATCACCAGAGGCAAAGTTATATAATT
TAAGGGAGTTTAATTGTAAAAATGTTTTATTATCGCAATATTTGCGCGGAATTACAAGTTGTCAAAATGAATGGTTCAGTTTCAAATTGGCTGT
CCATATGTTTTTTACAAAACAGTTTATCGTGTTTTTAATCTTTCAACTTTAATATGGGTTAGAAGTGGCGGCAAATTTTTATATAATATTATTA
ATTATCTGCTATAACAAAAATATTGACTTAAACCGAAATTCTATTCACGAATCTGAAAGGAATATGCCAATCATTGTTAGATCTTTCTTAGCGT
TCATTTAGACATTGCACTGCTTGGCAGTAGTAAAGGAACAATTTTTTCAGACTACGTGTAAATATAAAGCAAGGAAACTATCCAATTTAAATTT
ATTTTTAAATTAAATTTTTATTTTTTTTTTTAAGTTCCAAATAAAAACGTATTCATTCAATAGAAAAGTGAAGGGCATGTACATTTATCAAATTT
AAACAATTATTGTTTAAAAACTTAAGAGCTAGGAACAAATTATGTAATCTAATACAATTTTTTTTTCTCAAAGGGAAGATTTTTGGTTTTCATGT
AAAAAACTTTTTATCTAAGCCGCCATAAATTTTGCATGAATAAATTATTATTGTTTTCACGTAGCGAATCACGGATGTAATCTGGTAGCCGTTT
GGAAAAGATCTCAGTTCCCATTTTCAATGATTACGTTCTGAAATCAACTTAATCGCAACAAAAAATGATCCAGGGTCCCGCCAAATTAAGCCGA
TTTTAAAAATTCTCTCTAAAAATTCATTATTAATCAAATTATTTTTGCTGCAAAACCCTGAGACTGTGGCATTAAATTATTTTTTGTTTGGTTC
TTTTTGAGAAAAAAATATTTGAGGAATTATTTATAGGAGATAAATAGTTGTAGGCGTTTTAAACTTTAAACTGCTTGATCATATTCATGCGCAAA
GTGATTGCGGTTTTTGGGTTTCCAGATTCCCCTTGGCTTGGGATAATCCAAAACACCAACATTTTTCGTGTAATTACAAGTGTTATCTATAAAG
CTCTTAAAATTTTCATCTGCCCTGGTTATTTTTTTGTTGGAATTTTATCTGCAGACACTGTTCAGATAGTAGATGAAACTCCAGTATCTCGTTT
CAAAATGAAGCCACTTTTTGTAACTGCAGCTAATATGTCGAACAGATGAAATAAACTGAGTTAGTTGCAGTTGTAAACTCGGTGGAATTGTTTC
ATAAAATAAAATGGCTTTTTATGTGAATGTAATTATCGACTTTTACTTAAGTTAACACCAACCAAATATTAATTATTATTGCATATGTGTGGTG
AAATTAAACTGATGTATTTATTGTTATTTTTTATAATTAATATTTATTGCGTGAGTTTTTGTTGCGCTCCGCTATTGTAAGTATAATTTTTAAT
GTGTGGAAAACCAGTAGAAAATGGGCAACTATATCAATAAAATAAACTTTTCAAATTACA

Protein RF2: 3419/3251 -> 4732 (437/493AA)

KKHLLQKKRKAGQAGLDADNTKVPFEEKTKIVLVEKGRNHFDVLPEGWIQVTHNSGMPVYLHKTTRVCTLSRPYFLGPGSARKHLIPVNAIPCL
NYKKALEDEKKEQQEVEEKRTDETKLPENLPNARIETVQENIETQNLTAEALRNYCENVFQFQSINVLRFKSWSERRKFTKKRKHESQLQRPTL
PDGTKLITFPIKDIDSSEKTNPNAKKEWIMNPNGKSYVCILHEYVQHALKKQPTYQFTELENAATPYAATVIIMDMRYGVGYGTSKKQAKSEAA
RATLEILIPEMKSKITTDNQTGRTRKEKEQDLSFFDEIRVEDPRIAEFCAKTTEPAPYDILLTCLQRNFGLDDLKIHYQGNTSKHQGNEFTMTV
GKYTTTVACKNKRDGKQRASQAILQALHPSITSWGSLLRLYGNHSVKSFKEKKMEEQEITSLQSKAAVNQPNFAILNKLKMELGKLDEKRKTVR
PVGLVNTSEGEIILKSSNDKKNI

Comparison with *Tribolium* double stranded binding protein (563AA)

```
Query  4     LLQKKRKAGQAGLDADNTKVPFEEKTKIVLVEKGRNHFDVLPEGWIQVTHNSGMETPVYL  63
             L   KRKA +AGLD    K PFEEK K+VL+EK +NHFDVLPEGWIQVTHNSGM  P+YL
Sbjct  80    LKNNKRKASEAGLDESAAKQPFEEKDKVVLIEKSQNHFDVLPEGWIQVTHNSGM--PLYL  137

Query  64    HKTTRVCTLSRPYFLGPGSARKHLIPVNAIPCLNYKKALEDEKKEQQEVEEKRTDETKLP  123
             HK +RVCTLS+PYFLGPGS RKH IP++AIPCL+Y++AL+ EK +  E
Sbjct  138   HKNSRVCTLSKPYFLGPGSVRKHEIPLSAIPCLSYRRALDSEKTQTPESSS---------  188

Query  124   ENLPNARIETVQENIETQNLTAEALRNYCENVFQFQSINVLRFKSWSERRKFTKKRKHES  183
              +LPNARIETV+ENIE+QNL  E +R Y   +FQF++I V+RFKSWSERR+FTKKRKHE
Sbjct  189   -DLPNARIETVKENIESQNLKPEDVRKYASKLFQFKTIKVMRFKSWSERRQFTKKRKHEQ  247

Query  184   QLQRPTLPDGTKLITFPIKDIDSSEKTNPNAKKEWIMETNPNGKSYVCILHEYVQHALKK  243
             QLQRP LP GTKLITFPI+  ++SE TN NAKKEWIM  NPNGKSYVCILHEYVQHALKK
Sbjct  248   QLQRPNLPAGTKLITFPIQPNEASENTNSNAKKEWIM--NPNGKSYVCILHEYVQHALKK  305

Query  244   QPTYQFTELENAATPYAATVIIMETDMETRYGVGYGTSKKQAKSEAARATLEILIPEMET  303
             QPTY+FTELENAATPYAATV I   DM+  YGVGYGTSKKQAKSEAARATLEILIPEM
Sbjct  306   QPTYKFTELENAATPYAATVSI--NDMQ--YGVGYGTSKKQAKSEAARATLEILIPEM--  359

Query  304   KSKITTDNQTGRT-RKEKEQDLSFFDEIRVEDPRIAEFCAKTTEPAPYDILLTCLQRNFG  362
             KSKITTD +TG +  ++++QDLSFFDEIR+EDPR+AEFCAKTTEP+P+DILLTCLQRNFG
Sbjct  360   KSKITTDAKTGSSASRDQDQDLSFFDEIRIEDPRVAEFCAKTTEPSPHDILLTCLQRNFG  419

Query  363   LDDLKIHYQGNTSKHQGNEFTMTVGKYTTTVACKNKRDGKQRASQAILQALHPSITSWGS  422
             L+DL+I YQGNT K++ N+FTMTVGK+T TV CKNKRDGKQRASQAILQALHP ITSWGS
Sbjct  420   LNDLQISYQGNTLKNKKNQFTMTVGKHTATVVCKNKRDGKQRASQAILQALHPHITSWGS  479

Query  423   LLRLYGNHSVKSFKEKKMEEQEITSLQSKAAVNQPNFAILNKLKMELGKLDEKRKTVRPV  482
             LLRLYGN SVKSFKEKK+EEQEIT LQSKAA+N PNFAIL+KLK+EL KL +KR  ++P+
```

```
Sbjct  480  LLRLYGNGSVKSFKEKKLEEQEITLLQSKAAINSPNFAILDKLKLELSKLRDKRTQIKPI  539

Query  483  GLVNTSEGEIILK-SSNDKKNI  503
            G+    +E + + K SS++ KN+
Sbjct  540  GVFIPTESDSLPKLSSSNLKNV  561
```

Graphical representation

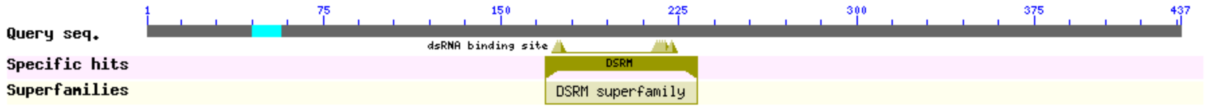

Exportin-5

>Cb.comp42659_c0_seq1 len=4175

cDNA

TTTTTTTTTTTTGAAAAAAATCGTTTATTTATAAAACGTCATAACGAAGATTGAAGCTAACAAAAATATGTACACAAATGCACGTTAGTACCTT
CCGAGTCGGTGTGGGCGGGGCGTCGGTGCATAGTTCCATCTGTCACTTTTCTAAAATCCGTTACGCTGTCGCACCCGTCGACCTAACCTTAACA
GGTAACTATGCGCGGATACCCCCCTCCCAAGGCAAATAAATAAAACAACCAACAACGCCAGGTGGGCGTGGCCGCGTTCGTGCGGGAATGTTCT
GTCCGATTAAAAGCCGCGTACGCGAAAAAAAAAAGACAGTACTAACTGAAAACGTTCCTCAGGTCGGTAATCAGTTCGGGTTTGTCCGCTTTTT
TTGGGTATCACTAGCTGCGGCAAGTCTTGGATCTTCACCTCCTTCTTAAACAGCTGACCGACGCTCCGGCCGATGAGGTTCGCTGTAATCTTTT
TGAACAAGTCCTTCTTGACTTTTTCGACCTTGTTGCCCTTGCTAGTGCTGCCCGATATCCTCTCGTCGAGTTTCTGCAAGTCGACGGGGTTCGC
GCCGGGTATCTGCTGCATCACCGCCAGCACTTCGACGAACGACGGTCGCAGCAGCTCGTATAATTGAGCACCGAGCGTGAGCAACGAGCCCTGA
TTCGCTTCGTGTTGCCCGTGCAGCATCAGAGCGTTCAGTATGGCGGCCATGATGTGGCTGGCCATCTCGCCGTTCAGGGAGTTATCTTGCGAAA
GCTGCCGAACGATTGGTCCGGCCAACATCGTCGCCTTCAACGAGGCGTTGCTGTCTATCCAGGACAGTGCTCCGAGCACCGCCAGCACGATCGG
TTGACACGTCTTCGTGTTGCGCAGAAGGATGAGGCCGAGGTCTGAGATGACCTCGGACGTGACGCTAGATCTAACTGGGGGCGGGGTCGGCGAG
TTGCTCAACTCTTCCGTTTCCATCGTTTCGGACGCGGTCTCGGGTGTCAGGCTACCGCCCACCAGCGCCACCTTCAACAGGTCGATGTATTCGC
GGGTCAACGCGCGCGTCAGGATGTCCTCGAGCACTTCCTGCGTGTCGGCGTTGTCTTCCTGCACCTCCCTGTTGCGGAACTCGATCACGCGTTG
CCAATTCGCGTGCAGACGGTTGAATATTATGGGCGTGATGTGACCGAGCAGGGGCAGGATGAGGGTCTCGTAGTACGGGGGCGGGCACGAGAAG
ATGAATGACTTGAGGAATACGCGGATAATCGGCCGGATTCGGTAGTCGGGGACGTACTGCAGGTACGCCAGCACGCTGTTGATGATCGCGAGCG
CCAGGTCCGGTATGCTGTACAGGTCCCTGCCCAGGGACGGTCCCATCGAGCCTATCAGGTGGTAAAAACTCTCGTGGAGGCACGTGAGGAAGCG
CTGCATCCTCTCGAGCGGACTCTGCACGATCAAGCTGTCCCCGTTATCGGCCGAATGGCCGGTGAGTCCGAGCAAGTTCGATTTCTCGGTCTCG
GGCATCGCCAGGCATCCCTTGTAGCTGTGATGGATCGCGTTCTGCGCGTCCGGCGTAAACAGCTCATTGAAGATACATATGAGGGAGAGCATGT
GAGGCAACAGCGGTATGACGTGAGGCGCGGCCGGATTACGACACACGGGGTTGCCGGATTCGGTCAGGGAGACGACGAAACCGCCCCTAGTGGC
GCGTTCGGGGTCGTCGGCCACGAGCAACGTTTAATCGCGCCGAGGACGAGGTTGACGCAGAAAACGAAACCGCTACGGTTGTGTCCGCACGGA
TCGTCCGTATTGGCGGGCACGGGTGGTTTGTCGAGACCGACGAACGAGATGAAAGGGACCGCCCCCTTCAGCGCGCCCGAATTAACGATCGAGA
CGAAACGGGCGCTAGCTTCGCGGAGCACCTCGCCTACGAACGCGCTCTGCCTCTCGTAATCGGCGAAATGGTTAGATATGAGGAGGAGAGCCTC
TTGCAGCGTGACCTTCTCGAGGGTGCTCAGTTGGGCGGGGCCGTCCGGTTTCGAAAGGTTCTCGACGGTCGTGCGGATCTGGTCGAAGACCGGC
AACAGCAGTAGCGGATACTTGTTGCCGATTTTTACCATGAGCGAGGCGGCGTGACGCCTCACGTTCTTGAGCGGTCGAGACCTCACCTCCCGCG
ACTGGTTGTTCGGGTTGTAAACGAGGGTCGAAAAGATCTTCTCGAGCACTTGGGGCAGTAGCGCGGCGCCAGACACCGCCACCGAGTTCGCCGC
CGGCGCCATCTGCCCGGTCGACATGCTCAAAAAAACGAACAGAGCCGATATGCACGTCAACAGGGTGGACATTATCAGTGGATCGGCTGGCTGA
TACCGCAGACACATCTCGAGCAGCCTCAAGCCCGATGGTATGGAAGGTCTCTCATGGGCCTGCAGGACCCTGCTGAGCACGCTCTCGAGGAAAT
TGGCGAGCGCCTCCCACTCTTGGTAGACGGGGTCGCTCATCGACAACCCGAACGTCGTGTTCGGCACCTGTAGGCACTTCACCAGCCACTGTTC
CACGTAGTTGAACGTTATCAACGGCGCGACTAGCGTCGCTTGCCGGAACGAATCCAAAAAATCCGACCGACACCGATAGAAGTAGGCGGAGAAC
TCCTCCTCGCTGTCAAAGTCTATTTTAGCGTAGGCGACCGTCTCGTTCAGGTCGTCGCCGGGACTCTTCCCCGCAGGATAATTAAATTTTATTA
TTTTCGGGGCGGTGCACTGCACCCACTTGGGAACGAACGACAAAAACGTCGGGTCCCTGGCCACGCCCTCCTGTTTCAGCATACTGTTCCACAG
CGGGTTGGCCAGGTGAGCGAGGAACAAGCTCGCGTGACCGCTGTACGCGAGCACCGCCTCGAGGAACATCGAGAAGTTTTGCGGCTGAAACGAC
GCGTCCTTGTTCCATAGGGTGCAGATCTGGGTGGTCAGGCCGCCAAAAACCTGGATCAGTTTCCGTTTGAACAGGTAATGCTGCTCGTTGAACG
TGGTGCCCGGATTTTTCGCTGCCGTCAACAGGCATTGCATAGTTTCGCTACCTAGCAGCATCAGCAATGGTTTCCTCTCGTCAATTTTACCCTT
ACGATTCACGATCTGAGACAGGCATTCCGCGGCTGAGTATTGGAAAGCAGTGTCATTGACCAACAAGCACAGTATTTGAAGGAGGCGTCCATTT
TGAGCGGTGATGTGAGTCATATTGACCCATTCGACGAACCCCGTTAAGGTTAGTAGTACCACTTGGACGACGCGCCCATGAGCTGCGGCTTTCG
ATGGGTCCGTCTCGGTACACGCCCGCAATTGGCTAACGTGCAACTCGATTAACCTCAAAAAAAACTCGAATATCAGAGACATGTTGGCGGTGAG
CGCGTGATAGATATCTTTGCGTCTTTGATTCGACTCGAGAGTTTGTAATAAAGCTACGTCCTCGACGAGACGTAAGAAAACAAACAACACCAGT
TCGGTTTGGGTCTCCCCGCAAGCGCAAGCGTCCGAAAGCTCGGCTAACAGGCCCGGCCACTGTTGTGGCCATTCCCTCTTAACCATTTCGACGA
CGACGCGGCTCAGAGCGTCTTTCATGTGAGGTTCGTCGCCTATGCCGCCGGCGGCCAGTAATTTCATCGCGTTTTCCTTGATAAAAATTTTTTC
ATGCTGGGATATTTGGGTCCAGCGGTATTTGACAGTGTGCTCCATGAGCTGCAGTCCGAAATGGCGAGCTATGAGGGAATGCTGCGTGCCGGCG
GCAAGAAAGAGCCCTGCTTCTGCACACAGGGGTGACGTTTCTTTGAAGCTCTCACACGCCTGATAGGCTTTGAGGCGGTCGCTTTGGGAAGCGC
CGGAACTCATAGTGAGCTCGACCGCCCTCGCTAAATCAGCCGCCAGGGCGGCAACGTCCGGGCCGGCCATCTGGATATAGTGCTTCAAGTATGT

CCCTTTTCGTTCTAAACGTCCATCACGTTAATTTAAAAAAAAATTAGTTCAAATATTTTTAAAACCTGAACCGGCAGCAGTCAAAAATGCTTCA
ATTTAGCTCCACAGAAAACGGATTGGTGGAAAAACATATAATTTGGAAATATGTAATAGCAAAATATAGTAAAATTGAAATGCGATTTGAAATT
TTTCTTTTTTGAAATTACAGCATATTTTCTGCAGCTCAG

Protein RF-3 -3924 -> -289 (1211AA)

MAGPDVAALAADLARAVELTMSSGASQSDRLKAYQACESFKETSPLCAEAGLFLAAGTQHSLIARHFGLQLMEHTVKYRWTQISQHEKIFIKEN
AMKLLAAGGIGDEPHMKDALSRVVVEMVKREWPQQWPGLLAELSDACACGETQTELVLFVFLRLVEDVALLQTLESNQRRKDIYHALTANMSLI
FEFFLRLIELHVSQLRACTETDPSKAAAHGRVVQVVLLTLTGFVEWVNMTHITAQNGRLLQILCLLVNDTAFQYSAAECLSQIVNRKGKIDERK
PLLMLLGSETMQCLLTAAKNPGTTFNEQHYLFKRKLIQVFGGLTTQICTLWNKDASFQPQNFSMFLEAVLAYSGHASLFLAHLANPLWNSMLKQ
EGVARDPTFLSFVPKWVQCTAPKIIKFNYPAGKSPGDDLNETVAYAKIDFDSEEEFSAYFYRCRSDFLDSFRQATLVAPLITFNYVEQWLVKCL
QVPNTTFGLSMSDPVYQEWEALANFLESVLSRVLQAHERPSIPSGLRLLEMCLRYQPADPLIMSTLLTCISALFVFLSMSTGQMAPAANSVAVS
GAALLPQVLEKIFSTLVYNPNNQSREVRSRPLKNVRRHAASLMVKIGNKYPLLLLPVFDQIRTTVENLSKPDGPAQLSTLEKVTLQEALLLISN
HFADYERQSAFVGEVLREASARFVSIVNSGALKGAVPFISFVGLDKPPVPANTDDPCGHNRSGFVFCVNLVLGAIKRCSWPDDPERATRGGFVV
SLTESGNPVCRNPAAPHVIPLLPHMLSLICIFNELFTPDAQNAIHHSYKGCLAMPETEKSNLLGLTGHSADNGDSLIVQSPLERMQRFLTCLHE
SFYHLIGSMGPSLGRDLYSIPDLALAIINSVLAYLQYVPDYRIRPIIRVFLKSFIFSCPPPYYETLILPLLGHITPIIFNRLHANWQRVIEFRN
REVQEDNADTQEVLEDILTRALTREYIDLLKVALVGGSLTPETASETMETEELSNSPTPPPVRSSVTSEVISDLGLILLRNTKTCQPIVLAVLG
ALSWIDSNASLKATMLAGPIVRQLSQDNSLNGEMASHIMAAILNALMLHGQHEANQGSLLTLGAQLYELLRPSFVEVLAVMQQIPGANPVDLQK
LDERISGSTSKGNKVEKVKKDLFKKITANLIGRSVGQLFKKEVKIQDLPQLVIPKKSGQTRTDYRPEERFQLVLSFFFRVRGF

Comparison with *Tribolium* chromosome region maintenance protein 5/exportin (1204AA)

```
Query  1     MAGPDVAALAADLARAVELTMSSGASQSDRLKAYQACESFKETSPLCAEAGLFLAAGTQH  60
             MAGPDVAALAADLARAVELTMS+GASQ+DRLKAY ACESFKETSPLCAEAGL+LAAGTQH
Sbjct  1     MAGPDVAALAADLARAVELTMSTGASQTDRLKAYNACESFKETSPLCAEAGLYLAAGTQH  60

Query  61    SLIARHFGLQLMEHTVKYRWTQISQHEKIFIKENAMKLLAAGGIGDEPHMKDALSRVVVE  120
             SLI+RHFGLQLMEHTVKYRWTQISQ EKIFIKENAMKLLAAGGI DEPHMKDALSRV+VE
Sbjct  61    SLISRHFGLQLMEHTVKYRWTQISQQEKIFIKENAMKLLAAGGISDEPHMKDALSRVIVE  120

Query  121   MVKREWPQQWPGLLAELSDACACGETQTELVLFVFLRLVEDVALLQTLESNQRRKDIYHA  180
             MVKREWPQQWPGLL+ELS+AC+CGE QTELVL VFLRLVEDVALLQTLESNQRRKDIYHA
Sbjct  121   MVKREWPQQWPGLLSELSEACSCGEIQTELVLLVFLRLVEDVALLQTLESNQRRKDIYHA  180

Query  181   LTANMSLIFEFFLRLIELHVSQLRACTETDPS-KAAAHGRVVQVVLLTLTGFVEWVNMTH  239
             LTANM++IF+FFLRLIELHV+Q R C ET+ + K+ AHGRVVQVVLLTLTGFVEWV+M+H
Sbjct  181   LTANMAVIFDFFLRLIELHVNQFRICGETNNTPKSTAHGRVVQVVLLTLTGFVEWVSMSH  240

Query  240   ITAQNGRLLQILCLLVNDTAFQYSAAECLSQIVNRKGKIDERKPLLMLLGSETMQCLLTA  299
             I AQNGRLL ILCLL+ND AFQY AAECLSQIVNRKGK+DERKPLL+L   E +QCL++A
Sbjct  241   IMAQNGRLLHILCLLLNDLAFQYPAAECLSQIVNRKGKVDERKPLLLLFNDEPIQCLVSA  300

Query  300   AKNPGTTFNEQHYLFKRKLIQVFGGLTTQICTLWNKDASFQPQNFSMFLEAVLAYSGHAS  359
             +KNPG   +EQHYLFK+KL+QV GGLTTQ+  LW KD+   +P NFS FLEA+LA+S H S
Sbjct  301   SKNPGAILDEQHYLFKKKLVQVLGGLTTQLVVLWGKDSISRPNNFSAFLEAILAFSSHQS  360

Query  360   LFLAHLANPLWNSMLKQEGVARDPTFLSFVPKWVQCTAPKIIKFNYPAGKSPGDDLNETV  419
             L L+H+ANPLWNSMLK E ++RDP FLS++P+WVQCTAPKI+KFNYPA K    D
Sbjct  361   LTLSHMANPLWNSMLKHEHISRDPVFLSYIPQWVQCTAPKIVKFNYPASKVQNTDTGGAA  420

Query  420   AYAKIDFDSEEEFSAYFYRCRSDFLDSFRQATLVAPLITFNYVEQWLVKCLQVPNTTFGL  479
             AYAKIDFDSEEEFS YFYRCRSDFLDSFRQAT+VAPL+TFNYVEQWL+KCLQVPN T GL
Sbjct  421   AYAKIDFDSEEEFSTYFYRCRSDFLDSFRQATVVAPLVTFNYVEQWLMKCLQVPNVTSGL  480

Query  480   SMSDPVYQEWEALANFLESVLSRVLQAHERPSIPSGLRLLEMCLRYQPADPLIMSTLLTC  539
              +SDP++ EWEAL+ FLES+LSRVLQA ERPSI SGLRLL++CL YQP DPLI+STLLTC
Sbjct  481   VLSDPLFHEWEALSTFLESILSRVLQAQERPSIASGLRLLQLCLVYQPVDPLILSTLLTC  540

Query  540   ISALFVFLSMSTGQMAPAANSVAVSGAALLPQVLEKIFSTLVYN-PNNQSREVRSRPLKN  598
             ISALFVFLSMSTGQMAP ANSVA SGAALLPQVL+KIFSTLVY  P+ QS++ RSR +KN
Sbjct  541   ISALFVFLSMSTGQMAPTANSVAASGAALLPQVLDKIFSTLVYAPPDEQSKDTRSRAVKN  600

Query  599   VRRHAASLMVKIGNKYPLLLLPVFDQIRTTVENLSKPDGPAQLSTLEKVTLQEALLLISN  658
             VRRHAASLMVKIGNKYPLLLLPVFDQIR TVENLS+ D  A LSTLEKVTLQEALLLISN
Sbjct  601   VRRHAASLMVKIGNKYPLLLLPVFDQIRATVENLSRSDSVAGLSTLEKVTLQEALLLISN  660

Query  659   HFADYERQSAFVGEVLREASARFVSIVNSGALKGAVPFISFVGLDKPPVPANTDDPCGHN  718
             HF DY+RQS FV EVL EA+A++  IV SGA + A  FISFVGLD PPV  + D+P GHN
Sbjct  661   HFCDYDRQSNFVREVLAEANAQWRLIVASGAFESASKFISFVGLDTPPVAPHADNPHGHN  720

Query  719   RSGFVFCVNLVLGAIKRCSWPDDPERATRGGFVVSLTESGNPVCRNPAAPHVIPLLPHML  778
             RS  VFC+NL+LGAIKRCSWP+DPERATRGGFVV+LTESGNPVCRNPAAPHV+PLLP +L
Sbjct  721   RSSIVFCINLLLGAIKRCSWPEDPERATRGGFVVALTESGNPVCRNPAAPHVVPLLPDIL  780

Query  779   SLICIFNELFTPDAQNAIHHSYKGCLAMPETEKSNLLGLTGHS-ADNGDSLIVQSPLERM  837
             SLI +FNELFT +AQN IH SYKGCL M ETEKSNLLGL GHS  D G+    VQSP+ERM
Sbjct  781   SLIRVFNELFTCEAQNLIHESYKGCLGMLETEKSNLLGLIGHSVGDLGELQAVQSPMERM  840
```

```
Query  838   QRFLTCLHESFYHLIGSMGPSLGRDLYSIPDLALAIINSVLAYLQYVPDYRIRPIIRVFL  897
             QRFL  LHES YH+IGSMGPSLGRDLY++PD+ LAIINSVLA LQ +PDYR+RPIIRVFL
Sbjct  841   QRFLFGLHESCYHMIGSMGPSLGRDLYTLPDIGLAIINSVLACLQCIPDYRMRPIIRVFL  900

Query  898   KSFIFSCPPPYYETLILPLLGHITPIIFNRLHANWQRVIEFRNREVQEDNADTQEVLEDI  957
             K FI+SCP P+YE ++LP++ HI P++ +RLHA W +V EFRNRE QEDNADTQEVLEDI
Sbjct  901   KPFIYSCPTPFYEAVLLPIVAHIAPLMLSRLHAKWLQVNEFRNREGQEDNADTQEVLEDI  960

Query  958   LTRALTREYIDLLKVALVGGSLTPETASETMETEELS--NSPTPPPVRSSVTSEVISDLG  1015
             LTRALTREY+D+LKVALVGG LTPET +E METE+LS  +   PPP RS++T+EVISDLG
Sbjct  961   LTRALTREYLDVLKVALVGGGLTPETNTENMETEDLSMDSPTPPPPTRSNMTTEVISDLG  1020

Query  1016  LILLRNTKTCQPIVLAVLGALSWIDSNASLKATMLAGPIVRQLSQDNSLNGEMASHIMAA  1075
             L+LLR+ KTCQ IVLAVLGALSWIDSNASLKAT L GPIVRQL  D+SLNGEMA+HIMA+
Sbjct  1021  LVLLRSEKTCQSIVLAVLGALSWIDSNASLKATFLTGPIVRQLVSDSSLNGEMAAHIMAS  1080

Query  1076  ILNALMLHGQHEANQGSLLTLGAQLYELLRPSFVEVLAVMQQIPGANPVDLQKLDERISG  1135
             +LNALMLHGQHEANQGSLLTLGAQ+YE+LRP+F+EVL VMQQIPG NPVDLQKLDERISG
Sbjct  1081  VLNALMLHGQHEANQGSLLTLGAQMYEMLRPTFLEVLGVMQQIPGVNPVDLQKLDERISG  1140

Query  1136  STSKGNKVEKVKKDLFKKITANLIGRSVGQLFKKEVKIQDLPQLVIPKK  1184
             STSKGNKVEKVKKDLF+KIT NLIGRS+GQLFKKEVKI DLP L    KK
Sbjct  1141  STSKGNKVEKVKKDLFRKITGNLIGRSMGQLFKKEVKIHDLPSLAFSKK  1189
```

Graphical representation

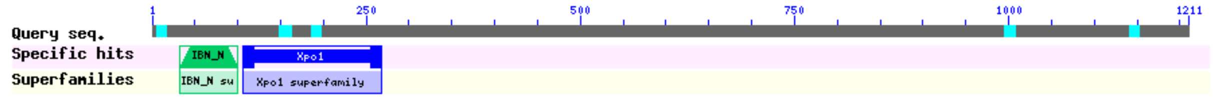

piRNAi pathway *Cylas brunneus*

AGO-3

>Cb.comp38974_c0_seq1 len=3095

cDNA

AAAGTAAAAGAAGAAGAAGAAGATTTTCTATGGTCTAATGGTATTTTGGTCTTAACCTGGATTGTGAATAAACAAGCTGTTCTTTTTCCAAAAA
AAATTATTTAACATCGATAATTTATGTTATTTGAGAGTTTGCTTCTAAAAGAACACGGTGTTTGATCGTGACGGCTAAGTGTAGAACTCGTTTT
TTTTTATTTCTTGCCTTTGGGACCATGGCAGATAGTGGGGTCCCGGCGCCTCGTGGCCGAGCCGCTGCTTTAAAAGCGAAACTTGATCAGTTAA
AAGCTCAAACTTCAGTTGGAGTTGCTAGCGGCGATGGTAGGGCCGAACACCCAAAACCCAGAGGTAGAGCTGCAATGCTGCAGAGGTTACAAAT
GCAAAAAGTCGGAGAGAGTGGTGAGTGCAGTGTAATGGCTGCAAAGTCTGAGGGCTCTTGTAGTGATTCTGAAAGAGTTAAACCTATTTCCAAG
ATGGAAACTGTGTCAAAAAAGTTAGAGGAGGTGAACATATCGGTGGAAAGAAAACCTGTAAACTATAAGGGCGAGTCAGGAAAACCACTTAAGT
TGTCTGCTAATTACATTCGACTCGACATCGAAAAAGGCCATGGGGTATTTGAGTATGAAGTAAAATTTGATCCTGAATTGGACGCGAAAAACCA
GCGAATACGAATGGTGAACCAAATGATGAATGACATGGAATCAGTAAAGGTTTTTGATGGAGGCCATTGCTTGTATCTTCCACACAAAATTTCT
GAAGAGATAAAGACATACAAGGGATTATTGCCTGGAGGCGACCAAGAGGTTACAGTCACTATTATTTACAAGCGACAGAAGAGTTTTGGAGATA
GAGAATGTCTACATTTGTACAATATACTGTTTAAAAGAATTATGCACATTTTACTTTATACTCAGATGGGAAGAAATTATTTTAATCCTGCCCA
TAAGCATTTGATTCCTCAGCATAAATTGGAGGTGTATCCAGGTTTTGCGGTAACTGTAGACGAACTTGAAGGTGGTTTGTTGTTATGCCTAGAT
ACTCAGCATAGAGTATTACGTACTCAAAATGCCTATGAACTCTTAACTGAGATAAGATGTGCGTCAGATCCAAGAAGGTTTAAAGAAGATGCCA
GGAAAAGTATTATTGGCTCGTGTGTTTTTACCCGTTACAACAACAAGACCTATATTATTGATGACCTTCTGTGGGACATGACACCAAACGATAC
ATTCCCAACCCGGGATGGAAACACAATCACTTTTGTGGATTATTACAAACAGCAGTACAATATTAGTATTAATGACGTAAACCAACCTCTGCTT
TTACACAGAAGAAGTGTTAAAGTGTCAGGCAAAGCTGAAAAGGAGGATAGAATGATTTGTCTTGTACCTGAACTGTCATTTTTGACAGGATTGA
CGGAGACCATGAGGAACGATTTTAAAGTTATGAAAGATGTTGCCCAGTACACTCGGGTTACGCCACACCAGAGAATGCAGGCCCTGAGAGTATA
TTTGGAGAACGTCAGAAGCAGTGAGAAGGCCCAACAAGTTCTGGCGCAGTGGGGTTTATCAATAGCCCCTGCCAATATTGAATTACAAGGTCGT
CAGCTTGAGCCTGAACGCATCCGATTTGGAAATTCTAGTGAAATAACAGCCGGTCTCGGGGCCGATTGGAACCGAGATTTGGCCAACAACACGG
TTGTCGCGCCGGTAGATCTCTATAATTGGGTGGTGTTTTACACCCAGCAAGATACAAAGTATGCCAATGATTTCGTCCAGCATATGGGTAGACT
GGCTGGTACTTTAGGTTGCGTCATCAGCAAACCTCGAATGGAGAGGCTTCAGAATGATCAAACTCAAACTTATGTAACTGTTGTTAAGGATAAG
ATCGACAAAAATGTTCAGGTTGCTGTGTTTATTTGCCCGACGATGAGGAGCGATAGATACGCTGTAATAAAGAAACTGTGCTCCGCCCAATTGC
CAGTCGCGTCACAGGTGATCAATTCCCGGACGTTGTCCAAGCCGGACAAACTTCGTTCGATTATTTTAAAGATTGCATTGCAAATTAACTGCAA
ACTGGGCGGCAGTCTGTGGACTGTACGGTTTCCGTTCAGCGGTTGGATGATATGTGGCATCGACGTTTATCATGGTTCGCCGCCTAATTCGGTA
TGCGGTTTTGTAACCAGCGTGAATGACAGTATTTCACGGTGGTACTCGACAGCATTGTTTCAAAGCAAAGAGCTAGGCGACTTTTTCAAGATGG
CATTTATGAAATCGCTTGAGCAGTACAAAGATAGTACCGGAAATTTCCCCGCGAAGGTAGTCATCATTAGGGACGGAGTGGGAGACGGACAGCT
AGACCACTGTCGCAGGTATGAGGTGGAGCAGTTTGAAAATGTAATCAGAGAGTTTGGGCTATCGACAACCATATGTTTTGTGGTGGTACAGAAA
CGCATCAACACTAGGATGTTCAGTTTTGGCAGAAACGGTCAGGCGGAAAATCCGCCACCAGGCACGATTCTAGATCACACAGTGACGAGAAAAT
ATTTGTACGATTTCTTCATGGTTCCGCAGAGTGTTAGACAAGGAACGGTCAACCCAACTCACTATATAGTGCTTCACGATACTTGCAAACTGAA
ACCTGACCATGTACAAAGGTTGTGCTACAAGCTTTGTCACTTATATTACAATTGGCCAGGGACCATAAGGGTACCTGCCCCATGCCAATATGCC
CACAAATTGGCTGCTATGGTGGGTCAGTATGTCAAGACAAAGCCTAGCGCGGAGCTTGCGGACAGGCTGTGGTTCTTATAATTAGTTTGGAGGA
TTTAAAATTTTCGGTTGATTTTCGCCATTGTCATTGTGCATCCTTCAGTTCAGTTTAGAATAAATAAATCATTGATATTGTTTCACTTACTTTG
GTAAAAGTTTGCATTTGTTTTATATTCAGTAATGGATTGAGGGACAAAAAAGTTCTGAATGTCTCTTTCTTTGTATTGTATCATAGTGTTGATG
TACCTTTAGTTATTGTAATTTTTCTTGTATGTAGTTATAATATTCAGTTTTCTAATTTCAATAAATATTTTTCTAATATAAAAAAAA

Protein RF3 213 -> 2807 (864AA)

MADSGVPAPRGRAAALKAKLDQLKAQTSVGVASGDGRAEHPKPRGRAAMLQRLQMQKVGESGECSVMAAKSEGSCSDSERVKPISKMETVSKKL
EEVNISVERKPVNYKGESGKPLKLSANYIRLDIEKGHGVFEYEVKFDPELDAKNQRIRMVNQMMNDMESVKVFDGGHCLYLPHKISEEIKTYKG
LLPGGDQEVTVTIIYKRQKSFGDRECLHLYNILFKRIMHILLYTQMGRNYFNPAHKHLIPQHKLEVYPGFAVTVDELEGGLLLCLDTQHRVLRT
QNAYELLTEIRCASDPRRFKEDARKSIIGSCVFTRYNNKTYIIDDLLWDMTPNDTFPTRDGNTITFVDYYKQQYNISINDVNQPLLLHRRSVKV
SGKAEKEDRMICLVPELSFLTGLTETMRNDFKVMKDVAQYTRVTPHQRMQALRVYLENVRSSEKAQQVLAQWGLSIAPANIELQGRQLEPERIR
FGNSSEITAGLGADWNRDLANNTVVAPVDLYNWVVFYTQQDTKYANDFVQHMGRLAGTLGCVISKPRMERLQNDQTQTYVTVVKDKIDKNVQVA
VFICPTMRSDRYAVIKKLCSAQLPVASQVINSRTLSKPDKLRSIILKIALQINCKLGGSLWTVRFPFSGWMICGIDVYHGSPPNSVCGFVTSVN
DSISRWYSTALFQSKELGDFFKMAFMKSLEQYKDSTGNFPAKVVIIRDGVGDGQLDHCRRYEVEQFENVIREFGLSTTICFVVVQKRINTRMFS
FGRNGQAENPPPGTILDHTVTRKYLYDFFMVPQSVRQGTVNPTHYIVLHDTCKLKPDHVQRLCYKLCHLYYNWPGTIRVPAPCQYAHKLAAMVG
QYVKTKPSAELADRLWFL

Comparison with *Tribolium* argonaute-3 (853AA)

```
Query  7    PAPRGRAAALKAKLDQLKAQTSVGVASGDGRAEHPKPRGRAAMLQRLQMQKVGESGECSV  66
            PAP+GR A L+  L + K   + G          PK RGRA +LQ++Q  K  ++G  S
Sbjct  5    PAPKGRGALLEM-LKKHKEARAGGAGEPVEEQAPPKTRGRAMLLQKIQEAKERKAGGDSG  63

Query  67   MAAKSEGSCSDSERVKPISKMETVSKKLEEVNISVERKPVNYKGESGKPLKLSANYIRLD  126
             +     S   SE  + +S    V+K L EV I+   +  +Y+GESG P+K +ANYI L+
Sbjct  64   QLSTPGPSTVPSETRRGVSG---VTKALGEVAITAS-ETCSYRGESGTPIKATANYILLN  119
```

```
Query  127  IEKGHGVFEYEVKFDPELDAKNQRIRMVNQMMNDMESVKVFDGGHCLYLPHKISEEIKTY  186
            +EK  GVFEYEV+F P++DAK+ RI++VNQ + ++ + KV+DG  CLYLP       + +
Sbjct  120  VEKDRGVFEYEVRFQPDIDAKSNRIKLVNQALGELSTTKVYDGDVCLYLPCLAFSPRQEF  179

Query  187  KGLLPGGDQEVTVTIIYKRQKSFGDRECLHLYNILFKRIMHILLYTQMGRNYFNPAHKHL  246
            + ++P  +  VT T+IYKR++     ECLHLYN+LFKRIMHILLY +MGRNYF+P HK+L
Sbjct  180  ESVIPNTETPVTTTLIYKRKRKLS--ECLHLYNVLFKRIMHILLYQRMGRNYFSPDHKYL  237

Query  247  IPQHKLEVYPGFAVTVDELEGGLLLCLDTQHRVLRTQNAYELLTEIRCASDPRRFKEDAR  306
            +PQHKLEV PGF V VDE+EGGL++CLDTQHRV+R+Q  YEL  EIR A++PR F+E+
Sbjct  238  VPQHKLEVLPGFCVHVDEMEGGLMVCLDTQHRVIRSQTVYELFHEIR-ATNPRNFREEVT  296

Query  307  KSIIGSCVFTRYNNKTYIIDDLLWDMTPNDTFPTRDGNTITFVDYYKQQYNISINDVNQP  366
            K++IG+CV T+YNN+TYIIDD+ W+M P DTF  R      F+DYY++ YNI I DV+QP
Sbjct  297  KNVIGACVLTKYNNRTYIIDDIAWNMNPKDTFEDRSKGPSCFIDYYREHYNIRIEDVDQP  356

Query  367  LLLHRRSVKVSGKAEKEDRMICLVPELSFLTGLTETMRNDFKVMKDVAQYTRVTPHQRMQ  426
            LL+ R+ VK S   + E RMICL+PEL +LTGLT+ MRNDFKVMKDVA +TR+TP QRM
Sbjct  357  LLITRQ-VKQSPDGKIE-RMICLIPELCYLTGLTDAMRNDFKVMKDVAAFTRITPNQRML  414

Query  427  ALRVYLENVRSSEKAQQVLAQWGLSIAPANIELQGRQLEPERIRFG--NSSEITAGLGAD  484
            ALR YL+ VR SEKA+QVL+ WGLS+A   ++++ R L  E I FG  ++       G D
Sbjct  415  ALRTYLDRVRQSEKAKQVLSGWGLSLADDTVDVKARVLPQEAIYFGGPDAEAHKYTGGTD  474

Query  485  WNRDLANNTVVAPVDLYNWVVFYTQQDTKYANDFVQHMGRLAGTLGCVISKPRMERLQND  544
            WN+ +++N +  PV++ NW ++YT++D KYA +F Q + RL   +GCVI  PR   L +D
Sbjct  475  WNKAISDNKLTGPVNITNWQLYYTRRDQKYAANFAQTIVRLGKGMGCVIQDPRHIVLDDD  534

Query  545  QTQTYVTVVKDKIDKNVQVAVFICPTMRSDRYAVIKKLCSAQLPVASQVINSRTLSKPDK  604
            +T+TY+T ++D +  N QVAVFICPT+R+DRY++IKK+C   +PVASQVI S+TLS P K
Sbjct  535  RTETYMTAIRDNV-ANTQVAVFICPTLRADRYSIIKKMCCVNIPVASQVILSKTLSNPQK  593

Query  605  LRSIILKIALQINCKLGGSLWTVRFPFSGWMICGIDVYHGSPPNSVCGFVTSVNDSISRW  664
            +R+II KIA+QI CKLGG+LW+V+ P SGWM+CGIDVYHG+   SVCGFV S+N S++++
Sbjct  594  VRTIIHKIAMQITCKLGGTLWSVKIPVSGWMVCGIDVYHGANNQSVCGFVASINGSMTKY  653

Query  665  YSTALFQSKELGDFFKMAFMKSLEQYKDSTGNFPAKVVIIRDGVGDGQLDHCRRYEVEQF  724
            +S A+FQ  E+GD+FKM F + L+  KD  G FP+KV++ RDGVGDGQL+HCR+YE+ Q
Sbjct  654  FSKAMFQDGEIGDYFKMPFRQMLQAAKDREGAFPSKVIVFRDGVGDGQLEHCRKYEITQL  713

Query  725  ENVIREFGLSTTICFVVVQKRINTRMFSFGRNGQAENPPPGTILDHTVTRKYLYDFFMVP  784
            + VI+E  + TTI FVVVQKRINTR+F        ENPP GT++D+ VTR+  YDFF+VP
Sbjct  714  QEVIKELNIETTITFVVVQKRINTRIFRTVNETNFENPPSGTVVDNMVTRRQFYDFFLVP  773

Query  785  QSVRQGTVNPTHYIVLHDTCKLKPDHVQRLCYKLCHLYYNWPGTIRVPAPCQYAHKLAAM  844
            QSVRQGTVNPTHY+VL D   +KPDH+QRL YKLCHLYYNW GTIRVPAPC YAHKLAA+
Sbjct  774  QSVRQGTVNPTHYVVLVDEGNIKPDHLQRLAYKLCHLYYNWSGTIRVPAPCLYAHKLAAI  833

Query  845  VGQYVKTKPSAELADRLWFL  864
            VGQY+K  PS +L D+L++L
Sbjct  834  VGQYIKKTPSTQLDDKLFYL  853
```

Graphical representation

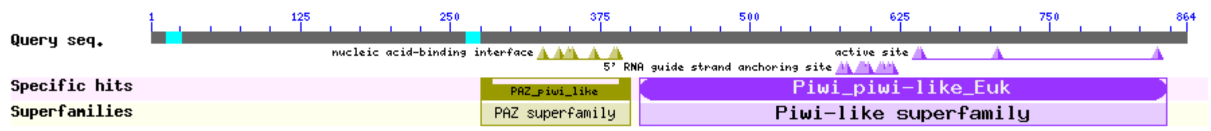

Aubergine

>Cb.comp37817_c0_seq1 len=3215

cDNA

TTTTTTTTTTAACTATGCTCAGTCGGTAAGATAGGAATAAGTTTTATCATAAGGGTCTCCATCTACCGTTTTTGCTGAAATAGCCA
TTTCTATTTTATTTTCGGATTGGCAGCGTTAATGCTGTGAAGATTTTGTCTTTGGAGAAAGATGGAACCAAGAGGGAAAGGAAGGG
CACGAGGTCGTGCTCGTGCCGGAGCCCAACAAGCAGGAGGTCAGCCTCAACAACCAAGACCTGGAGGTGCTCAAGGACCAGCTCCA
CAAGGAGCTTGGGGTGCGAGGCCTACTGGTGTTTCGACACAAGGAGCTCCTCCTGGAGCTTGGGCTAGTAGATCTCAGCCCACTCA
GCCAGTCCAACAACAATGGACAGCCAGACCACCTGCACCTGAAGCTGTTCAGACGCAAACTGTTGGTCGAGGATCAAGGCAAGGAG
GAGGAGGCGGAGATGATAGATCAGTCACAGGCGAAACAAGACAAGTTTCTCAAGGTGGTGATCCCGGTTTGGAGCGTCGTGGTGGA
GGAAACGGAGGAGTAAGGGGCCGAACCAACAGAAATGAAATCATAAGCACTAGACCTTCTCATGTTCTATGTAAAAAAGGTACAGA
TGGCACACCCATTAGACTCAGAGCCAACTATTTTCTATTAATCCCAAAAGGTCATTGGGGCCTAAATCAATATCGGGTGGATTTTG
TTCCGGATCTCGACAATACATCAACTCGAAAATATTTAGTAAGGACAGGATTACAAAACAAAAATGTGTCTGGTTATTTATTCGAC
GGTACTGTTCTGTACACTCCTAATCGCATTCATCCCGATCCACTTTCATTTGTTGTGGACACAGATGATGGAAATCATGTTACGGT
GACGGTACGTTTAGTTGGGGAAGTGAAATGGGGCGATTGGCACTACTTACAACTATTTAACATTATGATGAGGAAGTGTTTAACCT
TTATGGACCTTAAATTAATGGGAAGGAACTACTTCGATCCAAAGCTAAAGATATCCGTACCTGAACATAATCTCGAGCTATGGCCG
GGATATTTCACGTCAATAAGACAGTATGAAAAAGATATAATGATAAATGCTGACCTGTCATTTAAAGTTTTACGAACAGACAATGT
GTATGACTTGTTACTCGAATGTGGTCAAAGTAGGAATCCCCAGAACGAGTTTCGCCAGAGAGTTATTGGGAATATTGTTCTAACAT
ATTATAATAATAAAACTTATCGAATTGATGATGTAGATTTCAAACAGACACCAGCATCAACATTTCAAAAGAGAGATGGATCATCA
ATCAGCTATGCTCAATATTTTCAAGAGCGATATAGAGTTCCAATTAATGTTATGGAACAGCCTATGCTTGTTTCAAGGAGCAAACC
GCGAGAAATTAAGGCCGGCATGCCTGAAACTGTTGTACTTATTCCATCTCTGTGTATAATGACCGGCTTGACTGATAAACAGCGCG
AAAATTTCCATTTAATGAAAGCATTGGGTGAACATACTAGGGTTGGACCCCAAGGTCGCATTCAAAAACTTAGGGAATTCAGTCAG
AGGTTGCAGAATTGCCGCGAAGCAATGGAGGAAATAAGACGTTGGGACCTTGACATGGCTCGAGATCTTATTGAAATAACTGGGCG
GGTGTTACCAGAAGAGACTTTGGTTTTAAGGAACGGCACGCAAATCACCGGCGGGCCTGAAGCTGATTTCACGAAAAGTTTAAGGA
CTGCGCCAATGTACACTGTTGCTCCTGTGGATAAGTTGGCCGTGCTTTGCCCTGGCAGATTCAGGCAGGGGACGAGTGAGTTTATA
AACTGCCTGCTAAGAGTTGGAAAAGGGATGCATTTCAATCTTGGAAATCCAAAGATAATAGACATGCAGGATGATCGTGCGCAATC
CTACTTAGACAATCTTGATCACATCATTACAAATTTGCAACCAAAAATGGTATTGTGTGTATTACCGAATAACTCAGCTGATCGAT
ACAATGCAGTAAAAAAGAAATGTTACGTTGATCGCGCCATGCCTTGTCAGGTTGTTGTTGGAAAAACATTATCCAACAAAGGTGTG
ATGTCGATTGCAACTAAGGTTGCAATTCAGATGAATTGTAAGATGGGAGGAGCTCCATGGGGAACAAGTCTGCCTAAAGCAACAAT
GGTCGTTGGGTATGATGTATGTCGTGACACTGCTAATAGAGGAAAAAGTTTTGCTGGAATGGTGGCCTCCATGGACACTGCTTGTA
CCCAGTATTATAGTCTGGCAACTGAACATGAACAAGAACAAGAGCTAAGCAGCAGTATTGTATCATTTCTTTTGTTTGCTTGTAAA
ACATATCAGGAGAGAAATAAAATTATTCCAGAGCGCATAGTTATATTTAGGGACGGGGTTGGTGATGGCCAGATACAGTATGTCAA
AGAACATGAGCTTGAACTCGTTAAAAAGAAGCTGCAGGGTGACGTTTACAAGACTCAACCTCTTAAGATGGCATTCATAATTGTGA
CAAAAAGAATAAACACAAAAATATTCCGAACTCAAGGTGTTGGACCTAAAAATGATTTTAATCCACCACCTGGAACCGTAGTTGAT
GATGTTATTACTTGGCCTGAAAGGTATGACTTCTATATCGTATCTCAATGTGTTAAGCAAGGTACTGTTGCTCCAACTAGTTACAA
TGTTATTGAAGACACTTTGGGTATCGATGCGAATAAATTGCAGAGATTCACGTTTAAGTTGTGCCATATGTACTACAATTGGTCAG
GCACTGTGCGAGTGCCAGCGCCATGCCAGTATGCGCATAAGCTCGCATTTCTCACAGCGCAAAGTTTGCACAGAGCTGCTAATCCG
GCTTTGAACAACACTTTATATTATCTTTAAATGTTTGGTCAAATATTGTTTAAAATTTGACAAATCGTTCAGTTTTCTATTTACTA
AGAGTATAGTTTTAATTTTATTTGTTCCAATACTTTTTGCCAGTTTGTTGACCTTTTCGGTTTTAATCTCTTGTCTAATAAGGCTT
TTCTTCTCGTCTCTTGAAACATTTTATGTTAAAAGTTGGGTTATCATTTTCTGATTACTCATTAAGAATTTTTGATACCATGATTT
ATTTTTTCCTTTGCATCTTGACTGGATGCATGTCTAGTTCTAAGGTTTTTACACGACGTTAAAGTATATAATGTTATTTTTTTAAG
AGCCTTTTCAATAAAAGTTTAGAAAAAAAAAAA

Protein RF1 148 -> 2868 (906AA)

MEPRGKGRARGRARAGAQQAGGQPQQPRPGGAQGPAPQGAWGARPTGVSTQGAPPGAWASRSQPTQPVQQQWTARPPAPEAVQTQTVGRGSRQG
GGGGDDRSVTGETRQVSQGGDPGLERRGGGNGGVRGRTNRNEIISTRPSHVLCKKGTDGTPIRLRANYFLLIPKGHWGLNQYRVDFVPDLDNTS
TRKYLVRTGLQNKNVSGYLFDGTVLYTPNRIHPDPLSFVVDTDDGNHVTVTVRLVGEVKWGDWHYLQLFNIMMRKCLTFMDLKLMGRNYFDPKL
KISVPEHNLELWPGYFTSIRQYEKDIMINADLSFKVLRTDNVYDLLLECGQSRNPQNEFRQRVIGNIVLTYYNNKTYRIDDVDFKQTPASTFQK
RDGSSISYAQYFQERYRVPINVMEQPMLVSRSKPREIKAGMPETVVLIPSLCIMTGLTDKQRENFHLMKALGEHTRVGPQGRIQKLREFSQRLQ
NCREAMEEIRRWDLDMARDLIEITGRVLPEETLVLRNGTQITGGPEADFTKSLRTAPMYTVAPVDKLAVLCPGRFRQGTSEFINCLLRVGKGMH
FNLGNPKIIDMQDDRAQSYLDNLDHIITNLQPKMVLCVLPNNSADRYNAVKKKCYVDRAMPCQVVVGKTLSNKGVMSIATKVAIQMNCKMGGAP
WGTSLPKATMVVGYDVCRDTANRGKSFAGMVASMDTACTQYYSLATEHEQEQELSSSIVSFLLFACKTYQERNKIIPERIVIFRDGVGDGQIQY
VKEHELELVKKKLQGDVYKTQPLKMAFIIVTKRINTKIFRTQGVGPKNDFNPPPGTVVDDVITWPERYDFYIVSQCVKQGTVAPTSYNVIEDTL
GIDANKLQRFTFKLCHMYYNWSGTVRVPAPCQYAHKLAFLTAQSLHRAANPALNNTLYYL

Comparison with *Tribolium* Aubergine (901AA)

```
Query  103  VTGETRQVSQGGDPGLERRGGGNGGVRGRTNRNEIISTRPSHVLCKKGTDGTPIRLRANY  162
            + GE  Q +Q G  G  R GGG   VRGR  R EI+ TRP ++  KKGT GTPI L ANY
Sbjct  107  IAGEGDQGNQEGSQGAAR-GGGASSVRGRVVRKEILYTRPQNLKSKKGTIGTPINLIANY  165

Query  163  FLLIPKGHWGLNQYRVDFVPDLDNTSTRKYLVRTGLQNKNVSGYLFDGTVLYTPNRIHPD  222
              LI +G W L QYRVD  PD+DNT+ RK LVR  +++    GYLFDGTVLYT  RI+ D
Sbjct  166  LPLIKQGKWCLYQYRVDMAPDVDNTNKRKELVRVAVKDLLKGGYLFDGTVLYTTQRINND  225

Query  223  PLSFVVDTDDGNHVTVTVRLVGEVKWGDWHYLQLFNIMMRKCLTFMDLKLMGRNYFDPKL  282
             +   VD + G +V +T+RLVG++ WGD HY+QLFNI++RKCL  M L+ +GRNYF P
Sbjct  226  SVDLFVD-NSGENVRITIRLVGDLAWGDMHYIQLFNIIIRKCLKLMGLQQVGRNYFMPDN  284

Query  283  KISVPEHNLELWPGYFTSIRQYEKDIMINADLSFKVLRTDNVYDLLLECGQSRNPQNEFR  342
            KI + EH ++LWPGYFTS+RQ+EKDI++N DL FK +RTD VYD LLEC Q  N + EF+
Sbjct  285  KIVISEHKIQLWPGYFTSMRQHEKDILLNVDLQFKFMRTDTVYDNLLEC-QGANARKEFQ  343

Query  343  QRVIGNIVLTYYNNKTYRIDDVDFKQTPASTFQKRDGSSISYAQYFQERYRVPINVMEQP  402
             ++IG++VLT+YNNKTY+IDDVDF  TPA TF+ +DGS  ++  YF+++Y V I V +QP
Sbjct  344  SKIIGSVVLTHYNNKTYKIDDVDFNSTPAHTFKLKDGSETTFKDYFKKKYNVDIRVKDQP  403

Query  403  MLVSRSKPREIKAGMPETVVLIPSLCIMTGLTDKQRENFHLMKALGEHTRVGPQGRIQKL  462
            ML+SRSKPREI+ G+PETV L+P LC+MTGLTD+QRENF+LMK L  HTR+G +GRI+KL
Sbjct  404  MLISRSKPREIRVGVPETVYLVPELCLMTGLTDRQRENFNLMKMLATHTRIGVEGRIKKL  463

Query  463  REFSQRLQNCREAMEEIRRWDLDMARDLIEITGRVLPEETLVLRNGTQITGGPEADFTKS  522
             EFSQ+L N  + + EIRRW LD+   L+   GRVLP+ET+V  N  + + GP+AD+TK
Sbjct  464  MEFSQKLHNKPDVVNEIRRWGLDVGNSLVRFQGRVLPQETVVGGNDAKYSAGPQADWTKE  523

Query  523  LRTAPMYTVAPVDKLAVLCPGRFRQGTSEFINCLLRVGKGMHFNLGNPKIIDMQDDRAQS  582
            LR+ PM  +  +++LAV+C  R +  T +FI  L +   GM ++LGNPKI D+QDDR+ S
Sbjct  524  LRSRPMLYMPKMERLAVVCSHRNKSATQDFIQLLAKTAGGMRWSLGNPKIFDIQDDRSGS  583

Query  583  YLDNLDHIITNLQPKMVLCVLPNNSADRYNAVKKKCYVDRAMPCQVVVGKTLSNKGVMSI  642
            Y++ ++ II   QP M+L +LPNNS +RY+A+KKKCYVDR +P Q+ V + L++KGVMSI
Sbjct  584  YIEQIEKIINMNQPTMILVILPNNSTERYSAIKKKCYVDRGIPTQMFVARNLTSKGVMSI  643

Query  643  ATKVAIQMNCKMGGAPWGTSLP-KATMVVGYDVCRDTANRGKSFAGMVASMDTACTQYYS  701
            ATKVAIQMNCK+GGAPW   +P    MVVGYDVCRDT N+ KSFAG+V S+D   +++Y+
Sbjct  644  ATKVAIQMNCKIGGAPWCVPIPLSGLMVVGYDVCRDTVNKKKSFAGIVGSLDKNISRFYN  703

Query  702  LATEHEQEQELSSSIVSFLLFACKTYQERNKIIPERIVIFRDGVGDGQIQYVKEHELELV  761
            +  EH+ E+ELS +  + ++  CK Y+E+N   PERI+I+RDGVG+GQ+ +V EHE+  +
Sbjct  704  ICCEHKMEEELSDNFAAAVVLLCKQYKEQNGHYPERILIYRDGVGEGQLPFVVEHEVANI  763

Query  762  KKKLQGDVYKTQPLKMAFIIVTKRINTKIFRTQGVGPKNDFNPPPGTVVDDVITWPERYD  821
            K+KLQ ++Y    +KMAF++V+KRINT+IF  +        NPPPGTVVDDVIT PERYD
Sbjct  764  KRKLQEEIYINGEVKMAFVVVSKRINTRIFTEKD-------NPPPGTVVDDVITLPERYD  816

Query  822  FYIVSQCVKQGTVAPTSYNVIEDTLGIDANKLQRFTFKLCHMYYNWSGTVRVPAPCQYAH  881
            FYIVSQCV+QGTVAPTSYNVIED++G+   KLQ  T+KL HMYYNWSGTVRVPAPCQYAH
Sbjct  817  FYIVSQCVRQGTVAPTSYNVIEDSMGLPPEKLQYLTYKLTHMYYNWSGTVRVPAPCQYAH  876

Query  882  KLAFLTAQSLHRAANPALNNTLYYL  906
            KLAF+ +Q +HR A+  L+N LYYL
Sbjct  877  KLAFMVSQYIHRPAHHDLDNVLYYL  901
```

Graphical representation

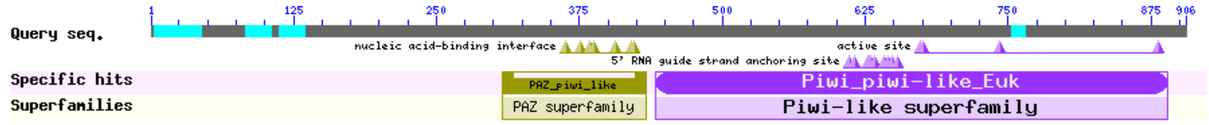

Zucchini

>Cb.comp31873_c0_seq2 len=840

cDNA
ACAAATTTGACACATATATTAGAAAAAATATATTTCTTTTAACATTAGGTATTTACAAATGATTCCATAAATCTTCAAATTCTTGACTAAACAT
TTTAATCCAATCTGAATTGTTAGTTAAAACAACTGCCTCAAAATTCTTGCATATCCCTTGCATTGTTAAGTTCAAGCTTCCAAAAAACATTTTT
GCCAACTCTGGATCGTTTTCGTCAATGAGACAGAATTTGTGATGCACCATGACATCCATGGAAACAGGAATTTTATAAGAAATGCTCCATTTAT
CAAAATACTTCATATTGGATATGGATGGCGTAGTCTGAGACATTGCATTGTCCATGATAAGTCTCACTTTTACTCCTCTACGAGCAGCATTCAC
AAGCTCCTTAGATATTTGCTTTAAGGTCATGGTATACATGCACATACTGATACTGTGTTTGGCGCTTCCTATAAACTTTATCAGTCTTAATAAT
TCAACGTAAGAACAAGCCTCTCCGCACTCATTTTTATTTAGAATGTGAGGTCTGCATTCAAAGTTCTTGTAATCGAAAAAAATGCACTGATAGT
AGCTATTGTCGTCGGCATTTTGAAATTTTTCGATTAATTTTCGATAACGTTTTTTCAAAAAATAGCTTATTATTAGGGGTAGTGTGGAAAAACT
TAAAAATATAGCTGCACAACTCAATTTGTTCATAACTGCGATTCAGTAGACACCATAGGTGATCTTCTTCCAATAGTTTTTTCGTGAACAAGAC
TGTCAGGGTCTTTGGCCCAAGCACGAATGAAATATAAAATAGAAGAAAGTCATGTTAAAACCAACATATTGATGAACGCACTCTTCAA

Protein RF-3 -653 -> -53 (212AA)
MNKLSCAAIFLSFSTLPLIISYFLKKRYRKLIEKFQNADDNSYYQCIFFDYKNFECRPHILNKNECGEACSYVELLRLIKFIGSAKHSISMCMY
TMTLKQISKELVNAARRGVKVRLIMDNAMSQTTPSISNMKYFDKWSISYKIPVSMDVMVHHKFCLIDENDPELAKMFFGSLNLTMQGICKNFEA
VVLTNNSDWIKMFSQEFEDLWNHL

Comparison with *Tribolium* hypothetical protein TcasGA2 TC000031 (239AA) LOW

```
Query  1    MNKLSCAAIF-LSFSTLPLIISYFLKKRYRKLIEKFQNA---DDNSYYQCIFFDYKNFEC  56
            M+++   A F L  + LP+I++Y +K+R++  ++K ++      D+SYY CIFF  KN  C
Sbjct  1    MHRMWNRAFFALGATVLPMILNYLIKRRHKFKLKKLRDEKWDQDSSYYHCIFFTMKNVMC  60

Query  57   RPHILNKNECGEACSYVELLRLIKFIGSAKHSISMCMYTMTLKQISKELVNAARRGVKVR  116
              H      C + CS   L  L++F+GSAK+SIS+CMY +TLKQ++ EL+ A  RGVKVR
Sbjct  61   SSHFNTHTACEDNCSVTHLNTLLRFLGSAKYSISLCMYMVTLKQVTDELIKAEDRGVKVR  120

Query  117  LIMDNAMSQTTPS--------ISNMKYFDKWS--------ISYKIPVSMDVMVHHKFCLI  160
            +I D+ M +   S          N K+F  +S            + P   D ++HHK+CL+
Sbjct  121  IITDHVMYKMERSKTKLLKERYGNSKFFKYFSGKIIKIVGFEVRTPPYQDSLMHHKYCLV  180

Query  161  DENDPELAKMFFGSLNLTMQGICKNFEAVVLTNNSDWIKMFSQEFEDLW  209
            D  DP L KMF GSLNLT+QG  KNFE V +TNNS  IK +++EFE LW
Sbjct  181  DAEDPNLQKMFLGSLNLTVQGCLKNFEFVCITNNSTMIKRYNEEFESLW  229
```

Graphical representation

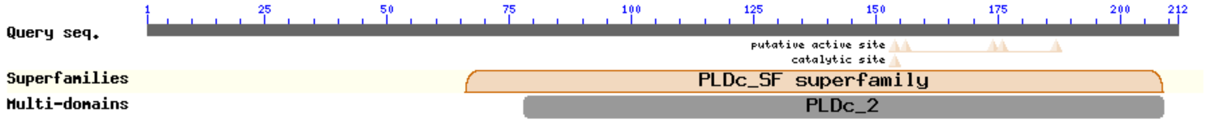

>Cb.comp42309_c1_seq15 len=2388

cDNA
CACAGATATTTGAAAGAAAACATTTTGTAAATTTTGTCATAGAATATTTTTAGGTAATTTTCAGTTACAAAATGATAAACACAAGTATTTCCAT
ATAACGGAAAATATTTTGTAAAAGTCACAAAAACTGTGAATCACGCAATAACGTTTTATTTATTAAGCCTTCATTGTCTATTTTCAAATTATCC
CAACATTGTTCAAAGTGGCTTTTAAAACCGTTTACTAAATAGAGGTTGGAGCTGAACACCATAGACTCGTAATTGTTGAAAATACTGCTGCCAG
TTAAATTCATGGAACCAGTACACAAACATCGGGAGTCACTGTTTGCATCTTTCACCATAAACTTGTGGTGCATAATAGAATCGGTCGCCGATGT
CGGCGCCACAAAAAACTGGAACTCAACTCCTTCTTTTATCAGTTTTCTAATATCTTCAGTGCTGCTTCCGGTATGGTGAAAATTATTTATTATC
CGAATACACACGCCCCTTCTTCTAGCATTTCGCAGTTCCTCGTAAATCACTTTTAAGTTTATTATCATAAAAGCAATACACAATGATTGTTCCG
CGGTTCTTATGAAATAAATAAAAGGGTCGCATAAATCTTCGATCAGGCTATATATTTCCGAGATCGGTGCGCCGTGGTGCCATCCGAATAAACC
CTTTTTGGGTCGGTAAGTGACCACGCAATTGCACTTTTTGTGAAATAAAAGTAGTTCGTCGTTTTCTTTTCGAAGTGTCCGCCACAGTTGCCAC
AACGACTTCCACGTATAGAACGGTATAACAACAACGCAAGCTGCGAACGAAACGAAAATTTTATTTATGGGAATATTTCCGATCATATTCATCC

AAATATATTATAACTACTAATGAGGTTATGTTGTTGTAAAACGTCAAACGTGCGTCAAACAGTAACAGCGTCGTCAGAGGTGTTGTATCGCAAT
TCAATTCGTTTGTGGGAGATAAGACGAAAAATATGAAAAACTGGAATTTGTATCGGGGGGCTTGAACTTTTAGATAGACAGGCCGCGGCACGAC
GGCGATGACGCGTTATCGAGACACACCAAATGAAAGCTAGTGTTATAACAAACGAGCGGTTGCGGAGTTAAACATGAGCGAACTGCAGGACAAC
GGGCGCGAGGCTTTGGTTGGGGTGATAGATGCCAGTACGAGGACTGTAAAATTTTGCGTGTTTGTTAGTCAGCATATAAAAGAAATAGCGGAGC
ATGCCATAGACCTGGAACCAATAACCCCGCAAGAGGGGTGGTCTGAGCAAGATCCTTTAAAAATACTGGCCGCAGTTAAGGGGTGTATGCAAAA
AGTAATAAACTCTTTAGGGCCCAAAGCGAATAACATTGTAACAATCGGGATCACAAATCAAAGGGAAACGACGATAGTATGGGATAAGACTACT
GGGATGCCCCTTTATAACGCTTTGGTGTGGAACGATATACGGACCGACACGACGGTGGACGTGATTTTGGCGAAGGTTCCCGAAAACAACAAGA
ACCATTTCAAGCACATTTGCGGTCTTCCCGTTTCGCCGTACTTTAGCGCCTTTAAATTAAAGTGGCTTATGCACCATGTACCCGCAGTTAGGAA
AGCGATCAAGGACAAAAAGTGCCTCTTCGGGACAGTCGACACGTGGCTGTTATGGAATCTGACAGGCGGCGTATCCGGCGGTAAACACGTGACA
GACGTCACCAACGCCTCCAGAACTTTCCTCATGAACATAGAAACGCTCCACTGGGATCCGTTCTTGTTGAACACTTTCAAGATACCGCTCGAGA
TCTTGCCCGAAATTCGGAGCAGTTCCGAAATTTACGGGCGCGTTTTCAAGGGCTGGCCCTTGGAGGACGTTCCTATATCGGGAGTGAGTACGAT
AGACCGTCGTTTATTAGATTTATGTTCCCCTCAAAGCACACATAAAAATTATTGCGTTACCAACATTTAAATTATCGTTTAGATTTTAGGGAAC
CAGCAGTCGGCGCTGATAGGGCAGAGCTGTTTCAAGGAGGGCCAGGCGAAAAACACGTACAGGAGCGGGTGTTTTTTGCTCTACAATACGGGCA
CGACGAGGGTCCAGTCGTCGCACGGATTGGTGACTACGGTCGCGTACAAATTCGGCGATTCTCCCGCCATCTATGCTTTGGAAGGTAAGTTATT
AACTAAGGTGGCAACCGGGAACGGTTGGCACGACCGTCCGCGTTGCAGGCAGTGTCGCGGTGGCAGGCGCGGCCATGAACTGGCTGAGGGACAA
CATGGGTTTCGTCAAGAACATATCCGAGGACACGGAGTCGTTGGCCAAGGAAGTGTTTAGTACCGGCGACGTGTACTTCGTGCCCGCGTTCAAG
GGGTTGTACGCGCCGTACTGGCGAAAAGACGCGAGAGG

Protein RF-3 -844 -> -119 (241AA)

MNMIGNIPINKIFVSFAACVVVIPFYTWKSLWQLWRTLRKENDELLLFHKKCNCVVTYRPKKGLFGWHHGAPISEIYSLIEDLCDPFIYFIRTA
EQSLCIAFMIINLKVIYEELRNARRRGVCIRIINNFHHTGSSTEDIRKLIKEGVEFQFFVAPTSATDSIMHHKFMVKDANSDSRCLCTGSMNLT
GSSIFNNYESMVFSSNLYLVNGFKSHFEQCWDNLKIDNEGLINKTLLRDSQFL

Comparison with *Tribolium* hypothetical protein TcasGA2_TC010319 (236AA) LOW

```
Query  14    VSFAACVVVIPFYTWKSLWQLWRTLRKENDELLLFHKKCNCVVTYRPKKGLFGWHHGAPI   73
              +F A + V   Y +K L+  +++L+++ ++  LF+++ NCVV Y  K  + GW     +
Sbjct  8     TAFTAILFVPCVYMFKKLYSTYKSLKQQYEDEELFYQRHNCVVMYS-KTHITGWPPEYKM   66

Query  74    SEIYSLIEDLCDPFIYFIRTAEQSLCIAFMIINLKVIYEELRNARRRGVCIRIINNFHHT   133
             S     ++   DP++YFI T++ S+ +A M  +LK + E L++A  RGV +R+I N+
Sbjct  67    SISTKNLDKFLDPYLYFINTSKHSIDLAVMTFSLKPLMEALQSALTRGVKVRLIVNYLSV   126

Query  134   GSSTEDIRKLIKEGVEFQFFVAPTSATDSIMHHKFMVKDANSDSRCLCTGSMNLTG-SSI   192
              +  +   +L+K G++  F++  T++  +IMH K+ VKD +     L  GSMNLTG S+I
Sbjct  127   KNQAKQYNELMKSGIKVAFYIEKTTSLSNIMHCKYSVKDYDGSKGFLFMGSMNLTGMSTI   186

Query  193   FNNYESMVFSSNLYLVNGFKSHFEQCWDNLKIDNEGLINKTLLRDSQF   240
              NNYE + F+SN+YLV  F   F+  W+ +  DNE   NKT+L DS F
Sbjct  187   LNNYEDVTFTSNIYLVETFHKSFQDSWNMIMEDNENFYNKTVLADSGF   234
```

Graphical representation

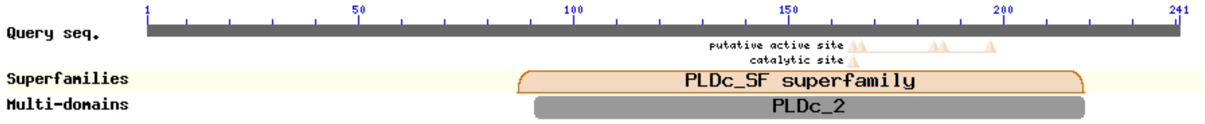

Protein methyltransferase 5 gene (dPRMT5/capsuleenn/dart5)

>Cb.comp15581_c0_seq1 len=2062

cDNA

ATTTCTTTATGGATTACCAACATCCTATAATTTTAATCTTACCACAAGTAACCACATTTTATTCTATCATTCTAACCTCTTTAGCTGAGACGAT
TTGAAACAAAATTCGTTTGGTTTCTTTTAAATTGACTTTGTTAAACGTGCTACCAATAATGGAAGATGGTGATAAAACGGGCGAAAGGCGTAAA
CAGATGTCTTGCGGTCTTTACAGCAGTTGCCCGGCTTCACTTAAAGCAGCTTTGCACTCGGCGTTTGATTATGGATTTCATTTTATAGTAACCC
AAATTACGCATCCCAATTACACAAGGGATTTAAATAAACCTGACTCTCCATTTATTATTGGCCGCACTGATCGAGTGCTTAAGGGAACTGAATG
GAATAGATTGATTGTTGGCGAACTGACTAGCAACATTGAGGTGGATAGCGAAGTTGAACATGTCAGAAGAGTAAGCAAAGATATACTAAAACAG
GAACTTGGGTTTGCAACTCATTTAGGGATACCTGCTGTCCTATTACATCTTTCCAGACCTGACAACTTGCAGTTGGCTCAAATAATTAACAGTC
ACCTTGTTCCAAACTCTTGTTTTGCAGTTTGGGTCCAGGTGCCATTAGTGCACCCTTCCAGAACAAGCAGTATTAGTGATAAGGAGAGTGATTC
TTGGGAGTGGTGGAATAATTTTCGAATTCATTGTGATTATGAAAAACGAGTAGGTGTCGTTTTAGAATTGCCAGATATAAATTCTATTCCAACA
GTGGAGGAACTAGACAGATGGATTGGTGAACCGGTAAAAGCATTGGTTCTACCAACTTCATATTTTTTAACTAACCAATATGGTAAGCCAGTAT
TATCCAAAGCACACCAGGAAATTATCAGAAAATTCATAGCAATTGATGTACAGTATATTATTCATTTAGATACGGAAGCAGACTTTTTTATGTA
TGTTAAATATATGAATTTCTTAGGAAAGAAGTTGTACAGTTGTGATATCATGGCTGAATTTGTTCAAGGCTGTGAAGATTATTTGCAAAGTCCT
TTGCAGCCTCTCACGGAACATTTAGAAACCAATGTTTATGAGGTGTTTGAAAAAGATCAAGTAAAATATGATGTTTACCAAAAAGCTGTGTATG
ATGCCTTGCAAGAATGGACTGAAGACAGAAATCCTGTCATAATGGTGGTCGGCGCTGGAAGAGGACCTTTGGTGCAGGCAGTTCTCAATGTGTC
TGTATTGCTTAATAGAGGGGTAAAATTGTATGCTATAGAGAAGAACCCTTATGCTGTCAATACCTTGGCAGAGAGAGTTAGGCGTGAATGGGGC
TCAGATAAAGTGACTTTGGTGAAAACTGATATGCGCAGTTGGAAACCACCCGAGAAAGCTGATATATTAGTGTCAGAGTTGCTTGGCTCATTTG
GTGATAATGAGCTGTCCCCCGAATGCTTAGATGGTGCACAGCAATTGTTATGTCCTCGGACAGGCGTTTCTATTCCCGCTTCCTATACATCCTT
TTTAGCGCCCCTTCAAAGTATAAAGATATATAACGAGATTCGAGCCAACAGACCTTCCGATAAAACATTACGACAAGTTTTTGAAACGCCATAT
GTGGTACACCTTGCGAATTATCAACAACTCGCTCCCGCTCAGGCTTTGTTTACGTTCGAGCATCCAAATTTTGCAGCGCGTATCGATAACAGAC
GACAAAAAAGACTTCGGTTTCCTCCCGTACGTCAAGCGTGCGTGCTGACATCTTTCGCCGGCTTCTTCGAAGCTTATCTTTACGGCAACGTCGT
ATTGTCAACAAATCCGACTACACATACTCCTGACATGGTTTCTTGGTTTCCGATTGTTTTCCCTTTAGCGGAACCCGTCCAATTGCGTGCCGGA
GACGTGGTTCAAGTTTCGTTTTGGCGTGAGGAATCCACCGATCGCGTCTGGTACGAATGGTGCTTGGAGTTACCTGTACGGTCATCCATCATGA
ACCCCAATGGACGCTCTTACCATATAAAAAAGCATTAATTAATTAGCGTTCGTCGCAAAATAAACGTTTTTTATTTTCTTAAAAAAAA

Protein RF2 455 -> 2311 (618AA)

MEDGDKTGERRKQMSCGLYSSCPASLKAALHSAFDYGFHFIVTQITHPNYTRDLNKPDSPFIIGRTDRVLKGTEWNRLIVGELTSNIEVDSEVE
HVRRVSKDILKQELGFATHLGIPAVLLHLSRPDNLQLAQIINSHLVPNSCFAVWVQVPLVHPSRTSSISDKESDSWEWWNNFRIHCDYEKRVGV
VLELPDINSIPTVEELDRWIGEPVKALVLPTSYFLTNQYGKPVLSKAHQEIIRKFIAIDVQYIIHLDTEADFFMYVKYMNFLGKKLYSCDIMAE
FVQGCEDYLQSPLQPLTEHLETNVYEVFEKDQVKYDVYQKAVYDALQEWTEDRNPVIMVVGAGRGPLVQAVLNVSVLLNRGVKLYAIEKNPYAV
NTLAERVRREWGSDKVTLVKTDMRSWKPPEKADILVSELLGSFGDNELSPECLDGAQQLLCPRTGVSIPASYTSFLAPLQSIKIYNEIRANRPS
DKTLRQVFETPYVVHLANYQQLAPAQALFTFEHPNFAARIDNRRQKRLRFPPVRQACVLTSFAGFFEAYLYGNVVLSTNPTTHTPDMVSWFPIV
FPLAEPVQLRAGDVVQVSFWREESTDRVWYEWCLELPVRSSIMNPNGRSYHIKKH

Comparison with *Tribolium* PRMT5 (624AA)

```
Query  3     DGDKTGERRKQMSCGLYSSCPASLKAALHSAFDYGFHFIVTQITHPNYTRDLNKPDSPFI  62
             DGD   E+RK+MS GL  +CP SL+ A+ SA++YG+HF+VTQITHPNY RDL     P
Sbjct  10    DGD--NEKRKRMSTGLQVNCPHSLRLAIQSAYEYGYHFLVTQITHPNYARDLLHGKPPPA  67

Query  63    IGRTDRVLKGTEWNRLIVGELTSNIEVDSEVEHVRRVSKDILKQELGFATHLGIPAVLLH  122
             IGRTDR+L+  EW R IV ELT  I VDSE+EHV+R SK +  QELGFA HLG+P +
Sbjct  68    IGRTDRILQSLEWGRYIVAELTPTINVDSEIEHVQRKSKALFLQELGFAVHLGVPVIKFS  127

Query  123   LSRPDNLQLAQIINSHLVPNSCFAVWVQVPLVHPSRTSSI--SDKESDSWEWWNNFRIHC  180
             L++  N QL ++IN  LV      + WV +P+VHPS+ S I   D++ DSWEWWN+FR +C
Sbjct  128   LTKRHNAQLGRLINEKLVNGFTSSFWVTLPMVHPSQFSPICTEDEKEDSWEWWNDFRTYC  187

Query  181   DYEKRVGVVLELPDINSIPTVEELDRWIGEPVKALVLPTSYFLTNQYGKPVLSKAHQEII  240
             +Y+K VG+VLELP+I  IP+  E++RWIGEPVKAL++PT+YF+ N +GKPVL +AHQ+II
Sbjct  188   NYDKHVGLVLELPEIAHIPSQSEVNRWIGEPVKALIIPTTYFILNNHGKPVLPRAHQDII  247

Query  241   RKFIAIDVQYIIHLDTEADFFMYVKYMNFLGKKLYSCDIMAEFVQGCEDYLQSPLQPLTE  300
             ++F+ IDVQYII  D+E D  +Y KY++FLGKKLY  D   EF+QGCED+LQ+PLQPLTE
Sbjct  248   QRFLTIDVQYIIKSDSETDLSLYTKYLHFLGKKLYVGDPNLEFIQGCEDFLQNPLQPLTE  307

Query  301   HLETNVYEVFEKDQVKYDVYQKAVYDALQEWTEDRN-PVIMVVGAGRGPLVQAVLNVSVL  359
             HLETN+YEVFEKDQ+KY  YQ A+  AL +  +D   PVIMVVGAGRGPLVQA LNVS +
Sbjct  308   HLETNIYEVFEKDQIKYTTYQNAIQKALADVPQDVALPVIMVVGAGRGPLVQAALNVSYI  367
```

```
Query  360  LNRGVKLYAIEKNPYAVNTLAERVRREWGSDKVTLVKTDMRSWKPPEKADILVSELLGSF  419
            L+R VK+YA+EKNPYA+NTL +RV  +W   +VTL+  DMR ++PPEKADILVSELLGSF
Sbjct  368  LHRKVKVYAVEKNPYAINTLIDRVNHDWNG-QVTLINEDMRVYEPPEKADILVSELLGSF  426

Query  420  GDNELSPECLDGAQQLLCPRTGVSIPASYTSFLAPLQSIKIYNEIRANRPSDKTLRQVFE  479
            GDNELSPECLDGAQ+ L  ++G+SIP SYTS+LAPLQSIKI+NEIR NRP+DKTLR  +E
Sbjct  427  GDNELSPECLDGAQRFL-KKSGISIPCSYTSYLAPLQSIKIFNEIRNNRPADKTLRTCYE  485

Query  480  TPYVVHLANYQQLAPAQALFTFEHPNFAARIDNRRQKRLRFPPVRQACVLTSFAGFFEAY  539
            TPYV+HL NY Q+APAQ LF FEHPN+   I+N R K+LRF    Q+C+LT F GFF+
Sbjct  486  TPYVIHLVNYYQIAPAQPLFKFEHPNWNDVINNERYKKLRF-NCEQSCILTGFVGFFDTV  544

Query  540  LYGNVVLSTNPTTHTPDMVSWFPIVFPLAEPVQLRAGDVVQVSFWREESTDRVWYEWCLE  599
            LY +V+LS +P THT +MVSWFPIVFPL EP+++ AG V+++SFWR E+ D+VWYEWCLE
Sbjct  545  LYKDVMLSIHPETHTREMVSWFPIVFPLMEPLKVEAGSVIEISFWRVENADKVWYEWCLE  604

Query  600  LPVRSSIMNPNGRSYHIKKH  619
             P++  +MNP GRSY IKKH
Sbjct  605  KPLKGCVMNPAGRSYFIKKH  624
```

Graphical representation

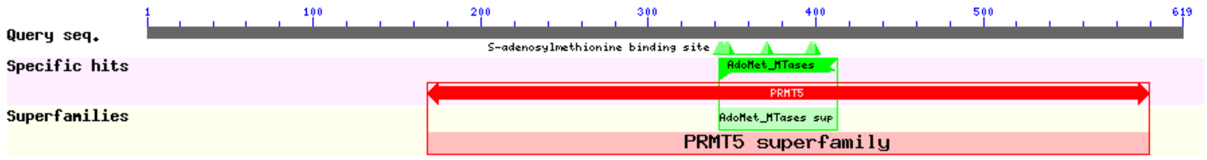

Tudor-domain containing proteins

>Cb.comp38296_c0_seq1 len=3696

cDNA

CCGAGCATAGAGGTTGATTTGCTAAATCCATTGTATCATAGATAGAATCTATCCTTGCCCACAGACGATTTCAATGGACTATTTTAACAAGTTG
TTATTCCATAGCCGATTTTTTTTTAGTGTATTTTATATAATCTGTGATAGAAAGGTTCTTATTCTTTGAATTTGTGCAGTCGATTTACGAATTT
TGTGTGGAATAGTGATAGCGAATGATAAGTGCACCAAATTTTGTTATAGGCAAAATTAAATGAACCACAATTTTTTGACAATCAGCAAGATAAT
TTTACAATGGATACATTCCGGGAAGAAGTGATCAAATGTGTCAGAGGATGTCTTATTTCTACAAAAGAAATGGTCTCGTTAAAACAGTTGAACA
ACGACTATTACACATTGATCGGTGAGCGAATACCATACCAGAAGTTAGGGTTTAGGAAATTAGAAGATTTCATTCAATCTTCAAATGAATTGGA
ACTTACAAAGAGGGGCACTGAGTATTTTGTTGGCGCTGTACCTGATAAGAAATCATCCCACATTCTTAAGCTGGTTTCGAAGCAGAAAACTAAT
TGCAAAAGAAAACCGCCCGCGCATAGAGTTCGCTTTCACTTACCACAGCAGCAAGCGCCGAGATTTACAAATGGCCGTGAGCCGCTTGCAAATT
GGCGGCCGAAATACAACGCAGCTGTGTTTAACAATTCCAAAGAGCCTACTAGGCCTCGAACTGTTGCCCATTCCCAGTTTATTAATCGCAGCTT
TAATAGGTCAAATTCGGTAGCATCCAAGGTGGTAGTACCAGAATATTCTACTTATAATCTTCAAATTAATACTCCAATTTCCACCTCGTATGAT
AGAGACACTAAACAACAGACAACTAAAAAGGATATTCAGGGTAGGTTGGGGATATGCAAAGTAAAAGAATTACACCCTTCCAGTGGTGCTGAAC
AAAACGGTAATCAGCATAGAAAACAATCATTGAGCGATAGCATACCTAGCGCTCCAACTACTCCCACATTTGAAGAACCATCCCAACAAGTGGG
TTCTGCCAGACAAAGAATAAGCAGAATGATGTCGGAAATAAATTTGGGAAGGGATAGCGGTAATAGTAGTCCGGTAAGTGATACTTCTCCCCCA
TCCCCATGCAAGACACCGGAATTTATTCGCACGGATGACCCACTTGCCGATCTCGAAGCGTTCGCTGTATTATATCATCTTGGCGAAGTCGTGA
TATCGATGCGAGAAGTGAAAATCAAACGAAATCAGATTATGTTCAGCTGCAAAATTAAGATTGGAAAGCACACTTACAGCAGTTACCCGAATGA
CTTTAGGAACAAAATGGATGCTCAAAATTTTTGCTGCGAAGAAGCGCTGAGTGACCTGCTGCCCAAATATCACAGGAGAAAGTCATTGCTTATA
TCGAGCGAGGTGGATGTATTGGAACGGATACCGCCTATGCTTGAAAAACACAACAATGGTATCTGGGCCAAGCAACTGAAGCTGGACTATGCTG
ATCGATTTAATGAGCAGTTACCAGACGATTGGTTGCAGATAGTCGACTCAAGTTCATTGGTACAGATTGAGGCTGTCTCGGACGACCATATTCT
GCAGTACTGCAAATCTGGTGTTAAGGGACAGAGGCGGGGCGTAGGCATGACAATATACAACGTATCAGTGCCGTCCAAAACCGTGGACTTTGGC
GAGGACGGAAAGTTGATCGCGCAAATAACGTACGTCATGTCCGCCAACGAGATTTGGTGTCATCAAATTCAAACCAGTGAATATGAGCAGTATT
TAGAAATGATGAGCAGAATGGAGCTGTACTACAACAGCCGCGAAGACAATTTAAAAGCTTTCAACATCGTTCAGTCCGGTTATTATGTCGCCAA
TGTGGACGGCTCCTATTTTAGGGTCAGAGCAGTCAGCGTCTCCGACAATGAAGTAAACTGTTTCTGTATTGACTACGGGGACGAGGTGACGGCC
CGCAAGACTGATATTTTCGAGTTAAAAAGGGAATATGCGACCACGCAGGCGCAGGCGTTTGTATGTCGTCTAGCTGGGCTAGAAGAATTATACG
AGATTTCTGCAAATTCGGAACTTTTGGCCGATCTTGTTACGACGCAGGTTATTTTAGAAGTGGCAGAGGCATGTTCAGATGGTAGCAGTGAAAA
TGTTCTTCCAGTGGTCATGTATGAACTTGAAACGGGTAATTCCATCAATGGGGATTTGATACCAAAATTGACCATAGAGTCTGCTTTGCCAATC
CTCCACAAGGAAGGGATAACTGAAGTCTATGTCTCTCATATCGAGACCCACGACGAAATTTATGTGCAAACTAGAAATTATGGTTTTGAACATT
TTAACAAAATAAGGGAATCTCTTGAAAGTGATATTAGCACTCGGCTTGGAGATAAGCTGGAACCTGTCACTCAAGCCAACAGCAATGACAAGCT
TTATTTTGCCAGGTCCAAGTCCGACGGACACTGGTACCGGATTAAACTGATCGATTGGTCGCCTCAAGGCGATTTTGCAAAGATCCATTACATT
GATCGAGGGGAGGCAGATATTATCCAAGTAGCTGCCGAGAAGTTGTACGCGCTGGATGGGCTCAGTGACGTTTTGTGCCAGTATCCACCGCAAG
CTGTCCGGGTTCAAATGGCGTTAGAGGAGGTTCCAGACGATTTTGCAGAACTCGCTGGGAGGCACATGCCTCCGGAGCAGGCCATTCTTCTGAA
GGTGCTTGGGGAAGACGAGGTTCCTCGTGTTGAGTTCTTCAAAAGGAATGAGGACGGGGGACTGTTTTGCGTCAACAAGTCCATCGCTTTGGAT
ATGGAACTAAGGAAAGAGGATTCCAAATCAAAACTGAAAATAACTCAGTTTAAATCGAAGTGCGTACCTTCTGCCGGTAAATTGGCAGCTCCGG
CGCTAACCCAAGTGGGTGAATTGTTTGAGGTGCACATTCCGATCGCTGTGAACCCGTATAACTTCTTCATCCAGCCTTTAGCCTCTAAGGGTCA
GCTTGATGAAATGATGGTGAAATTGCAAGCGAAGTACAATAACCTTAAATGTGGGAGACTGTCTGCGGAAGAGATTGTACCTGGACAAATATAC
GCCTCCAAGCATGAGGATGGCGTTTGGTACAGAACGAGCGTCATCAAAGTGATTCATGCGCGCTCAATATCTGTATTTTTCTGTGATTTTGGTT
ACTATAGGAATCTCGTTGTCGAGCAACTTGTACTTTTGGATGAGGAATTTTTAGAATTGCCTTACCAAGCGTTGAAAGCGAAATTATCAAATAT
AAAACCAAAGCAAAATAAGTGGACAATGGAAGACTGCGACGCATTCAAAAAGCTTGTGGAGAAGAAAGATTTATATTCGTTATTAATCAAAATT
GAAAAAGACGTTTTGTACGACAGCGATTTTGTGTTAGAACTAGTTCTCATCGATACTAAAACAGATGAGGATGTTTATATCGATAAGGAGCTTG
TGAGGCAAAATATAGCCATTAAAGGTTAGAGTATATTTTTTTATAAATATATTAATGGTTAGAGCATGTTTTTTTTTAAATAAATATTCCAAT
AGTTTATTGTTTTCTTCATATGTTGGTTTTCTTATTAACCCATGAGACACATGAAAAATATTTATATTTTTGTTGAAATATAGTCCTAAGGTAG
CAAGTAATTTTTTAAAAGTTCGATTCTGAT

Protein RF3 289 -> 3507 (1072AA)

MDTFREEVIKCVRGCLISTKEMVSLKQLNNDYYTLIGERIPYQKLGFRKLEDFIQSSNELELTKRGTEYFVGAVPDKKSSHILKLVSKQKTNCK
RKPPAHRVRFHLPQQQAPRFTNGREPLANWRPKYNAAVFNNSKEPTRPRTVAHSQFINRSFNRSNSVASKVVVPEYSTYNLQINTPISTSYDRD
TKQQTTKKDIQGRLGICKVKELHPSSGAEQNGNQHRKQSLSDSIPSAPTTPTFEEPSQQVGSARQRISRMMSEINLGRDSGNSSPVSDTSPPSP
CKTPEFIRTDDPLADLEAFAVLYHLGEVVISMREVKIKRNQIMFSCKIKIGKHTYSSYPNDFRNKMDAQNFCCEEALSDLLPKYHRRKSLLISS
EVDVLERIPPMLEKHNNGIWAKQLKLDYADRFNEQLPDDWLQIVDSSSLVQIEAVSDDHILQYCKSGVKGQRRGVGMTIYNVSVPSKTVDFGED
GKLIAQITYVMSANEIWCHQIQTSEYEQYLEMMSRMELYYNSREDNLKAFNIVQSGYYVANVDGSYFRVRAVSVSDNEVNCFCIDYGDEVTARK
TDIFELKREYATTQAQAFVCRLAGLEELYEISANSELLADLVTTQVILEVAEACSDGSSENVLPVVMYELETGNSINGDLIPKLTIESALPILH
KEGITEVYVSHIETHDEIYVQTRNYGFEHFNKIRESLESDISTRLGDKLEPVTQANSNDKLYFARSKSDGHWYRIKLIDWSPQGDFAKIHYIDR
GEADIIQVAAEKLYALDGLSDVLCQYPPQAVRVQMALEEVPDDFAELAGRHMPPEQAILLKVLGEDEVPRVEFFKRNEDGGLFCVNKSIALDME
LRKEDSKSKLKITQFKSKCVPSAGKLAAPALTQVGELFEVHIPIAVNPYNFFIQPLASKGQLDEMMVKLQAKYNNLKCGRLSAEEIVPGQIYAS
KHEDGVWYRTSVIKVIHARSISVFFCDFGYYRNLVVEQLVLLDEEFLELPYQALKAKLSNIKPKQNKWTMEDCDAFKKLVEKKDLYSLLIKIEK
DVLYDSDFVLELVLIDTKTDEDVYIDKELVRQNIAIKG

Comparison with *Tribolium* similar to CG8920 CG8920-PB (1045AA)

```
Query  1     MDTFREEVIKCVRGCLISTKEMVSLKQLNNDYYTLIGERIPYQKLGFRKLEDFIQSSNEL  60
             M+ FR E++  +R CLISTK  V+L+QL +DY TL+GERIPY KLG + LE FI S   +
Sbjct  1     MEEFRNEIVSRIRSCLISTKGQVTLRQLEDDYRTLLGERIPYAKLGHKTLESFIISIPTI  60

Query  61    ELTKRGT-EYFVGAVPDKKSSHILKLVSKQKTNCKRKPPAHRVRFHLPQQQAPRFTNGR-  118
               ++  + E  V A   +K++HI  +V KQK+      P   VR       AP+
Sbjct  61    ITSRSPSGEILVDAQVSEKTAHISSMVRKQKS-----VPKKHVRI------APKLNRAMA  109

Query  119   EP---LANWRPKYNAAVFNNSKEPTRPRTVAHSQFINRSFNRSNSVASKVVVPEYSTYNL  175
             +P    A WRPK    +         +   V+H+QF+N                YS Y
Sbjct  110   QPPPNAAKWRPKQKPLMRKTYGNTPKLAAVSHNQFVNN----------------YSGYGK  153

Query  176   QINTPISTSYDRDTKQQTTKKDIQGRLGICKVKELHPSSGAEQNGNQHRKQSLSDSIPSA  235
             +   P           ++      ++ +            ++G E+  N  R +
Sbjct  154   K-TVPSKVVVVERKEEVKRNNSVEVQRN---------NNGLEEVKNNSRLEK--------  195

Query  236   PTTPTFEEPSQQVGSARQRISRMMSEINLGRDSGNSSPVSDTSPPSPCKTPEFIRTDDPL  295
                   +E +    S  +RI+++M ++N+  DSG SSP ++ +      + +F++T DP+
Sbjct  196   ------DEENSMFSSTLKRITQVMKKVNVETDSGTSSPTTEYAAGYKLSS-DFLKTGDPI  248

Query  296   ADLEAFAVLYHLGEVVISMREVKIKRNQI-MFSCKIKIGKHTYSSYPNDFRNKMDAQNFC  354
             +DL   F    + LG+V +    E K+K++++    CK+ +G+H YSSYP DF ++  A+
Sbjct  249   SDLRNFVAYHKLGKVDVKFTETKLKKSKVPQCHCKVTVGQHKYSSYPEDFYDRDAAERHA  308

Query  355   CEEALSDLLPKYHRRKSLLISSEVDVLERIPPMLEKHNNGIWAKQLKLDYADRFNEQLPD  414
              ++AL DL+ KY RR+SLL+SS  D++ERIPPMLEKHNN +W  Q++ DY DRFNEQLP
Sbjct  309   SQKALDDLMQKYSRRRSLLLSSNDDIIERIPPMLEKHNNAVWMWQIEADYRDRFNEQLPP  368

Query  415   DWLQIVDSSSLVQIEAVSDDHILQYCKSG---VKGQRRGVGMTIYNVSVPSKTVDFGEDG  471
             DWLQ++D+S  V IE      +L++C       KG++  + + + +VSVP  TVDFG+
Sbjct  369   DWLQVIDNSPFVSIEKCHGGCVLKHCNPDDVLQKGKKLDISLNVGDVSVPCNTVDFGDSN  428

Query  472   KLIAQITYVMSANEIWCHQIQTSEYEQYLEMMSRMELYYNSREDNLKAFNIVQSGYYVAN  531
             +L A +T   S NEIWC    T EYE+++EM   ME YY S +  LKA  I      YV
Sbjct  429   RLYAVVTVAHSVNEIWCQHCGTPEYEKFVEMTQNMESYYESYKTELKAKMINAGSCYVIQ  488

Query  532   VDGSYFRVRAVSVSDNE-VNCFCIDYGDEVTARKTDIFELKREYATTQAQAFVCRLAGLE  590
              +G + RVR +   +N  V+CF IDYG+E++    +++ LKR++AT QAQAFVCRL GLE
Sbjct  489   NEGLWIRVRVLKTPENGYVDCFLIDYGEELSISIDNVYLLKRQFATEQAQAFVCRLDGLE  548

Query  591   ELYEISANSELLADLVTTQVILEVAEACSDGSSENVLPVVMYELETGNSINGDLIPKLTI  650
             + YE S +SE+LA LV  + +LE+       + +  +PVVMY++ +G SIN +LI  LTI
Sbjct  549   DFYEASIDSEILAGLVGKEYVLEIVTDDISDTGDVTIPVVMYDVTSGASINEELISSLTI  608

Query  651   ESALPILHKEGITEVYVSHIETHDEIYVQTRNYGFEHFNKIRESLESDISTRLGDKLEPV  710
             ESA+P+L K+ ITEVYVS+IE + ++YVQ R  G+    +  ++L  +I+++   +L
Sbjct  609   ESAIPVLEKDSITEVYVSNIEPNGDVYVQVRTVGYFSLMEDLKNLTDNITSKNPSQLSTT  668

Query  711   T--QANSNDKLYFARSKSDGHWYRIKLIDWSPQGDFAKIHYIDRGEADIIQVAAEKLYAL  768
             T  + NS  K+Y    K    W R  ++DWSP+GD A++++ID+G A ++ V  EK+Y L
Sbjct  669   TPTKDNSTGKIYLVMCKMTQQWLRATIVDWSPKGDLAQVYFIDQGNAQVVNVTNEKMYEL  728

Query  769   DGLSDVLCQYPPQAVRVQMALEEVPDDFAELAGRHMPPEQAILLKVLGEDE--VPRVEFF  826
             D L  VL QYP QA++V+  +E++P DF + A + +P ++ +LLK++  D   V  VEFF
Sbjct  729   DKLDSVLSQYPGQAIKVRFMIEKIPSDFVQKAEKLLPKDRPVLLKIISYDNENVAWVEFF  788

Query  827   KRNEDGGLFCVNKSIALDMELRKE---------DSKSKLKITQFK--SKCVPSAGKLAAP  875
             KR  DG L  +NKSI+++ EL++           + K  L + QF   SK VPS G L  P
Sbjct  789   KRTTDGVLVFINKSISVEAELQQNVDVSNNNETNRKRLLNLVQFNEASKNVPSGGSLRKP  848

Query  876   ALTQVGELFEVHIPIAVNPYNFFIQPLASKGQLDEMMVKLQAKYNNLKCGRLSAEEIVPG  935
              L ++G+ F V+IP AVNP+NFF+QPL S  +L  +M ++Q  Y +     +  EEI+PG
Sbjct  849   DLPKMGDYFNVNIPFAVNPWNFFVQPLDSFARLKALMNEMQEHYKDTHFSPMPLEEIIPG  908

Query  936   QIYASKHEDGVWYRTSVIKVIHARSISVFFCDFGYYRNLVVEQLVLLDEEFLELPYQALK  995
             +IYASKHEDG WYRT+V+KVIH  SISVF+CDFGYY NL ++QLV LD +++ LPYQALK
Sbjct  909   KIYASKHEDGQWYRTNVLKVIHEGSISVFYCDFGYYTNLTLDQLVPLDAKYMGLPYQALK  968

Query  996   AKLSNIKPKQNKWTMEDCDAFKKLVEKKDLYSLLIKIEKDVLYDSDFVLELVLIDTKTDE  1055
             AK+S IKP +NKWTMEDC++FK L+ KK    ++  I++D   + SD +LE++LIDT ++E
Sbjct  969   AKISGIKPIKNKWTMEDCESFKDLILKKQFVGVITNIDRDEFHKSDLILEVLLIDTSSEE  1028

Query  1056  DVYIDKELVRQNIA  1069
             DV I + L+++ IA
Sbjct  1029  DVNIKEVLIQKGIA  1042
```

Graphical representation

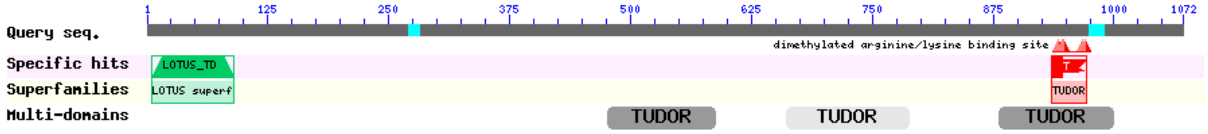

<u>Antiviral RNAi in *Cylas brunneus*</u>

<u>Ars2</u>

>Cb.comp43566_c0_seq35 len=7361

<u>cDNA</u>

AGGTAATTATATGCGGATTAGGGCGAGCTGCCGTACTTTGTGTCACGATTAGTTTCAATTACTTTCTATGCAAATGGCAACCTACATTTTTGTT
GGTTAGTCCTACATTTTCTAAAGCTATATTTAATGACATAACCGCATTAGTTCGCACTTAATCGCCTAGCGTTTGTTTTTAAAACTCCATTAAA
ACTGAAGGGTTTACGCTAGAGCCTAATGACGAGAGCGGAGCCAACGTAGACGCGGCCAAAATGTGGAGGGAGAATAGGGAAGAGTTTAATCAGA
TAGCAGAACAATTGGTTAGGAAAACGCTGGGCATACCAACATAAAATTTATAATTCTTTTATTTATACTTTTGGTGGTGTACAAAGGGGGGAAA
AAATAATAAACGGTTGTTCTGTTTTATCCTTTATATTGGCAAGTATGTCGAAACACAAATAAATAATCCCGGGGAAAAAAATTCCTTAAAATTAC
TCACAACCTACAGAAAATTATGCACCGGCAACACATACGCAACCATTTTCATCCCCCCTCCTAGAGACTATTGTGCGTATTATAGTACATACAG
GGTACTTACTCGTTTCGAAGTGACGATCTGTCAAAGCGACCACCTTATCACGTCTAGTTTGAAGAGGAAAAAACGCTCTACGCAAAACTTGGGC
ATAAACGTAAAGTCGAGTGTCCAAAAATTAAATAATTTGAAGAAAATCTATTTTTTGGTTATAAAAATGCCTATGTTGTGTATATTTCCGCTAA
AGACATTTTAAGGGAAGAAATCACCCTGTATCTGTAAGACCCTACAGCAACTAAGATCACATATAAAGACTTTGGTTTCCAAATTGAAAAAAAA
AGGTACACGTCGAACACCTAAACTGCGTCACAGTATACTGCGACCTCGCTATTATTGCACAAACCAAAAAGAATAAGGTATTCTATTCGTTCGC
CGCATAGCCGGCTAATCCTTATTATCCACTTTTAAAAAGCGGAAGAGCGATCATCTCACGGCCGACGTCGACTTTTTGGACGACAAGAGAATCA
AGACACCCTGCGTTAAACGGGATCCTACCCCATGGTATAACCTTAAGATTGTCCGCGCGCGGCGTTGTTCGCGACGCCGCCGTTACTATTACTA
TTGTTATTCAGTTCGACGTAGTCATTTACAGGTACAATGGGATAACTAACTATTCGGACGTTGTTCGGGCGGGTGGAGGCAAAGTCCGACGGCG
TTCCGTCGGACGTGGCCGTCACCTCTTCGTTACTCGCTTCCGGAACATCCACGAAGTGCTCCGAATAATAATGCGGGGTATTTAGGTAATCGAT
CATGCGTCGTGGCAGCGGCAGGTCCGGAATCAAATCCCTCCGGACGTTTTTGTGGATTACGAACCTGCACATGTGCTGCAAACTTTGCACCTGT
TTGAACCGCGACACGGGATGCAGCAGTTGCACCCGCACGGGACCTATAACGGGCCTGCGGTGCAGGAAGAATAAATACCGGCCGCTCCTGGAAT
GTTCTACCGCGTTTTCGATGAACTCGACGATCGTCTGCGACTTGAACTTTGTACAGCTGCCGAAACTGAAATTACCCTGATCGTGTTCGATTCG
GACGTGCCGCACGCAGTTGTTCAGCTTAAAGGTTAGTGAAAATATGTAATGGTCGTCGCTACTGTCCCGAACGATGAACGAACCGTCCGGCTCG
TTGGATAGTATCTTTTCCGCGGCCTCGCTCGATATCGGGCCCCAATACCAACCGTAATCTTTAACTCTCTGTATGCTGGTGGCAAAATCGAGGG
CTTGTTCTTCGGGGGTCGACAACTCGTCCTCGATTTTCTCGTCTTTGTGATTAGGCAGCGGAGGCAGAGCCCGGCCGCTCAGCGTGTTGCGCGA
ATCGTCGCCGACTTGCGCTTGATTTACGTCGTAACCCTTTTCGTTGTCAGTCTTCGCGTCGTTCGACAGGAACCGTCGAAAGCGGAAAATATTG
CACAGTGCCTGCTTGAAACCGAACAGCCTGCGTTTTTTCTCCTCCTTCTTGGACGCGGATAGCAGCGTTTTCGGATTGTCACAGCTGTCTTTTA
CCTTTTTGTTTTTGATGTTGATCAGGAAGGTGGCCTTTTTGCGATTGGCCGGCTGCAGCGACGCTTTGTCCTGAGTGTTCAAATTGCATCCAAC
GCTCCTGGTGTGGTATTTATTGTTGGGAGCGGCCAGCGCAGAGTTAGCTTTGGGCCTGTACGGGTTCTCCTTGGAATCGGACGATTCCGATGAG
CTGGAATACAGCGTCTGCAGATAGTGCCTCACTTCCTGCAGAGTCATGTGTATGGGTTCCCCGCTGTACGGGGCCGAAGTGCCCGCTAAGCTGT
GACGTTTTGTTTCCGATTGGAAATACCAGTGCGACCGTTCTGATTTGGCGACGCTTCTCCGCCCGCCACCGATCTCACGCACTTGCATCGGTCT
CCTCGGCTCAACCGGCGCCTGTCCTCTCGTTTCCGCGTCCCTACATCCGGCCGACGTGTTCCTACGTCTCTTCTTGGCCCCGCCGACGACGACG
ACGCTCCTTTTCGGCTTAAGCGGCGGCCTGACCACATCTACCGGCACTGCATACACGTCGCTGTCGAAAGGTACATTGTAGAGGTCGCTGATCT
GGTTAGACGAGAGGGTGTCGTGCGAAACGGCTTGCGTTATGAACGTCGAGTCGTCCACTTTGCATGTGGTGGGAAGGGAGAGGGAATTGTGGGA
GGAGGACAGGCGTGACGAACCGCCGAGCGAGCTGCGCGAATTGGGTCGCGTGGTGTGGTGGTGGGCGTGGCAAGGGCTAAAAGGAACGAATTCG
GACGCGAAATAAGTGGGACGCGCGGATAAGTGACTAGTACGATTAGGCGTGGTGCGGGAATTGAGAACGGGCGACTGAAACGGCGAACGGCCGC
GGTCTGTCAATTTTCGAGGCTGCGCTTCTCTGAAATCCAACATCGCGCGCATAACTATTTGCAACGACAGCTTGTTGCAGAATAACTCTATCAA
AGGATACTTGGGGTTATCCTGGAACTGTAGCGGCGGTTCTATGGGCGAAGTCGACTGCTTAACTTCGGTCCAGTGTGGGCCCCTTTTTCAAAGTG
TTAAAATTTTCGCCGCACATGATGTCGGACATGCCGTCCAGCGAATCATCTTGGGACAAGGATTTCTCCTCGCAGTCGCTGTCCTCGAAGTGGA
AGTGTCTTTTATCGCTGCGACTCTCGAAACTGGTGCTCCAACTCGACGCCGAGCCGTTCGTTAACAATAAACCGCTGTCGTCGCTCAGAATAAA
AGCCTGCGACCAACTGGAGCTCGACGGCGGCGATTCCATTTCTTTTTACGCTTGTTTATGCATTTTCTAGCGCCATTTAACCACACTTTCCGAT
TACGGTCCGTAAACGATTTAATTCCGAGCGTTAGACTTACGGTAAAGCGCTAATTTAAGCGTTCGCATCTTGAATTAGCTCGTTAATTAATAAA
AACGGCAAAAATTTAATGAGCCAAACGCATATATGCGAACAACGAGAATTCTAACCAGAGAACACATGTGCCACAGAACCCAAATAATGTCTAT
GTAATGAACTATTGACCTTAATCGCTTACCGTGGGTTCACGTTATCTCGTTAAGATTGATCTGATGTAATAACTCAAAAAATATAAATGAGTAA
TAAATGGCAATCGCTACTCGACCCTGAACTTTTCGTTTGATCTCATCGTCAAATTCTGTTATGACGAGTTTTAATACTTTTTTCTCCTAATCTA
TGGTTTGAAAGTTAACAGCTGTAAAGTGACGTATAAGATCGAGTCCGCGTTTAGTTTAGCTTTTTGTGTATTAAAAATGTCGGAGTCGGACCAA
ACGGAAGAAGTGTCTTCGGAAAAGATAGAATTCAAGCCGAAGAAGCGCAAGAACTTGCGACAGCGAGTAAAACTTGAGGAAAGCGACGACGAAG
AGATACAACGAGTGAACTGCAAGCTGGAAGAAATGAAAGAGGTGCAACGCTTGCGGAAGAGACCTAATGGCGTCAGCGTCATCGGTCTGGCTCT
GGGCACGAAAGTGTCCGCAGAAGAGGAAGTAGTTGCCAAGGACCCGTTCAAAGTGCAGGCGGGCGGCATGGTGAACATGCAAGCATTGAAACTA
GGCAAGGTCAAACAAGTGGACGACGCGTACGATACCGGTATCGGTACCCAGTTTTCCGTGGAGACCAATAAGCGAGACGAGGACGAGGAGATGA
TGAAGTTCATCGAAGAGGAACTATCAAAGCGGAAAGGCAAAATTGAACCGCCCGCGCCCCGCGACCTTTACTAAAAACAAAAGCACATACTTGAG
CCCCGAAGAGGCAGCATTGCAGGCGGTTCCGGAGCATTTGCGAGAATCGTCAACGAAACGATCCGAAGAGATGTTATCGAACCAAATGCTAAGC
GGCATACCCGAGGTCGACTTGGGTATAGAGGCGAAGATTAAGAACATCGAGGCGACGGAGGAGGCCAAACTGAGGTTACTCTGGGAGAAACAGA
ACAAGAAGGACGGCCCGTCGCAGTTTGTCCCGACCAACATGGCCGTGAATTTCGTCCAGCACAACCGATTTAACGTGGACCACTCGGAGATCGC
GAAAAAGCGGGCCAAAGTAGTGGACGCGGAAAAGCCGAAAAAGAAGACTGAGAAGGCGACCGACGATTACCACTTTGAAAAGTTCAAAAAACAG
TTCCGGAGGTAGTGCGGGGAGGGGTTTATTTTGTAGCCGGAAATAAGGCGCCACACGTACCGAGGTATGTTTTAGATATCCGTCAGCGCTTAAT
CAAACCTGTACTGTACGCGTTGCGCAATCGCGTTCGCACTGTACCGGCGAGTATGAAAAATGGAGGGGGGAATAAAATCCGATCGTTAATCCGT
TTTGGTATTATTTATTTACGTTTGTTGACACGGACAGGGCGTAGCGAAAAAGCTACACGGCCGATAAAGACTCCGAGATAAACCATAAAAAAAG
AGCCACGTTTATTTCAAACGTACCCACAAACAAACGCATATTATTCCACAATTATTATTAAGTTAATCATACAAACTCTTCCGGTTCTCGCGGC
GCGTCCAAGTCCCGGTAGTGGATGATCGGTCGTCCAAAGTCGCCGGCCGGCCCGCCCCTGGCCCTGGGCGCGTATCCCCGCGACCTCGGCGCGA
AGCCCTGATTGTAATAGGGCTGCGGCCTGCCGAATCCGCCGCCGTAACCGCCACCGCCGCCGCCCCCGTAACCCGGATGGCTGTACGCCTGCGG

TTCGTCCCGTTTCTGCTGCGGGGCCTCAGCCAGCATCGGCCGTTTCGGGTCTTTGAGGTAATTGTTGAAAAACTCGACCTCCTTTTTCACCTCT
TCGATTTTTTCCGCGTGCTTATTGAAAATGTGCTTCCTCACAAAGTCGGGCCCCTTGAACTTTTTCCCGGAAAGCGGGCACAGCCACTTGTCCT
TGGCCAATTCGCGAGTGTTCGCCTGCACGAACTTGTCGACCTCCGAATCGACGTCCTTCAAGGCCATCGCTATCAGTTTCGTCTCGTTCTTGTC
TTTCTCCTCTTTGGGTTTCTCCGGCAAAAAGTTCGACATTTTCGCTTCGACCGGCGGCCCGATGAACAGCGTCATGTCGGTCTTGGAGGCGGGA
GGCGGTCCCCTGGCGTGCAGAATACCGCAACGGTTCGGCATTTCGTCCTCGTTCGGGTACTCGCAATGGTTGTAGTAGTCTACGGAGTGGACGA
CGCGCAGGTACAAGATAATCCTGTCCAGCACTCGGATCAGATTATCGTCTCGATCGACCGTCGACACCGCTTCCTGGTTTTCCGTCGACGGTTC
CAACCCCAACAGCTCCTCCTCTTCCGCGCTTGCCTCTTCGATAAGGTAGTCGGTAATGTTGTGCAACACGGGGTTGTTCGAAACGAGCCCGAAA
CTCTGGTGTGCTTTTTCTTTCCTTTCGCCATCGTCGGGCCACAAGCCGGCCTTGGCGTCCAAGTGCAAGGTTACCCTCGCCGCTATCCTAATGT
CAGACCTTACCACCTGCTTGTGCGCCATGATACCGTTAACGGGCCTGATCCGCCGACTCAAATCCCTATTGACGATGGCGCCCAACTCGCACTC
CCTCAGCCTTATGTTGTTCAAATTCCAACAGATCTCCTTGATGTTAGCCTCCCGTTTAAAAGTAACCCATCCGCGACGCAACCACCTCCGCTCC
GGCTGCGGGTCCGCCAGAGCGACCCTTAGAAACCCTTCGTAACGCGAGCACACCGCCTCCACCTCCTGCTTGGTGATGGTGGGCGCCAGATTCC
TCAAAAATATCGACGTAGTCTTGTGTAACGCCTTGGGTTTCTCCGGCTTGCTGTCGTCCGATATCGAATGCACCTCCTCGTCCTTATCCTTGTC
TTCCTTCTCTTTCTCTACCTCTTCTTCTTTGTTCTCGTTATTCTCTTTCGGTTTGTTCGCTTCTTCGTCGTCGCTATCGCTACTGGAACTCGAC
GACGAGCTACTTCGGCTGCTGCCCGAGTAGGATCGCTTCCGTTTCTTGCCGTTTTCTTTCTTCTCCGCCTTTTCTTTACTCTCCTTAGATTCTG
TCGTCTCCGTTTCGTTATCAGCGCCTTTCTCCGGCTCTTTCATTTCCGCATCCCCGTTGGTCGCCTCTTTAAGAGTGTCTTCCTCCTCCCCGAG
CTTTTCCTGCTCTTCCACCCCTTTGTCTTCATTTCCTTTATCGTCATTGTCTTCGTTCTTCTGTACCTCTTCGATCACGTCGTCCGGCGCTTCG
CCGTCTTCTTTCGCGTCGCCTTCGACTTGTCCCGCCTCCTCGGCTTGGTCGGGGCCGCTTTCGTCCCCCTTCTTTTGTTCCACCTCCTTTTCGA
CCTTTATTTTCGCCTCGATTTCGCCGTCCTCTTTTTGATTGGCCACCTCCGCTTGTCGGGCTTCCCTCGCCTTTTCCGCCGCCGCCTCTTTTTC
TTTTTGCTCGCGATACTTTTGTTCCATCTCCTCGTACTCCTGTTCCAAGGCGGCGAGGTCGTCGTCGGTACCGCCCTCGAGTCTGATGACGACC
GTGTCGAGCAATTTGAGGAGGTCGGTGGTGCGCGCGCAGTCCACCGTCGTACTCTCGATCTTGCCGTTATTTAGGAGATCTATGAACACCTCGA
GCCGCCTCTTGAGCGCGGCCGCCTGTTCCTGCTTACGCCTGATCGAATCTTCGGGGTGGTACTTCAACCGGAACCTACGAACCGATCCCGAAAC
GCGCAACGTTAGATACCGTCGTCGGGATGTCGCCTGCGTCTCACTCTTTGTGATATTCGCAATTAGTGTGACAAACCCATTCACGCAACACGCG
CGACCCGTGGATAAAAAAGAAAGGAATAT

Protein RF -1: -6983->-5049 (644AA)

MESPPSSSSWSQAFILSDDSGLLLTNGSASSWSTSFESRSDKRHFHFEDSDCEEKSLSQDDSLDGMSDIMCGENFNTLKKGPHWTEVKQSTSPI
EPPLQFQDNPKYPLIELFCNKLSLQIVMRAMLDFREAQPRKLTDRGRSPFQSPVLNSRTTPNRTSHLSARPTYFASEFVPFSPCHAHHHTTRPN
SRSSLGGSSRLSSSHNSLSLPTTCKVDDSTFITQAVSHDTLSSNQISDLYNVPFDSDVYAVPVDVVRPPLKPKRSVVVVGGAKKRRRNTSAGCR
DAETRGQAPVEPRRPMQVREIGGGRRSVAKSERSHWYFQSETKRHSLAGTSAPYSGEPIHMTLQEVRHYLQTLYSSSSESSDSKENPYRPKANS
ALAAPNNKYHTRSVGCNLNTQDKASLQPANRKKATFLINIKNKKVKDSCDNPKTLLSASKKEEKKRRLFGFKQALCNIFRFRRFLSNDAKTDNE
KGYDVNQAQVGDDSRNTLSGRALPPLPNHKDEKIEDELSTPEEQALDFATSIQRVKDYGWYWGPISSEAAEKILSNEPDGSFIVRDSSDDHYIF
SLTFKLNNCVRHVRIEHDQGNFSFGSCTKFKSQTIVEFIENAVEHSRSGRYLFFLHRRPVIGPVRVQLLHPVSRFKQVQSLQHMCRFVIHKNVR
RDLIPDLPLPRRMIDYLNTPHYYSEHFVDVPEASNEEVTATSDGTPSDFASTRPNNVRIVSYPIVPVNDYVELNNNSNSNGGVANNAARGQS

Comparison with *Tribolium* hypothetical protein TcasGA2_TC003562 (819AA)

```
Query  203  EEVHSISDDSKPEKPKALHKTTSIFLRNLAPTITKQEVEAVCSRYEGFLRVALADPQPER  262
            E + ++ DD KPEKPK+LHKTTSIFLRNLAPTITKQEVEAVC RYEGFLRVALADPQPER
Sbjct  388  EHIETVEDD-KPEKPKSLHKTTSIFLRNLAPTITKQEVEAVCGRYEGFLRVALADPQPER  446

Query  263  RWLRRGWVTFKREANIKEICWNLNNIRLRECELGAIVNRDLSRRIRPVNGIMAHKQVVRS  322
            RWLRRGWVTFKR+ANIKEICWNLNNIRLR+CELGAIVNRDLSRRIRPVNGI AHKQVVRS
Sbjct  447  RWLRRGWVTFKRDANIKEICWNLNNIRLRDCELGAIVNRDLSRRIRPVNGITAHKQVVRS  506

Query  323  DIRIAARVTLHLDAKAGLWPDDGERKEKAHQSFGLVSNNPVLHNITDYLIEEASAEEEEL  382
            DIRI+A+V LHLD K GLW DD E+K+K  Q+FGLVSNNPVLHNITDYLIEEASAEEEEL
Sbjct  507  DIRISAKVALHLDNKVGLWLDD-EKKDKPQQTFGLVSNNPVLHNITDYLIEEASAEEEEL  565

Query  383  LGLEPSTENQEAVSTVDRDDNLIRVLDRIILYLRVVHSVDYYNHCEYPNEDEMPNRCGIL  442
            LGLEP+ E Q++ +TV+RD+ LI VLDRIILYLRVVHSVDYYNHCEYPNEDEMPNRCGIL
Sbjct  566  LGLEPTAETQDSATTVERDEQLISVLDRIILYLRVVHSVDYYNHCEYPNEDEMPNRCGIL  625

Query  443  HARGPPPASKTDMTLFIGPPVEAKMSNFLPEKPKEEKDKNETKLIAMALKDVDSEVDKFV  502
            HARGPPP +KTDMT FIG PVEAKM++FLPEKP++E +KNETKLI ++LKDVD+E+DKFV
Sbjct  626  HARGPPPTTKTDMTQFIGAPVEAKMTSFLPEKPRDE-NKNETKLINLSLKDVDTEIDKFV  684

Query  503  QANTRELAKDKWLCPLSGKKFKGPDFVRKHIFNKHAEKIEEVKKEVEFFNNYLKDPKRPM  562
            QANTRELAKDKWLCPLSGKKFKGPDFVRKHIFNKHAEKIEEVKKEVEFFNNYL+DPKRPM
Sbjct  685  QANTRELAKDKWLCPLSGKKFKGPDFVRKHIFNKHAEKIEEVKKEVEFFNNYLRDPKRPM  744

Query  563  LAEAPQQKRDEPQAYSHPGYGGGGGGGYGGGFGRPQPYYNQGFAPRSRGYAPRARGGPAG  622
            LAEAPQ KR+EP +Y+HP YGG  GG      GR  PYYNQG++ RSRGY PR+RGGP G
Sbjct  745  LAEAPQPKREEPPSYNHPSYGGSYGGY-----GRLPPYYNQGYSQRSRGYTPRSRGGP-G  798

Query  623  DFGRPIIHYRDLDAPREPEEFV  644
            D+ RP+IHYRDLDAPREPEEF+
Sbjct  799  DY-RPVIHYRDLDAPREPEEFI  819
```

Graphical representation

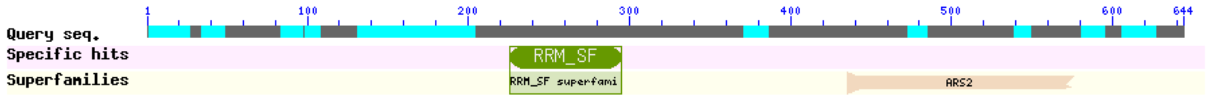

CG4572

>Cb.comp37407_c0_seq1 len=1629

cDNA

TTTTTTTTTTTTTTTTTTTTAACACAAAAATTATATTCCAATTTAAAAAGAGGCTAAATAGAGAGCTTGTTACATTACATTTTCATTTTTCTAA
TGAGTTTTTTGGAATGGCTTGTTTCTAGTAAATCTTGATATCATATCAAATGCCCACTGAGGTTGATCAGCTGGTACCATGTGTCCAGCATTTC
TAACTAAAACTTCTGTTAAATTGCCGACTTGTTTCACATAACCTGCTAGTTCCCCGCCAACAAACCACTTCAGTCTCTTTGCAGTTTTATATTG
ATCTGCACCACTAAACTTCAAATTTTGTAAAAAATTTTCTGTTAATGGATATGCAACAATAATATCCAACTGCCCATTGTAAATTAACACACGA
TAGTTTTCTAAAAGGTCAGATATCCAGGGAGCTACACTTTGCATTACATCTTGCAATAAATTGGTTTCAACATCTTGACCTATACCATTAAAGG
TGGTATTCCCAACATGAATGGCTGCTCTTATATCATTTCGTTGCACATAAGCACCCAAGAGCTCTATTTCAAGATCATTGGGATCTCTAGGATA
CAGAAAATTGAAGTAGTTATCAAAACCAGTGGAATTTTTAAATAATGAAGAGTGATTGTTCATATCACCATTAAGCAATGAGTCAAATACCTCA
AAAGCTTTAGCAAAATCTTTATTCTGAATATATTTAACTCCTTGTTGCTCATAATTTTTCACTAATTGTTGAGTATTCAAATCAATAAGACCAA
TTTGATACAAATAGTCTCCATATTTGAGCTGATGTTCTGGATCACACAAACCATTACCAATACTTAGTCCTTGTAGATTTATCTTAAGTTTGGC
AGATGGATTGTTTTCGTGTATTGTATATGCAATTGTAGGGACATATTTCCCTGCATAAGACTCGCCAGCAACAAAAAAATCATTTCTTTGTATT
TCTGGAAACAAAGTTAAAACCTGCAGAAGAGCAGCATACAAATCTCTACCCACTTTTGTTTCATTTTGTGCATATCCTTTATTTGTAAAACTGT
AACCAGTGCCCACAGGGTTGTCAAAATATAACACAGAGTGGCTTTTAGTCCAAGCATAAGGTCTAAGTTTTAAACCATGTTTTGGCTTTACCTT
AAATGGACCATTTTCTGCAAAGAGGCCAATTAAACTAGATGCTCCTGGTCCACCCTGCAGCCATAAAATAACCGGCGCATTCGTATAGTCTGTT
TGAGAAGGAAAGAACCAAAAAAACATATTTGAATCAAACTGCTTGTCTACTGTTAGATAACCGGAATAACTCTTTAAGTTCTTGAAACCGTTGA
AATGTACCTGTGCAGCATCCTGAGCTTCTTTAATTTTCTTCTGCTCAATAAGAGGTGTTAAAATGAGAGGAACTCCTGGATCTCCTTTCAATTT
TTCTTTTTTAATTTTGGGATACACATTTGGAAAAGATGCACTTGCAATAATACCCAAAGTAGCAAAAACAATACAAATATTCAAATTTGCCATA
ATATCAATCACAATACAGCAAAGATTTATTTTATGGAGTATTATCAGATTATTTCATATAAATGAACTAAAATTAAATGCCCCTAACCGGAAAG
GTTATCTCCACCACAGAACACTAGAGAGAAT

Protein RF -1: -1503->-91 (470AA)

MANLNICIVFATLGIIASASFPNVYPKIKKEKLKGDPGVPLILTPLIEQKKIKEAQDAAQVHFNGFKNLKSYSGYLTVDKQFDSNMFFWFFPSQ
TDYTNAPVILWLQGGPGASSLIGLFAENGPFKVKPKHGLKLRPYAWTKSHSVLYFDNPVGTGYSFTNKGYAQNETKVGRDLYAALLQVLTLFPE
IQRNDFFVAGESYAGKYVPTIAYTIHENNPSAKLKINLQGLSIGNGLCDPEHQLKYGDYLYQIGLIDLNTQQLVKNYEQQGVKYIQNKDFAKAF
EVFDSLLNGDMNNHSSLFKNSTGFDNYFNFLYPRDPNDLEIELLGAYVQRNDIRAAIHVGNTTFNGIGQDVETNLLQDVMQSVAPWISDLLENY
RVLIYNGQLDIIVAYPLTENFLQNLKFSGADQYKTAKRLKWFVGGELAGYVKQVGNLTEVLVRNAGHMVPADQPQWAFDMISRFTRNKPFQKTH

Comparison with *Tribolium* PREDICTED: similar to salivary/fat body serine carboxypeptidase (468AA)

```
Query  16    IASASFPNVYPKIKKEKLKGDPGVPLILTPLIEQKKIKEAQDAAQVHFNGFKNLKSYSGY  75
             ++S +FPNVY  IK++   + +PG+PLILTPLIEQ +IK+A  A++V+FNGFK ++SYSGY
Sbjct  18    LSSGAFPNVYGPIKQQPSE-NPGLPLILTPLIEQGRIKDALTASRVYFNGFKTIESYSGY  76

Query  76    LTVDKQFDSNMFFWFFPSQTDYTNAPVILWLQGGPGASSLIGLFAENGPFKVKPKHGLKL  135
              TV+K ++SN+FFWFFPSQTDY NAPV+LWLQGGPGA+SLIGLFAENGPF V   +HGLKL
Sbjct  77    FTVNKAYNSNLFFWFFPSQTDYANAPVVLWLQGGPGATSLIGLFAENGPFAVMRQHGLKL  136

Query  136   RPYAWTKSHSVLYFDNPVGTGYSFTNKGYAQNETKVGRDLYAALLQVLTLFPEIQRNDFF  195
             R Y+W K+HSV+Y DNP GTGYSFTN G+ QNET+VG DLY AL Q    LFP +Q+NDFF
Sbjct  137   RKYSWVKTHSVIYIDNPAGTGYSFTNNGFCQNETQVGLDLYNALQQFFLLFPALQKNDFF  196

Query  196   VAGESYAGKYVPTIAYTIHENNPSAKLKINLQGLSIGNGLCDPEHQLKYGDYLYQIGLID  255
             V+GESY GKY P IAYTIH  NP+AKLKINL+G+SIGNGL DP HQL Y DYLYQIGLID
Sbjct  197   VSGESYGGKYTPAIAYTIHTKNPTAKLKINLKGVSIGNGLTDPVHQLDYADYLYQIGLID  256

Query  256   LNTQQLVKNYEQQGVKYIQNKDFAKAFEVFDSLLNGDMNNHSSLFKNSTGFDNYFNFLYP  315
              N +   VK Y+ QG+KYIQ+KD+ KAF++FD+LLNGD+NNH+SLFKN TGFDNYFNFLYP
Sbjct  257   SNVRSTVKQYQDQGIKYIQSKDWVKAFQLFDNLLNGDLNNHTSLFKNVTGFDNYFNFLYP  316

Query  316   RDPNDLEIELLGAYVQRNDIRAAIHVGNTTFNGIGQDVETNLLQDVMQSVAPWISDLLEN  375
              DP++ E+  +G Y+QR+D+RAAIHVGN TF+G  Q+VE NL+ DVMQSVAPW+++LL +
Sbjct  317   IDPSN-ELIYMGEYIQRDDVRAAIHVGNATFHGESQEVELNLMTDVMQSVAPWVAELLSH  375
```

```
Query  376   YRVLIYNGQLDIIVAYPLTENFLQNLKFSGADQYKTAKRLKWFVGGELAGYVKQVGNLTE  435
             YRVLIYNGQLDIIVAYPLT N+LQNL FS AD+YK A+R KW+V  +LAGYVKQ GNLTE
Sbjct  376   YRVLIYNGQLDIIVAYPLTVNYLQNLNFSAADEYKKAQRYKWYVDEDLAGYVKQAGNLTE  435

Query  436   VLVRNAGHMVPADQPQWAFDMISRFTRNKPFQ  467
             VLVRNAGHMVPADQP+WAFD+ISRFTRNKPF
Sbjct  436   VLVRNAGHMVPADQPKWAFDLISRFTRNKPFH  467
```

Graphical representation

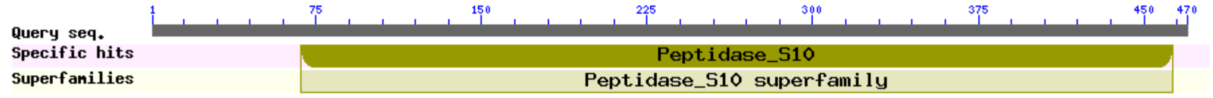

>Cb.comp33398_c0_seq1 len=2579

cDNA

```
GTCGGGCCTCTGCATAATCTACATAAGAACGTCCGTTTGAAAGAAAGCTTTGTCCATTTACATCCAGAATATAAAAGCGTTAATATTCATTCAT
TCATCGCATTTACATACGCTCTGATGCAACATCGTTATTTGTTAATCCCCAGACTTGTATTAGAGGCCATATCGAACGATAAGCGATAATTTAG
TCGTTTGGAGATAATAAGAGCCAAATATTTATAAAGCATAGTCGATAATACTCAACCTTAACATTCGAATTTTTGACTAGCCAACAAACCAAAT
GTCGTCTCGGAGGCTGTTTGCAGTTATTTTTATTCTCTTCGTTCTGGATGAGTGCCAGGGCAGATTCCAGCAGTACAACAAAAATTTCAAAGTA
ATACCTCTGGATGGGGATCCTGGAGAGCCGCTTATTTTAACTCCATTACTTCGACAAAACAAAGTGCAAGAAGCTAGAGAAAAGGCGCGAGTTG
TAGACGAGAGATTTCTTAACATTACGAGCCTTTCGGGTTACTTCACCGTTGACGAAACGTACGATTCTAACTTGTTCTTCTGGTTCTTTCCTTC
AGAGAATAACTACGTTACCGACCCAGTAGTTTTATGGCTCCAAGGAGGTCCGGGAGCCTCTAGTCTGTTGGCGCTTTTTACAGAAAATGGTCCT
TTCGTCGTCGGTACCGATTACAGAATAATCCAAAGATCATACTACTGGAGTCAATACTACTCGGTTCTTTTTATTGACAGTCCCGCTGGGACTG
GTTTTAGTTTTACCAACGGTGGATACGCCCAGAACCAGACTAAAGTAGGAGCCGATCTCTACAATGCACTACTACAGTTCTTTCAGCTATTTCC
TGAACTGAACCAGAACGATTTTTACATCTCCGGGGAATCTTATGCTGGAAAATACATCCCGGCTATCGCTCACACCATCTTGACAAGAAACCCC
GTTGCTGAGCAAGTGATCAATTTGAAAGGCTTGCTTATTGGCAATGGCTTGAGTGATCCAGAACACCAGTTTGAGTATGGGGAATACCTGTATC
AGATAGGTCTGATTGATTCGAACACTAGAGACACAATGAACTCAGTTGAAGATTCTATTATTCGATACATCCACGAAGAGAACTATCAGAAAGC
GGTAGAAGGTTTCAGCACGCTCATTTTAGGCGATGAAAAGGGTGAGGAATCAACGATTTTCGAAAACGCAACCGGTTTTGAGAGCCACTATAAC
TACCTAAGACCAAAAGAAGATTATGATTATTGGGCAGATCTGATCCAAAGGTCCGATCTGCGATCTGCTATCCACGTTGGTAACACGACATTTG
GAGATGAAAAGGTACGCGAAAACCTTGTCTTGGATATAACCAAGAGCGTGGCGCCTTGGATCTCAGAGTTGTTGGATCATTACAGAATACTCAC
TTACAACGGCCAGCTTGATATAATCGTCGCCTACCCATTGACGGAGAATTATTTGAAAAATCTGAATTTTAGCGCTGCCGATGAATACTCGAAG
GCGTCGAGGGTGATTTGGGAGGTCGACGGTGAAATAGCAGGGTATTATAAGAAGGCAGGAAAGCTTACGGAAGCGATGGTTAGAAACGCGGGAC
ACATGGTGCCTGGAGATCAACCGAAATGGGCTATGGATTTGGTTAAGAGATTTATAAGAGACACTTTGTGAGCCTTAGTTTTCCATTAATAAAT
AATTATTGCTAAAATATTCTGAAAACGTTTCGCTAGCCGAAAATAGTTGGGCATTCTAATGAAAACCAGCTATTGAATTGTTTTTTTTAATTTT
TGTATTGTTTTATCGTGCGCTTGCTGGTATCTAACTTGCAATAGGCAGATCCTTCCTGTTATTGTATATTTAATATAGTCGAGCTAATATGACA
CCAAATAATATCAAATATGAAATGGAATAATATAAATCCAATAGCAAATACATCCGTACGGGCAGTTGGAAAGGCTGAGTACAACACGTAATTA
ATTAGATTAGATTGAGATGAAGATAGTAACATTGACACAGATAATTTTAAAGTAAAATAGAATTAAGTTGTCAAAATTGATTTTTTGTGTGCCA
TATTCTTTTGTTTCTGCAAATGACATTAAAAGTGAGAGTTTAATGACACAAACACAAGGGATTTAAAAGGGTATTTTTACTCGTATCTTAAGAG
TACACTTATAATCTCGTAATATTTTACCAGTAGAAATGTATTCGAAGATAGCGGGCTTCTTGCATGCGTCATAATTTAAAAAATATTCTACATA
ATATAAAAGAAAAATTTAAATTAATATAATACAAAAATTAATTAGACACTTAGTAATTCGCCGCCAGGTAATAATACCAGCTGGGCAACCTGGA
TTGGTATAGTATAGTATAGTATTTTATAAGATTTATGAAAGTGACCTGGCCTCAGTGAATAAATGAAATGCAATAAAAATATTAAATTAATCAT
AAGACATCGGACCTTTATCGTAACAAAGTCTGCTCCTGCTAATATATGTATGTCACTAATATGTGTAGGATATAACCATTATACAATACATGAA
AAGGGTAGATACAAGAAGTCTGTTTTTAGCAGCCAAAAATA
```

Protein RF 2: 281->1669 (462AA)

```
MSSRRLFAVIFILFVLDECQGRFQQYNKNFKVIPLDGDPGEPLILTPLLRQNKVQEAREKARVVDERFLNITSLSGYFTVDETYDSNLFFWFFP
SENNYVTDPVVLWLQGGPGASSLLALFTENGPFVVGTDYRIIQRSYYWSQYYSVLFIDSPAGTGFSFTNGGYAQNQTKVGADLYNALLQFFQLF
PELNQNDFYISGESYAGKYIPAIAHTILTRNPVAEQVINLKGLLIGNGLSDPEHQFEYGEYLYQIGLIDSNTRDTMNSVEDSIIRYIHEENYQK
AVEGFSTLILGDEKGEESTIFENATGFESHYNYLRPKEDYDYWADLIQRSDLRSAIHVGNTTFGDEKVRENLVLDITKSVAPWISELLDHYRIL
TYNGQLDIIVAYPLTENYLKNLNFSAADEYSKASRVIWEVDGEIAGYYKKAGKLTEAMVRNAGHMVPGDQPKWAMDLVKRFIRDTL
```

Comparison with *Tribolium* PREDICTED: similar to salivary/fat body serine carboxypeptidase (468AA)

```
Query  8     AVIFILFVLDECQGRFQQYNKNFKVIPLDGDPGEPLILTPLLRQNKVQEAREKARVVDER  67
             AV+ + F L+    G F        K   P + +PG PLILTPL+ Q ++++A    +RV
Sbjct  8     AVLLLTFSLNLSSGAFPNVYGPIKQQPSE-NPGLPLILTPLIEQGRIKDALTASRVYFNG  66
```

```
Query  68   FLNITSLSGYFTVDETYDSNLFFWFFPSENNYVTDPVVLWLQGGPGASSLLALFTENGPF  127
            F  I S SGYFTV++ Y+SNLFFWFFPS+ +Y   PVVLWLQGGPGA+SL+ LF ENGPF
Sbjct  67   FKTIESYSGYFTVNKAYNSNLFFWFFPSQTDYANAPVVLWLQGGPGATSLIGLFAENGPF  126

Query  128  VVGTDYRIIQRSYYWSQYYSVLFIDSPAGTGFSFTNGGYAQNQTKVGADLYNALLQFFQL  187
             V   + +  R Y W + +SV++ID+PAGTG+SFTN G+ QN+T+VG DLYNAL QFF L
Sbjct  127  AVMRQHGLKLRKYSWVKTHSVIYIDNPAGTGYSFTNNGFCQNETQVGLDLYNALQQFFLL  186

Query  188  FPELNQNDFYISGESYAGKYIPAIAHTILTRNPVAEQVINLKGLLIGNGLSDPEHQFEYG  247
            FP L +NDF++SGESY GKY PAIA+TI T+NP A+  INLKG+ IGNGL+DP HQ +Y
Sbjct  187  FPALQKNDFFVSGESYGGKYTPAIAYTIHTKNPTAKLKINLKGVSIGNGLTDPVHQLDYA  246

Query  248  EYLYQIGLIDSNTRDTMNSVEDSIIRYIHEENYQKAVEGFSTLILGDEKGEESTIFENAT  307
            +YLYQIGLIDSN R T+   +D  I+YI  +++ KA + F  L+ GD     S +F+N T
Sbjct  247  DYLYQIGLIDSNVRSTVKQYQDQGIKYIQSKDWVKAFQLFDNLLNGDLNNHTS-LFKNVT  305

Query  308  GFESHYNYL---RPKEDYDYWADLIQRSDLRSAIHVGNTTFGDE--KVRENLVLDITKSV  362
            GF++++N+L    P  +  Y  + IQR D+R+AIHVGN TF  E  +V  NL+ D+ +SV
Sbjct  306  GFDNYFNFLYPIDPSNELIYMGEYIQRDDVRAAIHVGNATFHGESQEVELNLMTDVMQSV  365

Query  363  APWISELLDHYRILTYNGQLDIIVAYPLTENYLKNLNFSAADEYSKASRVIWEVDGEIAG  422
            APW++ELL HYR+L YNGQLDIIVAYPLT NYL+NLNFSAADEY KA R  W VD ++AG
Sbjct  366  APWVAELLSHYRVLIYNGQLDIIVAYPLTVNYLQNLNFSAADEYKKAQRYKWYVDEDLAG  425

Query  423  YYKKAGKLTEAMVRNAGHMVPGDQPKWAMDLVKRFIRD  460
            Y K+AG LTE +VRNAGHMVP DQPKWA DL+ RF R+
Sbjct  426  YVKQAGNLTEVLVRNAGHMVPADQPKWAFDLISRFTRN  463
```

Graphical representation

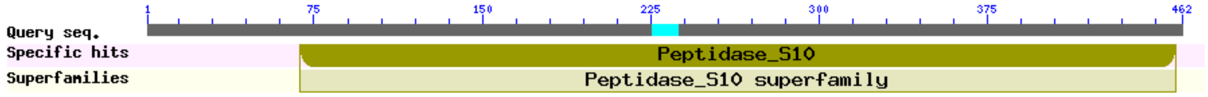

>Cb.comp44717_c0_seq1 len=2780

cDNA

GTCACATAGATTATCATCAACGGATGGCACTAAACCTAAACTGATTATCTCCGAAGATTGGAATTATATAAGCTTCGTTATCCGGCAACTACAA
CGACTACCAACTAAGCAACCATACTGGGGATATGAAATTTGCAACGTTCATAATACTACTGGCTCTCGGAGGCAGCCAAGCATCCTTCTTCAAC
TGGGAGAAAAGAATAAGGCGTCTTCCGATTCCAGATGATGTCGGTGAACCGCTTATCTTGACTCCTTACCTAAAAGAAAATAGAAGTGAAGAAG
CCAGAAAAGCTGCGGAAGTTAACTACGATGGATTCCAAGGTGTAAAGAGTTATTCCGGATATTTTACAGTAGATGCAAGGTTCGATTCTAATCT
GTTTTTCTGGTTCTTTCCTTCTGCTAACGATTACGAAAACGATCCGGTCCTATTATGGTTGCAAGGTGGTCCTGGAGCTCCCAGTTTATATGCC
CTTTTCACAGAAAATGGTCCCTTCGAACTCGATGATGATGATTCTCTTAAACTAAGAGAGTATTCGTGGCATAAAAACCACTCTATATTATACA
TAGACAGTCCAGTGGGCACCGGTTTCAGCTATACCAATGGCGGCCTGGCTGACAATCAAACAAAGGTCGGAGAGAACCTTTATCAAGCTCTTGT
TCAGTTCTTTACGTTGTTTCCAGAAATTCAAAAGAATGACTTCTTTGTAACCGGTGAATCGTATGCTGGTAAATATATACCAGCTATTGGGTAT
ACCATTTACAAGAATAACCCTACAGCCGACGTTTTTATAAATCTGAAGGGCTTGTTAATTGGTAATGGGCTCTCAGATCCCATCAATCAATTGG
ATTACGGCGACTATGTCTACCAACTTGGGCTCGTCGACAGCGATACCAGAGACTTGCTCAATGAAATGAAACAAGAAACGATTGATTTAATTCA
CAAAGGCGATTATGAAGGTGCGACGGAGATCATGAACGGTGACATGATGTGGGTAATTATGGGCGCTTCTGGTATCACAGATATTTACAACTAT
GAAAATCTGGAAATTGAATATCTGGACGAATGGCAGGACTTTGTTCAGGCGAATCTACGTTCAGTTATTCACGTGGGAAATGTGGTAATCGGAA
GCGGGGACGTATGGGACCACTTGGAGGCAGATATTACCAAAAGTGTTGCACCTTGGGTTTCGGAGCTGCTTAGCAACTATCGTGTATTGATTTA
TAACGGCCAACTAGACATTATCGTAGCTTATCCATTAACTGTCAACTACTTGCGAAACCTTAACTTCAGTGCAGCTGCCGAATATAAATCTGCC
TACAGATCCCTGTGGGTAGTCGATGACGATGTAGCCGGTTACGCGAAAACAGCTGGCAACTTGACGGAGGTTCTTGTAAGGAACGCGGGTCATA
TGGTGCCAAAGGATCAACCAAAATGGGGTTACGATTTGGTTTATAGGTTCACTAGGAACTTGACTATAGGTTTTTGACCGCTGTGATTTTGTTT
AACTATTTGAAGCGTAATAAAAGCTAAAATATAAACATATTATTATAAATATTATATTGTAACGTAACTTTCGAGATCCACGAATTTGAGAGTA
TACGCCGACCTAGCCCGTGAGTCGGGGTGGGTCGTGTGGTATAATTTCAGATTCTTAATTAAGTGGGCGCCAAGTGTTTTATGAAACGTAACTC
ACCTATCACCAGTTGTAATACAAGCCAAAAACGAAACACTAAATATCTTGACTTGGACTCAAAATTTTCTCTTCTGGCAGGTAAAAAAAAATAA
ATAAATCAAACATATATATTTAATTAAAGATTTAATACGAGCTGAGGCGAAATTACTACCTCTGGGCCACCCTGCACACTAACAATAAATAAAT
AAAAATACCCAAGCGACTAGCAAAGTATAACGCAATTATTAATAGGCAAACTCTCGAGAGCCGCAATCCGTGTTTGTAAAATAGCTTACAAGTT
TTAAATTTAGGAATATACAATATACAATCGGAAAACTCCAGTTATATCAGATAGCCACGCAAAATTACAAACATACCGTTAAACCACACAGCAA
TAATATATATAATTACATCCCGGTACAGATATTTGGCGTGTATATTTGTTTGCAAATAAAAAAAATAACTGAATATATATCTATATAAATCGAA
CGAAAGATGATACCGGAAGCACAGTCTATGATTCCCCTAGGGGGTACTTATCCTGTCCTGAGGAAGGTCACCCGCGCTCTAGCTCTGACAACCC
TGGATTGTGGCTCCAACAGCCTTATACAGAATTGTACCCCGAAGTTGCCAATTCGGATCACACTTTTTAATGTCCATGCTGTTTTTCTACGAA

AACGGGTCTATCTACACAAAAAAAACGCCTCTGTTCCCAGATACCCCAAAGCAAAGAGGCGTTTCCTAGCCCTTACTCCAAGCACTAAACCAAT
GAAATAAATCGGTGATTCGACGAAGTCCAAATAGCACACACTAGGCTAAACCCTAAGAACTTCTCGAGCGGAAAACTAAGACAAAGACTAAGAC
AAAAAATTTTTCTACAGCCAAGCCACAAAAAAAAAACGGCACGCCATATACGTATATTCCGCGCTATTAAAGTTTACAGGGTGAGTCCCAAATGC
ATGTTTCGCCTCCTTTCTTTCTCCATTGTTTGTTTTCTATATGTATATTTTTTTAAAGACATCAAACTCGTATCATTAAATCGTCATATTCAAC
AATATATATTGTCAAATTGACTGACAAATGTCAGCGATGACAGTTGACGCCATT

Protein RF 3: 126->1487 (453AA)

MKFATFIILLALGGSQASFFNWEKRIRRLPIPDDVGEPLILTPYLKENRSEEARKAAEVNYDGFQGVKSYSGYFTVDARFDSNLFFWFFPSAND
YENDPVLLWLQGGPGAPSLYALFTENGPFELDDDDSLKLREYSWHKNHSILYIDSPVGTGFSYTNGGLADNQTKVGENLYQALVQFFTLFPEIQ
KNDFFVTGESYAGKYIPAIGYTIYKNNPTADVFINLKGLLIGNGLSDPINQLDYGDYVYQLGLVDSDTRDLLNEMKQETIDLIHKGDYEGATEI
MNGDMMWVIMGASGITDIYNYENLEIEYLDEWQDFVQANLRSVIHVGNVVIGSGDVWDHLEADITKSVAPWVSELLSNYRVLIYNGQLDIIVAY
PLTVNYLRNLNFSAAAEYKSAYRSLWVVDDDVAGYAKTAGNLTEVLVRNAGHMVPKDQPKWGYDLVYRFTRNLTIGF

Comparison with *Tribolium* PREDICTED: similar to salivary/fat body serine carboxypeptidase (468AA)

```
Query  4    ATFIILLALGGSQASFFNWEKRIRRLPIPDDVGEPLILTPYLKENRSEEARKAAEVNYDG  63
            A  ++  +L  S  +F N    I++ P  ++ G PLILTP +++ R ++A  A+ V ++G
Sbjct  8    AVLLLTFSLNLSSGAFPNVYGPIKQQP-SENPGLPLILTPLIEQGRIKDALTASRVYFNG  66

Query  64   FQGVKSYSGYFTVDARFDSNLFFWFFPSANDYENDPVLLWLQGGPGAPSLYALFTENGPF  123
            F+  ++SYSGYFTV+  ++SNLFFWFFPS  DY N PV+LWLQGGPGA SL  LF ENGPF
Sbjct  67   FKTIESYSGYFTVNKAYNSNLFFWFFPSQTDYANAPVVLWLQGGPGATSLIGLFAENGPF  126

Query  124  ELDDDDSLKLREYSWHKNHSILYIDSPVGTGFSYTNGGLADNQTKVGENLYQALVQFFTL  183
             +     LKLR+YSW K HS++YID+P GTG+S+TN G   N+T+VG +LY AL QFF L
Sbjct  127  AVMRQHGLKLRKYSWVKTHSVIYIDNPAGTGYSFTNNGFCQNETQVGLDLYNALQQFFLL  186

Query  184  FPEIQKNDFFVTGESYAGKYIPAIGYTIYKNNPTADVFINLKGLLIGNGLSDPINQLDYG  243
            FP +QKNDFFV+GESY GKY PAI YTI+  NPTA + INLKG+ IGNGL+DP++QLDY
Sbjct  187  FPALQKNDFFVSGESYGGKYTPAIAYTIHTKNPTAKLKINLKGVSIGNGLTDPVHQLDYA  246

Query  244  DYVYQLGLVDSDTRDLLNEMKQETIDLIHKGDYEGATE----IMNGDMMWVIMGASGITD  299
            DY+YQ+GL+DS+ R  + + + + I  I   D+  A +    ++NGD+         +T
Sbjct  247  DYLYQIGLIDSNVRSTVKQYQDQGIKYIQSKDWVKAFQLFDNLLNGDLNNHTSLFKNVTG  306

Query  300  IYNYENL--------EIEYLDEWQDFVQANLRSVIHVGNVVIG--SGDVWDHLEADITKS  349
              NY N         E+ Y+ E+    + ++R+ IHVGN      S +V  +L  D+ +S
Sbjct  307  FDNYFNFLYPIDPSNELIYMGEY--IQRDDVRAAIHVGNATFHGESQEVELNLMTDVMQS  364

Query  350  VAPWVSELLSNYRVLIYNGQLDIIVAYPLTVNYLRNLNFSAAAEYKSAYRSLWVVDDDVA  409
            VAPWV+ELLS+YRVLIYNGQLDIIVAYPLTVNYL+NLNFSAA EYK A R  W VD+D+A
Sbjct  365  VAPWVAELLSHYRVLIYNGQLDIIVAYPLTVNYLQNLNFSAADEYKKAQRYKWYVDEDLA  424

Query  410  GYAKTAGNLTEVLVRNAGHMVPKDQPKWGYDLVYRFTRN  448
            GY K AGNLTEVLVRNAGHMVP DQPKW +DL+ RFTRN
Sbjct  425  GYVKQAGNLTEVLVRNAGHMVPADQPKWAFDLISRFTRN  463
```

Graphical representation

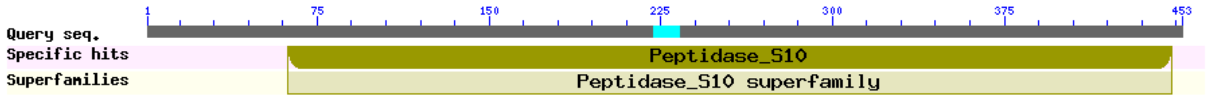

Egghead

>Cb.comp36926_c0_seq1 len=4181

cDNA

GGAATTCACCTTAGATTTTACTCGCTTATTGAATTCACAGAAAATGTATAGTTAATGCCATTATTGATTTAGGGTTCCAAACCACTGGCTTAGA
TTACTGACTGGTCAAGCCTGGCTGCGTGTATATTATGTTGTATGCAAATTCCAACTTTTAATGTTAAACCAGAGACGAAGTTCTCTCCGGTTAA
ACCTTCCGGTAGAATGCGCCAGTGTACGAGACCTATTATTTTCGACCGGCATTGTTTTTGTCAGTGTCAGTTGACTGGTAGATGTGTGTTTGGA

GCTATGTAGCAGTGAATCAATCATTAATTATTGTGGCAAGTGGTGATCGTTCAACGGTTTGTTGTAGTGACGGTAGTTTTTGAGTGAACACAAA
AAGTAAAAACTAAAAAATGTTTAAAGAGTTTTCGAAATGAATTACAGTGACCCAAGCGGAGGTTGCCCTGTTAACAATATTAGAGTTTAAAGGG
GAGTGATTTGAGAATTTCGTGTCGATTTTTCATTATGCTTGGTAGCAAAACTAAACATTACCTCCACTGTATGCTTTTTTTTACTGTAATATTA
TTTTTTGAAATATTTTCTGGTGGTTTACGACTTTTTAAGGGAGGCATTTTTGTACCGGCATCAGAAATCAATCCATGGGTGGAGTACGGTTTTT
TTGGAGCGTGTATTTTATACGCCCTACGCTTGGTTACCTTTTTGCCATTGCCTCAAGTAATATTTAATTTTATTGGTCTTACGTTTTATAACGC
TTTTCCGGAAAAAGTTTCTTTAAATGGGTCGCCTCTTTTAGCGCCTTTTATTTGTATTAGGGTAGTTACTAGGGGCGACTTTCCCCAATTAGTG
AAAAACAATGTAAACAGAAACATGAACAGTTGTTTAGATACGGGTTTAGAAAACTTTCTAATAGAAGTAGTGTCTGATAAACCAATTGGATTGG
ACAACCATAGAAGAATTCGTGAAATAGTGGTACCCCCTGATTACCGCACGCCGACAGGAGCTCTTTTTAAAGCCAGAGCGTTACAGTATTGTTT
AGAAGATAAAATAAACATTTTAAGTGATAATGATTGGATAGTTCATTTGGATGAAGAGACCGTTCTCACGGAAAACTCCATCAAAGGGATTTTG
AATTTTGTCATAGATGGAAGGCACCATTTTGGGCAAGGGCTAATCACGTACGCCAACGAGGAAGTTGTCAATTGGATCACAACGTTAGCCGATA
GCTTTAGGGTTACAGATGACATGGGCAAGTTGAAATTACAGTTTTTGTGGTTCCACAAGCCCTTGTTTAGTTGGAAAGGGTCATTTGTTGTCAC
CCAGCTTGGAGCTGAGAGGGATGTTTCGTTCGACAACGGATTGGACGGATCGATTGCTGAAGACTGCTTTTTCGCCATGAAGGCGTACAGTATG
GGCTATAGTTTTACGTTTATTGAAGGCGACATGTGGGAAAAGTCGCCTTTTACATTATGGGACTTTATCCAGCAGCGCAAACGGTGGATCCAGG
GTATCCTGTTGGTCGTACACTCGCGATCGATTCCGTTTAGAAATAAATTACTGTTAGCGTGCGCCTGTTATTCGTGGGTGACACTGCCGTTGTC
TACATCGAACATATTTCTAGCCTCTAAGTTTCCGATACCGTGTCCGCCTTTGATCGATTTCCTGGTGGCGTTTATAGGTGCCGTTAACATTTAT
ATGTTTATATTTGGGGTAATAAAATCATTTAACGTGTACAGGTTCGGTTTAGCAAGGTTTTTTTTGTGTATGTTCGGCGCGGTGATCGTCATGC
CCTTTAATTTAGTAATAGAGAATATCGCTGTATTATGGGGTATATTTGGAAAGAAACACAAATTTTACGTTGTGAATAAAAGTATAGGTCATGA
GATTACGGTATAAAAATATTACGAAATTACCTCGCCCATTTTTGGTTTGAGCTGGCCATTCAATCAAGTATCCAGAAATTTAGAAAATGTGGAA
AAATGTACCTCAAAGTGTTTATATTTTTGTAAATTTAGTATATTTTGACATTAAGGAAACTGTTCGGGTACAGCCCAACGCCTTACAAAACTCT
ACTTTGAAAATGTACCATGCATTTAGTTTTTGAGATACCAAAATGTTTTTTCTAAATAATTTCATCTTCATTATTTTTAATTAACTTTTATTAT
GAATAAATTATATAAGTATAAAATTTCGCGCTCATCTACTGATAACGCAACGTCATTTGTTAGCAGAAAGCCCACCGCTTTTTATCGTTGTTTC
TACGCGAATAGTTTTTACTTTTAGCAAAAAAGTTCCTCTTGATGTTTGATGCAATTTTACTGGGTGAATATTGAACAATAGATAATCAAACTAA
CCAAAACTTTTATACAAGTTTGTAAGAGAGTAACTTGAAATAAGACAAACACAAGCATGACCAAATTTTTCAAGTACGTAGTCCAGTAGGCTTT
GCTATCGAATGGCCAAATGCGAACTGACAAGTGGCGTCTGCGCCAATCACAACACGTCCTCAGGCACGCTTAAAATTTAAATTTAGCGCTACTT
TAACTAGCGAATAAAATATTGTCTATGTGCAAATTAGGTTAGCGGATCACATAATTTGGCAATAATTTAATGTTTTTCTATTGCTTTATGTAGA
TTTTATATAAACAGGCAATATGCTATGCAACGTAGAAATTATGTCGTCACGGAAACCTTTTTTGGTATCATACTCCAGACGTTTTCGAGAAAAT
GCTAGCAAACACGAATATGGAATTCCTTAACCAATGATAGAAAATGTAGAAGTGTTCCTCTCGTCCACTTTTATTAAAAATAGTGCGGTTCTTC
AAAATTCTAGGTCGGAGAAGCGAGTCTATGATATTGATAAAACAACACCTGTATGAAAGCACATTAATTAATGACAATTATTACCCCTTCAAGG
TGAAGCAAAATTTATCTCGTTTTTTATTGGAAATACCGATCAAATGATTCATTTTAATTGTATGTCCTGTCTATGTAGGTAATTATAAATTGTA
TTAATTTAATTATAATGTAAATTAGCTTGGGAAGCAAATGCATTGAATCATTTTATGCACCTACTTAAAAATTTTAAAAATTATGTTGTTCTGT
GTATTGTTCTACATTTGACTAAAAAAGTTACAATAAACTGCGTTCAAAAGATTCTTACCAAAAAAAATGGCGTGACTGGTATCATTATCCTCTA
CTTTGAATATACATAATATTTTAACTCAAAATGGCAATTTTGAAACGGTAATCTTTAGGTATTATTGTGCACCAAAAAAAAAAATGCGTTTAAAA
TACATTACAAAATATTGTTATTATTGTTGCTATTGTTTCTTAACATACGGGAGTTTCGAAACGAAATTTTAGGTTTTGGTCAACCTTTACGGAT
TGATTGAAATTCTAAAGTTGATCGTTGAACTTCAAGGGTATTTGTGTTAATTTTAAAGGTTAAATTCTCCAAAAGTGTTAAAAATACCCTGTAT
TGTAAAATGATTTTTTATTCTCTGAAAATATGCTTTTACTTATTTGCTTAAAAGTGTAACTAAAAAAGCTTAAGGTTTTGCATAATCAGTGGGA
CTAACATTGTCACTTAATTTGATAGTAATTACTATATATGTAATAATGAGTGTCAATGAAAGTGTTGGGTATGTATTGCGTATAGACCGCCTTA
AAGCTTAGAAAATTTATATGTATAACGTCCCAAATGTACAAATACATTTTTTCCACGATTTCGTGGCTATAAGATCTGCTTTAATTCTTTGAGT
TGGATATTGGGCGAGTTTAAAATCGAACATCACTTTGAATAAAATCATCTTCAAGTTAAACATAAAAGTCAATTTTTTTACGTTTTTTTTTTGC
ATTGGCACATTAAATTTTTGTAAGGAGAGTATAATAATTTGGTCGCTCCATTTTATTTCTTAAAAGCATGTAAAATTGCAAAAAAAAATGTTTTT
ATGTGTGCTAGTCATTAGATAATTTTATTAAAAGTAGAAAAATATGTACATATTATTGATCTCCTGTGGCAAATATCTAGTTTTTGAACTTAAC
TAATGTTTGGCGACAACTGGACAGCCCACATAGTCAAAGAAAAGCGAGTACACGATTCTACAGGTACTTATTTAAGTATACATTTTCTGTATTT
AATGGGAGTCGAACCAATAAAGAGAAAACCAAATCTAAAAAAAAA

Protein RF 1: 505->1883 (462AA)

MLGSKTKHYLHCMLFFTVILFFEIFSGGLRLFKGGIFVPASEINPWVEYGFFGACILYALRLVTFLPLPQVIFNFIGLTFYNAFPEKVSLNGSP
LLAPFICIRVVTRGDFPQLVKNNVNRNMNSCLDTGLENFLIEVVSDKPIGLDNHRRIREIVVPPDYRTPTGALFKARALQYCLEDKINILSDND
WIVHLDEETVLTENSIKGILNFVIDGRHHFGQGLITYANEEVVNWITTLADSFRVTDDMGKLKLQFLWFHKPLFSWKGSFVVTQLGAERDVSFD
NGLDGSIAEDCFFAMKAYSMGYSFTFIEGDMWEKSPFTLWDFIQQRKRWIQGILLVVHSRSIPFRNKLLLACACYSWVTLPLSTSNIFLASKFP
IPCPPLIDFLVAFIGAVNIYMFIFGVIKSFNVYRFGLARFFLCMFGAVIVMPFNLVIENIAVLWGIFGKKHKFYVVNKSIGHEITV

Comparison with *Tribolium* PREDICTED: similar to conserved hypothetical protein (464AA)

```
Query  1    MLGSKTKHYLHCMLFFTVILFFEIFSGGLRLFKGGIFVPASEINPWVEYGFFGACILYAL  60
            ML SK KHYLHC LF  VI  FEIF+GG++L  G  FVPA +INPWV YG+ GA +LY L
Sbjct  3    MLTSKAKHYLHCCLFIYVIFMFEIFTGGIKLLDGAFFVPAEDINPWVHYGYLGALVLYLL  62

Query  61   RLVTFLPLPQVIFNFIGLTFYNAFPEKVSLNGSPLLAPFICIRVVTRGDFPQLVKNNVNR  120
            RLVTFLPLPQV+FNFIGLT+YNAFP+KV L  SP+LAPFICIRVVTRGDFPQLVKNNVNR
Sbjct  63   RLVTFLPLPQVLFNFIGLTYYNAFPDKVVLKASPILAPFICIRVVTRGDFPQLVKNNVNR  122

Query  121  NMNSCLDTGLENFLIEVVSDKPIGLDNHRRIREIVVPPDYRTPTGALFKARALQYCLEDK  180
            NMN CLD GLENFLIEVV+DK +G++ HR++REIVVP DYRT +GALFKARALQYCLED
```

```
Sbjct  123  NMNKCLDAGLENFLIEVVTDKKLGMEKHRKVREIVVPQDYRTKSGALFKARALQYCLEDD  182

Query  181  INILSDNDWIVHLDEETVLTENSIKGILNFVIDGRHHFGQGLITYANEEVVNWITTLADS  240
            +N+LS NDWIVHLDEET+LTENS++GILNFV DG+H FGQGLITYANEEVVNWITTLADS
Sbjct  183  VNVLSPNDWIVHLDEETLLTENSVRGILNFVGDGKHQFGQGLITYANEEVVNWITTLADS  242

Query  241  FRVTDDMGKLKLQFLWFHKPLFSWKGSFVVTQLGAERDVSFDNGLDGSIAEDCFFAMKAY  300
            FRVTDDMGKLKLQF  FHKPLFSWKGSFVVTQ+GAER+VSFDNGLDGSIAEDCFFAMKA+
Sbjct  243  FRVTDDMGKLKLQFRMFHKPLFSWKGSFVVTQVGAEREVSFDNGLDGSIAEDCFFAMKAF  302

Query  301  SMGYSFTFIEGDMWEKSPFTLWDFIQQRKRWIQGILLVVHSRSIPFRNKLLLACACYSWV  360
            S GYSF FIEG+MWEKSPFT WDFIQQRKRWIQGILLVVHS+ IP RNKLLLAC+CYSW+
Sbjct  303  SKGYSFNFIEGEMWEKSPFTFWDFIQQRKRWIQGILLVVHSKDIPLRNKLLLACSCYSWL  362

Query  361  TLPLSTSNIFLASKFPIPCPPLIDFLVAFIGAVNIYMFIFGVIKSFNVYRFGLARFFLCM  420
            TLPLS SN+ LASKFPIPCPP+IDF+ AFIGAVNIYMF+FGVIKSF VYRFGL RF LC+
Sbjct  363  TLPLSVSNLVLASKFPIPCPPIIDFICAFIGAVNIYMFVFGVIKSFTVYRFGLGRFVLCL  422

Query  421  FGAVIVMPFNLVIENIAVLWGIFGKKHKFYVVNKSIGHEITV  462
             GAV+V+PFNLVIEN+AV+WGIFGKKHKFYVVNK+    ++TV
Sbjct  423  CGAVLVIPFNLVIENVAVIWGIFGKKHKFYVVNKNARPQVTV  464
```

Graphical representation

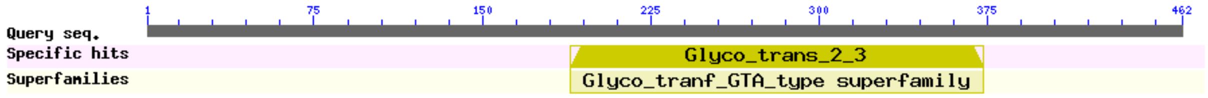

ninaC

>Cb.comp34434_c0_seq1 len=3669

cDNA

TAAGTGCGCTACGAAATTAGCCAAGACCGTATAATATAACCCATGAAATTTAATGAGCAAGGGAAAAAATTTACCGAAGATAAACGAAGAGCGG
AGAGGAATGTGAAATCTCTTAGATGCGTATAGAAACGCCTCGAAATACTCAAATTCAAATTCGTTAAAATGAAGTCGGGCCTGGGCAATGTACC
CGATCCGGGTGATAGGTACACGTTTGGTGACTTATTAGGAACCGGAGTATTTGGCAAAGTTTACAAAGCGTTCGACAATCAAGCCAATCAAAAA
GCGGTTGCCATAAAGATACAAAAATTTGATCAAGATAACAAGGAGTTTGTCCAAGAAGAATACGAAATCCTGAAGGACTTTAGTCACATTGTCT
ATTTGGTTGATTTTTACGGAGTTTTTAAACGAGATAACGAAATTTGGTTTGTTTTAGAGCAATGCGAAGCAAATACGGTCGTAGACTTGGTGCA
GGGACTCTTACTCAAAAATAGGCGAATATCTGAGGAGCATATCGCGCATATTCTTAAAGAACTGGTTAAGGCCATAATTTACTTGCATGAGCAC
AACGTAGTCCACAGAGACATAAAAGCCAGCAACATACTGCTAACGAAAGAAGGAGAAATAAAGTTATGCGACTTCGGCCTGTCAAAACGGGTGG
TTAGTAGAGAAGGCAAGGCAGCAGAATGTGTCGGATCGCCCTGCTGGATGGCTCCGGAAGTTGTCATTGCTAATCCTAGTCGGGAAAATAGTTT
CTACGACAACAGAGTGGACGTGTGGTCTCTAGGTATTACCGCTCTAGAAATAGCTGAAGGGCAGGCGCCTTTTCAAAACATGCATCCGACCAGA
GCCCTGTTTCAGATCGTGAAAAATCCCCCTCCCAGTTTGCAAAAAATCAGCAACTGGTCGGATAATTTTCGCGACTTTATCAACGAGTGTCTTG
TAAAATTTTACGAACACCGTCCTTACATAATGGAAGTAATCGACCATCCATTTCTGGGACAAATTCCCGAAAATAATTATCACTTGAGTTTAGA
AATTAAGACGCTTTTGGAGGATATAGAGACTTTCGGTATCCCCAAACGCTGTCCAGAGGTGTCCGTCAAAGGTCGATATTTAAAACGAAACATC
GATGGCAAAATGGAAAAAATGCTTGAGGAAGATTTAACCGATATCGGGCAGATCACGGAGGACAAAATTTTAGATTTGCTTGATGCTAGGACCA
AACAAGGCGAATTCTATAGTTTCGTTGGAGATATTCTTTTGGCTGTTAATCCGAATCAAAAGCTTAATAGATATGATAGTGAGTTTCACGAAAA
GTACGTGTGCAAGTCAAGATCAGATAACGCGCCACACATTTACGCAATTGCCGACGCAGCGATCCAGAACGTTTTGCACCACCAAGTAGCCCAA
CAAATCATATTCACCGGTGAATCTGGATCTGGAAAAACCACCAACTACTTGTACATAATAGACCACCTGTTTCATCTCGCGTCCAATCATCCCG
TGAATTCTGACAGAATCAAGAATGGCATCAAACTGATCCATGCCTTAACGCATGCATCCACTCCATCAAATGATTATTCCACCAGATGTGTCCT
GAAAACGAACGTGACCCTAGGACGGACAGGAAAACTCACTGCGGCAGACTTCAAAGTGATGTGTCTTGATAAATGGAGGGTCTCTTCGGTTGAT
ATGGACCAAAGCAACTTTCACGTATTCTACTATATATACGATGGGATGGTTAATAACGGCTCCATTGAAAAGTACAAACTCAACGCCGATAGAG
ATTACAGATACTTACGTATACTGGAGGCAAACTCCGACTCAAAAAGACCTAGAGATAATGTCGAAGCAAACCTAATCAAGTACAAAAAAATCTA
CAGTTATTTGGAGGAGTACGAGTTCAACGAAGAACAAACGACTACATTTTTGAGCGTGATAGCTGCCATTTTAAATTTGGGAGAGGTGAGATTC
AAAGAAGACGGCAAAGACGGTTCGGCCAAGATACAGAACCAAGAATTTATCGAAAACTTTGCTAGCCTTATGGAGGTTGACGAGAAAAAATTAA
CTTGGGCGTTGACGAATTACTGCCTCGTGAAGCACGGTGACGTCATCAAAAAGAAGAGCACTACGGACGAGGCGAGGGATGCGAGAGACGTGCT
CGCAAATAATCTTTACTGCAGACTGGTAGACTACGTAATCGGAGTGATTAACGATAAACTGGCCATAGGAAAACAAATCTTTGGCGAAAAATTC
ACCATAAAACTACTGGACTATTTCGGTTTCGAGTGCTTCAAGAGGAACCACTTGCCCCAGTTCTTCGTTAATTGCTTCAACGAACAGCTCCAGT
ACCATTACGTGCAAAGAATATTCGCGTGGGAATTACTAGATCTTCAGACGGAAGAAATCGAGTTTAAACAATTCGGTTACGTTGACAATGGAAA
GACGTTAAATCAACTGCTCAGCAAACCGGACGGTGTTTTGTGCATCATCGATGAAGCGTCGCGCAAAAATCTGGACGCTAGATACGTCATGACG

AATATTCAAAAGCAAGAAACTAGTCGAGTTCTGGTAACGGGTTCGTCCGAATTTGCGGTAGCGCATTACACTGGTATCGTTCCATATTATGCCG
GTGAAATGACAGATAAGAACAGAGATTTCCTGCCGCCGGAGTTGATAGAGACGCTGAGGGATTCGGAGAATCCCATAATAAAATTGTTGTTTAC
TAACAAACTAGATAGAACCGGAAACCTCAACGTTCACTTCGAACGCCAACGAAGGAAAGTTGTATATGGTAAAAGATAAACGCTCCGGATCAG
TTTTCTCAAGTAAAACGAATGCGCACCTCGGCGACCATTTACCGGGCGCTATGTTTGGAGTTATTAAAGGAACTCTCGGTGGGAAGTAGTTCAG
GAGGAACTCATTTTGTCAGATGTATCAGGTCTGACCTTAAGGACAGACCTCAGTATTTCAATAGGGAACTTGTCAAGCAGCAGCTGCGGGCCAT
GACGGTGACAGAATCGGCCAGAATTAGACAGAACGGCTATCCTCAAAGGATCAGCTTTTCAGAGTTTTTGCGTCGATATCAGTTCCTAGCGTTC
GACTTCGACGAAAATGTGGAATTTTCGAAAGAAAATTGCCGCCTTTTATTTATAAGGTTAAATATGGAGGGGTGGGCAATAGGAAAATCTAAAG
TATTCCTCAAATACTACAACGAGGAATACCTGTCAAGGCTATACGAAACGCAAGTAAAAAAGATCGTCAAAATTCAAAGCATCATGAGAGGATT
TCTGGCTAAGTGTCGAATAAACAAAAAAATTAAGGATCAAGACAACAAATGCGTTAATGAATGTAAAACTAGGAGAAGTAGCGTTCTGACGCCA
GATGAAGCTGCGGAAATAATACAGAAAGCTTACAGAAGATCTGTCGCGAGAGATTCCAAAAGCGCTTTTGATCATCTAACCGAAGAGGAATGTA
AATTTATTACGCCGTACGCGAAAAAATGGAAATCTCCTTCGTTGTTTCAAGTATTAGTTCAATACCGATCTGCGAGGTTGCATGATTTTTTCAA
CTTTTCTCAGCAGGTTCACCTTTACAATCAAAACGCATTCTATCAATCGCAAAAATTGCAAAAATTCGTCGATTTGAAACATGTCAATGGCAAA
GCC

Protein RF 1: 163->3668 (1168AA)

MKSGLGNVPDPGDRYTFGDLLGTGVFGKVYKAFDNQANQKAVAIKIQKFDQDNKEFVQEEYEILKDFSHIVYLVDFYGVFKRDNEIWFVLEQCE
ANTVVDLVQGLLLKNRRISEEHIAHILKELVKAIIYLHEHNVVHRDIKASNILLTKEGEIKLCDFGLSKRVVSREGKAAECVGSPCWMAPEVVI
ANPSRENSFYDNRVDVWSLGITALEIAEGQAPFQNMHPTRALFQIVKNPPPSLQKISNWSDNFRDFINECLVKFYEHRPYIMEVIDHPFLGQIP
ENNYHLSLEIKTLLEDIETFGIPKRCPEVSVKGRYLKRNIDGKMEKMLEEDLTDIGQITEDKILDLLDARTKQGEFYSFVGDILLAVNPNQKLN
RYDSEFHEKYVCKSRSDNAPHIYAIADAAIQNVLHHQVAQQIIFTGESGSGKTTNYLYIIDHLFHLASNHPVNSDRIKNGIKLIHALTHASTPS
NDYSTRCVLKTNVTLGRTGKLTAADFKVMCLDKWRVSSVDMDQSNFHVFYYIYDGMVNNGSIEKYKLNADRDYRYLRILEANSDSKRPRDNVEA
NLIKYKKIYSYLEEYEFNEEQTTTFLSVIAAILNLGEVRFKEDGKDGSAKIQNQEFIENFASLMEVDEKKLTWALTNYCLVKHGDVIKKKSTTD
EARDARDVLANNLYCRLVDYVIGVINDKLAIGKQIFGEKFTIKLLDYFGFECFKRNHLPQFFVNCFNEQLQYHYVQRIFAWELLDLQTEEIEFK
QFGYVDNGKTLNQLLSKPDGVLCIIDEASRKNLDARYVMTNIQKQETSRVLVTGSSEFAVAHYTGIVPYYAGEMTDKNRDFLPPELIETLRDSE
NPIIKLLFTNKLDRTGNLNVHFERQRRKVVYGKKINAPDQFSQVKRMRTSATIYRALCLELLKELSVGSSSGGTHFVRCIRSDLKDRPQYFNRE
LVKQQLRAMTVTESARIRQNGYPQRISFSEFLRRYQFLAFDFDENVEFSKENCRLLFIRLNMEGWAIGKSKVFLKYYNEEYLSRLYETQVKKIV
KIQSIMRGFLAKCRINKKIKDQDNKCVNECKTRRSSVLTPDEAAEIIQKAYRRSVARDSKSAFDHLTEEECKFITPYAKKWKSPSLFQVLVQYR
SARLHDFFNFSQQVHLYNQNAFYQSQKLQKFVDLKHVNGKA

Comparison with *Tribolium* PREDICTED: similar to myosin IIIA (1109AA)

```
Query  9    PDPGDRYTFGDL----LGTGVFGKVYKAFDNQANQKAVAIKIQKFDQDNK-E-FVQEEYE  62
            P PG+RY    L    LG G FG V  A D QA+ K VAIK+QK  +  K E ++Q EY
Sbjct  7    PSPGERY----LVEDCLGVGAFGSVHSARDTQADNKQVAIKVQK--HTKKFEKYIQHEYK  60

Query  63   ILKDFS-HIVYLVDFYGVFKRDNEIWFVLEQCEANT--VVDLVQGLLLKNRRISEEHIAH  119
            +LKD S H   LVDFYG+F  ++++WFVLE C      V DLVQ LL KNRR  EEHIA
Sbjct  61   VLKDLSWH-GNLVDFYGIFRKEDDVWFVLEIC--SSCCVMDLVQNLLDKNRRMREEHIAY  117

Query  120  ILKELVKAIIYLHEHNV-VHRDIKASNILLTKEGEIKLCDFGLS---KRVVSREGKAAEC  175
            ILKE VKA I+LHE N  +HRDI  SNILLT  G++KL DFG S     V    G   +C
Sbjct  118  ILKEVVKAAIFLHE-NCCIHRDIRGSNILLTNNGDVKLGDFGFSCFLNDVL---GGTNDC  173

Query  176  VGSPCWMAPEVVIANPSRENSFYDNRVDVWSLGITALEIAEGQAPFQNMHPTRALFQIVK  235
            VGSPCWMAPEVV       N  Y NRVDVWSLGITA E  +G AP+Q M P R LFQIV
Sbjct  174  VGSPCWMAPEVVTCKRTKRN--YGNRVDVWSLGITAIELGDGTAPYQSMPPSRILFQIVT  231

Query  236  NPPPSL-QKISNWSDNFRDFINECLVKFYEHRPYIMEVIDHPFLGQIPENNYH--L--SL  290
            NPPP L  K  NWS+N+ DFINECLVK  EHRPY  EVI+HPFL Q+PENNYH  L   L
Sbjct  232  NPPPTLYRKF-NWSENYIDFINECLVKNAEHRPYMVEVINHPFLQQVPENNYHCGLDGGL  290

Query  291  EIKTLLED  298
            E K L ED
Sbjct  291  E-KILAED  297

Query  323  DGKMEKMLEEDLTDIGQITEDKILDLLDARTKQGEFYSFVGDILLAVNPNQKLNRYDSEF  382
            DG +EK L EDL       E+ ++ LL+AR K G+F  F+G+ILL  NPN+K + Y  EF
Sbjct  287  DGGLEKILAEDLASLDSLQEEDVMKLLEARFKSGQFQTFIGEILLILNPNEKKDIYGDEF  346

Query  383  HEKY-VCKSRSDNAPHIYAIADAAIQNVLHHQVAQQIIFTGESGSGKTTNYLYIIDHLFH  441
            H KY   KSRSDN PHI+AIAD A QN LHH++ Q I+  GESGSGKTTN+    +HL
Sbjct  347  HRKYQM-KSRSDNEPHIFAIADSAYQNALHHHISQKIVLSGESGSGKTTNFFHLLNHLIY  405

Query  442  LASNHPVNSDRIKNGIKLIHALTHASTPSNDYSTRCVLKTNVTLGRTGKLTAADFKVMCL  501
            L  N  +N  RI N +KLIH LTHA TP N+YSTRCV K ++  G TGK   A F V  L
Sbjct  406  LGQNDNINLQRIVNAVKLIHSLTHALTPINNYSTRCVFKVDIKFGNTGKVSGAIFNVFQL  465
```

```
Query  502  DKWRVSSVD  510
            +KWRVSS D
Sbjct  466  EKWRVSSTD  474

Query  577   EEYEFNEEQTTTFLSVIAAILNLGEVRFKEDGKDGSAKIQNQEFIENFASLMEVDEKKLT  636
             E++EFN+ +  T LS+  AIL LGE  F E    +   K     E +E  A L+++D  K
Sbjct  482   EDFEFNDSEIDTILSITSAILILGEMSFVEADLESGDK----ECVEKIAQLLQIDPCKFH  537

Query  637   WALTNYCLVKHGDVI-KKKSTTDEARDARDVLANNLYCRLVDYVIGVINDKLAIGKQIFG  695
             WAL NYCL+K  DVI  KK T DEA   RD LANNLY RLVDY++   IN+ L  G  IFG
Sbjct  538   WALANYCLIKK-DVIIRKKNTEDEAKSVRDALANNLYLRLVDYIVNTINNRLSAGRKIFG  596

Query  696   EKFTIKLLDYFGFECFKRNHLPQFFVNCFNEQLQYHYVQRIFAWELLDLQTEEIEFKQFG  755
             E + +   LDYFGFECFK N L Q FVNCFNEQ++Y Y QR F WE LD+  E+   F
Sbjct  597   ETYSVQILDYFGFECFKENYLSQLFVNCFNEQMHYYYTQRNFYWEYLDMKDEDLNFNTSS  656

Query  756   -YVDNGKT-LNQLLSKPDGVLCIIDEASRKNLDARYVMTN-IQKQETS--R-VLVTGSSE  809
              Y DN K  L++LL KP+GV  IIDE S  N +    V  N I   E S    V V G  +
Sbjct  657   NYYDN-KSCLDELLGKPEGVFSIIDEVSKMNQNEKHVI-NFI---ENSDLKFVKVVGNLD  711

Query  810   FAVAHYTGIVPYYAG-EMTDKNRDFLPPELIETLRDSENPIIKLLFTNKLDRTGNLNVHF  868
             FAVAHYTG V Y  G +  +KNRDFLP E IETLR S+NP +KLLFTNKL+RTGNL V
Sbjct  712   FAVAHYTGTVTY-KGCDICEKNRDFLPAEVIETLRLSDNPTVKLLFTNKLNRTGNLIVD-  769

Query  869   ERQRRKVVY---GKKINAPDQFSQVKRMRTSATIYRALCLELLKELSVGSSSGGTHFVRC  925
                R K  Y    KK     +Q+SQ+K + TS    + AL  ELLK+LS G      THFVRC
Sbjct  770   APDRSK--YKFTSKKL-THNQYSQIKHLTTSLRKFKALGVELLKDLSKGC----THFVRC  822

Query  926   IRSDLKDRPQYFNRELVKQQLRAMTVTESARIRQNGYPQRISFSEFLRRYQFLAFDFDEN  985
             +R DL    P  F+  LVKQQ RA+ V E A  RQNGY  R SF EFLRRY FLAFDF+EN
Sbjct  823   VRTDLHQVPKNFDCGLVKQQIRALEVVETAKLRQNGYSYRTSFHEFLRRYKFLAFDFNEN  882

Query  986   VEFSKENCRLLFIRLNMEGWAIGKSKVFLKYYNEEYLSRLYETQVKKIVKIQSIMRGFLA  1045
             VE  KENCRLL IRL  EGW +GKSKVFLKYY EEYL RL+ETQVKKI+KIQSI+R FLA
Sbjct  883   VETTKENCRLLLIRLGVEGWDVGKSKVFLKYYVEEYLTRLFETQVKKIIKIQSILRRFLA  942

Query  1046  KCRINKKIKDQDNKCV-----NECKTRRSSVLTPDEAAEIIQKAYRRSVARDSKS--AF-  1097
             KC + K    +    KC+         E K R     +T +EAA IIQKAYR S      KS    F
Sbjct  943   KCLVTKHLQNKGEKCILGHVQAE-KYR----MTEEEAAIIIQKAYRESTVK--KSYQDFA  995

Query  1098  -DH--LTEEECKFITPYAKKWKSPSLFQVLVQYRSARLHDFFNFSQQ  1141
              D   L EE C FI  +A KWK    F +L  Y S R +D FNFSQQ
Sbjct  996   EDYKILDEETCGFIRKFALKWKNNTVFRILFLYKSVRHQDCFNFSQQ  1042
```

Graphical representation

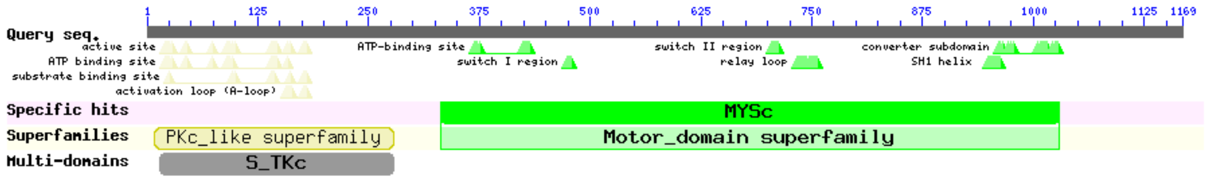

<u>dsRNA uptake in *Cylas brunneus*</u>

<u>CG4966=orthologous to the Hermansky-Pudlak Syndrome 4 (HPS4)</u>

>Cb.comp43091_c0_seq27 len=5468

<u>cDNA</u>

```
TCGCACTTGACCCAAAAAACGCCTGAAACGCGCGCGCCGCCCCGTATTCCTGGTGTCGTCAAAGTCCAGCAGGGTGCTGTCGTACACCGTCCTC
ACCCTCAGCTGTCTCTTGACGGCCGAAATTAGGGGCGGCCTCTGAAACAGCGCCCCCTTTAACTTCTCGAGACCCTGCGTGATCTTTTTTATGC
CCCCCTCCACTTGCGAGTGCAGCTTGAAGACCGTGACATACTCTTTGCCGGCGGACTGCTGCGATTTGACCAACCGGGTGGCTCGTTCGACGCA
CACTATGAGACAACCGGTGGTTTTGGGGTCGAGCGTGCCCGAGTGTCCCGTCTTTTGCACCCCCAGTATGCGTTTGATCCACGCGACCACCTCG
TGACTGCTCGGATTGCTCGGTTTGTCCAGGTTTATGTAACCCGACTTTATATACTCGGAGAGTTCGCGCCTCAGGGGCGACGAGCCGCACGGTA
TCGGCGTGTAATGGTTCGTCCGCACGTTGAGGCGGTCAAAGTGCTTTAAGAGGAGAGGCCATTGCGACGTGTCTAATTTGGCGATTTTTTCCGA
CGACTGCAGCTGAAAGCTACTCTCCTTTTGCAACTGGCCGAGCTTGTCCGTTTTGCTCTTTTTCTTCTTCTTCTCCGTCAACTCCGCGTTTTCC
ATCGTCGAACGTTGTAACGCACGTGTTTTCGTCCTCGCACGAAACTTGCGCCGACAGTTGGGTTATGTTTGGTTTCGACCGCCCGCGCCATCTA
CGCAAAAGCGCACGCCATCGCGGACAACAAAACTAGTTTCCGTGGCGGGCTTGAATTGAGGTTATGGTGTCGTAAATATTGACTTTTCGCGTCG
GGACCGGATGGAACTCGAATCGAGCCCGTTTTCTGACTAGGGGCGACTCGAAACGGCCGCCGCGATGGCCAAGGAAACGACCATCATATTCGTC
TACGACACGCAGAGGCTGAGCAAGGAGGACGACGATCCGGCGCAGGCGATACTTTACTTCCACCCGACGTGGGTGTCGGATCAGCAAAAAACCG
CGCTTTGCGGCCAGATAGTCGGCACGATAATGTGCGCGAGGTCGATCTTCCGCATGCCGCGCGTCGTCGGCCTGCAGACCGGGAAGTTTTACGT
CATCGAAAACGGGCGCTACATTCTAGCCGTCGGCACCGACAGGAACGTGGCCGATTGGCTGCTCGAGCACCGCGCTACCCTGCTCTACTCCCTG
GTTAATTTTTTTAACAGAGACTTTGAGACGCTCGGCAAGTTATACCAAAACGAGGCGCTCACGGCCAAACTCTACCACCTGTTCGAGACATATC
TCAGGATGCTGTTCTTCGGGGGCAATATTTTCAGTCACGTGCCCCTCCTGTGTCTCCCCAAGAGCGCCAGCAGTGTGTTCACCGAAGCGATGAC
GTTCCTCGAGGCGTGTCAACAGTGCCCGCACGTCTTAGGCGGCGCCCTCTTGTACCATAACAAGGTCGTCGCCACCCAACTGACGACTGACGTT
ACGAAACGCGTCGTCATAGCCGACCCTTACCGCATCAAGTCGCCCGCGGAACCGGCCCCGGCCTCCTTCGATCTCCCGCTCGGCGTGCAGCTGT
TGCAGGTTTACGTCAGCAACGCGGCCTTCTGCGACCTGCTCGAGGCGTCGCTTCGCAACGAGTGCGTTTTCCAGTATTTAAACAACAAGAGCGT
CAAAACGCCGCACGGCGACGACAAGGTCTGCGCCCCCGCCGTCATGAAACGCGACCAATCTTTGCTGTTCACGGCCGTGCCCGAAGAGGGCCCG
GCCGAGATCGCGTTCGTCCCGAGCCAGAAAAAACAGCGCCCCAAGTTTTTGAACTTGAGGAGTTTCAGCGCGGAGTCGTCGAGGGCGACGCCGG
TGATGACGACGCCCGCGTGCGGTCAGACTAGCGTCGCGAGCACCCCCATGACGGAACTGAGACGGTTCGTGCACCAGAACCCGTTGACGCTGAT
CGAGAAGGAGGGGGAGCCCGAAAACGACGCCCGCGCGACTCAACGCGGGGCGTCGCCCCCCATGCGTCGCAGCACCAGCGTCCTGCACTTGAGG
GAGGTGCTGAACGGGATCGTGAAGGACGTAGATCTGGGGCGTTTCGACGCCAAAACATGGTCGGGGTCGCCGAAGCGCCGCGTGCCGCGGCGCT
CCCGTTCCATCCACGACCCGACGGTGCCCCTCGTCAAGCCGAACGGCACAGCCCTCTCGAAGGGCCACTACGAGCACCTGCTCGCGCTAGACGG
TGAGACTCTGCTAAGCGTCCCGCGGGCGCCGCTCGGGGACGTCGCCGCGAAGCCCATGGACAGGAACGCGACGCCCCCCAGGGACCGCCCGGAC
GTCGTCGCCGTCGACAAAATCCCGAAGGAGCGTCGGCGGTCCCTCGTCCTGCCCCTGAAACCGATCAGCGGCGACGCCGGTTCGAAACCCGAAC
GGAAACCTTCTCTGGGGGTCCAGCTGACGCCCCTCATGTCGAAGCTGAGCGTTTTGGCGTTCGAGGAGCAACGCCGCGTAAAAGACGCGCCCCC
GCCTCCCCCGTTTGCCGCCCCCAAAACCAAGGAGGTGAAAAACTTAAGGTTAGAAGACGGGTCAGCGGCGCGCAGGTGCGTGCTCTTCGTCTGC
GGGCAGCAGGACGTGGTGGCGTTGCTCCTGATGAAGGAGGAGTGCTGCAAGGACGAGGACGCCGTCGTCCGGATCTGGGAGATGTGCACCGAAC
ACCTGGGGCAGCTGGGGAAGCGGCTCAGCTTCTGCCTCGAGTCGTCGGGGGCGGGCGTCCACGAGAGCGAGCCCTACAGTTTCCTGTGCCTCGA
CGCGAAGTGGGACACGGTGAAGAGGGGCGGACCGTGGGGCACGGGCGACCTGTCCTCGCTGGTTGCCCTGCACCGCGATTTCGTCGACGCGCCC
GACATTACCGAGATTCTGTTAAGGAGCTCGGACTGCGCCGTTTACGGGTACCGATGCGGCCCGTCGGAGGTTTTCTACCACGAGGGCGCCGGCC
CGGCTGCGGGGCTGCCCCTCCCGTCCGACCCCGTCGGTCTCGTCCAGACGAAAGCGAGGCGACGCCTCGAGAGGGACCACGCGGTCGTCTTACT
CTAAATAAGGTCCGCCATGACGTAATAATACTCGAATGCGCATGTCTTAGAAGGTCGTTAGTTGTGTTTTTTAGGTTAGGTTCGCTCAGGTTAG
GTTTCGACGTTGTCTGTCCGTAGGTTTAGCGTAGGCACGCGACGGGGGCGCCTCCGGCTCTCATACTTATCAGTGCCAAAGCGAATATCGCGCC
CTTCGTCGCCAAGGGGCCCCCCGTCGGTAGACGTTCCCTTACCTCCATTTTTGTTGTTTTTTTTTATTAATTTATTTATAGAAGTAGCAGGAAA
TAAAATTGGTCGTTTCTTATCTAGCGTATTTATTTAAACTAAACTTGATCGATAAGCTTGAAGTTTTGATGCTGCTGCTTGGGCAGCACGTTAA
AGTACTTCTTGTAAAAGTCGGTGCCTTTCCTGAAACCGTGGCACCTTTGCACCCACGTGTGATCCCAATTCAGGTCGACTTTCTCGCACGTCAC
CAAAACCTTGCCCGGCACGATGGCGAACAGCGTGCCGTTCCGTCCCAAACCAACATTCAGGCCCGGGTGGAACCTCAAATTTCTCTGCGTCACG
AGGATTTTACCGGCCGGCACCTCGTGCCCGTCCAGCACTTTCCATCCTCTGTGCTTCGGTCTCGACTTGGGGCTGTTGTTGCGCGTACTGCCGC
TCGTTTTCTTGCTCGCGTGTCTCACGCAACTTATCAGAGTTTTTGCGGTTTCGAGAAACGAATTGCCGAAAAAATTCATTTTACAGTTAAGTAA
TCACGGCATAACCCCAAAAAGCAGGGTCTCCGCGTTTGGATATTCTTTGACTACAGCCCATCTATTTCTGCCATCTGTGGTCCGTAAAAGAAGA
GGCCAATAAACCTAAGAAGAATACAAAGTCGCAAAGAAGAAGATTCCGTTTATAACCCCGCTTGTTAACCGAATTTGCGCTTGTGAAAAGTTAA
ACCGAATAACGTTATGATAGCGACAACGGCGGAGCCGGATTTTAAACCGCTGGACGCTTTCAAGTTGACGGGACACGCTGGGACGAATTTCGCG
TGCTCGTACTCGCAAGACGACCGGTTTTCGTTGGTGGCAGACAACGGCGTCTACGTGATGGCTCTGAAACCCGACACGGGAAGTAGTTTCCAGT
CGTTCGGCTTCACGAAAACGTTCGTAAAACCGTCCGCGTACAACGTGTGCGACAACGTCGGCATGGACGTAAACGATTTCCTGTACGATCTTCC
GAAGGAAGACATGTACGCGGCCGTGCTTCAGGTCGACCTATCGTCTAACTTGCGCAACGCGCAGGCAGTAAATTCCGTGCCGCGCCGCGCCGAT
TGGTCCCCTAACGGTGTGGTGCGTAAAAGCAATTGCCTGCTCGCCGTGTTGACGAGTTTGCACAGTTTGGAGGTCTATTCGATGCACGTCGACG
AAAACATGATGACTCGTTACGAATTGATTGTAAACGTCGCGGAGCGGATCGCGGACGCCGAGAAACCGGCCTACAAAAAAGGCAACAGGATCCC
CAACGCCAAGGCCAAGTTTACCGAGTTTAAGAGGCGAGTAGAACGCGTCGCGCCGTCAGCTTTCTGTTGGTCTCATTCGTTTACCGTCGACGAT
CGAGCGTGTTCTGTCATTTTCGTCGGCCATTTCGACGGGTCCGTGACTTTTTGGAGGGTCTGCGCGGCGGACGATCGCAACGTCGAATGTCGTT
TGTTGGGCCGTAGTGCCACGAAACTGGGCGCCGTCAGCGCCGCCTACTGGCACCAAACCAAACCGTTTGGCGGCGGTTTATGTTTGGGCGACCG
GAACGGGAGGGTCTCCATTATCCGCGTGGAGAATTTGGAGGGCGAAGAGGTCGCCGCCGGCGACGAGGTCGAACTGTGGGTCGACGCTGACGTG
GCTGTAGATCAAATTTTGGTCACGTCGTTCGATAAATACACGCTAATCGTAGTCATCAAGCAGTGCCACGTGTTGTTCTTCGGACTCGACAAAA
CGGGCGCCATTTTCGATTTCGTCGCGCACAACGTCGAGCATTATTACGTAACAGGCGTCGCGTGTTTCGGCAACGTCCTGTACGTCCTCACGTT
```

CACCGGCACGCTTAAAATAATCACAATCTCCGTCAAACTGCACGACATCGTCGTCGAGGAGGCCGTCGCGACGCTCAAACTCGACTCGAAGCGG
CTGCGCACCCACGGCCTCGTGGTCTCCCCGAACCGGGCGTTTTTGGGCGTCGCCTCGCACCCGTACCACCAAAAGGACCTCACCAACGGCAAAC
AGTTCATCAACGTGCACTTGTTTCACGACGCCAACCTCAAACCGTTGCGCGTACTGCTAGACAACCCGCGAAACACCGTGCGACACGTCTGGGA
TTGCTTCGAAGTGCTC

Protein RF 2: 911->3106 (731AA)

MAKETTIIFVYDTQRLSKEDDDPAQAILYFHPTWVSDQQKTALCGQIVGTIMCARSIFRMPRVVGLQTGKFYVIENGRYILAVGTDRNVADWLL
EHRATLLYSLVNFFNRDFETLGKLYQNEALTAKLYHLFETYLRMLFFGGNIFSHVPLLCLPKSASSVFTEAMTFLEACQQCPHVLGGALLYHNK
VVATQLTTDVTKRVVIADPYRIKSPAEPAPASFDLPLGVQLLQVYVSNAAFCDLLEASLRNECVFQYLNNKSVKTPHGDDKVCAPAVMKRDQSL
LFTAVPEEGPAEIAFVPSQKKQRPKFLNLRSFSAESSRATPVMTTPACGQTSVASTPMTELRRFVHQNPLTLIEKEGEPENDARATQRGASPPM
RRSTSVLHLREVLNGIVKDVDLGRFDAKTWSGSPKRRVPRRSRSIHDPTVPLVKPNGTALSKGHYEHLLALDGETLLSVPRAPLGDVAAKPMDR
NATPPRDRPDVVAVDKIPKERRRSLVLPLKPISGDAGSKPERKPSLGVQLTPLMSKLSVLAFEEQRRVKDAPPPPPFAAPKTKEVKNLRLEDGS
AARRCVLFVCGQQDVVALLLMKEECCKDEDAVVRIWEMCTEHLGQLGKRLSFCLESSGAGVHESEPYSFLCLDAKWDTVKRGGPWGTGDLSSLV
ALHRDFVDAPDITEILLRSSDCAVYGYRCGPSEVFYHEGAGPAAGLPLPSDPVGLVQTKARRRLERDHAVVLL

Comparison with *Tribolium* PREDICTED: similar to CG4966 CG4966-PB (908AA)

```
Query  1     MAKETTIIFVYDTQRLSKEDDDPAQAILYFHPTWVSDQQKTALCGQIVGTIMCARSIFRM  60
             MAKE  II VYD+Q L KE+DDPA AILYFHPTWVSDQQKTALCGQ++GT+ C +SIF
Sbjct  1     MAKEMMIILVYDSQMLQKEEDDPASAILYFHPTWVSDQQKTALCGQLMGTVHCVKSIFSA  60

Query  61    PRVVGLQTGKFYVIENGRYILAVGTDRNVADWLLEHRATLLYSLVNFFNRDFETLGKLYQ  120
             P++V LQ+GKF++ E GRY++AVGTDRN+ADWLLEHRA   + SL++FF++D E + KLY
Sbjct  61    PKIVSLQSGKFFIKEYGRYLMAVGTDRNIADWLLEHRANTMSSLISFFHQDIEIMSKLYD  120

Query  121   NEA-LTAKLYHLFETYLRMLFFGGNIFSHVPLLCLPKSASSVFTEAMTFLEACQQCPHVL  179
             N A L+AKLY LFETYL+ +F GGNIFS+ P L LPKSAS+VF EA+  L+ CQ+  +V+
Sbjct  121   NSAKLSAKLYQLFETYLKYMFLGGNIFSYTPSLKLPKSASNVFLEAIQILQCCQELNYVM  180

Query  180   GGALLYHNKVVATQLTTDVTKRVVIADPYRIKSPAEPAPASFDLPLGVQLLQVYVSNAAF  239
             GG LLYHNKVVATQL++D+TKR+V+ DPYRIK PAE    +F+LPLGVQLLQVY+S+  +
Sbjct  181   GGTLLYHNKVVATQLSSDITKRIVLTDPYRIKCPAETPSVNFELPLGVQLLQVYISSKEY  240

Query  240   CDLLEASLRNECVFQYLNNKSVK--TPHGDDKVCAPAVMKRDQSLLFTAVPEEGPA-EIA  296
               L E + R+  +FQYL++KS+K   P    +    A MKRDQS++FTAVPEE    +I+
Sbjct  241   YKLHEEATRSRSIFQYLSSKSIKKGKPVASKEPVISA-MKRDQSIIFTAVPEEDSEPQIS  299

Query  297   FV--PSQKKQRPKFLNLRSFSAESSRATPVM-TTPACGQTSVASTPMTELRRFVHQNPLT  353
              V  P   + RPKFLNL+  + +  +  PV  +TP  GQTSV STPMT+L + +H  PL+
Sbjct  300   KVEKPISSQNRPKFLNLKHKTTDEKK--PVAPSTPFHGQTSVCSTPMTDLSKVLHSKPLS  357

Query  354   L-----IEKEGEPENDARATQRG-------ASPPMRRSTSVLH----------LREVLNG  391
             +     I  E  PE  +   + G       A  P    TS LH          +RE L
Sbjct  358   ICINETIPCEKTPEKTSIFAKNGTDLNSVIARVPYLTVTSNLHDCKKFSSVFDVREKLKS  417

Query  392   IVKDVDLGRFDAKTWSGSPKRR--VPRRSR---SIHDPTVPLVKPNGTALSKGHYEHLLA  446
             + + + +  ++++            P  SR   +I DP  P+ + +GT +S   Y+  L
Sbjct  418   LDRGITMKYYNSEFREKYKISHDVTPDESRVFKTITDPNYPIFRSDGTVVSHPFYQDYLT  477

Query  447   LDGETLLSVPRAPLGDVAAKPMDRNATPPRDRPDVVAVDKIP----------------KE  490
                E + S  +    D++    D   +   D   V +V KIP                KE
Sbjct  478   SQMELITSEIKEEKPDLSHHSFDDFDSNLGDF--VQSVKKIPDKVVESIEFSALPRPSKE  535

Query  491   RRRSLVLPLKPISGDAGSK---PERKPSLGVQLTPLMSKLSVLAFEEQRRVKD-------  540
              R+SL LPLK +S D G +   P R+ S  V LTPLMSKLS  +FE
Sbjct  536   HRKSLTLPLKSLSVDGGGEEVSPMRRHSSSVLLTPLMSKLS--SFESSGFCSRDTTPIFT  593

Query  541   -APPPPPFAAPKTKEVKNLRLEDGSAARRCVLFVCGQQDVVALLLMKEECCKDEDAVVRI  599
                P  PFA  + K++    L +  + R+CVLFVCGQQD+V  LL+++E C   + + ++
Sbjct  594   PTQPNFPFAFKRPKKL----LPETDSLRKCVLFVCGQQDMVVTLLLQDEACASLELLTKL  649

Query  600   WEMCTEHLGQLGKRLSFCLES--SGAGVHESEPYSFLCLDAKWDTVKRGGPWGTGDLSSL  657
             WE+CTE+LG+L K+L  CLE+   G    ++EPYS+L LD+ WDT+ RGGPWG+ +L +L
Sbjct  650   WEICTENLGKLEKQLHHCLETYPGGGAPGDTEPYSYLYLDSDWDTIHRGGPWGSAELGAL  709

Query  658   VALHRDFVDAPDITEILLRS  677
                HRDF ++  + EIL+ S
Sbjct  710   TYFHRDFQESSSLIEILMSS  729

Query  679   DCAVYGYRCGPSEVFYHEGAGPAAGLPLPSDPVGLVQTKARRRLERDHAVVLL  731
             D  +YGY+CG SEVFYH+ A P AGLP P+DP+  V  KARRRLERDH+++LL
Sbjct  856   DGVIYGYQCGKSEVFYHQAANPNAGLPTPADPMSNVPLKARRRLERDHSIILL  908
```

Graphical representation

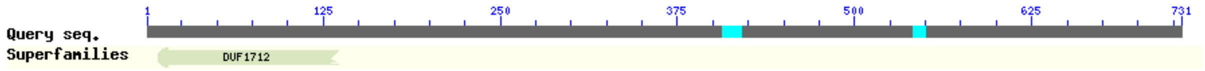

FBX011 ortholog

>Cb.comp41779_c0_seq1

cDNA

TGTTGTTGCTGCTGCTGCTGCTGTTGGAGTTGCTGCTGCTGCTGCTGGTAGACGTGGTGCGGTGGCGGTACCGGGCTGAGCACGCGCGGGCTCA
CCCCGTATCCACCACCGCCCGTGTTGTTACCGTGCAACTTGAGTATCGATATTAACATATCCCGATGACGGGGCTGCAGGGCAGGATTCGCCAG
TTGCTGGTGCAGATGCTGAGTGGTGATCTCATACCGTTTCAGACCTTGAAGGATAGCCTGCGCTTCGGGCCTGTTGAGCAGCTCCCTGCTCTGC
TGCACGCCCATGTCCATCAGCGGCGAAGCCCTCATCTGCGGCGGCACCCCCGCATTGATCAGTTTGTTGAGCATCTCCATTTGCTGGTGTTGCA
TGGCGGCAGCGGCCGCCGCCGCAGCTGCCGCGGCCGCCCATCAGTCGCCGTACGATTTGCGGCGCAAGTCGCCGTCGCACAATCACGGGGGCGG
CGCGCCCGCCGTAGACGGAGGTCCCGGGCCCAGCACGAGCGCCGCGACGGCCAGCGGCAGCCCCGCCTCCCCTACGACTCCTTGCAACGTGCCT
GTCGCGCAGGGCGGGTACAGTTCCGTCATGCAACCGGCGAGGAAACGGCCCAGGCGGACCTGCTCCGCGTCGTACGAGAATTGTACAAATACGG
CCGCTCATTATTTGCAATACGAATTGCCCGACGAGGTGTTGCTCACCATATTCAACTACCTGCTCGAGCAGGACCTGTGCCGCGTGAGTCAGGT
GTGCAAGAGGTTCCAGGTGATCGCCAACGACACCGAGCTGTGGAAACGCCTGTATCAGAACGTGTACGAGTATGATTTGCCGCTGTTCAACCCG
TCCCCGTGCAGGTTCGAGTTTGTCAGCTCGGACGAGTCGGAGTTGGCAAATCCGTGGAAGGAGTCGTTCCGGCAGCTGTACCGGGGCATACACG
TGCGGCCCGGCTATCAAGAGTTGAGCTTCAAGGGGCGCAATTTGGTTTACTTCAACACCGTGCAGGGCGCCCTAGACTACGCTGACGAGCGAAG
CGGCAGCGGCTCCTCCACGGGCCTGGTCGCCGGCACGTGCGGAAGCCACCCGCCGGACACCGGCGCTCAGGGGGCGTTGATTTTCCTGCACGCG
GGCACCTACCGCGGCGAGTTTCTTGTCATCGACTCGGACATCGCGCTGATCGGTGCTGCGGCCGGCAACGTTGCCGAATCGGTCATACTGGAGC
GGGAGTCGGAATCGACCGTCATGTTCGTCGAGGGCGCGAAGAACGCGTACGCGGGCCACCTGACCTTAAAGTTTTCCCCCGAACCCGCCTCGGC
CACGCCCCACCACAAGCACTACTGCCTCGAGGTCGGCGAGAATTGTAGCCCCACCATCGACCACTGCATCATCAGGAGTACGTCGGTCGTTGGG
GCGGCGGTGTGCGTGAGCGGCGTCGGCGCCAACCCCGTCATCCGGCACTGCGACATCAGCGACTGCGAGAACGTCGGTCTGTACGTCACCGATT
ACGCGCAGGGCACCTACGAAGACAACGAAATAAGCCGGAACGCGTTGGCCGGCATCTGGGTGAAGAATTACGCGAACCCGATCATGCGACGCAA
TCACATCCACCACGGACGCGACGTCGGCATCTTCACCTTCGACAACGGCCTCGGTTACTTTGAGGCGAACGACATCCACAACAACCGGATCGCC
GGTTTCGAGGTGAAGGCCGGCGCGAACCCGACCGTCGTCCACTGCGAGATCCACCACGGCCAGACGGGCGGCATCTACGTCCACGAAAACGGCC
TCGGCCAATTCATCGACAACAAGATCCACTCGAACAATTTTGCCGGAGTGTGGATCACCTCCAACAGCAACCCCACCATCAGGCGGAACGAGAT
ATACAACGGGCACCAGGGCGGCGTCTATATCTTCGGGGAGGGGCGCGGCCTCATCGAGCACAACAACATCTACGGCAACGCGTTGGCCGGCATC
CAGATCCGCACCAACAGCGACCCGATCGTCAGGCACAACAAGATCCATCACGGGCAGCACGGTGGCATCTACGTCCACGAAAAGGGCCAAGGCC
TGATCGAGGAGAACGAGGTGTACGCGAACACGCTCGCCGGCGTGTGGATCACGACGGGCAGCACGCCGGTGCTGCGCCGCAACCGCATCCATTC
GGGCAAGCAGGTGGGCGTGTACTTTTACGACAACGGCCACGGCAAGCTCGAGGACAACGACATCTTCAACCATCTGTATTCGGGCGTGCAGATC
CGGACCGGCAGTAATCCGGTGATTAGGGGCAACAAAATATGGGGCGGCCAGAACGGCGGCGTGCTCGTCTACAACGGCGGCCTCGGCCTCCTCG
AGCAGAACGAGATCTTTGACAACGCGATGGCCGGCGTCTGGATCAAGACCGACTCGAATCCGACGCTCAAGCGGAACAAGATTTTCGACGGCCG
CGACGGCGGTATATGCATTTTTAACGGCGGCAAGGGTATCCTCGAGGAAAACGACATATTTCGTAACGCGCAGGCCGGTGTGCTCATTTCGACC
CAGAGTCACCCGATCCTCAGACGTAACCGAATATTCGACGGTCTGGCGGCCGGCGTTGAAATCACAAACAACGCGACCGCGACCCTCGAATTCA
ATCAAATTTTCAACAACCGTTTCGGCGGGTTGTGCCTGGCTAGTGGCGTGCAGCCGATCGTGCGCGGCAACAAGATATTCAGCAACCAGGACGC
GGTCGAGAAGGCGGTCGCAAACGGCCAGTGCCTGTACAAAATCTCGTCGTACACGTCGTTCCCGATGCACGACTTTTACCGGTGCCAAACGTGC
AATACGACCGACCGCAACGCCATCTGCGTCAACTGCATCAAGACGTGTCACGCGGGCCACGACGTCGAGTTCATCAGGCACGACCGGTTCTTCT
GCGACTGCGGGGCGGGGACGCTGACCAACCAATGTCAGCTGCAGGGCGAGCCCACTCAGGACACTGACACGTTGTACGACTCGGCCGCCCCCAT
GGAGTCGCACACGCTGATGGTTAATTAGATGCGGCGGGCGCTACGTCGGTTCCAGGTGTTCCTGTTTCTCGGACTCTCGGGCGGCGGACGCCCT
TGAGCGGGGCGCCCGTCCGCCGCCGCGCTACTATTTATGAAATATACATATATATATAACGTGCGTGTGTCGAGCGCGTGTGCGGTGGGGGGCG
TGGCTCCTACCCAATGAATCTCGTCGAAGTCTTGTACAGGCAGTACGATTCGATAGTTTTGTCGTTTTTACTATTATCACTGTTTAAGTTTATA
CACGCAAGTGTTCGTTGAGAGCGAGTTGTTTGTTGTGCTATTATTATTATTTTTATTATTGTTCGCCGGGCAACAAATCCGAATAGAATGTTCA
AGTAGCAAAAACGGTACCGATTGATTATTATTTATTGCGTACCGATTAAAATCGGCTCGCCACGTGTTGACCACGGGAGGAGATGATATGGCTC
CACTTTTATAATAGTTACTTGTCGTTTTGATACTTTTAGCTCTTATTTATATTATTTTTCAATTTAAATGTGAATTTGGTTCTAGAAGCTGCAG
CCCGCAGGACGACGGTTTGTAGCGCGTCGTGTACGTGGTGTACATTATTCTAGTTCAACTAGTGGCAGGCCACCACAAAAATTATATTGCTTTT
ATTCCCCAGTAAACTAGCGAAAACGTTGGCCTGAACATCAGATTATGTTCGTCATTTTTTTGCACTTTGCTTCCACGTCCGCAGGAGACGTCCT
CGTCGTCGGTACCATGAATCGTACCGATCGTTTGGAGAGTGACTATTATTGTTGATTGGTTTGGAAATCTTTAAGAGTTGTGTATATAATTTCT
TGACCCCGGCTACCTTTTTTGTTAATTTCATTTATCGCGTGCTAGGGTGAGAGTCGGGTTCGAGCCGCCACTGCGCCACTTTTGTGGCTCAAAA
AGGGCACCCGAACCCGTTTATTGTTTTGATACGACTGAAGATGTGTGCGTGTATAATATCTTGTAAAATATCGTCTTTTTCTATTAATAAACCC
CCCCTCTAATTAATAACGAAAATTATAATTAACTATACTTAAGAGTTTAAGTGGTTAGGCTTAAGTGAACCTTATATGTCGGGCTTTAAATAAT
AAACAACAACAAAAAATAGGATCTACTGACGAGACAGTGATCGTTCAAAGTAGATAGCTAGGCACGTATTGTACTGTGTACATTATTATATACA
AACAAAAAAATATATATGGACAAAAATTTTGGAAAAAAAAAAA

Protein RF 1: 292->3036 (914AA)

MSISGEALICGGTPALISLLSISICWCCMAAAAAAAAAAAAAHQSPYDLRRKSPSHNHGGGAPAVDGGPGPSTSAATASGSPASPTTPCNVPVAQ
GGYSSVMQPARKRPRRTCSASYENCTNTAAHYLQYELPDEVLLTIFNYLLEQDLCRVSQVCKRFQVIANDTELWKRLYQNVYEYDLPLFNPSPC
RFEFVSSDESELANPWKESFRQLYRGIHVRPGYQELSFKGRNLVYFNTVQGALDYADERSGSGSSTGLVAGTCGSHPPDTGAQGALIFLHAGTY
RGEFLVIDSDIALIGAAAGNVAESVILERESESTVMFVEGAKNAYAGHLTLKFSPEPASATPHHKHYCLEVGENCSPTIDHCIIRSTSVVGAAV
CVSGVGANPVIRHCDISDCENVGLYVTDYAQGTYEDNEISRNALAGIWVKNYANPIMRRNHIHHGRDVGIFTFDNGLGYFEANDIHNNRIAGFE
VKAGANPTVVHCEIHHGQTGGIYVHENGLGQFIDNKIHSNNFAGVWITSNSNPTIRRNEIYNGHQGGVYIFGEGRGLIEHNNIYGNALAGIQIR
TNSDPIVRHNKIHHGQHGGIYVHEKGQGLIEENEVYANTLAGVWITTGSTPVLRRNRIHSGKQVGVYFYDNGHGKLEDNDIFNHLYSGVQIRTG
SNPVIRGNKIWGGQNGGVLVYNGGLGLLEQNEIFDNAMAGVWIKTDSNPTLKRNKIFDGRDGGICIFNGGKGILEENDIFRNAQAGVLISTQSH

PILRRNRIFDGLAAGVEITNNATATLEFNQIFNNRFGGLCLASGVQPIVRGNKIFSNQDAVEKAVANGQCLYKISSYTSFPMHDFYRCQTCNTT
DRNAICVNCIKTCHAGHDVEFIRHDRFFCDCGAGTLTNQCQLQGEPTQDTDTLYDSAAPMESHTLMVN

<u>Comparison with *Tribolium* hypothetical protein TcasGA2 TC010102 (915AA)</u>

```
Query  31   AAAAAAAAAAAHQSPYDLRRKSPSHNHGGGAPAVDGGPGPSTSAATASGSPASPTTPCNV  90
            A   +  A  AH SPYDLRRKSPSH+          GPGPSTSAATA GSP SP TP
Sbjct  66   APGTSGGAIPAHHSPYDLRRKSPSHH---------DGPGPSTSAATA-GSPTSPATPT--  113

Query  91   PVAQGGYSSVMQPARKRPRRTCSASYENCTNTAAHYLQYELPDEVLLTIFNYLLEQDLCR  150
                 GY+S M PARKRPRRTCSASYENCTNTAAHYLQYELPDEVLLTIFNYLLEQDLCR
Sbjct  114  -APAQGYTSAMLPARKRPRRTCSASYENCTNTAAHYLQYELPDEVLLTIFNYLLEQDLCR  172

Query  151  VSQVCKRFQVIANDTELWKRLYQNVYEYDLPLFNPSPCRFEFVSSDESELANPWKESFRQ  210
            VSQVCKRFQ IANDTE+WKRLYQ+VYEYDLPLFNP+PC F+F+S +ES+LANPWKESFRQ
Sbjct  173  VSQVCKRFQAIANDTEIWKRLYQSVYEYDLPLFNPAPCVFQFISPEESDLANPWKESFRQ  232

Query  211  LYRGIHVRPGYQELSFKGRNLVYFNTVQGALDYADERSGSGSSTGLVAGTCGSHPPDTGA  270
            LYRGIHVRPGYQ+L+FKGRNLVYFNT+Q ALDYADERSGS
Sbjct  233  LYRGIHVRPGYQDLTFKGRNLVYFNTIQAALDYADERSGS--------------------  272

Query  271  QGALIFLHAGTYRGEFLVID-SDIALIGAAAGNVAESVILERESESTVMFVEGAKNAYAG  329
              ALIFLHAGTYRGEFLVID SDIALIGAA GNVAESVILERESESTVMFVEGAKNAY G
Sbjct  273  --ALIFLHAGTYRGEFLVIDDSDIALIGAAPGNVAESVILERESESTVMFVEGAKNAYCG  330

Query  330  HLTLKFSPEPASATPHHKHYCLEVGENCSPTIDHCIIRSTSVVGAAVCVSGVGANPVIRH  389
            HLTLKFSP+  S  PHHKHYCLEVGENCSPTIDHCIIRSTSVVGAAVCVSG GANPVIRH
Sbjct  331  HLTLKFSPDVTSTVPHHKHYCLEVGENCSPTIDHCIIRSTSVVGAAVCVSGAGANPVIRH  390

Query  390  CDISDCENVGLYVTDYAQGTYEDNEISRNALAGIWVKNYANPIMRRNHIHHGRDVGIFTF  449
            CDISDCENVGLYVTD+AQGTYEDNEISRNALAGIWVKN ANPIMRRNHIHHGRDVGIFTF
Sbjct  391  CDISDCENVGLYVTDFAQGTYEDNEISRNALAGIWVKNNANPIMRRNHIHHGRDVGIFTF  450

Query  450  DNGLGYFEANDIHNNRIAGFEVKAGANPTVVHCEIHHGQTGGIYVHENGLGQFIDNKIHS  509
            D+G+GYFEANDIHNNRIAGFEVKAGANPTVV CEIHHGQTGGIYVHENGLGQFIDNKIHS
Sbjct  451  DSGMGYFEANDIHNNRIAGFEVKAGANPTVVQCEIHHGQTGGIYVHENGLGQFIDNKIHS  510

Query  510  NNFAGVWITSNSNPTIRRNEIYNGHQGGVYIFGEGRGLIEHNNIYGNALAGIQIRTNSDP  569
            NNFAGVWITSNSNPTIRRNEIYNGHQGGVYIFGEGRGLIEHNNIYGNALAGIQIRTNSDP
Sbjct  511  NNFAGVWITSNSNPTIRRNEIYNGHQGGVYIFGEGRGLIEHNNIYGNALAGIQIRTNSDP  570

Query  570  IVRHNKIHHGQHGGIYVHEKGQGLIEENEVYANTLAGVWITTGSTPVLRRNRIHSGKQVG  629
            IVRHNKIHHGQHGGIYVHEKGQGLIEENEVYANTLAGVWITTGS+PVLRRNRIHSGKQVG
Sbjct  571  IVRHNKIHHGQHGGIYVHEKGQGLIEENEVYANTLAGVWITTGSSPVLRRNRIHSGKQVG  630

Query  630  VYFYDNGHGKLEDNDIFNHLYSGVQIRTGSNPVIRGNKIWGGQNGGVLVYNGGLGLLEQN  689
            VYFYDNGHGKLEDNDIFNHLYSGVQIRTGSNPVIRGNKIWGGQNGGVLVYNGGLGLLEQN
Sbjct  631  VYFYDNGHGKLEDNDIFNHLYSGVQIRTGSNPVIRGNKIWGGQNGGVLVYNGGLGLLEQN  690

Query  690  EIFDNAMAGVWIKTDSNPTLKRNKIFDGRDGGICIFNGGKGILEENDIFRNAQAGVLIST  749
            EIFDNAMAGVWIKTDSNPTLKRNKIFDGRDGGICIFNGGKGILEENDIFRNAQAGVLIST
Sbjct  691  EIFDNAMAGVWIKTDSNPTLKRNKIFDGRDGGICIFNGGKGILEENDIFRNAQAGVLIST  750

Query  750  QSHPILRRNRIFDGLAAGVEITNNATATLEFNQIFNNRFGGLCLASGVQPIVRGNKIFSN  809
            QSHPILRRNRIFDGLAAGVEITNNATATLE NQIFNNRFGGLCLASGVQPIVRGNKIF+N
Sbjct  751  QSHPILRRNRIFDGLAAGVEITNNATATLESNQIFNNRFGGLCLASGVQPIVRGNKIFNN  810

Query  810  QDAVEKAVANGQCLYKISSYTSFPMHDFYRCQTCNTTDRNAICVNCIKTCHAGHDVEFIR  869
            QDAVEKAVANGQCLYKISSYTSFPMHDFYRCQTCNTTDRNAICVNCIKTCHAGHDVEFIR
Sbjct  811  QDAVEKAVANGQCLYKISSYTSFPMHDFYRCQTCNTTDRNAICVNCIKTCHAGHDVEFIR  870

Query  870  HDRFFCDCGAGTLTNQCQLQGEPTQDTDTLYDSAAPMESHTLMVN  914
            HDRFFCDCGAGTLTNQCQLQGEPTQDTDTLYDSAAPMESHTLMVN
Sbjct  871  HDRFFCDCGAGTLTNQCQLQGEPTQDTDTLYDSAAPMESHTLMVN  915
```

<u>Graphical representation</u>

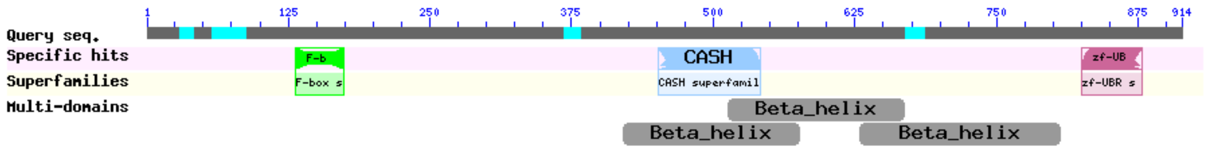

Scavenger receptor SR-C-like protein

>Cb.comp41729_c0_seq2 len=1340

cDNA

```
ATGAAAATAATAAAAATAAAAAAAAACTTGCAGCCGCACGCTGCAACAACTTGCCTGGTTTCCTTTCGACATAAATGGCGACTTACCGACCAAA
CACGCGTCAAAGTAGTCGGGACAGCAACGGTTCTGGTCGAAACAGTTGTCGTCGCAATCACAAGATATCCTGGTCCTGTTTTCTTCGACGCCCT
TTGCGACGAGTCCGCACCTGTTCGCGCAAGACTCGACCGTTTTCGGCGTTTCGTTTTCTGTCGCCGGTTCTTCGGTGGTGGTGTAAGCGTAATC
GTCCGGATCGCAATTTTCGATGATCCTGACGTCGTCGATCGCGATGTCGCTGACGTATCCCGGACCCCTCACCCCCTCCATGACAATCTGAAAG
TCGTCGTCTATTGTGCCGAGATTGTGGAACGACCGGTACCACACGTCGCCCTGGTTGCCGCTTCTGCTAAAAATTGCGTTTTGCGGGGAGAGCG
GCCACGGATCTTTCACCTTTTTCACGTAGGCCCGCAACGTTCCGGTCGTTTTGCCGAACATGTGGTACCAGAACTCCAGGCACGTGTTGTTCGT
CGCCATCTTGTCGTATACGGGCGAAATCAGACGCGCCGTGTCGTTTTCCCTCCGCGACGACGACTCGATGTACATGTAGTGACCGTTTTTGCCT
TTGGCCCCCTTCGTGTGATCGAAACTCGGCCCGGTGCCGATCGATCCGCTCGGCGTCTGATAACTTTCCCGTTTCCAGTCGAAGTGATGGTTTA
GATCGTGCGTCCAACTGCAGATGTCGTTGTCTTCGAAATCGCAAAACAGTTTCGGCCTCGAATCCGTCGGGAAACACGTGGGCGGTACGTTATC
CCACTTGAGGCCGTCGCAGTAGGCGACGGAGGTGCCTCGCAACTCGTAGCCTGCTTTGCAATAAAAATTTAAAACGCCACCTCCATGGGACGGA
AATATCAGCCCGTTTTCCGGCGGTTTCGCTTTGTTTTGACAGGTCGGACGAACGCATTTCGGCGGCGGCAAATCCCAACGTCCGTGGGAACAAA
TGCTGTATTTCTCGCCGGCCAAGAGGTACCCGGCGTTGCATATGAATTTGAGGAATCTGCCTCTCTGTCTCGACCTGATTCTGCCGTTCTTCAA
GCTCACAGAAGGGCAACCGTCTGCCGATCTTTCAAACACAAGGCCAACTATGCACAAACAAACCAACAAAAACAAGCGCATTTCTGCGAGACCG
TTTTTTTATGCGAAGTCGCGCTCAAAAGGCGAACATAATCCGCTAGGCACTAAACCAAAAAATCTACAGCTCGTGCTTAAACTTGAATAGTTCT
CCCCGTTCTTCAAGCTCACAGAAG
```

Protein RF -3: -1209->-1 (402AA)

```
MRLFLLVCLCIVGLVFERSADGCPSVSLKNGRIRSRQRGRFLKFICNAGYLLAGEKYSICSHGRWDLPPPKCVRPTCQNKAKPPENGLIFPSHG
GGVLNFYCKAGYELRGTSVAYCDGLKWDNVPPTCFPTDSRPKLFCDFEDNDICSWTHDLNHHFDWKRESYQTPSGSIGTGPSFDHTKGAKGKNG
HYMYIESSSRRENDTARLISPVYDKMATNNTCLEFWYHMFGKTTGTLRAYVKKVKDPWPLSPQNAIFSRSGNQGDVWYRSFHNLGTIDDDFQIV
MEGVRGPGYVSDIAIDDVRIIENCDPDDYAYTTTEEPATENETPKTVESCANRCGLVAKGVEENRTRISCDCDDNCFDQNRCCPDYFDACLVGK
SPFMSKGNQASCCSVRLQVFFYFYYFH
```

Comparison with *Tribolium* PREDICTED: similar to scavenger receptor SR-C-like protein (AA)

```
Query  23   CPSVSLKNGRIRSRQRGRFLKFICNAGYLLAGEKYSICSHGRWDLPPPKCVRPTCQNKAK  82
            CP + + NGR+R RQRG+  + +CN GY LAG++Y++C  G WD   PKCVR TC+  AK
Sbjct  59   CPPIKVPNGRVRYRQRGKIARVLCNTGYTLAGDRYTVCVQGVWDNTYPKCVRATCR-AAK  117

Query  83   PPENGLIFPSHGGGVLNFYCKAGYELRGTSVAYCDGLKWDNVPPTCFPTDSRPKLFCDFE  142
            PP NGLI+PSHGG VLNF+CK+ ++LRG+S+AYCDG KWDN  P C PT+S P L CDFE
Sbjct  118  PPANGLIYPSHGGAVLNFFCKSHFQLRGSSIAYCDGFKWDNPLPACLPTNSSPALSCDFE  177

Query  143  DNDICSWTHDLNHHFDWKRESYQTPSGSIGTGPSFDHTKGAKGKNGHYMYIESSSRREND  202
              D+C W HDLNH FDW R +Y TPSGSIGTGPS DHTKGA GK+G YMYIESS+R  ND
Sbjct  178  SGDLCGWNHDLNHDFDWMRLNYATPSGSIGTGPSHDHTKGA-GKDGFYMYIESSARNIND  236

Query  203  TARLISPVYDKMATNNTCLEFWYHMFGKTTGTLRAYVKKVKDPWPLSPQNAIFSRSGNQG  262
            TARLISPV+DK     N C EF+YHM+G TTG+LR YVKKV + W L P+ +++ ++GNQG
Sbjct  237  TARLISPVFDK-TDENVCFEFYYHMYGVTTGSLRIYVKKVNETWQLDPKKSLWEKTGNQG  295

Query  263  DVWYRSFHNLGTIDDDFQIVMEGVRGPGYVSDIAIDDVRIIENCDPDDY--AYTTTEEPA  320
            + W+R F  +G I DD+QIV+EGVRG  YVSDIAIDDVR+I NC PDD      TTT EP+
Sbjct  296  NRWFRGFVTIGAISDDYQIVIEGVRGSSYVSDIAIDDVRVIVNCSPDDAIETETTTAEPS  355

Query  321  TENETPKTVESCANRCGLVAKGVEENRTRISCDCDDNCFDQNRCCPDYFDACL  373
            T   TP +VESC NRC      +  +   I+CDCD+ CF+++RCCPDYFD CL
Sbjct  356  T--WTPISVESCENRCDTNDTHLAHDWL-ITCDCDEACFERSRCCPDYFDFCL  405
```

Graphical representation

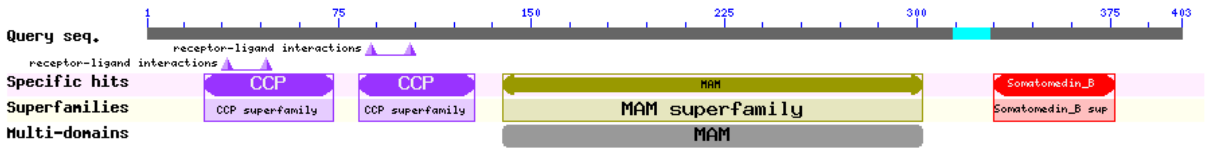

Eater

>Cb.comp30666_c0_seq1 len=4667

cDNA

AAAACACACATGACCCTACGAATGGTGATTATCCGGTCGTCGTGGGCCTTTGTCGTATCAGTGACCCAGAGTCTTTTGTGTTATGGAGTAGTTT
CGATAGAGCGCGAGGAAGCTGTGCGTATTAATTGGTGGGAGTGACTTAATATCGGCGAAACTTTTAAGGAGAATTGGATTGAAATATTCTTAAG
AGTTGAAGATGCAGGCCTTGTTCTTGTTTACAATTTGTTGTTGCGTTTGTTCATCGATTGAAAAACTCGCCGGAGATCACATCTGCCAGATGCA
AATCAGTGAACCCGTAGAGACATTAGTGTGGGAGATCAAAAACAGAACAGTGCGCACTTTCCAATGGTGCCTGAATGTTCCACCGAGATGCTCA
AGCTATTCGAACATTCGAGTCAACGAAACTCAGATATTGGTCAATTACATCAATAAAACAGTAGATCTGTGCTGCGATGGATACTACCAACATG
AAGGCAAATGTGTCAATGAAGCCTGCAAAGGATGCCAAAACGGTTATTGCAGCAATGGGTTTTGTGACTGTAAGGCGGGTTGGATGGGAACTTC
CTGCGATACAACTTGCATGACTGATCAACGCGGTTCCAACTGTAAAAATGAGTGCAACTGTTGGAAATATATACAATGCGTTGTCGAAAACAAA
GCAGAGTGTGATTGCGTCTCAGGTTGGCTGCCGGACAGTTGCGAACTGACCTGTCCAAAACCCTACTACGGAGCAAAATGCGTTCACCTATGTA
CTTGCAACATACTGACACAGACCTGCACCATTGAAAAAGGAAAATGCGTCATTAAGGAGAAAAAATTTAAAGACGTAATTGATTACGAGAATGA
AAGAGACGAGTCCACATCTGTCACCCTTAATACTGCTGACCTCAAACCATCGTCAACGCCTCGATCGCCATTTGTAACGAAAATAACATTACTG
GTTACTAACCAGTCGGGCTCTCCAGATAACCTTTCAGAGAACGAAAATTTTCCCGTGAGAGATCAACAACAAAAGGAGATAGCGAGTAGAAATT
TATTTTTGAGTATTGCGTCGGTGTCAGCCATCACTGTGATTGTCACTGTAACTGCGATTGGGTTGTTGGCGTTGAAATTACTAAGGAAAAAAGC
AGACAAACGTTGCTCGGATATAAATTCTCCGGCTGTGGCTGTTTATACTACCAGTATATTCCACACCCCTCTCCCAGAGCCACCCCTCTTTGAA
AACCCCACTTATTACTCAGCTATGGAAAACAATATTACCGAGCTGCGGGAAATAAGATTGAGAGATTTGGACAAACTAGACCGAAATAAGCAAA
TTGCCAAAAAAGCCAATCTCGAAAATCTATCCTGGCATCCAGACATACCTCGACCTACGTCAGCTACAGGAATACTGGCTAGGGATGCATCGGA
GAATGTTTCTCCTCAACCATTCTCGGAAGAGTTGGAACCTTTATACGACGAGATACCCTCGAGAGAGACCCCTAAATTGTGTTCTGTCCATTCT
CAACATTCGATTCCAGATCAGTTATCGACCTATATGAATGCTGCCGTTATATTGAAAAATAATAGATAGCAGCATTGATCACCGATCTTCGTTC
GGGAATAATTTAATTTTGAGTGCAATCAATGTCTCGATTCTAAAGTTTATGGTAATACAAACAAATAAACAAATTGTTTAGACAAGCGCCATCT
ATAAATAAATAGCGAAACGAAAACGGGTGGCCGTGCAACGTACTCTCTGTCGTAATAATTAAAAACAGATCGTCTGATCATGCGTTTTAATTAA
CAAATGGAGGCTAAAATCGTTTCATCATAAAAATTAAATGTATTAATTAATATAGTAAACGATTGTTCGAGTATAAGTTTCATTTATTTTATCT
GTCTTCTAATCTTTAATGGCCCATAAGAACTTCTCGGGGGATCAGTTCGAAATGCATCAATGACCTCCGCAACCTCCTTACGCAGACATTGCTA
CCCTTGTGCTCTTTTACAAATCCGGCGTTACAGATGCAGAAATTAGGCGCAGAACAAACACCATTCAAACAGCCTTCTGGACAAAAAGCTACGC
ATTTGTGCCCATTAGTTCCTGGAGATTCAATGTATCCCTTCTTGCAAGTGCACGTATCTGGAGCAGTACAGTCTCCATTCAAGCATGGCGAGCT
ACAATGCGGTACGCAGCTGGCTCCGTTTTTACTATCTAGACTCCAGCCGGGTTTACAAGTACAAGTGTTTGGAGCGGAACAAATACCATTTAAA
CAACCTTGAGGGCAGTCCGGAGCACAAGAGTTCCCTAAAAGCTTGTAACCGGGCTTACAAGAACACTTGTTGGGTCCTATGCAACTGCCTCCAC
CACAGCCTCCCTCGCAATGGTACTCACATTTGCGGCTGTCCTTATTTACTGCAAAACCGGATGAGCAACTGCAAGTCTCGGGTCCTATGCATTT
ACCTCCAATACCACATCCTCCCGTGCACGCCGGTACGCAATACTTTCCGTCAGCACTGTGAGTGTATCCAGCGTTGCAGTCGCAAAAACCTCTG
ACGGAGCATACCCCGTTTAGGCATCCGATGGGACAGGTGGGAATACATTTGTTTGGATCTTCGCCCTTAATATGACCTCTCTTGCAGGTGCACA
CATTCGGAGCTGTACAGTTTCCATTCTCACAGCCGTTCTCGCAGACTGGACGGCACTCATAATAATTGTGGGGTATTCTTTCGTATCCGGAACA
ACAAATTTCAATCCTGCTTAGACCTTCCCTGGACCCGTTGCCTCTGGGAACTACACCGTGTCTATCCTGCGGATCTATTATGTCGATAGTGGGC
ACTTCAAGGGTGCAAATTCCGGTCCTATGAGAGGTGGAATTGGTGTGAAAAGTGTTGTTTGCCAAACGTTGACCTAAGGCCGGTGCCACGTAAA
TAGAAAGTAATGCGGCAAAAGCGTAGTAACCGAACCGGTGCATTTTTACGAATTATACGCTACCACTCACCAAACTGAACTAATCTGTTATTAA
CTATTATTACAAAATGCAGAAAGTGTACCAGCGGATGCTGCTGAAACGATAAGAACCCCAAACTTAAATAACCATATTTATCGAGAATTGTTGT
TCGGCGTGTGGGTCATTCTTGTACAGGGTCACCCGATTTGTTTGAACCTCGGTTATTTTCTTCGTGGTCAGAATAAATTGTCGTATACAGAGTG
TTCTAATAGGCCCCTATTAATACGCGTTTAAGCGGCTGTTGTTGAAGCGGTACAATGTTAGCGAATGAAGAACCAGATCTAGAGGACAGTCGAA
GCCTCTGGAGATGAAATCCTGTCTGCTCAGGGTGATGTTGGTTTTGTATTTGCCTTTGCCATATACAGGACAGCGTCTAGTGCCACCCAGATCA
TACCAAGATGATGAGGACGACCATGACGACCAAAATTTGCTGGGCGAGGACAGGGACTTATATCAGCACAAAAACTGGACAGACGAAAAAGGCT
TGTTCGTCGGCGCTCCATGTGAGCATGTTTGCAGCTCGAGGCTGCAACACGCTTACTGCAATTTGGTTACCAACATATGCGAATGCGAGAAGAA
GTATCCGGTTAGACTTAGTAACCCCTACTCAGGATGTAGCAAACCTAAGAGTTTGGGCGAACAGTGCTACTACCACGAAACTTGCGAGTACACA
GACCAGCACGCGTCCTGCATTCAGGTCCATCACAACGCCATATGTCAATGCCAGACTGGCTACCATGCCGTGTCTTTCCAGAAACCTTCGAAAC
GGACATTTTGCGCCGAAGACGCCGTCGTCGTAGCGACAGACTTTTCCACTTTCGCGGGGGTCTTGTCGGGTATAGCTATACTCTCTGGCCTCAT
TTGTTTCGTATTGCATCTTTTTAATCAGAATTTGTACGGGTCTGGACACCGCAGGCATAGGTTTGGCAATGCCAATCTTGCTCCGCCTATATTG
TTTTCTAGCGATCCAGGTTCAAGGAGGCCCAGTATAGCGTCGGTGCATAGTTCCAATTCTATAAGGAGCTACAGTGCGCGGAGATACGAGCGAG
AGAGGGAACAAAAGGAAGAGAGGGAGATGCAGAGGCGTCTGTCGAAAATGGCGGCCGGCTCTATGAGTATAGGTCCTCCGACTCCTAGTCCTCA
TTCTACAGATGACCTCCTGCCAACTTTGAAGGAAGATGTTCAAGCACCCCCGTTGGCCACTAAAGAAAATTCGACGACCAGTTTTCAAGAGGAA
AATCTGCCCTCGACGTCGAAGCAGTATTCGTAGAGACATTTGATATGAGTTCTTTAATATTTAAACGGAGCGAAACTTTTCTCTCGAATCGCCA
TATCGCTGAGAAACGTAGCTGAGATTGCATTTTTTTGTAAAACAGTCAAAAACTGACTGTGTTGGGATGTCGTGTCATACCATGATCAAACATA
TATCCTTTTTAGAGTCGATTATTTGGTCGCATTTAGGCAGTTTTTACACTATATAAAAACGCGGGTGTATTTAATTTTCGGTAGTTGTTAAGTT
TGTGTGGCCGAGCTAAAATTAAAAAAAAACTTTCTGAGAATACTTAAGAAACAATTGGTACGATAAATTTTGGTGTTTTATTATTTTGTGAACT
GATTTTTCGAAAACGCCTGGTGATGATCTTCATTTACGAATTATTTATATGTTACAGGAAG

Protein RF -1: -2957->-1893 (354AA)

MHRFGYYAFAALLSIYVAPALGQRLANNTFHTNSTSHRTGICTLEVPTIDIIDPQDRHGVVPRGNGSREGLSRIEICCSGYERIPHNYYECRPV
CENGCENGNCTAPNVCTCKRGHIKGEDPNKCIPTCPIGCLNGVCSVRGFCDCNAGYTHSADGKYCVPACTGGCGIGGKCIGPETCSCSSGFAVN
KDSRKCEYHCEGGCGGGSCIGPNKCSCKPGYKLLGNSCAPDCPQGCLNGICSAPNTCTCKPGWSLDSKNGASCVPHCSSPCLNGDCTAPDTCTC
KKGYIESPGTNGHKCVAFCPEGCLNGVCSAPNFCICNAGFVKEHKGSNVCVRRLRRSLMHFELIPREVLMGH

Comparison with *Tribolium* nimrod B (AA)

```
Query  38   RTGICTLEVPTIDIIDPQDR-HGVVPRGNGSREGLSRIEICCSGYERIPHNYYECRPVCE  96
            R GIC LEVPTID+I P+DR  G+ P+GNG+R G S+IEICCSG+ R PH+++EC PVCE
Sbjct  39   RAGICALEVPTIDLISPEDRASGIRPQGNGTRPGFSKIEICCSGWARKPHSHFECEPVCE  98

Query  97   NGCENGNCTAPNVCTCKRGHIKGEDPNKCIPTCPIGCLNGVCSVRGFCDCNAGYTHSADG  156
            NGC NGNCTAPNVC+CKRG+IK    N CIPTCPIGCL+GVC+  G C CNAG+  S DG
Sbjct  99   NGCPNGNCTAPNVCSCKRGYIKDTLQN-CIPTCPIGCLHGVCTNSGLCSCNAGFQLSPDG  157

Query  157  KYCVPACTGGCGIGGKCIGPETCSCSSGFAVNKDSRKCEYHCEGGCGGGSCIGPNKCSCK  216
            K+C P CTGGCG+GG+CIG E C C  GF +N  + KCEY CEGGCGGG+CIGPN+CSCK
Sbjct  158  KFCTPVCTGGCGMGGECIGAEACRCKPGFTLNPQTNKCEYFCEGGCGGGTCIGPNQCSCK  217

Query  217  PGYKLLGNSCAPDCPQGCLNGICSAPNTCTCKPGWSLDSKNGASCVPHCSSPCLNGDCTA  276
            PG+K +G +C   CPQGC NG+C+APN C+C+PGWSLD K G+ CVPHC  PCLN +C+A
Sbjct  218  PGFKQVGAACVAQCPQGCKNGLCTAPNVCSCEPGWSLD-KTGSVCVPHCREPCLNAECSA  276

Query  277  PDTCTCKKGYIESPGT-NGHKCVAFCPEGCLNGVCSAPNFCICNAGFVKEHKGSNVCVRR  335
            PDTCTCKKGY   P    G++CVAFCP GC NG CSAPNFCICN GFVKE KGSN CVRR
Sbjct  277  PDTCTCKKGYTVDPSNPKGNRCVAFCPGGCENGTCSAPNFCICNPGFVKEAKGSNRCVRR  336

Query  336  LRR-SLMHFELIPRE  349
            LRR ++MHFELIP++
Sbjct  337  LRRAAMMHFELIPQQ  351
```

Graphical representation

NO PUTATIVE CONSERVED DOMAINS HAVE BEEN DETECTED

Systemic RNAi defective protein 1 (Tribolium)

>Cb.comp42797_c0_seq1 len=4083

NOT PRESENT

>Cb.comp37306_c0_seq1 len=1386

cDNA

```
GAAAAATGAATTGTTCCCTGGTTTCGGTAAGATCTTGAAAATACGAATAGGGCGTTTCGTTGAGGGAGCGCTTTGAGCTCCGCGACTTTTGAAT
TCCAAGCTGCTGCCCTCAAGATGAAAAGAAGAAGACTTTCGGGCATGTGGTCCATCGTGGTATGACGTAATGAAAGTTTAAGTTATGAGTTCGT
GCGCAGCGGGTTCGTGAAGGGTAAAGAAGAAGAAATTCTGAGTTGCGCGACTAACCCGTTTTGAGTCTGTGGGTTGGGTGTTATTGTTTGTGGG
GCTAAGTTATTTTGGGATTTTTCGGACTTTTGTTCGTTGCGCGTGTCGAGGGTCGGTCGAAAATTCTTTATGTTCGATTATGTGGGCGCAGTAT
AGAAGGCATCGTTAGAATTGTTCTTCTCGCGGCAGGCGTATCAAAATGTTTAGGCCTTTGGTGGCGATACTTTGTTTGTGTTCGGCGTATTGTC
GGATCTTGAATACGATCGTGGAGGATTTGCCGTACTCGTCGCCGTTCGTGGCCACCGTCAACAATACCGTCGAGTACATACTCGTGTTTTCGGG
CGGAAGTGACGTTCTGCCGCCTCGCGTGACGGTGTCGTCGAATAGCGCGACCGTAGCCGCGCCGTTGATGATTGTCGCGGCACAACCGAAAAGT
TTACTGTCTTGGGACTTGCCGCTTCTGGTGGAAAGCGCTCAAGGCGTCCGAAGTTACATGGAATCTTCACGGACCCTATGCCTGGACATGTTTA
AGGGCTTACGCTCCGACAACAGCAACCTGATTGTGACGATATCGTCGGCTTCAAACAGTAACGTGGATTTCAAGTTGGTGGTCAATTCGAAAGA
GGAGTTCCGGTTGCACCACTCGGTCGAGTATAACATTACGATATCGCCCAGCGAGCCACAGTATTTCTTTTATAACTTCACTTCGAATTTGACG
GACGTCGTGTCCAACTTCGACACCGTCATTTTGGAGGTTACGTCGCAAGATTCCGTCTGCACGATCGTCAGTATTCAAAACGTTAGCTGTCCCG
TCCTTGATTTGAACCAGGACATCACTTTCCGCGGCTTTTACGAAACTTTCGACGCGAAAGGCGGCATCACTATACCGAAGGACAAGTTCCCGTT
CGGTTTTTTCGTGGTGTTCGTCGCGAAAGCCGACGACTCGCAATGCACGGGAACGCCCACTCCGACCAGTAACGATCGTACCAAGACGATCACG
CTCGTCATAAAGCCGAGCATCAGTTACAACGATTACGTGGTCGCGGTCATATACACGCTCTGCTCGATCGGCGCGTTCTACTTCGTGTTCGGCG
TGTTGGCGTTCGCGCCGTGCACGCGCCACTGCTGGTTCCCGCTTCGGCTCGACGACGACGACGACGCCGA
```

Protein RF 2: 422->1385 (321AA)

```
MFRPLVAILCLCSAYCRILNTIVEDLPYSSPFVATVNNTVEYILVFSGGSDVLPPRVTVSSNSATVAAPLMIVAAQPKSLLSWDLPLLVESAQG
VRSYMESSRTLCLDMFKGLRSDNSNLIVTISSASNSNVDFKLVVNSKEEFRLHHSVEYNITISPSEPQYFFYNFTSNLTDVVSNFDTVILEVTS
QDSVCTIVSIQNVSCPVLDLNQDITFRGFYETFDAKGGITIPKDKFPFGFFVVFVAKADDSQCTGTPTPTSNDRTKTITLVIKPSISYNDYVVA
VIYTLCSIGAFYFVFGVLAFAPCTRHCWFPLRLDDDDDA
```

Comparison with *Tribolium* systemic RNA interference defective protein 1 (757AA)

```
Query  31   PFVATVNNTVEYILVFSGGSDVLPPRVTVSSNSATVAAPLMIVAAQPKSLLSWDLPLLVE  90
            PF+   N T E++LVF      + P RV   S+ A +A+P+++V  Q + ++SW +P +V+
Sbjct  36   PFL--FNQTTEHVLVFPTSDSIYPYRVKAWSSGAKLASPVLVVVRQEREVISWQVPFVVD  93

Query  91   S--AQGVRSYMESSRTLCL-DMFKGLRSD----------NSNLIVTISSASNSNVDFKLV  137
            +   +GV  +   +SRTLC  DM +  ++            + N I+ +S++S  NVD  ++
Sbjct  94   TTMKEGVVHFHNTSRTLCHNDMPRIAKAKATSRILPIQLSQNFIIALSTSSLVNVDISVM  153

Query  138  VNSKEEFRLHHSVEYNITISPSEPQYFFYNFTSNLTDVVSNFDTVILEVTSQDSVCTIVS  197
            V  + +F L      Y +++SPSE +Y++Y F            + ++E+ S D VC  VS
Sbjct  154  VEEERDFYLQEGRPYEVSVSPSESKYYYYKFHDKKNT------SAMIEINSDDDVCLTVS  207

Query  198  IQNVSCPVLDLNQDITFRGFYETFDAKGGITIPKDKFPFGFFVVFVAKADDSQCTGTPTP  257
            IQ+  CPVLDL++DIT+ G Y+T + KGG+TI + +FP GFF+VFVAKAD+ QC+    +
Sbjct  208  IQDSFCPVLDLDKDITYEGKYQTINRKGGMTIRQREFPDGFFLVFVAKADNYQCSQKHSV  267

Query  258  ----------TSNDRTKTITLVIKPSISYNDYVVAVIYTLCSIGAFYFVFGVLAFA  303
                         +RT TIT  I   I+  +Y +A + TL ++ +F  V  ++ FA
Sbjct  268  LLVEHRKQHLILANRTSTITFTINKGINGKEYEIASLATLGALLSFCIVSTIMIFA  323
```

Graphical representation

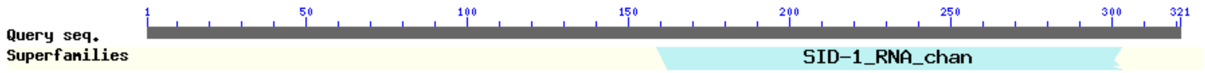

Sid-1-related B precursor (Tribolium)

NOT PRESENT

Sid-1-related C precursor (Tribolium)

>Cb.comp42797_c0_seq1 len=4083

cDNA

```
TGACGTCATCCCGTTGCCAGACCGTTGATAAACTTAACAATTGAATAAATTACGAAAACTACAATAAATTCCTTTATTTAGCACAATCATTATG
GTACATTTAGTATATTGATCATTATGTACAATAAAATCATCAAAGTCGGTAAGTTGTGCACCACGTGCGTTTGTAACGTATGTGCAAGAACAAA
AATTGTGCGGCAATGAATATTCCTTGGAGCATTATCTTTAAATGGAATGTTTATTTGTATCACAACGAAAAGAAAAAAACAATATTCGAAACGA
TTCATTAATATTACGCTAATGTCATTCTGATTCTGTTATTATATGTTCTCTTTTTAACAAATCGTTTTATTACCAACGGTGGAAACAAATATTC
CTAAAAGAGTACCAACCGAAGTTAAAAAATTTACCTACATTTATCTCTACGACACTATTTTAATAATAATAAAGCCATTTTAAGTAGTAAAACG
ACGATAAAAAATCGAAATATATAAGAAATGCGTGTGCGACCCTCTACCTAACAATTTAAGCAAACTCTCGAGGTAATCCTAGTGTTTAAAAACT
ACACCAACAATTCGTTAATAATATTATCTAATGACCGGTAACGATAAATATAAAGCAATTGCGTTAATACGGTGGGAATTTCGACTTGGTAAAA
TTTTCGCTTTATGGGCGTGTCTAATTTCCTAACTGCGCGAGCGTCTATACGCGGCAGAGCCAAAACAGGCAGATTTCATTTAGGACTTTTTTTC
GCCGAATTTGCCCAACGAACGAGTTACATTAAATTTTGCTTAGTACCGCCCCTCCGCCACCGCCGTCTACGATTCCACGGGAACAATAAAAACA
AATATCACACAGCTAACCAATGAGAACATCGATTTTTTTTTTTACCGCGCACCCTAATAAACCGTTTAAGTAATTAACATTTTGCATTTCATTGA
CATTATTATCACCCCCCTCCGTGCTCGACACGCGACCCTACCTAACCTAACATCCTGCTGAAAAAATAGGGGTAAAACGACTTCCGTTGCTTGT
TGTTGCGTTGAAGCTGACGTTTACGGTTAGGTTTTTTCTCCTCCTCCGATGTGAAGACGTTGTAAACGGTCGAGTTGTCGTCGTCGGGGTTTAG
GGTGGAAACGTCAGGCAGTAACGACTTGGGGAGGAAATGCCTTTTCTCCTCGTCCAATAATTCATCGGCACCTACGTACGGTTCGCTGTCGTTG
TTCTCCGGCTGGTTTCGGTTGAGCTCGTCGATCTTCGGGTCGATGATCGTCGGTTCCCGTGTACCCGACTCGATGTTCTCCAAAATGGACGCGA
TGTTCGGTTCGAAACTCTCCCCGTCCTCGTTCTTCAACGCCGACAATATAATCGTCTCCGTGATGTTCGTGTTCTTGTTCTCCTCCCGGAAACT
```

```
AATCTCCAGGTTATCCTCCAGCTCGCCGTTGATGTTGTTGTTGACCGAAAAATTGATAATCTTCGGCGTCCGAAAGTTTTCTCCTTCCAGCAGG
GGCACCTCCTTCCTGTACGTACGGTTCAAGGTCGTCGACGAGTCCCTGATGTCTGGCAGCAGTTCGTACTCCTTGTTCAAGTCCGTTTCTGCCG
TTTTGCGCGTCTTCGTTTTGGGCGAAGTCGGTTTCTTCGATGGTTCCCTGATCACCGGTTTCTTCGTCGAGGGCGGAGCTTTGGTTTCGTACTT
CTTCGCGGGGTAAGTTTCCCACGAGGGAGTGTTAATCCTGGGGGTAGATTCCGTCGGGGGTTCTTCGGTCGATTTCGGCTCAGCTCGTCCGTCT
TCGGCCGTGTCGTTGAGCTCTATGATGTCGAACATGGTGCCGTTTTCGATTATCCCGAACAACTGGCCTTGGTTGATGGCGCTAGGGTATTCTT
CCGTGTTAGTGTTCGGGGTCGTGGTTTGTTCCGGGCGGGGGTGGTTCTTGGAGGGATACTTCAGGTCTCGACTTGCCTCTCCGTTCGCGGCATC
CTCGGGAGCCACGGTGCCTGCGACCGTCGTCGCCTTCTCCGTCGAGGTCGTCGCCTCCTCACCCTCCACGTCGTGCCCGTTATCTACGACGGAG
GTGACTGTCAATTTCGGGCTCCGGGTGGCCGTGTTGATGGATTTATCTGAGGAAATCCCCTTCTGGATGGCGGATACGAAAGTTGCGTTGTCCG
GGCTCCAGGGGTCCGAGTGATTGGCGGCGGCGGTTGGGGTTGCCGCCGCCATGCGTAGCTCTGTGGGGGTTTGTGCGGTCGTGAGAACGGCTTC
CTGGTGCCTGGACAGTTTCCACGGTAGATCGGTAGTGGTGGACGCCTGATTAAAGGAGCTCTTCGTCGAGACGGGACTCAAAGTGTCCTCCGTC
TCTCTTCTGTGCCTCGTAGGCATTCCGTAATCAACGTCGACAGTGTTGGACGTCCAAATCCAACTTTTCGTCGTTTTACCGTTTAATGAATTCT
CGTCGACGACGGTAGTGGTTTCGTAATCAGCGGTGACGCTCGACGACGACGACGACGCCGACGCCGAAGCGGTACCGCCCACCGACTCGGACCC
GGTCCGAGCCGTTAACGTGTCGAGCGGCGAGGACAGCGAGTCGATCGACGAGGTCGACTCGCAACGTAATTTTCCGTACGACCGCGAGACGAGA
CGTTGCAGGACGCAGCCCTACCTCGACGACTTGGCGACTCGCCACCCCCACGTGCTAGCGAAAAAAAGTTACATCTACCTCTACAACGTGACGA
CGGTCGCGCTGTTTTACGGGTTGCCCGTCGTCCAGCTCGTAATCACGTACCAGCGGGTGCTGAACGAGACCGGGCAGCAGGATTTGTGCTATTA
CAATTTCCTGTGCAGTCACCCGTTTGGTTTCCTCAGCGATTTCAATCACGTCTTCTCGAACGTCGGTTACGTGCTGCTCGGTTTGTTGTTTCTC
GGGATCACGTATCGGCGCGAGATGTCGCACGGCGACGACCTCGACTTTGACAGGCACTACGGTATCCCGCAACACTACGGGCTGTTTTACGCGA
TGGGCGTGGCCCTGATCATGGAGGGGGTGCTCAGCGGCAGTTATCACGTGTGCCCGAGCCAGACGAACTTCCAATTCGATACGAGTTTCATGTA
CGTGATGGCGGTACTGTGCATGGTCAAGCTGTACCAGAACCGTCACCCCGACATTAATGCCAACGCGTACACCACGTTCGCCGTGCTCGCCCTC
GCCATCTTTTTGAGCATGGTCGGCATCTTCGAGAACACGGAGAGTTTTTGGACATTCTTCGTCGTCTTCTACGTAGCGTCGTGCCTCTACCTCT
CGTTGAAAGTCTACTACATGGGTTGCTGGAGTCTGAGTACGACGTCCCTGACGAGGATCGGCCACACGTTGAGACACGACTTTCGGTCGAACCG
GTTGAACGCGCTCGTGCCGTCTCACAAAGCCCGTTTCTGCGTGCTGCTGTTCGGCAACGCTTGCAATTGGGCGTTGGGGTGCGCCGCGATGTAC
TACCGTCCCCGAAATTTCGCGGTCTTCCTCCTCTTGATCTTCATGTCCAACACGCTGCTCTACTTTTTCTTTTACATCGTGATGAAGTACGTCA
ACGGGGAACGCGCGAGACTCGTCTGCTGGACTTACTTGGCCGCGTCCAGCGTCTGCGCGTGCGCCGCCTTGTATTTTTTCCTGCACAAATCGAT
ATCGTGGTCGGACACGCCCGCGCAGTCGCGACGCATCAACACGGAATGCACGCTGCTGAGGTTCTACGATTACCACGACGTGTGGCACTTTTTG
AGCGCGGCCGGCATGTTCTTTACGTTCATGGTGTTGCTCACCCTCGACGACGACATCGCGCATACCCACCACTCCCGGATAACGGTGTTTTAAG
CTCGGGACTGGGTTCGGTTTTTCGTTTTCACTATTTTTCGAACGGATAGTAAAGATTAGGTGTATTTTTTAAAATAATAAAATTTAGGACTTTT
ATTAAATAACGTGAACTGCGGGTGTTTTGGAAACTTCTAAAATTGCCTGTACCGATTACCCATCCGTGAAAAATTTTTACAAAGGGTAGTCCCA
TTCTGGATATTAGGCGGCCAAAAATATTTTCAGCAAAATTT
```

Protein RF 2: 2471->3853 (460AA)

```
SAVTLDDDDDADAEAVPPTDSDPVRAVNVSSGEDSESIDEVDSQRNFPYDRETRRCRTQPYLDDLATRHPHVLAKKSYIYLYNVTTVALFYGLP
VVQLVITYQRVLNETGQQDLCYYNFLCSHPFGFLSDFNHVFSNVGYVLLGLLFLGITYRREMSHGDDLDFDRHYGIPQHYGLFYAMGVALIMEG
VLSGSYHVCPSQTNFQFDTSFMYVMAVLCMVKLYQNRHPDINANAYTTFAVLALAIFLSMVGIFENTESFWTFFVVFYVASCLYLSLKVYYMGC
WSLSTTSLTRIGHTLRHDFRSNRLNALVPSHKARFCVLLFGNACNWALGCAAMYYRPRNFAVFLLLIFMSNTLLYFFFYIVMKYVNGERARLVC
WTYLAASSVCACAALYFFLHKSISWSDTPAQSRRINTECTLLRFYDYHDVWHFLSAAGMFFTFMVLLTLDDDIAHTHHSRITVF
```

Comparison with *Tribolium* Sid-1-related C precursor (768AA)

```
Query  32   GEDSESID----EVDSQRNFPYDRETRRCRTQPYLDDLATRHPHVLAKKSYIYLYNVTTV  87
            GE+ + I     E D       D+  R  ++  YL DLA + P V   KSY+YLYNV TV
Sbjct  337  GEEVDEISLDETEYDVVSEADQDKSIRLGKSVVYLSDLARKDPRVHKYKSYLYLYNVLTV  396

Query  88   ALFYGLPVVQLVITYQRVLNETGQQDLCYYNFLCSHPFGFLSDFNHVFSNVGYVLLGLLF  147
            ALFYGLPV+QLV+TYQR LNETGQQDLCYYNFLC+HP G +SDFNHVFSN GYVLLGLLF
Sbjct  397  ALFYGLPVIQLVVTYQRALNETGQQDLCYYNFLCAHPLGVISDFNHVFSNSGYVLLGLLF  456

Query  148  LGITYRREMSHGDDLDFDRHYGIPQHYGLFYAMGVALIMEGVLSGSYHVCPSQTNFQFDT  207
            LGITYRRE++H  DL+F+R YGIPQHYG+FYAMGVALIMEGVLSGSYHVCP+  NFQFD+
Sbjct  457  LGITYRREITH-KDLNFERQYGIPQHYGMFYAMGVALIMEGVLSGSYHVCPNTANFQFDS  515

Query  208  SFMYVMAVLCMVKLYQNRHPDINANAYTTFAVLALAIFLSMVGIFENTESFWTFFVVFYV  267
            SFMYVMAVLCMVKLYQNRHPDINA AY TF VLA+AI L M+GI E    FW  F + Y+
Sbjct  516  SFMYVMAVLCMVKLYQNRHPDINATAYATFGVLAVAILLGMIGILEGNLYFWIVFTIIYL  575

Query  268  ASCLYLSLKVYYMGCWSLSTTSLTRIGHTLRHDFRSNRLNALVPSHKARFCVLLFGNACN  327
             SC YLS+++YYMGCW L      R+     ++F S  LN + P HKAR C+L+  N CN
Sbjct  576  LSCFYLSIQIYYMGCWKLDAGLAMRVWRICVYEFWSGPLNVIKPIHKARMCLLIIANLCN  635

Query  328  WALGCAAMYYRPRNFAVFLLLIFMSNTLLYFFFYIVMKYVNGERARLVCWTYLAASSVCA  387
            W +    +Y   ++FA+FLL IFM NTLLYF FYIVMK +N ER   +   +L+ S +CA
Sbjct  636  WGMAFWGVYKHQKDFALFLLAIFMGNTLLYFSFYIVMKIINKERVNKLSLFFLSLSVLCA  695

Query  388  CAALYFFLHKSISWSDTPAQSRRINTECTLLRFYDYHDVWHFLSAAGMFFTFMVLLTLDD  447
             +A+YFFL+KSISWS TPAQSR+ N EC LLRFYD+HD+WHFLSA GMFFTFMVLLTLDD
Sbjct  696  ISAMYFFLNKSISWSRTPAQSRQFNQECKLLRFYDFHDIWHFLSAIGMFFTFMVLLTLDD  755

Query  448  DIAHTHHSRITVF  460
            D++HTH ++I VF
Sbjct  756  DLSHTHRNKIVVF  768
```

Graphical representation

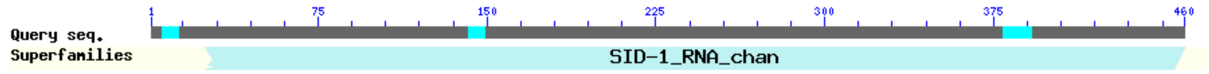

>Cb.comp37306_c0_seq1 len=1386

cDNA

```
GAAAAATGAATTGTTCCCTGGTTTCGGTAAGATCTTGAAAATACGAATAGGGCGTTTCGTTGAGGGAGCGCTTTGAGCTCCGCGACTTTTGAAT
TCCAAGCTGCTGCCCTCAAGATGAAAAGAAGAAGACTTTCGGGCATGTGGTCCATCGTGGTATGACGTAATGAAAGTTTAAGTTATGAGTTCGT
GCGCAGCGGGTTCGTGAAGGGTAAAGAAGAAGAAATTCTGAGTTGCGCGACTAACCCGTTTTGAGTCTGTGGGTTGGGTGTTATTGTTTGTGGG
GCTAAGTTATTTTGGGATTTTTCGGACTTTTGTTCGTTGCGCGTGTCGAGGGTCGGTCGAAAATTCTTTATGTTCGATTATGTGGGCGCAGTAT
AGAAGGCATCGTTAGAATTGTTCTTCTCGCGGCAGGCGTATCAAAATGTTTAGGCCTTTGGTGGCGATACTTTGTTTGTGTTCGGCGTATTGTC
GGATCTTGAATACGATCGTGGAGGATTTGCCGTACTCGTCGCCGTTCGTGGCCACCGTCAACAATACCGTCGAGTACATACTCGTGTTTTCGGG
CGGAAGTGACGTTCTGCCGCCTCGCGTGACGGTGTCGTCGAATAGCGCGACCGTAGCCGCGCCGTTGATGATTGTCGCGGCACAACCGAAAAGT
TTACTGTCTTGGGACTTGCCGCTTCTGGTGGAAAGCGCTCAAGGCGTCCGAAGTTACATGGAATCTTCACGGACCCTATGCCTGGACATGTTTA
AGGGCTTACGCTCCGACAACAGCAACCTGATTGTGACGATATCGTCGGCTTCAAACAGTAACGTGGATTTCAAGTTGGTGGTCAATTCGAAAGA
GGAGTTCCGGTTGCACCACTCGGTCGAGTATAACATTACGATATCGCCCAGCGAGCCACAGTATTTCTTTTATAACTTCACTTCGAATTTGACG
GACGTCGTGTCCAACTTCGACACCGTCATTTTGGAGGTTACGTCGCAAGATTCCGTCTGCACGATCGTCAGTATTCAAAACGTTAGCTGTCCCG
TCCTTGATTTGAACCAGGACATCACTTTCCGCGGCTTTTACGAAACTTTCGACGCGAAAGGCGGCATCACTATACCGAAGGACAAGTTCCCGTT
CGGTTTTTTCGTGGTGTTCGTCGCGAAAGCCGACGACTCGCAATGCACGGGAACGCCCACTCCGACCAGTAACGATCGTACCAAGACGATCACG
CTCGTCATAAAGCCGAGCATCAGTTACAACGATTACGTGGTCGCGGTCATATACACGCTCTGCTCGATCGGCGCGTTCTACTTCGTGTTCGGCG
TGTTGGCGTTCGCGCCGTGCACGCGCCACTGCTGGTTCCCGCTTCGGCTCGACGACGACGACGACGCCGA
```

Protein RF 2: 422->1385 (321AA)

```
MFRPLVAILCLCSAYCRILNTIVEDLPYSSPFVATVNNTVEYILVFSGGSDVLPPRVTVSSNSATVAAPLMIVAAQPKSLLSWDLPLLVESAQG
VRSYMESSRTLCLDMFKGLRSDNSNLIVTISSASNSNVDFKLVVNSKEEFRLHHSVEYNITISPSEPQYFFYNFTSNLTDVVSNFDTVILEVTS
QDSVCTIVSIQNVSCPVLDLNQDITFRGFYETFDAKGGITIPKDKFPFGFFVVFVAKADDSQCTGTPTPTSNDRTKTITLVIKPSISYNDYVVA
VIYTLCSIGAFYFVFGVLAFAPCTRHCWFPLRLDDDDDA
```

Comparison with *Tribolium* Sid-1-related C precursor (768AA)

```
Query  5     LVAILCLCSAYCRILNTIVEDLPYSSPFVATVNNTVEYILVFSGGSDVLPPRVTVSSNSA  64
             L  I+   +  C   N I  +L YS+ +  ++N +VEYIL FS      PPRVT++S+ A
Sbjct  8     LFLIMSAVTVICDSFNPIYLNLSYSNFYTFSINKSVEYILEFSAPELKYPPRVTINSSDA  67

Query  65    TVAAPLMIVAAQPKSLLSWDLPLLVESAQGVRSYMESSRTLCLDMFKGLRSDNSNL---I  121
              +  PLM+VA QPK LLSW LP+++ES  G  ++ + SRTLC DM++   S    +   I
Sbjct  68    QIKTPLMVVARQPKELLSWQLPMVLESDTGNHNFTKISRTLCHDMYRDYASRGITVDSPI  127

Query  122   VTISSASNSNVDFKLVVNSKEEFRLHHSVEYNITISPSEPQYFFYNFTSNLTDVV-SNFD  180
             V++S+A+  NV F + V+ +++F +  SV+YN  I+PSEP+++FYNFT+N+T+   SN++
Sbjct  128   VSVSTAAPRNVTFTVQVDYQKDFFIKPSVKYNFNITPSEPRFYFYNFTANITESPNSNYE  187

Query  181   TVILEVTSQDSVCTIVSIQNVSCPVLDLNQDITFRGFYETFDAKGGITIPKDKFPFGFFV  240
             TVILEV S D VC  VSIQN SC V D NQDITFRGFYET + +GGITIPK KFP+GFF
Sbjct  188   TVILEVFSDDFVCMTVSIQNASCLVFDTNQDITFRGFYETVNTQGGITIPKYKFPYGFFA  247

Query  241   VFVAKADDSQCTGTPTPTSN-DRTKTITLVIKPSISYNDYVVAVIYTLCSIGAFYFVFGV  299
             VFVAK DDS CTG P+   + +RTKTITL++KPSISY DYV AVI TL SIG FYFV  +
Sbjct  248   VFVAKPDDSDCTGIPSLYYDTNRTKTITLIVKPSISYQDYVNAVIATLSSIGIFYFVL-I  306

Query  300   LAFAPCTRHCWFPLRLD  316
               F  C++  + P +++
Sbjct  307   AGFIFCSKRGYVPRQME  323
```

Graphical representation

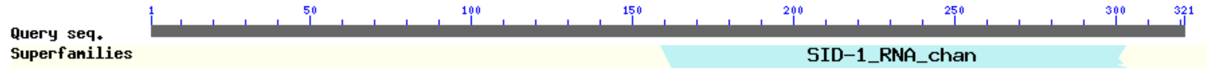

Nucleases in *Cylas brunneus*

Snipper = Eri1

>Cb.comp34051_c0_seq1 len=1020

cDNA

```
ATTTTCCTTGGGTTTAACCATAGATGAAAATGAAACATATAAAGGATATTATAAATGTTTTTCAATCAACAAACTTACATTTAAAAATTTTAAC
GTACAATGGATAAACCTCAAATAAAAACTTTAGCACTTGCAAGATCTCTGAATTGCATTGAAACCATATTTGCGGAAAATAAGACCTTTAGACG
CGAGAAACAATTCTTTGACTATTTAGTGGTATTAGATTTTGAAGCAACCTGTTGGAGTAAAACCGATTATGACAAGGGCCAATCTGAAATAATT
GAGTTTCCTTGTGTATTATATGATGTTTTGAATAACAAGATTATCTCAGAGTTCCAACAGTATGTTATGCCATCTGAAAGACCAAAATTAAGCA
CTTTCTGTACAGAGTTGACTGGTATACAACAGATACAAGTTGATGATGGTGTTCCTTTAAAAACATGTCTTGTATTATTCCACAGATGGTTAAA
TCAGCAAATTCTTAAGTACAATATATCTCTTAAATATCCCACATCATTTTGTAAAAGTTGTGTATTTGCAACATGGTCTGACTGGGATTTGGGG
AACTGTCTTAAAAATGAATGTAGAAGAAAAGGTATTATCAGACATGATATGTTCAACAGGTGGATTGATGTACGAAGTTTATATGTGGAGCATT
ATAATGTGAAACCCAAGGGTCTTTTGGGTGCGCTAAATGTAGTTGGACTAACCTTTGAAGGTACTCAACATTGTGGCCTTCATGATGCTAGAAA
TACCGCCAAGTTAATAGGCAAAATGGTGGATGATGGGGTAGGGTTAAGAATAACAAAAGACATATCAAATAATAAATAAATTTAATGTATTAAA
TAATAGAAAATATTTTTCACATGTCAATCGGATTTATCTGAAATTAGAAGTTTTTCCTGTTTCAATTTCCTTCTCTTCTTCCTTGCTTTATTGG
TACAATGATTTGCTTGAATTTCTAAAACACCACCATAATCTCCTGGTATGTTTGATATTAGTCTAGATGAGGAGTTTAAA
```

Protein RF 1: 100->831 (243AA)

```
MDKPQIKTLALARSLNCIETIFAENKTFRREKQFFDYLVVLDFEATCWSKTDYDKGQSEIIEFPCVLYDVLNNKIISEFQQYVMPSERPKLSTF
CTELTGIQQIQVDDGVPLKTCLVLFHRWLNQQILKYNISLKYPTSFCKSCVFATWSDWDLGNCLKNECRRKGIIRHDMFNRWIDVRSLYVEHYN
VKPKGLLGALNVVGLTFEGTQHCGLHDARNTAKLIGKMVDDGVGLRITKDISNNK
```

Comparison with *Tribolium* Snipper (232AA)

```
Query  6    IKTLALARSLNCIETIFAENKTFRREKQFFDYLVVLDFEATCWSKTDYDKGQSEIIEFPC  65
            + T  LAR L  +E I++  K      Q FDYL+VLDFEATCWS  D  K  +EIIEFP
Sbjct  1    MSTRELARKLGALEVIYSTAKA--TTPQPFDYLLVLDFEATCWSNGDPRKNPAEIIEFPV  58

Query  66   VLYDVLNNKIISEFQQYVMPSERPKLSTFCTELTGIQQIQVDDGVPLKTCLVLFHRWLNQ  125
            VLYDV N KII+EFQQYVMP E PKLS FCTELTGIQQ QVD+GVPL+ CL+LF RW+ +
Sbjct  59   VLYDVKNAKIIAEFQQYVMPVENPKLSDFCTELTGIQQHQVDNGVPLQACLLLFSRWVAE  118

Query  126  QILKYNISLKYPTS-FCKSCVFATWSDWDLGNCLKNECRRKGIIRHDMFNRWIDVRSLYV  184
            ++  Y++      S   K+C FATWSDWDLG CL+ EC RK I    M+ +WID+R+L+
Sbjct  119  KMSLYDMDFPNGESQATKTCAFATWSDWDLGTCLRKECIRKNIRIEKMYRKWIDIRALFK  178

Query  185  EHYNVKPKGLLGALNVVGLTFEGTQHCGLHDARNTAKLIGKMVDDGVGLRITK  237
             +      GL GAL  +GLTFEGT+HCGLHDARNTA+L+GKMVD GV L++T+
Sbjct  179  RYIRRPFIGLAGALAELGLTFEGTEHCGLHDARNTARLVGKMVDKGVVLQLTR  231
```

Graphical representation

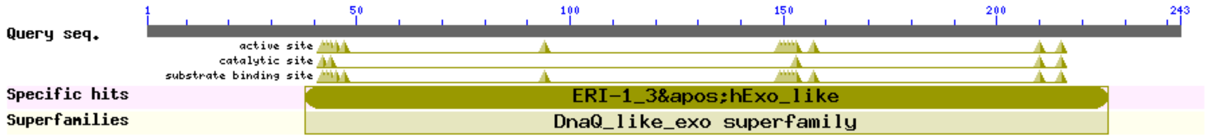

Nibbler

>Cb.comp42400_c0_seq1 len=2946

cDNA

AAATCGGTACCCGTCACTCTCCGATAGAGGACGCGCGTCTGTTGCAAAACGTGTTGCATACGCCACAAAGTTACAAAATCGAGGGAGCATTTTT
AAGCTGACGTACTAAGCGAAAAATGCTTGATGCCTTAGAAACGGTGTTAACTTTGCACGCAGATTCGTAAACCGACGCAATTTCATCGCGCAGG
GGACGTGTTCGACACTTATGCGTAATTTTCCCGACGACACCGTAACTAGATAGACCGGATATGTTTGCGAGGGGCAGAGGAAGAGCCGCTCACG
TCGGCCCCCGGCACGTCGCGGGACAAACAACCAGACCGGCCAAAAACATATCAACCCACGACAATAACTGCTGCAAAACTATCAGCCTAAACTT
GAGCTTGAACCCGGAGGATTCAGCGTTTTTCGACGAACTGAAGAGCATGTACAACATGGTAAAACGGAGCCCGCCGGTCGTCTCGAAGCTCGAG
CAGTATTTCGGGTCGTGCGCCGACCCTTACGAGCAAACGCTCCGCCTCCTCAACAACTGCCAGGACTTTTTTAACGCGAAAAACAAGAGCCTGC
CCATTTTCATAGCGGAAGAGTTCCGGCAGTGGAGCCGAGCGCGCCGAGACCGTATCTCGCACCTCCTAGTGCCCCAAATCAAACTGGACGCGTT
CAAATTGATCACGAAGCAAAATTTCCAAGGACTAACGAAACTCGTGTTCGACAGTTACGAAATGGCGCGACACCCTCACTTGTTCGTCGACTGT
CTGCGTTGCCTGATCGAGAACAAGCAGTACAAGGAGGCCTGCCAGTACGCGACGATGCTTAACCTCCACGGCGAATTCGGCGTGGACGACTTCC
TGGTGCCGCTGGTGCTCCAGGACAAGCTGTACAGCGTCGACGAGTTTCTCGCCGGAAGTCCGCGTCATCAGTTGGAGTTGGTGCGGTTCTTGGA
CTCCGCCCTCAGTCAGATGTCTGTCGGCGACACGATGAACCCGTACATCGAGGAGAAGGGCATACCGGACGTCAAGTACGACAAGGTGCACTCG
AAGCCTTGGAAGAAAATGGTGACGCGGTTCGCGAAAATGTTCAAGATGGCGCCGGAGCTGACGCCGTACCTGAACAAAAAACGGAACGAGGGCG
CGCTAAAGTTCCTCATCCACAAACGCTTCACCGAGAACACCTTCAGCGACGAGAGTTGGAGGGAGATGGTCCAGGAGGCGGTCGGCGACGACGA
CTCGTTGCGGAAGGAAGTCGTGCAGCTCGTAGCGCAGTACGGGGAGTTCCGCGAGGCGTTGTGGTGGGCGCGGTACTACGACGTCGACCGCAAC
GATTGGCCGTACAGCGTGCGCCTCCTGGACGAGAACAAGCTGGTGGAGGCGGAGCCGGCGACGCCCCCTGCCGACGATTGGGACGTCGAGACCG
CGAACAAGGTCGAGTACCACAAACTCGAGTTGGCGGTGGACCGCGTCGTCTTGGTCGACACCGTCGAGAAGTTTAGCGGCGTGGTCGACACGGG
ATTCGTGAACGCCGACATGGTAGGCATCGATTGCGAGTGGAAGCCGAGCTTCGCCGGTCAACTCAACGAACTCGCCCTGATGCAGATCTCCACC
AGGGAGTCGGTGTTCGTGCTCGACGTCGTCAATTTGGCGGGACGGGAGCGTCGCTCGTGGGAGACGCTCGGCCGCGACTTGTTCAACAATTGTG
ATATTTTAAAATTAGGTTTCAGCCTGACCAGCGACTTCCACATGATCCAGCAGGCGCTGCCCGAGCTTAATTTTTCGAGTGGGCGGGCCGGCTT
CCTCGACCTCTGCTCCCTCTGGAAGCACCTGGACAAGTTCCCCAAAGTCGTCCTGCCCAACGAAGTGCAGGGCGGCGGTCCGAGCCTCAGCACG
TTGGTGCAGGCGTGTCTCGGCCGGCCCCTCGACAAGTCCGAGCAGTTTTCGAACTGGGAGAACCGGCCGCTGCGCCAAAGTCAGATCCTCTACG
CGGCGCTGGACGCGTACTGCCTGATCGAGGTGTACGACGTGCTGAAACGTTGCTGCGAGTTGGCCGACTGTCCGTTCTACGAGATATGCGACAG
TTTGGTGAGCAGCGAGCGCGCGGCCAAAAAGAAACCGAAAAAAACGTCGGGCGGCAAGAAGCGGCACCACCACAACCGCGAGGAGGAGGCAGCG
CAGCCGCCCGGCCCGCACCCGACGCCGATTCGCGCCGAAGAGCTCAAGGTCGTGTGCGACACGATGCTGCAGGGTTTAGGCAAGAACCTGAGAC
GGTGCGGCATCGACACCGCCATATTGGAAAACCACCAGGATCACCAGCAGTGCGTCGCGTACCACGCGAAAGAAACGCGGTACATCCTGACCAG
GAAGGGGCCATTTAAGACGTTGAGCGGGCGCGTGCCTGCCGGACACTGCTTGAAGATCGTCTCGGACGACGTGGACGAGCAGCTGCAGGAGGTG
CTCGACTACTACAGGGTGTCGGTGACGAAGGACGACGTGCTCAGCCGTTGTCAGGCGTGCAACGGGAAGAGTTTCGCGGAAATCCCGCGGTCGA
CGATGTTGGCGCTGAACGCCGACGCGACGGCGACGTCCCCCAAGTACGTCCCCGCGTGTTACTACGATGACGAGTCGACGGGGTTCACGAGCGA
CGAGGATTTCGACGAAGACGCCCCCGGGCATAGTTCGACGAGGTCTGCGGCGGCGGCGGCGACGCCCGACGTCGGTGACCGCGTGACGAGACTC
GGGGTGACGATAAAGGCGGACCAGATCCCCAGGCCGGTTATCCTCAAATACGACGTCTTCTACGTGTGCGAGGAGTGCGGGAAAGTCTACTACG
ACGGCAGCCATTACGGGCGGCTGTTGAACGGCAGGCTCCAGGGCATCGTGCATTGAACGGCATTTACGATTCGCATGTTTTAATTTGTAACCGA
TTTTATTAAACGTTTTTTAAGTTAAAAAAAAA

Protein RF 3: 249->2876 (875AA)

MFARGRGRAAHVGPRHVAGQTTRPAKNISTHDNNCCKTISLNLSLNPEDSAFFDELKSMYNMVKRSPPVVSKLEQYFGSCADPYEQTLRLLNNC
QDFFNAKNKSLPIFIAEEFRQWSRARRDRISHLLVPQIKLDAFKLITKQNFQGLTKLVFDSYEMARHPHLFVDCLRCLIENKQYKEACQYATML
NLHGEFGVDDFLVPLVLQDKLYSVDEFLAGSPRHQLELVRFLDSALSQMSVGDTMNPYIEEKGIPDVKYDKVHSKPWKKMVTRFAKMFKMAPEL
TPYLNKKRNEGALKFLIHKRFTENTFSDESWREMVQEAVGDDDSLRKEVVQLVAQYGEFREALWWARYYDVDRNDWPYSVRLLDENKLVEAEPA
TPPADDWDVETANKVEYHKLELAVDRVVLVDTVEKFSGVVDTGFVNADMVGIDCEWKPSFAGQLNELALMQISTRESVFVLDVVNLAGRERRSW
ETLGRDLFNNCDILKLGFSLTSDFHMIQQALPELNFSSGRAGFLDLCSLWKHLDKFPKVVLPNEVQGGGPSLSTLVQACLGRPLDKSEQFSNWE
NRPLRQSQILYAALDAYCLIEVYDVLKRCCELADCPFYEICDSLVSSERAAKKKPKKTSGGKKRHHHNREEEAAQPPGPHPTPIRAEELKVVCD
TMLQGLGKNLRRCGIDTAILENHQDHQQCVAYHAKETRYILTRKGPFKTLSGRVPAGHCLKIVSDDVDEQLQEVLDYYRVSVTKDDVLSRCQAC
NGKSFAEIPRSTMLALNADATATSPKYVPACYYDDESTGFTSDEDFDEDAPGHSSTRSAAAAATPDVGDRVTRLGVTIKADQIPRPVILKYDVF
YVCEECGKVYYDGSHYGRLLNGRLQGIVH

Comparison with *Tribolium* hypothetical protein TcasGA2_TC002596 (1249AA)

```
Query  56    LKSMYNMVKRSPPVVSKLEQYFGSCADPYEQTLRLLNNCQDFFNAKNKSLPIFIAEEFRQ  115
             LK + + VK+SPPVV+KL QYF  C +PYE T+RL+ NCQ+F +AK+KSLP FI EEF+
Sbjct  405   LKCLLSTVKKSPPVVNKLHQYFHLCENPYEHTIRLMYNCQEFNSAKSKSLPFFIIEEFKI  464

Query  116   WSRARRDRISHLLVPQIKLDAFKLITKQNFQGLTKLVFDSYEMARHPHLFVDCLRCLIEN  175
             W    R+++ HLL P++K+D FK+I+KQN Q LTKLV D YEMA+   +F+D ++C+IE
Sbjct  465   WLSVHRNKVVHLLTPKLKIDVFKIISKQNAQNLTKLVVDVYEMAQDGEIFLDIIKCMIER  524

Query  176   KQYKEACQYATMLNLHGEFGVDDFLVPLVLQDKLYSVDEFLAGSPRHQLELVRFLDSALS  235
             K+YKEACQ A + NL  +F V+DFL+PL+LQDKLY +D+FL  SPRHQ+ELV  LDS L
Sbjct  525   KRYKEACQSAVLFNLQDKFSVEDFLLPLILQDKLYGIDDFLTVSPRHQVELVTLLDSTLG  584

Query  236   QMSVGDTMNPYIEEKGIPDVKYDKVHSKPWKKMVTRFAKMFKMAPELTPYLNKKRNEGAL  295
             + SV D +  Y+    +PD+K+DK+H+KP KK++TR  KMFK+   +TP LNK+RNEGAL
Sbjct  585   RTSVRDALASYVFNLDVPDIKWDKLHAKPLKKLITRLVKMFKLPTNITPNLNKRRNEGAL  644
```

```
Query  296   KFLIHKRFTENTFSDESWREMVQEAVGDDDSLRKEVVQLVAQYGEFREALWWARYYDVDR  355
             +FL+HKRF EN+F DESW+EMVQEA+G+D+ L++E+V  V+ YG   EALWWA +Y+VD+
Sbjct  645   QFLLHKRFVENSFGDESWKEMVQEAIGEDEELQRELVAQVSTYGAVAEALWWAHFYNVDK  704

Query  356   NDWPYSVRLLDEN---KLVEAEPATPPADDW---DVETANKVEYHKLELAVDRVVLVDTV  409
               WPY+VR+L+EN   + +      P  + W   +V+    VEYHK  L    + L+D+
Sbjct  705   QHWPYNVRMLEENPDEERLHQRNILPEEESWGYDEVQNTEPVEYHKFPLPFSSIHLIDSE  764

Query  410   EKFSGVVDTGFVNADMVGIDCEWKPSFAGQLNELALMQISTRESVFVLDVVNLAGRERRS  469
             E F   +D G  + ++VGIDCEWKP+F  Q NELALMQI++R++VF+LD++++  +
Sbjct  765   ESFERFLDGGLQDVEVVGIDCEWKPNFGSQKNELALMQIASRKNVFILDIISIGTKVPHL  824

Query  470   WETLGRDLFNNCDILKLGFSLTSDFHMIQQALPELNFSSGRAGFLDLCSLWKHLDKFPKV  529
             W+ LG+ LFNNCDILKLGF  TSD  MI+ +LPELNF+  + GFLDL SLWK L+K+PKV
Sbjct  825   WQELGKFLFNNCDILKLGFGFTSDILMIKHSLPELNFTPKQVGFLDLLSLWKLLEKYPKV  884

Query  530   VLPNEVQGGGPSLSTLVQACLGRPLDKSEQFSNWENRPLRQSQILYAALDAYCLIEVYDV  589
             VLP EVQG GPSL TLV  CLGRPLDKS+QFSNWE RPLR SQ++YAALDAYCLIEVYDV
Sbjct  885   VLPYEVQGSGPSLGTLVNQCLGRPLDKSDQFSNWEKRPLRNSQLVYAALDAYCLIEVYDV  944

Query  590   LKRCCELADCPFYEICDSLVSSERAAKKKPKKTSGGKKRHHHNREEEAAQPPGPHPTPIR  649
             +K CCE A+ PF E C +L+++E+A KKK KK    K +     +EE AQPP PH + +
Sbjct  945   IKGCCEKAEFPFDETCYNLMTNEKAPKKKAKKPVQKKPKPLQ-ADEEIAQPPSPHSSQVP  1003

Query  650   AEELKVVCDTMLQGLGKNLRRCGIDTAILENHQDHQQCVAYHAKETRYILTRKGPFKTLS  709
             A  +KVVCDTMLQGLGKNLRRCGIDTAILEN+ DH +CV Y   E RYILT+   F  L
Sbjct  1004  AASIKVVCDTMLQGLGKNLRRCGIDTAILENYMDHMECVRYAQDEQRYILTKGNVFNKLY  1063

Query  710   GRVPAGHCLKIVSDDVDEQLQEVLDYYRVSVTKDDVLSRCQACNGKSFAEIPRSTMLALN  769
             G VP GHCL++ SD+VDEQL+E +DYY+V+VT +DV S CQ+CNG+SF ++ RSTMLAL
Sbjct  1064  GYVPLGHCLRVNSDNVDEQLKEFVDYYKVNVTVNDVFSVCQSCNGRSFIKVSRSTMLALT  1123

Query  770   ADATATSPKYVPACYYD--DESTGFTSDEDFD-EDAPGHSSTRS----------------  810
                +  S +YVP  Y +  DE+TGF+SD+DFD E  P   +TR
Sbjct  1124  --QSQNSLQYVPPDYDNDIDEATGFSSDDDFDFEPGPPVQTTRKWDLCMHFTYNYYFSLT  1181

Query  811   ---AAAAATPDVGDRVTRLGVTIKADQIPRPVILKYDVFYVCEECGKVYYDGSHYGRLLN  867
                  +    DVG   TRLG  I+   IP  V+ K ++FYVCE CGK+++DGSH  R+L
Sbjct  1182  FIILDSDEKLDVGLCQTRLGAKIQVATIPDGVLEKTELFYVCEHCGKIFWDGSHLERVLT  1241

Query  868   GRLQGIVH  875
             GRLQGIV
Sbjct  1242  GRLQGIVQ  1249
```

Graphical representation

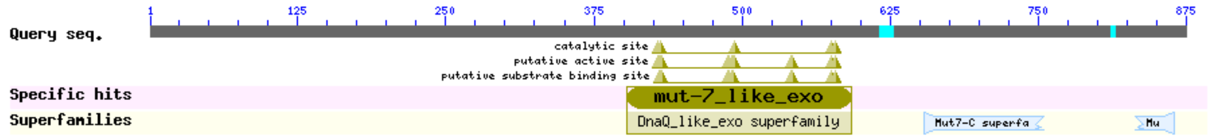

Sdn1-like (small RNA-degrading nuclease 1)

>Cb.comp38443_c0_seq2 len=2149

cDNA

```
TCATTTGTTCTGGTATCTAACCTTAAACAATAACATTCAAATTCAAAAACACAGAATAGGGTATGATCCGATACAATTTCAAATTTTTTATTGA
TGTAGCAAAAACAGTTTTGTCAATTCATATTTTAATATCTGTTTGTTATTGGTTGTTATATGGCAGCAGAATGACAGGGCCCACAAACGTTTCG
TCAAAAAGAAAATTAAGACTCGAAAATAAGAAGAAAAAAATGGCTGCACTTTTAGACATTGCAAAACTAAACGAATGTGACAGACAGAAAAACA
AGTTACACCAACAGGACCATCACAAAGAATCAAATAATACAATGGTGGCTGCAGAACCCAGTGCAAAGAAATTTAAACCAGAACAAAAAGAAAA
TGGATCAGCAGTTGTCAGTTCCGGAAAACCGAAGCTTGAAGGAGAAGCATTTGAAGAATTGAAAAGAATGCTCAGAGAAAAAACAAATCAGATT
CGTAATTGTCCAAAGTTTCGGCTCAGAGATATGGGCGACAGTGCCACTCTGAAGATCCCACTAGATAACCGTTCACCATTATTTCTCTCTGACA
TTCAGCACCTTATTATGTACTCCCAAGTTGGAGTACATTCTCCATACTCACCAACAAGATGGTGCTCTCTCGAAAAGTACAACAGACTGAGGAG
TGTAAACTTATTAATTGTGGAGAATGTCTCCTTGTACCATTATGAAGCATTTGAGAGTGAGTTTGAATTTTTGAAATCCAATCTTGAACACAAA
CTTGAATTTGTATCACCTTTATCTTACCGTGGAGATATTATTAAGGAACTGTCTATGGTACCCCTCTCCGCAACGCAGATGCGGAAATTAATCA
ACGAGTACGGGAGTATGGCAGACGCCGCGAAAAATTGTACCGAAATATTTGACACCGTTAAAAATTTTTTCCCCATCGACGAGTGCCCTGACGC
AAATGGGGAAACGATCGCAGGGTTGCCAACATCCGACAAATTTTCCAGGACTCAATTATTACTGTCAGGTTGGCAAATGATTGAAGAAAATTTT
CCGTTGCCCATCAAGGGGCTCGTTGAAAGGAAATATGCCGATTACAAATTAACTAAAGAGAGGTATAAAAACGTCACGCCCACCAGTCCAATGG
TGGGGATTGACTGCGAGATGTGCAGGACCAGTACAGGCGAACTTGAGTTAACGAGAGTATCAGCTGTCAACGAGAAGCACGAGGTATTCTACGA
TACATTGGTGAAACCAGACAATAAAATTGTCGACTATTTGACTCGATTTTCCGGGATAAATTCAAAGATGATGAAATCGGTAACGAAAAAACTA
AAAGACGTGCAGAACGATCTCAGGAAGCTGCTACCAGATGACGCCATTTTAGTCGGCCAGTCTTTGTCTAACGACTTGCACGCGTTAAAAATGA
TGCATCCTTATGTGATAGACACTAGCGTAATTTACAATATGACCGGAGACCGCGCCCGCAAATCCAAACTGCAAACTCTGGCGAAGGAGTTCCT
GGACGAAACGATCCAATCGGGTCACGGGCACTGCTCGAACGAGGACAGTCTCGCGTGCATAAAACTCGCGCAGCTCAAACTCAAAAAGCACCTT
TATTTCGGCGACGCGATCATGGGCTCGATACTTAGCGAACAAAGGGCGTATCCCGACATCGGAACATCCAGCTACGCCACCAGTATGCTACGGC
AGTGCACCAAAGTAGATAAAACGGCGAGCGTCGTCGGCATCGACGACACCGCCGACAAATATAAATTTTACGTGGACAAAAATCTGAGCCGCGA
TATAACCAACATCGCGTTCAAGTCCGAAACGTCGAACAGGGGCGTAGTGAAACAGTTCTGCGAGAACGTTAACCGTTATTCATTGAACATCGGT
CAGATTAGGATATCGGAAAGCGAATTGGACAATTCGAACCACGCGTTTCGAACTCTCGATAAGTGGATAGGGGAAGTTTACGCGTGCGCAGAGA
TGCCGACGTTTTTGGCGGTGCTCTTCGGCGGTCAGAAAGAAGGCGGGAACGGGGCGTGCTTCCTGCAACTGCACAGGGAATTCGTATAATTTAA
AACGGCGCGGAAATTCGCGCCGTTTTGTACATATTACGTCGTTAATTCGATTAAACGCATTTTTTTCCTCTGTTAAAATAA
```

Protein RF 3: 63->2063 (666AA)

```
MIRYNFKFFIDVAKTVLSIHILISVCYWLLYGSRMTGPTNVSSKRKLRLENKKKKMAALLDIAKLNECDRQKNKLHQQDHHKESNNTMVAAEPS
AKKFKPEQKENGSAVVSSGKPKLEGEAFEELKRMLREKTNQIRNCPKFRLRDMGDSATLKIPLDNRSPLFLSDIQHLIMYSQVGVHSPYSPTRW
CSLEKYNRLRSVNLLIVENVSLYHYEAFESEFEFLKSNLEHKLEFVSPLSYRGDIIKELSMVPLSATQMRKLINEYGSMADAAKNCTEIFDTVK
NFFPIDECPDANGETIAGLPTSDKFSRTQLLLSGWQMIEENFPLPIKGLVERKYADYKLTKERYKNVTPTSPMVGIDCEMCRTSTGELELTRVS
AVNEKHEVFYDTLVKPDNKIVDYLTRFSGINSKMMKSVTKKLKDVQNDLRKLLPDDAILVGQSLSNDLHALKMMHPYVIDTSVIYNMTGDRARK
SKLQTLAKEFLDETIQSGHGHCSNEDSLACIKLAQLKLKKHLYFGDAIMGSILSEQRAYPDIGTSSYATSMLRQCTKVDKTASVVGIDDTADKY
KFYVDKNLSRDITNIAFKSETSNRGVVKQFCENVNRYSLNIGQIRISESELDNSNHAFRTLDKWIGEVYACAEMPTFLAVLFGGQKEGGNGACF
LQLHREFV
```

Comparison with *Tribolium* PREDICTED: similar to CG8368 CG8368-PA (631AA)

```
Query  41    VSSKRKLRLENKKKKMAALLDIAKLNECDRQKNKLHQQDHHKESNNTMVAAEPSAKKFKP  100
             + SK   R+ENKKKKMAAL++I++LNE DR   K     + +  +++ +   EPS KK +
Sbjct  1     MKSKSTKRIENKKKKMAALIEISRLNEYDRNLKKTQISEANGSNSSEL---EPSVKKPRT  57

Query  101   E--------QKENGSAVVSSGKPKLEGEAFEELKRMLREKTNQIRNCPKFRLRDMGDSAT  152
             E        QK     +  SGKPKL G   +ELK+MLREKT ++R  P F+LRDMG +A+
Sbjct  58    EAPIGDTVEQKTLLPELGPSGKPKLSGLELQELKKMLREKTTKMRQQPVFKLRDMGTNAS  117

Query  153   LKIPLDNRSPLFLSDIQHLIMYSQVGVHSPYSPTRWCSLEKYNRLRSVNLLIVENVSLYH  212
             L   L+NR PLFLSD+QHLIMYSQ+G H+PYSP RWC+LEK+N+L +  LL+VEN+++ H
Sbjct  118   LSTDLENRVPLFLSDLQHLIMYSQLGHHAPYSPARWCALEKFNKLSTTCLLVVENMTVNH  177

Query  213   YEAFESEFEFLKSNLEHKLEFVSPLSYRGDIIKELSMVPLSATQMRKLINEYGSMADAAK  272
             Y   E+ F F+ S  EHKLE ++P S   D+++ELSMVPL+ATQ++K   ++G++ DA
Sbjct  178   YTTHENIFPFVSSTFEHKLEILAPNSSNSDVVRELSMVPLTATQVKKFSTKFGTLEDAVH  237

Query  273   NCTEIFDTVKNFFPIDECPDANGETIAGLPTSDKFSRTQLLLSGWQMIEENFPLPIKGLV  332
               TE+FD+V++ FPI++  ++       LP +D+F RTQLLLSGWQM+EENFPLPIKGL+
Sbjct  238   RTTEVFDSVRSLFPIEKDKESKNGLSMDLPFTDRFPRTQLLLSGWQMVEENFPLPIKGLM  297

Query  333   ERKYADYKLTKERYKNVTPTSPMVGIDCEMCRTSTGELELTRVSAVNEKHEVFYDTLVKP  392
             E KYA Y LTK+RY++VTP S M GIDCEMC+T+ G+LELTRVS V+E    FYDTLVKP
Sbjct  298   ETKYAGYVLTKDRYEDVTPFSKMFGIDCEMCKTTIGDLELTRVSVVDEHLNTFYDTLVKP  357

Query  393   DNKIVDYLTRFSGINSKMMKSVTKKLKDVQNDLRKLLPDDAILVGQSLSNDLHALKMMHP  452
             DN+I DYLTRFSGI  KMM+++T +LKDVQ+DLR+LLP DAILVGQSL NDLHALKMMHP
Sbjct  358   DNRITDYLTRFSGITYKMMRNITTRLKDVQDDLRRLLPADAILVGQSLGNDLHALKMMHP  417
```

```
Query  453  YVIDTSVIYNMTGDRARKSKLQTLAKEFLDETIQSGH-GHCSNEDSLACIKLAQLKLKKH  511
            YVIDTSVI+N+TGDR+RK+KL+TL +EFL E IQ G  GHCS EDSLA +KLAQLKL+K
Sbjct  418  YVIDTSVIFNITGDRSRKTKLKTLTEEFLSEKIQEGQGGHCSTEDSLASLKLAQLKLRKS  477

Query  512  LYFGDAIMGSILSEQRAYPDIGTSSYATSMLRQCTKVDKTASVVGIDDTADKYKFYVDKN  571
            LYFGDA+MG++ +E R  P++GT +YATSML+Q TK+DKTA VV  ++    KYK+ VDK
Sbjct  478  LYFGDAVMGNVHNEIRTCPELGTYNYATSMLKQTTKLDKTALVVCPEEITSKYKYCVDKG  537

Query  572  LSRDITN--IAFKSETSNRGVVKQFCENVNRYSLNIGQIRISESELDNSNHAFRTLDKWI  629
                  N  I F SE S + VV++ C+++  +SLNIG +++ E +L+ +   F+ +DKW+
Sbjct  538  AEVRQQNEKIKFFSEKSCKEVVRKMCDSLGMFSLNIGHVKLQEGQLEGTK-VFKNVDKWV  596

Query  630  GEVYACAEMPTFLAVLFGGQKEGGNGACFLQLHRE  664
             E+Y      P  + VLF G  EG NG CF+QL R+
Sbjct  597  KEIYEKMPTPGLVIVLFPGV-EGSNGCCFIQLKRD  630
```

Graphical representation

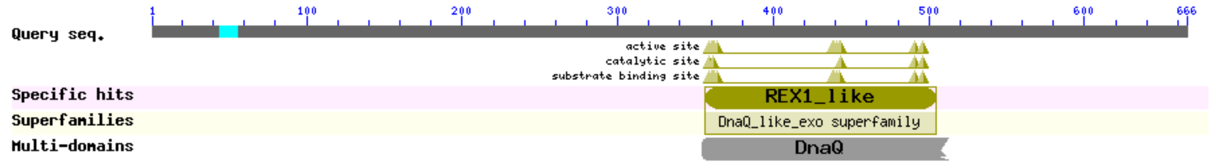

>Cb.comp40516_c0_seq1 len=3610

cDNA

AGCAATTTAATTAATTTGAGGTTATCGAAATGGTTTTTTAACATCAACAGTTATTTGGATATGAAAATCTTAGCAAGAAACGAAAAAATAGCAT
AAAACTATGCTGCCGACGAAAGGATATTTTCAGGACATTCAATGCCCTTTTAATGATACATCTTGTGGTCGGCCTTACTGTCATTTTAGGCACA
GGAAAAGGCCAGCTGAAAACCTAGAGGAATCTATTGCAGAAACCTCCAAGTCAAGTGTACCTATTTACAAACCTACCCCTAAAACTGAATTAGA
GAACATTCAAAATAAAACGCACATACCGATAAGTTATGTACCTGACCTGGCCTTTAGGAATGATCGACCATTAAGAACTTTTCCAAAATTTGAG
AAACCAACTTATAAGCCGACACCTTTAAGTTTACTTTCATCAGCCATCAGTAAAAGTGCTTTGTCTGATCACAATGAGAAGCAACAGGAAACAA
TCAAAGATATACAACAAAACATTGCCAATAATGAATATGATCCACTGAAATCTGAAATTAACTTTGAAGATCTGAGTAGTGAATTCGATTTAAT
AGATGAGATTATTAATGAAGATGAATCTGATCACACAGAGCCAGAAACTTTCATATCAGAAAATTTAACTAAGATAAATAGTGATATAAAAAAG
GAACAGAATCGATTAACTTTGTTATTAAAAAATGATTTAAATACTGATTCCAACATTGAAAAAATAAATAAAAAAGAAAAGATTGCAATAGAAA
CAAATGTGGGAGCTAAAATGGTAACCTCTGAAAACAATGAACAAGTTAATGTAAAGGGGGAAAAAAATGCAAAGGACTTAAAAAAATCAGAAAC
TGATGTAAACAGAAAAGAGAAAAGGACTGATAAATGGGATAAAAAAACAGGTAACAGTGACAAAAAAGTTAAAGTGGAAAAAATCAAAACTAAT
GAATTATCTCAAGAAAAAAAAGGCAGTTCCGAGAAGTCTCAGCTCAAGATGAAAGATAGGGATGAAAAAAAATGTGACTCCAAATCTCGCGAAC
GTGAAAAACATCGTAAAGAAGAAAAAAGGGAAAGCAAAGCTAATCAGAAACATGAACATAAAACCCAGGATCGGAGTAAAAGTGCAAAGGAAGA
CAAAAGCAATAAAAGAAAGACTTCTAGTGATAATAGTGACAGTGAGAAACCCAATAGAAGGGACAAGAGTAGAAGCAGTAGAGAACAAAAACAT
AAAAAGCGTAATCATAGCAGGAGGAGAAGCAAAACCAGGGAAAAAGAAAGAAGTAACCATAAAAGGAAAAAAGAAACAGAGAGTGCTGAAATGG
CGCGGAAAAAATCTTGTAGTTCTGGAAGCGACAGTGAGCGTAGCACTGTTTCTGCAAGGGGTAAAAATAATAATTCTGTTAGTGATAGATATAA
ACATGGTGCTAAAAAAAAGTCTGTTACCTCAGAAGTGGAGTCAAAAAGTCTGTTTAAGGGGAAACCTAGTAAAGTTAGCAATGAGAGAACATAT
AAACCAACTGTTTGTGATAGTGATAATGACAATGACCTAATTTCAATTGATGCTGATGAACTAGGACTGTCTGATATTGACTTAGACTTGGAAG
ACGAAGATGAAACAATGAGAGAATGTTTTAGGATATTTAATGAATATAAGCCTAAGCCCTTGAGGATCCCTTCACCCAAGACTGAAGAAACAGA
TAAAAGTCAATTAATTGAGGATGAATACCATTCAGCTTCAGTTAAGAGACGTGTGGCCCATAGTGGTGCTGAAAACTCTAAGTTGCTATCGGTA
CCGGTTGCCTCTAAAACAAAACCAATTATAACTCCTGGTCAAATTATGAGCAACCGGTATAAAATCGCTAAACTAGCCCAGGCGAATAATGAAC
AGGAAAACATTATGAATGAAGTTAGACAAATTACCGCAATACGATCGGCTCCAAGTCTATTGGAGGCTGCCAGAATGCATAAGTTGCGTAGACT
GGAACGTCAACAACAACTCCAAAAAGCTGCGCAGAAGCCCTCGACGAACGTTGTAGACGATATAATAAATGGAGTTCAAAAACCGTGCGCCTCG
AAAGTTAAACTTCCCGTAAAGAGAATCGCAGCCGTGCCGAACGTTGCGTTAATTGAAAAGGCCAAAGAGCGTATTTCTTTAATAAAGCAGCGTC
GGCTCGAGGTCCCAAAAACGGTTGCGCAGACGCAGAAAAGTGGTCGCGTGGCGCATGTCCCCGAAGTGTCCCTGCCCGACATTCCAGACGTTTT
ACAGGCTGATAAGTCGAAGCTGCCTGTAAATGTTAGAACCCGATTTTTAACAATGATAGTCGAAGAATGTTTCAAGCTCTATATTGCGAAACAG
GACGCTTACGCTAGAGCGTTAAGCGAAGAGTTTTCTTGTTACGAAAAATGCAAAGTGCTGTCGACTTACAGGAATTCGGCAATGCTTGCGGTAA
ACAGGCTGAGAAAAGAAATCCAGGACCGGGAAAATCGCAATTTGGGACCTTTGTTAAGCGGCGAATCGTCGTCTACCGATAAAGACTCAATTTT
TAGGGGCAGAAAGTTTTACGATCATGTAAAGAAATGGGTGCTCACTGAAGATGAACTCGATTTGCATGGTTACCCACGAGAAAGCAGGGAGAAG
GGGAAAGCAGTTATTAATAATCAAAAAGATGTGGACTATTCTATTGTAGACGAGAATTTAAGAAAATGCAGCAGGTGTTCGAAAATATACCAGG
TAGATGACGACGGCTGGCCCTTATTTGAGGAGGAGTGCATGTATCACCCGCTCAAAAAGCGAACGATAAGAGGTGAACAGGTGTTCTTATGCTG
TAAAAGTACCGACGAAACGGGCTGCGTGACATCTGACACTCATGTTTGTGAGGGATCCGATAGCCATCAGCTTGAAGGCTATCAAACTACACTC
CCCCCAGAAAGGGAGAACGACCCCAGAAGTTGCGCGGTCTACGCATTGGACTGCGAAATGTGTTATACTACCAAAGGTTTAGAGTTGACTAGAG
TGACCATAGTGGATCCTGATTGCAAAACCATCTATGAAAGTTTAGTGAAACCCCTAAACCCGATTGTTGATTATAACACTCGATTTTCGGGCAT
TACGAAGGAACAGATGGATAGAACCAGTACCAGTATATTACAGGTTCAGGCTAATATTTTGCATTTGTGCAACTCTGAGACTATTTTAGCCGGA
CATAGTTTAGAGTCGGACATGAAAGCATTAAAAATTGTGCATAGTTCTGTTATCGATACATCAGTGTTGTTTCCGCATAAAATGGGACTACCCC
ACAAACGGGCATTGCGCGCGTTAGCCAGTGAATACTTAAAAAAAATTATTCAGAATGATGTCAGTGGACACGACAGTGCCGAGGACGCCATCGC
TTGCATGGAGTTAATTAAATGGAAGTTGAAGGAAGAGTGCAAAGTGCGTACAAAATAGCTGAGGTGTCAAAACATTTACGTGGACTTTCTTTTT

AAGTTATTTATTTGTACCGGATTCAAGTACACCACCATACTAATAATAAATGTAAATACATTAGTATTATAAATTGTCAAGTCAATTGGTTAGC
TGTTCGGTTCAGAATATATTGTTTCGTATATTTATTAA

Protein RF 2: 101->3442 (1113AA)

MLPTKGYFQDIQCPFNDTSCGRPYCHFRHRKRPAENLEESIAETSKSSVPIYKPTPKTELENIQNKTHIPISYVPDLAFRNDRPLRTFPKFEKP
TYKPTPLSLLSSAISKSALSDHNEKQQETIKDIQQNIANNEYDPLKSEINFEDLSSEFDLIDEIINEDESDHTEPETFISENLTKINSDIKKEQ
NRLTLLLKNDLNTDSNIEKINKKEKIAIETNVGAKMVTSENNEQVNVKGEKNAKDLKKSETDVNRKEKRTDKWDKKTGNSDKKVKVEKIKTNEL
SQEKKGSSEKSQLKMKDRDEKKCDSKSREREKHRKEEKRESKANQKHEHKTQDRSKSAKEDKSNKRKTSSDNSDSEKPNRRDKSRSSREQKHKK
RNHSRRRSKTREKERSNHKRKKETESAEMARKKSCSSGSDSERSTVSARGKNNNSVSDRYKHGAKKKSVTSEVESKSLFKGKPSKVSNERTYKP
TVCDSDNDNDLISIDADELGLSDIDLDLEDEDETMRECFRIFNEYKPKPLRIPSPKTEETDKSQLIEDEYHSASVKRRVAHSGAENSKLLSVPV
ASKTKPIITPGQIMSNRYKIAKLAQANNEQENIMNEVRQITAIRSAPSLLEAARMHKLRRLERQQQLQKAAQKPSTNVVDDIINGVQKPCASKV
KLPVKRIAAVPNVALIEKAKERISLIKQRRLEVPKTVAQTQKSGRVAHVPEVSLPDIPDVLQADKSKLPVNVRTRFLTMIVEECFKLYIAKQDA
YARALSEEFSCYEKCKVLSTYRNSAMLAVNRLRKEIQDRENRNLGPLLSGESSSTDKDSIFRGRKFYDHVKKWVLTEDELDLHGYPRESREKGK
AVINNQKDVDYSIVDENLRKCSRCSKIYQVDDDGWPLFEEECMYHPLKKRTIRGEQVFLCCKSTDETGCVTSDTHVCEGSDSHQLEGYQTTLPP
ERENDPRSCAVYALDCEMCYTTKGLELTRVTIVDPDCKTIYESLVKPLNPIVDYNTRFSGITKEQMDRTSTSILQVQANILHLCNSETILAGHS
LESDMKALKIVHSSVIDTSVLFPHKMGLPHKRALRALASEYLKKIIQNDVSGHDSAEDAIACMELIKWKLKEECKVRTK

Comparison with *Tribolium* (AA)

```
Query  1     MLPTKGYFQDIQCPFNDTSCGRPYCHFRHRKRPAENLEESIAETSKS-SVPIYKPTPKTE  59
             MLPTKGYFQDI+CP+ D++C RPYCHFRHRK+  E +EE   ET K   VP YKPTPK+E
Sbjct  1     MLPTKGYFQDIECPYFDSTCNRPYCHFRHRKKTQETIEEVANETPKEVEVPTYKPTPKSE  60

Query  60    LENIQNKTHIPISYVPDLAFRNDRPLRTFPK--FEKPTYKPTPLSLLSSAISKSALSDHN  117
             L NI  K+HIPISYVPDLAFR+DR +R  PK  FEKPTYKPTPLS+LSSA  +  + + +
Sbjct  61    LANI--KSHIPISYVPDLAFRSDRTIRPLPKFTFEKPTYKPTPLSILSSASKRENVLEDD  118

Query  118   EKQQ---ETIKDIQQNIANNEYDP---LKSEINFEDLSSEFDLIDEIINE  161
             E++    E I++++QNIAN+EY+P   L+ +INFEDLS+EFD+ID++I E
Sbjct  119   ERETSDIEAIREVKQNIANDEYNPEISLQDDINFEDLSAEFDMIDDLIEE  168


Query  481    LISIDADELGLSDIDLDLEDEDETMRECFRIFNEYKPKPLRIPSPKTEETDKSQLIEDEY  540
              L S + DE+   D D   +DE++T+ EC++IF EY+P  + +  P   E    ++I++E
Sbjct  315    LYSNNFDEIPALDFD---DDEEDTLSECYKIFKEYEPPKVEVKEPPPAE---PEVIKEET  368

Query  541    HSASVKRRVAHSGAENSKLLSVPVASKTKPIITPGQIMSNRYKIAKLAQANNEQENIMNE  600
              +++  K+R+AHS A  S+  S     K K    P Q M+NR+K+AKLAQANNEQ+N+MNE
Sbjct  369    NAS--KKRIAHSSANPSEGGSKINYVKPKIQANPAQAMANRFKLAKLAQANNEQKNLMNE  426

Query  601    VRQITAIRSAPSLLEAARMHKLRRLERQQQLQKAAQKPS--TNVVDDIINGVQ-KPCASK  657
              V+Q T  R APSLLEAAR +KL+RL        A  KPS   NV+D I+N  + KP
Sbjct  427    VKQ-TVKRPAPSLLEAARNYKLQRL--------AKPKPSENANVIDSILNSAKNKP----  473

Query  658    VKLPVKRIAAVPNVALIEKAKERIS-LIKQRRLE-VPKTVAQTQKSGRVAHVPEVSLPDI  715
                   K+IA V NV  I++AK RI  L KQ+  E + KT AQT K  R+AHVP++SL DI
Sbjct  474    -----KKIAPVQNVNSIQRAKARIEELAKQKATETLNKTPAQTVKGKRIAHVPDISLSDI  528

Query  716    PDVLQADKSKLPVNVRTRFLTMIVEECFKLYIAKQDAYARALSEEFSCYEKCKVLSTYRN  775
              PDVL ADKSKLP+NVRTRFLTMI +EC KLY+ K+DAY RAL+EEF CYEKCKVL+TY+N
Sbjct  529    PDVLNADKSKLPINVRTRFLTMIADECVKLYLIKEDAYTRALNEEFVCYEKCKVLATYKN  588

Query  776    SAMLAVNRLRKEIQDRENRNLGPLLSGESSSTDKDSIFRGRKFYDHVKKWVLTEDELDLH  835
              SAMLAVNRLRKE+Q+R+   LGP+  GE+ + D  S ++G KFY+H+K + LT +ELD+H
Sbjct  589    SAMLAVNRLRKELQERDRLGLGPIGEGEAPANDTASNYKGAKFYNHIKGYALTNEELDIH  648

Query  836    GYPRESREKGKAVINNQKDVDYSIVDENLRKCSRCSKIYQVDDDGWPLFEEECMYHPLKK  895
              GYPRES   G+A I N+K   +S + EN RKCSRC KIY VD+DG+   + EEC+YHPLKK
Sbjct  649    GYPRESATPGRATIKNRKTTAWSSLRENQRKCSRCGKIYLVDEDGFVQYPEECIYHPLKK  708

Query  896    RTIRGEQVFLCCKSTDETGCVTSDTHVCEGSDSHQLEGYQTTLPPERENDPRSCAVYALD  955
              RT+RGEQ +LCCKS D+ GC TS+THV E     +LEG+QTT+ PE E DPRS AVYALD
Sbjct  709    RTLRGEQTYLCCKSNDDVGCATSNTHVSEACGDAELEGFQTTMEPESEEDPRSQAVYALD  768

Query  956    CEMCYTTKGLELTRVTIVDPDCKTIYESLVKPLNPIVDYNTRFSGITKEQMDRTSTSILQ  1015
              CEMCYT KGLELTRVTIVD +CKT+YE+LVKPLNPI+DYNT FSGITKEQM+RTSTSILQ
Sbjct  769    CEMCYTIKGLELTRVTIVDSECKTVYETLVKPLNPIIDYNTTFSGITKEQMERTSTSILQ  828

Query  1016  VQANILHLCNSETILAGHSLESDMKALKIVHSSVIDTSVLFPHKMGLPHKRALRALASEY  1075
             VQANILHLCNS+TIL GHSLESDMKALKI+H +VIDTSVLFPHKMGLPHKRAL+ALAS++
Sbjct  829    VQANILHLCNSKTILIGHSLESDMKALKIIHGTVIDTSVLFPHKMGLPHKRALKALASDF  888

Query  1076  LKKIIQNDVSGHDSAEDAIACMELIKWKLKEECKVR  1111
             LKKIIQN VSGHDSAEDAI CMEL+KWKL+EE KVR
Sbjct  889    LKKIIQNSVSGHDSAEDAITCMELVKWKLREELKVR  924
```

Graphical representation

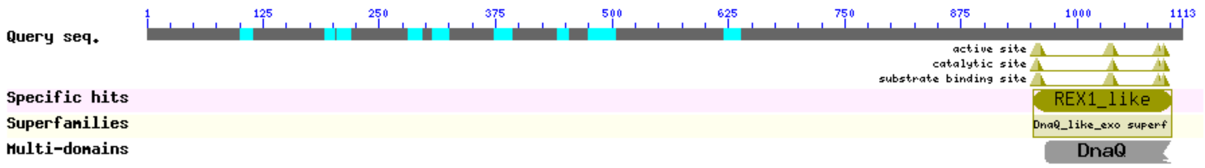

dsRNAse (Bombyx-Drosophila)

>Cb.comp34069_c0_seq2 len=1795

cDNA

TTTTTTTTCAAGCTATTGATTGAGCAGCATGACGAGTTTCTCCATTTTGCAAATATGTGGTGTTTTAAATATCATTTTCAGTTTGGGAAGCATT
GTAATTGCGACTGATTGTGAAGTATACCCATTTGAAGTCGATCCGTCGCCGCTTCTGGTTTTATCCGACACCAGTAAATTTCTATATCCCGAGC
CTTTTGAAAAGACCCTGAAATTCACATCCGGCCAATCTTTGGACATCCTCTGCCCGGGCAGAACTTTGCTATTAGGATCGACAAAAACAAATGA
CTCGTATTTAAAAGGAACTTGCGTTAAAAACGACACGTTCCTAGTAAATGGAAGAGAAATCCTTTGGAAAGAAATAGCGTGCAACAACTATCCG
TCGAAGAGTGCTAGACGATCGGGTCTGTCGTGTGACAATGGTGGCACAGACATAGAAATCGGTTTCGAAGTGGGAGATGGTCGTTTTTTGAAGT
CTCTAAGCGTTTGTTTTAACGAAACCAGTCAACAAGCTTTGTACTCTTTTCACAATATGACCGCGGCTATTAACCAAAGGGTGCTGAATACACC
AAGACCATCGTGGGTGCAAGGTTCGGGATTCTATAGTATCGGTACTGTCACGAATTATTACGTGAGGAACAGTCAGAGACGTACCATCAACACC
TTACTAGGACTAGATGTCAACTCCACGAAATATATCGAAGACAATACCAACTACTACTTAGCTCGAGGTCATATGAGCGCTCGAAGTGACCATT
ATTATGCCGCCCAGCAAAACGCTACATTTTACATGATGAATATAGCACCACAGTGGCAAACGTTCAATGGTCTCAACTGGAACCAAGTCGAGAT
AGATATCAGAGATTACGCTGAAGATCGCGGGGTTAATCTGCAAGTGTGGACCGGAGTCTACGGAGTAACCACTTTGCCTCACGAACAAACTGGT
CTTCCAGTAGAGTTGTATTTATATGTGGATGAAAATAACAATAAAGCTCTACCCGTACCAGAAGTATATTGGAAAGTGGCATACAATCCAATCA
CAGAAAGGGGACTTGTGATGATTGGAATAAACAATCCGTATCTTACGAATTATACAAAAATTTGTGATGACGTTTCGGACAGAATAACGTGGCT
ACATTGGAAGAAAGACGATCCAGCCAGAGGTCTTTCGTATGCGTGCGCTGTTGATGTTTTCCGACGTGTTGTTACGTCTATGCCCGACGTGCCA
ATAAGGGGGTTGCTATTATAAATACGTAAAGTTCAAGTGTAAGTTTGAGTTGTAAATTAAACTGCCAATTCTTCCCGTAGATTCTTCTAACTAT
CCAACGGCTCATGATATCTGTCTGGGAAGCAAAATATTTTTGTTTGTGTAAGAATTGCTTACATAATTATTTGTGTGCAAAAAGTGTATATTTG
TTGCAAGGGGCATCTTTCGGGAGCTGCAATCGTGCAACAAATATTCTTTTCCACATCCTATTACCTTTATCCCAGAGTTGTATAAGGTTCGGAT
TGTTGGAATGGAAAAAAACCTTCGAAAAAAATATATTTTTGGTGATGGACTTTTGACATTGTAAGACAAAACTTTTTTGGGATTTATTTCCAAA
TTATCTCTAGAATACACAACATAACAACCACAACTTATATAATGTTTTGGGCGCACCTGTCGCTAACCAAGTTTGCTTGCGAACAGAGATCTAT
ATATCATCTGCTGATGATACCAATTTAGTTACTCAGTTTACATCATCTGATGATGCTAAATTGGTTAGGGTCCAGAACGTTATACCGTGACCGG
CAAAGAGTT

Protein RF 2: 29->1243 (404AA)

MTSFSILQICGVLNIIFSLGSIVIATDCEVYPFEVDPSPLLVLSDTSKFLYPEPFEKTLKFTSGQSLDILCPGRTLLLGSTKTNDSYLKGTCVK
NDTFLVNGREILWKEIACNNYPSKSARRSGLSCDNGGTDIEIGFEVGDGRFLKSLSVCFNETSQQALYSFHNMTAAINQRVLNTPRPSWVQGSG
FYSIGTVTNYYVRNSQRRTINTLLGLDVNSTKYIEDNTNYYLARGHMSARSDHYYAAQQNATFYMMNIAPQWQTFNGLNWNQVEIDIRDYAEDR
GVNLQVWTGVYGVTTLPHEQTGLPVELYLYVDENNNKALPVPEVYWKVAYNPITERGLVMIGINNPYLTNYTKICDDVSDRITWLHWKKDDPAR
GLSYACAVDVFRRVVTSMPDVPIRGLLL

Comparison with *Tribolium* PREDICTED: similar to CG6839 CG6839-PA (400AA)

```
Query  18   SLGSIVI--ATDCEVYPFEVDPSPLLVLSDTSKFLYPEPFEKTLKFTSGQSLDILCPGRT  75
            SLG   I  A DC +    +DP P++V   T  FLY  P   ++   SG+++ I CPG
Sbjct  13   SLGDFFILRAPDCNIQISNLDPEPIVV-DGTYTFLYAAPDASSVLVKSGETIIISCPGGE  71

Query  76   LLLGSTKTNDSYLKGTCVKNDTFLVNGREILWKEIACNNYPSKSARRSGLSCDNGGTDIE  135
            + +GST  N S +  TCV N  F V    I + +I C+  P  +AR +G  C+  G +IE
Sbjct  72   ITVGSTSFN-STVSATCVSNSDFSVGSATINFNQIVCSWNPFHTARYTGKLCEKQGKEIE  130

Query  136  IGFEVGDGRFLKSLSVCFNETSQQALYSFHNMTAAINQRVLNTPRPSWVQGSGFYSIGT-  194
            +GF + +  F + +++CF+  +   LYS + +T +I        RP +++   FY++
Sbjct  131  VGFVINE-NFAREITICFDNANLNTLYSSYEITKSIGHHESGVSRPFFIEDD-FYNLDVK  188

Query  195  VTNYYVRNSQRRTINTLLGLDVNSTKYIEDNTNYYLARGHMSARSDHYYAAQQNATFYMM  254
            V + YVR  QR TIN+LLGL   STKYI+D  ++YLARGH +A++D  YA QQ ATF+ +
```

```
Sbjct  189   VNSLYVRGGQRTTINSLLGLPAGSTKYIQDGNDFYLARGHFAAKADFVYAPQQTATFHYV  248

Query  255   NIAPQWQTFNGLNWNQVEIDIRDYAEDRGVNLQVWTGVYGVTTLPHEQTGLPVELYLYVD  314
             N+APQWQ+FNG NWNQVE D+RDYAE  G++L+++TG YGVTTLPHE+TG    LYLY+
Sbjct  249   NVAPQWQSFNGYNWNQVESDVRDYAEKNGIDLKMYTGTYGVTTLPHEETGEETPLYLYIG  308

Query  315   ENNNKALPVPEVYWKVAYNPITERGLVMIGINNPYLTNYTK--ICDDVSDRITWLHWKKD  372
              N  + + VPE+YWKVAYNP T+ G+ ++GINNPY  +  K  IC+DVS +I WLHW
Sbjct  309   SNGIQGIAVPELYWKVAYNPETQLGVALLGINNPYQKDINKSIICEDVSAKINWLHWNAS  368

Query  373   DPARGLSYACAVDVFRRVVTSMPDVPIRGLLL  404
             D   G SYAC VD FR+ VT +PD  ++GLLL
Sbjct  369   DTKAGYSYACEVDAFRKRVTYLPDFVVKGLLL  400
```

Graphical representation

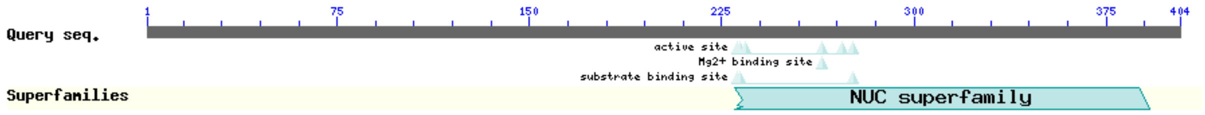

>Cb.comp37521_c0_seq1 len=1966

cDNA

```
CTCCTATCTTATATTAAAACATGCAGAATTATTAAATGCCTTCAAAATATTATTAAATATGCATACTCTTAATTCTACATAAATTTTTAAAACT
TTTTATTTTGATGTTAAAATACCTGATGATTTTGTACGTAAAAAAGAGAAAGAAGAAAAAAATCATCAACGCTCTTTTTCACATTATAGTTGGAA
AAAATCGTCGAAAAAATGTTTACATGATGTTTTTTCCATGTAAATACCTCAGAAATCACCACAAAATTTTTACCAATATTTAGGAAAATCCGTCT
TTGGGGTCTCGATACAAAATCCCCAGGTATCATATTTTAAAATCAATAAACTAACCAAAACTAAGGAAAATATTAGGCGAAAATAAACACGTTC
TTATGCAGCAGTCGCCCTTAAAACTCCCGCCACCACCAGGTTCGGCGCAGACACCCCGACGGCCTCCTTGAAGGAAGAAACTTCGCAGCAATAA
AGGTACCCTTTTCTCGGATTGGCCCAGTTGGGGTTGGCCCATCCCGCAGGAGTGCATATGTCGGGACACAACAGATCTTCGGCTTTGGGACCGT
CCTTCAGGAACGGGTCGTTGATTACGACAAAAGCTATCCCGAAATTGCTTCCGGAGTTATAAACGATCTTCCAGAAATATTTCGGAACGGGTAG
TATCCCGTTTTCCGCCAAATAAATCGGACTCGGATTGTCGTTGACGTCCGGGTAAGTCAAAAGCCCGTGAGTGCCGGTGAAAATGTCCACGGTA
CCGTTTTTACGTACCGCCATCTCGAGACTTTTCCAGTTGGCATTGTTCACGATCTGCCACTTGGGGGCGACGTTCAGATAGAAGTAGGTGGTGT
ATTGACTGATGGCGAGTAAGAAATCAGCATGAGGTGCTAGGTGCCCTTTTACCAAGAACGAGTTCGGGCCGATGTAACTATCTGCGACGGTGGT
AGAGTTGAGGGCTGTCGAGAACGCCCTCTTCTGAGAGGCAATGGGATAGGCGGAAGCTGCATCGATCTGCAAACCTTCCTCGCTGAACTCGGGC
CTATAGCTTGACTTGGACGCGTATTCGATTTCTGAACCAACCAGTTGGTGCTTTGCGTAGAAAGTGTTGCCGCTGCTCGAGTCGTGGCACACGT
CAATTAAACCTATCCACTGACCTGGCGGTAGCTTGTGACCGATGCCATACAGGGTACCCCTTCCGCCGGCGCACGTCCCAAGCCCTGCGTTCAC
CTGGCCCTTGACTATAGTCGAGCATCGGAAGTTGAACAATGGCCGGTCGATGCCGTTCAGCTTAAGGATGTTTGATTTCTCACATCTCGGCTGG
TGGATCTTCTCGGTCCAATCTTACCAACGATAATCTGTTTCTCGCTCCCGGACACACCATATTCAAAGCCTGACCATGACTCAAGTGTATTCGT
CCTCTGTGGGGGACGAATACCCTATACGGAGGTTCCGCACTCAACGGTACCGGCAAGTACTTTTCGACAGTTGGATCAGTGACGTGCAACGAGC
AGTATTCATCTGTGGCCCTGTTCCTCCCAGGTTCTGTATCGATGTGCGGATAAACGTGCGCGTACCACTGGGATGTGCTCAATGTTTTCTGCCT
CTTCTTTTTCGCGCCATCGGTGTCTGTCGCGGCCGTTGTACTGGCCGTTGACTGTGGTGACCTGTATGTATTGTAATGATAAGAAACCTTTCGT
TGAAATAAAAGGCCCGACTGTAGAACACTAGTTGCCAATACTGCCACACCTGCACCCGATATCCCACAAGGGGCTTTAACTGCTTTAACCAGTG
GGTCGAAATGCATAAATCTCTTATTCCTCTTGCCTATTTCGCCTTCGGTACCATCGGAGCGCCTCTGTTCCAAATTCTGTTCGTAAACTGGATC
GACACTACGAGCTAACGATATCGATAACGGACAACATACGCAAGCGAGCAATAAAATGTGAAGTTTGGTGGCCATCTTGACTATAG
```

Protein RF : -1321->-392 (AA)

Comparison with *Tribolium* PREDICTED: similar to deoxyribonuclease I (396AA)

```
Query  1321  IHQPRCEKSNILKLNGIDRPLFNFRCSTIVKGQVNAGLGTCAGGRGTLYGIGHKLPPGQW  1142
             I Q +C +  IL++   D   + +C  I++G V      C   +G +Y IG+++     +
Sbjct  81    ITQAQCVQGGILRVANQDLTFRDLQCKKIIRGTVAKTTKKCGENKGRIYKIGYQISSRNF  140

Query  1141  IGLIDVCHDSSSGNTFYAKHQLVGSEIEYASKSSYRPEFSEEGLQIDAASAYPIASQKRA  962
             + LI+VC+D +SG T Y +H L G +I+YASKS+YRP FS E   + A+ AY    QK
Sbjct  141   LTLIEVCYDPNSGTTLYTEHALHGQDIKYASKSNYRPAFSPEASAVAASVAYKQTFQKST  200

Query  961   FSTALNSTTVADSYIGPNSFLVKGHLAPHADFLLAISQYTTYFYLNVAPKWQIVNNANWK  782
             F+  + S   A  YI  NSFL +GHL+P ADFL A +QYT+Y+Y+N AP+WQ +N  NWK
Sbjct  201   FNKLMKSALKAQEYINENSFLSRGHLSPDADFLYAATQYTSYYYINAAPQWQTINAGNWK  260

Query  781   SLEMAVRK-----NGTVDIFTGTHGLLTYPDVNDNPSPIYLAENGILPVPKYFWKIVYNS  617
              +E+ VRK       T+ + TGT+G+LT PDVNDN   +YL     LPVPK+FWKI+Y
Sbjct  261   KIELLVRKLADNLQETLTVITGTYGVLTLPDVNDNEVDVYLVSGSKLPVPKFFWKIIYAK  320

Query  616   GSNFGIAFVVINDPFLKDGPKAEDLLCPDICTPAGWANPNWANPRKGYLYCCEVSSFKEA  437
              S   +  V +N+PF+K+  K  D LC ++C+  GW + +W+N  +G++YCC+   F +
Sbjct  321   HSRQAVVLVSLNNPFVKEIGKG-DFLCSNVCSKVGWGSGSWSNYERGFVYCCDYKQFVDK  379
```

```
Query  436   VGVSAPNLVVAGVLR  392
             V  +AP L V GVL+
Sbjct  380   VE-TAPKLSVVGVLQ  393
```

Graphical representation

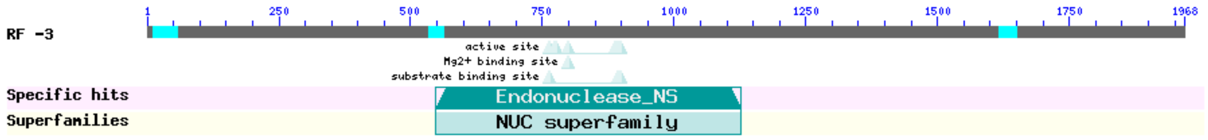

Exosome

>Cb.comp37515_c0_seq1 len=2944

cDNA

GTCAATAACAAAGAACATAATATTAAAGCATTTATTTTAATTTTGAATATTTGTATATATAAATATTAGAATATACATTAATTGCAACATAACT
TTGTAAATATTAATTTCTCTTTTTAGAATTATTATTTCCAAAATTCCTCTTCTTTCCTTTAGATTTAAATTTACTTTTAAATGCTTGTGGTTTC
TCGACTCCTCTGGCACCCCCTTCAAATTGCCTGAAATCGACCATAGAATAATCATAGGCATTAAACTGTTGTTCATTTTGATTAACATTTCGTG
TTGCACCTTTTTTACGTTTTTGCCAGAATTGCTTATTTTTCTGAGACTTCTTTGAATTTTCACCACTATTACGATTAGTTACTTGTTCAATGGT
GGACGAAGTCTCACCAGTTGAAGTTTCACCATCTTGTGCCACTTGCTTATTTGTGACATGCTTCTGTTTTCTTCTCATTTTTCTTTTGTTGTTG
TTTGCTAAATAACTCTGTGATTTACGCTTTTGTCCCTGTGACTTGACTGTTTTCTGTGTGCTTGAATTAATTCCAATATTTCCTGAAGCCTTT
CTTCAAGTGCACTTGAATCTAAATGACATGAGTTTGTTTCTGAGTTGGAATTGACGTCTGAATTCACATTATCTATCTCAGTCCGTTTTCTCTT
CTTGTCATTTTCATTTGTTGGGACTTTTTCAAGAGACTTATTAGAAAGTTCCAAAAAATGTTTTCTTATATCGTCAATTCTTTCTGCATCTGTT
CTAGTATCATTTTCATTTAGTTCATGTTGCTCATTAATAGATTGTTTCTTATTTTTGGCATTTTCCTCAATAATTTTCTGTTCCTTTGCCTGAA
TAAAAGGCCTAATCATTTTATACCTGGTGTAGGGGCTTAAAAAATTTAATACTCCTTTGAATCTTCTCGTCAGAGATTTGTTAGTACCATCTGA
ATTTTCCAGTGGATTAAATAGAGAGTAAACTGGAGACTCTTGTTCAATTTGTGGGGCATCAATGTTGCTGAGATCAAGTTTGTCCCCTCCAAAA
AGGGTAGGCAAATTATCACAAACTTCTCCTCTATTGGTTAAGTCATGAGGACAATGTATCGGATTATTTATATTGATTTTAGACAATTTTGTAG
TTATCCCTTTAGATCGAGTATCTTCCTTCAAAATAGGTAACTCAAATGGTTGCTCCAAAGCTTTTAACATAATTTTATGTAACTTGAGTAGATG
GGCTTTTACCAGTGGTGGCGTAGGATTGCAACAAGCGAGAATTCCTTGCATTTCTCGAGGCAATTGCTCAGACATTTCCAGCAACATATTGTTT
GGTAAAACATAGCCTACACTTTCGTCCTCTTCACGAGAGACCTCGTCTCTCCACTTGTACAATTCTTTCAGGGCAAACAACTGCCTGTTGTGAA
ACATTCTGCTGCAACGCCGATAAAGATTTAAATAACTCTCATCATTAAAAACAGGTTTATAATATCGAGTTTTGCACAAATCAGTACTCATTTG
TATAACAGACTTCAATAGGTTATCTTGGCCTTTGGCTCTATCTAAGAGCTCATTTTTCATTTTTTGATATATATAAATCAGGTAATGAGTATCT
TCTCTGGCATAAGATTTCAATTCAACAGGTAAAGGACGTAATCTCCAATCAGCCAACTGAAATTGTTTGTTTGGTGCAAAATTACAAAACTTTT
GCATTAAATAAGCTAAAGAGAGTCCCGGATACTCAAGTTGTTTAGCAGCAAAATAAGTGTCAAACATATTAACAACATAAAGGGATAAATCTCT
CTGCAACCACTGAATGTCAAAATGTGCTCCATGAAATATTTTTGTAATCTTTGGATTAGTAAATACCTCATTTAAAACATGTAACTTGTCTCTT
AAGATCAAGGTGTCAATTAAATAATCCTTTTCCAAAGTAGATATCTGAAGAAGGCAAGTTATTCCCATAAAGGATCTGTATGAGTGATGTTCTA
AATCAATGCCAATTTCCTTAAAATTTTGAAGATCATCAATTAAATCTTTTAATTGTTCCTCTTGTGTAATTTCTACCAATGGTGTGTCTTTTAA
TAATTTTGGTGTGACTATGGTAGTTCTTCTTAAGTGATCTTCTGAAGGTGAAAACCTCTCTAGTTCATACTCATAGGGATGTGAATATTCCTGT
CCATTCACAGTTTCTTCCAAAAATATTGCAAGTGGTTTAATTGAATTTGGTTTTTCCTTGATTCGCGGTTCCCATGGTCTTTCTCTGCTATTAT
CGATTTTATCTTTAAACATTTTTTGAGGCCTAATTATATTTTCTGCGGTAATTAATCGAATAGTCTGGGTAGGACCTGGAGATATTGTATAGTC
TGAAGGAATAGTAGCAGATGAGGAGACCACCGGTCTACTGATTCGATTCCATGATCCATTGATTGGCAACTGTACACTGACAGTTTGAAATACT
GTAGGCTCCACATTAATTTTCTTTATTCCATTTTGCTCATCAATGTTATCTGAGACCCTCTCAAGGATAACATCACAAGCTTCTATTAATAATT
CAGATTTCTTGTCCAAAATATTATTACACATGCTTCCTTTAACATCGTTTTTCCTTAAAACTAAATTCATAGAATTCAAGAGATTATCGCTGGC
TTCTTGTAAAGTAATTTTGTTAGAGTCCACCATATAAATCTTAAATTTCTTACTACTCGGTAATGCATTTGACAATTTTAAGGTTTCTTTAACA
ATTTCGTATCCGTTCTTAAGAAATTCTTCCACGGATTTATTTGTTCCGATTTGGTTAATATCATCTTTGTTTTCAATGTTTAATGATTTATTGT
TGGTCAACATTTTAACTCTTTTTCACACTTAACCTTAACAAAAAAAATGTGAATGAAGTGTAGTGTTTCGCATTGCGCATGATGTTCTGGTTTT
TTGTTAGAAGAAGACGAAAAGAAGAACCAA

Protein RF -1: -2830->-104 (908AA)

MLTNNKSLNIENKDDINQIGTNKSVEEFLKNGYEIVKETLKLSNALPSSKKFKIYMVDSNKITLQEASDNLLNSMNLVLRKNDVKGSMCNNILD
KKSELLIEACDVILERVSDNIDEQNGIKKINVEPTVFQTVSVQLPINGSWNRISRPVVSSSATIPSDYTISPGPTQTIRLITAENIIRPQKMFK
DKIDNSRERPWEPRIKEKPNSIKPLAIFLEETVNGQEYSHPYEYELERFSPSEDHLRRTTIVTPKLLKDTPLVEITQEEQLKDLIDDLQNFKEI
GIDLEHHSYRSFMGITCLLQISTLEKDYLIDTLILRDKLHVLNEVFTNPKITKIFHGAHFDIQWLQRDLSLYVVNMFDTYFAAKQLEYPGLSLA

```
YLMQKFCNFAPNKQFQLADWRLRPLPVELKSYAREDTHYLIYIYQKMKNELLDRAKGQDNLLKSVIQMSTDLCKTRYYKPVFNDESYLNLYRRC
SRMFHNRQLFALKELYKWRDEVSREEDESVGYVLPNNMLLEMSEQLPREMQGILACCNPTPPLVKAHLLKLHKIMLKALEQPFELPILKEDTRS
KGITTKLSKININNPIHCPHDLTNRGEVCDNLPTLFGGDKLDLSNIDAPQIEQESPVYSLFNPLENSDGTNKSLTRRFKGVLNFLSPYTRYKMI
RPFIQAKEQKIIEENAKNKKQSINEQHELNENDTRTDAERIDDIRKHFLELSNKSLEKVPTNENDKKRKRTEIDNVNSDVNSNSETNSCHLDSS
ALEERLQENIGINSSTQKTVKSQGQKRKSQSYLANNNKRKMRRKQKHVTNKQVAQDGETSTGETSSTIEQVTNRNSGENSKKSQKNKQFWQKRK
KGATRNVNQNEQQFNAYDYSMVDFRQFEGGARGVEKPQAFKSKFKSKGKKRNFGNNNSKKRN
```

Comparison with *Tribolium* PREDICTED: similar to Rrp6 CG7292-PB (947AA)

```
Query  24   SVEEFLKNGYEIVKETLKLSNALPSSKKFKIYMV-DSNKITLQEASDNLLNSMNLVLRKN  82
            S+E+F K+G++++ E +K SNALPS + +  Y + DS K  ++   +++L  MN V+R N
Sbjct  24   SMEQFTKDGFKVLMEAIKHSNALPSGRDWDFYNISDSFKEIMKVEGNHVLRLMNQVMRCN  83

Query  83   DVKGSMCNNILDKKSELLIEACDVILERVSDNIDEQNGIKKINVEPTVFQTVSVQLPING  142
            D+   ++ N +LD+K EL+IEA D+ILE+V++NIDE NGI+K  V P V QTVS QLP+NG
Sbjct  84   DLDSNLRNRVLDEKIELVIEANDIILEKVANNIDEMNGIRKTVVAPVVLQTVSAQLPVNG  143

Query  143  SWNRISRPVVSSSATIPSDYTISPGPTQTIRLITAENIIRPQKMFKDKIDNSRERPWEPR  202
            SWNR +   V+ S+ +P     S G    I+LITA+NIIRPQK FKD+IDN  + PW PR
Sbjct  144  SWNRQTAATVTVSSVVPE----SSG-QNCIKLITAKNIIRPQKFFKDQIDNRNKTPWSPR  198

Query  203  IKEKPNSIKPLAIFLEETVNGQEYSHPYEYELERFSPSEDHLRRTTIVTPKLLKDTPLVE  262
            I EKPNS+KPLAIFLEE  + QEYSHPYE+EL+RF P+   L     V PK L DTPL+E
Sbjct  199  ITEKPNSLKPLAIFLEEYEDRQEYSHPYEFELDRFQPTPSQLIDEKSVPPKSLSDTPLIE  258

Query  263  ITQEEQLKDLIDDLQNFKEIGIDLEHHSYRSFMGITCLLQISTLEKDYLIDTLILRDKLH  322
            I + EQL +L++ L++ KE  +D+EHHSYRSFMGITCL+QIST +KDYLID L LRDKL
Sbjct  259  IDKAEQLDELVETLRHCKEFSVDVEHHSYRSFMGITCLIQISTEDKDYLIDALALRDKLS  318

Query  323  VLNEVFTNPKITKIFHGAHFDIQWLQRDLSLYVVNMFDTYFAAKQLEYPGLSLAYLMQKF  382
            +LNEVFT   I KIFHGA  DI+WLQRDLSLYVVNMFDT+ AAK L+YP LSLA+LM+KF
Sbjct  319  ILNEVFTKNTIVKIFHGADKDIEWLQRDLSLYVVNMFDTHQAAKALQYPALSLAFLMKKF  378

Query  383  CNFAPNKQFQLADWRLRPLPVELKSYAREDTHYLIYIYQKMKNELLDRAKGQDNLLKSVI  442
            CN  PNKQFQLADWR+RPLP ELKSYAREDTHYLIYIY+ MK ELL +    D LL+SVI
Sbjct  379  CNVTPNKQFQLADWRIRPLPDELKSYAREDTHYLIYIYKMMKRELLHKTNKCDKLLRSVI  438

Query  443  QMSTDLCKTRYYKPVFNDESYLNLYRRCSRMFHNRQLFALKE---LYKWRDEVSREEDES  499
            + ST++CK RY+KP+ +++S+L LYR+C +MF NRQ++ALKE                 S
Sbjct  439  ERSTEVCKKRYFKPILHEDSHLELYRKCKKMFDNRQMYALKEXXXXXXXXXXXXXXXXXS  498

Query  500  VGYVLPNNMLLEMSEQLPREMQGILACCNPTPPLVKAHLLKLHKIMLKALEQPFELPILK  559
              YVLPN+MLL++SE LPREMQGILACCNP PPLV++HLL+LH+I+LKA EQP E  ILK
Sbjct  499  CSYVLPNHMLLQISELLPREMQGILACCNPIPPLVRSHLLELHQIILKAREQPLEKAILK  558

Query  560  EDTRSKGITTKLSKININNPIHCPHDLTNRGEVCDNLPTLFGGDKLDLSNIDAPQIE---  616
            E T  +G+  ++SK+N+++ +HCPHDLT   E  D+LPTL   ++    N    Q++
Sbjct  559  E-TSGRGVLKEMSKVNMDSVLHCPHDLTKTNEFRDDLPTLLQNEQYKAENKRVAQVDLAI  617

Query  617  QESPVYSLFNPLENSDGTNKSLTRRFKGVLNFLSPYTRYKMIRPFIQAKEQKIIEENAKN  676
            ++   YS+FN   +  G       +      +LSPY RYK+++PF+ A+     +E AK
Sbjct  618  EKPSSYSIFN---SDQGFKPGEAFKKLHTSAYLSPYERYKLVKPFVVAE-----DEAAKA  669

Query  677  KKQSINEQHELNENDTRTDAERIDDIRKHFLELSNKSLEKVPTNEN--------------  722
            +K          E D +TD ERI  IR HF++LS K  E++   +
Sbjct  670  QK----------EKDEKTDDERITSIRDHFVQLSKKFSEELQAQKEEEERKERELSLIEM  719

Query  723  --DKKRKRTEIDNVNSDV  738
               +KRKR EID+ + DV
Sbjct  720  GASRKRKRDEIDSESPDV  737
```

Graphical representation

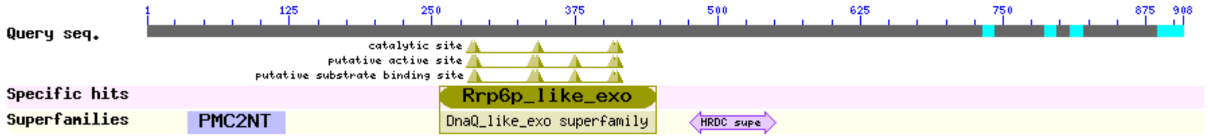

Poly(A) polymerase (Pla1 homolog Schizosaccharomyces)

>Cb.comp42000_c0_seq4 len=2741

cDNA

```
CTCGGGGAAAAATATTTCATTTATTGTACTTAATCACTTACAGGGACGACTTTACATTGACGAAAACCTCCTCGCACTAACCTAACGCAAAATT
AGTCAGAAATTCAAATAAATATTCAATATACACTTTGCCAAAACCGAACGATATCTTCCCCAACTTTTTAATGTGATTATACAAAATCAATAAT
ACAGAGCGGAAAAACGTTACATAATAAATTCGGTAAAAAACGATGACGTCGTGGCCCGACACGGATACGCAAAAGAAAAGTAATATCAAAAATA
AAAAGGAGGGGCAAAACCTGCAGGTCATAAGCATGAGCGATGCTTATAAAGCCCCCTCCTGGGTGTCGTGTTCCAAATAAAACACCAACTATGT
ACAAAAGTGCACTACTGTAAAATATTACCGCATCACGACTGTCCATTCGCATACACATTTCGAAAACGAAACGTCCCGCTACAATCCAAATAAA
GGTTCGAGAAAAAAAATTACTTAATTATAAACGTAATAAATAGATAACACGCATTCTCTCACTTAACTACACGCCTATTTAATGAAATTGCACT
TACGCGATAATATTTTACATACGCCCCGCCCCCACTGAGGAGGCGACCTCCTGGCGCGCGTCTTCGTCGCCCGCCGGGTGGTCCTCGGCCAACA
CTTATATCCTAAAGGAGACCAGCTGAAGTTCACGAGCACACCGTTTCCTGTTTGTTCGCTGTCGGCACCTGTGGTAATTTCACGGGACTGGCGG
CCGACGCCCCCGACGTATCCGCGAAACTGCTGCTCGAATCGTCATTGTTCAGCGAATTGTTCGAACTGTCGTCGAAGCTTGCGCCGTTCACTTC
GTTCGACAACCGCGTTTTCTTTGCTGAGTTATCCCCGTCGCTGGAATCGGACCGCCTCTTCCGGCTGTCTTTCTGCTCCTTGCCCTCGTTGACC
ATCATCAGGTACTTCTTCTCGCGTTTGAGGATCCCGTGCGGTAGGTAAGTGGGGAGCATTTTCCTCTTGACGTGTCGGGCTTCCAGTTTCATCG
TCTCCTTGAACATTTGTATGTGCATGGCGTGCTTGTTGACGGTTTCGGTGAATTGTTGGATGTCCTGTGTGAGATCCACGTTGAGACCCTCCAT
TTTCCGAAACTCGAGTCCGATGAACCACATGGAATATTTAAATTTCGGTTTTCGAAGGGGTTCGGGCAACATGAAGTTTTCCGGATTTATGTGC
GCCAATGTTATTGCGGTGTTTCTCTCCAAGGTCGAAATTAACAATCGTATCTTGCTCTCCACCAGACCGCTCCATTCTAACAGATCGGCCGAAG
TTTCCGCGCTGATTATCAGCACAATGAAGTGCCTGTACTTCATAAAAAATGGCGGTTGTATGAACAGTCGCTCCCAAGTCGATTTGTTTAACAT
AATTTGATCTGTAATTTTTAGACCCCTTTCAAACTCTTCCATGATGATGTTCCTGGTCGAGTTGGACACGTTAAAAGTCGAATTTTGTTGTGGA
TACGCAGGAGTAATTATCGGCATCAGGTGGTAACGATCTTGGATGTTTACCCTCGGATCCCAAACCGCGAATCCCAAACTTGCATTGGTCGGTT
GCTTGAGGAGCACAGGCTGGGGCCACTGCCATTTAGAAAATATGAGAAAAAACTTATGCACTAATGTAGCGGCAGCTGCATTCGGATACAACTG
GCAGGTACGGGCCACGAGCATTGCCCACGAAACGCCTCCGAGATACCCCAATACGTTGCTGTAAATGCCATGCCTCTTTGCCCAGAGCTTTATA
GTCCGTAATGCCAATCTAAAAGTTTCAATATTGGGAACGAGTCTAAGAATTTCGTCTGTAACCCGGCAACCGTTAAGACTGCGGACACACTTCT
GGTCAAGGTTTTTTAGCAAGTTATCATCGCGAAGATCCATAGAGTCGGGAATTTCTTTTTGCAACAGTCGGGCAAAAAGTAAATCAATTTCAAT
TCCATCAAAACTCATTTTAATAACTGGTACAAAGGCCTCTTCTACTGACCTTAGCTCTGTAACTTCAGATTGCTTCTTTAACAACTCATAAAAA
GAACTGAAAAAATCATTCCGACAAATATGCTTGGGTGCTACACATAATGCATCTATATCGGCTCCCTTGTTATGTACACCGAGTCTGTAGCTAC
CAAAAGTATACACTTTCCCTCCAACATTTTCAGCTACACTTTCAGGCATGTTACATTTTAGTGAAAGTTCTTTAATCCACTGCTTAACTAAACA
ATACAATTTTCCTAAAATGAGCATACGATGGTTTAGTTCCTGTTCGGTTTCAAAAACATCAAAGGGTATCAAAGTTTCTTCCAACTCCTTAGTT
TTGACAAGGTCAATGGGCTTAGGAGGAGCCGCAGAAATAGCGGATGTCATTCCTAAGGTAACTAAGTTGTCTTCTGATTGAGTTTGATTTTCCT
TGTTATTTTGCGTGGAGTTTGATTGAGAGGACCACATTATTATAGTGATGTAACACAAAGCAAGATATTAGGAAATCTTAAGGTCGCGAATATA
AACGCGTATAAAGCAGATAACGATATTCGTAGTACATCAATTAGTTTTCTCCCTTAATAAAAATCGGTGGCACGATAACTGTTAACTAAAGACA
TTTCAAAAATCATCAAAAAAATAGTTTATTTTGATGATTTTTTCCAATGATTATTATTTTGGAACAAAATGTCCAAACCAGCCATTTGTCACTA
CTGGTTGCAATGTTG
```

Protein RF -3: -2481->-688 (597AA)

```
MWSSQSNSTQNNKENQTQSEDNLVTLGMTSAISAAPPKPIDLVKTKELEETLIPFDVFETEQELNHRMLILGKLYCLVKQWIKELSLKCNMPES
VAENVGGKVYTFGSYRLGVHNKGADIDALCVAPKHICRNDFFSSFYELLKKQSEVTELRSVEEAFVPVIKMSFDGIEIDLLFARLLQKEIPDSM
DLRDDNLLKNLDQKCVRSLNGCRVTDEILRLVPNIETFRLALRTIKLWAKRHGIYSNVLGYLGGVSWAMLVARTCQLYPNAAAATLVHKFFLIF
SKWQWPQPVLLKQPTNASLGFAVWDPRVNIQDRYHLMPIITPAYPQQNSTFNVSNSTRNIIMEEFERGLKITDQIMLNKSTWERLFIQPPFFMK
YRHFIVLIISAETSADLLEWSGLVESKIRLLISTLERNTAITLAHINPENFMLPEPLRKPKFKYSMWFIGLEFRKMEGLNVDLTQDIQQFTETV
NKHAMHIQMFKETMKLEARHVKRKMLPTYLPHGILKREKKYLMMVNEGKEQKDSRKRRSDSSDGDNSAKKTRLSNEVNGASFDDSSNNSLNNDD
SSSSFADTSGASAASPVKLPQVPTANKQETVCS
```

Comparison with *Tribolium* hypothetical protein TcasGA2 TC003818 (565AA)

```
Query  1    MWSSQ--SNSTQNNKENQTQSEDNLV-TLGMTSAISAAPPKPIDLVKTKELEETLIPFDV  57
            MWSSQ  +N TQNNKEN TQ  D  + TLGMTSAIS APPKP DL+KT+ELEE L PF V
Sbjct  1    MWSSQPVNNGTQNNKENVTQKNDTKIPTLGMTSAISTAPPKPSDLLKTQELEEALKPFGV  60

Query  58   FETEQELNHRMLILGKLYCLVKQWIKELSLKCNMPESVAENVGGKVYTFGSYRLGVHNKG  117
            FE+EQELNHRM+ILGKLY LVKQWIK++S+  NMPESVAENVGGK+YTFGSYRLGVHN+G
Sbjct  61   FESEQELNHRMVILGKLYSLVKQWIKDVSISKNMPESVAENVGGKIYTFGSYRLGVHNRG  120

Query  118  ADIDALCVAPKHICRNDFFSSFYELLKKQSEVTELRSVEEAFVPVIKMSFDGIEIDLLFA  177
            ADIDALCVAP+HI RNDFF SFYELLKKQ EVT+LR+VEEAFVPVIKM+FDGIEID+LFA
Sbjct  121  ADIDALCVAPRHISRNDFFGSFYELLKKQPEVTDLRAVEEAFVPVIKMNFDGIEIDMLFA  180

Query  178  RLLQKEIPDSMDLRDDNLLKNLDQKCVRSLNGCRVTDEILRLVPNIETFRLALRTIKLWA  237
            RLL KEIPDSMDLRDD LLKNLDQKCVRSLNGCRVTDEILRLVPN++ FRLALR IKLWA
Sbjct  181  RLLLKEIPDSMDLRDDLLLKNLDQKCVRSLNGCRVTDEILRLVPNVDNFRLALRAIKLWA  240

Query  238  KRHGIYSNVLGYLGGVSWAMLVARTCQLYPNAAAATLVHKFFLIFSKWQWPQPVLLKQPT  297
            KRHGIYSN LGYLGGVSWAMLVARTCQLYPNAA ATLVHKFFL+FS+W+WPQPVLLKQP+
Sbjct  241  KRHGIYSNALGYLGGVSWAMLVARTCQLYPNAAPATLVHKFFLVFSQWKWPQPVLLKQPS  300
```

```
Query  298  NASLGFAVWDPRVNIQDRYHLMPIITPAYPQQNSTFNVSNSTRNIIMEEFERGLKITDQI  357
            N +LGFAVWDPRVNIQDRYHLMPIITPAYPQQNSTFNVS STR IIMEEF+ GL++TD I
Sbjct  301  NVNLGFAVWDPRVNIQDRYHLMPIITPAYPQQNSTFNVSGSTRQIIMEEFKLGLQLTDDI  360

Query  358  MLNKSTWERLFIQPPFFMKYRHFIVLIISAETSADLLEWSGLVESKIRLLISTLERNTAI  417
            ML+K TW++LF  P FFMKY+HFIVL++SAE+  D LEW GLVESK RLLI TLERN  I
Sbjct  361  MLSKQTWDKLFEPPLFFMKYKHFIVLLVSAESPEDHLEWCGLVESKFRLLIGTLERNQHI  420

Query  418  TLAHINPENFMLPEPLRKPKFKYSMWFIGLEFRKMEGLNVDLTQDIQQFTETVNKHAMHI  477
            TLAHINPE+F L E  R+      SMWFIGLEF K E LNV+LT DIQQFTETV  HA++I
Sbjct  421  TLAHINPESFSLLESQRESNTHCSMWFIGLEFAKSENLNVNLTFDIQQFTETVQNHALNI  480

Query  478  QMFKETMKLEARHVKRKMLPTYLPHGILKREKKYLMMVNEGKEQKDSRKRRSD--SSDGD  535
             M KE MKLEARHVKRK L  YL   +LKRE+K   + V       DS+KR SD  +SD D
Sbjct  481  SMLKEGMKLEARHVKRKQLYQYLSPSLLKRERKTSITVKSQSNGTDSKKRLSDPGNSDSD  540

Query  536  NSAKKTRLSNEVNGA  550
            N  KK RLS E++
Sbjct  541  NPNKKIRLSEEMHST  555
```

Graphical representation

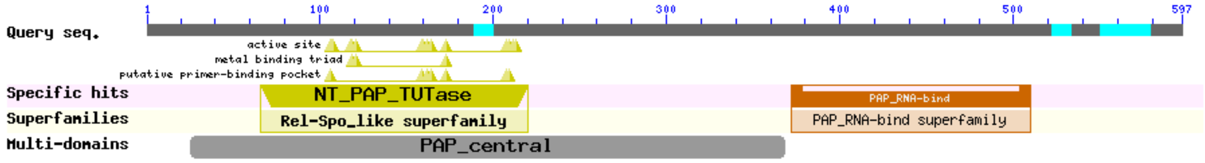

Auxiliary factors RISC in *Cylas brunneus*

Translin

>Cb.comp39483_c0_seq2 len=2184

cDNA

GGTAATACATTTTCTATGGTAATGAGCATGTGGCCTCCTGGCGGAGCCCATGTGATATTAATGTAATGTATTCTTCTAAAACCCGTTAAAGGCA
AAATTCCATTCCAACCTACAAATTGTTGTTGTGGGGCTTATTCTTTTCGATATCTCCCTATCATGATTTTTCTGTTTGTTCTGACCAGGTATCA
GTAAACGAGGCCATTCATATTTTTTTGATTTGCAAGCTCATTAACAATAAATAAATCAAAGAATAAATATAGAAATATCCGAAAATATTTTGAG
ACAATTTTGCAAAATGACTCAACCAAATAAAGTTATTGCAGATATATTTGCTCCATTTCAGGATTATATAAACGCCGAACAAGATGTTCGAGAG
GAAATTCGAACTATATTAAAGAGCATTGAGAAATTTCTCAGAGAAATACACACATCGTTGCAAATAATTCACTGCGAATTAAACTGTGATCAAG
TACATGCAGCTTGCCTGAAAGCAAGACAAATTTTTGATGAAGTTAGTAAAGAGTTCGATAATCTGGATAAAATCATTCCAAGTGGACAATATTA
CAGATATAATGACCATTGGAGATATGCCACCCAACGACTGTGTTTTTTGGCTGCATTAGTTGTATTTTTAGAACAAGGAATTCTTATTGACAAA
GTTACTGTAGCAAAGATTCTCGGAGTCCATGAAAAACAACACATCCATTTAGATCTGGAGGACTACCTGATGGGCATGCTAAACTTGGCAACAG
AGTTGGCTAGATTTGCTGTAAACTCAGTTACATATGGAGATTACAGTAGACCACTACAAATATCCAGGTTTGTTGCTCAATTAAATGCTGGTTT
TCGACTATTAAATTTGAAAAATGATTCACTCCGTAAAAGATTTGATGCCTTAAAGTATGATGTTAAGAAGATTGAAGAAGTAGTGTATGATTTA
TCAATAAGAGGTCTTGTACAGACTACCGATACAGGCAGTACCCAAAACGATAATCAGCGGGTCGCACAATAGCTTTCTACAAAATTAGACATTT
CTTTTGTAATGCTAAACATGTATCTACCTATAATGATGTAATAAAACTGGTAAACATTATCTTTCTGTTAAAAAGGTCTAGATATTATACTACT
GCTTTTATTTGCTGTATATGGAGCATTTCCTATGTAATACTTTAGAAACTAATAGCAGTTCACACTTGCAAGATGATGTTCATTCTTGGTATAA
GATCAAATTATCAAACTAGATGTCAAATTGAAGAGATGAGAAAGAATGAGAAAACAAGTGCTTTTCGAACCAATAACATCTTGGCTTTTAACTT
TTCCGCCGACAGCTGGTGAATTCGACAATATTTTGTTCAAAAGGTGCCATTAAATTTTCATAGAAAAAACGTATTATGTCTGTGGAGATAGCTG
TTTGAAGAATCGATATAAGCTAAGAGTACCTTTTTACATTCAAAATCTTGAGATATATTTTATAATCAAAATTTGGGGAAAAAAACACCCATTA
TGTTTATCCTTAACCCGTCTAGTCTAGTACACCCCGATAAAATCTTTTAAATTCTGATTCAGTATCAAAAGTTTATGAGATGTATATGAAGGTG
TTTGGAAAAACCGTCTTCAGAGTGTCCCATTTAAAGGGGTGGGGAAGCATTTTCCGATAAATGTTTATATGAACTTTTTTTCATGTATATCAGG
CATTACCCCAGACTTTAAAAGCGGGAGGGTGTTTTTGTTAACTAAAGCCGCATCAGGTTGCAGTGATATCCTACGCCAATGCTCAATCCCATTT
TTGTAAGTATTTCTTGTACTGTGTAATGTGGATAGTTGCGTCTAGTATTTATTAGGTTTAAAGTTTTTTATGCCTTGACTGCGGACTGCTTTTG
ACATTTTCTGTTACCTATATTTTTAATAATGAAGTTGAGAAAACTAAATATATTATTTCAGAATAAATTTTATGTTGAACCTGCACTAGAAAAG
ATTTTTTTATTATTCTTATAAATTTCATCTACTTCATTATTTTGTTTAGTATCTTGGAAACGTTTTACCATTTGTATCCAAAAGTGTTTATAAT
TGTCTGTAGATATCTAACAGGAGCATTTAGTCCCGGATCACAAAAATTTCACGCCGGCCCCGAAAGGTTAACCCCTTGGGTATGGATTTATCTG
AGAAAATATTTGTATCGCAAAT

Protein RF 2: 298->1012 (238AA)

MTQPNKVIADIFAPFQDYINAEQDVREEIRTILKSIEKFLREIHTSLQIIHCELNCDQVHAACLKARQIFDEVSKEFDNLDKIIPSGQYYRYND
HWRYATQRLCFLAALVVFLEQGILIDKVTVAKILGVHEKQHIHLDLEDYLMGMLNLATELARFAVNSVTYGDYSRPLQISRFVAQLNAGFRLLN
LKNDSLRKRFDALKYDVKKIEEVVYDLSIRGLVQTTDTGSTQNDNQRVAQ

Comparison with *Tribolium* (AA)

```
Query  5    NKVIADIFAPFQDYINAEQDVREEIRTILKSIEKFLREIHTSLQIIHCELNCDQVHAACL  64
            + ++ +IF PFQ+ IN EQDVREEIR I+K IEK LREI T+LQIIH   N ++   AC
Sbjct  4    DNILENIFTPFQECINNEQDVREEIRNIMKDIEKPLREIVTTLQIIHRTHNGEE--TACF  61

Query  65   KARQIFDEVSKEFDNLDKIIPSGQYYRYNDHWRYATQRLCFLAALVVFLEQGILIDKVTV  124
             AR++F+ V   ++ LD ++P+GQYYRYNDHWR+ATQRLCFLAAL++FLE+G L+DK T
Sbjct  62   AARELFESVRAGYEKLDGVVPAGQYYRYNDHWRFATQRLCFLAALIIFLEKGFLVDKETT  121

Query  125  AKILGVHEKQHIHLDLEDYLMGMLNLATELARFAVNSVTYGDYSRPLQISRFVAQLNAGF  184
            A+ILG+HEK  +HLDLEDYLMG+LNLATEL+RFAVNSVTYGDY+RPLQIS+FVA+LNAGF
Sbjct  122  AQILGLHEKSRLHLDLEDYLMGLLNLATELSRFAVNSVTYGDYNRPLQISKFVAELNAGF  181

Query  185  RLLNLKNDSLRKRFDALKYDVKKIEEVVYDLSIRGLV  221
            RLLNLKNDSLRKRFDALKYDVKKIEEVVYDLS+RGLV
Sbjct  182  RLLNLKNDSLRKRFDALKYDVKKIEEVVYDLSLRGLV  218
```

Graphical representation

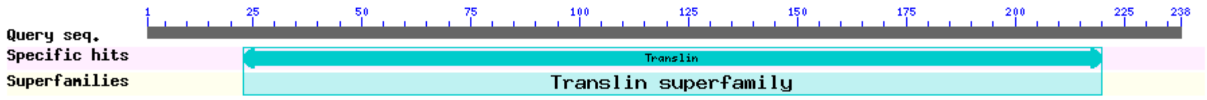

Similar to translin associated factor X

>Cb.comp39981_c0_seq1 len=3487

cDNA

```
TTTTGATATTGTTTTTTCATGGCCTTCTTATATCGAATCGATTCGATAAGAAACGAGGTCCATAGATAAATAATACGAATCCAAATCAAAGCCT
TCCAATGTCCTCGTGTGGAAGAGTGTGGTTATGTGTTTTATCTTGTATGCCTATTGCAATTGGCCCAAGGTATTATGTGTTTCGTGGTCCGTAG
ACACAGCTTATGCAGAATTTTGTGGTTATGTTGTGATATTTAATGTTATTATTACCTTCAAATATATTATAGTTACCTTCATATATGCAATTTT
CATCTAATTATGTCTAAATACAAAGGGCACAGTCGAACAAAGAAAAATAAATTTTCGGTTGGAAAACAGGCGAAACAAGTATTGGAATCAATTG
ATATAGACAATCTTTGTATTCAAATGTTTGAAGACTTTTCTGCAGAGTTAGATGATAAACATGATCGATATGAAAAAATAGTTAAATTTAGTAG
AGATATCACAATTGAATCAAAAAGAATAATTTTTTTACTTCATAATACTAAAACAGACATAGAATCAAAGCGGGACTTGGTGTTGGAAGAGGCG
GCTGACAGACTTTCAAAATTATACAAAACCAATTTCAGAAATATTGCTTTGGAGCTAAAAGGTCAAGACCATTATTTGTATCATAAAGCATTTA
CCAGTGGCATGCAAGAGTTTATAGAGGCTTTTTGTTTTTATCACTATATAAAAAATGAGAATATGCCATTGTGGTTGGATGTAAACAAGTATTT
TCAGTATGAAGAAGATGAACTTAGTTTGTTATTTACACAGTACGATTTTATACTGGGTATAGCTGACTTCACTGGTGAATTGATGAGAAAATGT
ATTAATGTATTGAGTGTTGGAAATATAAATGAGTGCTTTAAACTTTGTAATTTTGTCAGGAACATCCACACTGGCTTTTTAGGATTATGCTTCT
CAGGAAATAAAGAACTCTCAAAGAAGTCAAATGTATTACGACAGAGTCTGGCTAAAATGGAGTTAGTTTGCTACAATATTAAAATTAGAGGGAG
CGAAATTCCAAATCATATGCTGCTGAGTGTTATAGAATCAAATGATAATGAGGTAGATGAAGGCTATGAGCTATGAGGGTTAAAGTGTAGACCT
CTTAAGTCCATTTATATAAATTTTACAGAAGCACAAAATGGTTACATTCTTTGATTTAAAATTTTAAAAATGCATTTTAAATGGAATGCTCTGT
GTTATGCTGAAGAATGTTTTATTGATATCTATAAGTTTTTAAAATTTTTGCTCATTACTTCTTGGTTTGTAAAAACTTATGAGGTTTTACAAAC
CATCAATGTGAACGAAAGACAGCTGAAATAAAGTATTTTTATTTGATTTTTACATTTAGAGTTAACAGAAAAAATGTAAATTTTATTTGGGAAA
ATAAAATGTGTCTATGCTAAATATTAAATAATTAATTTCTATATTAATTGTAATCTTTGTGCAGGTGGTTGTCTTAAACTCAAAAAAACAATTT
ATATAAGCCAAATTATTTAAATATAAGTTGGGGAATCTTTCAGGCTCTGGCCACATGCCTGACATATTTTTTCACTCTTGGGGTTCACCAATGT
ACAATGTGAACACTCTGCACCACCTACTTCAATGGGTTGTTCTTGACCAAGGTCGCGACTCTTACTGCACATTTCACAGATATCTTTTGGTGTT
GTGTTTAAGAAGGTGCATGCCTTGCACATCCATTTAGGTTTGTTCTCTGCTTCCTGTTTATTGTTTAAAACATCCACTACTTTACTGGTGCTCT
GCCTGACCCTTTCATGTGGTTTCTGAAGGGCCTTATTTTGAGAAGGATTTTCTGCTGACGGGTTGGTAGGCATTTTCGCAACATTTTGAGGCTC
TTTCGGCGGTACATTATCATAAGAACTACTCTGAGAGTGTCTTCTTTCCTGAGTTGGTATCTTAATTTTTTCTGAAATGTTTTGTTTTGCATCC
TCGAGATTTTCAGTTGTTAAGGGACGGTCTTTAATTGTCATTTTATTCATGGCTTCATCCAAATTTGTCATTTGGTGTCTCGATTCTCGACTTT
GTTTCCGTTGTTTGTTTCCAGCTGACAAGATATCTCCTCTTTCTCCTAAATCTTTGGTGTACCCTCGGCTTTCTAAATTGCGATAAACATATTC
CCAATTTTCAAGTTCTTCATTTCCACCGCTTTTGGTGACGCCATTATTGTAGGCCACCCCATTTCTATAGGACCTTTCAAAATCAGGTACATCA
TGCGAGGGGTAAGCAGTTTTTCTTGACCTAGATGAAACCGTTGGAGCATCAACAACATCATATCCATTTGGTGTCTCTAATTCAATCAGCTGTG
CAGTAGGAACAGGGTAAGGGCATGAAGAAGGTGTAATAGGAAAACCATTGGGGTAATAACCACTGGGTGGTAAAACAGTAGGTGGTGATCGTTT
AAGTGCAAGGTGAGGATTGAAAGGAACATAATTGCACATTGAGTACATGTGAGGACAACAGCCATTCATGCACATATTATAAGGAGCAGCTGTA
TACTGTGTGACTGCTGGATGAATTTGGGAAGAATATGGGTTGAAGGAATTCCCGTTTTGAATAGCCGGCTGGCTGTGAGAAAGAGTGTTTGGGT
ATTTTGAATGTAAATTGCTTATTGTGTCTGGGTAATGTCCTTGATTTAAGTTATACTGAAGAAATTGTACACACTGCTTGGGATCACTGAGATG
CACCTTACGAAATTCAAGCATATCCAACCAACTAATATTAAACATTGGAGACACTTCCTCCCAAATATGTTTCAAAATCTGACACTCAACATAG
GCAACTAGACAATCTCTGGACACATGTGATACACGATCTGGACAAATTGGTTCATCTAATTGTAAAACACCATTACCAGTATGTTTATATCCCA
TTGCTTCAAATATTTTGTCAGCACCCAAAAGGTTAGCTTCAACTTGGTGCTTATAAAATCCACAATATGTCTTAATTTGATGATACTCTTTTCT
CCATGGCTGCGAGAGCAAGTTACCAGCATACATCTGTATAGCATTAAATCCAGTTGCCGCTTTGTATCCGCTAAAATCTTTTTTATTTGCGGCA
GATCTGTATAATACATCTTCAGTTTCATTTAAAAAAAACTTCTGTTCTGGAGACGCAATATAGAGAAAATCTTGAATGCAAGCCTCTAGTTTTG
ACCGTTGTTCAATTTTGTGGGAGCTCTCATCCATCTCCAAATAGCTGATATGAAGACGGTCTATTTTAAGCCACAATTCTTGAATCTTTTCTTG
ACACAAATAACTCTCCATTTTAACCATGTTATAGCATTTATCGCATTTTACAACAAAAATTCTAGAATATTACAATGTTCTAGATATACATTCT
AAATTTTGTTTTCATAATATTTAAAATTATCAATCATTGAAAATTGATAATTATACTAAACGTTAAAAGTCAAAAGCAGCTGTTTTCAGTCTGT
CGTCTGTCA
```

Protein RF 1: 292->1110 (272AA)

```
MSKYKGHSRTKKNKFSVGKQAKQVLESIDIDNLCIQMFEDFSAELDDKHDRYEKIVKFSRDITIESKRIIFLLHNTKTDIESKRDLVLEEAADR
LSKLYKTNFRNIALELKGQDHYLYHKAFTSGMQEFIEAFCFYHYIKNENMPLWLDVNKYFQYEEDELSLLFTQYDFILGIADFTGELMRKCINV
LSVGNINECFKLCNFVRNIHTGFLGLCFSGNKELSKKSNVLRQSLAKMELVCYNIKIRGSEIPNHMLLSVIESNDNEVDEGYEL
```

Comparison with *Tribolium* PREDICTED: similar to translin associated factor x (548AA)

```
Query  17   VGKQAKQVLESIDIDNLCIQMFEDFSAELDDKHDRYEKIVKFSRDITIESKRIIFLLHNT  76
            +G++ +QVLE+ID +N  I+MF  F  ELD+KHDRYEKIVK SRDITIE+KRIIFLLH+T
Sbjct  6    IGEKGRQVLENIDENNRVIKMFLGFRKELDEKHDRYEKIVKLSRDITIENKRIIFLLHST  65

Query  77   KTDIESKRDLVLEEAADRLSKLYKTNFRNIALELKGQDHYLYHKAFTSGMQEFIEAFCFY  136
             TDIE KR+ VL+EA  RL  +   NF+ IA  LK  D Y Y KA+TSG+QEFIEA  FY
Sbjct  66   NTDIEGKREAVLDEACKRLKVITDENFKTIASILKDFDSYQYQKAYTSGLQEFIEALVFY  125

Query  137  HYIKNENMPLWLDVNKYFQYEED--ELSLLFTQYDFILGIADFTGELMRKCINVLSVGNI  194
             ++ +  +  W  +NK+FQYE+D  + SLLF Q DFILGIADFTGELMR+CIN L VGN+
Sbjct  126  QFLHSNKIESWESINKFFQYEQDGEKFSLLFPQLDFILGIADFTGELMRRCINNLGVGNV  185
```

```
Query  195  NECFKLCNFVRNIHTGFLGLCFSGNKELSKKSNVLRQSLAKMELVCYNIKIRGSEIPNHM  254
            ++CFK CNFV++I+TGFLG+   G KE+ +K+ VL+QSLAKMELVCYNI+IRGSEIP HM
Sbjct  186  SDCFKTCNFVKDIYTGFLGIINPGAKEMGRKTYVLKQSLAKMELVCYNIQIRGSEIPKHM  245

Query  255  LLSVIESND--NEVDEGYEL  272
            L++VIES+D   E DEGY++
Sbjct  246  LVNVIESSDMNTEEDEGYDV  265
```

Graphical representation

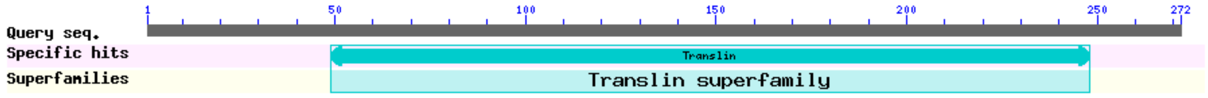

HEN1

>Cb.comp43241_c0_seq15 len=5842

cDNA

AGAAAAACTTCGAAACATATCAGCAACAATCAGACCTTTAAGTAAATCGTAAAGAACCGTAGTATAAAGTTAAACGCGAGGATCTGACATTCAG
CGAGGGAAAACGGCCAAGAATCGTTTATAAACGGTATTGCACAATCCCATTTTGACGTTCTAGATAGTTTAATTCGTTTTTAATGTTTGATTCG
AATTAGAAGGTGTTTTAAGCATATTTGTGTAAAATTGTGGTTAGTTTTGCGGTTTGAGTATTTAATACGTCGAAATAATTTGCAATAATGTATT
AATTCTTGACGGTGCTGGGTGCATGACGTAATGAGTAGTCACATAGAAATAAAGAAAAAAAAAACGTGAATTAAATGAAACGTTGCTCATAAAA
ATTTTAGAGATTGGTGCATTTTATTAATGAAATGGGAATTAATAAAACTTAATCTGGTGGAAATAATCTAGATCAAAGTTAATTAAAATTAATT
GTCCAACACACGAATCACTTAAGAACATTTAAGGATTATTTAAACGACCCTGAATTAGTTTACTTTTAAGTATGAATTATTGCTATTTAAATAT
AAAGATTTTCTTTTGTTGAACGTCTGGAGATAAACTTAATAGGCATCATAATTTTTATTGCAAGTTTTAACGTTTTGGCAATTTTGTTTACCGG
TATGCTACGCATAATTCGGTATAGTCATCAGGTCATCAGTTCGAGTTTTTGAATAAAAAAAAGAACCACGATTTAAAACAAGAATTTATTAAAA
CGGTAATAACATATTAGACATTGTTCAAAATCAACGACAAAAAAGTAAAATTTACAAATACGTGAAAATATTTTTTGAACTTGGATTAAATCTC
TATCACAGACAATTTTATTCGAGAAATGCTACAATGGTTCGGAATACCTTGTTCGAAATCTCTTTAAGTAAATAACCCGTGTCAATTGCACCCT
TTTTAATTCTTTTCAAAGAAATTGTATAAATATAATATTAAGTACCTACTACTCACGCTAAAAAAAATTAAAACCTGCCAATAAAAAAAAATTC
TACAATATTGCAAAAGCTACCGCATATTAAAACCACCGCTCAATCCGTTGTGCGACTTTTTATCTTAATCGAATTAAAATCGGTAAGTTTCATG
GACAATTTCAATTTCTTTCGCAACTTCCTTTCCAGGGGTGGTATGAACAAATTGGGATCGTAGTTTTCATCGTCCACCATCTTCCCAAACAGTT
TCCTAATCCCAAAACTCACCAAGTATCCCACAATGAATGTTATCAGCAGTCCTATCACACCGTTGTACAAATAAGAAACCCGGTATATCCAAAA
ATATTCGTCTTCATTAATTTTGGGACTGTTAGCGATATTCTCAACGAAGTGACTAGAATTTGAGCTATCACAGCCTTCAATGCTCGCCGGCAAA
GCCCGAGGGGCCGGTTTAGGGGCGAAAGCAATCCACAACGATATCGAAACGCCGCAAATCAGCCCCGTCACCGATCCCTGTTCATTAGCTGTTA
ACGTAAACATGCCCAATGAAAATAGTCCCAATAAGGGCCCACCGACGACCCCGAAAATCGTAAGCGCTGCTTGGAGCACCCCTCCTAATAGCTG
CGCCAAAAAAGCTACCCCAATACATATAAGACCATAGATAAGGGCTATAATTTTGGTCCGAACGGATATTGCGTTCAAAGACATGTTCCGTTTA
AATATTAGTCGGTGTAAGGGTTTGTAGTAATCCTCGACCGTCACTGCGGCGAGGCTGTTTAAAGCGGCCGACACGGTTGATAAGGACGCGCAGA
AAATACCGGCGACGAACAATCCTGAAAGACCGGGGACTGCCCCCATAGTGTCAACTACATAAAATGGCATCAATTGATCTATGCTATTTACGTG
GCCTGCTTTGACTGGATCACAGTTAAAGTATCTAGAGTAAATACACAGACCAGAGAAGGAGGTGCTTATGCTTAGAGTTGTCAGAATGGGCCAG
TTCCACCAAAGCGCTATTTGGGCCCGTTTCAGATCTTTGATGGTCAGGTAGCGCTGGACTTGTGTTTGATTAACGGCGTAGAGCGAGAGAAATG
TCACCCCGCCACCGATTATAAGCGAGAACCAAGAATGTCTTACCGTCGGATCTGGGTCGAAGTTTAACAGCTCAGTCCTGTTTCCGTTTTCCGC
GATTCTCCATATTTCCGCAAAAGTACCGTGTTCTTTCAACCCATAACCAATAACACTGAACACCGCCACAAACATTAACAGTGACAAACCTATG
GCAAGAATGGCAATTTCTTTCGTGATGCCTGTCAGAGCCTCCAACGCTAAGGCAGGGGCGTAGACCACGATTCCCATGTACAAAGTCATTTGCA
ATGTGTAAGAGAGGGATGCTGCCAGTCTTGAAGTTTTTCCAAATCTGAGCTCCAAATATTCATATGCGCTCGTCGCCTGCAGCTTAAAAAATAC
GGGTAGGTATAAATAAGCAGCGATTGGCGTGAAGAGTCCATATGAAATGTTGATAACTATAAACTGAAACCCGTAAGTGTAATTCTCACTCGAG
ACTCCCAGTATTGTTATAGCAGACATGAATGAAGCCATTAACGAGAACGCCACCGGCGCGATGGACATATTTTTGTCCGCTAATAAATATTCTT
GCATGGTTTTCTGTTTGCCGCCCGTAAATCGGTAGTATATCCCAATGAAGGAGGAAATTAATAAGACTACTGCTATTACCACATAATCCCAAAT
ACCGAATACTTCCATTTCGACTGATTTACAAGCAAATAACGAATTACACAATGAGGCGACCTACATTTATCTTATTAACAAACCTTAAGATAAG
TTTACTAAAAACTAGAAGAACATTTCAAAATCTCCAGGTTTTATAACAAAAAAAAAGCACGGAAATTATATTGGTCAGTTGTAAAAGTGACAAA
CGCGGAAAGAAGAATGTTAAAATAACCTCGTCATGTCAATATTTGAGTGTTAAATTTAAAAAGTTAAAAATGATATTTGTGTTCCATTGCCTGA
ACATGTTAATTTATCGGCTGTTTAGAAATATAGTAAGGCTGAAAGAATTCGAAATTTCGCCAGAGACATATTCGGTATATGCGGGCGAAGTAGA
AGCGGAGTATGAGTTAAAATTCGACCCTCCCGTTTTTAAGCAAAGATACGAAAAAGTTTATCACATATTAATAGACAAACGATGGAGAACCAAA
ATTAGAAAACTTGTAGATTTTGGTTGTGCCGAATTCGGTATGTTCATATTTCTCAGGAATCTCAATATTGAAGAAATGTTCTTTATCGACATTG
ATGAAGTTCTGCTTGAAGAAAAGGTGATTAGAATAGAACCGCTGTTTTGTGACCATTTGAAACGGCGTAACTATCCCTTGCAAGTATCAGTGTT
TGCAGGCAGTGTTTCAGACCCTGATTATAGGTTGAGGGATACAAGCGTAGTCACTGCTATAGAACTAATTGAACATCTCTATCCAGACATTCTG
GATGCTTTCCCCTACAATGTGTTTTCATTTATTGAACCAAAACTTGTGATAGTATCAACACCGAATGCAGATTTCAATGTGTTGTTTAATAAAG

TCAATCTATTTCGGCACTATGATCATAAGTTTGAATGGACCCGGCAGCAGTTTGAAGATTGGGCTCAAAATATTGTCCAAAGATTTCCTGATTA
TACAGTTGAATTCTCTGGAGTAGGATGGGCTCCTTCCGGTTCTAAAGACCTTGGCACCTGTTCACAGATGGCTTTGTTTGTGCACAAAAGTTTT
ATTTGTGAGAAATATATATCCACCTCTTATACCCGGCAGTGTTTGTGCCTGGTTGACAGTTTTTGCAAAAGTGCAGTCTCAGAAGGTAAACTTT
TGTGCAAGTGTGTCTGTCAACTGTGTATGCCAGACCATAGCGTTGGAATTTGCACTTACTCCAGTCTCTCAGCTGGTCCTGGGAAATTTAAAAA
TCTGGAGTATAACCCGTCTTTTAATATTTACTATAAGATGGTGCAGAAAATTGTTTATCCTTATGAAATAGATGACAAAACTGATGAGGAAAGA
TTACTGGACACCTTTAAGTATAGGATACACACTTTTGGTTCTGTCAACAGCAGATTTTATGTCGAAAAACGCGAGAGGTGCGAAATCCCAGTAA
TGGACATTATGTACGGAAATGTGGACATATCAGAGCATGAAGTATGTGAAATATTAATAAGATCCGGTTACACCATAGAAAAATGTGTTATACC
AGAAACACTGCTGAGTGAGAACTGCATTATTTGGGAGCCTCAGAACATGGAAAACTTTAGCAGCACTTCAGAAGCTTCAAGTTACAGCAGCGAG
TATACCCCTGGCGTTAAGACAGAGTCTAATAGGGATTGCGAACCCATTTCTGATTGGGATGAGTTTCGAGCAGACGAAAAAAGCCAGTGGGCAA
GTTACGAAGAAGACAAAGAGCCTGAGAAACTTTTTCAAACAGAAAATAAACCGCAGTTGGATTCCCTATTGGACTCTGGTTATCAAAAGTCCCC
TTCACCGCAAGATGACAGTCCACAGTCCCTTTTAGAGGATTTAGAGAAAGCAGACCACTTTGAGGATGGTAAAACGACGCCTAATCTTGCTGGA
AACGATAATAGGATTCACTTGAAGAGACTGATTAGGGAAAAACGCGTTTCGGTGCCAGCAGAGGCAAACAGTGTCAAATCGGAAACGGTTAAGG
TAAGCGACTCGGACAAAATTTTTACGACGAAAACTGTCGCGCACGGCGTTTTAAAACCGGTGAAAAAGTTGGCCGCGAAAGATTTCGCGATCGC
GGGCCCGTCGTCTTCCCTGAAAAAGAAAAAGGTCAAGTTGAAGTGTGAAGATAGTTCAGAAGAAGACGTCAAAAGCATCGCCGACTGTATAATT
CAAAACAGTTTGAATAAAATCGATGTCGAATGCGAAAGCGCCGCCGAATTGATCCACTACGTGGACGATGTCGAGGAGCGGCCGATTATCGATG
ATCAGCACGCCGAAGAGCCCCAGGTGGTCGAGAACGGCGATCTGGCCAATAATAATAGAGACGATGAAGGTAATAATTTTGCCGGCGATATTCG
AGTACCCGACGAAGGGGCGGACGATTTAAACGACAATGTGGAAGAGCCGGTGCCGCCGTTGCAGGATAACGGTGCCGGTTTGGAAAATAATAAC
GAGGACAACCTTCGCGAACGCGATGAAGGTGTGCAGGATGTTCCGGCAGTACAGCATATCGAAGAAAGGCTGGAACCTTTGATTGAGCCAGATG
AGGTATCCAGCGCCAGCAGGGAAGCGTTGTTCGATCCACGCTCGGAGCAGGACCTTTTGCAGGATTTCGATCAGAGACCTCAAGCGGACGACGA
TCAATCGAACGGAGGCGCGTACGAGCCGCAAGCAGCCTCTGTTCAAATCGGTAATTTTCCTGGCTGGCTGTTGCAGATCCTGGGGGGCCACCGC
ATATCGTTGGAGTCTCCGACTCCAATTGAGACCCACTTTTACTGTCAGGGCGACGGTTTAGGCGTGCATCCGTCCGTGACCACTCTCGACGCGA
ACGGAGATGCGGATGATGACGAGACAGAGTCCTCGAATAATACAGCCGATTTTGCGGAAGTGGAGCCGGGCGCATCGGATCATGACACAAGCTC
TTTGCCTGAAGAATCTCTAGCAAACAGCAGTATTAACGAGGGAAGTGAGGCTAGAGAGATCTCGTCATTATCCGATGAGTGTTTTAGCGGTGTG
CAGAAAACAAAATGACTATTTTATAAACCTGTATGGTTTATCATAAGACTGAAAGTTTTTCTACAGGCTCTTGATTAAACGCCAAACATCCGGT
AAAAAAAAAAAAAA

Protein RF 2: 2984->5749 (921AA)

MIFVFHCLNMLIYRLFRNIVRLKEFEISPETYSVYAGEVEAEYELKFDPPVFKQRYEKVYHILIDKRWRTKIRKLVDFGCAEFGMFIFLRNLNI
EEMFFIDIDEVLLEEKVIRIEPLFCDHLKRRNYPLQVSVFAGSVSDPDYRLRDTSVVTAIELIEHLYPDILDAFPYNVFSFIEPKLVIVSTPNA
DFNVLFNKVNLFRHYDHKFEWTRQQFEDWAQNIVQRFPDYTVEFSGVGWAPSGSKDLGTCSQMALFVHKSFICEKYISTSYTRQCLCLVDSFCK
SAVSEGKLLCKCVCQLCMPDHSVGICTYSSLSAGPGKFKNLEYNPSFNIYYKMVQKIVYPYEIDDKTDEERLLDTFKYRIHTFGSVNSRFYVEK
RERCEIPVMDIMYGNVDISEHEVCEILIRSGYTIEKCVIPETLLSENCIIWEPQNMENFSSTSEASSYSSEYTPGVKTESNRDCEPISDWDEFR
ADEKSQWASYEEDKEPEKLFQTENKPQLDSLLDSGYQKSPSPQDDSPQSLLEDLEKADHFEDGKTTPNLAGNDNRIHLKRLIREKRVSVPAEAN
SVKSETVKVSDSDKIFTTKTVAHGVLKPVKKLAAKDFAIAGPSSSLKKKKVKLKCEDSSEEDVKSIADCIIQNSLNKIDVECESAAELIHYVDD
VEERPIIDDQHAEEPQVVENGDLANNNRDDEGNNFAGDIRVPDEGADDLNDNVEEPVPPLQDNGAGLENNNEDNLRERDEGVQDVPAVQHIEER
LEPLIEPDEVSSASREALFDPRSEQDLLQDFDQRPQADDDQSNGGAYEPQAASVQIGNFPGWLLQILGGHRISLESPTPIETHFYCQGDGLGVH
PSVTTLDANGDADDDETESSNNTADFAEVEPGASDHDTSSLPEESLANSSINEGSEAREISSLSDECFSGVQKTK

Comparison with *Tribolium* (AA)

```
Query  40   EAEYELKFDPPVFKQRYEKVYHILIDKRWRTKIRKLVDFGCAEFGMFIFLRN-LNIEEMF  98
            +AE ++KFDPPV+KQRYE+   IL+D++W+ ++ K+VDFGCAEFG F+FL+N L++ E+
Sbjct  28   DAENDIKFDPPVYKQRYERAVDILLDEKWKNQVNKVVDFGCAEFGFFVFLKNRLSLSELL  87

Query  99   FIDIDEVLLEEKVIRIEPLFCDHLKRRNYPLQVSVFAGSVSDPDYRLRDTSVVTAIELIE  158
             IDID++LL + + R+ PL  DHL  R  PL V+V+AGS+++PD  L +T  V A+E+IE
Sbjct  88   LIDIDDLLLNDYLYRVYPLNADHLVGRPKPLTVNVYAGSIAEPDPSLLNTDAVIALEIIE  147

Query  159  HLYPDILDAFPYNVFSFIEPKLVIVSTPNADFNVLFNKVNLFRHYDHKFEWTRQQFEDWA  218
            HLYPD LDA PYN+FS+I PKLVIV+TPNA+FNVLF K+  FRH DHKFEWTR+QF+ WA
Sbjct  148  HLYPDTLDALPYNIFSYIRPKLVIVTTPNAEFNVLFTKLQKFRHADHKFEWTREQFQSWA  207

Query  219  QNIVQRFPDYTVEFSGVGWAPSGSKD-LGTCSQMALFVHKSFICEKYISTSYTRQCLCLV  277
             NI  RFP YTV+F GVG  P G+ D +G CSQ+A+F+ K  IC+ Y  T
Sbjct  208  TNITSRFPSYTVQFDGVGLGPHGTDDSIGCCSQLAVFIRKDVICDTYEET----------  257

Query  278  DSFCKSAVSEGKLLCKCVCQLCMPDHSVGICTYSSLSAGPGKFKNLEYNPSFNIYYKMVQ  337
                                          C  S+ S G               YYK++
Sbjct  258  ------------------------------CNVSNDSCG---------------YYKLIA  272

Query  338  KIVYPYEIDDKTDEERLLDTFKYRIHTFGSVNSRFYVEKRERCEIPVMDIMYGNVD--IS  395
             I YPY++D +T++E++LD  KYR+H F +    FY  + +  +IP+  ++Y
Sbjct  273  SINYPYDVDTRTEDEKILDELKYRMHLFENSEEEFYNVETKCFQIPLNQLIYHITKPFPP  332

Query  396  EHEVCEILIRSGYTIEKCVIPETLLSENCIIWEPQNMENFSSTS--EASSYSSEYTPGVK  453
            E ++ +IL++  Y IE+C  P T   E+C+I+E + M++  S S  EAS Y S+
Sbjct  333  EPDIRKILLKYNYKIEECRNPITKKLESCVIYEAE-MDSGGSGSDTEASGYGSD------  385

Query  454  TESNRDCEPISDWDEFRADEKSQWASYEEDKEPEKLFQTE--------NKPQLDSLLDSG  505
             + N D    SDWDE      S  +   E     K  +T+          PQ  +L DSG
Sbjct  386  NKYNVDEGKFSDWDENDLTWTSSTSKENEPAVSSKGVKTQLHLEVNESKNPQ--ALFDSG  443
```

```
Query  506  YQKSPSPQDDSPQSLLEDLEKADHFEDGKTTPN-----LAGNDNRIHLKRLIREKRVSVP  560
            YQKSP   DDSPQS  +D   A  F+     PN     L+  DN  +   L
Sbjct  444  YQKSPP--DDSPQS--KDQAVALDFKSLNDRPNKLSHILSVFDNFDKINEL---------  490

Query  561  AEANSVKSETVKVSDSDKIFTTKTVAHGVLKPVKKLAAKDFAIAGPSSSLKK----KKVK  616
               ++V+ E    +     F +      V+K + +  AK   IAGPS   KK    KK +
Sbjct  491  ---DAVRREKKYFN-----FASNLPHREVMKKICQEIAK-HPIAGPSRDPKKGKQLKKSQ  541

Query  617  LKCEDSSEEDVKSIADCIIQNSLNKIDVECESA-AELIHYV---DDVEERPIIDDQHAEE  672
                D S +DVKSI  CI++NSLNKI+ + E     LI  +   +++EE P++     E
Sbjct  542  SSDSDESVDDVKSITTCILENSLNKIECQDEDIRGNLIQELIPEENLEEIPVV-----EP  596

Query  673  PQVVENGDLANNNRDDEGNNFAGDIRVPDEGADDLNDNVEEPVPPLQDNGAGLENNNEDN  732
              +VENGDLANNNRD EGNN+          A+D+  N E     + DN   +  NN D
Sbjct  597  ILIVENGDLANNNRDLEGNNYP---------AEDVEQNDE-----IVDNQELINANNND-  641

Query  733  LRERDEGVQDVPAVQHIEERLEPLIEPDEVSSASREALFDPRSEQDLLQDFDQRPQADDD  792
                +  V      +     +EP ++  EV+ +SREALFD  S+ DLL+DF+   +A D
Sbjct  642  ---VEVAVAQAEEDEEENIEIEPPVDV-EVAWSSREALFDINSQVDLLEDFEM--EASDV  695

Query  793  QSNGGAYEPQAASVQIGN----------FPGWLLQILG-GHRISLESPTPIETHFYCQGD  841
              NG ++      V   N          FP WLLQI      +  E     E HFYCQGD
Sbjct  696  VVNGVSFPNSCVIVAENNDPVLPPEESGFPNWLLQIFDEAEVLPPEDDLHDEPHFYCQGD  755

Query  842  GLGVHPSVTTLDAN-----GDADDDETESSNNTADFAEVEPGASDHDTSSLPEESLANSS  896
            GLGVHPS   ++ +     G+    ++ SS+ + DFA     A DH   S+ E  +A+ S
Sbjct  756  GLGVHPSTVAINDDDADDEGNDSGSDSSSSSGSVDFA----NADDH---SVQEVFVADES  808

Query  897  INEGS  901
             N+ S
Sbjct  809  RNDNS  813
```

Graphical representation

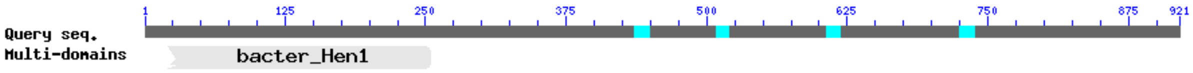

Similar to gawky CG31992-PA

>Cb.comp42534_c0_seq11 len=5194

cDNA

CGCAATGCCACGGCGTAAATCTCCGACACCGTCCGAGTCGCGTAGTCGGTTATTGTGCGGCGACGCTGCCACAGTGCCACGTAATTGCGGATCA
ATAAACAAAAATCCGTGTGGTGCCCCCCCTCCGCGCGTATGCAAGTATTTTAACGCCAAATCGTTTAATAAATATTGTGATAATAAGTATTCTC
ATTAGTTCAGTGTGATAAAATTGCACCGGCTCTGTACAGTTTCGCGACGACAAAAAGATCTCTGGCTGGCCAGACTGATATGGAGCACTAGTGA
TGATGCGCGCCCCTACCCCCTCCGAGCCGAAGTCTAATACATTTCCTACCTACCAAGTGCCTCAAGAGTCAGCCATGAGGGGCAGCGCACCCCA
ATCAGTACAACACAAAGTCGCTCGGGCAGCTTGGGGGGGTCGAGCCGACCCCCCGACCAGCCCCCTCGGCGCCGCCCACGACGGTGGCGCCACC
CCGTCTGTGATAACTGGCTCAAGTTGCCGTCCCGACGCGATGACGATTACACGCGCGCCAGGCGGCAACTCTAATGTTAAGATGCAATCTGTGA
CCGATAACTGTCTTCTGAACTCTGTTACCGTACCAAAAATACAACGTAACGGCCGTCACACGGCCACCCACAATAATTTAAATAATAATATAAA
TAGCGATAAGTTAGATGATAGTAAGTTTGGTGCTTTGCCCCTCGGACGAGACATTCCCAACCAAAAGTCTGATGACCTCGAAGTACTCGACCAC
TCTAGTGCACTGAAGTTTATGCTTAACTTAAATGCTTTTAACTGCGATAACAACGATCACGACAAAGAACGTATCAACAACGGCGACTACGATC
AGGATGCCGAATATTTGTGGAACAAGATGCAAAAACTGCGCACAACAAACGAAGACGACGACTTTGTGGGTTGTTACGCCCCCCTCAGGCTCAG
AGGCGGTGGTGAGAGCTCACTTAGCACGGGCACTTCCGGCTGGGGCACGCCCCCGGCACAGCAGGCCAGTAATAACAATGCCAACAAATCGAAT
ACGAACGGACAACAACAACAACCCCCAACTACTCAGTCGAATAATTCGGGATGGGGACAGCCTGGTGCTAAACCGCCGACCAATACCAACGGTC
CGGCCACGTCCAGCGCGTCTGCCAACAACGCGACACCGAACAACAACCAAAACAATTCGACGACATCGACCAAACAGCAACTCGAGCAATTGAA
CAACATGCGGGAAGCAATCTTTAGCCAGGAAGGTTGGGGAGGGCAACACGTCAACCAGGACACAAATTGGGACATCCCGCCCAGTCCCGAGCCG
CAAATCAAGATGGACGGTGGCGCGGGTCCACCGCCATGGAAACCCGCGGTGAACAACGGCACCGAATTGTGGGAGGCGAATTTACGTAACGGCG
GTCAGCCGCCCCCGCAGCCCCAACAGAAAACGCCATGGGGACACACCCCCTCGACGAATATCGGCGGTACATGGGGCGAAGATGACGACGCGAC
CGACAGCTCAAACGTATGGACGGGCGTGCCTTCCAATCAGCAGCAGTGGGGCAACAACGGCGGCAACGCCATGTGGGGCGGTGCAGGGGCCGCA
GGCGCAGGTACTGGCGGTACCGCGCCGACGCCTGGTTGGGGCGACCCGCGGGCTTCCGATCCGAGAGCCGCGGTGGCCGCGGTGTCTGCCATGG
ACATGCGGCCGGACATGAGAGTGGCGGCGGCGGCGGCGGCTGGTAATCTGGACCCGAGGCAGCTCGACCCGCGCGAACAAATGAGACACATGAC
AGGCGGCGGCGACATGCGGGGAGACCCGAGAGGTATCACGGGAAGACTGAACGGCGCCGGTGCCGAGTTTTGGGGTCAAACCGGGCCCCTGGGC
GGCCCAGCTGGTATGCATCATCAAAATAAAATGCCCGGTGTTGGTCCCGGTAACGGGACAGCCTGGGATGAACCCTCGCCACCCTCACAGAGGC
GCACCATGCCCAACTACGATGACGGAACATCTTTATGGGGCAACCCCCAACCAGGAGCAGGCGGTATGGGTGGTCGCGGGGGCGGTCCGAGCGG

TCCACCCGGAATGGCCCCCTCCCGCGGCGGTGGAGGCCTGAAACCCGACGGTTCCGTGTGGGGCGGCGGCCCGGGCAGTGCCGGCGTCGGCCGC
GGCAACGGATGGGACGATGTTGGGGCCGGTCCGACCGCGGGCGGCGGCTGGGACGACCCGAGCGTCGGCCCGTGGCCTAAGCAAAAAATTCCCG
GCGCCGCAAGTGGTCTGTGGGACACGGGAGATTTAGACTGGAGCCACAAGCAGAGTATCAAACCCCAACTGACGAAGGAAATGGTCTGGAATTC
GAAGCAATTCAGGACGCTGGTTGATATGGGCCACAAGAAGGAGGACGTAGAGAATGCCCTAAGGATGCGCGAAATGAACTTTGACGAAGCGTTG
GACATGTTGAGTCTTCCGCGCAACCGCGCTGATCCCAGCTGGATGAGCCGCCACGACGATCACTACGACCATCCCCAGTTCCCGGGTATGGGTG
CGCAGCGGGGATTCCCGAACGTCGTCGGGCCGGCAAATCCGTTATCGAATGCGTTCCCCCCGAACAACGCGCCCAATTTACTGAACAACATGCC
CGGGGCGGGCGGACAGTCGAGCAGCTCGCTTATAAACAACATTTCCCCCGCGATAATGCAGAAGATGTTGACTCAGCAAGGGGGAGTGGGCGGC
GCGGCCCAAAGCTTTGGCGGAGCACCCGCCGGCGGGCGACCCCTGCAGCCTCAGTCTCAGCCCTCCACCCAGCAACTCAGAATGCTAGTCCAGC
AAATCCAGATGGCAGTTCAGACGGGATATTTGAATCATCAGATCTTAAACCAGCCTCTGGCACCCCAAACGCTGGTCCTCCTGAACCAGCTATT
GCAGCAAATCAAAACGTTGCATCAACTAAACAATCAGCATCACATAGCGGCCGGCTCGGGCAAAGGAAACTTGAGCAACAACGTACTCTTGAAT
TATTCCGTAATGATTACAAAAACCAAGCAGCAAATTTTGAATATTCAGAACCAAATTCAGGCGCAGCAAGCGCTTTACGTCAAACAGCAGCAAA
ATAGCGGCAACATCAGTTACGATTCATTCAAGACTAACACAATGCACGACACGATTCACGCCCTCCAAGGCAATTTTGCGGAGCTCGGCATCGC
CAAAGAGTCCCAAGTGAATCAGCAGCAATCGAGACTTAACCAGTGGATAAACAAGGACAAGGAGGAGAACGGCGAGTTTAGCCGAGCTCCAGGC
TCATCTTCCAAGCCCGTAGCTACCTCCCCCAACATGGCTCCTCTCGGTTTAACCCAGCCGGACGGGCCGTGGTCGACTGGTCGCACGGGTGACA
CCGGATGGCCGGACTCGAGCGGTGGCGATTCCTCGAATGATAAAGACGCACAGTGGGCAACGACTGCTCAACCTTCCCTGACTGATCTTGTACC
TGAATTTGAACCTGGAAAACCGTGGAAGGGCAATCAGATTAAGATAGAAGACGATCCCAGTATTACGCCCGGTTCGGTGGTGCGTTCACCGTTG
TCAATCGCCACGATCAAGGATAACGAACTGTTCAGCATGAACACGAGCAAAAGCCCGCCGGTCACCGACGCAATGCAGTCCTTGAGTCTCAGCT
CGTCTACTTGGAGCTTTAATCCACCCTCTACCTCTAGCGCGTTTACGAGCAGCCCTCAAATCAAACTGCCCACCACAAAGGGCGGTCTCGGCGA
CCTGAATCCTTCGATGGCGATCACGTCGGAGCTGTGGGGCGCACCCAAATCTCGCGGTCCGCCGCCCGGTCTCTCCGGCAAAGGTGGCGGCGGC
GGCGGTGGCGGGGGTGGCGGAGCCCCACTCGCCAACGGCTGGTCGAGCAATATCGCGGGATCGGTGCCTTGGGGCGGAGCCTCGGGCAGCGCCG
CCCAGCGCAACTCTGGCAACTGGGGCGTGTCACAGTCGCAGTGGCTGCTACTTAGGAACTTGACGGCTCAGATCGACGGTTCGACTCTACGCAC
ATTGTGCATGCAACACGGCCCATTGCAAAGTTTCCACCTCCACCTTCATCAGGGTTTCGCACTTGCCAAGTATTCATCCCGCGAGGAAGCCACT
AAAGCGCAGACCGCGCTGAACAATTGCGTCCTCGGCAACACGTCTATTCTTGCGGAGAACCCAACCGACTGGGACGCGAGCACCCTACTCCAGA
GCATCGCCAATCAACAGGGCGCGTCATCGGGCGGATGGCGGGCGTCGTCCTCTAAACCGGGCGTCGCGGCCGGAGACACATGGAGCACCGGTTG
GCCGAACAACCCGTCCGGTGTAGGCCTGTGGGCGACCACGTCGCTCGACACGAACGACCCCGCCCGGGCCACGCCTGCCAGCCTTAATTCATTT
TTGCCGAATGACCTCTTGGGCGGTGAGTCTATGTAAAAGGAAAGAACGCGAGTAGAATTAAAACAGAACAAAAAAATTATCCAGAGAGAGACTA
CACAAAAACGGTTATTCCTAATACTGAGATATAGTAAGTGTGGATGGGAAAAAAATTTTATTGATGTTTTAAAAGTTTTTTTTTATTATTATTA
TTATTGATTATTAATAATTATTATTGTAGGTCGAGTATAAAATTTTTGAAATGATATTTATTGTATTAATCGATTAAGACACTTGGACTTTTTA
ACGCTGTATAGGTGTAGACATATCGCTACTTTTAATTTTGACGGTGATGTAGTTTTCGTTAGGGAGTTTTGTTTATTTTTTTTTAATTCTCTTA
AGTTACGGACTAAAGATTAAGGTTATGCGGTTATGCCAAATGTACACTGTTCGTTTATCGTTTTGCGACGGCGCGCCTCACGCCCAATCCGCGT
CGAGTCCCCACCCACGCGAAGTGGCGTTGAAAAAAAACTGGTACCGCCGCGCCGGGAGGTCGTACACGCGGGTAAATAACTGTTGAGAAATATT
TTTAAAAAATGTCGGATTCGAATACCTCTACCCCCGCGCCCGAAAGTAAACTCCCCGCGGTACATTTGACCTACTACTTACGGTGCACCGTACT
ATAAATTTTGCGGGTTGTTTATGAGGCGAATCGTCCGCGAACGCCGGAATTGCGTTATTTAATTTTTTCCTCTTTAAGTTACGGAGTTGTTGC
CATTTAGTTTTTCGTTTTTTTTCT

Protein RF 3: 282->4454 (1390AA)

MMRAPTPSEPKSNTFPTYQVPQESAMRGSAPQSVQHKVARAAWGGRADPPTSPLGAAHDGGATPSVITGSSCRPDAMTITRAPGGNSNVKMQSV
TDNCLLNSVTVPKIQRNGRHTATHNNLNNNINSDKLDDSKFGALPLGRDIPNQKSDDLEVLDHSSALKFMLNLNAFNCDNNDHDKERINNGDYD
QDAEYLWNKMQKLRTTNEDDDFVGCYAPLRLRGGGESSLSTGTSGWGTPPAQQASNNNANKSNTNGQQQQPPTTQSNNSGWGQPGAKPPTNTNG
PATSSASANNATPNNNQNNSTTSTKQQLEQLNNMREAIFSQEGWGGQHVNQDTNWDIPPSPEPQIKMDGGAGPPPWKPAVNNGTELWEANLRNG
GQPPPQPQQKTPWGHTPSTNIGGTWGEDDDATDSSNVWTGVPSNQQQWGNNGGNAMWGGAGAAGAGTGGTAPTPGWGDPRASDPRAAVAAVSAM
DMRPDMRVAAAAAAGNLDPRQLDPREQMRHMTGGGDMRGDPRGITGRLNGAGAEFWGQTGPLGGPAGMHHQNKMPGVGPGNGTAWDEPSPPSQR
RTMPNYDDGTSLWGNPQPGAGGMGGRGGGPSGPPGMAPSRGGGGLKPDGSVWGGGPGSAGVGRGNGWDDVGAGPTAGGGWDDPSVGPWPKQKIP
GAASGLWDTGDLDWSHKQSIKPQLTKEMVWNSKQFRTLVDMGHKKEDVENALRMREMNFDEALDMLSLPRNRADPSWMSRHDDHYDHPQFPGMG
AQRGFPNVVGPANPLSNAFPPNNAPNLLNNMPGAGGQSSSSLINNISPAIMQKMLTQQGGVGGAAQSFGGAPAGGRPLQPQSQPSTQQLRMLVQ
QIQMAVQTGYLNHQILNQPLAPQTLVLLNQLLQQIKTLHQLNNQHHIAAGSGKGNLSNNVLLNYSVMITKTKQQILNIQNQIQAQQALYVKQQQ
NSGNISYDSFKTNTMHDTIHALQGNFAELGIAKESQVNQQQSRLNQWINKDKEENGEFSRAPGSSSKPVATSPNMAPLGLTQPDGPWSTGRTGD
TGWPDSSGGDSSNDKDAQWATTAQPSLTDLVPEFEPGKPWKGNQIKIEDDPSITPGSVVRSPLSIATIKDNELFSMNTSKSPPVTDAMQSLSLS
SSTWSFNPPSTSSAFTSSPQIKLPTTKGGLGDLNPSMAITSELWGAPKSRGPPPGLSGKGGGGGGGGGGGAPLANGWSSNIAGSVPWGGASGSA
AQRNSGNWGVSQSQWLLLRNLTAQIDGSTLRTLCMQHGPLQSFHLHLHQGFALAKYSSREEATKAQTALNNCVLGNTSILAENPTDWDASTLLQ
SIANQQGASSGGWRASSSKPGVAAGDTWSTGWPNNPSGVGLWATTSLDTNDPARATPASLNSFLPNDLLGGESM

Comparison with *Tribolium* PREDICTED: similar to gawky CG31992-PA [Tribolium castaneum] (1014AA)

```
Query  316   MREAIFSQEGWGGQHVNQDTNWDIPPSPEPQIKMDGGAGPPPWKPAVNNGTELWEANLRN  375
             MREAIFSQ+GWGGQHVNQDTNWDIP SPEP +KMDG A PPPWKPA+NNGTELWEANLRN
Sbjct  1     MREAIFSQDGWGGQHVNQDTNWDIPGSPEPSMKMDGSA-PPPWKPAINNGTELWEANLRN  59

Query  376   GGQPPPQPQQKTPWGHTPSTNIGGTWGEDDDATDSSNVWTGVPSNQQQWGNNGGNA-MWG  434
             GGQPPPQPQQKTPWGHTPSTNIGGTWGEDDDA DSSNVWTGVPS QQQWGN   +  MWG
Sbjct  60    GGQPPPQPQQKTPWGHTPSTNIGGTWGEDDDA-DSSNVWTGVPSGQQQWGNTANSGGMWG  118

Query  435   GAGAAGAGTGGTAPTPGWGDPR-ASDPRAAVAAVSAMDMRPDMRVAAAAAAGNLDP-RQL  492
             G     +  G  A  PGWGDPR A+DPRA    +   DMRPD+R      AG+ DP R L
Sbjct  119   GPKKE-SEWGAAAGNPGWGDPRTATDPRA-TGGIDPRDMRPDLR---DMRAGSSDPMRLL  173

Query  493   DPREQMRHMTGGGDMRGDPRGITGRLNGAGAE--FWGQTGPLGGPAGMHHQNKMPGVGPG  550
             DPREQMR    G DMRGDPRGITGRLNGAGA   FWGQ GP  G   +HHQNKMP VGPG
```

```
Sbjct  174   DPREQMR--LAGSDMRGDPRGITGRLNGAGAADAFWGQAGPHTGTQHIHHQNKMP-VGPG  230

Query  551   NGTAWDEPSPPSQRRTMPNYDDGTSLWGNPQPGAGGMGGRGGGPSGPPGMAPSRGGGGLK  610
             NG  W+EPSPP+QRR MPNYDDGTSLWGNPQ GA  MG   G  +GPPGMA SR    +K
Sbjct  231   NGAGWEEPSPPTQRRNMPNYDDGTSLWGNPQQGAS-MGR--GSTAGPPGMAQSR----IK  283

Query  611   PDGSVWGGGPGSAGVGRGNGWDDVGAGPTAGGGWDDPSVGPWPKQKIPGAASGLWDTGDL  670
             PDGSVW         GR   WD+ G G      WD+ SVG W KQK+  A + LW   ++
Sbjct  284   PDGSVWC-------HGRNGSWDETGPG------WDE-SVGGWNKQKM--AGTHLWGDNEI  327

Query  671   DWSHKQSIKPQLTKEMVWNSKQFRTLVDMGHKKEDVENALRMREMNFDEALDMLSLPRNR  730
             DW H +  K  LTKEM+WNSK FR L+DMG+KKEDVE ALR  +MN+++AL++L    +R
Sbjct  328   DWGHNKGPKQNLTKEMIWNSKCFRMLMDMGYKKEDVETALRRGDMNYEDALEILG---SR  384

Query  731   ADPSWMSRHDDHYDHPQFPGMGAQRGFPNVVGPANPLSNAFPP-NNAPNLLNNMPGAGGQ  789
                 W +RHDDHYDH QFPG   QR FP+  GP   +S  FP  NNAPNLLNNM  +GG
Sbjct  385   NPDGWRNRHDDHYDHQQFPG---QR-FPS--GPPGQMS--FPQGNNAPNLLNNMNSSGG-  435

Query  790   SSSSLINNISPAIMQKMLTQQGGVGGAAQSFGGAPAGGRPLQPQSQPSTQQLRMLVQQIQ  849
              ++SLINNISPA + KMLTQ GG      +   A   GR LQPQSQPSTQQLRMLVQQIQ
Sbjct  436   PNNSLINNISPAGVHKMLTQGGGGSQGFGAVSAA---GRNLQPQSQPSTQQLRMLVQQIQ  492

Query  850   MAVQTGYLNHQILNQPLAPQTLVLLNQLLQQIKTLHQLNNQHHIAAGSGKGNLSNNVLLN  909
             MAVQ GYLNHQILNQPLAPQTL+LLNQLLQQIKTL QL  Q  +A         N+ LL
Sbjct  493   MAVQAGYLNHQILNQPLAPQTLILLNQLLQQIKTLQQLMTQQSVAQSQCINGKPNSALLQ  552

Query  910   YSVMITKTKQQILNIQNQIQAQQALYVKQQQNSGNI---SYDSFKTNT-MHDTIHALQGN  965
              SV+ITKTKQQI N+QNQI AQQA+YVK QQN G+I     D FKT   MHD+I+ALQ N
Sbjct  553   CSVLITKTKQQITNLQNQIAAQQAIYVK-QQNHGSIGGGQSDLFKTAAPMHDSINALQSN  611

Query  966   FAELGIAKESQVNQQQSRLNQWINKDKEENGEFSRAPGSSSKPVATSPNMAPLGLTQPDG  1025
             FA+LGI  + QVNQ QSRLNQWINKDKEE GEFSRAPGSSSKP+ATSPNM PLGLTQPDG
Sbjct  612   FADLGI--KDQVNQSQSRLNQWINKDKEEGGEFSRAPGSSSKPLATSPNMNPLGLTQPDG  669

Query  1026  PWSTGRTGDTGWPDSSGGDSSND-KDAQWATTAQPSLTDLVPEFEPGKPWKGNQIK-IED  1083
             PWS+GRTGD GWP+S GGDSSND KDAQW T  QPSL+DLVPEFEPGKPWKGNQIK IED
Sbjct  670   PWSSGRTGDGGWPESGGGDSSNDGKDAQWPTPTQPSLSDLVPEFEPGKPWKGNQIKSIED  729

Query  1084  DPSITPGSVVRSPLSIATIKDNELFSMN-TSKSPPVTDAMQSLSLSSSTWSFNPPSTSSA  1142
             DPSITPGSVVRS LSIATIKD ELF MN  +KSPP  D +Q LSLSSSTWSFNPPS++S+
Sbjct  730   DPSITPGSVVRSSLSIATIKDTELFQMNPNNKSPPAGDTIQPLSLSSSTWSFNPPSSTSS  789

Query  1143  FTSSPQIKLPTTKGGLGDLNPSMAITSELWGAPKSRGPPPGLSGKGGGGGGGGGGGAPLA  1202
               +SPQ KLP++K GLG+LNP+ A+TSELW APKSRGPPPGLS KGG           L
Sbjct  790   AFTSPQNKLPSSKSGLGELNPTTAVTSELWAAPKSRGPPPGLSAKGGA----------LV  839

Query  1203  NGWSSNIAGSVPWGGASGSAAQRNSGNWGVSQSQWLLLRNLTAQIDGSTLRTLCMQHGPL  1262
             NGWSS    +  WGG       QR SG+WG   S WLLLRNLTAQIDGSTLRTLCMQHGPL
Sbjct  840   NGWSS----AASWGG-----GQRGSGSWG--GSPWLLLRNLTAQIDGSTLRTLCMQHGPL  888

Query  1263  QSFHLHLHQGFALAKYSSREEATKAQTALNNCVLGNTSILAENPTDWDASTLLQSIANQQ  1322
             QSFHL+LHQGFALAKYS+REEATKAQTALNNCVLGNT+ILAENP++WDA+ LLQ +A+QQ
Sbjct  889   QSFHLYLHQGFALAKYSTREEATKAQTALNNCVLGNTTILAENPSEWDANALLQQVASQQ  948

Query  1323  GASSGGWRASSSKPGVAAGDTWSTGWPNNPSGVGLWATTSLDTNDPARATPASLNSFLPN  1382
              +SSG WR S+ +P   + DTWSTGW N+ S   LW +T+LDT DPARATP+SLNSFLP
Sbjct  949   -SSSGAWRGSTKQPSTGS-DTWSTGWSNSQSSASLWGSTTLDTTDPARATPSSLNSFLPG  1006

Query  1383  DLLGGESM  1390
             DLLGGESM
Sbjct  1007  DLLGGESM  1014
```

Graphical representation

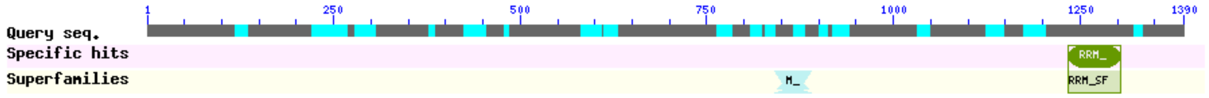

Similar to fragile X mental retardation syndrome-related protein 1

>Cb.comp32338_c0_seq1 len=1738

cDNA

GTCTTCGCCGCCGCCGCCGCCGCCTCCAGCTCCCCTGCGCATATCGCCTCGGCCCCTGCCCCCGCCTCGCCCACCCCTGTAGCCGCCGTATCTT
CGCGGGCCGCCGTCCTGAATACGTTCGTCGACGGTATCCGGTGTTTGATGACGGGATCCCGAATTGTAGCGGTCATTCTGTCGGGGACCGCCTC
CGCCCCGCCCGCGGCCGCCGCCGCGGCCCCTCATTGAGCCGCCCCTGCCTGAACTGCGGCCGTCCATGTCCGAACTGTAGCCGCGGTCGTTCCG
TCGCCCACTCATCGACAGACTCTGCATTGAGCCCAGGTTCGAGCCGTGGTGGATGCTGCGCAGTTGCTGATCGATCTCCAGCTTTTCTTGCCTG
AGTTGCTCAACTTCCTTGAGGTGCGCCAGGTGATACTGCAGCAGCACTTTGGCATTGGATATACTCTCGACGGTACCGACAAACACGAACGGCA
CCTGGCCCTCCTCCCTCGGGTACGTGGGTTGCGGCTCGTTGTCCCCTTCAATTTTCACCCTCACCACTCCACTCTTGTCGACGATCTCCTGTAT
GATGCGCCCGTTCTTGCCTATAACTTTGCCGACGAGCGTGCGCGGCACCTGTAACGACTCCTCGCTGTATTCCAGCAGCGAGCGCGCCTTCTTC
ACCGCCTCGTCGGTCTCCCCGTAAATTTTGAACGTGCACGAGTTCTCCTCCAGCTCGATGTTGGTAACGCCCTCGACCTTGCGCGCCTGCTGTA
TGTTTACGCCGTGCGCGCCGATCGCCAGCCCCATCAGGTCCTCTTTGACGTGGAACTCGTCCGAGTAGCCGCCGATCGTCGCCAGCTTGGTACT
CTCCAGCTGACGCGCCGCCTCCTCCGTCCTTTTCATCAGCAACACCTTCTGCTGCAGGCTACGAAAGTGCATGTCCTGCAGTAACGACGCCCGC
TTCCGGCTGGTCTCGCATCTCGATATCGCGACTAGGACGCCCTTATCGGGGTTGTAGAAAATGCGTCCCGCGCCTATCGCCTTCTGGAACTCCT
TGTGCGTGTCCTTCTTGCAGTAGTCTCTAACATCTTCGGGAACTTCGATTTCAAACTTGTGGAACATGCTGCTCTCTATGGGAGGATTGTTGTT
CTTCATCCTGAGGCGGTCGTTGCTAACAATTTCCGTGTAGGTACTGTCCCAGCCCAAGTACTCAAGGACGTAAAACTCGCCCTTCATCATCTTA
ATCCTGGCCTTCCACCAGCCATAGCCTTCTTGATTATTTGATCTGGAGAACACCTCCACTTCCATATTCTCTGTGAACTCCACTTTCTGATCTG
CATTAGGGGGTAACCTAACCTGTTCGAAGGGAAACTTCGAATCCGGCTGCCACTCGTCTTCAAAGTGAACCAGAACTCCATCATCGTGTACATC
CAGAATGGAGCCCTTATAAAGAGCCCCATTTTCGCCGACAACTTCTACAGCCAGGTCCTCCATGTTGGTTTGGTTCTTGGGTGAGGAAACAGCC
TTGAGCTGCTGAGAGCGAGTCGCAATGCCGTTCTCCATAAACATCATCATCGTATAAAATACACAAGTGCCTGTGACAGGTCGCTGTTAAACAG
TAGAATGTTTTGTCAGTACTCTAAAGCCAACTAAGTTGTCATTCTAATTGGAAAGTTGTTTGAATTGTTTAAACTTGTGGTGACAGTGTTATAA
GCATCTAAACTCCAAATATTTGTAGAAAACCAAGAAAACCTTCAGA

Protein RF -2: -1554->-1 (518AA)

MMMFMENGIATRSQQLKAVSSPKNQTNMEDLAVEVVGENGALYKGSILDVHDDGVLVHFEDEWQPDSKFPFEQVRLPPNADQKVEFTENMEVEV
FSRSNNQEGYGWWKARIKMMKGEFYVLEYLGWDSTYTEIVSNDRLRMKNNNPPIESSMFHKFEIEVPEDVRDYCKKDTHKEFQKAIGAGRIFYN
PDKGVLVAISRCETSRKRASLLQDMHFRSLQQKVLLMKRTEEAARQLESTKLATIGGYSDEFHVKEDLMGLAIGAHGVNIQQARKVEGVTNIEL
EENSCTFKIYGETDEAVKKARSLLEYSEESLQVPRTLVGKVIGKNGRIIQEIVDKSGVVRVKIEGDNEPQPTYPREEGQVPFVFVGTVESISNA
KVLLQYHLAHLKEVEQLRQEKLEIDQQLRSIHHGSNLGSMQSLSMSGRRNDRGYSSDMDGRSSGRGGSMRGRGGGRGRGGGGPRQNDRYNSGSR
HQTPDTVDERIQDGGPRRYGGYRGGRGGGRGRGDMRRGAGGGGGGGED

Comparison with *Tribolium* PREDICTED: similar to fragile X mental retardation syndrome-related protein 1, putative (660AA)

```
Query  28   MEDLAVEVVGENGALYKGSILDVHDDGVLVHFEDEWQPDSKFPFEQVRLPPNADQKVEFT  87
            MEDLAVEV GENGALYKG ++DV +D VL+HFEDEWQPDSKFPF QVRLPP  D KVEFT
Sbjct  1    MEDLAVEVCGENGALYKGYVVDVFEDSVLIHFEDEWQPDSKFPFSQVRLPPKPDPKVEFT  60

Query  88   ENMEVEVFSRSNNQEGYGWWKARIKMMKGEFYVLEYLGWDSTYTEIVSNDRLRMKNNNPP  147
            ENMEVEV+SR+N+QE YGWWK+RIKMMKG+FYVLEY+GWD+TYTEIVS+DRLR+KN+NPP
Sbjct  61   ENMEVEVYSRANHQEAYGWWKSRIKMMKGDFYVLEYVGWDTTYTEIVSDDRLRVKNSNPP  120

Query  148  IESSMFHKFEIEVPEDVRDYCK-KDTHKEFQKAIGAGRIFYNPDKGVLVAISRCETSRKR  206
            I+SSMF KFEIEVPEDVR+Y K ++ HKEFQ AIGA  I Y P+KGVLV ISR E+SR+
Sbjct  121  IDSSMFVKFEIEVPEDVREYAKIENAHKEFQNAIGASLIRYVPEKGVLVVISRNESSRRC  180

Query  207  ASLLQDMHFRSLQQKVLLMKRTEEAARQLESTKLATIGGYSDEFHVKEDLMGLAIGAHGV  266
            A L+QDMHFRSL QKVLL+KRTEEAARQLESTKLATIGG+SDEF+V+EDLMGLAIGAHG
Sbjct  181  ARLVQDMHFRSLSQKVLLLKRTEEAARQLESTKLATIGGFSDEFNVREDLMGLAIGAHGA  240

Query  267  NIQQARKVEGVTNIELEENSCTFKIYGETDEAVKKARSLLEYSEESLQVPRTLVGKVIGK  326
            NIQQARKV+G+TNIELEENSCTFKIYGETDEAVKKARS+LEYSEESLQVPR LVGKVIGK
Sbjct  241  NIQQARKVDGITNIELEENSCTFKIYGETDEAVKKARSMLEYSEESLQVPRALVGKVIGK  300

Query  327  NGRIIQEIVDKSGVVRVKIEGDNEPQPTYPREEGQVPFVFVGTVESISNAKVLLQYHLAH  386
            NGRIIQEIVDKSGVVRVKIEGDNEPQPT PREEGQVPFVFVGTVESISNAKVLL+YHLAH
Sbjct  301  NGRIIQEIVDKSGVVRVKIEGDNEPQPTIPREEGQVPFVFVGTVESISNAKVLLKYHLAH  360

Query  387  LKEVEQLRQEKLEIDQQLRSIHHGSNLGSMQSLSMSGRRNDRGYSSDMD-GRSSGRGGSM  445
            LKEVEQLRQEKLEIDQQLRSI HG+ LGSMQSLSMS RRNDRGY+SDMD G   GRG
Sbjct  361  LKEVEQLRQEKLEIDQQLRSI-HGNALGSMQSLSMS-RRNDRGYNSDMDGGGRPGRGSMR  418

Query  446  RGRGGGRGRGGGGPRQNDRYNSGSRHQTP--DTVDER  480
               G GRG GG G RQNDRYNSG+   T   + VD+R
Sbjct  419  GRGGRGRGGGGPGGRQNDRYNSGTSTITDYVNNVDKR  455
```

Graphical representation

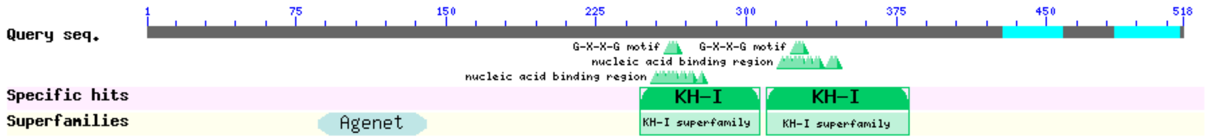

Maelstrom

>Cb.comp40771_c0_seq3 len=1873

cDNA

```
GCTAAACTAGAAAAAAAACCGGGATTCTCTTTGGTCAGTTCCCGCCGAGCAGATATATTATAACTAGTAATATATTTGCCGCCGAGCATATTTT
CATCCTATATAAGCTAACATCAAAAACTAGAGACAAAAAAAAAATTGCGATTCCTAGAAGTAATCTCTTTCGGCAAAAATAAACAATCTGCGAG
TTTAGCGAACACTTTTATCGCGTCAGTAGCCGATTAACAAATCGACAATGGCTCCGAAAAAACCCGCGGCCCCAAACGCCTTTAGCCTATTTGT
TATGGATTTCAAAAATCAACAAGGTAGGACATTCAATTCCACCAAGGAGGCGTACGAAGCCGCGGGTCCGGTATGGGCGAGGATGGGCGCCGCA
GATAGACGCCCTTATCAAGAAAGAGCTAAACTAGAAAAAAAACCGGGTAGATACACCTCCGAGGGGGTAAACGTCGAGGACTTGAAGCGAAAGG
AGCAAGAGGAAGCCGAATTTCAAGAAAAAATGAGACAGGACATAAGGCAGACGATCGATCTGGCTAACATGAAAGGACCTGAAGCGCTAGCGAA
CGAAACGTTTTTTATCATCCACTTCAATATCTTTTGCTTCCACATGCCCGCCAACCAATACTACCCGGCCGAAGTGGCTGCTGTGCGTTTTAAC
TTGAAGGACGGCGTAAAGCCGGAAAATGTATTCCACGAATTTATTTTGCCCGGTCCGTTACCTCTTGGATATTCGTTCGAAGCGAAACAACATT
CGGACGAAACACATCAAATCACAGTCCCTTACGATGACGTCGAAAACAACATGGGGGAGGTTTTCGCGAAACTCGTCACGTTTTTAGAGAAAAA
GAAAGCGGCTGGCGTGAGCCGAATGCCGATACTCTACGCTAACGAAAAGTACCAGAAAATGTTCCAGAACATTTTGGATCGTTGGAGTTGGGAC
TACGGCGGCGAGGAAACCATGTTCCGAGTCTACAGTTTGCAGGTCCTGTTTTTCTGGTTACGGAACAGGGTGTCCGGCGGGGAAGTGTGGCAGA
CTTATACTTTCAGCGATCGCGAGATCGAGAAAGATGTTTATGCTTACGTTCCCGATATCGCTTGTGATTATCACAGCGCCATGCCGAAACCGTT
GTACTGCAGCAAGTCGACGGTTTTGCGCATGGTCTACATCATATGCGACAACTGCTCCGAAGAATTGGACATAGAACTGTTGCCGGGGCAACAC
GTGCCCTACAGGGCCGTTGTGCCATCCGGAGCCTCGAGCGTCAAAAGTTTTAATACCCGCAGCAGCCGCACCGCCCCTTGGGGTCTGGGTACCG
AAGACGACGACGACTCGACCACGGACTGGGAGAGCAAGTCTTTGATATCGGAATCTTCCGTTACGACAGTCGATTTTCCACCATTGAGAGCACG
CAGCGGCAACGAGGACCGTTCGGATCAGTTTCCGAGCCTGCCTCAAAGTAACTCGTCGACCGCTCGGAATTATTCGGATCCGTTCGGGGTGGAT
GCTTTGCCGTCGGCCTTTGTGAATGTCCGCGGCAAGGGTAGGGGTTTCCGTAAAAGGGACGACGATAGCAGCAGTGTGGCGTCTTCGGTGGTGG
GTAGGGGCGAATCGAATGTGGCGCCGAGAGGCAGGGGTATGCGATTCAAGGCTGTGACGAAACCTGGACCGGTTAATTAGAGTGAGCGTGGGAA
GTGCCTATAATAGAATCTTAAGAAATTTTGTTGTTTCCAGAGTCCCCATATATATATATTTATTTCGTTTTTTTTAGATGATAGAAGACTTACTT
TACTTAAGTTTCATTTACTTTACCATTTAAGTTGCCAAAGGTCTGTTTATACCGTTTTAATAAAGGTTACTATTTTTTCAAAAAAAA
```

Protein RF 2: 236->1678 (480AA)

```
MAPKKPAAPNAFSLFVMDFKNQQGRTFNSTKEAYEAAGPVWARMGAADRRPYQERAKLEKKPGRYTSEGVNVEDLKRKEQEEAEFQEKMRQDIR
QTIDLANMKGPEALANETFFIIHFNIFCFHMPANQYYPAEVAAVRFNLKDGVKPENVFHEFILPGPLPLGYSFEAKQHSDETHQITVPYDDVEN
NMGEVFAKLVTFLEKKKAAGVSRMPILYANEKYQKMFQNILDRWSWDYGGEETMFRVYSLQVLFFWLRNRVSGGEVWQTYTFSDREIEKDVYAY
VPDIACDYHSAMPKPLYCSKSTVLRMVYIICDNCSEELDIELLPGQHVPYRAVVPSGASSVKSFNTRSSRTAPWGLGTEDDDDSTTDWESKSLI
SESSVTTVDFPPLRARSGNEDRSDQFPSLPQSNSSTARNYSDPFGVDALPSAFVNVRGKGRGFRKRDDDSSSVASSVVGRGESNVAPRGRGMRF
KAVTKPGPVN
```

Comparison with *Tribolium* maelstrom(480AA)

```
Query  15   FVMDFKNQQGRTFNSTKEAYEAAGPVWARMGAADRRPYQERAKLEKK---PGRYTSEGVN  71
            FV+D +N+      N    E  E A    WA M    +RRPY+ERA L ++    P RYT++G++
Sbjct  17   FVLDCRNKHPNKQN-MHEVQEYAARKWASMSKEERRPYEERALLAREMYSPARYTTDGID  75

Query  72   VEDLKRKEQEEAEFQEKMRQDIRQTIDLANMKGPEALANETFFIIHFNIFCFHMPANQYY  131
            +E ++RKE++EA   +++M+ DI +T+   A         L   + F +IH N    ++  ++Y+
Sbjct  76   IEVVERKERDEARKKQEMKDDITRTLKAAYFATD--LDEKIFLVIHINHLAYYPTEDKYF  133

Query  132  PAEVAAVRFNLKDGVKPENVFHEFILPGPLPLGYSFEAKQHSDETHQITVPYDD--VENN  189
              E+A      +LK+GV   E+VFH   + PG LPLGY    A  HS ETHQ+       D    ENN
Sbjct  134  ICEIAIAAVSLKNGV--EDVFHRIVKPGKLPLGYYGGALTHSKETHQMLELVQDEPYENN  191

Query  190  MGEVFAKLVTFLEKKKAAGVSRMPILYANEKYQKMFQNILDRWSWDYGGEETMFRVYSLQ  249
              EVF  ++ +FL+    +   G       I+YA+EK   +M     ++D  +    ++      + + +VY+ Q
Sbjct  192  TREVFNEMTSFLKLWRGKGSD--SIVYADEKTHEMITKVIDNFCQEFNYPDEI-KVYNFQ  248

Query  250  VLFFWLRNRVSGGEVWQTYTFSDREIEKDVYAYVPDIACDYHSAMPKPLYCSKSTVLRMV  309
```

```
               LFF LRN V+    VW T T+S  E+EKD+Y+Y PDI+C++H      +YCSKS V R
Sbjct  249   YLFFALRNSVAARTVWPTETYSSTELEKDLYSYTPDISCEFHEMSDISVYCSKSIVTRYC  308

Query  310   YIICDNCSEELDIELLPGQHVPYRAVVPSGASSVKSFNTRSSRTAPWGLGTED  362
             Y +CD+C   +L+I+L+ G HVP  + +   +S        R++  AP    T+D
Sbjct  309   YTLCDHCCTDLNIQLVAGFHVPKNSRIAVDSS-------RTNSKAPSVCSTDD  354
```

Graphical representation

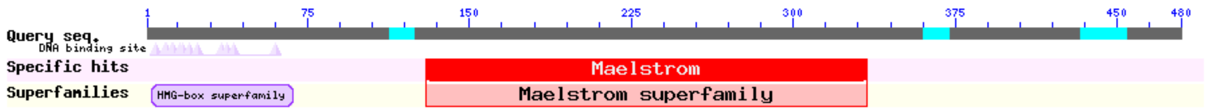

Tudor-SN

>Cb.comp39931_c0_seq1 len=3113

cDNA

```
TTTTTTTTTTAAATATTGGTCAATTTAATTATTTTCTATCTTATATAAATGTCAATATGAGTGAATAAAGATAGATGATCCGTTCCAACTCCCG
GACAACGGTGTCCCATTCCCTGTACATTTAGGTACGTGCTCTGGCTCGTGTTTGCTGCCTGGACGACCGATCGTTAATTGCCCAGGCCGAATTC
CTTGGCGTCGTCTTCTGTAATGTCGCCATACTCCCATATGTTTAAATGCTCCTTTTTCGCTTGATCTTGAGCCTGTCTGTAATCTTCAAGCAGC
TTGTTTTGCCTTCTCGATTTGACCTTGTCGACCATCAGCAAGCCCTCGGAGATCAAGCCGCGGAAAATATCGGACGCTCCGGACGTGTCCTTGT
GCAAGGAGGCGGCCGGAGGCAAGCCCTGATTCCTGTACTCCACGTTTAGGTGGAGTTTGCTTACGCTCGTGTCCTCTTTTAGGAATTTGATCGC
CATGTCCCGGAACTCTTCGTCTTTCGGTAACGATACGTAAGGCATGACGTATTCCGAAGCGAAAGGTTTGTCCGCGGTGTACGCGGCCGGTAAA
CTGGCCAATCGCGTGGTTGGCAACACTTCACGATTACCGTAATCTATGTAGTGTACGGTAGCTTTACCTCCGGTCACCTTTTCCACTTTCACCC
TGTACCACTCGTTGTCGACGCTGAACTGCGCGGCGCACACGTCCCCTTTCCTCGGATTATAAGCGCCGGGCAACGGCGGATTCGCCTCGAACTC
CTGCCTAAGTTTCGCGCACAGCGCGTCGAGCTTCGGCCCGTCGGCGAACCGCTGCACGAAGAAACCGCCTTCCGGTGTGATTTCCGTCACGACC
ACTTCCTCGAAGTTAACCTTTCGCTCGACGTTCACCCGTTCTTCCTCCACTTTCTCTTTCACTTCCTCCTCGACGTAATTCTTCCACCGTCGCA
ACTTTTGCGCTTTCGCGCTCTCCTCCGCCTGTTTCAGCTGGGACGCGTACGCGGACTTTTCGCCGGTAAAATGGACGCTCGCGAAACCTTCCTT
GACGAGCGACACTGACAAATTGACGTTGTCGATCCACAACCATCCGATAAAGTTGCCGGCCTTGTCGTGCGTGTCGACTTGGATCGACACCTCG
CGTTGCAAGCAACGCTCTTTCGAGAACTGAAGCGCTTCGTCGCCGAACTCTTCGCCTTCGGATGCCGGAAGGGTCCCGGTCGCCGGCCGGCTCG
CCCTAGGGCAGTTTATGCCGCCTAACAAAAATGTGCAGAGGCTATTGGATTTCGGAATGTACAGTCTGAATCGCGATCCGCTGGCCACAAACTC
CACGATGGCGTCCAAACGTTGAGCGCGCTGGAACGTGGCGAGCTCCAGTTTGGCTCGGGCGGCGTCGATCTCGGTGACCCGCAGAGGGGCGCTG
TCTTTCTTCGAATGCAAACCGAGCTGGGACTTTTCCGCTTTCGACTCTGCCTTAACGAGCTCGTCGTATCTACCGCTCCTCTGGTCGTCGTCTT
GCCTGTACCTTACGACCGTCGCGAGTCCTTTCGAGACGAGCGCCTCCGCAACGTTCTTGCTGTTGGAGAGAACGGTCGCGCAGACTTTTTCCGG
GAACCCGTCCCTCGCCTCTTGGACGTAGTCGATAATCACCTGCACTTTCTTCCCGATCAGCTTCTTCCTGAGGTATTCCCTGGCCTCGAACATC
CACGGGATGTCGTATAGGGGTCTGAAACCTTTCGGCCGAGGCAACGGTTTTCCTTCGTCGTCGTTCGCTTTGCCCGTTTCTCTGGGCGGTCTTA
TGCTCGCGAGGAATATTTTTTTAGACGTCCCGTTGGCCAGTTTCACTTGCAAAGCGTCGCCGTTCACCACTTCCACCACCGTCGCGGTAAACTC
TTTCTCTTTGCCAGTTATCTGGGGCGTGGTCGATTGCCAACCTTTCCAGAGGCGTTTCTGTTCGTTCTTCGCCTGACGTTCCGCGGCCCTCAGT
TTTTCCACGACGTCAGAGGGAAGCGGCGCCAAGGACCAGTCCACGCACTTCGCGAAACCCTCTCTGACGAGGTTCTCAGCGATGTTTCCTTTTG
GATGCAGAATGGTTCCCACGACGTTGTTGTTGGTGTTGTTGACCGAATGCAAGACGATCTTGACGTCACGTTGCAACAACCGCACCTCGACGTA
ATATCGAGCCTCTTCGGCATATTCCACCTTCTGGTTGGGATCGGGCCTGCCCTGGCTGTCCAATTTAAAACCGGGACACCTTATCCCGGCCACC
ATCATGGTTACATAATAGAAGTCGGGTAGCAGGAACGCCCTGACCGTGGACCCGTCTCGGACATGCTCGATGATGGCGTCGAACTGTTTGTTCT
CGTTTTTCTCCACGAAAGCCCTCATGTTGTCGATGCTCCATTTGATGTCGCGCACGTGCTCCGACGAAGGGGCGCCGCCCCATTTGCCTTTCCC
CGCCGATTTGGCCGCGTCCTCTAGTTCCTGCAGACGGGTTAGTTCGGGAGTGGGTCTCACCCCTTCCCGTCTTACCGAAGCCAAACCTTCGGAC
ACCAGCGATTCCGTTATATTTTCGCCGCTGTTGATGTCTTTTCCCAAGTAAATGGTCCCGTATTCTCTGTTAGCGTTCGCCGGTTTTTCCGATA
CGAAAATGACCTCCTCCCCGATGAGTTTTTTTCGAAGGAACTCTCTCGCTTCCCACGCCCATGGTTCGTCTTTACTTTCGCTCCCGGCAGCGTT
CGAGTCCCCGGGCCGCCTCGCCAACTTGGGCGCCGTGACGCCCGAAAAATTTATTTGTTTTTCGGGGGGTGGCGCGCCCGCTGATGCCCTAATG
ATCACGGAGTCCCCGGAGAGGATCTGCTTAACGATCCCACGTTTGGCCGAAATCGGAGTTTGCTGCGGTGTCGTCATCGTGTAGCCATTAACTT
GCGAGAGAGTTGTCGGTGTGTTTTCGTCTAAACTGTCTAAATTCGTGGAGCGACGTTAGAAAAAGACAGCGGTAATCAAATTCGTGTTCGGAAT
GTTTTCGGGGTTAGCGGCGTCTCAGTCACAGTCGCAACATTAGAGGGAGATGGACTGTGGACGGATCCGGCCTGGCCGGGTCGGAACTCGCGGC
GAACGAGCGAG
```

Protein RF -1: -2897->-168 (909AA)

```
MTTPQQTPISAKRGIVKQILSGDSVIIRASAGAPPPEKQINFSGVTAPKLARRPGDSNAAGSESKDEPWAWEAREFLRKKLIGEEVIFVSEKPA
NANREYGTIYLGKDINSGENITESLVSEGLASVRREGVRPTPELTRLQELEDAAKSAGKGKWGGAPSSEHVRDIKWSIDNMRAFVEKNENKQFD
AIIEHVRDGSTVRAFLLPDFYYVTMMVAGIRCPGFKLDSQGRPDPNQKVEYAEEARYYVEVRLLQRDVKIVLHSVNNTNNNVVGTILHPKGNIA
```

ENLVREGFAKCVDWSLAPLPSDVVEKLRAAERQAKNEQKRLWKGWQSTTPQITGKEKEFTATVVEVVNGDALQVKLANGTSKKIFLASIRPPRE
TGKANDDEGKPLPRPKGFRPLYDIPWMFEAREYLRKKLIGKKVQVIIDYVQEARDGFPEKVCATVLSNSKNVAEALVSKGLATVVRYRQDDDQR
SGRYDELVKAESKAEKSQLGLHSKKDSAPLRVTEIDAARAKLELATFQRAQRLDAIVEFVASGSRFRLYIPKSNSLCTFLLGGINCPRASRPAT
GTLPASEGEEFGDEALQFSKERCLQREVSIQVDTHDKAGNFIGWLWIDNVNLSVSLVKEGFASVHFTGEKSAYASQLKQAEESAKAQKLRRWKN
YVEEEVKEKVEEERVNVERKVNFEEVVVTEITPEGGFFVQRFADGPKLDALCAKLRQEFEANPPLPGAYNPRKGDVCAAQFSVDNEWYRVKVEK
VTGGKATVHYIDYGNREVLPTTRLASLPAAYTADKPFASEYVMPYVSLPKDEEFRDMAIKFLKEDTSVSKLHLNVEYRNQGLPPAASLHKDTSG
ASDIFRGLISEGLLMVDKVKSRRQNKLLEDYRQAQDQAKKEHLNIWEYGDITEDDAKEFGLGN

Comparison with *Tribolium* PREDICTED: similar to ebna2 binding protein P100 (1389AA)

```
Query  1    MTTPQQTPISAKRGIVKQILSGDSVIIRASAGAPPPEKQINFSGVTAPKLARRPGDSNAA  60
            MTT Q  P   KRGIVKQILSGDSVIIR   GAPPPEKQINFSG+ APKLARR GD +
Sbjct  1    MTTQQNQP---KRGIVKQILSGDSVIIRGPTGAPPPEKQINFSGIVAPKLARRAGDQS--  55

Query  61   GSESKDEPWAWEAREFLRKKLIGEEVIFVSEKPANANREYGTIYLGKDINSGENITESLV  120
               +KDEPWAWEAREFLRKKLIGEEV F SEKP NANREYGT+YLGKD NS ENITESLV
Sbjct  56   -EPTKDEPWAWEAREFLRKKLIGEEVFFTSEKPPNANREYGTVYLGKDFNSAENITESLV  114

Query  121  SEGLASVRREGVRPTPELTRLQELEDAAKSAGKGKWGGAPSSEHVRDIKWSIDNMRAFVE  180
            SEGL +VRREGVR +PE  RL ELEDAAK+AGKGKWG +P SEHVRDIKWS++NMR+FV+
Sbjct  115  SEGLVTVRREGVRQSPEGARLAELEDAAKAAGKGKWGSSPPSEHVRDIKWSVENMRSFVD  174

Query  181  KNENKQFDAIIEHVRDGSTVRAFLLPDFYYVTMMVAGIRCPGFKLDSQGRPDPNQKVEYA  240
            K   K   AIIEHVRDGSTVRAFLLP+FY+VT+M++GIRCPGFKLD+ G+PDP+ KV YA
Sbjct  175  KLGYKPVKAIIEHVRDGSTVRAFLLPEFYHVTLMISGIRCPGFKLDANGKPDPSIKVPYA  234

Query  241  EEARYYVEVRLLQRDVKIVLHSVNNTNNNVVGTILHPKGNIAENLVREGFAKCVDWSLAP  300
            EEARY+VE+RLLQR+V IVL SVNN  NN VGTI+HPKGNIAE L++EGFA CVDWS+A
Sbjct  235  EEARYFVEIRLLQREVDIVLESVNN--NNFVGTIIHPKGNIAEALLKEGFAHCVDWSIAF  292

Query  301  LPSDVVEKLRAAERQAKNEQKRLWKGWQSTTPQITGKEKEFTATVVEVVNGDALQVKLAN  360
            + S V E LRAAE++AK  + R+WK WQS  PQ+TGKEKEF+ATV EV+NGDAL VKL N
Sbjct  293  MKSGV-EGLRAAEKKAKMARLRIWKDWQSNAPQVTGKEKEFSATVAEVINGDALSVKLNN  351

Query  361  GTSKKIFLASIRPPRETGKANDDEGKPLPRPKGFRPLYDIPWMFEAREYLRKKLIGKKVQ  420
            G  KKIFL+SIRPP+E G+  D++GK  PRPKGFRPLYDIPWMFEAREYLRKKLIGKKV
Sbjct  352  GQYKKIFLSSIRPPKEPGRVADEDGKTAPRPKGFRPLYDIPWMFEAREYLRKKLIGKKVH  411

Query  421  VIIDYVQEARDGFPEKVCATVLSNSKNVAEALVSKGLATVVRYRQDDDQRSGRYDELVKA  480
            V+IDY+QEARDG+PEKVCATV    KNVAEALV+KGLA+VV+YR DDDQRS +YD+L+ A
Sbjct  412  VVIDYIQEARDGYPEKVCATVTVGGKNVAEALVAKGLASVVKYRPDDDQRSSKYDDLLAA  471

Query  481  ESKAEKSQLGLHSKKDSAPLRVTEIDAARAKLELATFQRAQRLDAIVEFVASGSRFRLYI  540
            ESKA KS +G+H+KKD    RVTEIDAARAKLEL++FQRAQR+DA+VEFVASG+R R++I
Sbjct  472  ESKAMKSGIGIHNKKDVPIHRVTEIDAARAKLELSSFQRAQRIDAVVEFVASGTRLRVFI  531

Query  541  PKSNSLCTFLLGGINCPRASRPATGTLPASEGEEFGDEALQFSKERCLQREVSIQVDTHD  600
            PKSNSLCTFLLGGINCPRASR AT   PA EGE FGDEALQF+KE+CLQREVSIQVDTHD
Sbjct  532  PKSNSLCTFLLGGINCPRASRQATNAQPAVEGEPFGDEALQFTKEKCLQREVSIQVDTHD  591

Query  601  KAGNFIGWLWIDNVNLSVSLVKEGFASVHFTGEKSAYASQLKQAEESAKAQKLRRWKNYV  660
            KAGNFIGWLWIDNVNLSV+LVKEGFASVH TGEKS YA+ LK+AE+SAK  +LR WKNY
Sbjct  592  KAGNFIGWLWIDNVNLSVALVKEGFASVHRTGEKSQYAALLKEAEDSAKQHRLRIWKNYE  651

Query  661  EEEVKEKVEEERVNVERKVNFEEVVVTEITPEGGFFVQRFADGPKLDALCAKLRQEFEAN  720
            EE+ +   EEE+ NVERKV++EEVVVTE+TPEG FFVQ  ++GPK +AL AKLRQEF+AN
Sbjct  652  EEKEEPHAEEEKPNVERKVSYEEVVVTEVTPEGSFFVQTISEGPKAEALNAKLRQEFQAN  711

Query  721  PPLPGAYNPRKGDVCAAQFSVDNEWYRVKVEKVTGGKATVHYIDYGNREVLPTTRLASLP  780
            PPLPGAY P++GD+CAA+++VD+EWYRVKVEKV GGKA+VHYIDYGNRE LP+TRLASLP
Sbjct  712  PPLPGAYTPKRGDICAAKYTVDDEWYRVKVEKVQGGKASVHYIDYGNRETLPSTRLASLP  771

Query  781  AAYTADKPFASEYVMPYVSLPKDEEFRDMAIKFLKEDTSVSKLHLNVEYRNQGLPPAASL  840
            AAY  +KP+A+EY++PYV+LPKDEE+  MA+K+L+EDT+VSKL LNVEYR QG P AASL
Sbjct  772  AAYAGEKPYATEYILPYVTLPKDEEYAAMALKYLREDTAVSKLLLNVEYRVQGGPSAASL  831

Query  841  HKDTSGASDIFRGLISEGLLMVDKVKSRRQNKLLEDYRQAQDQAKKEHLNIWEYGDITED  900
            H D +    DI + LI+EGLL+V+  K RRQNKLL  Y++AQ+ AK+ H NIWEYGDITED
Sbjct  832  HTDNTAEGDIIKNLITEGLLLVENRKERRQNKLLGAYKEAQEVAKRNHSNIWEYGDITED  891

Query  901  DAKEFGLG  908
            DAKEFGLG
Sbjct  892  DAKEFGLG  899
```

Graphical representation

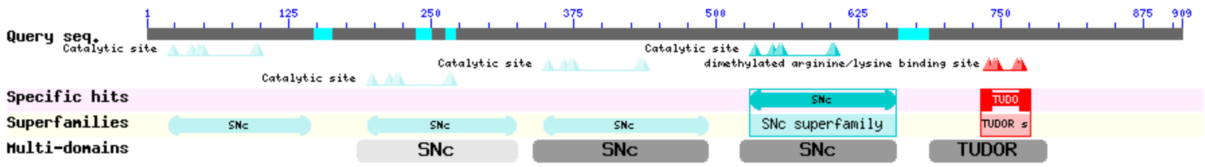

Elp-1

>Cb.comp42860_c1_seq1 len=5361

cDNA

```
TATTAACAGCTTTGGAAGTCGACGTTAATTCAGATACCATTTTCAGATAAAACGCACTTGTTTTTACCAAATTATTATGTTATTAAACTGCTTT
TGCATATATAAGTCCCAAACCACAATTTTACTAAACAAAAAGTATGTATGTTGCGTTTTGAGGATTACCTGCTAAACCAGTTTACATTTTTCGC
CACCAGAAGTCTCATTGATCGTTGGATTCGCCAACAAACGACAAACTTTTTTCCAGTTAATATTTATCCAATATAGCAAAATTGTACTAAGAAA
ACTGTTTTTCAATACTGTGAAAGATCGGAGTGCATGTGAGTGCTTTTCTTATAAGCGACTTTATAACAGAATAAAATGTAGAAATACTTTATCC
TGCTCTCTAATGAGGGAAGAATTAATAATTTAATTTACTAACACAGAACAGTTTCAAATTTATTTATGGCCAATAATTTAGGTGCCAGTTTTGC
ATTTCAATGATTTCAAAACATATTTTCCACTTTCACAAATCACAGAAAATATACATATATATAATTCTTGCATGCCTGTCATATGGTTGAGATA
AAAAGGAATGCATTTGATTGCAGGCCTTTGATAAGGGCGGGAGTTTATAAAAATGCTGGCGTTAACTTCGGAACTTGATTTTCTGTATAGTATC
AAGGACTTGGTCTATTAAAAATTAGTGTATCACAGATATCCTTAATATTGATAATTATAAATATATTCCTGATATTATTGAGTATGTGACTGAT
TTTGTAATGCGAAAAATTGCAAGAATATTAAACTCTACTTTAATAAATCAAAAACTAGAGGAAACTTCACTACAGGTCATCCCGATGACATAAT
AATATGAAAAAAAATAAAAAAAAGAAAAAGCATTTTCAACTATGGAATTTAGTACTGGCACTAATATAAAAAAAAAAAAAGTTAACACGATAAA
AACAATCACTTTTTAAAAAGTTATAAAAGATTATCTGCATGAAATCACATTTTAATATGTTAAGTAAGGGCTAAGTACTAAGATTTCAATCTAA
ATTTTCTACTTTTAATTTTAAAATTATAACGAAAGTTTTTCTCTATAATAAGGATTTAGGATTGCACTACCTTTCTGCCAGAAAGTAGTGTCCC
AAAGTAAACAGACCACCAAATTCTATATAAATAAATAAATTGTATGTTTTTTTTAAGGTACTGGTACTTCTGAAAGGAATACATACTCTTATTC
TCTGACCAATAAATGATTAATATTTTGAATTTCCAAATGTATCAAACAATTTTATTTGATTTTTGGAGGTTTTTTAAGAAGCTAGATTATTATA
AATGATAAATGTTTTCAGCATGAATATAATCCGTATATATATGTTCAGTTTTATATAATTTAAGTAATATTAGCCAATAGTGGCAACGCCGCAC
GACACTACCGAAAAGGGGGTTTTGCGGGATGAAAAGAGAGCCGGTTGAGAGACGCGAATAAACTATACATTTTCTATGGAATAAACTGTCGTAA
AAATAGGTTGCAATACAAGGTGGGACTGACCACGAGAAATAAACATTTTTATTTGTTTTGTAGAATAAGTCCAATCTGTTTTTCACACTTTGTT
CGATGCAAATTAAATGCATTAGTAGTTCGCGTGGGTGTTATTTCAACATCAAATAAGTGAGAAAACCAATATAAATGAGTTAAAGAGAGAGCTT
TTCCAGTTAGGGGGAGTTCACGAGACTCAAAAGACACCTGAAAGTTGTGATAAGTAATAGTAAAGTTTTTAAATTTTTGCAAAATTCAAAATTT
AGAATTTTACTGAAATGTTAATAATTTCCAGTTTTCATTAAATTCCATCAGAAGTGGTGGAGTACGAAATTCTATATCCAATTGTTCTCTATTT
TGTACAACAGCTAATATTTCGGGCTCTGCAGACTGAGCTGCATTAACAAATGTATTAGACCATATTTCTGGAATAGTTGATATAACTTTCTTTT
GGACTTCGCTAAGATTTTTATGTAACATTGCACTATACGCAAAGGACGAATCATCCGTTAACGTTTGGCAAATTTCACGAACCTCTTGGCCACA
TTTAAATATACTGGCAGTTAACAGATATAAGTGCCTTATCAATGCAATATCCTCAAACACGCTTCCTTCACGCAGATCGGTCTTTTTCTTATTA
AGTTTCCTTCTGTTTTTCGCTGAAGTTGTTGACATTTTTGATCTGCTGGATCCTAAAGTAGAACCCTGGGAGGATATTAAGCTTCCATACTCTG
AATAAAGGTCGCATTCTCCGAACGAGTCATATACTTCGCTTCTATACTTGTTTCTCAAATGCAGTAACTTATTGTGGCGAACTGTTTGCAAACG
TTCAGAACATATTAAAAATTCCTTTCCGAGATCTGAAATTCTACTGTACAATTCATTTCTGTATTTGATTATAGCCGGTCGAATATGCTTATCA
ACTAAATCCAAACAATTATACTTCTTTGCCATACACAATGCTTTCTGAAACAACTTTTTGTCCAACAAAATATATATTGCACTCAAGTGATCTT
CACAATGCATCTCATACAATACAACCGCTTCATCAATTCTATTCTGTTTGACCAAACCTTCTGCCAATTCTGATATAATTTTTTTACTCTTGGA
TGTTTCAACATTAAGTTGTACTAATAAGCTGATAACTTGATACCAATTAAGAGACTTTTTATATTCGGTTATTGCATCATTGTATAATTCTGCT
CTTTGCAGTATTAAGCCAGCTTCATCATACCTCAGTTTTGTTGACAAGTAAGTAGAATAAAGTTGGGAACATAAAATGAAATTTGAATTTTTCC
TAGTTACACTTTTATAGGCATCTTCATATACGTCATGTTTTGCAATAAACTCCTTTATGAATTTTTCCTCATACTTTGGGCATCTAATTAGATA
TTTTACAGCTCCTTTATAATTTTTTGCGAATACATTCATCTGAAACCTCAAATCAACAGGTTCTATGTGTTTTAATGTCTTTAGTTGAGGCTCA
AATACCTTAGGATCCTGAGAACAACAAGTATGCACAAATTTCATGAATTCAATATCATATAAAGTGAAACTTGTGTTTATAATAGCATCACATT
GATGATACAACAAAATTTGAAATATGGCTTTCTTGCATATTTCTAAATGACCTAGTTTAAACACTATATGCACAGACTTGAGTGCCGATTTTAA
CGAGAAATGCCTTATTTGTAAAATAATGATGCTAATCAAATTAGTCACCAGATCTAAATTTATAATGCAGTTCAATATTTCTGACAATATCTTA
TGTTTATTATCAACTGTGTGTACTGGAAGAGTGCTATCTTTGACACACTGAGAGTACATGGTTTTGAGACAGTTGGTTGTACCCAGTTCAGACA
CAATAGAATTCAACAAAGACACTGATTCAGCTGCTCTTAGAAATTCTAATATGTGGTTGTCAAATCTTTGGGGATTTAAATCTATAAGAAGATT
CCAATTCAATTTATTGTGCCTAATTAAACTGATCGCATCCTCCCACTTATTATTAGATATCATTTTTTCTATATTGTCGATACATATTAGACGA
CACTTAATAGTCTCCAAATTTCCTCTAGGCAGTTCCATAATAATTTCGGTTTTTGCATTTACACAAACAATTTTTGCACCTTGTTCTATATCTC
TAGAGTAATAGTCATTCAAGTCCCAATTTGGTTTATCAACATTTTCAGATGTCAGTCTAACACAGTACAGTTTACAGGAGGCATGAGTAAAGAG
AAAGTAACGTTTGTATATAAAAATTGAAAATACTTTACTACAGACTACATTTTTGTTTATGAGTAAATCTTGACACTGATTTAAAGCAAATTCA
TAAAATCTATTATCTATTCTGCAACCTGTACAATATACTAGAGATGAATTGCCTCTTTCATTTAAATTTATTGGCTCTACTGCCTCTGTGACAT
CACTAAAGTGGAGATAATTTAATTTTGTAACCAAATCATCTACAGTATTCCCAACACAGAGTGGCATTTTTTTCAGCACATTATTTTCAAATTT
AATCACATTTATAATTAAAGCATTATCAATCAGAATACATTGCTCAATTTCAGGATGGAAATGAATCTTATTGATGGGCTTATCAAATTTTAAA
TAAGTATTGGACATGGGAGGGGGAACAATTTCAGACGAAAAAAATGTAAAATTAACAGTAGAGCCATCAATAACTGCCACAGTCCCGGTGGTAG
GACACCTATTTATCACCATTCTAAAAACAATATGCTCAACATTTTGTGCAGTTAGAATGTTCAATTTGCAAATCAAATTTTCCAAATTATCTGA
CCAGTGAAGACATTTTACTTGATTTTCAGCTTGATAGGAGAGCTGCTGTTTCAGATACCATTTTGCATTTAAATAGAGAAAGATATGAATATAG
CTATACCCAGATTCCTCAATGCTGCAGATAACCAATATTTGATGAAATGGATGATATAAAAGCTTATGAATATCACCCCTAACTTCTGGAATGA
AAAATTCTGATTTGAGCAAGCAGTTTTTCTCAAAAATGACAATTTTATTCTGATCATTGTGAACTGCGGCACACGCAATAAAATTTCCTTGACC
```

```
TTTAAATGATATTGGAGGTAACAGATTTGGATAATAATTAGATTGATACAAAGGCCTTAATGAGTTGTCAAAAAGTTTCAGATACCTTCTTCCT
TCTTTCCAAAAATTTATTACAAACTGTTCTCCATTTCCTCTCCAGGAAATTGTGGGTGGTGTATACTTTTGTGGAATTTCTCTTATTTCTACCT
GTCCTTTTTTTTTGTGATCCCTTGAATTGAGTATTGTAGGAACCCCATCCTACGTAGACAGAATTTGGTACAGGATCTTGCAAATGTGTTTTCC
AAGTTTCCATGAATTTGTATTCTTGCAAATCGATATTGCATTCAAATATAAGACCATGGATGGTTTCCTCATTATCACAAACTACTAAACAAGT
TTCGCTAGGATTCCATGCCACACTTTCAATTTTAACGGCAAAAGAATAAACAACAACAAACTCTTTTGATTTGGTTTTGTATATTATTAAATCG
AAATCATTAGTCAGGAACAAAACTTCGTGAACCGATAAGTATACAAGATTTCTTATATCTCTAATGTTTTCACCGATAGGGTTAGCATGGTGAA
TTTGTTTTTCATACAAGTACAAATGATTTTTGTCAGTGGAAAAAGAACTTGCACTGTCTTGCAAAGTACCTACATTTATATTTCCGGTTTCCTG
TTTATTAATTTCTTTGACTTCACGAAAGAGAACCTCGATATTATTCATTCTTAAACTTTAAAATTCGTGTCAAAATGTATAAAGTATTGGGTTA
ATA
```

Protein RF -3: -4842->-1793 (1015AA)

```
DGVPTILNSRDHKKKGQVEIREIPQKYTPPTISWRGNGEQFVINFWKEGRRYLKLFDNSLRPLYQSNYYPNLLPPISFKGQGNFIACAAVHNDQ
NIVIFEKNCLLKSEFFIPEVRGDIHKLLYHPFHQILVICSIEESGYSYIHIFLYLNAKWYLKQQLSYQAENQVKCLHWSDNLENLICKLNILTA
QNVEHIVFRMVINRCPTTGTVAVIDGSTVNFTFFSSEIVPPPMSNTYLKFDKPINKIHFHPEIEQCILIDNALIINVIKFENNVLKKMPLCVG
NTVDDLVTKLNYLHFSDVTEAVEPINLNERGNSSLVYCTGCRIDNRFYEFALNQCQDLLINKNVVCSKVFSIFIYKRYFLFTHASCKLYCVRLT
SENVDKPNWDLNDYYSRDIEQGAKIVCVNAKTEIIMELPRGNLETIKCRLICIDNIEKMISNNKWEDAISLIRHNKLNWNLLIDLNPQRFDNHI
LEFLRAAESVSLLNSIVSELGTTNCLKTMYSQCVKDSTLPVHTVDNKHKILSEILNCIINLDLVTNLISIIILQIRHFSLKSALKSVHIVFKLG
HLEICKKAIFQILLYHQCDAIINTSFTLYDIEFMKFVHTCCSQDPKVFEPQLKTLKHIEPVDLRFQMNVFAKNYKGAVKYLIRCPKYEEKFIKE
FIAKHDVYEDAYKSVTRKNSNFILCSQLYSTYLSTKLRYDEAGLILQRAELYNDAITEYKKSLNWYQVISLLVQLNVETSKSKKIISELAEGLV
KQNRIDEAVVLYEMHCEDHLSAIYILLDKKLFQKALCMAKKYNCLDLVDKHIRPAIIKYRNELYSRISDLGKEFLICSERLQTVRHNKLLHLRN
KYRSEVYDSFGECDLYSEYGSLISSQGSTLGSSRSKMSTTSAKNRRKLNKKKTDLREGSVFEDIALIRHLYLLTASIFKCGQEVREICQTLTDD
SSFAYSAMLHKNLSEVQKKVISTIPEIWSNTFVNAAQSAEPEILAVVQNREQLDIEFRTPPLLMEFNENWKLLTFQ
```

Comparison with *Tribolium* PREDICTED: similar to CG10535 CG10535-PA (1172AA)

```
Query  5      TILNSRDHKKKGQVEIREIPQKYTPPTISWRGNGEQFVINFWKEGRRYLKLFDNSLRPLY  64
              T     + K K Q  +   P     P ISWRGNGE FV+N+WK+ +R   +F+   + LY
Sbjct  156    TQFRGSEGKIKDQTPVETKPVFDQKPRISWRGNGEMFVVNYWKDQKRQFIVFETPCKALY  215

Query  65     QSNYYPNLLPPISFKGQGNFIACAAVHNDQNIVIFEKNCLLKSEFFIPEVRGD--IHKLL  122
              +S   P L P ++++  GN IA  +V N Q IVIFEKN   + +F   ++  D  I  L
Sbjct  216    RSEECPGLQPQVAWRPVGNMIAGLSVTNRQKIVIFEKNGQRRFDF---DLTFDVMIKNLK  272

Query  123    YHPFHQILVICSIEESGYSYIHIFLYLNAKWYLKQQLSYQAENQVKCLHWSDNLENLICK  182
              + P  QIL I ++  +G + IH+    N KWY KQ L + AEN +    W D       +
Sbjct  273    WSPCAQILAIHTVTPTGQT-IHLLTSSNYKWYEKQVLEFPAENALLDFDWLDT-----NQ  326

Query  183    LNILTAQNVEHIVFRMVINRCPTTGTVAVIDGSTVNFTFFSSEIVPPPMSNTYLKFDKPI  242
              L ++T  +V    FR V++   +     VIDG  +N T F++ ++PP         DK I
Sbjct  327    LQVVTQSDVIKYTFRNVVHH-NSAAICGVIDGKHLNLTDFNNAVIPPISYARRFTNDKQI  385

Query  243    NKIHFHPEIEQCILIDNAL-IINVIKFENNV----LKKM---------------------  276
              N + F  ++   I  +N L I NV + +  +    LKK+
Sbjct  386    NFVTFRHDLAMIIDSENDLKIFNVAEPDALLVTINLKKIVDLPQFALSCHHFLLSSESVY  445

Query  277    -----------PLCVGNTVDDLVTKL--NYLHFSDVTEAVEPI-NLNERGNSSL------  316
                          L   N   + +T + N+ H   ++   + I  L   GN+
Sbjct  446    FAVTSDESVFYSLDWKNPQANFLTAITDNFSHLLQISGDYDNISGLKSLGNNLFINNNDS  505

Query  317    VYCTGCRIDNRFYEFALNQCQDLLINKNVVCSKVFSIFIYKRYFLFTHASCKLYCVRLTS  376
              + C    + ++ Y   L       +N+  +     S  ++  Y L+T    +L+C+RL
Sbjct  506    ILCQIQTLGDQTYVCNLTSNNHFYLNEAKISDCANSFTLFDSYLLYTTKQSELFCLRLGQ  565

Query  377    ENVDKPNWDLNDYYSRDIEQGAKIVC-VNAKTEIIMELPRGNLETIKCRLICIDNIEKMI  435
              E  +         + R++EQGA IVC V    +I+++LPRGNLETI CRLI ID ++K++
Sbjct  566    EGQN---------FRRNVEQGATIVCAVPNSPQIVLQLPRGNLETISCRLISIDILDKLL  616

Query  436    SNNKWEDAISLIRHNKLNWNLLIDLNPQRFDNHILEFLRAAESVSLLNSIVSELGTTNCL  495
              +  KW +A+  IR  KLN NLL DLNP+RF   I  F++   +++ L +I  E    N L
Sbjct  617    NEQKWAEAVRFIRLEKLNANLLFDLNPERFLRQIAHFVQGVHTINELTAICLEFEEGNVL  676

Query  496    KTMYSQCVKDSTLPVHTVDNKHKILSEILNCIINLDLVTNLISIIILQIRHFSLKSALKS  555
               ++Y    K +  P       + I + +     ++D    + +I+ + +  F L+ AL
Sbjct  677    TSIYKNWGKTTDFPQKI----NTIFASLFKYFDSVDYSVYITTIVAVNLNFFKLRDALIY  732

Query  556    VHIVFKLGHLEICKKAIFQILLYHQCD--AIINTSFTLYDIEFMKFVHTCCSQDPKVFEP  613
              +  +++  +L+         L  + CD   +      LYD+E   F+ +CC  DP+V+EP
Sbjct  733    LQDLYRRTNLKEKLLNAVNTLKIYGCDHEKLYTECLLLYDLELAGFIASCCQLDPRVYEP  792

Query  614    QLKTLKHIEPVDLRFQMNVFAKNYKGAVKYLIRCPKYEEKFIKEFIAKHDVYEDAYKSVT  673
               LK L  +  V++R+++N+FAK  K A+ YL+RCPK +   +  FI  H+V   A+++
```

```
Sbjct  793   HLKQLSGLNEVEMRYEINLFAKKPKTAIIYLLRCPKAQTSDVLAFIKTHNVSRQAFENCP  852

Query  674   RKNSNFILCSQLYSTYLSTKLRYDEAGLILQRAELYNDAITEYKKSLNWYQVISLLVQLN  733
              KN  +   S  ++  LS K  + EAG++L+RA L  +A+ E++  LNW QV++LL +LN
Sbjct  853   PKNRFYQSVSHAFAGDLSAKGCHTEAGVVLKRAGLPEEALAEFQLGLNWRQVLNLLEELN  912

Query  734   VETSKSKKIISELAEGLVKQNRIDEAVVLYEMHCEDHLSAIYILLDKKLFQKALCMAKKY  793
             V+  +  KI+++LA  LV+ N + +A +L+E + +++  A+ +L++   F++A+ +A K+
Sbjct  913   VDKVEKIKIVNDLATRLVQSN-VRQAAILFEFYADNYEMAVKVLIEGFFFEEAIHIAMKH  971

Query  794   NCLDLVDKHIRPAIIKYRNELYSRISDLGKEFLICSERLQTVRHNKLLHLRNKYRSEVYD  853
                D++   + P ++K++  L  ++ +L + +    +RL  VR  K     +++ +++ D
Sbjct  972   KRGDIIVSDVIPMLMKHKIYLEEKLQNLNESYNKYKQRLAQVRQQKF----SRFNNDLDD  1027

Query  854   SFGECDLYSEYGSLISSQGSTLGSSRSKMSTTSAKNRRKLNKKKTDLREGSVFEDIALIR  913
                  DL+S+ GS IS    +   SRS+ ST S++NRRK  KKK DLREG ++EDIALIR
Sbjct  1028  CDERDDLFSDAGSTISKSSRS---SRSRSSTASSRNRRKEEKKKQDLREGGIYEDIALIR  1084

Query  914   HLYLLTASIFKCGQEVREICQTLTDDSSFAYSAM--LHKNLSEVQKKVISTIPEIWSNTF  971
              L+      +  G+EVR  C  L   +S +Y  +  +H    +   ++ + + EIW   F
Sbjct  1085  ALHSTIKEFYNKGEEVRNCCVLLLQESDVSYDEIKRIHDFYWKFDAEIQNGVAEIWPPHF  1144

Query  972   VNAAQSAEPEILAVVQNREQL  992
                 Q+ +   LA ++N E L
Sbjct  1145  YKNYQTLDVRELADLENFEHL  1165


Query  5171  IHHANPIGENIRDIRNLVYLSVHEVLFLTNDFDLIIYKTKSKEFVVVYSFAVKIESVAWN  4992
             I    PI  +   +++L+ +     + + ND  +I    K  +     +F  KI  ++WN
Sbjct  50    IQLGPPIFADASHLKSLI-IENKTCVVVNNDLFIIALPNKFDKI----TFDKKIIEISWN  104

Query  4991  PSETCLVVCDNEETIHGLIFECNIDLQEYKFMETWKTHLQDPVPNSVYVGWGSYNTQFKG  4812
             P+E  + V  ++    G I    ID +  +     K+     +P++VYVGWGS +TQF+G
Sbjct  105   PTEELVAVVFDD----GEISTFCIDYENAEAFPQGKSSTDAGIPDTVYVGWGSKDTQFRG  160

Query  4811  SQKK  4800
             S+ K
Sbjct  161   SEGK  164
```

Graphical representation

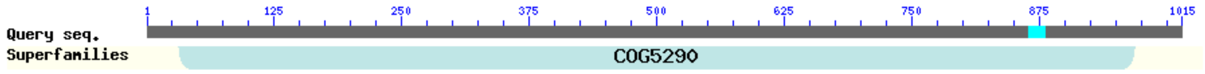

VIG (Vasa Intronic Gene)

>Cb.comp35716_c0_seq2 len=2030

cDNA

```
GTCATTCACAGACGCTGTCATTCAAATGCGCAAACCCATGCACGCTATCCTCGTGCGACCGGCCATATTGCATTGAATGAGTGCTTGAATAACA
GAGGGCTGAAGTAGCCAAAAATAAATCTTCCAACATCCCAAAGTTGCGAAGACTTTTTGCCATTTCTACCATCATGGAAAATTCGTACGGGATT
GGCGTAGCCAACAGGTACGCACTTTTTTTGGATGATGAATCCGATCCATTGGAAACGCTATCCATAAAAGAGCAAGAGAAAGAGCTTAAGAAAA
AAGCCAAAGTAGCGGAAAAGGAAAATAAAGGTAAAACCGAACAGCCCAAGCCGAAATCGTTGGCAAATGCCCAGAAAAAACCCATCAAGGAAAC
GACTAGTAATAAAGCTCAAGAAAATAAACGAGAAGACAATAAACCGAGTCAAAGGGCAACGGCAGATGGAAAACAGGACAGGACTTTTGCCAAG
TTCAATAATGAAAATCGGGAGGAAAGGAATAATAGGAGAAACCGCGAAGACCGACCTTACAATGGACCTGCAGAAAACCGTGACCGCGACCGCG
AAAATAGGCCGAGACGTGAAAACTCTGAGAATTTCGAAAACCGCAATCGTGATAGGGGCGAGAGAGGCGAACGACGAGAAGGAAGAGCAATCGG
TAATCGTCCTGGCGGACCCCGCGGACCTAAAAAGACCTTCGATGATAGAAGAGGTAAAAGGGAATTTGACCGACAAAGCGGATCTGACAAGACA
GGAGTTAAGCCTATTGAAAAGCGGGACGGTGCTGGGGCTCACAACTGGGGTTCTCATAAAGACATTATCGAAGCCGAAACCGAGAGGCCTAGCG
ATGCGGACCAGAGCTGGGGCGAAACCGAGAAGATCGAAACAAACGAAACGGAAAAAAAGGAAGAACAAGCCGAAGTGGAAGCTGCGCCCGTCGA
AGAAGAACCTAAAGAATTGACCCTGGACGAATGGAAGGCTCAGCGTGCCGGACGTGCCAAGCCGCAGTACAACATTCGGAAAGCGGGCGAGGGC
GAAGACCCGAGCCAATGGAAAAAGATGTTCGAGTTGAAGAAAAAAGAAAAGGAAGAAGAGTCCGAGGACGAAGAGTACGACGTATCGGAATATC
CCCAACGAGTTGGACGACAGAAGCATGTTTTGGATATCGACATTCAGTTTAACGACAACAGGCGTCCCGGCGGTGGTCGCGGAAGAGGACAAAG
ATCTGGCGTCAGAGGTGGACGGGGTGGTGGTTTGCCGCGCGGAGGCGGAGCACCACCGCGCGACGGCAGAGGTGACGGTTTGGAAAGACCGCGA
TTTAGGGACGAGCAGAGTGACGAGAAAGGTACTCCGAGGGCTCCCAAAGTCGATGACGAGCGCGACTTCCCATCACTTGGTTAAAAAGACGTTT
ACAATTGTACGCGGAATTGTGCCGCATTATGTTTTCGGTGGTAACCCATCACATCAAATTATAATGTATAGATAATACGATTGAATTAACTACT
```

TTGTTACATTGGGATGAATGGAAAAAAAATATGTTCGGTAATTGTCTACCGAAATATTTTTGGCGATTGTTTAACGACATTTTTCACCAACACC
CGAATTTTCCTCTAGAACTTATTGTGTGGTTTTAGTGTCTTGTAATTAAAATGCTATAATCGAAGTTAACTGTTATTATATTTTTAACGATATG
GGGAAAAAAAGCTCGGGCGTTGGCGAAATTTAATCCGAAGGTGAAAATTTATCCGCGAATCCAATTTCGAAAATTATCGTTTGCCATGATCAAA
AAAAAACTTTAATAAATCTTTAAGCTAGTTTTTCTCTACGATTATTGTGTATCAAGTTAGGACTGCGTGCTCCGCCCCCACCCCCAAGCTGAGG
ATGACGGTTACTAGTCAGTGTATGGCTGCATTGTTGACAGTTTAATACATGACTGCTCATAATAACGTCGTATTTTGTTTTTTTTTTGTTTTTT
TTTTTTTGTTTTTTTTTTTCAAGCAGAAGACGGGATACGAGATTACAAGGTGACTGG

Protein RF 3: 168->1400 (410AA)

MENSYGIGVANRYALFLDDESDPLETLSIKEQEKELKKKAKVAEKENKGKTEQPKPKSLANAQKKPIKETTSNKAQENKREDNKPSQRATADGK
QDRTFAKFNNENREERNNRRNREDRPYNGPAENRDRDRENRPRRENSENFENRNRDRGERGERREGRAIGNRPGGPRGPKKTFDDRRGKREFDR
QSGSDKTGVKPIEKRDGAGAHNWGSHKDIIEAETERPSDADQSWGETEKIETNETEKKEEQAEVEAAPVEEEPKELTLDEWKAQRAGRAKPQYN
IRKAGEGEDPSQWKKMFELKKKEKEEESEDEEYDVSEYPQRVGRQKHVLDIDIQFNDNRRPGGGRGRGQRSGVRGGRGGGLPRGGGAPPRDGRG
DGLERPRFRDEQSDEKGTPRAPKVDDERDFPSLG

Comparison with *Tribolium* hypothetical protein TcasGA2_TC001877 (346AA)

```
Query  82   DNKPS-QRATADGKQDRTFAKFNNENREERNNRRNREDRPYNGPAENRDRDRENRPRREN  140
            D KP+ QR+   DGK +R F KFNNENREERNNRRNRE+R +NGP ENR+R+    R R  N
Sbjct  23   DAKPNHQRSNVDGKPERNFNKFNNENREERNNRRNREERTFNGPTENREREERPR-RENN  81

Query  141  SENFENRNRDRGERGERREGRAIGNRPGGPRGPKKTFDDRRGKREFDRQSGSDKTGVKPI  200
             ENFENRNR+RGERG     GRA+GN+P GPRGP++ FDDRRGKREFDRQSGSDKTGVKPI
Sbjct  82   GENFENRNRERGERG----GRALGNKPAGPRGPRRNFDDRRGKREFDRQSGSDKTGVKPI  137

Query  201  EKRDGAGAHNWGSHKDIIEAETERPSDADQSWGETEKIETNETEKKEEQAEVEAAPVEEE  260
            +KRDGAGAHNWGSHKD+IE E ++P+DADQSW E ++ ETN    + +E+ E     PVEEE
Sbjct  138  DKRDGAGAHNWGSHKDVIE-EADKPNDADQSWSENDRPETNAAPETKEETE-NETPVEEE  195

Query  261  PKELTLDEWKAQRAGRAKPQYNIRKAGEGEDPSQWKKMFELKKKEKEEESEDEEYDVSEY  320
            PKELTLDEWKAQRAGRAKPQ+NIRKAGEG DPSQWKKM+EL+KKE E ESEDEEYD +EY
Sbjct  196  PKELTLDEWKAQRAGRAKPQFNIRKAGEGVDPSQWKKMYELRKKENESESEDEEYDAAEY  255

Query  321  PQRVGRQKHVLDIDIQFNDNRRPGGGRGR----------GQRSGVRGGRGGGLPRGGGAP  370
            PQRVGRQKHVLDIDIQFND RR  G                QR G   G G G    G  P
Sbjct  256  PQRVGRQKHVLDIDIQFNDTRRGAGRGRGQRSGPRGNRPTQRGGTGTGTGTGAAPAGERP  315

Query  371  PRDGRGDGLERPRFRDEQSDEKG-TPRAPKVDDERDFPSLG  410
             R           R+RDEQ+DE+     RAPKVDDERDFPSLG
Sbjct  316  ER----------RYRDEQADERTENRRAPKVDDERDFPSLG  346
```

Graphical representation

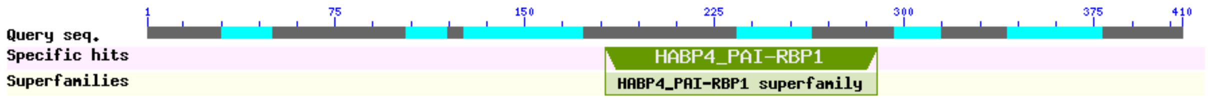

Homeless (spindle-E)

>Cb.comp40708_c0_seq1 len=4594

cDNA

GTAAAAGAAGAAGAAGAAGAAGATTTTGTATGGTGAACAATTTTTAACGGCGGTAAATTTTACTTTTTATCTCCGCGATCTTTTAAGACTTGAT
TTTTGATGTAATTCTATTAACACTGGTATAACTTGCCTCCATCTCCAGTCATGAACCAGTTACTGCAGAAGGTGGGTAAAAATATCACTTATCC
TATTGGCCGAGTAAACGGGACAATGTGTGTCCCCTACCGGGACGACGACTTCGATACTTCCGACAGCGAAGGGGACGAGTGTTCCGACAAACCC
TACGAGCAAGAGTTTGTCGAAAAAGAACTCGCGCTGTACGGGGAACCCAGCAACACCAACTTGTCCAAAGGGTTCGCTGACGATGTGAGCAGCC
ACACGGGGTTTTCCGAAATCGACTCCATCATAACTGACGGAAAAAGCTTGCCTGCCGAAATCTATCGCTCTTATAATTTTAAACTGAACACCGA
CATAAAGAAAGATTTGCCCATTGACTCTTACAAACAACAGATCCTATCAAGGGTCGACCTTAATCAAGTGATAGTTATCAAGGGCCCGACTGGC
TGCGGCAAGTCCACCCAAGTTCCGCAAATGATTATGGACAATTTTCGAGAAAAAAACATGTACTGTAATGTCGTGGTCACCCAGCCTCGAAAAA
TTGCAACTATAAATGTGGCCAAAAGGGTTTGCCAAGAGAGAGGGTGGACTTTGGGGACCGTCTGCGGGTATCAGGTGGGTTTGGAGAAGAAGTT

GTCCCCGGACGTCATCATAACCTATATGACTATGGGGGTGTTGTTGCAAAAGCTGATACGGGCCAAGTCCTTACGGGAATACACGCATATAGTA
ATCGACGAGGTCCATGAACGAAATCAAGAACTTGACTTCCTCCTTCTGATCATCCGCAAGTTTTTATTCACCAATTCTCCACAGACGAAGATTG
TGCTAATGTCTGCCACAATAAAAGCAGACGAGTTTGCTTACTATTTCCGGAGGCGGACATTCGGGCAGACCATTCCCGCCCCCATTATTTCCAT
CAGCAAGGAGAGTCTGTACACCAAAACAATTTTTTATTTGGACAAGATTGACAAGATTAGCCAAAAGTTGCCAAAGTTCGACTTGATGAGGCCT
GAAATATCAAACGAGGTGTGGAACGTGTTCCTGTTCCTCGTAGCTATCTTCGACCGGCTCGAGCAGGGCCATTCGGTCATGCACCGGAACGGCA
CCGTGCTCGTCTTTTTGCCGGGCATTTACGAAATAGAAGAAGCGCACGCTAGGCTCGCGAAAGAGGACCCCGATGGCAAGAAATGGGACATCAT
ACCGCTTCACTCGTCGCTTCCCAACGACGAGCAGGCCCGGGCCTTTGCTCCATCCAAATCAGGAACCCGGAAGATCATCCTATCGACCAACATC
GCCGAGAGTTCGGTCACGGTGCCCGACTCGTCGTTCGTGATCGACTTTTGTCTGACGAAAGTTATGACGGTCAATCCGGAGACGAAATACTCGA
GCCTGAAGCTTGAGTGGGCGTCGCACGTAAACTGCGACCAAAGGGCCGGCAGGGTGGGGAGAACGTGCGACGGTCGCGTGTACAGGCTGGTCAC
CCGCGAATTTTACATGGAGTTGAACCGACAAGGCACGCCCGAGATGTTAAACGCGCCTCTCAACCGAGTCGTCTTGCAGGCGAAGATGCTCGAA
CTGGACGAGACGCCTGCGCAGATCTTGGCGCTCGCGATGACCCCGCCCCCTCTCAGGAACATCGAGGCGACCATTTGGCAGCTGAAGGAGATTG
GCGGCCTCCTAAAAACTTGCCGCGGCATTCCCGCGAACGCGGACGGCGACATAACCTTCCTGGGTCGGGTGATGTCCGGCTTGCCCATAGATGT
GCACCTGTCCAAACTTATTGTTTTGGGTCAGTTGTTCAGCTGCCTCGAGGAGACGATAGTCATAGCCGCCGGATGCTCCATCCAGAACATATTC
GCCGTGCCTTTTCAAAGACGATTGGAGGCTTATCGCAAGCAGCTACTGTGGTCGGACGGTTCTTGCAGCGACCTGATTGCCTTGCTTAACTTGT
ATACCGTTTGGTTGAGTTTGAAGCGAGAAAACGCGTTCGCGTCTCATAGCCAGGAGTTGATCTGGTGCCGCACCAACATGGTCAGCTTGAAGGG
TCTGAAGGAGTGGAACCTGCTGATCGCGGAGATCAAACAGCGATTGGAGAAAATGCAGATTAAAGAGACAGACGGTCCAGGCAAAGTGATTTTG
AGTGACACAGAGAAACCGACCGTCTTGAAGGTCATAATGGCGGGCGCGTTCTATCCGAATTTCTTCGTCAAAACGCCGGATTCGAGTCAGATGA
TGGAACGTGAGGCGGTAAAGGCGGTAGGCGGTCGCGACCCCTTCTCCACGGTATACTTTACGGGGATGGACCCGAAGCAGCCGGGTCAGGTGTA
CGTCCGGCCGATCAAGCGACTGATCAGAGAGGACGGCGAGCGGGAAACTGACGTGCAGATCGGTTTCGACGGTAGCTCGAAAATCTACGTCGAG
TTCAAGGGGGCGGTGCCGCGCGAACCGATCACCGTCAACGTGGACGGCCACCAGAGGCTGACGAACGTGCCGGGACGCGTGCCGTTGCCCGTGT
ACGAGATACTGCGCAAGCGGCAACTGCAGTACCCATTTCAGCTGAAGGTGTTGCCGCACAACGAGGCGTGGGAGTTTGCCGAGAAGCACGGACT
GCGTCGGAACGTACCCTCGGGCCAGCTGTCGCATCGCGAGCATCAGAACTGCTACTCGTCGGTGAAGTACTCGCCGTTACCGCCGATGGACATC
GCGTTTATCTCGCTACATATATCGAACTTTATAGACGCGGGGCATTTCTGGGCTCAGAACGCCACCGAGGAGACTAACGTCTACCTGCAGCAGA
TCGACGAGGCGCTGAACAAGCAAGTACTCGTGCAGGTGACGGAGGGTGTCAAACTGGATAAGGTGTATGCAGCGCGATTCAGGGAGGACGGCCA
GTTCTATAGGTGCAGGGTGGCGGCGACGGGAGGGCGGATCAACCAGATCATATTCATCGACTACGGCAACCTGCAAGAAGTAGAAACGAACGAG
CTGTACTACGTGCCTCAGAGGCCGCAGTGCGTAATGGCGCCGCTGGCTTTCGAGTGCGTGCTGCACGGCGTCAAACCGACGTTTCGTCTCAATC
CTTCCGGCGTGTGGGACGAAACAGTTAACACCGACTTCAAACGGCTGACTGAAGGGATTCTGTTGTACGGAGAGGTGTATTCGGTGGTCGGAGA
CGTGGTGGAGCTTACGCTGTACCGTACCGAGAACAAGGAGATTTCTCTGAACCAGTACTTGCTGAACAAGCACTTCGCGGAGGCGTGCGCGCCT
AGTTTTCTATCGAAGCAAAACCACGAGCAGAGGCTGGCGGTCCAGGCAGCCGAAAACCCCGACCAAGAGGCGAACCGACTCTCGTACGACAAAA
TAGTCAGCTACAGCGACTTTTTGGCTCCCGAAGCGACCGTCGGGCATCACCACGCCGTCATCCATCTGCGCGGTCCGTTTAGCCCGCTCGAGGT
TAAGCTGTACTGCTGCACGATGGCGGGCAGCGCGAAAAGCGTCGACGTCGAGGGCACGTCCGTTAACGCGGTACTGCTCGACAACGAGCCGGAG
AGTCCGCACGCCCGTCTTCTGGTCGCCGCCCACGTAGGTCAGTCCGGAAACGGGGACCGGCTCAAGCTCAGACTGACGACGCTGATGCCGAACC
TGCCCGGGCTGCCGATGCTCCTCGCCGCCATCTTTTGCCCGACTATGGAACCCAAACTGACCGACGACAATACTCTAGTGGCGGCGGTATTGTG
CGGTCTGGGGTGCAACGAGGCTACCGGCAGGGCCCTTTACCCCGCCCACGACATATGCCTGACGCTCGACACGGAGCTGACGATGGGGGAACTC
GAAATGATCAACCGTCTCAGGTACCTGATGAACTCGGGCGTTAAGTTGCTGCACAACATCGAACGAAACATGTCGACCCAGAACGAGCTCGTCG
ACACGCAGATCAAAATCAAGGAGCATCTGTTTAGATTGATTATAATGGAACGCGTCCCAATCGACCGGCGCAACATTGCGCCGCGCAGCACCGC
GTGGGGCAAACACGGCGGCCTGGGCTTGCTCAAACCAAGTATGGACAACGAAGAGCAGGATGTTTGGAGTCTGCTATGGTTCGTCAAGTTGGCG
GGGCAGACCAGGCGGCTGAAACTTGCCGCCACTAATCTGGAAACCATGCAGGATATGAGTCTCAACATGTTGCCGTTCAAGGACATCGGGTGCG
TCCTCTGCGGGAAGGAGCTGTTCTCCATTTATGAGCTTAGGCTGCATCTGGTGTCGCGTGGTCACAAGGAGGAAGTGGAACGGTACCAGGAGCG
GCTCGACGAGTCAGACGACGGCGACGACGACGACGAATGAGCGTTTTTAGGGGGAGGGGGTGGCGTTTTGTTTTTGTTTTTTATTTTTGAGCGC
TTTTTGTTTCATTTTATAGAGTTTCGATTTATGTCCGGACGTGCCTGAAGGCTCAAATAAAAGATTTGATTAAAAAAAAAAA

Protein RF 1: 145->4458 (1437AA)

MNQLLQKVGKNITYPIGRVNGTMCVPYRDDDFDTSDSEGDECSDKPYEQEFVEKELALYGEPSNTNLSKGFADDVSSHTGFSEIDSIITDGKSL
PAEIYRSYNFKLNTDIKKDLPIDSYKQQILSRVDLNQVIVIKGPTGCGKSTQVPQMIMDNFREKNMYCNVVVTQPRKIATINVAKRVCQERGWT
LGTVCGYQVGLEKKLSPDVIITYMTMGVLLQKLIRAKSLREYTHIVIDEVHERNQELDFLLLIIRKFLFTNSPQTKIVLMSATIKADEFAYYFR
RRTFGQTIPAPIISISKESLYTKTIFYLDKIDKISQKLPKFDLMRPEISNEVWNVFLFLVAIFDRLEQGHSVMHRNGTVLVFLPGIYEIEEAHA
RLAKEDPDGKKWDIIPLHSSLPNDEQARAFAPSKSGTRKIILSTNIAESSVTVPDSSFVIDFCLTKVMTVNPETKYSSLKLEWASHVNCDQRAG
RVGRTCDGRVYRLVTREFYMELNRQGTPEMLNAPLNRVVLQAKMLELDETPAQILALAMTPPPLRNIEATIWQLKEIGGLLKTCRGIPANADGD
ITFLGRVMSGLPIDVHLSKLIVLGQLFSCLEETIVIAAGCSIQNIFAVPFQRRLEAYRKQLLWSDGSCSDLIALLNLYTVWLSLKRENAFASHS
QELIWCRTNMVSLKGLKEWNLLIAEIKQRLEKMQIKETDGPGKVILSDTEKPTVLKVIMAGAFYPNFFVKTPDSSQMMEREAVKAVGGRDPFST
VYFTGMDPKQPGQVYVRPIKRLIREDGERETDVQIGFDGSSKIYVEFKGAVPREPITVNVDGHQRLTNVPGRVPLPVYEILRKRQLQYPFQLKV
LPHNEAWEFAEKHGLRRNVPSGQLSHREHQNCYSSVKYSPLPPMDIAFISLHISNFIDAGHFWAQNATEETNVYLQQIDEALNKQVLVQVTEGV
KLDKVYAARFREDGQFYRCRVAATGGRINQIIFIDYGNLQEVETNELYYVPQRPQCVMAPLAFECVLHGVKPTFRLNPSGVWDETVNTDFKRLT
EGILLYGEVYSVVGDVVELTLYRTENKEISLNQYLLNKHFAEACAPSFLSKQNHEQRLAVQAAENPDQEANRLSYDKIVSYSDFLAPEATVGHH
HAVIHLRGPFSPLEVKLYCCTMAGSAKSVDVEGTSVNAVLLDNEPESPHARLLVAAHVGQSGNGDRLKLRLTTLMPNLPGLPMLLAAIFCPTME
PKLTDDNTLVAAVLCGLGCNEATGRALYPAHDICLTLDTELTMGELEMINRLRYLMNSGVKLLHNIERNMSTQNELVDTQIKIKEHLFRLIIME
RVPIDRRNIAPRSTAWGKHGGLGLLKPSMDNEEQDVWSLLWFVKLAGQTRRLKLAATNLETMQDMSLNMLPFKDIGCVLCGKELFSIYELRLHL
VSRGHKEEVERYQERLDESDDGDDDDE

Comparison with *Tribolium* spindle E (1431AA)

```
Query  17    GRVNGTMCVPYRDDDFDTSDSEGDECSDKPYEQEFVEKELALYGEPSNTNLSKGFADDVS  76
             G VNG M   Y   + +T +S   E      +QE+++KEL+ Y   +    S G  D
Sbjct  20    GVVNGIM--DYEKPEENTYNSSDSESESDTEQQEYIKKELSTYFPETEAPCSGGLTDIED  77

Query  77    SHTGFSEIDSIITDGKSLPAEIYRSYNFKLNTDIKKDLPIDSYKQQILSRVDLNQVIVIK  136
              H   S + + I +   +P +++ +Y F  +T  KK+LPIDS + +IL  ++ N V++I
```

```
Sbjct  78    GHEE-SVLGTDIMELLHVP-KVFETYKF--DTYYKKELPIDSSRDKILDMINTNSVVIIH  133

Query  137   GPTGCGKSTQVPQMIMDNFREKNMYCNVVVTQPRKIATINVAKRVCQERGWTLGTVCGYQ  196
             GPTGCGK+TQVPQ I+D+ R      CN+VVTQPR+IA IN+A+RVC+ERGW +GTVCGYQ
Sbjct  134   GPTGCGKTTQVPQYILDHCRATKSPCNIVVTQPRRIAAINIAQRVCEERGWAIGTVCGYQ  193

Query  197   VGLEKKLSPDVIITYMTMGVLLQKLIRAKSLREYTHIVIDEVHERNQELDFLLLIIRKFL  256
             VGL+K +  DVI+TYMT  VLLQKLI  K+L  +TH++IDEVHER++ LDFLLLI+RK+L
Sbjct  194   VGLDKNVGDDVILTYMTTEVLLQKLISQKNLNRFTHVIIDEVHERSKSLDFLLLIVRKYL  253

Query  257   FTNSPQTKIVLMSATIKADEFAYYFR---RRTFGQTIPAPIISISKESLYTKTIFYLDKI  313
             FTNS   KI+LMSAT++A +FAYYFR   R    Q + AP++ ++K+S Y  +I+Y
Sbjct  254   FTNSSSVKIILMSATMEAQDFAYYFRSISRNNPQQYLLAPVLPVTKKSEYKVSIYY---N  310

Query  314   DKISQKLPKFDLMRPEISNEVWNVFLFLVAIFDRLEQGHSVM----HRNGTVLVFLPGIY  369
             +  +  +P ++   P +  E ++V   L+++FD+LE+  S +      NG+VLVFLPG +
Sbjct  311   EHFASAMPPYNFEEPCMHKEQYDVCAKLISLFDKLEENESKLTLAERINGSVLVFLPGFH  370

Query  370   EIEEAHARLAKE-DPDGKKWDIIPLHSSLPNDEQARAFAPSKSGTRKIILSTNIAESSVT  428
             EIEE H  L +E +    +W+IIPLHSSL  +   +AF   +   RKIILSTNIAESSVT
Sbjct  371   EIEEMHKVLVRERNTSVLEWEIIPLHSSLAQEHMVKAFQKPRQRCRKIILSTNIAESSVT  430

Query  429   VPDSSFVIDFCLTKVMTVNPETKYSSLKLEWASHVNCDQRAGRVGRTCDGRVYRLVTREF  488
             VPD +FVIDFCLTK MTVN  TK+SSL L+WAS+ NC QRAGRVGR  +GRVYR+V   F
Sbjct  431   VPDVNFVIDFCLTKNMTVNEVTKFSSLSLQWASYTNCIQRAGRVGRVANGRVYRVVPTSF  490

Query  489   YM-ELNRQGTPEMLNAPLNRVVLQAKMLELDETPAQILALAMTPPPLRNIEATIWQLKEI  547
             Y+ E+ +   PE+  APL  V+L  K+L L++TP  +L+LA++PP L+++E  +W LKE+
Sbjct  491   YLHEMKQTTVPELQRAPLENVILYMKLLGLNDTPKNVLSLALSPPNLKDVEQCVWHLKEV  550

Query  548   GGLLKTCRGIPANADGDITFLGRVMSGLPIDVHLSKLIVLGQLFSCLEETIVIAAGCSIQ  607
             G LL+TCRG    ADGDITF+GRVM  LPID+HLSKLI+LG +FSCL+E +++AAGC  +
Sbjct  551   GALLQTCRGHRTPADGDITFMGRVMGSLPIDIHLSKLILLGHMFSCLDEAVIMAAGCMTK  610

Query  608   NIFAVPFQRRLEAYRKQLLWSDGSCSDLIALLNLYTVWLSLKRENAFASHSQELIWCRTN  667
             NIF   F  R   YR++L+W+DGS SD + LLNLY VWLS+KR+ AF+S  QE+ WC+T+
Sbjct  611   NIFVQNFYDRFRTYRQKLVWADGSHSDFMILLNLYNVWLSMKRDRAFSSSHQEIGWCKTH  670

Query  668   MVSLKGLKEWNLLIAEIKQRLEKMQIKETDGPGKVILSDTEKPTVLKVIMAGAFYPNFFV  727
              V+LKGL+EW++LI EI  RL+++ I++  GP  + LS  EKP VLKVI+ GAFYP +F+
Sbjct  671   FVNLKGLREWDILIQEIHSRLKRLNIQKLPGPSSIPLSIVEKPMVLKVIICGAFYPYYFI  730

Query  728   KTPDSSQMMEREAVKAVGGRDPFSTVYFTGMDPKQPGQVYVRPIKRLIREDGERETDVQI  787
             K+ D   +  +EAVK + GRDP +TVYFT M   QPGQ+YVR IK+L+  + E + +VQI
Sbjct  731   KSSDFGNVDAKEAVKILNGRDPCNTVYFTNMKMNQPGQIYVRQIKKLM--NCEDKPNVQI  788

Query  788   GFD-GSSKIYVEFKGAVPREPITVNVDGHQRLTNVPGRVPLPVYEILRKRQLQYPFQLKV  846
             GFD  S+K++VEFK    R+P  V +DG Q +  V   + + VYE +RKRQ++ PF L++
Sbjct  789   GFDPQSTKVFVEFKAT--RQPEQVTIDGRQYIATVASNIAVDVYEAVRKRQMRVPFVLRI  846

Query  847   LPHNEAWEFAEKHGLRRNVPSGQLSHREHQNCYSSVKYSPLPPMDIAFISLHISNFIDAG  906
             LP ++AWEFA     +R     Q++  E  NC++++ YSPLP +DI +I++ +++ IDAG
Sbjct  847   LPDSKAWEFANMTQAKR-----QIAESEDVNCFTTLDYSPLPTLDIEYITVTVTHIIDAG  901

Query  907   HFWAQNATEETNVYLQQIDEALNKQ--VLVQVTEGVKLDK-VYAARFREDGQFYRCRVA-  962
             HF+ QN  EET + L QI  ALN     L    E +K++  +YAA F EDG+FYRC+V
Sbjct  902   HFYCQNWNEETRMLLDQIFAALNGPGVFLEPAGEKIKVNSDIYAALFNEDGKFYRCKVID  961

Query  963   ATGGRIN--QIIFIDYGNLQEVETNELYYVPQRPQ-CVMAPLAFECVLHGVKPTFRLNPS  1019
              T G+ N  Q+ FIDYGN+Q V  N LY +P+  + C + P+A  CVL GV+P   LNP
Sbjct  962   LTPGQPNVAQVCFIDYGNVQRVPKNRLYKLPENSEPCRVQPIAMCCVLSGVQPDLVLNPK  1021

Query  1020  GVWDETVNTDFKRLTEGILLYGEVYSVVGDVVELTLYRTE--NKEISLNQYLLNKHFAEA  1077
              +W E+VN  ++ T G+LL  +V+SVV +VV L L+       +S NQ+L+N+   +
Sbjct  1022  ALWSESVNNILRKKTTGVLLNAKVFSVVDEVVHLELFLQNPGRNSVSFNQWLINEGLGQK  1081

Query  1078  CAPSFLSKQNHEQRLAVQAAENPDQEANRLSYDKIV-SYSDFLAPEATVGHHHAVIHLRG  1136
             C  S  SK +HE RL VQ++E+P   +   + ++IV SY+DF APE++      +I L+G
Sbjct  1082  CEESQRSKMDHEMRLKVQSSEDPSNMSALFNKNQIVTSYADFEAPESSEATE--IIELKG  1139

Query  1137  PFSPLEVKLYCCTMAGSAKSVDVEGTSVNAVLLDNEPESPHARLLVAAHVGQSGNGDRLK  1196
             PFSPLE+K+      A     V V+G SVNAV+LD+ PE  HA LLVA  V QS     +K
Sbjct  1140  PFSPLEMKVCGLVQASQGAPVHVDGDSVNAVMLDDNPEDYHATLLVAGQVSQSSTTSAVK  1199

Query  1197  LRLTTLMPNLPGLPMLLAAIFCPTMEPKLTDDNTLVAAVLCGLGCNEATGRALYPAHDIC  1256
             +  TT+MPN+PG PML+  +FCP MEPKLT D + VA++LCGLG  E T RA +P HDIC
Sbjct  1200  ISQTTIMPNIPGFPMLMCLLFCPQMEPKLTPDGSRVASILCGLGYKEVTQRANFPMHDIC  1259
```

```
Query  1257  LTLDTELTMGELEMINRLRYLMNSGVKLLHNIERNMSTQNELVDTQIKIKEHLFRLIIME  1316
             L LDT+L     +   IN LRY MN   +K++  I+   ++     E+   TQ   +K+ LF L+ M
Sbjct  1260  LVLDTDLRSEIITKINALRYYMNEAIKIMSQIQEELARPEEMYTTQRFLKDELFHLLHMR  1319

Query  1317  RVPIDRRNIAPRSTAWGKH-GGLGLLKPSMDNEEQDVWSLLWFVKLAGQTRRLKLAAT-N  1374
             +   +DR  N+        W  K      + +L+   MD++E+ +WS  LWFVK   G+  R   K++      N
Sbjct  1320  QQTVDRVNVR-YPDVWNKGLDNMEILRIDMDDDEEAIWSYLWFVKF-GEDRLSKMSINKN  1377

Query  1375  LETMQDMSLNMLPFKDIGCVLCGKE-LFSIYELRLHLVSRGHKEEVERY  1422
             L+ +   ++     P ++I  C LC     L +I ++R+HL     HK   + +Y
Sbjct  1378  LDELTQIARRTAPEREIKCELCNSATLRNISDVRIHLFREEHKSNLAKY  1426
```

Graphical representation

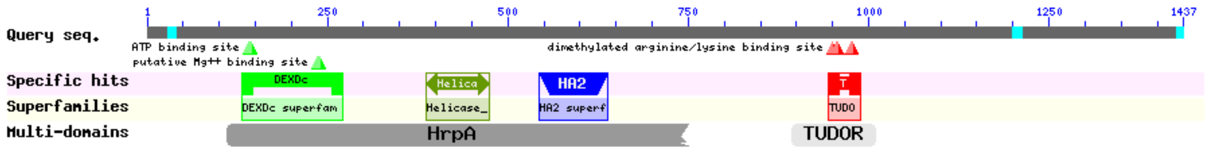

Staufen

>Cb.comp23511_c0_seq2 len=3907

cDNA

TTTTTTTTTTTTGGTGATACTGTTTATTGATTTCAAATTCAAATGAGGCTACATGCTTACTTTATATCCGTAATACAGAAGAATGATCTAACTC
TTAAACTACAATACACAACAGTTCAATCCAAAGGCCCCGGAATAATGAAATCAATCTACAAACAATAAGTGAGAAATAAATACATTTCAGCAGA
TCAGAATTGTCGTGTAGAAGAATTCAAGCGCCTAAAGCTATACGCTAAATTTGACAACAAAAATTTTTAATTTGCATTCTTTAAACAAAAAAGA
AAAATGTATCGCTATCGTGAACTCGATAATTCTTCCAGTGTGTTCAAAGCGCTGAGCAAGTAAAAAATTTATGCACTTACACGGGTCGCACTTA
ATTTTCGAGTGACAATTTAACAAATCAATTCTGAACTACTGGACCAGATTTCGTTTTCGGTCGCCCTGTAACTAAAACGAAATACGGTAAACAC
GGTCTTTTTTTTTCTTTTTCCTTTTTTTTTGAAATATACAGTGTAAGCAATAACCAGAATTATACGTCATACATGAAATCATTTCATATATTTC
ACATATGTTAATGACATCACATAAATATGAGATTAATAATAATTCTAAGTTATCCAAGTATATGAATGATGCAACATTTTTATTTTTTTCGTTTT
TTTTTATTTTTTCTTTGACATGGCCACATGGAACTCCCAGTAGTTGAAGTCTTCTGTACCAATGAAAAAGGATACTGTAAATACTAGATAGTGC
TTTCAAAGTGTTGGAAGAACCGGTAAAGGAATTTATCGACATCATAAACAGCCACATTCATTGGTATAGTCTACAAAGAGTTCAAAGTTTATCA
AACATATATTATTTTTCTTCGAAAAGACTTGTTTCATTCCGTTCAAAAATTAAAATAAAAAAAATTAAAGATCAATTAAGACGCAACCTCGATA
GTCCAAAGCACATGGTTAGCCACTACTAACACTATAACCCAGGTCAAATGGTAATAAATCAAACCGGTTTAGTAGTCGAATAAAATTAATTAAC
ACATATACTTTCTTTTGTACGAAAAAAAACAAGCGCACTAATGAACGTTTCTACTTCTCTATTTGGGCATTGCCATCCTTGGGGGCAATGTCGA
GGCCCAACTCTGAAAGAACCTTCAAAGCTTCCAAAGCAGCTTTCTCGTGCGATGCTTCCGTGGTTGGGCCTTCGCCATGACAAACTTGAGTCGG
ATTTGTTCCAAGACTTACCAGAGTCAAATACATTTCATGGTTTGCCTTTGGGAAATCTGAAAACTGTACCTGAATGTTCATCAACTGGGCCAAA
TACAAGAGCTGGTCTTTGGATCTAACCCCTGGCGGCGGATTTTTCATCGTCGCCGGTTGCATTTGTACCGGTTTCGGTTCAATATTGTCACTTT
TCGGCTTACTGAATCCGGCATTTTGCTCGGTAACCAACAGTACACCCGGTACAAGTTGTCGTCCGCCGCTGCCTCCGATTGATTGAACCGGAGC
TTGCTCCACTTTTTCCTCCACAAAAGTGACCTTACGATTTTTTTCGTTACTACCAATCAACGAATCAACTTCCTTGTCACTTTTATTTTGGGGC
TGGGTTTTGGTCCCGGTGGTACTATAACCCAGTTCAGTCAACAGGGCGTCTGCCGCGTTACGTTTCGCTACCTTTTTATTAGGTCCGACTCCTG
TGCATGAGTGACCGTTAACGGAGGCTTCGATAACAAACTCTCGTCTGCGCGGGGCCCCGCGCTCTTCTAAAACTGTATAAACAGGTTCCCTTTC
TTTGTTAGCCTGTTGAATTTGAATCAAACGAGAGATGGGATTTATTTCTTCCGGAGTTTCCGAATTTTTATCCACGTTAACTTTAATAAGATTC
CGTGTTTTCTTCTTGTTCGTTACCCTCTTGCGTTTCATTTGCGATATATTCGCCACGTTCGGGAGTGGTGGCAGTTTGGATAACTCCTCCAGCA
TCTTTTCCGCTGCCTTTTTTTTTCGAAATCTTTTTCCCGTTACCCTCGCCTTCCGCTAGAAAATCACCGACCCGACACTGTGTTACGAAAACTTT
CATATGGGGTGGACCCTTCTCACTCAAAACTTCAAAAGTAACATTCAGATTTCTCTTTAGAGATATCTCGTAAACCAACGAAATGGGCGATTTT
AGTTCGGAATTCACATCATTGGTTAACGAATTCGACAGTGTTTGTTGGTCATTTATTTGCCCGTTTTCAGTATTTGTCGCATGATCAGTATCAT
CCACTACACCGAGTTGTTTTATATGCTCTATGGCTTTGGCGGCAGCATCATGCCGTGCGGCTTGCACGGTGTATCCATGACCTGGATACTCCCG
TTCCCCCACTCTTAACCTTACAGTATATGGGTCGCCCGGATTATGATGAGGAGCGCCAGGTCTGAATTGGCCATAATACCGATTGTCATATGTA
TAATGACCTCTGATATTACGCCGGGCGTCATAACCATATCTGGGTTGAGCCTGGGGATAGTTGAGATTGTGCCTTGGATAATAACTCGGCTGGT
TCATATAGCCCTGATGAGGTGGTGCCCCTCCACCTTCAACAACGTACACTGTACGTTCTCCCCTTTTCATTGCCAAAGCATTGAGTTCGACTGT
TGGAGTGACAACACCTGGATTCGAGGGTCTATTGCCAGGCCTATTAATAATTGTTTTCTGTGGTGGATGCTTAAATTCGGTCTTGGTTAATGCT
TTAGCGGCGGCAGAATGTTGGGCCTTCTTAATACTTGGACCCTCGGCTTCATATTCCTCTTTTCCTAGTTTCAAGGTAACTGTGAATATTTTTT
TGTGTGCGGGACCTTGTTCTCCCGTTAGCCGATATTGATGTTGGATTTTATTGTACCTTGCTAATTCATTTACCAAGCACATGGGTGTTTTTTC
TTTTATGTTTGCCAAAGTCATGTTGGGCGCTGCAGAAGTACTAGTGTTTGGACAATTTTGATTCAGTTGTTGTTGATCTTGGTTATTCATGAGC
TGTTGTGACTGTGGTTGTTGTGAATGTTGATCTGGCTCTGGCTGTTGATTATGTGGGACAGGTTGTGTAGGTGGTGCTTCATAATGATAAACTT

```
GAGGCTGCTGAAGATTTGGTTTGGCTTGTGGTTGCGGGATTGAAGTTGTGGTTATAAGAGCAGGCCCGGGCCCAGGTGACATTGAGACAAGTAC
CCCAGTTGCAGGCATGCTCATAATCATAGGAGTTCGTTGATTTGGAGGCGGCCCCATTTGCTGCATTCCCATTCTTGGATTTCTCTGCATGCCG
TTTTGATTAATGTGATGCTCTTGAAGCATGATAATAGAAACGAAATAATTAAAAATGCTAACCGAAATCTCTACAAGTTTGGGAGTGCACCTAT
TTAACCGGCGTCAGCGTATTATCGAATTACAGTAGAATTCCAGTACGTAGTAATAATCGTTGACAACAAATCTTGATTTCTTAACCAAACACCA
CTACTAAATGTTGTACGTTCGTTGGCAAACAATAAAGAAAAAAACAGCAACTAACATGCGGCCATGTTAGAAATATCACGACGATGGATTTTAT
AAATGAAATCAACGCATAGCGACCGCTAGTGTCGCTATTTTCTTGTGGGTCGTTCAGCAGAGGGGAGCGCTTTCCAATAGCAGCAGCACTTGCA
TTAGGCCTGCGCAAAAATTCGTATTATTTCTCGTCCGTTTCGTTTAGAGAATATCATTGTCTGATCAAGGTTTCTAGAAGGGAGAACATGCCAA
GTTTAGGAACGCGATACATTTACTATGGGAACGCGATCGCACGGCAGCAATCACAACCAAACGGCTTATTTGATATTCAAAAATTTAGTACGAT
TACGTTCCTTTCCAATTAATTTATGAAATTATTTACACCTAACAATATAATCA
```

Protein RF -1: -3319->-1085 (744AA)

```
MLQEHHINQNGMQRNPRMGMQQMGPPPNQRTPMIMSMPATGVLVSMSPGPGPALITTTSIPQPQAKPNLQQPQVYHYEAPPTQPVPHNQQPEPD
QHSQQPQSQQLMNNQDQQQLNQNCPNTSTSAAPNMTLANIKEKTPMCLVNELARYNKIQHQYRLTGEQGPAHKKIFTVTLKLGKEEYEAEGPSI
KKAQHSAAAKALTKTEFKHPPQKTIINRPGNRPSNPGVVTPTVELNALAMKRGERTVYVVEGGGAPPHQGYMNQPSYYPRHNLNYPQAQPRYGY
DARRNIRGHYTYDNRYYGQFRPGAPHHNPGDPYTVRLRVGEREYPGHGYTVQAARHDAAAKAIEHIKQLGVVDDTDHATNTENGQINDQQTLSN
SLTNDVNSELKSPISLVYEISLKRNLNVTFEVLSEKGPPHMKVFVTQCRVGDFLAEGEGNGKKISKKKAAEKMLEELSKLPPLPNVANISQMKR
KRVTNKKKTRNLIKVNVDKNSETPEEINPISRLIQIQQANKEREPVYTVLEERGAPRRREFVIEASVNGHSCTGVGPNKKVAKRNAADALLTEL
GYSTTGTKTQPQNKSDKEVDSLIGSNEKNRKVTFVEEKVEQAPVQSIGGSGGRQLVPGVLLVTEQNAGFSKPKSDNIEPKPVQMQPATMKNPPP
GVRSKDQLLYLAQLMNIQVQFSDFPKANHEMYLTLVSLGTNPTQVCHGEGPTTEASHEKAALEALKVLSELGLDIAPKDGNAQIEK
```

Comparison with *Tribolium* staufen(724AA)

```
Query  18    MGMQQMGPPPNQRT-PMIMSM--PAT----GVLVSMSPGPGPALITTTSIPQ-PQAKPNL  69
             MGMQQMGPPPN R+ PM+MS+  P T    GVLVSM PGP   LI+T SIPQ PQ K N+
Sbjct  1     MGMQQMGPPPNHRSQPMLMSIQPPVTAAHAGVLVSMPPGP--PLISTPSIPQQPQPKNNV  58

Query  70    QQPQVYHYEAPPTQPVPHNQQPEPDQHSQQPQSQQLMNNQDQQQLNQNCPNTSTSAAP-N  128
             QQ  VYHYE                 ++   +++Q  +N  QQ  +Q  PNTSTS+AP +
Sbjct  59    QQ--VYHYE-----------------NTASSETEQAQHNDTQQ--HQTGPNTSTSSAPPS  97

Query  129   MTLANIKEKTPMCLVNELARYNKIQHQYRLTGEQGPAHKKIFTVTLKLGKEEYEAEGPSI  188
              TLANIKEKTPMCLVNELARYNKIQHQY+LT E GPAHKK+FTVTLKLG EEY++EGPSI
Sbjct  98    TTLANIKEKTPMCLVNELARYNKIQHQYQLTEESGPAHKKVFTVTLKLGNEEYKSEGPSI  157

Query  189   KKAQHSAAAKALTKTEFKHPPQKTIINRPGNRPSNPGVVTPTVELNALAMKRGERTVYVV  248
             KKAQHSAAA++L KTEFKHPP KT  NRPG R +NPGV+TPTVELNALAMKRGER VY+V
Sbjct  158   KKAQHSAAAQSLAKTEFKHPPSKTARNRPGTRATNPGVMTPTVELNALAMKRGERPVYIV  217

Query  249   EGGGAPPHQGYMNQPSYYPRHNLNYPQAQPRYGYDARRNIRGHYTY-DNRYYGQFRPGAP  307
             E    PPHQGY++Q  YYPR N+   Q QPRYGYD RRN+R HY Y +NRYYGQ+RP  P
Sbjct  218   EN--PPPHQGYISQAGYYPRQNIYGTQNQPRYGYDTRRNMRPHYPYHENRYYGQYRPTGP  275

Query  308   HHNPGDPYTVRLRVGEREYPGHGYTVQAARHDAAAKAIEHIKQLGVVDDTDHATNTENGQ  367
             H NPGDPYTVRLRVG+REYPG GYTVQAARHDAAAKAIE IKQLG  D TD +   E
Sbjct  276   H-NPGDPYTVRLRVGDREYPGVGYTVQAARHDAAAKAIEDIKQLG--DCTDQSGTVE---  329

Query  368   INDQQTLSNSLTNDVNSELKSPISLVYEISLKRNLNVTFEVLSEKGPPHMKVFVTQCRVG  427
                   + N+ +ND+N+ELKSPISLV+EI+LKRNL+VTFEVLSEKGPPHMKVFVTQCRVG
Sbjct  330   ----APVENTGSNDINTELKSPISLVHEIALKRNLSVTFEVLSEKGPPHMKVFVTQCRVG  385

Query  428   DFLAEGEGNGKKISKKKAAEKMLEELSKLPPLPNVANISQMKRKRVTNKKKTRNLIKVNV  487
             +F+AEGEGNGKKISKK+AAEKMLEEL+KLPPLPN+ N+  +KRKRVTNKKKTRNLIKVN+
Sbjct  386   NFVAEGEGNGKKISKKRAAEKMLEELAKLPPLPNMHNMVHLKRKRVTNKKKTRNLIKVNM  445

Query  488   DKNSETPEEINPISRLIQIQQANKEREPVYTVLEERGAPRRREFVIEASVNGHSCTGVGP  547
             DK+SE  EEINPISRLIQIQQANKEREPVYTVLEERGAPRRREFVIEASVNGHSCTGVGP
Sbjct  446   DKSSEFTEEINPISRLIQIQQANKEREPVYTVLEERGAPRRREFVIEASVNGHSCTGVGP  505

Query  548   NKKVAKRNAADALLTELGYSTTGTKTQPQNKSDKEVDSLIGSNEKNRKVTFVEEKVEQAP  607
             NKK+AKRNAA+ALL +LGY    +  +P  K++KE  S IG  +K RKVTFVEEK E  P
Sbjct  506   NKKIAKRNAAEALLNQLGYGNPPSNPKPVLKTEKE--SEIG--DKTRKVTFVEEKTESTP  561

Query  608   VQSIGGSGGRQLVPGVLLVTEQNAGF--SKPKSDNIE---------PKPVQMQPATMKNP  656
               S+GGSGGRQLVPG+LLV +Q+  F  +KPK   +           K  Q Q  T   P
Sbjct  562   --SVGGSGGRQLVPGILLVGDQSTNFQQNKPKESVMNNNSNSCVDTKKTGQTQMKTPPQP  619

Query  657   PPGVRSKDQLLYLAQLMNIQVQFSDFPKANHEMYLTLVSLGTNPTQVCHGEGPTTEASHE  716
               GVRSKDQL+YLAQLMNIQVQFSDFPKANHEMYLTLVSL TNP QVCHGEGPTTEASHE
Sbjct  620   IQGVRSKDQLMYLAQLMNIQVQFSDFPKANHEMYLTLVSLSTNPPQVCHGEGPTTEASHE  679
```

```
Query  717  KAALEALKVLSELGLDIAP--KDGNAQI  742
            KAALEALKVLSELGLDI    K+G  +I
Sbjct  680  KAALEALKVLSELGLDIVGPNKEGGNEI  707
```

Graphical representation

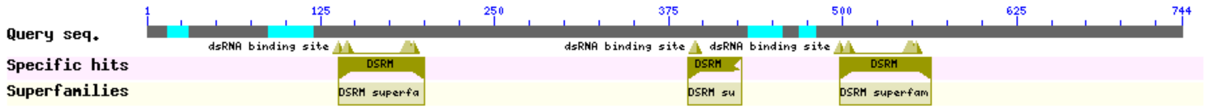

Clp1 homolog (kinase)

>Cb.comp39887_c0_seq2 len=1592

cDNA

AAGATACTCTTAATTCTATGGTATATCTCATTGAATCATGGTAATTTGCATAGAATAAAGATCATTCTATGGTCATTTGAGAGTTTGTGACTGC
CTTCGTGATTGCCTCGTTATTAGATTCAAATGGCATTTAACAAATATATTTTCTTTCATTTAACATATGTTATATGTTATATTTGAACTTTTAT
GAAATTGCGTATATAAAACTGTGTAGTAAATGAAGGTTATATTTTTGGTGTAATAATTTGTTTAACAATGAGTCAAGACAAAAAATCTCTTCTT
CAAGAATTTAAACTCGATTCAGATAATGAATTGCGTTTTGAGGTTGAATCGAAAAACGAAAAAGTTTATCTTACTCTGAAAAGTGGTCTGGCAG
AAGTTTTCGGAACGGAATTGGTAAAGGGAAAAACATATGAATTTACTTCTGGTGCAAAAGTTGCTGTATATACATGGCAAGGCTGTACAATTGA
AGTTAAAGGAAAGACTGATGTAATTTATACTGCTAAAGAAACTCCCATGGTGATTTATTCAAATTGTCATGCAGCTTTAGAGTTAATGAGAATT
GAAGCAGAAAAGGATAACAAACGAGGACCCATTGCTATGGTCGTGGGCCCTGGAGATGTTGGTAAATCTACCGTGTCTAGAATTCTATTGAACT
ATGCTGTTAGGATGGGACGCAGGCCTATTTATGTCGACTTGGATGTGGGTCAAGGGTCTATTTCTATCCCAGGGACAGTAGGTGCCTTGGTCAT
AGAACGTCCAGCAGGGATTGATGAAGGCTTCTCACAGGAAGCACCTTTGGTTTATAATTTTGGACATAAAAGTCCAAATCATAATTCCAAGTTG
TTTAAAATGATTACTGAACAATTGGCAACAATTGTAAAAGAAAGGCTTGAGGTTAACAAAAAAACCCGGCAATCTGGAGTTATAATTAATACTT
GTGGATGGATCAAGGGTGAGGGTTACAAACAAATACTACAGTCTGGGAAGTCCTTTGAAGTAGATGTAATAATGGTATTAGACCAAGAACGTTT
ATATAATGAGTTAGTCAGAGATATGCCAAATTATGTGAAAATAGTTTTCCTTCAAAAAAGTGGTGGTGTTGTTGAACGTTCAAAACACACTAGA
AGTGAAGCAAGAGATCAGAGAATACGGGAATATTTTTATGGACCTCCTAAAAATTCTCTATACCCTCATTCATTTGATGTAAAGTTCTCAGAAG
TCAAAATTTTCAAAATTGGGGCACCTGCCTTGCCAGATTCTTGCTTACCTTTAGGAATGAAGGCAGAGGATCATTTGACAAAAGTTGTACCTAT
TACACCAAATCCAGGGATACTGCATCACATATTGGGTGTAAGCTTTGCAGAAAAAGAAGAAGATGATATTATATTAGCTCATGTCGCTGGTTTT
GTATGTGTATCAAATGTGGACCTCGAAAGACAAACTATCACATTGTTGTCTCCCCAACCAAAGCCTCTACCAAACAATGTTTTGGTACTTTCAG
AAATTCAATTTATGGATAGCCATTAGTTATTGCTCAAGTCTGTACAGTAAATACGTATTTTTGAATACAAATTTTGATAAAAAAAAAA

Protein RF 1: 256->1530 (424AA)

MSQDKKSLLQEFKLDSDNELRFEVESKNEKVYLTLKSGLAEVFGTELVKGKTYEFTSGAKVAVYTWQGCTIEVKGKTDVIYTAKETPMVIYSNC
HAALELMRIEAEKDNKRGPIAMVVGPGDVGKSTVSRILLNYAVRMGRRPIYVDLDVGQGSISIPGTVGALVIERPAGIDEGFSQEAPLVYNFGH
KSPNHNSKLFKMITEQLATIVKERLEVNKKTRQSGVIINTCGWIKGEGYKQILQSGKSFEVDVIMVLDQERLYNELVRDMPNYVKIVFLQKSGG
VVERSKHTRSEARDQRIREYFYGPPKNSLYPHSFDVKFSEVKIFKIGAPALPDSCLPLGMKAEDHLTKVVPITPNPGILHHILGVSFAEKEEDD
IILAHVAGFVCVSNVDLERQTITLLSPQPKPLPNNVLVLSEIQFMDSH

Comparison with *Tribolium* hypothetical protein TcasGA2 TC009961 (406AA)

```
Query  1    MSQDKKSLLQEFKLDSDNELRFEVESKNEKVYLTLKSGLAEVFGTELVKGKTYEFTSGAK  60
            +++DKK+++Q+FKLD DNELRFEVESKNEKVY+TLKSG AEVFGTELVKGKTYEFTSGAK
Sbjct  3    LNEDKKTVIQDFKLDQDNELRFEVESKNEKVYVTLKSGKAEVFGTELVKGKTYEFTSGAK  62

Query  61   VAVYTWQGCTIEVKGKTDVIYTAKETPMVIYSNCHAALELMRIEAEKDNKRGPIAMVVGP  120
            VAVYTW GCTIEVKGKTDV Y AKETPMV YSNCHAALE MRIEAE++NK+GP  M+VGP
Sbjct  63   VAVYTWHGCTIEVKGKTDVSYVAKETPMVTYSNCHAALEFMRIEAERENKKGPTVMLVGP  122

Query  121  GDVGKSTVSRILLNYAVRMGRRPIYVDLDVGQGSISIPGTVGALVIERPAGIDEGFSQEA  180
             DVGKSTV RILLNYAVRMGRRPI+VDLDVGQG ISIPGT+GAL+IERPA IDEGFSQEA
Sbjct  123  NDVGKSTVCRILLNYAVRMGRRPIFVDLDVGQGQISIPGTIGALLIERPASIDEGFSQEA  182

Query  181  PLVYNFGHKSPNHNSKLFKMITEQLATIVKERLEVNKKTRQSGVIINTCGWIKGEGYKQI  240
            PLVY+ GHKSP  N  L+ M                     SGVIINTCGWIKG GYKQI
Sbjct  183  PLVYHTGHKSPQPNIALYSM--------------------ASGVIINTCGWIKGTGYKQI  222

Query  241  LQSGKSFEVDVIMVLDQERLYNELVRDMPNYVKIVFLQKSGGVVERSKHTRSEARDQRIR  300
            L S K+FEVDVI+VLDQERLYNELVRDMPN+VK++FLQKSGGVVERSK  RSEARDQRIR
Sbjct  223  LHSAKAFEVDVILVLDQERLYNELVRDMPNFVKVIFLQKSGGVVERSKSVRSEARDQRIR  282
```

```
Query  301  EYFYGPPKNSLYPHSFDVKFSEVKIFKIGAPALPDSCLPLGMKAEDHLTKVVPITPNPGI  360
            EYFYG PKNS+YPHSFDVK+SE+KI+KIGAPALPDSCLPLGMKAEDHLTK+VP+TPNPGI
Sbjct  283  EYFYGTPKNSMYPHSFDVKWSEIKIYKIGAPALPDSCLPLGMKAEDHLTKLVPVTPNPGI  342

Query  361  LHHILGVSFAEKEEDDIILAHVAGFVCVSNVDLERQTITLLSPQPKPLPNNVLVLSEIQF  420
            LHH+L VSF+E E++DII +HVAGFVCV+NVD +RQ +TLLSPQPKPLPNN+L+LSE+QF
Sbjct  343  LHHLLAVSFSEGEDEDIISSHVAGFVCVTNVDTDRQIVTLLSPQPKPLPNNILLLSELQF  402

Query  421  MDSH  424
            MDSH
Sbjct  403  MDSH  406
```

Graphical representation

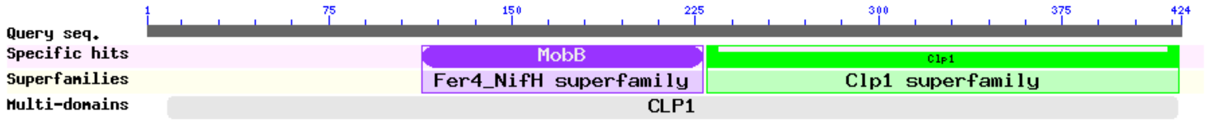

ATP-dependent RNA helicase Belle

>Cb.comp15415_c0_seq1 len=1390

cDNA

```
TTCAAAGCCCATATCCAACATACGGTCGGCTTCGTCTAGCACTAAGTATTTGCAATAGTCCAGGCCAATTCTCCCTCTATCAATCATGTCCAGC
AGACGTCCGGGGGTGGCTACTAGCAAATGGCATCCACGGTCCAGGTCGCGCATTTGATCACCAATATGGGCACCACCGTAAACCACACAAGGGC
GCACTCGCGATCTGTAAGCAAACTTTTTGCTTTCGTCATAGATCTGGGTTGCTAATTCTCTGGTAGGCGCCAAAACAAGACCCAGCGGGTATTG
CTTGCGCCGACTACGACCATGCTGAATGTTCGGCGGGCCCACTTCGTACATCTGATTCAAAATGGGCACCAAAAAGGCTGCGGTTTTACCGGAA
CCGGTCTGAGCGCAAGCCATAACGTCTCTTTTGTTCAAAATAATTGGGATCGCGTATTTCTGCACCGGAGTCGGTGTATCGTAACGAGCGGCCG
CGATATTGCTTCGTATGATTTCGGTCAGCTGTACCTCCTCAAAAGATGTAATGTGGCGCGGGACTTTCTCCCCGGTTGCTTCAACAGGTATATC
TTCATACTTGCTAAAGTTGATGCCCGTATTCCCAGCGCCGAACAGTTCCATTTCGAGGCGTTCATCCCGCGCCAATGGAATGGTCCAATCGTTT
TCATTGCGGCGGTCCCGATTGTCGTTCCACCTGCTATTGCCTCCGTCTCGACTGTTCGAACTTTCGTTCCAACGATCTCTAGGTTTTTCCGGTT
CTTGCCAGCGATCGTTGCGGGACAAGTCACGCTCGCCACTTTCTTGATATCTGCCACCGCTTCTACCCCACTCCTCCTTTTCTCTATTATCAAA
GACCTCTCCATTCGGGAAATTATCGCGTCTGTTTCTGGTATTGAAACTAGAGAAGTCGCCGCGATTCTCGCGAGCGTCGCGGTTTCCTCCGCCC
CTGTTGAAGTTGCGTCCTCCACCGCCGCCTCTGCCCCGTTCCCTATCGTAACCGGAACCTGATTCGGGGTGATTGCCACCGGCGCTACCGCTGT
GTTGCTGTTTATTGCGCAAGTGCGGTGGCACGTAACGGTCGCTTGTTCCGCGACTCTGCAAGTCCAGACCAGCAAACTGCTGCTCTAGACCTGA
TCCATTTTGATTGGGTGCATTACTCATATTACTACAAATCAGTACTTATGGTAGAAGTTTTGATTCTCAAACTTCTAAAATTTATTCTTCAATA
AAATGTCTTCTCTCACTAACACAAAAGCGAATAGTTTATCCTCCACGGAATTTTTTTCGTGAATTACTGCGATTGCGCGATACCCGACTGCAGC
AACTGGTAACACTGGCTGTCAACTAGTTGGACAAATTACAGATCTGGGACATGGGACCAAAAAACTTATTTTGG
```

Protein RF **-2:** -1170->-1 (389AA)

```
MSNAPNQNGSGLEQQFAGLDLQSRGTSDRYVPPHLRNKQQHSGSAGGNHPESGSGYDRERGRGGGGGRNFNRGGGNRDARENRGDFSSFNTRNR
RDNFPNGEVFDNREKEEWGRSGGRYQESGERDLSRNDRWQEPEKPRDRWNESSNSRDGGNSRWNDNRDRRNENDWTIPLARDERLEMELFGAGN
TGINFSKYEDIPVEATGEKVPRHITSFEEVQLTEIIRSNIAAARYDTPTPVQKYAIPIILNKRDVMACAQTGSGKTAAFLVPILNQMYEVGPPN
IQHGRSRRKQYPLGLVLAPTRELATQIYDESKKFAYRSRVRPCVVYGGAHIGDQMRDLDRGCHLLVATPGRLLDMIDRGRIGLDYCKYLVLDEA
DRMLDMGFE
```

Comparison with *Tribolium* ATP-dependent RNA helicase belle (699AA)

```
Query  6    MSNAPNQNGSGLEQQFAGLDLQSRGTSDRYVPPHLRNKQQHSGSAGGNHPESGSGYDRER  65
            MSNAPNQNGSGLEQQFAGLDLQSR  S RYVPPHLRNKQ  + S+          YDR+R
Sbjct  1    MSNAPNQNGSGLEQQFAGLDLQSRAPSGRYVPPHLRNKQSSAESS----------YDRDR  50

Query  66   G--RGGGGGRNFNRGGGNRDARENRGDFSSFNTRNRRDNFPNGEVFDNREKEEWGRSGGR  123
            G  RGG G  N++  GG RD R   GD+SSFNTRNRRDNF NGE F+  E    G  GG
Sbjct  51   GESRGGSGRSNYSSRGGGRDNRA--GDYSSFNTRNRRDNFQNGETFEREEWGRGGGGGGG  108

Query  124  YQESGER-DLSRNDRWQEPEKPRD----RWNESSNS-------RDGGNSRWNDNRDRRNE  171
             ++      DL RNDRWQEPEKPR+    RW+++ N          GG  RWNDNRDR NE
Sbjct  109  GRQQERERDLPRNDRWQEPEKPREGGGGRWSDNRNENRGGGGGGGGGGRWNDNRDRHNE  168
```

```
Query  172  NDWTIPLARDERLEMELFGAGNTGINFSKYEDIPVEATGEKVPRHITSFEEVQLTEIIRS  231
            NDWT+P+ RDERLE ELFG GNTGINFSKYEDIPVEATG+KVPRHITSFEEVQLTEIIR+
Sbjct  169  NDWTVPMPRDERLEQELFGTGNTGINFSKYEDIPVEATGDKVPRHITSFEEVQLTEIIRN  228

Query  232  NIAAARYDTPTPVQKYAIPIILNKRDVMACAQTGSGKTAAFLVPILNQMYEVGPPNIQHG  291
            NI  ARYDTPTPVQKYAIPII+ KRDVMACAQTGSGKTAAFLVPILNQMYE GPPNI HG
Sbjct  229  NINLARYDTPTPVQKYAIPIIVGKRDVMACAQTGSGKTAAFLVPILNQMYEHGPPNITHG  288

Query  292  RSRRKQYPLGLVLAPTRELATQIYDESKKFAYRSRVRPCVVYGGAHIGDQMRDLDRGCHL  351
            RSRRKQYPLGLVLAPTRELATQIYDESKKFAYRSRVRPCVVYGGAHIGDQMRDLDRGCHL
Sbjct  289  RSRRKQYPLGLVLAPTRELATQIYDESKKFAYRSRVRPCVVYGGAHIGDQMRDLDRGCHL  348

Query  352  LVATPGRLLDMIDRGRIGLDYCKYLVLDEADRMLDMGFE  390
            LVATPGRLLDMIDRGRIGLDYC+YLVLDEADRMLDMGFE
Sbjct  349  LVATPGRLLDMIDRGRIGLDYCRYLVLDEADRMLDMGFE  387
```

Graphical representation

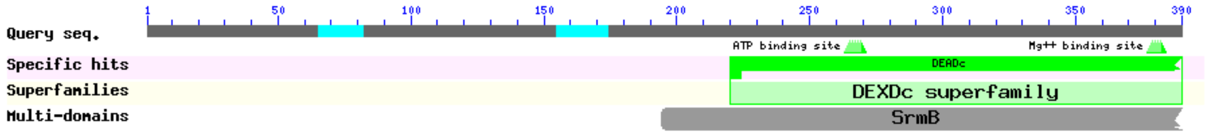

>Cb.comp38184_c0_seq1 len=3212

cDNA

```
GTATGTTGGATATGGGCTTTGAACTCCAAATCCGTCGGATTGTGGAAAAGGAATCAATGCCCAGGACAGGGGAAAGGCAGACTCTTATGTTTTC
AGCCACTTTTCCCCATCCTATTCAAATGTTAGCGCGCGACTTCCTTGATAATTACATATTTTTGGCTGTTGGTCGAGTCGGCTCTACCTCTGAG
AATATCACACAAAAGGTTGTGTGGGTGGAGGAACACGACAAGAGATCTCTTTTGCTGGATCTGTTGAACGTAAATGATTTGCACCAGCCATCGG
CGGAAAGCTTGACTTTGGTATTCGTCGAAACAAAGAAAGGCGCCGACTCACTCGAAGATTTTCTTGATCATGAAGGTTACCCGGTCACTTCGAT
CCACGGCGATCGTACACAACGAGAAAGAGAAGATGCATTGAAACAATTTCGCTCGGGAAATACCCCAATATTGGTTGCCACAGCAGTTGCCGCT
CGTGGTTTGGATATTCCGCATGTTAAACACGTAATCAATTTCGATTTACCATCAGATATCGAGGAATATGTTCATCGAATCGGACGTACTGGCA
GAATGGGTAACCTAGGATTGGCGACTTCGTTTTTCAACGATAGAAACCGTAACTTGGCCAGTGGAATGTTGGACCTGCTGATTGAAGCTAAACA
GGAATGTCCATCTTTTCTCGAAATTGTGGCTTCTGACAGTCGAATGCCAAGCTCTGGCAGAAGGGGTGGCAAGGGCCGTTATGGGGGAGGAGGC
GGTAGTAGTTTTGGCAGTAGAGATTATCGTCAACAGTCTGGCAATTCGAGGGGGAACCAAAGATCAGGTGGTGGAGGATACAGTAACAATGGAT
ATGGTAATGGAGGTGGAAACCATTACGGCGGCGGGGGTATGGACAGAGGAAGCAACTTTGGTGGGAACTACAATTCCAACAATCAAGATGATTG
GTGGTAAACCAAATTCATCCCCGTAGACATTTTTCTCTTACTGAAAATTACAAGCATCTTATTTGTACTTCATGAGTTTTTTTTTAATTAACCT
AGAAAGAATATATTAAAAATCACTGTCATCCAAAAAAAAAATTGTTTTCTAACATTTTTCCCAACTGTAATATATATAATTAAACCACATATTT
ATTAATAAAGAAGTAGATATTAGCTTCATCCTTACAAAATAAGTAACAATTCATGTTCCTTTTATTTTTCACAAAAATTGAACATTTAACAAAA
GTGGTAGCCTCTACCCCATTTCCAGACATCTTGTTAGTGTTTTTATTGTTTCATATCCAATTTTTTTTTTAAATTATATGGGATCTTTCCATGA
ATATATTTTTGCTTTTTTGCCACTTCTCTTTGCACTCTTTAAAAGCACTTAATTCTGCTAAACACCGGCGTTATAGGAAAAGTTACCGAGTTGT
TTGGTTCATTTATTTATATAATTTACTAAATAAAAAAGATTTAAAAAACATTAAAGTTGTAGGTTGATAACAGATGTTTAGTAATTACGGAACG
CGAGTAAGGAAAAAATTGCGGCTGGTTCTCCTCTGTATAAAGTCAATTTAATGTAGTAGCTTGAGTTGCAGATTTTAAGGAGTTAAATTGTGAA
AATTTATAAAATTAATCGGAATTTGACATGAGTAATAAATGATATAAAATAAAATAAAAAATAAAAATAAATGATAATAAACTGAAGTTCAGAA
CAGGAAACAATTTGATATGTATGGTTTTGGATAGATCCTGTATAACTTTTATGGCGACGTTATTATAATTAGTAATTTTGCAGTTTTCATACCC
AAAAGTTTTTTAATAACTTATTTAAAACTGTATTATTATCCATAAAAATACTTTGTTTATTGAAGACTTAAAATACACCGCAACCATTTATATT
TAGTTTTTTTTTAGTTATCTCAATGTTTTGGCTTTAAGAGGAACCAGAAAAAGTTTTTTTGGGTGTTTTCGAATTTGAGAACCAAAAATTAAGG
AGAGGTCCTCTTAAAGGACTGCTTCCTTGTAATATTTTCTTTGTGGTTTTACCTATGTTATTGATTACAGTTTACGGTAATGCATGGTTCTATT
AAGTCTAAGTATTTTAGGTATCTGTTGATATATGGAATGTTCTTCATCAAAACAATGAATATCTTATAAAATCTGCATTCAGCTTGAAAAATTT
TGAAATTATCTGGGTAACAAAGGTAAAATTATATGTGTGGTATTGGAGTGTATAAGACCCCATGCAATAGAAACATTTTCAGATTTTCTTTTAT
TTGAATTTAAAAACCGCATAACCGCACACAGACAAATTGACATATTAAAGGAAAGTAAATTAACATGGTGTATTTTAAGAGTTTGTGTTTCCGT
CTGTATCATTTCTAAGATTTACTTTTTCTTACTAGTGAACATGTCTGCTTGGAATAACTTGATTAAAGTAGGATAATACTATTTAAAGCAAATT
AATTTAATCAGGGGTCCAGGGCTCAAATAGCGTTGCAGTTACAGCTATTTAGAGCGCTAAGAGTATTCTCTAACTTGACTGTGATATTAACGAT
CTATATTGTAATAAACATAGTGCGTTAAAAACCGTGTATATGCCGAGAGGTACGAATGTCAAAAAGCAGTCATTGAGACTCGGTGGATAAGTCT
TAGCGTGTGGTTTATAATATTTTAACTTAACGTTTTTTTTTATTTAGTGTTTAAACAAAGATTTAATCGGCGTTTAATATTGGTTAGAGTGGGC
ACAACATTTTTGTGCCTCTTAAAGTTTTTGCTGCAATATCCAAAATTTGGTTCATTTCAGATGAAATATGTAGTTATCCTGTGATATGGTTAGA
AAATTCGTGCCTATTCTAACGGAATGTGGTTTCACATTCATTTTCAAGTAAGGAGCTGTGACTGCAACTGTGCGAAACGGGTTGCCGGCATGAG
GATAAGTAATTGCAGTTGTAATTTTAATAGTGATACTAGTGCTTTAATTGCTTGTCCTTTATTGCTTAACGACCAACCACAGTAATACAAGTCG
GGGCATGTGTTCAGTGATATTTTAGGCAACCTGTACTTTTGTTTAATGCTATTAAAAAGGTACGGCAAAATGCTCCGATGTTAAGTTCCCCCAC
AACAATTTTATCCTTTTTTAATAATGCATTTACAGTTTGTTCATGTAGCAGTGATTTTGAAGCATACTTGAAATTATATCTAAATAAAAATTTG
AAAGTTAAAAAAAAAAA
```

Protein RF 3: 3->947 (314AA)

```
MLDMGFELQIRRIVEKESMPRTGERQTLMFSATFPHPIQMLARDFLDNYIFLAVGRVGSTSENITQKVVWVEEHDKRSLLLDLLNVNDLHQPSA
ESLTLVFVETKKGADSLEDFLDHEGYPVTSIHGDRTQREREDALKQFRSGNTPILVATAVAARGLDIPHVKHVINFDLPSDIEEYVHRIGRTGR
MGNLGLATSFFNDRNRNLASGMLDLLIEAKQECPSFLEIVASDSRMPSSGRRGGKGRYGGGGGSSFGSRDYRQQSGNSRGNQRSGGGGYSNNGY
GNGGGNHYGGGGMDRGSNFGGNYNSNNQDDWW
```

Comparison with *Tribolium* ATP-dependent RNA helicase belle (699AA)

```
Query  1     MLDMGFELQIRRIVEKESMPRTGERQTLMFSATFPHPIQMLARDFLDNYIFLAVGRVGST  60
             MLDMGFELQIRRIVEKE+MP+TGERQTLMFSATFP PIQMLARDFLDNYIFLAVGRVGST
Sbjct  381   MLDMGFELQIRRIVEKETMPKTGERQTLMFSATFPSPIQMLARDFLDNYIFLAVGRVGST  440

Query  61    SENITQKVVWVEEHDKRSLLLDLLNVNDLHQPSAESLTLVFVETKKGADSLEDFLDHEGY  120
             SENITQKVVWVEEHDKRS LLDLLN   ++ QPSAESLTLVFVETKKGADSLE+FL  EGY
Sbjct  441   SENITQKVVWVEEHDKRSFLLDLLNAAEMSQPSAESLTLVFVETKKGADSLEEFLHFEGY  500

Query  121   PVTSIHGDRTQREREDALKQFRSGNTPILVATAVAARGLDIPHVKHVINFDLPSDIEEYV  180
             PVTSIHGDR+QREREDAL+QFRSGNTPILVATAVAARGLDIPHVKHVINFDLPSDIEEYV
Sbjct  501   PVTSIHGDRSQREREDALRQFRSGNTPILVATAVAARGLDIPHVKHVINFDLPSDIEEYV  560

Query  181   HRIGRTGRMGNLGLATSFFNDRNRNLASGMLDLLIEAKQECPSFLEIVASDSRMPSSGRR  240
             HRIGRTGRMGNLGLATSFFNDRNRNLASG+LDLLIEAKQE PS+LE VA+D RMPSSGRR
Sbjct  561   HRIGRTGRMGNLGLATSFFNDRNRNLASGLLDLLIEAKQEYPSWLEGVAADGRMPSSGRR  620

Query  241   GGKGRYGGGGGSSFGSRDYRQQSGNSRGNQRSGGGGYSNNGYGNGGGNHYGGGGMDRGSN  300
             GGK RYGGGGGSSFG RDYRQQSG    NQRSGGGG       G G        GG+DRG N
Sbjct  621   GGKSRYGGGGGSSFGGRDYRQQSGGMSRNQRSGGGG-GYGNNGFGNNGGGHYGGLDRGGN  679

Query  301   FGGNY----NSNNQDDWW  314
             FGGNY    NSN++DDWW
Sbjct  680   FGGNYNSNSNSNSRDDWW  697
```

Graphical representation

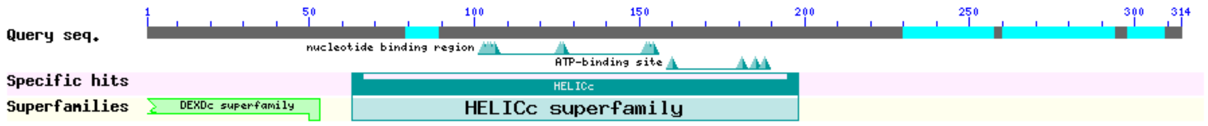

Drosophila homolog of p68 RNA helicase = ATP dependent RNA helicase p62 (Tribolium)

>Cb.comp35296_c0_seq1 len=1998

cDNA

```
GTTTTATAATTCGCTCAAACGAGCATCTCTACTCTGATTCTCTTTTCATTTGGACGTCGAGGAGATCCCTGATCACTTCATTGTCCTTTCGCAA
AGGAATAATCGACTTTAATATCAAGAGAAATATACTTCTATCTACAGCGATAAATATGTCATACAGTAGACAAAATGGAAGCAGCTATAGAAGT
CGGGAAGATGGTTATAATACAAGTTTAAGAGATAAAGGCTATGGGCAGAGAAATGGCTATGATGGTGGTGGTTACAGAAACAGTAGAGCAGGGT
TCAACAATCGTCTTAAAACTGTCGAATGGAGTAGCAAGCAGTTGCGCCCATTCAAAAAAGATTTTTATGTGCCACACCAGTCTATTTCTAACCG
TTCTACTTATGAAGTAGATCAATTCCGTGGTGCAAAAGAAATTACTGTAGAGGGTGACGCACCAAAGCCTATCCAAAGTTTCCATGAAGCCAAT
TTTCCAGATTATGTTATGGACGAAATTGTATCTCAAGGTTATGAATTTCCTACTGCTATTCAGGCCCAAGGGTGGCCGATCGCCATGAGTGGGC
ACGACATGGTTGGGGTTGCTCATACCGGTTCGGGCAAAACTTTGGCTTATATGTTACCTGCCATAGTTCACATAAACCATCAGCCAGAGGTACA
ACGCGGTGATGGTCCGATCGTGTTAGTCTTGGCCCCTACCAGAGAATTAGCTCAGCAGATCCAGCAAGTCGCTAATGATTTTGGACGTAGCTCA
AAAATTCGAAATTCCTGTGTATTCGGTGGTGCTCCTAAAGGACCGCAGGCCCGAGATTTGGAAAGGGGCGTCGAAATTTGCATCGCGACCCCTG
GCCGGTTGATAGACTTTTTGGAAAAAGGCACGACCAACTTGGAGAGATGTACTTACTTGGTCCTGGATGAAGCTGACCGTATGTTAGACATGGG
TTTCGAACCTCAGATTAGGAAAATCATTGGACAGATCAGGCCCGATAAACAAACGCTGATGTGGTCCGCTACTTGGCCTAAGGAGGTCAAAAAA
TTGGCTCAAGATTTCATGAATAACCCAATTCAGCTTAATGTTGGCTCGCTTCAGTTGTCCGCCAACCACAACATTTTGCAAATTGTAGATGTAT
GCCAAGAACATGAAAAGGAAACTAAGTTAAACAATCTGTTGCAAGAAATTGGCACAAATGGGGAACCAGACGCAAAAATAATTATTTTCGTCGA
AACTAAAAAGAAGGTGGAGGGCATCACCAGGACCATAAGAAGACTCGGTTGGCCCGCAGTTTGCATGCACGGCGACAAGAGCCAGCAGGAACGA
GATTATGTTCTGCGAGAATTTAGGAATGGAAAGTCTACTATATTGGTCGCTACTGATGTGGCTGCTCGTGGATTAGATGTGGACGGTATAAAAT
ATGTAGTAAACTACGACTATCCCAACTCGTCAGAGGATTATATCCATAGAATAGGGCGAACTGGTCGATCTGATTCTACTGGCACCTCATATGC
ATTCTTCACTCCATCGAACATAAGGCAGGCTAAAGATTTGGTCTCAGTGCTAAAGGAAGCAAACCAGGTTGTCAACCCAAAATTATCAGAAATG
```

GCAAGCAAATCAAGTGCTTATGGAGGTTTCCAAAGAACCGGCCGTTGGGGTAATGGAGGTGGTTCCTATAGGGGTAGGGAAAACAGTGGACCAA
AACACAGCAGATGGGGGGGCAGTTCTGGTGGATACAAGGCGAGCAATGGATATGGAAAAAGCTACTGAAAGACATTTGATTACGGACATTTCAT
TCAATATCAGTATTTTCCTTCACATATTCCACACTAGGACAATTGAATTTTTCTAATTTAGTTAGAATTTTATTTAAAAAAAAACGTTTGTATT
TATTTGACAATCTCTCTCTGTCATGATTGTAAATCTGTTTATAACTGTGATAGAAGTAGATTTCTATGTGATTTTGAAAATGTAAGAATAAAAG
TTTTTTTTATTTGTAAAAAAAAAA

<u>Protein RF 3:150 -> 1760(536AA)</u>

MSYSRQNGSSYRSREDGYNTSLRDKGYGQRNGYDGGGYRNSRAGFNNRLKTVEWSSKQLRPFKKDFYVPHQSISNRSTYEVDQFRGAKEITVEG
DAPKPIQSFHEANFPDYVMDEIVSQGYEFPTAIQAQGWPIAMSGHDMVGVAHTGSGKTLAYMLPAIVHINHQPEVQRGDGPIVLVLAPTRELAQ
QIQQVANDFGRSSKIRNSCVFGGAPKGPQARDLERGVEICIATPGRLIDFLEKGTTNLERCTYLVLDEADRMLDMGFEPQIRKIIGQIRPDKQT
LMWSATWPKEVKKLAQDFMNNPIQLNVGSLQLSANHNILQIVDVCQEHEKETKLNNLLQEIGTNGEPDAKIIIFVETKKKVEGITRTIRRLGWP
AVCMHGDKSQQERDYVLREFRNGKSTILVATDVAARGLDVDGIKYVVNYDYPNSSEDYIHRIGRTGRSDSTGTSYAFFTPSNIRQAKDLVSVLK
EANQVVNPKLSEMASKSSAYGGFQRTGRWGNGGGSYRGRENSGPKHSRWGGSSGGYKASNGYGKSY

<u>Comparison with *Tribolium* (549AA)</u>

```
Query  1    MSYSRQNGSSYRSR--EDGYNTSLRDKGYGQRNGYDGGG-YRNSRAGFN-----------  46
            MSY +QNG SYR R  E+G+       G   RNG+ GG  ++N   G
Sbjct  1    MSYGKQNGGSYRGRGSENGFG------GGASRNGFGGGSRFKNGGGGGGGGSRFGGRSGGG  54

Query  47   ----NRLKTVEWSSKQLRPFKKDFYVPHQSISNRSTYEVDQFRGAKEITVEGDAPKPIQS  102
                NRL+   W  K LRPFKKDFYVPH +++NRS YEV+Q+R +KEIT++GDAP PIQ+
Sbjct  55   GSPGNRLRKPNWDMKNLRPFKKDFYVPHPAVANRSKYEVEQYRRSKEITIDGDAPNPIQN  114

Query  103  FHEANFPDYVMDEIVSQGYEFPTAIQAQGWPIAMSGHDMVGVAHTGSGKTLAYMLPAIVH  162
            F EA FPDYV  EI  QGY+ PTAIQAQGWPIAMSG D+VG+A TGSGKTLAY+LPAIVH
Sbjct  115  FEEACFPDYVQHEIQKQGYDTPTAIQAQGWPIAMSGKDLVGIAQTGSGKTLAYILPAIVH  174

Query  163  INHQPEVQRGDGPIVLVLAPTRELAQQIQQVANDFGRSSKIRNSCVFGGAPKGPQARDLE  222
            IN+QP + RGDGPI LVLAPTRELAQQIQQVA+DFG SS +RN+C+FGGAPKGPQARDLE
Sbjct  175  INNQPSIARGDGPIALVLAPTRELAQQIQQVAHDFGSSSYVRNTCIFGGAPKGPQARDLE  234

Query  223  RGVEICIATPGRLIDFLEKGTTNLERCTYLVLDEADRMLDMGFEPQIRKIIGQIRPDKQT  282
            RGVEICIATPGRLIDFLEKGTTNL+RCTYLVLDEADRMLDMGFEPQIRKII QIRPD+QT
Sbjct  235  RGVEICIATPGRLIDFLEKGTTNLQRCTYLVLDEADRMLDMGFEPQIRKIIEQIRPDRQT  294

Query  283  LMWSATWPKEVKKLAQDFMNNPIQLNVGSLQLSANHNILQIVDVCQEHEKETKLNNLLQE  342
            LMWSATWPKEV+KLAQDF+ N +Q+N+GSLQLSANHNILQIVDVCQEHEKETKLNNLLQE
Sbjct  295  LMWSATWPKEVRKLAQDFLRNYVQINIGSLQLSANHNILQIVDVCQEHEKETKLNNLLQE  354

Query  343  IGTNGEPDAKIIIFVETKKKVEGITRTIRRLGWPAVCMHGDKSQQERDYVLREFRNGKST  402
            IG NGEP AKIIIFVETKKKVE ITRTIRR GWPAVCMHGDKSQQERD+VLREFRNGKS+
Sbjct  355  IGNNGEPGAKIIIFVETKKKVESITRTIRRYGWPAVCMHGDKSQQERDFVLREFRNGKSS  414

Query  403  ILVATDVAARGLDVDGIKYVVNYDYPNSSEDYIHRIGRTGRSDSTGTSYAFFTPSNIRQA  462
            IL+ATDVAARGLDV+GIKYV+NYDYPNSSEDYIHRIGRTGRSD+TGTSYAFFTPSN RQA
Sbjct  415  ILIATDVAARGLDVEGIKYVINYDYPNSSEDYIHRIGRTGRSDTTGTSYAFFTPSNFRQA  474

Query  463  KDLVSVLKEANQVVNPKLSEMASKS---SAYGGFQRTGRWGNGGGSYRGRENSGPK-HSR  518
            KDLVSVLKEANQ +NP+LSEMA++     + GG    G     GGS+RGRENSGP+ H R
Sbjct  475  KDLVSVLKEANQAINPRLSEMANRCSSYGSKGGSGGGGGRWGYGGSFRGRENSGPRNHQR  534

Query  519  W--GGSSGGYKASNG  531
            +  GG S GY   SNG
Sbjct  535  FTNGGRSNGY--SNG  547
```

<u>Graphical representation</u>

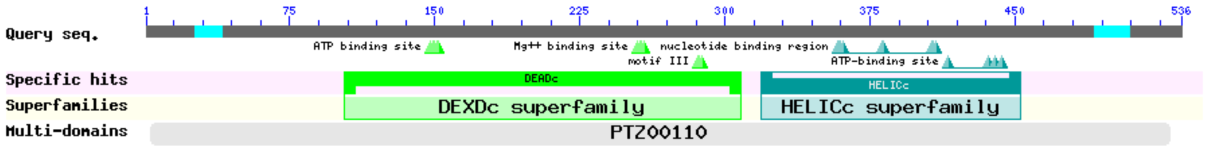

>Cb.comp33294_c0_seq1 len=2248

<u>cDNA</u>

GGTTACATTTCCGACAAAAATTGAAAATGAAAGACCAGCCCAGTTCCTCACATAAATTCAAATAAAATGCAATAATTGTAATTTTTTAATGTGA
AATACATGGAAAGTGAAGGAATCTGCTTCTATCACAGTTACAAAAAATTATAGTCTTTAAATAATAAATACAAAAATTATCAACTAAACAATTA

AATAGTAGTCTTTCAAACCAATAATACTTTTTAAAGCAAGACAAATCTTGAGTTGTTTAGTAAAGGAAAATAAAATGGTATCATATCCCAGTTG
CTATACTAAAATATAGTGTTGATAGGTGCAGCGAGATCTTCAGAACCCTTAATAACTTTTGGGGTAGTATCCTTGGTTTGAATGGAATCCATTA
TTGAACCTGGGAAACTGCTTTTGGCCCCCACCATAACCACCTCCCCAACGACTCGCCTTGGATGCCGCGAAGCCTGATTTGTTGGCAATTTCGT
TTAGTTGGGGATTCACAAACTGCTTGGTTTCCATCAGCATGGAGACAAGATCTTTGGCATGCTTGAAGTTTGATGGTGTGAAAAAGGCGTAGGA
CGTCCCGGTAGTATCAGATCGACCGGTTCTGCCAATTCTGTGTATATAGTCCTCTGACGAATGCGGGTAGTCGTAATTGACTACGTATTTATA
CCTTCTACATCAAGACCTCTTGCTGCGACATCAGTCGCTACAAGAATAGCGGACTTCCCACTTCTGAACTGATGAATTACACTGTCACGTTCTT
GTTGACTTTTATTACCATGAATAGAAATCGCGGGCCAGCCCGTCCTTCTTATAGTGTTGGTTATTGCCTCAACTTTCTTCTTGGTCTCAACAAA
AATCAAGATCTTCGCTTCCAACTCCGCGCCAATCTCTTGCAAAAGCTGGCTGAGTTTGTTTTCTTTCTCATGTTCTTGGCATACATCCACAATC
TGATTGATGTTGGGGTTGGCTGACAGTTGTAACGACCCAACATTCAGTTGGACATAATTTTTCATAAAATCAGAAGCTAGTTTTTTAACCACAG
TTGGCCAGGTTGCCGACCACATTAGTGTCTGCTTATCAGGCCTAATTTGGCTAATAATTTTGCGGATTTGAGGTTCAAAGCCCATATCCAACAT
ACGATCTGCTTCATCGAGCACCAAATAAGTGCACCTTTCCAAATTGGTGGTGCCTCTTTCCAAAAAATCAATCAACCTGCCTGGAGTCGCAATG
CATATTTCAACACCCCTTTCCAAATTGCGGGCTTGAGGAATTTTTGAGGCACCTCCAAATATACAACAATTACGAATTTGAGAGGTTTGACCAA
AATCATTGGCGACTTTTTGTATTTGTTGCGCTAATTCTCTCGTCGGCGCCAAAATCAAAGCAATCGGACCTTCGCCGCGCCTTACCGAAGATTG
ATTGTTGATATGAACAATAGCAGGTAATATATAAGCCAATGTTTTGCCAGATCCAGTTTGGGCAATTCCGACCATATCACGTCCACTCATAGCA
ATAGGCCAACCCTGAGCCTGAACGGATGTTGGATATTCATAACCCTGTGCTACAATTTCATCCATAACATAGTCTGGAAAGTTTGCCTCGTGGA
AATTCTGAATTGGTGCTGGAATATTATCGCCCTCTACCATAATTTTTTTGCCATCTCGGAATTGATCAACTTCATAGGAAGATCGGTTTGTGAT
AGTGGGGTGAGGGATGTAGAAGTCTTTTTTAAAAGGATGCAATTGCTTGCTACCCCATTCAACCTCCTGCAACTCTTTAGGAATACCTTCAGAT
TGGTAATTATTACCAAACCTATTTCCATTATTTCCACCCCAATTGTTTCGCTGAACAAAATTGTTTGCGTAATTATTTGAACGGAATCCGTTTT
GATGACCTCTAAACTGGCCACCGCCATTTTGTCTATCGTAAGACATAATACTGAAATCAACTGAAGCGATTTCTTCAAGGATTTCTAAATTTTA
TATTTCTCGGCAAGTGAACAAGAAGAGAAAGTTGAAATCTCTTCTAGGAACAATTGAAAAGAGAAATTTCGGGATGAAGCGATGATGCCGGAAA
TGACATTCGTCGCCCGCATGCAACGTTGTCGGTTCTAGCTTTATAAAGAATTGTAGTTCTATTCACAAGAAAAAGTTTTAATACCTACGTTACA
AGCAGTCAAAAAGTTACTAGTAAATTGAAATTGATTTATTGTATATCGATTCCAATTAACGTATTTGTGAAAACTTAGAATAATTT

Protein RF -2 : -1926->-331 (531AA)

MSYDRQNGGGQFRGHQNGFRSNNYANNFVQRNNWGGNNGNRFGNNYQSEGIPKELQEVEWGSKQLHPFKKDFYIPHPTITNRSSYEVDQFRDGK
KIMVEGDNIPAPIQNFHEANFPDYVMDEIVAQGYEYPTSVQAQGWPIAMSGRDMVGIAQTGSGKTLAYILPAIVHINNQSSVRRGEGPIALILA
PTRELAQQIQKVANDFGQTSQIRNCCIFGGASKIPQARNLERGVEICIATPGRLIDFLERGTTNLERCTYLVLDEADRMLDMGFEPQIRKIISQ
IRPDKQTLMWSATWPTVVKKLASDFMKNYVQLNVGSLQLSANPNINQIVDVCQEHEKENKLSQLLQEIGAELEAKILIFVETKKKVEAITNTIR
RTGWPAISIHGNKSQQERDSVIHQFRSGKSAILVATDVAARGLDVEGIKYVVNYDYPHSSEDYIHRIGRTGRSDTTGTSYAFFTPSNFKHAKDL
VSMLMETKQFVNPQLNEIANKSGFAASKASRWGGGYGGGQKQFPRFNNGFHSNQGYYPKSY

Comparison with *Tribolium* (AA)

726 bits(1874) 0.0 Compositional matrix adjust. 371/559(66%) 428/559(76%) 38/559(6%)

```
Query  1    MSYDRQNGGG-QFRGHQNGFRSNNYANNFVQRNNWGGNNGNRFGNNYQSEGIP-------  52
            MSY +QNGG  + RG +NGF           RN +GG  G+RF N     G
Sbjct  1    MSYGKQNGGSYRGRGSENGFGGG------ASRNGFGG--GSRFKNGGGGGGGSRFGGRSG  52

Query  53   ------KELQEVEWGSKQLHPFKKDFYIPHPTITNRSSYEVDQFRDGKKIMVEGDNIPAP  106
                    L++  W  K L PFKKDFY+PHP + NRS YEV+Q+R  K+I ++GD  P P
Sbjct  53   GGGSPGNRLRKPNWDMKNLRPFKKDFYVPHPAVANRSKYEVEQYRRSKEITIDGD-APNP  111

Query  107  IQNFHEANFPDYVMDEIVAQGYEYPTSVQAQGWPIAMSGRDMVGIAQTGSGKTLAYILPA  166
            IQNF EA FPDYV  EI  QGY+ PT++QAQGWPIAMSG+D+VGIAQTGSGKTLAYILPA
Sbjct  112  IQNFEEACFPDYVQHEIQKQGYDTPTAIQAQGWPIAMSGKDLVGIAQTGSGKTLAYILPA  171

Query  167  IVHINNQSSVRRGEGPIALILAPTRELAQQIQKVANDFGQTSQIRNCCIFGGASKIPQAR  226
            IVHINNQ S+ RG+GPIAL+LAPTRELAQQIQ+VA+DFG +S +RN CIFGGA K PQAR
Sbjct  172  IVHINNQPSIARGDGPIALVLAPTRELAQQIQQVAHDFGSSSYVRNTCIFGGAPKGPQAR  231

Query  227  NLERGVEICIATPGRLIDFLERGTTNLERCTYLVLDEADRMLDMGFEPQIRKIISQIRPD  286
            +LERGVEICIATPGRLIDFLE+GTTNL+RCTYLVLDEADRMLDMGFEPQIRKII QIRPD
Sbjct  232  DLERGVEICIATPGRLIDFLEKGTTNLQRCTYLVLDEADRMLDMGFEPQIRKIIEQIRPD  291

Query  287  KQTLMWSATWPTVVKKLASDFMKNYVQLNVGSLQLSANPNINQIVDVCQEHEKENKLSQL  346
            +QTLMWSATWP  V+KLA DF++NYVQ+N+GSLQLSAN NI QIVDVCQEHEKE KL+ L
Sbjct  292  RQTLMWSATWPKEVRKLAQDFLRNYVQINIGSLQLSANHNILQIVDVCQEHEKETKLNNL  351

Query  347  LQEIG--AELEAKILIFVETKKKVEAITNTIRRTGWPAISIHGNKSQQERDSVIHQFRSG  404
            LQEIG   E  AKI+IFVETKKKVE+IT TIRR GWPA+ +HG+KSQQERD V+ +FR+G
Sbjct  352  LQEIGNNGEPGAKIIIFVETKKKVESITRTIRRYGWPAVCMHGDKSQQERDFVLREFRNG  411

Query  405  KSAILVATDVAARGLDVEGIKYVVNYDYPHSSEDYIHRIGRTGRSDTTGTSYAFFTPSNF  464
            KS+IL+ATDVAARGLDVEGIKYV+NYDYP+SSEDYIHRIGRTGRSDTTGTSYAFFTPSNF
Sbjct  412  KSSILIATDVAARGLDVEGIKYVINYDYPNSSEDYIHRIGRTGRSDTTGTSYAFFTPSNF  471

Query  465  KHAKDLVSMLMETKQFVNPQLNEIANKS------------GFAASKASRWGGGYGGGQKQ  512
            + AKDLVS+L E  Q +NP+L+E+AN+             G        + G    G +
Sbjct  472  RQAKDLVSVLKEANQAINPRLSEMANRCSSYGSKGGSGGGGGRWGYGGSFRGRENSGPRN  531
```

```
Query  513  FPRFNNGFHSNQGYYPKSY  531
             RF NG   SN GY    SY
Sbjct  532  HQRFTNGGRSN-GYSNGSY  549
```

Graphical representation

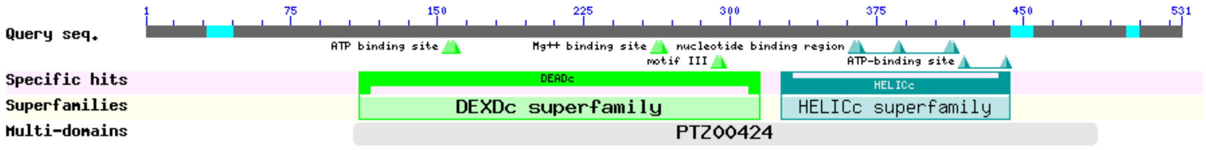

Gemin3 homolog

>Cb.comp41450_c0_seq3 len=3329

cDNA

GTTTGTTGAAACTTAAAAACATTGCTTAGGTTAAGAGATATCTTTTTATATAATATTTAATCTTATAATTGCACCAAAACTCAATGCACTATTC
CAGATCAATTTAAAACAACAGCTTTTTGTTCATTTTTGTTTATTTGTACTCCCACACTGTGCCAATGAAATATTTTCTTTTCATCACATATGTC
GGTTACAAATAAAATAAAAAAAATTTTGATTTGCTTGCAAGTTTATTGAATGGAAGTTTAAAAATACCAGCAAACTGAAATATCTCGCAAATAT
AGATAATTTTATTTACAATAATTACTATAGATAACCAGTCTGTTTTTACAAAGGCAATATTGAGAATAGTTTGTTGTAATAACTTTAATAATCC
ATGTACGCGTAGACATACATCCCTATTTCACTGTTGAAATTAGAAGTACATTCTAATACTCATAACTGCAATTTATATATAAGGATCTGCTTTT
TCTATTCTAACACCACACTTTTGTTGAATGTGTGCCATTTCTTCAACATAAATTTTTTGTTGAATACAATTTCTTACTGCTGAAACTTGAGCTT
GCCAGCCATAGCAGAACCACTGATCAAAATGTTCCACAGTTTCAAAAGACAACCCAGTTTGCCACAGATTTTCACTACAATGCCTAAAATATTC
TCCATATTGATCGCAATACTTCTCCATGTAAGATGGAGCTATACTTTGATTTTCAGATTCTGTTGTGTTATTAATGATAAAATTATTTTGAATG
ACTGGCATGCACTCATTAGATTTCTGAGACTTTGCTTCAACGGGGATCCATTTGAGACAATTAAAATCACCAACAGGCTGCATGTCATAATTTA
TTTCAACTTCTTTTTCTAACTCAAAGTTGTATTCCTCATCCTCCTCATATTCCTCTTCAAGCGCATCTTCATCCATGTCATCTTGGCAATTGTC
ATCGTCTGGGTGACCAGTTACTATTTTCATTCTTTCTTCTTCAGGAAGCAACTCTTGCCAATGAACTGATGAAGAGTTTATAGCAAACTGATAT
GCCATTGGGAAAATATTCTCCAAAACTTGTTCTTGGGAACACTTTTCTTGTCTTAACTCATTGGCCAATACCTTGCATTCCTGTAAATAATCCT
CATATCTTCTAGAATTCAGATACTGACCTATTAATTGAATGTCATCTTGATCTGTTTCACAGTTGCTTAGAGATTTGGCCACACTAAATAGAGT
TTTATTTTTTTTGTAAATAACATTTTCAGAATTTGAGTTTGAGGTGCTATCGTGAATAGCTAAATTTGCATTATTAACAGTCTCACCCTTATCA
GCTAGTTCTGTTAATATTTTTATTGTATCTATATCCTCACCTTGATGTCCTTCATTGCTTGGTATATTGTAGGCATCTGATGTTAATTGATTTA
ATAATTCAGAACCATCTATTTTGCAATTTGAATCATCTGAACTGCTATTGCTTAATATTTTGCTCAGTTCAAAATTGCCTTCTGCTAACTGCCC
CAAAATTGATGTTGTGTCAATATCTGTCAAGTGCTTATCAACAATTCCTTTAACTTTATCTAAATCTTCTTTTTTGTTCTTAATTTTCCTATTT
TTAATAGGCTTTACTTTCAGTTTACTTAGTTCTGCTTTTAAGTGGTTAATTGCTTCATTATTTTTTGGCACAATTCCAAAGAGCAAATTCTCTT
TAGATAAATCTGTTTCCTTTAGATTAAATGAGTCATCTAATATAGGTATCGACAAAGTGGTTCCCCCAATACTACCCATTATATTTTGCAACAA
CTCAACCTCTTGCCCTTCAGATGCCAAATTAATGCAAATACCTGTAGAACCATATCTGCCAGCCCTTCCCATACGATGTAAATATGTCATGACA
TCTTTCGGAATGTCATAATTTATAACTAAGTCTACATTAGCTACATCAATACCCCGAGCTGTAAGGTCTGTAGACAACAATATTCGAAACTTGA
AATCCTTTAAATTAGAAATTGCATTCAGTCTGTCATTTTGATTTTGAGCTCCAGATATATAAGTACAACTCCATCCATTGCGGTTAAGAAAATT
ACTTGTGCTTTCTGCTCGGGTTTGATAATTTGTAAATACTAAACACTGGGTAAAGGGTGTTACTGCAAAGATTTTTAATAAATTATCATTCTTA
ACTTTAGTTTGTTGCACAATATTATTTGCAAATTTCGTGACTCTCACAAACTGTTTAAGCCCCAAGAGCAATGGAGTTTCTAATTCAGCACTAA
CAAAAGTTGGGCTAAGCATATAATTAGTTAAAAGTGTCTCTAATTCCTTTGTATATGTGGCGCTACACATTATAACTTGTTTTTTTGAAGGCAA
ACTGTTATAAATTTCATTCACATCACTTTCAAAGCTACTCTCCATGAGCTTATCAGCCTCGTCAAGTACAAAAAGCTTAACAAAACTTACGGTG
AGACTACCAATTTTTATAAGGTGTTTAAGTCTGCCAGGAGTACCAATAGCTATATGACATGATCTACATTTAGGTTTATCCTGCTCTATTGAGA
ATCCTCCGATAAAACACTCTACAATTAATCCATCAAAATAACGTCCAATATCCTTAAATACATCCCCAATTTGCACTGCAATCTCTCTTGTCGG
GGTAACAATTAAAATTTGTGGCACCTTTTTTGCGATATCTATAGCTTCTAATGCTATCACTGTAAATACCAATGTTTTGCCAGTGCCAGATTTC
GATTTCACCAACAAATCGAATCCACATTTTCCTAATGGAATTGCTTTAATTTGAATAGGAGAAGCTTTCTGAAACCCGTTATCAATCAACCCTT
TGAGAATACTGCCCGGCAAAACTAGAGATTCGAATGATACATTTTCTTCGGTAATAACATCCCTGGTTCTCGTTTTATCCTCCAGTGAATGGGC
TAATTTACAATAACAAAATTTACATTAAGGAATTCCTGGAAAAAAATACCCCATCAATTATTTACAATGTTATGAGGCATTCTACTAGAAAATT
AGGCTTCTTCTAGCCGCTGGTCCTACACAAAATAAAAAATTTTGTAAGGTAAATTGCAAAAACAATAACAAGGTATTTTGATATTCGTTTTCGC
GGAAATTTTAAAAGTTCACGTTCTTTGTATTCAGAAATGGAGGAACTAAAGGTATACTACACAAACGTCGCTTAATAATCTGTGAATATTTCAA
GAAATCGATAACCTAAATTGTTTTAGGTTTCCATTTTACAGTATGATCTCTTTAATCCCTGTACGTGCTAATCCCTATATCCTGCAACAATTAC
ACAGCTCGAACATTTTTAAATTAAATTATTTAGTTAAAG

Protein RF -2: -2395-> -449(648AA)

MESSFESDVNEIYNSLPSKKQVIMCSATYTKELETLLTNYMLSPTFVSAELETPLLLGLKQFVRVTKFANNIVQQTKVKNDNLLKIFAVTPFTQ
CLVFTNYQTRAESTSNFLNRNGWSCTYISGAQNQNDRLNAISNLKDFKFRILLSTDLTARGIDVANVDLVINYDIPKDVMTYLHRMGRAGRYGS

```
TGICINLASEGQEVELLQNIMGSIGGTTLSIPILDDSFNLKETDLSKENLLFGIVPKNNEAINHLKAELSKLKVKPIKNRKIKNKKEDLDKVKG
IVDKHLTDIDTTSILGQLAEGNFELSKILSNSSSDDSNCKIDGSELLNQLTSDAYNIPSNEGHQGEDIDTIKILTELADKGETVNNANLAIHDS
TSNSNSENVIYKKNKTLFSVAKSLSNCETDQDDIQLIGQYLNSRRYEDYLQECKVLANELRQEKCSQEQVLENIFPMAYQFAINSSSVHWQELL
PEEERMKIVTGHPDDDNCQDDMDEDALEEEYEEDEEYNFELEKEVEINYDMQPVGDFNCLKWIPVEAKSQKSNECMPVIQNNFIINNTTESENQ
SIAPSYMEKYCDQYGEYFRHCSENLWQTGLSFETVEHFDQWFCYGWQAQVSAVRNCIQQKIYVEEMAHIQQKCGVRIEKADPYI
```

Comparison with *Tribolium* hypothetical protein TcasGA2 TC003675 (688AA)

```
Query  1    MESSFESDVNEIYNSLPSKKQVIMCSATYTKELETLLTNYMLSPTFVSAELETPLLLGLK  60
            ME SF+SD+NEIYNSLP +KQ+I+ SATY +EL+T L NYM SPT V++E ETPLLLGLK
Sbjct  179  MEESFQSDINEIYNSLPPRKQMIVSSATYPQELDTFLANYMQSPTHVTSENETPLLLGLK  238

Query  61   QFVRVTKFANNIVQQTKVKNDNLLKIFAVTPFTQCLVFTNYQTRAESTSNFLNRNGWSCT  120
            QF  + +    N VQQ K+KND L+ I      F QCLVFTNYQ+R E+ SN+LN+ GW
Sbjct  239  QFAAMLRPGLNSVQQMKIKNDLLITILTKVSFVQCLVFTNYQSRTETVSNYLNQKGWDSV  298

Query  121  YISGAQNQNDRLNAISNLKDFKFRILLSTDLTARGIDVANVDLVINYDIPKDVMTYLHRM  180
            +IS AQ Q +RL AI NLK FK RILLSTDLT+RGID  NVDLVINYD+P D +TYLHRM
Sbjct  299  FISAAQKQTERLEAIDNLKKFKNRILLSTDLTSRGIDAPNVDLVINYDLPCDAVTYLHRM  358

Query  181  GRAGRYGSTGICINLASEGQEVELLQNIMGSIGGTTLSIPILD--DSFNLKETDLSKENL  238
            GRAGRYGS G+CIN  SEG EV  LQ+I+G+IGG  LSI  L    +  +L + DL
Sbjct  359  GRAGRYGSGGLCINFVSEGPEVTKLQHILGAIGG-NLSIAKLPPLEGVDLWQVDLKTLEQ  417

Query  239  LFGIVPKN--NEAINHLKAELSKLK-VKPIKNRKIKNKKEDLD  278
            + G+VP +  N+   +LK+E+ +LK  K  K RK  N +  D
Sbjct  418  IRGVVPSDTSNDVSENLKSEVMELKERKDGKKRKRVNPEASPD  460
```

Graphical representation

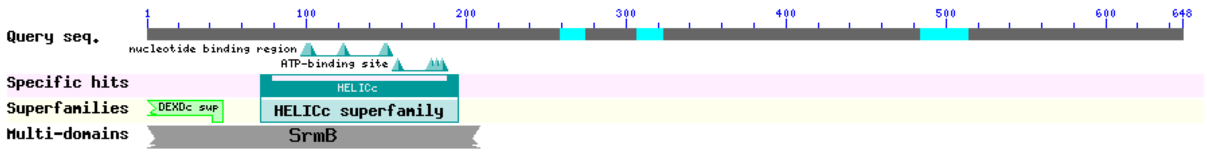

Armitage

>Cb.comp41200_c0_seq1 len=3735

cDNA

```
ATCTAACCTGTATGATTTTATAATAATTAATTTGTTAATGGTTTTGTTCGTAACATGTTAAGTTATGTGATGTCATTTTTTAGGCGTTCGTCAG
AGTCGGATTTGTCTTTAGAACAATGCCGCAAAATACTGGAAAATGAGACAACTGAAGATGCCGAATCCGGATGCCCCATCATTGCCGATGTTTC
GGGCAACCGTGAAGAATACGCGCTGTCCACAAAGATCGGCAAAATCACGGGCCAAAACGAAGACAAGTACATCATCGACGACCTGTATGAATTT
GATCCCAATGGTATTGGATACTCCATAGGGTCAGAAGTATCCTATCAAATGATAGTGGACAATGGAAAAATAGTTATTTATAATGTCAGTCTTA
ATTGCGACGACTGGAACCTGACCCACGCAAGGGAATCGACGTGGAGCACGCGTATAATAGTGTGTAAAGTCGACAAGCGGGAAAAGCGGATTCT
TTATTTATCACCCGGAGATGTCCAAATAGACCTAGACAAAGTTTCCATAGATTTTTTGCCGATGACGGGCGACTGGCTCGAGCTGGACGTTAAG
CACGAAGTGAACGAGTTCGCCATCGATTTGAGTGGAAAAATTTTGGATATTAACAGAATTTTGCCTGTGCGACCTCATATCGAAAAAGCCACAG
TAACTTCCTGGGATCCCGCGTCGGGCTCGGGCATTTTAAACAGACATATATTTTACAATCGGAATTCGTTATCGTGCGGTTACATGCCGGTGGT
CGGAGACAAAGTAGTGGCGGAAATAATCGAAAGCGCCCAACACGAGTGCGTATGGCGAGCTTTAAAATTGATACCGGAGTATGTGAGCAAACGT
TTCGATAAAATCGGCACGGACGCGTGGCAAATCGAAAAAAACCACCCGGATATCGAGATCGACGACATCAGTTTAAGTTTTTCGATGTTAAATG
AGCGTAAACGATTTAAGGTTAGGTTAATAAATAAAGCGGGTGAAAAAGTGACTTTGACTGGTGGTGAGTTTAGTAACGCGAACAGTCAGTGCAA
CATTATAAACGGATTTCCCCCAGACGTCGATATTTTACCGCGGGAATCGTTCGATCTAGAATGCGAGTGCACGGCGCGTAATATGGGCAAATCG
AATGAGTTTCTGTTAGTGTTTTTTAAAGGTTTCGACGTTAGCAAATGGATAGAAATAAATGTCGAACCCGAGAAAACGATAAATAACGGTTATT
ATCACAACAGACCTCAAAACTTTAGAAATAATTACGGTTCGAATAATCAGTTGATTCGAGGACAACGACGTGGCACCGTTAGATTTAATGCGGT
CCGAATACCGGATTACCCGGTGTCCAAAAAATTACTCGACCTGGTGGTTAGGTGTCGCGATTTAAACGACGCCGTCGGCCTTATCGAGGAATTG
AAAGCGACCAAACGGTCTCTATTCAGCGACCTAACCTCAATCAACTACGAGGACAAGTACCACAGCCTTCTGCATTTAGACGAGATCGAAAATT
TGATTTGCATACGAAACTACGACCAAGACTTGGCGTGTTTCATAAGAAACGGGGAGTTTTTGATGCTGGAAATTGAGAATCTGTCGGAAAAACG
GCCTTCCATAGTTTTGGGTGATAGAATCATAGCGAGCGATCCGTTAGGTCGGTCCAAAGAGGATTATGAGGGGAACGTTTTCAAGGTAGGAGCG
CAACACGTTTATTTGAAATTCTCGGGGCTGTTTCACGAGAACTACAATGGGGAAGATTATTCGGTGAGGGTCGTTCCCGGTCGAGCGGCTTACA
AAAGGCAACACCACGCCGTTTTTCTTGCAGCGAGAAATTTGGGTAAGGACTGGTTGTTTCCGTCGAAAATTGTCGAAAAAGACGCTCAAGTTCG
GTTCAAATACGACCGGTATTCGAGGGTAGTGCAATGCGTTAGAAGGTTGGGGCCCAAAGAGTTGTACGAAATAGTGGCCGAAGAAAACAGGTTA
AGAAAGTCGTTAGGAGATAAGGACGAGGACAATGGTGATAGCGATCTGCTGAAATTAGAATGGTTCAATCCCCACCTAAACTCCAAACAAAAAG
ACGCCGTCATTAATATCGTCAAAGGCGTGGCCAGGCCTCTGCCCTACGTAATATTCGGTCCCCCCGGTACCGGCAAAACCGTGACCGTAATAGA
ATCGATCCTCCAATTGGTACGGTTCGTTCCCGAGGCCAGACTGTTAGTCACGGCACCGTCCAACAGCGCCGCCGACCTGATAGCGCTGAGACTG
```

ATAGATTCGGGAATCCTTAAGCCTGGAAACTTGGTGCGACTCGTCTCAGTCAATTACGCCGTAGGCGACCACATACCGGCCAGACTCGTACCCT
ATTGCGCTACGGCGAGTTACGCCAAAGACGGCACCGCCGACGTCAATACCGTGTTGGAGAACGGCATGATGTGCGATTGCAGCAGGGCGGTGCT
CGGTAGACACAAGATCACAGTGTCTACGTGCTCGTCGGCCGGCTCGTTGTACTTGATGGGGTTCCCCCGGGGACACTTCACTCACATCGTCGTC
GACGAGGCGGGCCAAGCGGCCGAACCGGAAGTGATGATACCGCTGTCGTTCCTGGACAAGTGGACCGGGCAGGTGATTTTGGCAGGCGATCCGA
TGCAACTGGGACCGGTGGTGCTGTCAAAAATCGCCGAGGAATGCGGTCTCGGCGACTCCTTCCTGGAGAGATTGACAAACCGGTTTCCCTACGT
GAGGGACGCCGAAGGGTTTCCCCAATCCGGAGGTTACGATCCGAGGCTCGTTACCAAACTGCTCTACAATTACCGCTCGCTGGAGGCCATCTTG
GAGCTGTTTAGCTCGATTTTTTATCACGGAGAACTCATACCCACGATTTCCGAGAAACATAGCGACGAGGCTAAGCTCTTGGCGTCGTTGCGGG
AGATTCTTCCGGATCGGGACGACGGTACGGTACCGGCGATCGTTTTCCACGGCGTCGACGGCGAGAACTACCAAACGGCGGACTCGCCGTCGTG
GTACAACCCCCACGAAGTCGCCCAGGTCTTTTATTACGTCAACGAGCTGTATAGGTTAGGTTGCGGGCCCGCCCAACTGGGCGTCATCACGCCA
TACACCAAACAGGTGAAAGAGATCAAATCTGTACTGAAAGAGGCGGAGTTTGCCTTGCCCAAAGTGGGTACGGTGGAAGACTTTCAGGGGCAGG
AGTTCGACGTGGTTATACTGTCTACGGTCAGGTCGTCCAGGGATCACGTGCCGCGCGACTTGGAGCACAGCTTGGGGTTCGTTTCCAGTCCCCG
TCGTCTCAACGTCGCGATATCTAGGTCGAAGGCGTTGCTCGTTATCGTAGGCAATCCGAATTTGTTGTGCCACGACACGTACTGGCGGACCGTG
ATCGCTCATTGCCTGAAGCGGGGCGCTTATACCGGCTGCGATTTGAACGTGACGTGATACCGCGGCCGAAAAGTTAATATTCCGTGTTGTTTTT
TTTTTTATAATTTCCAGTTAAACGTGTCGAATACTGGCAAAGTTCGTTTTATCGCCGCGAGGAAACGACCATCGGGGCGACGTTGTCGGGTTAA
CTTTTTCCCCCGGCTTAGGGAAATTGCAAATTTGGCAACGTCGCGTCGCGGTACGTGCGTCACAGCGTAATATGATGACGTCGCTCGCTTGAGC
CGCCTCAAGCTATTTTCTTTTATAGCTGTTTTATTGCTGCCTCATTTATATACAGGGTGTCCCAATAGT

Protein RF 1: 55-> 3441(1128AA)

MLSYVMSFFRRSSESDLSLEQCRKILENETTEDAESGCPIIADVSGNREEYALSTKIGKITGQNEDKYIIDDLYEFDPNGIGYSIGSEVSYQMI
VDNGKIVIYNVSLNCDDWNLTHARESTWSTRIIVCKVDKREKRILYLSPGDVQIDLDKVSIDFLPMTGDWLELDVKHEVNEFAIDLSGKILDIN
RILPVRPHIEKATVTSWDPASGSGILNRHIFYNRNSLSCGYMPVVGDKVVAEIIESAQHECVWRALKLIPEYVSKRFDKIGTDAWQIEKNHPDI
EIDDISLSFSMLNERKRFKVRLINKAGEKVTLTGGEFSNANSQCNIINGFPPDVDILPRESFDLECECTARNMGKSNEFLLVFFKGFDVSKWIE
INVEPEKTINNGYYHNRPQNFRNNYGSNNQLIRGQRRGTVRFNAVRIPDYPVSKKLLDLVVRCRDLNDAVGLIEELKATKRSLFSDLTSINYED
KYHSLLHLDEIENLICIRNYDQDLACFIRNGEFLMLEIENLSEKRPSIVLGDRIIASDPLGRSKEDYEGNVFKVGAQHVYLKFSGLFHENYNGE
DYSVRVVPGRAAYKRQHHAVFLAARNLGKDWLFPSKIVEKDAQVRFKYDRYSRVVQCVRRLGPKELYEIVAEENRLRKSLGDKDEDNGDSDLLK
LEWFNPHLNSKQKDAVINIVKGVARPLPYVIFGPPGTGKTVTVIESILQLVRFVPEARLLVTAPSNSAADLIALRLIDSGILKPGNLVRLVSVN
YAVGDHIPARLVPYCATASYAKDGTADVNTVLENGMMCDCSRAVLGRHKITVSTCSSAGSLYLMGFPRGHFTHIVVDEAGQAAEPEVMIPLSFL
DKWTGQVILAGDPMQLGPVVLSKIAEECGLGDSFLERLTNRFPYVRDAEGFPQSGGYDPRLVTKLLYNYRSLEAILELFSSIFYHGELIPTISE
KHSDEAKLLASLREILPDRDDGTVPAIVFHGVDGENYQTADSPSWYNPHEVAQVFYYVNELYRLGCGPAQLGVITPYTKQVKEIKSVLKEAEFA
LPKVGTVEDFQGQEFDVVILSTVRSSRDHVPRDLEHSLGFVSSPRRLNVAISRSKALLVIVGNPNLLCHDTYWRTVIAHCLKRGAYTGCDLNVT

Comparison with *Tribolium* (AA)

NOT

Graphical representation

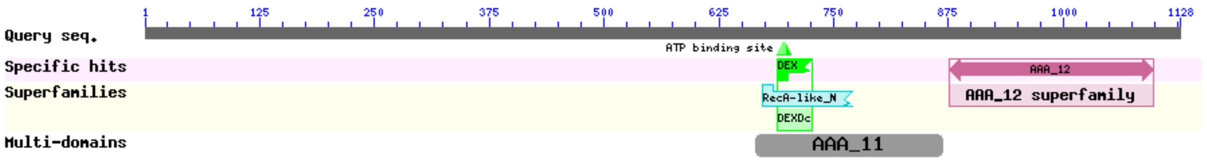

GLD-1 homolog

>Cb.comp41351_c0_seq6 len=3023

cDNA

CGGTCTTGGCGCGCGCGCACGCGCGCTCCGGCCTCCCGGATCGCCGCACTTCATTCCTCTCGCGTTGCTCGGCCGGTCAGTCGCGTACACGAGC
GTAAAGAGTCGAGTTGTGTGCGATCGCGGCGCGGCGTTGATGTTTATATGAAATTTTTGAGGTTTTGATTTTGAAGCGACTTGTGGGCCCGAAA
ACGAAAATAATGTGCGACCAAAATGCGGCCGCCGCCGCTGCTTCGACGCAGAGCATAGCGGACTACCTCGCGCAATTACTGAAAGACAGGAAAC
AACTGGCCGCGTTTCCAAACGTGTTTATTCACGTAGAGAGGCTTCTGGACGAAGAGATAGCGAAAGTGAGGGCTAGTCTGTTTCAAATAAACGG
AGTCAAGAAAGAGCCTCTGATACTGCCAGAACCAGACGGTCCGGTTACTACTCTAACCGAGAAAGTGTACGTCCCGGTAAAGGAGCACCCAGAC
TTCAACTTCGTGGGAAGGATCCTGGGCCCCAGGGGCATGACGGCCAAGCAATTAGAGCAAGAAACGGGCTGTAAAATCATGGTACGCGGGAAGG
GCTCGATGCGGGACAAAAAAAAGGAGGATCAAAATAGAGGGAAACCGAACTGGGAGCACTTGTCCGACGAGTTGCACGTCTTGTTAACCGTCGA
GGACACCGAGAACCGGGCCCAAATCAAACTCCAAAGGGCTGTGGACGAAGTCAGAAAACTACTCGTACCTCAAGCTGAGGGAGAAGACGAACTG
AAGAAAAGACAGTTGATGGAGTTGGCCATCATTAACGGAACGTATAGGGATTCTAGCGCAAAGGCTGCTTCTGCTTCAGACCTTTGCGCCCTTG
CAGCGTGCGACGAGGAGTGGAGGCGCGTCGCCGCCGCCGCAGTAGACGCCCAGCGTCTGCTGTCGCCGGGCATTCCCGGCCTCGCCACGCCCCT
AAGAGGTCCGGCCACCCCTTTAGGCGCGCCCCTTATACTCTCGCCGCGGATGTCGGTACCCACTACGGCGGCCTCAATATTGAACGGGTCTGCG
CCCCCGGGGTCTCTGTTGTCGCCGGGGGACCCGCACGGCCTCCTCTACGCCCCCTACGCGGACTACACGAATTACGCCGCGCTAGCGGCCACGC
CCCTTCTGACGGAGTACGCGACGGCAGACCATTCGGGTGCGTCGGCGGTTGCCAAGCAACGCAGGCATCTGGGGCAGATAAGAGAGCACCCCTA

TCAGAGGGCGGGCGCGCTCTCTTAACCGTTTTTTTTCTAAAGACAAACTGTCACACGAAAGCTTGCGGAGTGCTAACCAAATCGTCACACATCC
ATGCCAACTCCTAAGTACCAACTAACGAAACAATTCGGGAGGCGGCGCCCCTCCACAATCCACCCCTCGCCCGCCGGCTATAGAAAAGAATGGG
TAGCGGTACGTCTAACAAATTACATCACTAACATTTTTTTTTTTATTTTGGAACATTAATTCTACTGTTTATTTTATGTTTTTTTTTTTAATTTTT
GACAAACGGCCCTTCGTCCGAACGGTACGCCAATTTTTGTACCTTGAAGTATGGCGCGGGTCGTTTGTCGTAATGGCTGGTTTACACATTGCCG
TACATTTGACTAACATCGTGAGCGACGTAACCGTACCACTAGTAGTGCATGTCTCTTTTTCATGCCGTATTTTTATAATTATTACTATAATCGT
AACACTAAATTTAGACTAGAACGCAATTTGGACGACGCAATGTGTGAACGGGGCATCGGCACATGTATATCGAAGTAGGCTCGCGCGTTGTGCA
CTAAACCGAACTTCTTTTTAAAATATATTCGTGACGTCATGGTACATCTGTCAAATATTAATAGGCGTTCTTCGATTACCCGTAAATTTTCAAA
ACTTGTGCCTTGCCGTTTAGTGCACAACCGCTTGTAACTCCACACAAAGTTAGTATAGACTTATATAATGTTACTAATATCTTTTATACTTGTA
TGATGTCTGTCTTCAGTGAAGTACAAAAACTTTTAGTGCCAATTTTTCAAAAAGTTATATAAGCGGAATATTCCGGAAAAATTTATCGATTCAG
GGGTCAGATTCTCGAATATTCTCTGTGTTAAGTACTTACGGCTATGAATTTTTCCGGAAAAGCCTGTAGAGTAGAGGCAATATATAGAGGAGCA
ATATTTTGTAGTGTTTACGAATAACGAAAAGGTAAGTAAGACTTAAGTTAAGGTCGCTGACGACGTCGACGAAAACAAAGCTATGAATCTAATT
ATCTAAACTTGAATGCAATATAAATTTGATATTTTCGACATTTCGCAAATCTAGCGTTGAGTAGCAATAAAAAAATCTCCGTCCTGAAGTGAAA
AATAGTATATCAAAAAATACTCAGAACAATGAGAAGGTAATCAGGTGCAATACCGGTGGCTCAACATTTGATTTTTTTTTAACTACGTGGAGCC
AAACGTGCAATCTCAAGAGTTTTTTTTTATTATAGAAAAAATGATCTATATTTGTTTTAGAAGCTGGGACGGGAAACTTTATATAAACGTAATA
TTATTTATTATATAAATTTTCCCGATGAGTGTTATAGGTATATTCAATGTTGTACTTAGATATGATTATGAAAAAAAGCATATATTGTGTATCG
ATGTTATTATCCATATCTAATTAAGAATATAATTCGTGTTTGTTTGATCATTTTGACTCGAAACAAGGCATTAAGGGAGGGAAGCGTTAAAATT
ATCTGGCTATGATGGCGCTGTGCCTTTTATCAAATTACGTGCGCATCAAAACCAACAAGTTCCGATAATTTTGACGTCGACTCTCCAGTATATT
ATTTATGTTGAGTTCAGTGTAGTTATTATTTTTAAAGTGTCATATAAAATTAGGATTGCAGATAATTTTGATGTCTTTATTAAGAGAGGACGCA
TAGGGACAAAGGTTACCTGTGGTAGGTATGTCTGTATGCTAGAGTTGTCCCGGACATTGTGCACAAATCGGGAGGAAATTTAATTAAATTATTT
AACGTTAAAAAAAAA

Protein RF 3: 198->1247 (349AA)

MCDQNAAAAAAASTQSIADYLAQLLKDRKQLAAFPNVFIHVERLLDEEIAKVRASLFQINGVKKEPLILPEPDGPVTTLTEKVYVPVKEHPDFNF
VGRILGPRGMTAKQLEQETGCKIMVRGKGSMRDKKKEDQNRGKPNWEHLSDELHVLLTVEDTENRAQIKLQRAVDEVRKLLVPQAEGEDELKKR
QLMELAIINGTYRDSSAKAASASDLCALAACDEEWRRVAAAAVDAQRLLSPGIPGLATPLRGPATPLGAPLILSPRMSVPTTAASILNGSAPPG
SLLSPGDPHGLLYAPYADYTNYAALAATPLLTEYATADHSGASAVAKQRRHLGQIREHPYQRAGALS

Comparison with *Tribolium* Held out WIngs(340AA)

```
Query  1    MCDQNAAAAAAASTQSIADYLAQLLKDRKQLAAFPNVFIHVERLLDEEIAKVRASLFQING  60
            MCD        ASTQSIADYLAQLLKDRKQLAAFPNVFIHVERLLDEEIAKVRASLFQING
Sbjct  1    MCD----TTNASTQSIADYLAQLLKDRKQLAAFPNVFIHVERLLDEEIAKVRASLFQING  56

Query  61   VKKEPLILPEPDGPVTTLTEKVYVPVKEHPDFNFVGRILGPRGMTAKQLEQETGCKIMVR  120
            VKKEPL+LPE DGPVTTLTEKVYVPVKEHPDFNFVGRILGPRGMTAKQLEQETGCKIMVR
Sbjct  57   VKKEPLVLPEADGPVTTLTEKVYVPVKEHPDFNFVGRILGPRGMTAKQLEQETGCKIMVR  116

Query  121  GKGSMRDKKKEDQNRGKPNWEHLSDELHVLLTVEDTENRAQIKLQRAVDEVRKLLVPQAE  180
            GKGSMRDKKKEDQNRGKPNWEHLSD+LHVLLTVEDTENRAQIKLQRAV+EV+KLLVPQA+
Sbjct  117  GKGSMRDKKKEDQNRGKPNWEHLSDDLHVLLTVEDTENRAQIKLQRAVEEVKKLLVPQAD  176

Query  181  GEDELKKRQLMELAIINGTYRDSSAKAASASDLCALAACDEEWRRVAAAAVDAQRLLSPG  240
            GEDELKKRQLMELAIINGTYRDSS+KA SA+       ACDEEWRRVAAAA + QRLLSP
Sbjct  177  GEDELKKRQLMELAIINGTYRDSSSKAVSAT------ACDEEWRRVAAAAAETQRLLSPA  230

Query  241  IPGLATPLRGPATPLGAPLILSPRMSVPTTAASILNGSAPPGSLLSPGDPHGLLYAPYAD  300
            IPGLATPLR PATPLGAPLILSPRMSVPTTAASILNGSAPPGSLLSPGDPHGL+Y PYAD
Sbjct  231  IPGLATPLRTPATPLGAPLILSPRMSVPTTAASILNGSAPPGSLLSPGDPHGLIYTPYAD  290

Query  301  YTNYAALAATPLLTEYATADHSGASAV-AKQRRHLGQIREHPYQRAGALS  349
            YTNYAALAA+PLLTEYATADHSGA+AV AKQRRHLGQIREHPYQRAGALS
Sbjct  291  YTNYAALAASPLLTEYATADHSGAAAVAAKQRRHLGQIREHPYQRAGALS  340
```

Graphical representation

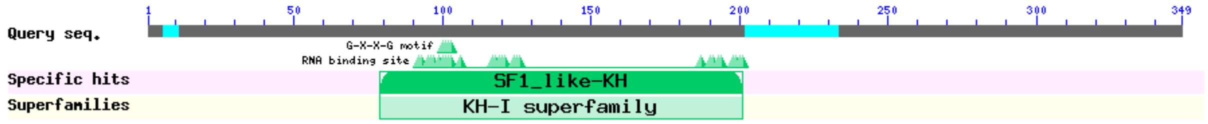

ACO-1 homolog

>Cb.comp24263_c0_seq1 len=3684

cDNA

```
GTGACACTGACAGTTGAAAGTACGTGTATTTTTTTCGATCTGTGATTGTCTATTCTTTGGACGCAAACAAACAATTAGTAATTTTTATAAGAAC
GTGTACAAAACTGGTTTCAACTGATAACATAATCATAATTCGTCGCGGCCACAACCGCATGTAATTTTTGTTATCTCCCAAAATCCGAAAATAC
TTTACGAAACTACGTGACTTAACACTTTATAGAAATGGCAGAAAATAATCCTTACAATAAGTATTTAAAAACTCTAACTGTAGGAAGCAAAGAA
TACGTTTACTATGATTTATCATCATTAGGGGAACAATACAATCGTTTACCATATTCTATAAGAATACTGCTAGAATCTGTAGTGAGAAATTGTG
ACAATTTTTCTGTAAAAGAACAGGATGTACAGAATGTCCTTAATTGGGAGTCAAATCAGGATAGTCAAGATGGTGTTGAAATTGCATTTAAACC
AGCCAGAGTTATTTTGCAGGACTTTACTGGAGTACCTGCTGTGGTTGACTTTGCAGCTATGAGGGATGCTGTGAGAGATTTAGGTGGAAATCCA
GAAAAAATTAATCCTATTTGTCCAGCAGATCTTGTTATTGACCATTCTGTTCAAGTGGATTTTGTGAGATCATCTGATGCCTTACAGAAAAATC
AAGATCTTGAATTTGAAAGAAACTTTGAAAGATTTATGTTTTTGAAGTGGGGTGCCAAAGCTTTTAATAACATGTTGATTGTACCACCAGGAAG
TGGTATTGTCCATCAAGTTAACTTGGAATATTTAGCAAGAGTAGTATTCACTGGAACTAAAAAACCTGTTTTGTACCCTGACACAGTAGTTGGT
ACTGACTCCCACACTACAATGATTAACGGTCTAGGTGTTTTGGGATGGGGAGTGGGGGGAATTGAAGCTGAAGCTGTTATGCTGGGTCAAGCAA
TTACAATGTTGCTACCTCAAGTTGTTGGATACAAACTCTATGGCACTTTAGGGCAGTACGTTACATCAACTGATTTAGTATTAACTATCACTAA
GCATTTGAGGCAAATTGGAGTTGTTGGAAAATTTGTTGAATTCTATGGACCTGGAGTGTCGGCATTATCCATTGCTGATCGTGCAACAATCTCC
AATATGTGTCCGGAATATGGAGCAACAGTTGGATTCTTCCCTGCTGATGAAACTTCTTTATCCTATTTGAGACAAACAAATCGGTCAGAAGAAC
AAGTAAAATTGATTGAAGGCTATTTGTTGGCGACAAAGCAAATGAGAAACTACTCATCGGAAGAGAACCCCATTTTTAGTCAGACTTTTGGCCT
CGATCTATCTACCGTTGTGTCATCTGTTAGTGGACCTAAAAGACCCAATGATAGAGTTTCTGTGTCCGACATGAAAAACGATTTCATTAGCAGT
TTGACCAACAAAATTGGATTTAAAGGATTTGGCTTGACCAAAGAAAAAGTTGCCACTAAGGCTAAGTTTATGTTTGATGGTAAATCTTATACTA
TAGGACATGGTAGTGTTATTATTGCAGCTATAACATCATGTACCAATACAAGCAATCCCAGTGTTATGCTTGGAGCCGGTTTGCTAGCAAAAAA
AGCTGTTGATGCTGGTTTATCAGTGGAACCATATATTAAAACCAGTCTGTCACCTGGTTCAGGTGTGGTCACTTATTACCTTCGCGAATCTGGA
GTTATACCTGCCTTGGAACAACTCGGATTTGACGTGGTTGGATATGGGTGTATGACATGCATTGGAAATTCTGGAGGAATTGATGAAAACATTG
CCAATGCAATTGAACAAAATGATTTGGTGTGTTGTGGAGTACTCTCTGGAAACAGAAACTTTGAAGGCAGAGTCCATCCTAACACCAGAGCCAA
TTATTTGGCTAGTCCGCTTCTGGTTATAGCATATGCTATTGCTGGCAGAGTTGATATTGACTTTGAGACTGAACCTTTGGGTCCAAGGGCTGAT
GGAACACAAATCTTTTTGCGGGATATTTGGCCTACAAGACGGGAAATTCAAAGTGTTGAACAGCAACATGTCATTCCTGCAATGTTTAAAGAGG
TGTATTCCAAAATCGAAAATGGATCAAGTCAGTGGCAAAAATTGAAGGCCCCAGAAGGTAAACTGTATCCCTGGTCCAATGAATCTACCTATAT
AAAAAAACCTCCCTTTTTTGATGGAATGACTAGGGAACTGCCGACACCAAAGCCAATTCAAGGTGCAAGAGTGCTGATTTACTTAGGAGATTCA
GTAACTACTGATCATATTAGTCCTGCTGGATCTATAGGCAGAAGTAGCCCTGCGGCTAGGTATCTGGCTGCAAAAGGTTTGACTCCAAGAGAGT
TCAACTCATATGGATCTAGAAGAGGTAACGATGCTATTATGGCCAGGGGTACTTTCGCGAATATCCGTTTGGTGAACAAGTTTATGAGCAAATC
TGGACCCACAACATTGTATTTACCAAACAATGAAGAGATGGATATTTTTGATTGTGCACAAAGATATGCCAGCAACAATATTCCTCTAATCATC
ATTGCGGGTAAAGATTATGGAACAGGATCGAGCAGAGACTGGGCTGCTAAAGGTCCCTTTTTGCTAGGCGTTCGAGCTGTTATAGCCGAATCTT
TTGAAAGGATCCATCGTTCTAATCTGGTCGGCATGGGATTAATTCCTCTGCAGTTTCTTCCGGGTGAAAACGCTGAAACTCTGGCTCTGACAGG
CAAAGAGGTTTACAATATTCAGCTACCCGAAAACTTGAAGCCCCTTGAACACATCACAGTCTCGACAGAAACCGGAAAGAAATTTAAAGTTTTG
CTTCGATTTGATACAGAGGTTGATTTATTATTTTACAAGCACGGAGGTATTCTTAATTACATGGTTAGAAAAATGATAAGTTAAAACAAAATTT
GTGTATATGGGGAAACACGCGGTTGGGTTTATTTTTTGTGAGTTGTTTGCTCTGTTTTTAAAAGTATAACGAACTTAAAATACTTTTATAACTT
TATATTTTTTCATTGCACCTGAAAATCTGCTTGATGGTTTTGCTTGCACAACATATGCTATACTATATATATCACAGAAATAACAAATTCTTTT
GAAATTGAAGGTTGGAAATGTCTCTGAGTAAAAGTATGGTTGCTAAATCCCTATCAGCCTGGGATAACCAATATCAGAAACACATTGAAAAAAT
GCCAATTTTAACATTAATTTTCGATCAGAGGTTATTTTGTTAATAAATTTTGCTTTAATTTTTTATCTTGGTTTGGTTTGAATACTTTCCAAGA
AATAAAAGGAGAATGGTTTGTTTTTATTGAACCTTAATGCTCCCTTTCTCTGTATTATAGGCCAACCTTTCGAAACATTTCATTTTTCATATAT
TATTTCCATTTTATAAAATTGGGATGTAATTTAATATACCTTGCAGACTTATCAGGCTATTATACCCAAGTTGTAATTGACATATTTCATATGA
AATGTTACTTGGAATTTAAAAATTTTAGCAGATGATTTCTAAAGTATCACTCAACCTATTCAATATTGTATCCACAACTTTAATATTTTTATGG
TGGTTTGAGGGGGAAGGATTTTTTACTTATTAGAAGCTAAGGTAACAAATGTTTTTAAGGCTTATTATATTTTATGAATTGTTAGCAATAAATC
TGCAATATTAAAAAAAAA
```

Protein RF 1: 223-> 2904(893AA)

```
MAENNPYNKYLKTLTVGSKEYVYYDLSSLGEQYNRLPYSIRILLESVVRNCDNFSVKEQDVQNVLNWESNQDSQDGVEIAFKPARVILQDFTGV
PAVVDFAAMRDAVRDLGGNPEKINPICPADLVIDHSVQVDFVRSSDALQKNQDLEFERNFERFMFLKWGAKAFNNMLIVPPGSGIVHQVNLEYL
ARVVFTGTKKPVLYPDTVVGTDSHTTMINGLGVLGWGVGGIEAEAVMLGQAITMLLPQVVGYKLYGTLGQYVTSTDLVLTITKHLRQIGVVGKF
VEFYGPGVSALSIADRATISNMCPEYGATVGFFPADETSLSYLRQTNRSEEQVKLIEGYLLATKQMRNYSSEENPIFSQTFGLDLSTVVSSVSG
PKRPNDRVSVSDMKNDFISSLTNKIGFKGFGLTKEKVATKAKFMFDGKSYTIGHGSVIIAAITSCTNTSNPSVMLGAGLLAKKAVDAGLSVEPY
IKTSLSPGSGVVTYYLRESGVIPALEQLGFDVVGYGCMTCIGNSGGIDENIANAIEQNDLVCCGVLSGNRNFEGRVHPNTRANYLASPLLVIAY
AIAGRVDIDFETEPLGPRADGTQIFLRDIWPTRREIQSVEQQHVIPAMFKEVYSKIENGSSQWQKLKAPEGKLYPWSNESTYIKKPPFFDGMTR
ELPTPKPIQGARVLIYLGDSVTTDHISPAGSIGRSSPAARYLAAKGLTPREFNSYGSRRGNDAIMARGTFANIRLVNKFMSKSGPTTLYLPNNE
EMDIFDCAQRYASNNIPLIIIAGKDYGTGSSRDWAAKGPFLLGVRAVIAESFERIHRSNLVGMGLIPLQFLPGENAETLALTGKEVYNIQLPEN
LKPLEHITVSTETGKKFKVLLRFDTEVDLLFYKHGGILNYMVRKMIS
```

Comparison with *Tribolium* PREDICTED: cytoplasmic aconitate hydratase-like (893AA)

```
Query  5    NPYNKYLKTLTVGSKEYVYYDLSSLGEQYNRLPYSIRILLESVVRNCDNFSVKEQDVQNV  64
            NP++KYLKTLTV SKEY YYDLS+LG QY+RLPYSIR+LLES VRNCDNF VKE DVQN+
Sbjct  4    NPFDKYLKTLTVESKEYKYYDLSALGAQYDRLPYSIRVLLESAVRNCDNFQVKENDVQNI  63

Query  65   LNWESNQDSQDGVEIAFKPARVILQDFTGVPAVVDFAAMRDAVRDLGGNPEKINPICPAD  124
            LNWE NQ  + G+EI FKPARVILQDFTGVPAVVDFAAMRDAV+ LGGNPEKINP CPAD
```

```
Sbjct  64   LNWEQNQSVEGGIEIPFKPARVILQDFTGVPAVVDFAAMRDAVKGLGGNPEKINPSCPAD  123

Query  125  LVIDHSVQVDFVRSSDALQKNQDLEFERNFERFMFLKWGAKAFNNMLIVPPGSGIVHQVN  184
            LVIDHSVQVDF RS  AL+KN+DLEFERN ERF FLKWGAKAFNNMLIVPPGSGIVHQVN
Sbjct  124  LVIDHSVQVDFARSPSALKKNEDLEFERNQERFTFLKWGAKAFNNMLIVPPGSGIVHQVN  183

Query  185  LEYLARVVFTGTKKPVLYPDTVVGTDSHTTMINGLGVLGWGVGGIEAEAVMLGQAITMLL  244
            LEYLARVVFTG  KP+LYPDTVVGTDSHTTMINGLGVLGWGVGGIEAEAVMLGQ+I+MLL
Sbjct  184  LEYLARVVFTGKDKPILYPDTVVGTDSHTTMINGLGVLGWGVGGIEAEAVMLGQSISMLL  243

Query  245  PQVVGYKLYGTLGQYVTSTDLVLTITKHLRQIGVVGKFVEFYGPGVSALSIADRATISNM  304
            P+VVGY+L+GTLGQYVTSTDLVLTITK+LRQ+GVVGKFVEFYGPGV+ALSIADRATI+NM
Sbjct  244  PKVVGYRLHGTLGQYVTSTDLVLTITKNLRQLGVVGKFVEFYGPGVAALSIADRATIANM  303

Query  305  CPEYGATVGFFPADETSLSYLRQTNRSEEQVKLIEGYLLATKQMRNYSSEEN-PIFSQTF  363
            CPEYGATVG+FP DE SL+YLRQT+R +EQ+KLIE YL ATKQ+RNY++E N PIFSQ+
Sbjct  304  CPEYGATVGYFPVDEHSLTYLRQTSRPDEQIKLIEAYLKATKQLRNYANEMNEPIFSQSV  363

Query  364  GLDLSTVVSSVSGPKRPNDRVSVSDMKNDFISSLTNKIGFKGFGLTKEKVATKAKFMFDG  423
             LDLSTVVSSVSGPKRPNDRVSVSDMKNDF   L+NKIGFKGFG+ + K+ T+AKFM++G
Sbjct  364  SLDLSTVVSSVSGPKRPNDRVSVSDMKNDFRLCLSNKIGFKGFGIPEAKLNTEAKFMYNG  423

Query  424  KSYTIGHGSVIIAAITSCTNTSNPSVMLGAGLLAKKAVDAGLSVEPYIKTSLSPGSGVVT  483
              YTI HGSVIIAAITSCTNTSNPSVMLGAGLLAK AV AGL+V PYIKTSLSPGSGVVT
Sbjct  424  SQYTIRHGSVIIAAITSCTNTSNPSVMLGAGLLAKNAVAAGLTVAPYIKTSLSPGSGVVT  483

Query  484  YYLRESGVIPALEQLGFDVVGYGCMTCIGNSGGIDENIANAIEQNDLVCCGVLSGNRNFE  543
            YYL+ES VI AL QLGFD+VGYGCMTCIGNSGGIDENI NAIEQNDLVCCGVLSGNRNFE
Sbjct  484  YYLQESKVIDALTQLGFDIVGYGCMTCIGNSGGIDENIVNAIEQNDLVCCGVLSGNRNFE  543

Query  544  GRVHPNTRANYLASPLLVIAYAIAGRVDIDFETEPLGPRADGTQIFLRDIWPTRREIQSV  603
            GR+HPNTRANYLASPLLVIAYAIAG VDIDFE EPLG R DG+ +FLR+IWPTR+EI +V
Sbjct  544  GRIHPNTRANYLASPLLVIAYAIAGTVDIDFEKEPLGKRPDGSPVFLREIWPTRKEIHAV  603

Query  604  EQQHVIPAMFKEVYSKIENGSSQWQKLKAPEGKLYPWSNESTYIKKPPFFDGMTRELPTP  663
            EQQ+VIPAMF++VYS+I+ GSS WQ L AP G LYPWS+ STYIKKPPFFDGMT++LP
Sbjct  604  EQQYVIPAMFQQVYSRIQLGSSSWQSLNAPSGILYPWSDSSTYIKKPPFFDGMTKQLPPM  663

Query  664  KPIQGARVLIYLGDSVTTDHISPAGSIGRSSPAARYLAAKGLTPREFNSYGSRRGNDAIM  723
            +PI GARVL+YLGDSVTTDHISPAGSIGR+SPAARYLA  GLTPREFNSYGSRRGNDAIM
Sbjct  664  QPISGARVLLYLGDSVTTDHISPAGSIGRNSPAARYLAQNGLTPREFNSYGSRRGNDAIM  723

Query  724  ARGTFANIRLVNKFMSKSGPTTLYLPNNEEMDIFDCAQRYASNNIPLIIIAGKDYGTGSS  783
            ARGTFANIRLVNKFMS +GP T+YLP NEEMD+FDCA+RY S    PLII+AGKDYG+GSS
Sbjct  724  ARGTFANIRLVNKFMSNAGPKTVYLPTNEEMDVFDCAERYKSAKTPLIILAGKDYGSGSS  783

Query  784  RDWAAKGPFLLGVRAVIAESFERIHRSNLVGMGLIPLQFLPGENAETLALTGKEVYNIQL  843
            RDWAAKGP+LLGVRAVIAESFERIHRSNLVGMG+IPLQFLP E AETL LTGKE+YNI++
Sbjct  784  RDWAAKGPYLLGVRAVIAESFERIHRSNLVGMGIIPLQFLPNETAETLGLTGKEIYNIEI  843

Query  844  PENLKPLEHITVSTETGKKFKVLLRFDTEVDLLFYKHGGILNYMVRKMIS  893
            P +LKP ++I +ST+T K F V+LRFDTEVDLLFYKHGGILNYM+RK+++
Sbjct  844  PADLKPGQNIKISTDTSKTFNVVLRFDTEVDLLFYKHGGILNYMIRKIVA  893
```

Graphical representation

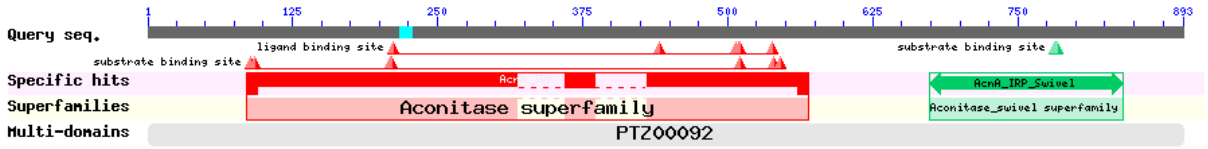

Similar to pre-mRNA splicing factor ATP-dependent RNA helicase PRP16 (Tribolium), mut6 homolog (Chlamydomonas)

>Cb.comp43081_c0_seq5 len=5418

cDNA

```
GGTGAGCTTTTTTCTTTTAATAGAATGACGTTTCAATATAAAGTTGAGAGAGAAAAATGAAATTCGAGGTGTAAAGTCGATCAAATTTTTGGAG
GAATCTTTTTTTTCCGTTTTGTTTATTGCAGGCCGGTCTACAAGAGGAAATATTTTAACGTCCCCCGCCGGATCCCAAGGTGTGCCACGAATCG
CTGATGGATCAGATCAAGCGCGGCGCCACCTTGAAACGGGCCAAAATCGTCAACGACCGCTCCGCCCCAAAGATTTACTAAACCCGATTCTAAT
TTAGGTCAGCAAGTTGACGTAAACGTTGAACCGGCAGTCGACGGACGAAACTATAAATGAAAATGTAACACGTAGACAGTTTTCATCAGTAGTT
CGCACTGGCTAAACATAACCTCAAACCGACGAAATAAATTGCCACTATCATCGAGTAGGAAATACATGTGGCAGTCTTATTATAATTTTGAAAA
TAATTCAAATTTCCGTGACATAGCCATTTCTAGGTTTTCCGATTTCAGAAAATGCGGCCTTTTAGTTTTTTTTACTGCTGTTGGCGACAAGTGG
AGGGTAATTCGTCCAACCGTTTTAGGTTGTGTTGGCTTGTCTTCTTCCTTCCTTAACTGGCCGATGTTATGTTAAGGTTATGAACCGCGAGAGA
GAGAGAAAAAGAGAGATTAAGCTTATTTAATTGTTCGACATGCGTGTTTGAGGCTAGAATAAGTTCCGTTTTCGTAGTGATCGAAGCCGAAGGA
ACGTCCACCGTCCGCGCGTACGACTGACTTATTTTTGTATTTTTCATAAAAATTCGAATGGAATATTGCAAGTATTACTCGTGGGACTGATGTT
AAAAGTAGCATTTAAAGTACACGTATCCAATATTATAATTATTAATTAGCCTAAAAGTAACGTAAGATGTTGTTTTTAATTTTCTTTTTAACGG
TCACTATTCTGAGGTGGCGTACAAAGAGCTGCAGTTTCCACGAACGGTTCTTATGTTTAAACCACAGCCCGTGTCGCCCACGAATCGGACGTCC
GCTCCCTTTTTTTTTTTTAAATAAGTTTTCGGGATCACTAATCGATTAGTTAGTTTTGTAAAATGTACAAAGGATTGCGTTCGTTGTTTAATAT
GATGTACATAATCATCAAGAAGAAATATGTCTAATATATTTACGTAGAGCCGCTAAGTAAAGAGGAGTGTTTTATTTTTTTTTCTGTTTCGCGT
TAATTCGTTTATGTTTAATTTTGTTCAGCTCGGTGTTAACGACAACAATAATAAAACAACATAATATTAATTGTTTAACTGTTGGATGGATCAT
TTCTATTTCTTAAAAAAAAATTGCATGCCCATAATATTTAAATGAAATAAACAAAATAAATGGTTTCTGTGAGCGCAAGTCTAGTATCATTCCA
ACTTTCCTTACGCGTTCGACGCATAACAATAAGGACAATTTTAAAACTCTCAGAATATATTTAGAAAAAGAAGGAAAGGTGACTGAAAGACTAC
AAGCCGAACCGCGCGGGAGTCCTCCTCGGCGTCGTCCCCGCGGTGACGATCTCCTGCCCTTTGTGCATTGCCGCCTCCTTCTTCTCCGCCGCCT
CCTTCCTGGCCCTCATCTCTTCCTGGGCCACCTGCATCTGGTTTTCCATTTCCAGCAAATGTTCGGCCGCCTGCTTCTTCTTCGCCCGGCCTGA
CTTGCCCGTCTCCTTGAGGGAGAAGAACATGGGTCCCAGCTCGGCGAGCCAGTGGCCGTCGACCGATGTTACGCACTGCATGTACTCCCTCGCA
GTCATAACCAGTTCGTGGTACACTACGTAGTCGGGGGTGTTTCCGAGACCGAAAAGGGCAGAGGTGGGGTGCAGATAGCACGGCATGCCCGTAC
GGCAATTGACGTATTCCCCGATTCCTTTTAACCGGGCCGCTTGGTGGAAGTAGGCGGAACAGATGCACTTCCTGACTACGTCCCAGTCCGTGCC
GCACGACTTGACCTCAAACTTTTGCTGCACGAGGATATCCTTAAGCTGCTGCCTAACCTCGCGCACTTTCCGCATCGCTTTCACGTGGATAAAA
TGCTCGTTGCACCAATGCGACGAGTAATTGTTTTGCCGCCACTGGTTGTAGACGTTGAGGAAAGTGAGGTGGTCGCTCTCGGGCACTTGAAACT
TTTCCCTCACTCCGTCCGACTCTTCTTCGCGACCCTTCGGTCTGTAGAATATCGACGGCACCGACAGCATCGAAACTATAATCAGGATTTCGGC
GGTGCATTCCATTTGGGTCGATACGATCAGCATCTGGCACTGGGGCGGATCGAGCGGGAACTCGGCCATCTGACGGCCCAGCTTTGTCAACACG
CCTGTATGGTCGAGTGCGCCCAAGATCCACAGCTGGTACAATGAATTCAAGATATTGTCCTGTGGTGGAGGGTCCATAAAATGGAACTGGAGCA
GGTCCTGGACCCCGAGCGACTTGAGGAGCAGCACCGTGTTTGCCAGATTGGTGCGTTGGATCTCCGGTACGGTCGTGACTAGAAGCTCGTCTTT
GTATTGACGCTCGGTGTAGAGGCGGAACGCTTGGCCTGGTCCGGTACGCCCCGCCCTACCGGACCTCTGGTTGGAGTTTGCCTGACTTATGGGG
TAGATCTGCAAAGCGTCCATGCCTATCCTCGGATTGTAAACCTTCAGCTTACAGTATCCCGAATCGATGACGAATATGATACCGTCGACGGTGA
GCGACGTTTCGGCTATGTTGGTGGCGACCACACATTTCCTGATACCCTCCGGTGACCGCTGGAAGATTTTCGCCTGCAGGTCGGACGGCAGCTG
CGAGTAAATCGGCAAGATCGACAGCTCGGGGGCGTTATCGATCTCCGCCAACCGTTCGGCGAGGACCTCGCACGTTACTTCGATGTCCTCCTGG
CCAGGCATAAAGATCAAAATGTCGCCAGAGGGAGGCTGCAGGTGTATCTGGAGGGCCTGCTTAACGGCCGCGTCGACGTAATCCTCAACGGCGT
TCTTGCTAAATAGCACCTCGACCGGGAAAGTGCGACCCGGTATCGTAAACGTAGGCACGTTCCCGAAAAACATGGAGAACTTGCTCGAGTCCAT
AGTCGCTGACGTCACGATCAGTTTGAGGTCGTGGCGGCGCGCCACTATCTCGCGCAGCAGACCGAACAGGACGTCGGTGCTGAGGGAACGCTCG
TGCGCCTCGTCCATGATTACCGCGCTGTAGTGGTCGAGGTCGGGTTCGCGTAAACTCTCGCGCAGCAGGATACCGTCTGTCATGTATTTGATGA
CAGTGTTCTCGGACGTGCAATCCTCGAACCGAATGGCGTAGCCGACCTCGTCGCCGAGCTGGGTACCCATCTCGTCGCTCACCCTCTTCGCCAC
GGACATGGCGGCCACGCGTCTCGGCTGCGTGCACCCGATCATCCCGTACTTGCTGTACCCGTCTTCGTGCAGGTACTGGGTCAGTTGCGTCGTT
TTCCCGCTGCCCGTCTCGCCCACAATGATAACGACCGAATTCTCCCTGATTACGTTCAGCAATTCCTGCCTGACGGCGAACACCGGCAGGTAAC
GTCTCTGTTCTGCTATCGACTTCTTGCGCGCGAACTCGCTCGAGGCCTCGCTCGTGCCTTTCATGTGCTCTGCGAACTTGTGATCCGCCTTGTA
ATCGGTGCTATCGTCCTCTTTATTGTATTTCCGGTCCTCCTCGTCCTCCTTCTTCTTAATTCCCATTATGTTGCCGATCTTCGTCCCGCCCAGT
TCCCAGTGTTTTTTCTGCGCTTTCCTTCGCTCCTTCTGCTCCCTGTAAACTCTGACCAGGTGGGAACCCTTGCGAGCCACCAGCGCCATATCCG
AGGTGGCGTCTTTGACTGGGACCACGGGCTCGGGCTGCTTCGTGAACACGATACGCCCGTCCAAGAATGGCGGTACTATATTATGCACCAGCAG
GTGAACCCTGTCGATCGATTCTTCGTCAAAGTCTTCGTTCACGTCCACCGACTGGACCACGCCCGAGGTCAACATTCTGTTCCTCTCCCACAGC
TCGTTATCCTTGTTGATCTGCCTCTGCTGCGCCGACAGCCTCTTCTTCTTCCTCTGCTCCAGTTGTTCTTCCTTTTTCTTGGTGTACTCTTCGC
TGACGCTTGAAAACGGGTTGTTCTCGTCGTCGTACCCCTCTCCCATGCTGTACCATTCGCGATCCAATCTTTTTTGCTCCTCTTCCCAGTTTTC
CCGTTCCACTGTGGCGTCCCATTTCGACGGTTCCGCGCCGGGAGTCGCTCCCGTTTTCTTCCTATCTTTCATCCAGTTGTTAAACTTGTGCGCG
GGCGTAGGCCTGGGCGTCTCGTCCCGTTTCGCATCCCGGGAACTCCTCTCGCGCTTATATGATCTATACGAGTCGTATTTCGTTTTAAAACTTC
TCTCGGACCAGTTCGTGTCCTCTTTTTTGTATGACGCAGGTGTCGGGAAATCCCACGAAGATTTTTTCGCGGGCGAAGGCTCGTCCTCGTCCTC
CCAGGAGCTTCTCGACGGGTTATCCTTCACCTTGATGTTTGGCGTTCGGGGCTCGTCTCTGAACCTCGGCGTTTCGTTCCTACGGGAACTGTAC
TTATCATTCTTTCTCTCTGACCTATCTCTACCAACTTTTTCCCCATGCCTGCCCCTATCACGTTTGTCTTTGGTGGACGCGTAAACGCCTTTCT
CTTTGTACTTGTCTTTCATGCGCTCCCGCAAACGTTCCCTCGCTTCCTTGCTCACCCCGCCTGTGTATGTGGGAGTTTCCTCGGCACTAGATCT
AAACCTGCGGCCGTCTTTTATTAAATTGCGGCCCTCGTACGCGTTCTCCGCGGCGACCTCGTCGTCCATCGAAAAGGACATTTTTCTGGCCGTC
TCCTCTTTCTCCCTACGCTTGGCCGCCGCCAAACGGTCCAACCCCAGCAGCGACACCTGGGGCACCTTGAAAGTGCTGGTGGTTTTCTTTTTTA
CGACAAGACCGCCTTTCTCGCTACCCGTCGTTCCCTCGAGACGGTGCAGGCCCGCTTCGTCGTCCATTATTAGGGGGTTTACTTTATGGATAC
CTTAAGATAGAAGACAAACTTTTTTTTTTGGTTGGAAAATTACTCCTTCCTTTACTGCCGAACATGCCTAATAGTTATAAAATAATTAGTTTTG
ACATACGTTCAGTTTCTATTTCTATTTTTTAAAAATATTTACGGCAGCAGCAACAAGCTCCGTGCACATATGATTTATAATTCTTCTTCTTTTT
GCCTTATCGGCCAAAGTGCTTTGATCATAAATTGCAAGAATAAAGGAAATAAACTAGAATTTGGTCCAGTGGCCCCTGATTAATGGGCCAATTA
TTCGAGAAAAACGAACACAATCGACCAACTGACAAGTCTATGGTTTTCCCTTTTTACTAT
```

Protein RF -1: -5049->-1501 (1182AA)

```
MDDEAGLHRLEGTTGSEKGGLVVKKKTTSTFKVPQVSLLGLDRLAAAKRREKEETARKMSFSMDDEVAAENAYEGRNLIKDGRRFRSSAEETPT
YTGGVSKEARERLRERMKDKYKEKGVYASTKDKRDRGRHGEKVGRDRSERKNDKYSSRRNETPRFRDEPRTPNIKVKDNPSRSSWEDEDEPSPA
KKSSWDFPTPASYKKEDTNWSERSFKTKYDSYRSYKRERSSRDAKRDETPRPTPAHKFNNWMKDRKKTGATPGAEPSKWDATVERENWEEEQKR
LDREWYSMGEGYDDENNPFSSVSEEYTKKKEEQLEQRKKKRLSAQQRQINKDNELWERNRMLTSGVVQSVDVNEDFDEESIDRVHLLVHNIVPP
FLDGRIVFTKQPEPVVPVKDATSDMALVARKGSHLVRVYREQKERRKAQKKHWELGGTKIGNIMGIKKKEDEEDRKYNKEDDSTDYKADHKFAE
HMKGTSEASSEFARKKSIAEQRRYLPVFAVRQELLNVIRENSVVIIVGETGSGKTTQLTQYLHEDGYSKYGMIGCTQPRRVAAMSVAKRVSDEM
GTQLGDEVGYAIRFEDCTSENTVIKYMTDGILLRESLREPDLDHYSAVIMDEAHERSLSTDVLFGLLREIVARRHDLKLIVTSATMDSSKFSMF
FGNVPTFTIPGRTFPVEVLFSKNAVEDYVDAAVKQALQIHLQPPSGDILIFMPGQEDIEVTCEVLAERLAEIDNAPELSILPIYSQLPSDLQAK
IFQRSPEGIRKCVVATNIAETSLTVDGIIFVIDSGYCKLKVYNPRIGMDALQIYPISQANSNQRSGRAGRTGPGQAFRLYTERQYKDELLVTTV
PEIQRTNLANTVLLLKSLGVQDLLQFHFMDPPPQDNILNSLYQLWILGALDHTGVLTKLGRQMAEFPLDPPQCQMLIVSTQMECTAEILIIVSM
LSVPSIFYRPKGREEESDGVREKFQVPESDHLTFLNVYNQWRQNNYSSHWCNEHFIHVKAMRKVREVRQQLKDILVQQKFEVKSCGTDWDVVRK
CICSAYFHQAARLKGIGEYVNCRTGMPCYLHPTSALFGLGNTPDYVVYHELVMTAREYMQCVTSVDGHWLAELGPMFFSLKETGKSGRAKKKQA
AEHLLEMENQMQVAQEEMRARKEAAEKKEAAMHKGQEIVTAGTTPRRTPARFGL
```

Comparison with *Tribolium* PREDICTED: similar to pre-mRNA splicing factor ATP-dependent RNA helicase PRP16 (1186AA)

```
Query  1    MDDEAGLHRLEGTTGSEKGGLVVKKKTTSTFKVPQVSLLGLDRLAAAKRREKEETARKMS  60
            M+ E  LHRLEG +  +KGGL+VKKK    TFKVPQ SLLGLDRLAAAKRREKEE ARKMS
Sbjct  1    MESEENLHRLEGIS-DQKGGLIVKKKP-PTFKVPQPSLLGLDRLAAAKRREKEEAARKMS  58

Query  61   FSMDDEVAAENAYE--GRNLIKDGRRFRSSAEETPTYTGGVSKEARERLRERMK-DKYKE  117
            F+MDD    +++        KD R+FRS   ETPTYTGG+S EARERL ER+K +K KE
Sbjct  59   FTMDDNDNTDDSSSLLKEKHSKDSRKFRSPHNETPTYTGGISDEARERLIERLKSNKQKE  118

Query  118  KGVYASTKDKRDRGRHGEKVGRDRSERKNDKY-SSRRNETPRFRDEPRTPNIKVKDNPSR  176
            KGVYA+TKD+        +  +DR   ++    SS R++TPRFRDEP+TPN+  KD  S+
Sbjct  119  KGVYATTKDRHRDRDRDRERDKDRDRGRHRDRESSHRSKTPRFRDEPKTPNLGHKDEISK  178

Query  177  SSWEDEDEPSPAKKSSWDFPTPASYKKEDTNWSERSFKT-KYDSYRSYKRERSSRDAKR-  234
            SSW+D+D+  P+KKSSWDFPTP++YK    +WSERS K+ KYD  +     RSSR++KR
Sbjct  179  SSWDDDDDVGPSKKSSWDFPTPSTYKGSGGDWSERSTKSRKYDESK-----RSSRESKRR  233

Query  235  ---DETPRPTPAHKFNNWMKDRKKTGATPGAEPS---KWDATVERENWEEEQKRLDREWY  288
               DE+ R TPAHK+N+W KDRK++GATP        KWD TV+RE WEEEQKR+DREWY
Sbjct  234  KYEDESARFTPAHKYNSWAKDRKRSGATPMPGKDGVIKWDNTVDRELWEEEQKRIDREWY  293

Query  289  SMGEGYDDENNPFSSVSEEYTKKKEEQLEQRKKKRLSAQQRQINKDNELWERNRMLTSGV  348
            +M EGYDD NNPFSSVSEEYTKKKEEQLEQRKKKRLSAQQRQINKDNELWERNRMLTSG
Sbjct  294  NMDEGYDDGNNPFSSVSEEYTKKKEEQLEQRKKKRLSAQQRQINKDNELWERNRMLTSGA  353

Query  349  VQSVDVNEDFDEESIDRVHLLVHNIVPPFLDGRIVFTKQPEPVVPVKDATSDMALVARKG  408
            V S+D NED+DEESIDRVHLLVHNIVPPFLDGRIVFTKQPEPV+PV+D TSDMA+V+RKG
Sbjct  354  VHSIDFNEDYDEESIDRVHLLVHNIVPPFLDGRIVFTKQPEPVIPVRDPTSDMAIVSRKG  413

Query  409  SHLVRVYREQKERRKAQKKHWELGGTKIGNIMGIKKKEDEEDRKYNKEDDSTDYKADHKF  468
            SHLVRVYREQKER+KAQKKHWELGGTKIGNIMGIKKKEDEED+++NKEDD+ DYK D KF
Sbjct  414  SHLVRVYREQKERKKAQKKHWELGGTKIGNIMGIKKKEDEEDKRFNKEDDTADYKTDQKF  473

Query  469  AEHMKGTSEASSEFARKKSIAEQRRYLPVFAVRQELLNVIRENSVVIIVGETGSGKTTQL  528
            AEHMK T EASS+FA+KK+I EQRRYLPVFAVRQELLNVIRENSVVIIVGETGSGKTTQL
Sbjct  474  AEHMKST-EASSDFAKKKTILEQRRYLPVFAVRQELLNVIRENSVVIIVGETGSGKTTQL  532

Query  529  TQYLHEDGYSKYGMIGCTQPRRVAAMSVAKRVSDEMGTQLGDEVGYAIRFEDCTSENTVI  588
            TQYLHEDGYSKYGMIGCTQPRRVAAMSVAKRVSDEMGTQLGD+VGYAIRFEDCTSENTVI
Sbjct  533  TQYLHEDGYSKYGMIGCTQPRRVAAMSVAKRVSDEMGTQLGDDVGYAIRFEDCTSENTVI  592

Query  589  KYMTDGILLRESLREPDLDHYSAVIMDEAHERSLSTDVLFGLLREIVARRHDLKLIVTSA  648
            KYMTDGILLRESLREPDLDHYSAVIMDEAHERSLSTDVLFGLLREIVARRHDLKLIVTSA
Sbjct  593  KYMTDGILLRESLREPDLDHYSAVIMDEAHERSLSTDVLFGLLREIVARRHDLKLIVTSA  652

Query  649  TMDSSKFSMFFGNVPTFTIPGRTFPVEVLFSKNAVEDYVDAAVKQALQIHLQPPSGDILI  708
            TMDSSKFSMFFGNVPTFTIPGRTFPVE+LFSKN VEDYVDAAVKQALQIHLQPPSGDILI
Sbjct  653  TMDSSKFSMFFGNVPTFTIPGRTFPVEILFSKNPVEDYVDAAVKQALQIHLQPPSGDILI  712

Query  709  FMPGQEDIEVTCEVLAERLAEIDNAPELSILPIYSQLPSDLQAKIFQRSPEGIRKCVVAT  768
            FMPGQEDIEVTCEVLAERLAEI+NAPELSILPIYSQLPSDLQAKIFQRSPEGIRKCVVAT
Sbjct  713  FMPGQEDIEVTCEVLAERLAEIENAPELSILPIYSQLPSDLQAKIFQRSPEGIRKCVVAT  772

Query  769  NIAETSLTVDGIIFVIDSGYCKLKVYNPRIGMDALQIYPISQANSNQRSGRAGRTGPGQA  828
```

```
             NIAETSLTVDGIIFVIDSGYCKLKVYNPRIGMDALQIYPISQAN+NQRSGRAGRTGPGQA
Sbjct  773   NIAETSLTVDGIIFVIDSGYCKLKVYNPRIGMDALQIYPISQANANQRSGRAGRTGPGQA  832

Query  829   FRLYTERQYKDELLVTTVPEIQRTNLANTVLLLKSLGVQDLLQFHFMDPPPQDNILNSLY  888
             FRLYTERQYK+ELLVTTVPEIQRTNLANTVLLLKSLGVQDLLQFHFMDPPPQDNILNSLY
Sbjct  833   FRLYTERQYKEELLVTTVPEIQRTNLANTVLLLKSLGVQDLLQFHFMDPPPQDNILNSLY  892

Query  889   QLWILGALDHTGVLTKLGRQMAEFPLDPPQCQMLIVSTQMECTAEILIIVSMLSVPSIFY  948
             QLWILGALDHTGVLTKLGRQMAEFPLDPPQCQMLIVS+QM CTAEILIIVSMLSVPSIFY
Sbjct  893   QLWILGALDHTGVLTKLGRQMAEFPLDPPQCQMLIVSSQMGCTAEILIIVSMLSVPSIFY  952

Query  949   RPKGREEESDGVREKFQVPESDHLTFLNVYNQWRQNNYSSHWCNEHFIHVKAMRKVREVR  1008
             RPKGREEE+DGVREKFQVPESDHLT+LNVYNQW+QN YSSHWCNEHFIH+KAMRKVREVR
Sbjct  953   RPKGREEEADGVREKFQVPESDHLTYLNVYNQWKQNKYSSHWCNEHFIHIKAMRKVREVR  1012

Query  1009  QQLKDILVQQKFEVKSCGTDWDVVRKCICSAYFHQAARLKGIGEYVNCRTGMPCYLHPTS  1068
             QQLKDILVQQK E+KSCGTDWD+VRKCICSAYFHQAARLKGIGEYVNCRTGMPC+LHPTS
Sbjct  1013  QQLKDILVQQKLEIKSCGTDWDIVRKCICSAYFHQAARLKGIGEYVNCRTGMPCHLHPTS  1072

Query  1069  ALFGLGNTPDYVVYHELVMTAREYMQCVTSVDGHWLAELGPMFFSLKETGKSGRAKKKQA  1128
             ALFGLG+TPDYVVYHELVMTAREYMQCVT+VDGHWLAELGPMFFSLKETGKSGRAKKKQA
Sbjct  1073  ALFGLGSTPDYVVYHELVMTAREYMQCVTAVDGHWLAELGPMFFSLKETGKSGRAKKKQA  1132

Query  1129  AEHLLEMENQMQVAQEEMRARKEAAEKKEAAMHKGQEIVTAGTTPRRTPARFGL  1182
             AEHL EMENQMQVAQEEMRARKEAA+K+EAAM+KGQEIV+AG TPRRTPARFGL
Sbjct  1133  AEHLQEMENQMQVAQEEMRARKEAADKREAAMNKGQEIVSAGATPRRTPARFGL  1186
```

Graphical representation

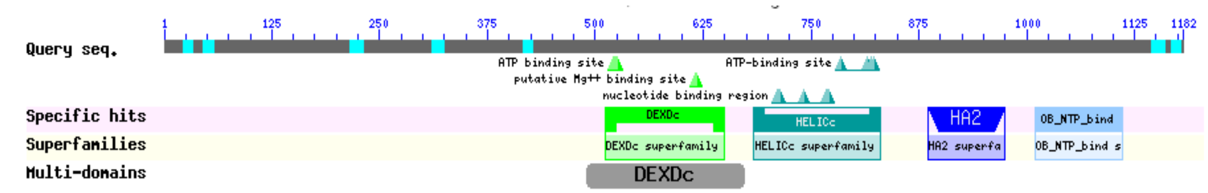

Supplementary data 3: dsRNA sequences and primers

ATPsynβ: F1 ATP synthase beta subunit, nucleotide-binding domain

Cb.comp41221_c1_seq1 len=1867

370bp
TGTCGCAGCCTTTCCAAGTAGCCGAGGTCTTCACGGCCACGCCGGTAAATTGGTACCCCTGGAGGAAA
CAATCAAAGGATTCCAAAGAATTCTGGGCGGAGATTACGACCATCTCCCCGAGGTTGCGTTCTACATGG
TCGGCCCTATCGAAGAGGTGGTACAGAAAGCCGAAAAATTGGCGGAACAGTCGTAAATCATATTGTTCA
TTAAAGGTTTAGAAAATTCCACCCTGTAAGAGGAGGTGTTGCCCTGAATCAGTAAATGACCTTTTTTTTC
AAAGTTGTTTTTTTTACGTAAATGATGTAAGTTACATTAAAC
GGCAAGGCAAATCATAATATTTGTTGTAAAAATAAAGGAAACAAATTACCTA

CbVha68FP GCGTAATACGACTCACTATAGGGAGATGTCGCAGCCTTTCCAAGT

CbVha68RP GCGTAATACGACTCACTATAGGGAGATAGGTAATTTGTTTCCTTTATTTTTAC

Vha68-2: V/A-type ATP synthase catalytic subunit A.

Cb.comp43394_c2_seq1 len=2700

436bp
GATATGGCAACGATACAGGTA
TATGAGGAAACATCGGGGGTAACTGTAGGAGACCCTGTGCTGAGAACAGGAAAACCATTG
TCAGTCGAGTTAGGTCCAGGCATTATGGGCTCTATTTTTGATGGTATTCAGCGTCCATTG
AAAGATATCAATGAAATGACTCAAAGTATCTATATTCCCAAGGGTGTAAATGTACCGGCT
CTTTCAAGAACAGCGCAGTGGGAGTTTCAGCCAGTGAGCATAAAGTTGGGAGCACATTTG
ACTGGCGGCGATATTTATGGTTTGGTCCATGAAAATACATTGGTAAAACACAAAATCATT
CTACCCCCAAGAGCAAAGGGTACAGTAACCTATGTGGCAGAAACTGGAAATTACACCATT
GATGATGTTGTTTTGGAAACAGAATTTGACGGAGAGCGTACAAAATATACAATGT

CbV/AFP GCGTAATACGACTCACTATAGGGAGAGATATGGCAACGATTCAGGTA

CbV/ARP GCGTAATACGACTCACTATAGGGAGAACATTGTATATTTTGTACGCTCTCCG

Syb: synaptobrevin, isoform A

Cb.comp43845_c0_seq1 len=1306

438bp
GCACTATTGCGCCACCCTGTATGTAATCTCGTATTTCACCTTTATGTCATAAAAGGCTT
TTTCACTAATTTTGGGAATAGATGAATTTTAGATGCAAATTACAGAAAAGAAAAATATTT
CAATAAAAATCATATTAACTCTAAATTGTTAACTCTAAGTTAATCCTGCCAAAAGACTTG
ATAAAATTTAATAAATTTTTTGCAAATAAACTTAGCAAGGTGGCGAGTATGTGTAGGATA
ATATTCTCATTTGATTGGTTTGATCAGTAGCTTTTCCTAATTGCTGAAAACGCCCTATAA
CACCATGGTGTATAGAGAGTTACATTTTAAAAATATATTACAAGGACAGTCAAAATAATT
TTCAGAGATTATGAATAATAATAAATACATATATTTCTTTATATGAAATACTTACTCCTC
AAATCACAGTTAACCACC

CbSYNFP GCGTAATACGACTCACTATAGGGAGAGCACTATTGCGCCACCCTG

CbSYNFP GCGTAATACGACTCACTATAGGGAGAGGTGGTTAACTGTGATTTGAGGAG

Pfk: Phosphofructokinase

Cb.comp40959_c0_seq7 len=2635

415bp

GTCACCGCCGCTAGTGAACACGGCAATGCCCTTGCCCTTATGGGCGCCCCTTTCTATAAA
TTTAGCACCGGTCTTTTCTGTCATTGTAGCCTGTTAATTGCGAATTAAAATCGCAATTTT
TTAAGATTCGAGTATCTACGGGTAAAAATGCAAACGCAGATTGGTACACTTTTCCTCACT
TGGCAAACCACAATTTCGGCGGAGAATTCAAAATAATTATGAATTTAAGCTCCAAGGTGA
CCTCTGCACCTACCAAGTAGGTCCTGTGACCAATGCGATTTTATTGGTTATAATTCACAC
TAAGGGGGGCTCCATCTCTGGTTTATCTTGGGTACTATGCAGCAGAATGGAGTGGTCGCT
GTTCAAAATGCTCTATGGATTATAGAATTTGTTTAACAACATAAAAAATATATAT

CbPfkFP GCGTAATACGACTCACTATAGGGAGAGTCACCGCCGCTAGTGAACAC

CbPfkFP GCGTAATACGACTCACTATAGGGAGAATATATATTTTTTATGTTGTTAAAC

Adk2: adenylate kinase-2

Cb.comp23778_c0_seq1 len=1289

370bp
CGTAACTGGCGAGCCCTTAATTAGACGTTCCGACGACAATGTGGATGCTCTAAAAAAGCG
TTTAACTACATATCATAATCAAACAAAACCTCTGGTTGATTATTATCAAATTAGGGGCAT
CCACCATCGGATTGACGCTGCGCAAGCTGCCAAAAATGTATTCAGTAATATCGACGACAT
TTTTTTGAAAAAGGCTGAACGCACAAGAATAAGTTCCAGGTTGTAATTTGTTCCTAACGA
GTTGCATTTAACCATATTATTAGTTAATACCATGTTGCTTTTTGGTCATTAGTTTTCATT
TTGGGATGTGCTGGATAATTAACTCCGTCGCCAATTTTTCAGTTGAA
CTCGCACAAATATCGGCAATAAT

CbAKFP GCGTAATACGACTCACTATAGGGAGACGTAACTGGCGAGCCCTT

CbAKRP GCGTAATACGACTCACTATAGGGAGAATTATTGCCGATATTTGTGCGAG

Fak: Focal adhesion Kinase isoform D

Cb.comp44033_c1_seq1 len=4984

400bp
CAAAGTCGGCCAGTTTGACGCACCTACGGCAAAGGACGCCACGCCCAATCGCGCCTCCCC
CACTACCCGCGAGCGGAAAGAAAGAGACTCACCTATCGGAACTGACTAGGACGTTTCTCG
CGGCGATATCTCTATGTACATATTTCTTAGATTCTAAGTAGCTCAAAGCGGTTGATAGCT
GGAATGCGTACAGCAGCAACGTCGCCAGGTCCAAGACGTGCTTGTTGTTTTGCAGGTAGG
CCCTCAGCTCGCCCAACTTGGCCAGCTCCATGATGATCCACACCGGCGAGTCCGAACACA
CCCCTATCAGTTTTATGATGTGGGGGTGATCGAATTTTTGCATGATATAGGCCTCCTCGA
GGAATTTCTCGGTCGTCGCGAGGTCGGCGTCGCCCTTGCA

CbFAKFP GCGTAATACGACTCACTATAGGGAGACAAAGTCGGCCAGTTTGACGC

CbFAKRP GCGTAATACGACTCACTATAGGGAGATGCAAGGGCGACGCCGACCT

γ-cop: gamma-coatomer protein, isoform C

Cb.comp41918_c0_seq1 len=3741

400bp
AGGCGTGGCCGAGCTTATAAC
ATCTGCGGTGGGTTAAGTGAAATAGTACTAAATCTTATCAATGTAATTGTTTTATTTAC
AACTAGGATTAAAAATCAGTATAAAAATTAAATTTAATATTTACAGTTCATCAGTCGTTT
GAATTGAACTTTGCCCTTAGTTCCTTTCTATATACTGTATTGACCGTCCTGGACTTTAGC
AATTTCGTCTTTAAATTGTTGAATGGTCTCCTTGGCTAATTTCAGTTTGGTTTTTGCAAT
AGCTATTTCTGCTTTTATTTTTTGTTTGGCTTCAATGATTACTTTCAAAGTTTTATCTTT
GCGATCTTCAAAGTCGGTATTATGTTCCCATCGGCCCAACGTTAAAGGTTTAGTAT
TTGTCAATAAATGGTGCATAAGTT

CbGCoaRP GCGTAATACGACTCACTATAGGGAGAAGGCGTGGCCGAGCTTATAAC

CbGCoaRP GCGTAATACGACTCACTATAGGGAGAAACTTATGCACCATTTATTGACAA

δ-cop: delta-coatomer protein, isoform A

Cb.comp25168_c0_seq1 len=2341

417bp
GCCCTTGGGGCATTGAATTCCAATG
ATCCCGTTTTATTCCCCGGATCTATAAGGGGTAAACTCCAAACGAGCTGATTGCGCTTGA
TCTCGTGTGTGTATGTACCATCACATTCGCCCACCACCGGAGAACAATTGATTGGCAACG
GAATATGAACATTAACATCTGCCAATTCCAAATTCGCGTGAGCGAGTTCGTACTCGATAT
TAACATCGCAGCTACCGTCGCCAGCCTCTGACGGCCAGCAGTTGATCAGCAGCGGAACGA
ACGATTCTTCGAGGCTCTGCAGCCGCCATTTCAACACACCAACGTCGGTATGCAATGGGA
ACGGCTTGGAAGGATGCTTTAATCCAATCTGCGACCGTAATTTGAACAGTTCTTTGTCAA
CGTTTGGGTGGGTTTGCAATTGAACGCCTCGC

CbDCoaFP GCGTAATACGACTCACTATAGGGAGAGCCCTTGGGGCATTGAATTCC

CbDCoaRP GCGTAATACGACTCACTATAGGGAGAGCGAGGCGTTCAATTGCAAACC

α-cop: Alpha-coatomer protein, isoform A

Cb.comp39348_c0_seq1 len=3957

400bp
AATCAAATTGGCCTTTACTGACCGTTAGCAGGGGCTATTT
TGAAAATACCGCAGCTGCGGCCACAGCAGCGAACAAATCACTAATGGCAGACCCAACCAT
CGATACCGGAATGGAAGAAGCAGGTGGTTGGGGCGACGAAGATGAACTAGAAATAGACCA
AGAGGAAAAGAAAGGGGTCGCTGCCGGCTCATCCGGGGAAGGTGAAGCGGGATGGGACGT
TGAAGATGCAGATCTCGAAATTCCCGACCTTGGACCTGCGCAGGCGCCAGAAGCCAGTGA
TAGCTATGTTCATCTTCCTGCTCAGGGTCCTTCACCAAGGCTTAGCTGGACTAAATCTTC
TCAATTAGCTGCCGATCATATCGCGGCAGGATCGTTTGAATCAGCTTGCAGGCTTCTTCA

CbACoaFP GCGTAATACGACTCACTATAGGGAGAAATCAAATTGGCCTTTACTGACCG

CbACoaRP GCGTAATACGACTCACTATAGGGAGATGAAGAAGCCTGCAAGCTGATTC

Taf1: TBP-associated factor 1, isoform

Cb.comp42070_c0_seq1 len=6171

380bp
GAAAAGTCCTCGGCTGAAGG
AGACTTTATATCAAATTCAGTCGAAACATGTTTAACATCTGAATTATTATCTAGCATAGA
TCCTCTACTATCTAGATCATAGTCAATGTCTGTTTCATCTTGGATTGTTTCTTCTCCAAT
CATTTCTTTTAGGAATGATCCAAGTGCCAACTTCCCAAGAGCGGCTAACTGTTTTTGGGA
CTCCCAATCTAGAATATCGCTTTCAAGTTTTCCGGATTCGTCGATATTACCGAAAAGAAA
TCCCATCAGATTAACCGATGGGTTATCTTCCATACTTTCGTCGTCGCTATCGCCCATTTT
AACAAATAAAAAATATTTGTTTTTAAAAAGACAGTTATAATTGTTGACACATAGAAAAT

CbTBPFP GCGTAATACGACTCACTATAGGGAGAGAAAAGTCCTCGGCTGAAGG

CbTBPRP GCGTAATACGACTCACTATAGGGAGAATTTTCTATGTGTCAACAATTATAAC

L(2)NC136: lethal (2) NC136, isoform B

Cb.comp15262_c0_seq1 len=590

400bp
TCAATTAAGGTCTCTGTCCTCCAGGTATTTATATTCAAAGGTGAAGCCCTCCTTCTTGCGCTGGCCCCACT
TTTCGTAGTCGAAATAGATGTACGTGCCCTGCTCGTACTCCTCGTTGATGATCTTGGGCTCCTCGTGCCG
CTGGAACCACATCATGTACTTGGTGTGGAAGCGCCAACTCTGCTTCTTGAGCGCCTTCGCGGCCAGGTAC
TGCGCCTTGGTGCCCTCCATGTAGTAGAAGACGAAGAAGAGGGTCTCGGTGCCCAGGCGCTGGTAAAA
CTCGAGGGTGTCCGAGTGCGCGAGCGGCGCCTGGATGTAATACGCGGGGGTGTTGTACGGGTTCCGCG
GCAGGTACGTCCGGATCCGTTCCGAGTCGGACGGG TGCGGCAGGTGGTAGAAC

CbLetFP GCGTAATACGACTCACTATAGGGAGATCAATTAAGGTCTCTGTCCTCC

CbLetRP GCGTAATACGACTCACTATAGGGAGAGTTCTACCACCTGCCGCA

Prosα2: Proteasome 20kD subunit

Cb.comp23391_c0_seq1 len=2482

400bp
TGGTCCAAATTGAGTACGCGTTGGCCGCTGTAGCTGCGGGAGCTCCTTCTGTGGGAATTA
AAGCTTCAAACGGCGTCGTTATCGCCACCGAGAATAAACACAAATCGATACTTTACGATG
AACACAGTGTTCACAAAGTGGAAATGATCACAAAGCATATAGGCATGGTGTACTCCGGCA
TGGGCCCCGATTATCGTCTGTTAGTTAAGCAAGCGCGCAAAATGGCACAGCAGTACTATC
TTGTTTACCATGAACCCATTCCAACTGTTCAGCTAGTTCAGAGAGTAGCTGCCGTTATGC
AAGAATATACTCAGTCGGGTGGCGTTAGACCGTTTGGTGTGTCACTTTTGATCTGTGGAT
GGGACAACGACAGACCCTATCTGTTCCAATGCGACCCTTC

CbPro20FP GCGTAATACGACTCACTATAGGGAGATGGTCCAAATTGAGTACGCG

CbPro20RP GCGTAATACGACTCACTATAGGGAGAGAAGGGTCGCATTGGAACAG

Dpit47: DNA polymerase interacting tpr containing protein of 47kD, isoform B

Cb.comp38762_c0_seq1 len=1569
400bp
ATTTTGTCGCAGAATTCGACGGCCTTGTCAAACTGTAATA
TTTCCAAGCAACAGTAGGCCGCGCGGTTTAGCGCCTTCTTGTAATCCGGCTTAACTTTTA
AAGCCAACTCGCAGTCTCTTAAAGAGGACCTGTAGTTTTTTAAAAACCAATGCGCGGCCG
CTCTGTTGTTAAACAAAGTCGCCTCAATTTCTGGATCACCGCACTTTTGCTTAATCCCCT
CCGTATAAGCGACTACCGCGAGTCTATAGTTCTTATGCTTGAAATTAAAATTACCGTCTT
CCTTATATGACGTTGCCAATTCGTGCGGTTCATTCTCTTCAGGATCATATTTTAATTTCT
GCAAGCCTTCGATCAATGGATGCAGTTCGTCTCCAGGTTTGGGGGGTTCCTTCATGAAAA

CbTPRFP GCGTAATACGACTCACTATAGGGAGAATTTTGTCGCAGAATTCGACGG

CbTPRRP GCGTAATACGACTCACTATAGGGAGATTTTCATGAAGGAACCCCCC

AP-2α: alpha-Adaptin, isoform A

Cb.comp40918_c0_seq1 len=8659

440bp
ATTATTGGTGTGGTTGGCGACAGGGGTCGGGCTCTTATTCTCTTTAACTTCATTCTCTGGTACCCTGCCCG
GTTTTTTCTTTTTAAGCACCGCCAAAATAGAACTCTCCCTTTCTGGGAAAGCAGGCATTTCTTCCAGCACA
GTGGCAAGAACATCTGGACTCGCAATGATACTCAGCTGAAGATATTCGGAAGCTCGCTGTTGCAGTTCA
GCATCCGCTGAGCGCAAATTGCTATCTTGTTTGAATACTTCTTGAACTTGGGTTCTTATTTCCGGGAACAA
ATTTATAAATTTGATGTATGTAGATAGTAACAAGGCCC
TGGTCATGGGTGAGCATAAATGATATTTCGAATGTAGCAATTGAAACTGGACTGCTGGTG
ATGACCTTTGATCGCCGGCAATTAGATTACCGAATTCACCTAAAATATAACCTCCCACTT

CbADAFP GCGTAATACGACTCACTATAGGGAGAATTATTGGTGTGGTTGGCGACA

CbADARP GCGTAATACGACTCACTATAGGGAGAAAGTGGGAGGTTATATTTTAGGTG

Mad1: mitotic arrest deficiency 1

Cb.comp43551_c0_seq4 len=3097

420
CTCGTCGAAGCCCGAAACAT
AATCTTAAGCTTAGAAAACCGCGTATCCCAAATGCACAACATTCGCAAAGAGATGCAGCT
CGTGTTCGAGAGCGAAACACAGGCCCTGAAAAGACAACAGGAAAATGATAGGAGGTCGAT
CGAGGAACTCGAGCAGCAGATGCAGATCGTTAGGAAGCGCGAGTCGCAATTCAAGCACGA
CCTCGCCGAGCTCAAAGACAAATACGACGACCTGAAATCGAGCAGCGAAGAACAGATTTC
GAGGTTACAAAAAGACATATCGGCCATCAAGGACGAAACGCAGGACGCGCAGCTCGAGGA
AAACATCGAATCGTCGAAACTAAAACGGCGAATAATGGAACTCGAGTCCGTGTTGAGGGC
CGCCCAAGAAGACGCCGACTCTCAGAAGAAACTGGCGTCC

CbMadFP GCGTAATACGACTCACTATAGGGAGACTCGTCGAAGCCCGAAACAT

CbMadRP GCGTAATACGACTCACTATAGGGAGAGGACGCCAGTTTCTTCTGAG

Lwr: lesswright (Ubiquitin conjugating enzyme E2)

Cb.comp44757_c0_seq1 len=1786

410bp
GCTTAGCGGAAGAAAGGAAAGCATGGAGAAAAGATCATCCATTTGGATTT
GTAGCTCGGCCATCAAAAAATCCTGACGGGTCCTTAAATTTAATGAACTGGGAATGTTCA
ATCCCTGGTAAGAAGGGAACGCCATGGGAAGAGGGACACTACAAACTACGTATGCTCTTC
AAGGAAGATTATCCTACCAGTCCACCCAAGTGCAAATTTGAACCGCCTTTGTTTCATCCT
AATGTATATCCATCGGGTACTGTTTGTTTATCTTTGCTGGATGAGGAAAAAGATTGGCGT
CCCGCCATTACTATAAAACAAATTTTATTGGGTATTCAAGATTTATTAAACGAACCGAAC
GTAAAGGATCCTGCTCAGGCCGAGGCCTACACAATCTATTGCCAAAATCGTTTAGAGTAT

CbUBFP GCGTAATACGACTCACTATAGGGAGAGCTTAGCGGAAGAAAGGAAAG

CbUBRP GCGTAATACGACTCACTATAGGGAGAATACTCTAAACGATTTTGGCAAT

Rpl135: RNA Polymerase I 135kD subunit

Cb.comp42515_c0_seq2 len=2951

400bp
ACCGGTCCCACCGATATTACAACGAGGCAGCCAATTAAAGGTCGAAAAAG
AGGTGGAGGTGTTCGTTTTGGTGAAATGGAACGAGACGCTTTGATTAGTCATGGTTCGCC
GTTTCTTTTGCAGGATCGACTACTAAATTGTTCCGATAAGACAACAGTTTCCATTTGCAC
TTCTTGTGGTACTATATTAGGACCAATCAGGATTATATCCAGGAGAGCTGATAAACCCCA
AATGTCGGAGAAGAGGGATACATGTCAGTTTTGTGGTCATGGTAGGAATGTTTCAACCAT
TCAGATTCCATACATATTTAAATATTTCGTTACGCAGTTAGCGAGCTGCAATATTAACGT
CAAAATTGAATGTAGAGAGGTATGATATGTAAGAATAAAGTGTCTGCAAA

CbRPFP GCGTAATACGACTCACTATAGGGAGAACCGGTCCCACCGATATTACA

CbRPRP GCGTAATACGACTCACTATAGGGAGATTTGCAGACACTTTATTCTTACAT

e-IF4a: Eukaryotic initiation factor 4a

Cb.comp35382_c0_seq1 len=1803

400bp
AAAACCATCTGCAATTCAACAGAGGGCTAT
AATACCTTGTGTCAAAGGGCATGATGTTATAGCTCAAGCACAGTCAGGTACTGGAAAGAC
AGCTACATTTTCCATATCAATTCTTCAACAAATTGATACATCAGTAAGGGAGTGTCAAGC
CCTTATTTTGGCACCTACTCGTGAATTAGCCCAACAAATTCAGAAAGTAGTTATTGCCTT
GGGAGATTTTATGTCTGCCCAATGTCATGCCTGCATTGGTGGAACCAATGTTAGGGAGGA
TATGAGAAAATTAGAAACTGGGGTACATGTAGTTGTAGGAACTCCTGGTCGAGTATATGA
TATGATTACTAGACGGTCACTAAGAACAAGCCACATAAAAATGTTTGT
GTTAGATGAAGCAGATGAAATG

CbRHFP GCGTAATACGACTCACTATAGGGAGAAAAACCATCTGCAATTCAACAGAG

CbRHRP GCGTAATACGACTCACTATAGGGAGACATTTCATCTGCTTCATCTAAC

RpS13: Ribosomal protein S13e

Cb.comp44615_c0_seq1 len=612

393bp
GCTTGCGAATAGCAACAGCTTTCTTAATCAAATAATACAAATCTTCGGGCAAATCAGGCGCC
AAACCCACAGCTTTCATGATGCGCAAAATTTTGTTGCCAGTAACAAACCTCACTTGTGCAAC
GCCATGAGAATCCCTTAGGATCACACCGATTTGCGAGGGAGTGAGACCCTTTTTACCCCATT
TAACTATTTGCTCCTTTACCTCTTCAGGAGTTACTTTTAACCAGGTAGGTACACTTCGTCTA
TATGGTAAAGCCGATTGAGCTATACCTTTCCCTGGGGCATGCATACGACCCATTTTGTAAGT
TTGTTGAAAAACTTGGGAACCGAAACCAACTCCAGGAAATAAAATACGAAAGAAATTGACAG
AAAGGAAATTGGTCTTTCCTG

CbS13FP GCGTAATACGACTCACTATAGGGAGAGCTTGCGAATAGCAACAGCTTTC

CbS13RP GCGTAATACGACTCACTATAGGGAGACAGGAAAGACCAATTTCCTTT

DNA pol-α50: DNA Polymerase alpha 50KD

Cb.comp31213_c0_seq1 len=2119

430bp

TCAGCAATTGCGGATCTAACATTATCATCCCAGTTTTGTGCTTGGACATCACATATCCAAC
AATGAATACCCCGTCTTCCTGAAAATATCCATAAAATGTGTTTAAATCCAAAATCTTCTC
GCAATGAAGCTTCTAGGATTTTGCTTGCAATCGCCATAAATTTCCAGCATTTGTAACACA
CATCAGCTCCAGAACAGCAAGTCCTAACTTCGTCATAATCTGTCATATCAATATCAAATA
CGATTTCCTTACTCACGGGTGATAATACACCCAGTGGTGTCCGGTCTTTTGGTTTTGATT
TATATATAGCCCCTATATCGATTTTCACAGGGAACTTTTTGTACAGTTCATTAACAAACT
CCTCATGAGAACTGAAGGATAAAAATCGAATATATATATC
GCCGATGAGAGTAAATGAGATTTCTCTTC

CbDPFP GCGTAATACGACTCACTATAGGGAGATCAGCAATTGCGGATCTAAC

CbDPRP GCGTAATACGACTCACTATAGGGAGAGAAGAGAAATCTCATTTACTCTC

Atpα: vATPase A

284bp

TCAGCGTCCATTGAAAGATATCAATGAAATGACTCAGAGTATCTATATTCCCAAGGGTGTAAATGTACCG
GCTCTTTCAAGAACAGCGCAGTGGGAGTTTCAGCCAGTGAGCATAAAGTTGGGAGCACATTTGACTGGC
GGCGATATTTATGGTTTGGTCCATGAAAATACATTGGTAAAACACAAAATCATTCTACCCCCAAGAGCAA
AGGGTACAGTAACCTATGTGGCAGAAACTGGAAATTACACCATTGATGATGTTGTTTTGGAAACAGAAT
TTGACG

CbATPα GCGTAATACGACTCACTATAGGGAGATCAGCGTCCATTGAAAGATA

CbATPα GCGTAATACGACTCACTATAGGGAGAGTTTTGGAAACAGAATTTGACG

Atpd: vATPase D

206bp

TGGAGGCCATTCATGTTGCTTCAACCCCAGCTGAATTGTATAATGCGGTGTTAGTTGATACACCTCTTGCT
CCATTCTTTGTTGATTGCATTAGTGAACAGGATTTAGATGAAATGAACATTGAGATTATCCGTAACACTTT
GTACAAAGCATACTTGGAAGCATTTTATGATTTTTGCAAGGAGATTGGTGGTACTACCGCTGAA

CbATPd GCGTAATACGACTCACTATAGGGAGATGGAGGCCATTCATGTTGCT

CbATPd GCGTAATACGACTCACTATAGGGAGATTGGTGGTACTACCGCTGAA

RpL19: Ribosomal Protein L19

203bp

GGCATCTGTACCACTCACTGTACATGAAAGCTAAGGGTAATGTATTCAAAAACAAGAGGGTACTCATGG
AGTACATCCACAAGAAAAAGGCAGAGAAGGCTCGTACCAAGATGTTGCAAGATCAGGCCAATGCGAGG
AGGCAGAAAGTTAAGCAGGCCAGAGAAAGGAGAGAAGAACGGATTGCTACCAAGAAACAAGAGGTG

CbRpL19 GCGTAATACGACTCACTATAGGGAGAGGCATCTGTACCACTCACTGTA

CbRpL19 GCGTAATACGACTCACTATAGGGAGATGCTACCAAGAAACAAGAGGTG

Snf7: Snf7 (shrub ortholog)

251bp

AGGGAAACGGAAGAAATGCTGCTGAAGAAACAGGATTTTCTTGAAAAGAAAATCGACGAGTACATGAG
TGTCGCTAGGAAAAACGCGTCTAAAAACAAAAGAGTGGCTCTACAAGCTTTGAAAAAAAAGAAACGATT
AGAGAAGAACCTGCAGCAAATTGATGGGACTCTTACTACTATAGAATTGCAAAGGGAAGCGTTAGAAG
GGGCAAACACGAATACAGCGGTACTAACTACCATGAAAAATGCCGC

CbSnf7 GCGTAATACGACTCACTATAGGGAGAAGGGAAACGGAAGAAATGCT

CbSnf7 GCGTAATACGACTCACTATAGGGAGAACTACCATGAAAAATGCCGC

qPCR primers

Target genes:

	primers	sequence	Product size	Tm
prosα	Fw2	5' GTGAAGATTTGGAGTTAGATGATGC 3'	144 bp	53
	Rv2	5' TAATGTGAGATGGTTCCAGTCTTCT 3'		53
Snf7	Fw1	5' GCTCTGAAAAACGCCCACAAA 3'	150 bp	51
	Rv1	5' AAGCTCGTCCTCATCCAGGT 3'		56
Rps13	Fw2	5' ACCAAAGCAGATGCCGTACT 3'	124 bp	59.8
	Rv2	5' AGGACAGCAAGTTCCGCTTA 3'		60

Reference genes:

	primers	sequence	Product size	Tm
rpl32	Fw1	5' GAGATGTTTGGACGCACCTT 3'	282 bp	51.5
	Rv1	5' ATGGTCGCCTGTTTCTTTTG 3'		51.8
beta actin	Fw1	5' CCACCTCACTCGAAAAGAGC 3'	194 bp	51.4
	Rv1	5' GGTGTTGGCGTACAAGTCCT 3'		51.5

Supplementary data 4: Statistical data oral bioassay

30µg/mL diet

Day 14

One way ANOVA

ANOVA

mortality

	Sum of Squares	df	Mean Square	F	Sig.
Between Groups	11149,333	3	3716,444	220,779	,000
Within Groups	134,667	8	16,833		
Total	11284,000	11			

Multiple Comparisons

Dependent Variable: mortality

	(I) treatment	(J) treatment	Mean Difference (I-J)	Std. Error	Sig.	95% Confidence Interval Lower Bound	95% Confidence Interval Upper Bound
Tukey HSD	control group	cb12	-73,33333*	3,34996	,000	-84,0611	-62,6056
		cb19	-70,00000*	3,34996	,000	-80,7277	-59,2723
		cb24	-67,33333*	3,34996	,000	-78,0611	-56,6056
	cb12	control group	73,33333*	3,34996	,000	62,6056	84,0611
		cb19	3,33333	3,34996	,756	-7,3944	14,0611
		cb24	6,00000	3,34996	,343	-4,7277	16,7277
	cb19	control group	70,00000*	3,34996	,000	59,2723	80,7277
		cb12	-3,33333	3,34996	,756	-14,0611	7,3944
		cb24	2,66667	3,34996	,854	-8,0611	13,3944
	cb24	control group	67,33333*	3,34996	,000	56,6056	78,0611
		cb12	-6,00000	3,34996	,343	-16,7277	4,7277
		cb19	-2,66667	3,34996	,854	-13,3944	8,0611

*. The mean difference is significant at the 0.05 level.

Day 7

One way ANOVA

ANOVA

mortality

	Sum of Squares	df	Mean Square	F	Sig.
Between Groups	4478,250	3	1492,750	14,731	,001
Within Groups	810,667	8	101,333		
Total	5288,917	11			

Multiple Comparisons

Dependent Variable: mortality

	(I) treatment	(J) treatment	Mean Difference (I-J)	Std. Error	Sig.	95% Confidence Interval Lower Bound	95% Confidence Interval Upper Bound
Tukey HSD	control group	cb12	-49,33333*	8,21922	,001	-75,6542	-23,0125
		cb19	-45,00000*	8,21922	,003	-71,3208	-18,6792
		cb24	-32,00000*	8,21922	,019	-58,3208	-5,6792
	cb12	control group	49,33333*	8,21922	,001	23,0125	75,6542
		cb19	4,33333	8,21922	,950	-21,9875	30,6542
		cb24	17,33333	8,21922	,229	-8,9875	43,6542
	cb19	control group	45,00000*	8,21922	,003	18,6792	71,3208
		cb12	-4,33333	8,21922	,950	-30,6542	21,9875
		cb24	13,00000	8,21922	,439	-13,3208	39,3208
	cb24	control group	32,00000*	8,21922	,019	5,6792	58,3208
		cb12	-17,33333	8,21922	,229	-43,6542	8,9875
		cb19	-13,00000	8,21922	,439	-39,3208	13,3208

*. The mean difference is significant at the 0.05 level.

10µg/mL diet

Day 14

One way ANOVA

ANOVA

mortality

	Sum of Squares	df	Mean Square	F	Sig.
Between Groups	6139,583	3	2046,528	39,231	,000
Within Groups	417,333	8	52,167		
Total	6556,917	11			

Multiple Comparisons

Dependent Variable: mortality

	(I) treatment	(J) treatment	Mean Difference (I-J)	Std. Error	Sig.	95% Confidence Interval Lower Bound	95% Confidence Interval Upper Bound
Tukey HSD	control group	cb12	-49,66667*	5,89727	,000	-68,5518	-30,7815
		cb19	-52,66667*	5,89727	,000	-71,5518	-33,7815
		cb24	-54,00000*	5,89727	,000	-72,8851	-35,1149
	cb12	control group	49,66667*	5,89727	,000	30,7815	68,5518
		cb19	-3,00000	5,89727	,955	-21,8851	15,8851
		cb24	-4,33333	5,89727	,881	-23,2185	14,5518
	cb19	control group	52,66667*	5,89727	,000	33,7815	71,5518
		cb12	3,00000	5,89727	,955	-15,8851	21,8851
		cb24	-1,33333	5,89727	,996	-20,2185	17,5518
	cb24	control group	54,00000*	5,89727	,000	35,1149	72,8851
		cb12	4,33333	5,89727	,881	-14,5518	23,2185
		cb19	1,33333	5,89727	,996	-17,5518	20,2185

*. The mean difference is significant at the 0.05 level.

Day 7

One way ANOVA

ANOVA

mortality

	Sum of Squares	df	Mean Square	F	Sig.
Between Groups	1692,250	3	564,083	6,534	,015
Within Groups	690,667	8	86,333		
Total	2382,917	11			

Multiple Comparisons

Dependent Variable: mortality

	(I) treatment	(J) treatment	Mean Difference (I-J)	Std. Error	Sig.	95% Confidence Interval Lower Bound	95% Confidence Interval Upper Bound
Tukey HSD	control group	cb12	-29,00000*	7,58654	,021	-53,2948	-4,7052
		cb19	-25,00000*	7,58654	,044	-49,2948	-,7052
		cb24	-27,66667*	7,58654	,027	-51,9614	-3,3719
	cb12	control group	29,00000*	7,58654	,021	4,7052	53,2948
		cb19	4,00000	7,58654	,950	-20,2948	28,2948
		cb24	1,33333	7,58654	,998	-22,9614	25,6281
	cb19	control group	25,00000*	7,58654	,044	,7052	49,2948
		cb12	-4,00000	7,58654	,950	-28,2948	20,2948
		cb24	-2,66667	7,58654	,984	-26,9614	21,6281
	cb24	control group	27,66667*	7,58654	,027	3,3719	51,9614
		cb12	-1,33333	7,58654	,998	-25,6281	22,9614
		cb19	2,66667	7,58654	,984	-21,6281	26,9614

*. The mean difference is significant at the 0.05 level.

1µg/mL diet

Day 14

One way ANOVA

ANOVA

mortality

	Sum of Squares	df	Mean Square	F	Sig.
Between Groups	3823,333	3	1274,444	7,908	,009
Within Groups	1289,333	8	161,167		
Total	5112,667	11			

Multiple Comparisons

Dependent Variable: mortality

	(I) treatment	(J) treatment	Mean Difference (I-J)	Std. Error	Sig.	95% Confidence Interval Lower Bound	95% Confidence Interval Upper Bound
Tukey HSD	control group	cb12	-44,33333*	10,36554	,012	-77,5274	-11,1392
		cb19	-38,00000*	10,36554	,026	-71,1941	-4,8059
		cb24	-40,33333*	10,36554	,019	-73,5274	-7,1392
	cb12	control group	44,33333*	10,36554	,012	11,1392	77,5274
		cb19	6,33333	10,36554	,926	-26,8608	39,5274
		cb24	4,00000	10,36554	,979	-29,1941	37,1941
	cb19	control group	38,00000*	10,36554	,026	4,8059	71,1941
		cb12	-6,33333	10,36554	,926	-39,5274	26,8608
		cb24	-2,33333	10,36554	,996	-35,5274	30,8608
	cb24	control group	40,33333*	10,36554	,019	7,1392	73,5274
		cb12	-4,00000	10,36554	,979	-37,1941	29,1941
		cb19	2,33333	10,36554	,996	-30,8608	35,5274

*. The mean difference is significant at the 0.05 level.

Day 7

One way ANOVA

ANOVA

mortality

	Sum of Squares	df	Mean Square	F	Sig.
Between Groups	1539,583	3	513,194	3,340	,077
Within Groups	1229,333	8	153,667		
Total	2768,917	11			

Multiple Comparisons

Dependent Variable: mortality

	(I) treatment	(J) treatment	Mean Difference (I-J)	Std. Error	Sig.	95% Confidence Interval Lower Bound	95% Confidence Interval Upper Bound
Tukey HSD	control group	cb12	-24,00000	10,12148	,161	-56,4126	8,4126
		cb19	-22,66667	10,12148	,192	-55,0792	9,7459
		cb24	-29,66667	10,12148	,073	-62,0792	2,7459
	cb12	control group	24,00000	10,12148	,161	-8,4126	56,4126
		cb19	1,33333	10,12148	,999	-31,0792	33,7459
		cb24	-5,66667	10,12148	,941	-38,0792	26,7459
	cb19	control group	22,66667	10,12148	,192	-9,7459	55,0792
		cb12	-1,33333	10,12148	,999	-33,7459	31,0792
		cb24	-7,00000	10,12148	,897	-39,4126	25,4126
	cb24	control group	29,66667	10,12148	,073	-2,7459	62,0792
		cb12	5,66667	10,12148	,941	-26,7459	38,0792
		cb19	7,00000	10,12148	,897	-25,4126	39,4126

*. The mean difference is significant at the 0.05 level.
